# Author Correction: Diversity-oriented synthesis encoded by deoxyoligonucleotides

**DOI:** 10.1038/s41467-023-43518-2

**Published:** 2023-11-21

**Authors:** Liam Hudson, Jeremy W. Mason, Matthias V. Westphal, Matthieu J. R. Richter, Jonathan R. Thielman, Bruce K. Hua, Christopher J. Gerry, Guoqin Xia, Heather L. Osswald, John M. Knapp, Zher Yin Tan, Praveen Kokkonda, Ben I. C. Tresco, Shuang Liu, Andrew G. Reidenbach, Katherine S. Lim, Jennifer Poirier, John Capece, Simone Bonazzi, Christian M. Gampe, Nichola J. Smith, James E. Bradner, Connor W. Coley, Paul A. Clemons, Bruno Melillo, C. Suk-Yee Hon, Johannes Ottl, Christoph E. Dumelin, Jonas V. Schaefer, Ann Marie E. Faust, Frédéric Berst, Stuart L. Schreiber, Frédéric J. Zécri, Karin Briner

**Affiliations:** 1 Chemical Biology and Therapeutics Science Program, Broad Institute, 415 Main Street, Cambridge, MA 02142 USA; 2 Novartis Institutes for BioMedical Research, 181 Massachusetts Avenue, Cambridge, MA 02139 USA; 3 Department of Chemistry, The Scripps Research Institute, 10550 North Torrey Pines Road, La Jolla, CA 92037 USA; 4 Department of Chemistry and Chemical Biology, Harvard University, 12 Oxford Street, Cambridge, MA 02138 USA; 5 Department of Chemical Engineering, MIT, Cambridge, MA 02139 USA; 6 Novartis Institutes for BioMedical Research, Novartis Pharma AG, Novartis Campus, CH-4002 Basel, Switzerland

**Keywords:** Drug discovery and development, Combinatorial libraries

Correction to: *Nature Communications* 10.1038/s41467-023-40575-5, published online 15 August 2023

The original version of this Article contained an error in the email address provided in the Discussion section which was incorrectly given as fast_del@novartis.com. The correct email address is fast.del@novartis.com. This has now been corrected in both the PDF and HTML versions of the Article.

