# Supplementary material for: Diversity-oriented synthesis encoded by deoxyoligonucleotides
Source: Nat Commun. 2023 Aug 15;14:4930. doi: 10.1038/s41467-023-40575-5 (PMC10427684; doi:10.1038/s41467-023-40575-5)
Supplement: Supplementary file 1 — Supplementary Information [file 41467_2023_40575_MOESM1_ESM.pdf]

Supplementary Information – Supplementary Methods, Figures, Tables, and References

Diversity oriented synthesis encoded by deoxyoligonucleotides

Liam Hudson,[1,2] Jeremy W. Mason,[1,2] Matthias V. Westphal,[1,2] Matthieu J. R. Richter,[1] Jonathan R. Thielman,[1] Bruce K. Hua,[1] Christopher J. Gerry,[1] Guoqin Xia,[3] Heather L. Osswald,[3] John M. Knapp,[1] Zher Yin Tan,[1] Praveen Kokkonda,[1] Ben I. C. Tresco,[1] Shuang Liu,[1,4] Andrew G. Reidenbach,[1] Katherine S. Lim,[1] Jennifer Poirier,[2] John Capece,[2] Simone Bonazzi,[2] Christian M. Gampe,[2] Nichola J. Smith,[2] James E. Bradner,[2] Connor Coley,[1,5] Paul A. Clemons,[1] Bruno Melillo,[3] C. Suk-Yee Hon,[1] Johannes Ottl,[6] Christoph E. Dumelin,[6] Jonas V. Schaefer,[6] Ann Marie E. Faust,[2] Frederic Berst,[6] Stuart L. Schreiber,[1,4] Frédéric J. Zécri,[2*] Karin Briner,[2]

[1] Chemical Biology and Therapeutics Science Program, Broad Institute, 415 Main Street, Cambridge, MA 02142, USA

[2] Novartis Institutes for BioMedical Research, 181 Massachusetts Avenue, Cambridge, MA 02139, USA

[3] Department of Chemistry, The Scripps Research Institute, 10550 North Torrey Pines Road, La Jolla, CA, 92037 USA

[4] Department of Chemistry and Chemical Biology, Harvard University, 12 Oxford Street, Cambridge, MA 02138, USA

[5] Department of Chemical Engineering, MIT, Cambridge, Massachusetts 02139, United States

[6] Novartis Institutes for BioMedical Research, Novartis Pharma AG, Novartis Campus, CH-4002, Basel, Switzerland

Table of Contents

1. Synthesis: off-DNA 2

1.1. β-CH arylated cyclic amino acids 2

1.2. Arylated and benzylated hydroxyproline stereoisomers 10

1.3. Spirocyclic azetidines 25

1.4. Enumerated DEL targets synthesized off-DNA 41

2. Synthesis: on-DNA 86

2.1. General protocols 86

2.2. Optimization prior to library synthesis 87

2.3. Library synthesis 101

2.4. Small scale library closure 110
2.5. Large scale library closure 112
3. Preparation of next generation sequencing libraries – Supplementary Figures........ 113
4. DEL screening – Supplementary Figures 117
5. Assay details – Supplementary Figures and Tables 117
6. In silico – Supplementary Figures 123
7. Small molecule NMR data 124
7.1. β-arylated cyclic amino acids 124
7.2. Hydroxyproline-derived skeletons 149
7.3. Spiro-azetdine skeletons 181
7.4. Enumerated DEL compounds off-DNA........ 192
8. On-DNA analytical data 255
8.1. DNA-skeleton conjugate purity 255
8.2. Amine capping building block validation data 264
8.3. Suzuki coupling building block validation data 285
9. Building block to sequence association information 307
10. Supplementary References 324

1. Synthesis: off-DNA

1.1. β-CH arylated cyclic amino acids

Synthesis followed published protocols[1] with minor adaptations as detailed below with reference to the general scheme outlined in Supplementary Fig. 1.

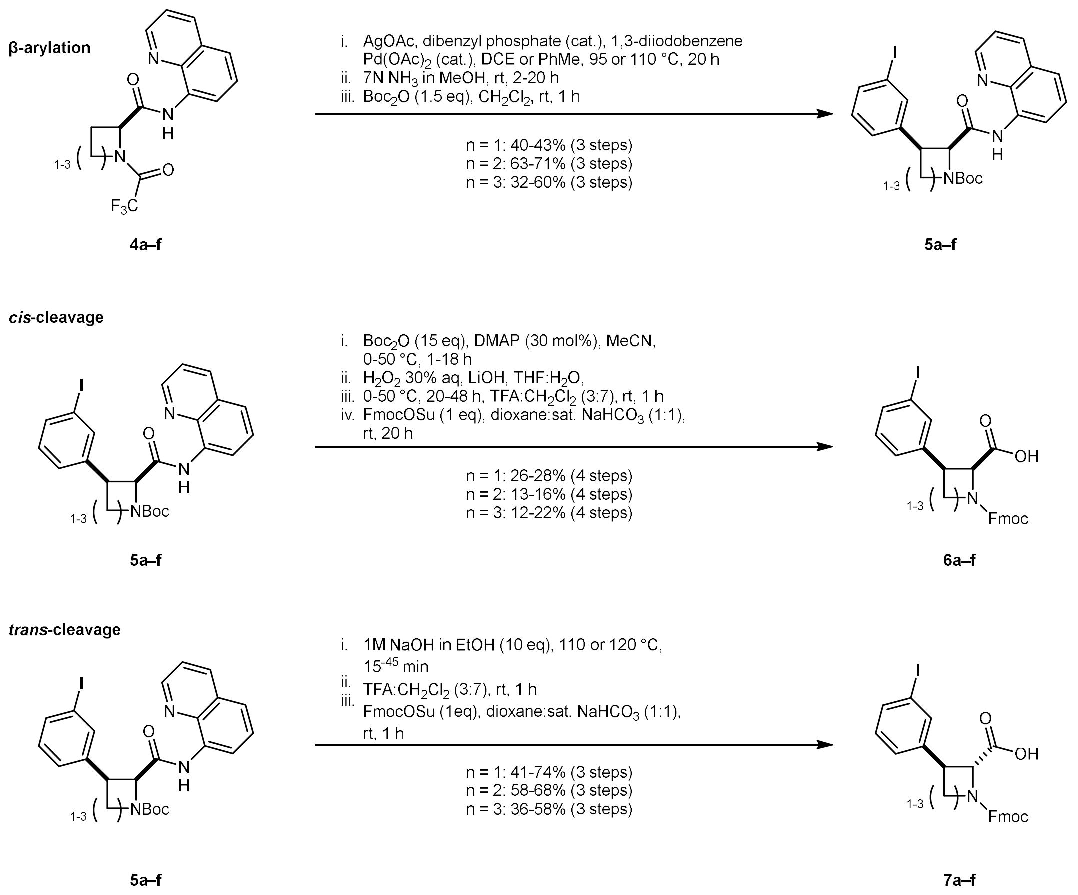

Supplementary Fig. 1 | Overview of synthetic route to meta-I-β-aryl cyclic Fmoc amino acids.

General β-arylation sequence protocol:

Arylation precursor (**4a–f**, 1 eq), dibenzyl hydrogen phosphate (0.2 eq), AgOAc (2 eq), $Pd(OAc)_2$ (0.1 eq) and 1,3-diiodobenzene (2 eq) were added to a reaction vessel (rbf or vial). Reaction solvent (DCE or toluene, 1 M) was added and the reaction vessel was evacuated of air by five cycles of vacuum and N_2 supply. The reaction vessel was placed in a preheated reaction block at 95 or 110 °C, and the mixture was stirred vigorously for 20 hr. After cooling to rt 7N NH_3 in MeOH (3 mL of solution per 0.2 mmol reactant) was added to the residue and stirred at rt until complete TFA-cleavage was observed by LCMS analysis, then removed volatiles under reduced pressure. The resulting residue was dissolved as far as possible in CH_2Cl_2 (0.1 M) before adding Boc_2O (1.5 eq) and stirred at rt until complete Boc-protection could be observed. Mono-Boc intermediate, **5a–f,** was partially purified by normal phase flash chromatography in a solvent system of 0-X% EtOAC in heptane (azetidines: X = 40; pyrrolidines: X = 30; piperidines: X = 20).

cis-cleavage:

5a–f (1 eq) was added to a reaction vial before dissolving in MeCN (0.2 M), adding Boc_2O (15 eq) and DMAP (0.1–0.3 eq). The mixture was then stirred between rt and 50 °C until complete conversion of mono- to di-Boc-protected intermediate was observed. Volatiles were removed under reduced pressure, and the deep red residue was dissolved in a 2:1 mixture of THF and water (0.2 M) before cooling to 0 °C. 30% H_2O_2 (10 eq, water) was added, followed by LiOH (6 eq), and stirring was continued at 0 °C for 1 hr, then warmed to 50 °C for 20 h. The crude reaction mixture was neutralized with 4 N HCl in 1,4-dioxane and volatiles were removed under reduced pressure. The residue was treated with TFA (as a 3:7 mixture with CH_2Cl_2; 10 mL per mmol of reactant). Upon completion of Boc-cleavage volatiles were removed under reduced pressure. To the residue was added 1,4-dioxane (5 mL per 1 mmol of reactant), saturated $NaHCO_3$ (5 mL per 1 mmol of reactant), and FmocOSu (1 eq) before stirring at rt overnight. The crude reaction mixture was diluted with 1 N aq. HCl and extracted with CH_2Cl_2 (three times). Combined organic portions were dried over Na_2SO_4, filtered, and solvent removed under reduced pressure. Crude Fmoc-protected amino acid, **6a–f**, was purified by preparative HPLC.

trans-cleavage:

5a–f (1 eq) was added to a Biotage μW vial and dissolved in a solution of NaOH (10 eq, 1 M in EtOH). The resulting solution was heated to either 110 or 120 °C for between 15 and 45 min under μW irradiation. The crude reaction mixture was neutralized with 4N HCl in 1,4-dioxane and volatiles were removed under reduced pressure. The bright orange residue was treated with TFA (as a 3:7 mixture with CH_2Cl_2; 10 mL per mmol of reactant). Upon completion of Boc-cleavage volatiles were removed under reduced pressure. To the residue was added 1,4-dioxane (5 mL per 1 mmol of reactant), saturated $NaHCO_3$ (5 mL per 1 mmol of reactant), and FmocOSu (1 eq) before stirring at rt overnight. The crude reaction mixture was diluted with 1 N aq. HCl and extracted with CH_2Cl_2 (three times). Combined organic portions were dried over Na_2SO_4, filtered, and solvent removed under reduced pressure. Crude Fmoc-protected amino acid, **7a–f**, was purified by preparative HPLC.

List of general changes to published route[1]:

1) Normal phase flash column chromatography was only performed for β-arylated N-Boc intermediates (**5a–f**), following β-arylation, TFA-protecting group removal, and mono-Boc installation.
2) All steps commencing with β-arylated N-Boc intermediates (**5a–f**) were telescoped without intermediate purification through to final Fmoc amino acids (**6a–f** and **7a–f**).
3) Target Fmoc-protected amino acids (**6a–f** and **7a–f**) were isolated by preparative HPLC.

Ring-size specific notes:

1) Directed β-arylation of piperidines performed well at slightly reduced temperatures (i.e., 90–100 °C), and it could be noted through chiral SFC analysis that high temperatures of above 110 °C

(e.g., due to hotplate instability, or from 'hot spots' if heated under μW irradiation) can lead to epimerization at the α-position.

2) Installation of the second Boc group (*cis*-cleavage pathway) was facile for azetidines under published conditions, required approximately 15 eq of Boc_2O and 10 mol% DMAP for pyrrolidines, and 15 eq of Boc_2O with 30 mol% DMAP and heating at 50 °C for >24 hr for piperidines.
3) Directing group cleavage (*trans*-configuration) was performed under μW irradiation. Azetidines required 15 min at 110 °C, pyrrolidines required 25 min at 110 °C and piperidines required 45 min at 120 °C. For all ring sizes some Boc-cleavage at the cyclic amine was observed, and the crude mixture could be progressed directly through complete Boc-deprotection and Fmoc installation prior to preparative HPLC purification.

Proton NMR data are reported at temperatures giving the greatest clarity for assignment of structure. In some cases, this may mean a temperature at which rotamers collapse to well-defined multiples; in other cases, rt proton NMR yielded clearly distinct rotamers, whereas higher temperatures lead to rotamer collapse with poor resolution.

(2*R*,3*S*)-1-(((9H-fluoren-9-yl)methoxy)carbonyl)-3-(3-iodophenyl)azetidine-2-carboxylic acid (**6a**)

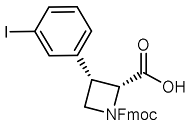

^{1}H NMR (400 MHz, DMSO+D_2O, 80 °C) δ 7.78 (d, *J* = 7.4 Hz, 2H), 7.63 – 7.51 (m, 4H), 7.33 (t, *J* = 7.6 Hz, 2H), 7.26 (q, *J* = 7.5 Hz, 3H), 7.06 (t, *J* = 7.8 Hz, 1H), 4.88 (d, *J* = 8.7 Hz, 1H), 4.24 – 4.09 (m, 6H). ^{13}C NMR (101 MHz, CD_3OD) δ 170.38, 156.69, 143.74, 141.20, 139.39, 137.05, 136.46, 129.82, 127.47, 127.29, 126.82, 124.90, 124.83, 119.54, 93.41, 67.40, 66.74, 52.24, 46.25, 36.71. HRMS (ESI$^+$) m/z calculated for $C_{25}H_{20}INO_4$ [M+H]$^+$ 526.0510; found 526.0543.

(2*S*,3*R*)-1-(((9H-fluoren-9-yl)methoxy)carbonyl)-3-(3-iodophenyl)azetidine-2-carboxylic acid (**6b**)

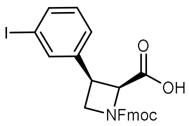

^{1}H NMR (400 MHz, DMSO+D_2O, 80 °C) δ 7.78 (d, *J* = 7.4 Hz, 2H), 7.63 – 7.51 (m, 4H), 7.33 (t, *J* = 7.6 Hz, 2H), 7.26 (q, *J* = 7.5 Hz, 3H), 7.06 (t, *J* = 7.8 Hz, 1H), 4.88 (d, *J* = 8.7 Hz, 1H), 4.24 – 4.09 (m, 6H). ^{13}C NMR (101 MHz, CD_3OD) δ 143.76, 141.20, 139.46, 137.07, 136.43, 129.81, 127.46, 127.30, 126.81, 124.91, 124.85, 119.53, 93.40, 67.42, 66.87, 52.19, 46.32, 36.72. HRMS (ESI$^+$) m/z calculated for $C_{25}H_{20}INO_4$ [M+H]$^+$ 526.0510; found 526.0532.

(2*R*,3*R*)-1-(((9H-fluoren-9-yl)methoxy)carbonyl)-3-(3-iodophenyl)pyrrolidine-2-carboxylic acid (**6c**)

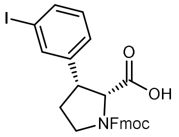

^{1}H NMR (400 MHz, DMSO+D_2O) (55:45 mixture of rotamers) δ 7.90 (app. dd, *J* = 12.3, 7.5 Hz, 2H), 7.74 – 7.62 (m, 4H), 7.48 – 7.30 (m, 5H), 7.16 (app. dt, *J* = 11.2, 7.7 Hz, 1H), 4.62 (d, *J* = 8.6 Hz, 0.45H), 4.46 (d, *J* = 8.7 Hz, 0.55H), 4.37 – 4.16 (m, 3H), 3.85 – 3.70 (m, 2H), 3.54 – 3.44 (m, 1H), 2.50 – 2.40 (m, 1H), 2.19 – 2.11 (m, 1H). ^{13}C NMR (101 MHz, CD_3OD) δ 172.95, 172.79, 155.08, 154.90, 143.91, 143.89, 143.78, 143.68, 141.27, 141.23, 141.17, 141.08, 139.53, 137.00, 136.95, 136.27, 136.25, 129.79, 129.77, 127.42, 126.85, 126.80, 124.87, 124.76, 124.72, 119.56, 119.52, 119.49, 93.47, 93.44, 67.90, 67.38, 64.08, 63.79, 46.08, 45.69, 28.05, 27.11. HRMS (ESI$^+$) m/z calculated for $C_{26}H_{22}INO_4$ $[M+H]^+$ 540.0666; found 540.0693.

(2*S*,3*S*)-1-(((9H-fluoren-9-yl)methoxy)carbonyl)-3-(3-iodophenyl)pyrrolidine-2-carboxylic acid (**6d**)

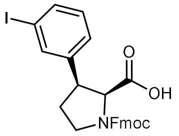

^{1}H NMR (400 MHz, DMSO+D_2O) (55:45 mixture of rotamers) δ 7.89 (app. dd, *J* = 12.1, 7.5 Hz, 2H), 7.72 – 7.60 (m, 4H), 7.46 – 7.27 (m, 5H), 7.14 (app. dt, *J* = 11.9, 7.7 Hz, 1H), 4.60 (d, *J* = 8.6 Hz, 0.45H), 4.43 (d, *J* = 8.6 Hz, 0.55H), 4.34 – 4.14 (m, 3H), 3.85 – 3.67 (m, 2H), 3.51 – 3.41 (m, 1H), 2.49 – 2.36 (m, 1H), 2.17 – 2.07 (m, 1H). ^{13}C NMR (101 MHz, CD_3OD) δ 172.89, 172.74, 155.08, 154.89, 143.91, 143.89, 143.77, 143.67, 141.27, 141.23, 141.18, 141.08, 139.51, 139.48, 136.99, 136.95, 136.29, 136.26, 129.80, 129.77, 127.41, 126.85, 126.81, 124.87, 124.76, 124.72, 119.56, 119.53, 119.50, 93.48, 93.45, 67.89, 67.39, 64.04, 63.72, 46.07, 45.69, 28.03, 27.07 HRMS (ESI$^+$) m/z calculated for $C_{26}H_{22}INO_4$ $[M+H]^+$ 540.0666; found 540.0697.

(2*R*,3*R*)-1-(((9H-fluoren-9-yl)methoxy)carbonyl)-3-(3-iodophenyl)piperidine-2-carboxylic acid (**6e**)

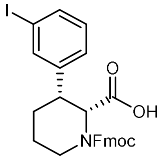

^{1}H NMR (400 MHz, DMSO+D_2O, 80 °C) δ 7.84 (d, *J* = 7.5 Hz, 2H), 7.64 (d, *J* = 7.5 Hz, 2H), 7.60 – 7.55 (m, 2H), 7.41 (t, *J* = 7.5 Hz, 2H), 7.33 (tt, *J* = 7.4, 1.6 Hz, 2H), 7.26 – 7.17 (m, 1H), 7.10 (t, *J* = 8.0 Hz, 1H), 4.83 – 4.75 (m, 1H), 4.51 – 4.40 (m, 2H), 4.28 (t, *J* = 6.4 Hz, 1H), 3.96 – 3.85 (m, 1H), 3.31 – 3.21 (m, 1H), 2.94 – 2.84 (m, 1H), 2.09 (app. qd, *J* = 13.0, 3.5 Hz, 1H), 1.85 – 1.74 (m, 2H), 1.54 – 1.41 (m,

1H). ^{13}C NMR (101 MHz, CD_3OD) (not all quaternary carbon peaks resolved) δ 143.70, 143.07, 136.41, 135.58, 129.63, 127.43, 127.04, 126.76, 124.72, 124.48, 119.70, 119.57, 93.36, 67.39, 59.03, 47.17, 43.10, 40.31, 24.66, 23.69. HRMS (ESI$^+$) m/z calculated for $C_{27}H_{24}INO_4$ $[M+H]^+$ 554.0823; found 554.0853.

(2*S*,3*S*)-1-(((9H-fluoren-9-yl)methoxy)carbonyl)-3-(3-iodophenyl)piperidine-2-carboxylic acid (**6f**)

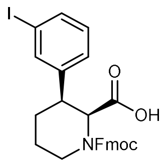

^1H NMR (400 MHz, DMSO+D_2O, 60 °C) δ 7.87 (d, *J* = 7.5 Hz, 2H), 7.65 (d, *J* = 7.4 Hz, 2H), 7.57 (d, *J* = 7.6 Hz, 2H), 7.42 (t, *J* = 7.4 Hz, 2H), 7.37 – 7.31 (m, 2H), 7.27 – 7.16 (m, 1H), 7.11 (t, *J* = 7.8 Hz, 1H), 4.85 – 4.68 (m, 1H), 4.50 – 4.35 (m, 2H), 4.29 (t, *J* = 6.5 Hz, 1H), 3.98 – 3.82 (m, 1H), 3.35 – 3.19 (m, 1H), 3.01 – 2.80 (m, 1H), 2.14 – 1.99 (m, 1H), 1.78 (app. ddd, *J* = 16.2, 12.9, 6.7 Hz, 2H), 1.46 (app. dtd, *J* = 18.2, 9.1, 4.8 Hz, 1H). ^{13}C NMR (101 MHz, CD_3OD) (not all quaternary carbon peaks resolved) δ 143.78, 136.46, 135.58, 129.63, 127.43, 127.06, 126.77, 124.71, 119.57, 93.36, 67.13, 59.42, 59.05, 43.11, 40.30, 24.70, 23.70. HRMS (ESI$^+$) m/z calculated for $C_{27}H_{24}INO_4$ $[M+H]^+$ 554.0823; found 554.0860.

(2*S*,3*S*)-1-(((9H-fluoren-9-yl)methoxy)carbonyl)-3-(3-iodophenyl)azetidine-2-carboxylic acid (**7a**)

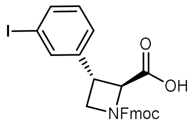

^1H NMR (400 MHz, DMSO, 80 °C) δ 7.86 (dt, *J* = 7.6, 1.0 Hz, 2H), 7.76 (t, *J* = 1.8 Hz, 1H), 7.71 – 7.64 (m, 3H), 7.45 – 7.39 (m, 3H), 7.34 (td, *J* = 7.4, 1.2 Hz, 2H), 7.22 (t, *J* = 7.8 Hz, 1H), 4.53 (d, *J* = 5.9 Hz, 1H), 4.38 – 4.33 (m, 2H), 4.31 – 4.23 (m, 2H), 3.88 – 3.83 (m, 1H), 3.83 – 3.75 (m, 1H). ^{13}C NMR (101 MHz, DMSO) (C=O signals assigned from 2D spectra) δ 144.07, 143.10, 141.18, 136.59, 136.16, 131.33, 128.18, 127.59, 127.00, 125.72, 120.57, 95.67, 67.35, 66.95, 53.17, 47.12, 38.07. HRMS (ESI$^+$) m/z calculated for $C_{25}H_{20}INO_4$ $[M+H]^+$ 526.0510; found 526.0540.

(2*R*,3*R*)-1-(((9H-fluoren-9-yl)methoxy)carbonyl)-3-(3-iodophenyl)azetidine-2-carboxylic acid (**7b**)

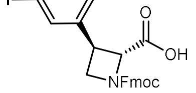

^1H NMR (400 MHz, DMSO, 80 °C) δ 7.86 (dd, *J* = 7.5, 1.1 Hz, 2H), 7.76 (t, *J* = 1.8 Hz, 1H), 7.71 – 7.64 (m, 3H), 7.44 – 7.38 (m, 3H), 7.33 (td, *J* = 7.5, 1.2 Hz, 2H), 7.22 (t, *J* = 7.8 Hz, 1H), 4.53 (d, *J* = 5.8 Hz,

1H), 4.38 – 4.33 (m, 2H), 4.30 – 4.22 (m, 2H), 3.88 – 3.82 (m, 1H), 3.82 – 3.75 (m, 1H). ^{13}C NMR (101 MHz, DMSO) (C=O signals assigned from 2D spectra) δ 144.07, 142.53, 141.18, 136.17, 135.24, 131.33, 128.18, 127.60, 127.01, 125.72, 120.58, 95.68, 66.23, 66.11, 53.21, 47.12, 37.21. HRMS (ESI$^+$) m/z calculated for $C_{25}H_{20}INO_4$ [M+H]$^+$ 526.0510; found 526.0543.

(2*S*,3*R*)-1-(((9H-fluoren-9-yl)methoxy)carbonyl)-3-(3-iodophenyl)pyrrolidine-2-carboxylic acid (**7c**)

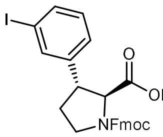

^{1}H NMR (400 MHz, DMSO) (approx. 55:45 mixture of rotamers [all peaks overlap]) δ 12.86 (s, 1H), 7.90 (app. dd, *J* = 14.4, 7.5 Hz, 2H), 7.76 – 7.60 (m, 4H), 7.47 – 7.28 (m, 5H), 7.18 (app. dt, *J* = 12.2, 7.8 Hz, 1H), 4.42 – 4.08 (m, 4H), 3.70 – 3.36 (m, 3H), 2.24 (app. ddd, *J* = 12.5, 8.5, 5.3 Hz, 1H), 2.00 (app. dt, *J* = 12.7, 7.8 Hz, 1H). ^{13}C NMR (101 MHz, DMSO) (C=O signals assigned from 2D spectra) δ 144.79, 144.18, 141.24, 136.38, 136.26, 131.30, 128.17, 127.62, 125.85, 125.62, 120.60, 95.63, 67.53, 66.70, 48.49, 47.14, 46.32, 33.60. HRMS (ESI$^+$) m/z calculated for $C_{26}H_{22}INO_4$ [M+H]$^+$ 540.0666; found 540.0701.

(2*R*,3*S*)-1-(((9H-fluoren-9-yl)methoxy)carbonyl)-3-(3-iodophenyl)pyrrolidine-2-carboxylic acid (**7d**)

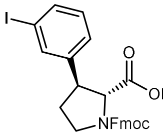

^{1}H NMR (400 MHz, DMSO) (approx. 55:45 mixture of rotamers [all peaks overlap]) δ 7.90 (app. dd, *J* = 14.4, 7.5 Hz, 2H), 7.76 – 7.60 (m, 4H), 7.47 – 7.28 (m, 5H), 7.18 (app. dt, *J* = 12.2, 7.8 Hz, 1H), 4.41 – 4.08 (m, 4H), 3.70 – 3.38 (m, 4H), 2.24 (app. dq, *J* = 12.4, 5.9, 4.2 Hz, 1H), 2.06 – 1.94 (m, 1H). ^{13}C NMR (101 MHz, DMSO) δ 173.06, 154.20, 154.11, 144.27, 144.18, 141.23, 141.11, 136.38, 136.26, 131.34, 131.29, 128.17, 127.65, 127.62, 127.01, 126.92, 125.72, 125.67, 125.63, 125.56, 120.64, 120.60, 120.54, 95.62, 67.54, 67.12, 65.89, 65.46, 49.16, 48.15, 47.14, 46.33, 40.61, 33.22, 32.21. HRMS (ESI$^+$) m/z calculated for $C_{26}H_{22}INO_4$ [M+H]$^+$ 540.0666; found 540.0701.

(2*S*,3*R*)-1-(((9H-fluoren-9-yl)methoxy)carbonyl)-3-(3-iodophenyl)piperidine-2-carboxylic acid (**7e**)

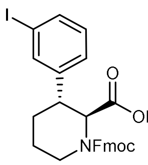

^{1}H NMR (400 MHz, DMSO, 80 °C) δ 7.86 (dt, *J* = 7.4, 1.2 Hz, 2H), 7.69 (t, *J* = 1.8 Hz, 1H), 7.65 – 7.57 (m, 3H), 7.41 (td, *J* = 7.5, 3.5 Hz, 2H), 7.34 – 7.26 (m, 3H), 7.12 (t, *J* = 7.8 Hz, 1H), 4.86 (d, *J* = 2.5 Hz, 1H), 4.50 – 4.38 (m, 2H), 4.31 (t, *J* = 6.6 Hz, 1H), 3.85 (d, *J* = 13.1 Hz, 1H), 3.53 (q, *J* = 3.9 Hz, 1H), 3.11

(td, J = 12.3, 4.2 Hz, 2H), 1.87 – 1.71 (m, 2H), 1.50 – 1.30 (m, 2H). ^{13}C NMR (101 MHz, DMSO) δ 172.53, 145.92, 144.21, 141.22, 136.65, 135.48, 130.98, 128.14, 127.61, 127.11, 125.43, 120.62, 95.53, 67.53, 57.17, 49.07, 47.11, 41.66, 38.29, 27.65, 19.29. HRMS (ESI$^+$) m/z calculated for $C_{27}H_{24}INO_4$ [M+H]$^+$ 554.0823; found 554.0859.

(2*R*,3*S*)-1-(((9H-fluoren-9-yl)methoxy)carbonyl)-3-(3-iodophenyl)piperidine-2-carboxylic acid (**7f**)

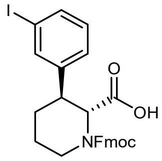

^{1}H NMR (400 MHz, DMSO, 80 °C) δ 7.86 (d, J = 7.6 Hz, 2H), 7.69 (t, J = 1.9 Hz, 1H), 7.62 (dd, J = 11.7, 7.6 Hz, 3H), 7.41 (td, J = 7.4, 3.5 Hz, 2H), 7.33 – 7.26 (m, 3H), 7.12 (t, J = 7.7 Hz, 1H), 4.86 (d, J = 2.5 Hz, 1H), 4.50 – 4.37 (m, 2H), 4.30 (t, J = 6.8 Hz, 1H), 3.85 (d, J = 13.1 Hz, 1H), 3.57 – 3.50 (m, 1H), 3.10 (dt, J = 12.5, 7.2 Hz, 2H), 1.86 – 1.71 (m, 2H), 1.49 – 1.33 (m, 2H). ^{13}C NMR (101 MHz, DMSO) δ 172.53, 145.92, 144.21, 141.22, 136.65, 135.47, 130.97, 128.14, 127.61, 127.11, 125.43, 120.61, 95.53, 67.52, 57.16, 49.07, 47.11, 41.65, 38.32, 27.65, 19.29. HRMS (ESI$^+$) m/z calculated for $C_{27}H_{24}INO_4$ [M+H]$^+$ 554.0823; found 554.0865.

Azetidine chiral SFC analysis [Column: Chiralpak IC 4.6 x 100 mm 5 µm; Mobile Phase: 5-55% MeOH w/ 10 mM NH_4OH/CO_2 5 mL/min 120 bar]:

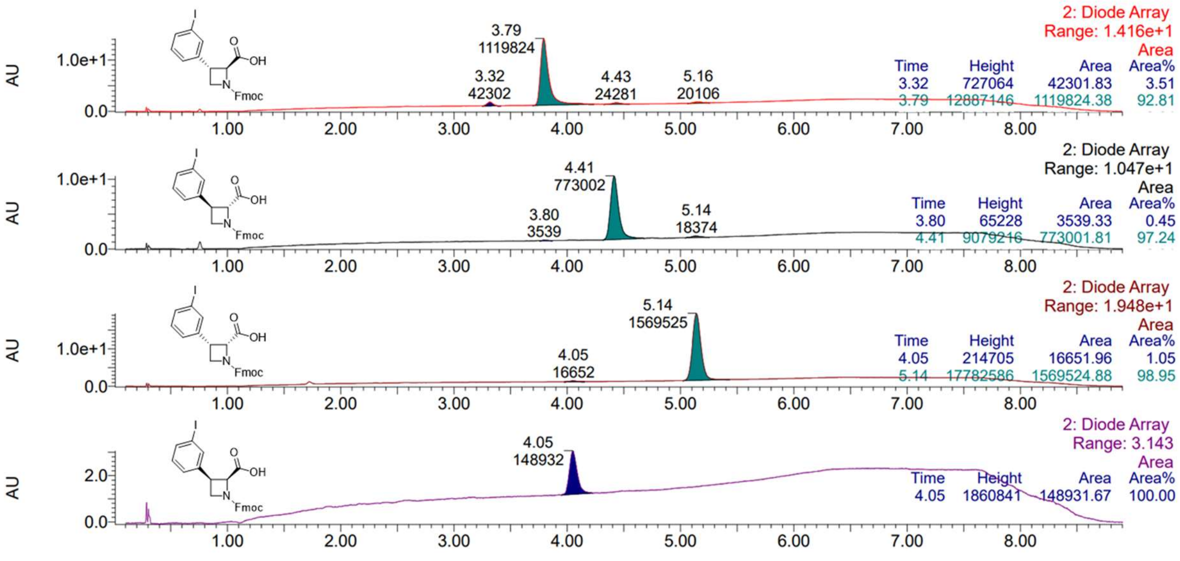

Pyrrolidine chiral SFC analysis [Column: Chiralcel OJ-H 4.6 x 100 mm 5 µm; Mobile Phase: 10% MeOH w/ 10 mM NH_4OH/CO_2 5 mL/min 120 bar]:

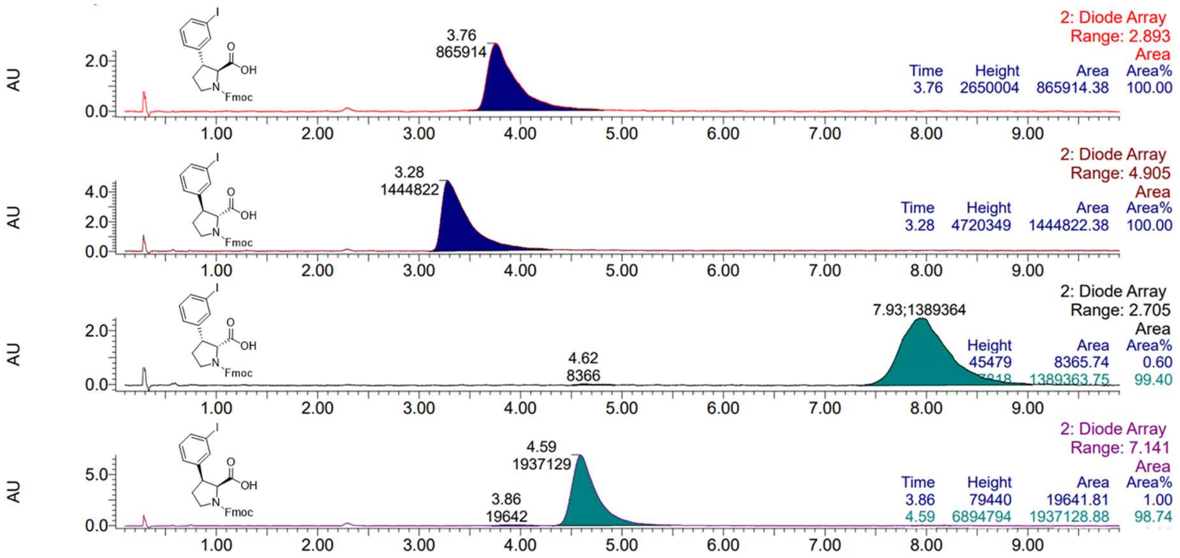

Piperidine chiral SFC analysis [Column: Chiralpak AD-3 3 x 100 mm; Mobile Phase: 5-55% 1:1 MeOH:IPA w/ 10 mM NH_4OH/CO_2 2.5 mL/min 120 bar]:

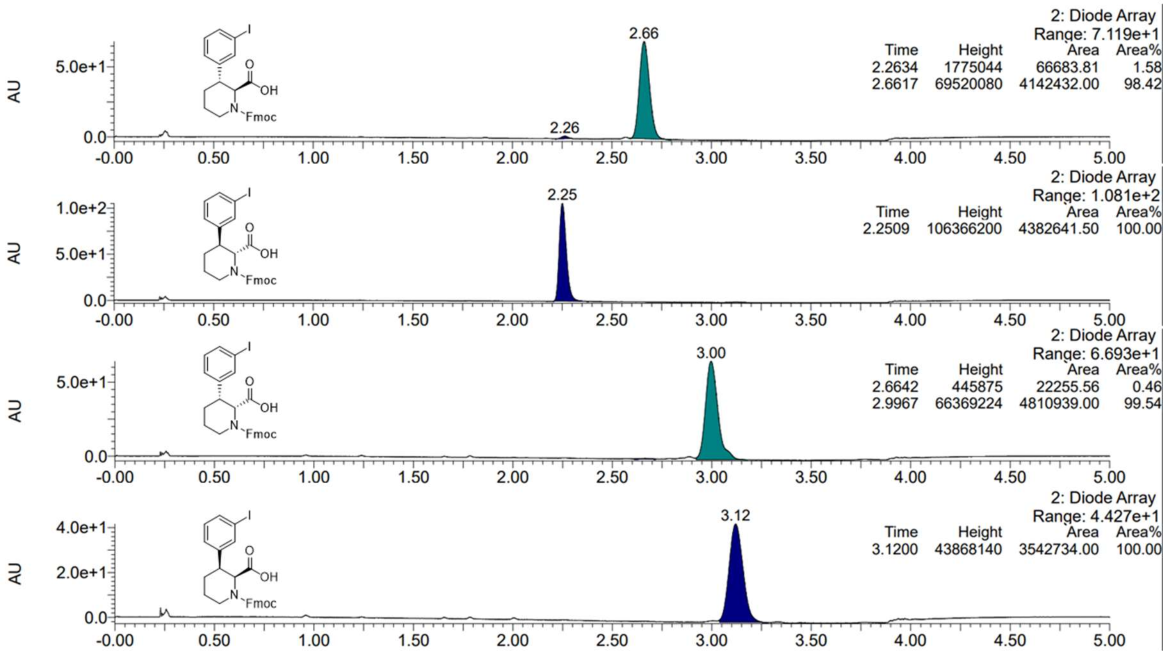

1.2. Arylated and benzylated hydroxyproline stereoisomers

General preparation (A) of O-aryl hydroxyproline derivatives were performed as described in Supplementary Fig. 2.

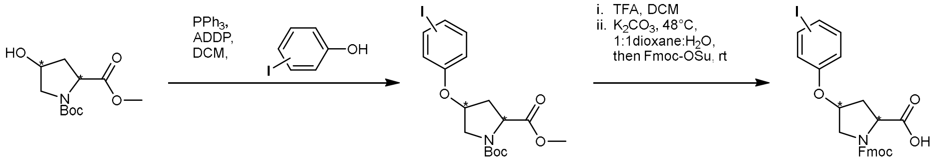

8a–d 9a–di–ii 10a–di–ii

Supplementary Fig. 2 | General preparation of O-aryl hydroxyproline derivatives.

Part 1: A commercially sourced chirally pure isomer of N-Boc hydroxyproline methyl ester (**8a–d**, 1 eq) was added to a reaction vessel, followed by PPh_3 (1.4 eq), 3- or 4-iodophenol (1.2 eq) and CH_2Cl_2 (0.1 M). The solution was cooled to 0 °C before the addition of ADDP (1.2 eq), and the reaction was stirred overnight while allowing to warm to rt. The reaction mixture was diluted with H_2O and extracted three times with CH_2Cl_2. Combined organic portions were evaporated onto Isolute HM-N and purified by flash chromatography to afford **9a–di–ii** as white foaming solids.

Part 2: A purified sample of **9a–di–ii** was dissolved in CH_2Cl_2 (0.16 M), then added TFA (40 eq) while stirring at rt. After complete Boc-deprotection could be observed by LCMS analysis the mixture was diluted with CH_2Cl_2 and volatile components were removed under reduced pressure. To the residue was added 1:1 1,4-dioxane:water (0.13 M) followed by K_2CO_3 (3–6 eq), and the resulting mixture was heated with stirring to 48 °C until complete ester hydrolysis could be observed by LCMS analysis. The mixture was cooled to rt before Fmoc-OSu (0.9 eq) was added and the mixture was stirred until complete Fmoc protection could be observed by LCMS. The crude reaction mixture was separated between ice-water and EtOAc, then pH was adjusted to approximately 2 with 1 M HCl. The aqueous layer was extracted three times with EtOAc, and pooled organic portions were dried over $MgSO_4$, filtered and solvent removed under reduced pressure. **10a–di–ii** was isolated by flash chromatography with a gradient of EtOAc+0.1% AcOH in heptane.

General preparation (B) of O-benzyl hydroxyproline derivatives were performed as described in Supplementary Fig. 3.

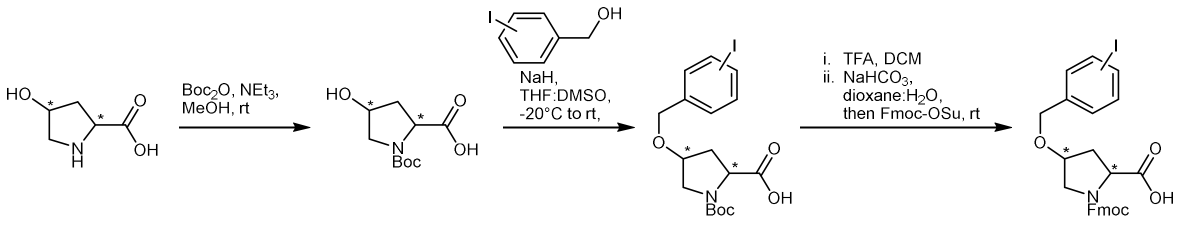

Supplementary Fig. 3 | General preparation of O-benzyl hydroxyproline derivatives.

Part 1: A commercially sourced chirally pure isomer of hydroxyproline (**11a–d**, 1 eq) was dissolved in MeOH (0.19 M) before adding NEt_3 (1.9 eq) and Boc_2O (2 eq). The resulting mixture was heated at reflux for 2 h, then cooled to rt and stirred overnight. Volatiles were removed under reduced pressure before adding water (25 rel. vol.) and EtOAc (25 rel. vol.). The mixture was cooled to approximately 0 °C before addition of NaH_2PO_4 (approximately 10% wt/wt), adjusting pH to approximately 2 with 0.2 M HCl (aq) and collecting the organic portion. The aqueous portion was extracted twice with EtOAc, and pooled organic portions were dried over $MgSO_4$, filtered and solvent removed under reduced pressure to afford **12a–d** as white foaming solids.

Part 2: NaH [60% in mineral oil] (2.5 eq) was added to a sample with septum before removing the mineral oil by rinsing with heptanes and drying under a stream of N_2. The resulting white solid was cooled to -20 °C with a bath of 7:3 H_2O:MeOH and dry ice, then a solution of **12a–d** (1 eq, 0.74 M) in a 10:1 mixture of THF:DMSO was added. After 20 min the reaction was warmed to 0 °C for 10 min, then cooled again to -20 °C before adding a solution of 3- or 4-iodobenzylbromide (1.3 eq, 0.84 M, THF). After approximately 20 min the reaction mixture was warmed to rt and stirred until LCMS indicated complete consumption of starting material. The crude reaction mixture was diluted with water and EtOAc, then pH was adjusted to approximately 2 with 1 M HCl. The organic phase was isolated, and the aqueous portion was extracted twice with EtOAc. Combined organic portions were dried over $MgSO_4$, filtered and solvent was removed under reduced pressure. **13a–di–ii** were purified by flash chromatography (EtOAc/heptane) to afford a white foaming solid.

Part 3: A purified sample of **13a–di–ii** was dissolved in CH_2Cl_2 (0.16 M), then added TFA (20–40 eq) while stirring at rt. After complete Boc-deprotection could be observed by LCMS analysis the mixture was diluted with CH_2Cl_2 and volatiles were removed under reduced pressure. To the residue was added 5:1 1,4-dioxane:water (0.10 M) followed by $NaHCO_3$ (3–6 eq) and Fmoc-OSu (1.2 eq), and the mixture stirred until complete Fmoc protection could be observed by LCMS. The crude reaction mixture was diluted with 0.1 M HCl and extracted three times with EtOAc. Pooled organic portions were dried over $MgSO_4$, filtered

and solvent removed under reduced pressure. **14a–di–ii** were isolated by preparative HPLC as a white solid.

(2*S*,4*S*)-1-*tert*-butyl 2-methyl 4-(4-iodophenoxy)pyrrolidine-1,2-dicarboxylate (**9ai**): Synthesized following general procedure A, Part 1. Yield: 81%.

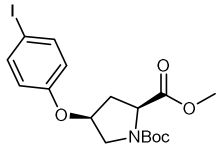

^{1}H NMR (400 MHz, $CDCl_3$) (57:43 mixture of rotamers) δ 7.58 – 7.51 (m, 2H), 6.58 (app. dd, *J* = 8.8, 3.4 Hz, 2H), 4.89 – 4.82 (m, 1H), 4.54 (dd, *J* = 8.3, 3.2 Hz, 0.43H), 4.42 (dd, *J* = 8.3, 3.5 Hz, 0.57H), 3.82 – 3.74 (m, 1H), 3.72 (app. d, *J* = 6.5 Hz, 3H), 3.71 – 3.60 (m, 1H), 2.50 – 2.40 (m, 2H), 1.46 (app. d, *J* = 18.1 Hz, 9H).

(2*S*,4*R*)-1-*tert*-butyl 2-methyl 4-(4-iodophenoxy)pyrrolidine-1,2-dicarboxylate (**9bi**): Synthesized following general procedure A, Part 1. Yield: 72%.

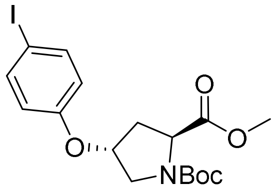

^{1}H NMR (400 MHz, $CDCl_3$) (3:2 mixture of rotamers) δ 7.55 – 7.49 (m, 2H), 6.64 – 6.58 (m, 2H), 4.88 – 4.80 (m, 1H), 4.45 (t, *J* = 7.7 Hz, 0.4H), 4.38 (t, *J* = 8.0 Hz, 0.6H), 3.77 – 3.73 (m, 1.6H), 3.72 (s, 3H), 3.62 (d, *J* = 11.9 Hz, 0.4H), 2.54 – 2.42 (m, 1H), 2.23 – 2.14 (m, 1H), 1.41 (app. d, *J* = 11.8 Hz, 9H). ^{13}C NMR (101 MHz, $CDCl_3$) δ 173.24, 173.03, 156.74, 154.25, 153.57, 138.42, 117.90, 83.70, 80.45, 75.52, 74.80, 57.91, 57.54, 52.34, 52.15, 52.00, 51.76, 36.43, 35.48, 28.36, 28.24. HRMS (ESI$^+$) m/z calculated for $C_{17}H_{22}INO_5$ $[M\text{-}Boc+H]^+$ 348.0097; found 348.0141.

(2*R*,4*R*)-1-*tert*-butyl 2-methyl 4-(4-iodophenoxy)pyrrolidine-1,2-dicarboxylate (**9ci**): Synthesized following general procedure A, Part 1. Yield: 60%.

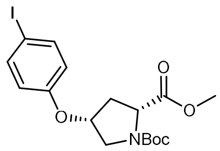

^{1}H NMR (400 MHz, $CDCl_3$) δ 7.53 – 7.47 (m, 2H), 6.55 (dd, *J* = 8.9, 3.4 Hz, 2H), 4.82 (dtd, *J* = 9.4, 4.7, 2.3 Hz, 1H), 4.50 (dd, *J* = 7.9, 3.4 Hz, 0.43H), 4.39 (dd, *J* = 8.5, 3.2 Hz, 0.57H), 3.74 (app. dd, *J* = 12.2, 5.0 Hz, 1H), 3.69 (s, 3H), 3.66 – 3.58 (m, 1H), 2.49 – 2.33 (m, 2H), 1.42 (app. d, *J* = 17.8 Hz, 9H). ^{13}C

NMR (101 MHz, $CDCl_3$) δ 172.35, 171.97, 156.47, 154.10, 153.71, 138.38, 117.92, 83.62, 80.29, 80.21, 75.63, 74.59, 57.77, 57.42, 52.23, 52.10, 51.87, 51.48, 36.14, 35.26, 28.39, 28.28. HRMS (ESI$^+$) m/z calculated for $C_{17}H_{22}INO_5$ [M-Boc+H]$^+$ 348.0097; found 348.0125.

(2*R*,4*S*)-1-*tert*-butyl 2-methyl 4-(4-iodophenoxy)pyrrolidine-1,2-dicarboxylate (**9di**): Synthesized following general procedure A, Part 1. Yield: 80%.

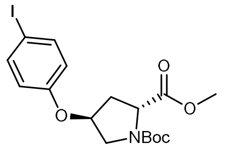

^{1}H NMR (400 MHz, $CDCl_3$) (3:2 mixture of rotamers) δ 7.51 (dd, *J* = 8.6, 6.0 Hz, 2H), 6.63 – 6.55 (m, 2H), 4.87 – 4.79 (m, 1H), 4.44 (t, *J* = 7.8 Hz, 0.4H), 4.37 (t, *J* = 8.0 Hz, 0.6H), 3.76 – 3.72 (m, 1.6H), 3.71 (s, 3H), 3.61 (d, *J* = 11.9 Hz, 0.4H), 2.54 – 2.41 (m, 1H), 2.23 – 2.13 (m, 1H), 1.40 (app. d, *J* = 11.9 Hz, 9H). ^{13}C NMR (101 MHz, $CDCl_3$) δ 173.22, 173.01, 156.73, 154.24, 153.57, 138.41, 117.89, 83.68, 80.44, 75.51, 74.79, 57.91, 57.54, 52.32, 52.14, 51.99, 51.75, 36.41, 35.47, 28.41, 28.22. HRMS (ESI$^+$) m/z calculated for $C_{17}H_{22}INO_5$ [M-Boc+H]$^+$ 348.0097; found 348.0134.

(2*S*,4*S*)-1-*tert*-butyl 2-methyl 4-(3-iodophenoxy)pyrrolidine-1,2-dicarboxylate (**9aii**): Synthesized following general procedure A, Part 1. Yield: 76%.

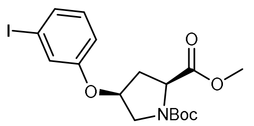

^{1}H NMR (400 MHz, $CDCl_3$) (3:2 mixture of rotamers) δ 7.31 – 7.24 (m, 1H), 7.16 – 7.11 (m, 1H), 7.00 – 6.93 (m, 1H), 6.78 – 6.72 (m, 1H), 4.89 – 4.81 (m, 1H), 4.53 (dd, *J* = 8.0, 3.3 Hz, 0.4H), 4.41 (dd, *J* = 8.6, 3.2 Hz, 0.6H), 3.80 – 3.73 (m, 1H), 3.71 (d, *J* = 8.6 Hz, 3H), 3.69 – 3.61 (m, 1H), 2.53 – 2.38 (m, 2H), 1.45 (app. d, *J* = 19.4 Hz, 9H). ^{13}C NMR (101 MHz, $CDCl_3$) δ 172.35, 171.96, 157.18, 154.14, 153.74, 130.96, 130.56, 124.78, 124.71, 115.11, 94.44, 94.41, 80.37, 80.28, 75.78, 74.69, 57.79, 57.43, 52.28, 52.14, 51.95, 51.53, 36.21, 35.29, 28.42, 28.29. HRMS (ESI$^+$) m/z calculated for $C_{17}H_{22}INO_5$ [M-Boc+H]$^+$ 348.0097; found 348.0111.

(2*S*,4*R*)-1-*tert*-butyl 2-methyl 4-(3-iodophenoxy)pyrrolidine-1,2-dicarboxylate (**9bii**): Synthesized following general procedure A, Part 1. Yield: 52%.

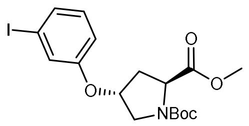

^{1}H NMR (400 MHz, $CDCl_3$) (3:2 mixture of rotamers) δ 7.34 – 7.27 (m, 1H), 7.23 – 7.19 (m, 1H), 7.03 – 6.96 (m, 1H), 6.85 – 6.79 (m, 1H), 4.91 – 4.84 (m, 1H), 4.48 (t, *J* = 7.8 Hz, 0.4H), 4.41 (t, *J* = 8.0 Hz, 0.6H), 3.80 – 3.73 (m, 4.6H), 3.66 (d, *J* = 11.9 Hz, 0.4H), 2.58 – 2.45 (m, 1H), 2.26 – 2.17 (m, 1H), 1.44 (app. d, *J* = 13.5 Hz, 9H). ^{13}C NMR (101 MHz, $CDCl_3$) δ 173.28, 173.10, 157.42, 154.30, 153.62, 130.97, 130.70, 130.63, 124.84, 124.76, 115.11, 114.96, 94.46, 80.55, 75.70, 74.87, 57.93, 57.55, 52.37, 52.17, 52.05, 51.81, 36.49, 35.54, 28.37, 28.24. HRMS (ESI$^+$) m/z calculated for $C_{17}H_{22}INO_5$ [M-Boc+H]$^+$ 348.0097; found 348.0135.

(2*R*,4*R*)-1-*tert*-butyl 2-methyl 4-(3-iodophenoxy)pyrrolidine-1,2-dicarboxylate (**9cii**): Synthesized following general procedure A, Part 1. Yield: 86%.

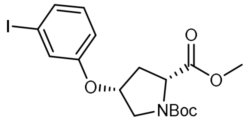

^{1}H NMR (400 MHz, $CDCl_3$) (3:2 mixture of rotamers) δ 7.27 (dd, *J* = 8.1, 4.9 Hz, 1H), 7.15 – 7.10 (m, 1H), 7.00 – 6.92 (m, 1H), 6.77 – 6.71 (m, 1H), 4.88 – 4.81 (m, 1H), 4.52 (dd, *J* = 8.0, 3.5 Hz, 0.4H), 4.40 (dd, *J* = 8.6, 3.1 Hz, 0.6H), 3.79 – 3.72 (m, 1H), 3.70 (app. d, *J* = 8.2 Hz, 3H), 3.64 (t, *J* = 13.6 Hz, 1H), 2.51 – 2.37 (m, 2H), 1.44 (app. d, *J* = 19.4 Hz, 9H). ^{13}C NMR (101 MHz, $CDCl_3$) δ 172.33, 171.95, 157.18, 154.10, 153.71, 130.96, 130.54, 124.78, 124.70, 115.11, 94.41, 80.31, 80.23, 75.78, 74.68, 57.78, 57.42, 52.26, 52.13, 51.94, 51.52, 36.20, 35.28, 28.41, 28.29. HRMS (ESI$^+$) m/z calculated for $C_{17}H_{22}INO_5$ [M-Boc+H]$^+$ 348.0097; found 348.0124.

(2*R*,4*S*)-1-*tert*-butyl 2-methyl 4-(3-iodophenoxy)pyrrolidine-1,2-dicarboxylate (**9dii**): Synthesized following general procedure A, Part 1. Yield: 71%.

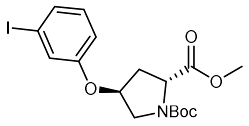

^{1}H NMR (400 MHz, $CDCl_3$) (3:2 mixture of rotamers) δ 7.28 (dd, *J* = 8.2, 6.3 Hz, 1H), 7.18 (dd, *J* = 2.4, 2.0 Hz, 1H), 6.97 (q, *J* = 7.5 Hz, 1H), 6.82 – 6.76 (m, 1H), 4.88 – 4.81 (m, 1H), 4.46 (t, *J* = 7.8 Hz, 0.4H), 4.38 (t, *J* = 8.0 Hz, 0.6H), 3.77 – 3.71 (m, 4.6H), 3.64 (d, *J* = 11.9 Hz, 0.4H), 2.56 – 2.42 (m, 1H), 2.24 –

2.14 (m, 1H), 1.42 (app. d, J = 13.1 Hz, 9H). ^{13}C NMR (101 MHz, $CDCl_3$) δ 173.23, 173.04, 157.44, 157.39, 154.27, 153.60, 130.97, 130.66, 130.58, 124.83, 124.74, 115.08, 114.94, 94.46, 80.51, 75.69, 74.85, 57.91, 57.54, 52.34, 52.16, 52.05, 51.80, 36.46, 35.51, 28.35, 28.23. HRMS (ESI$^+$) m/z calculated for $C_{17}H_{22}INO_5$ [M-Boc+H]$^+$ 348.0097; found 348.0121.

(2*S*,4*S*)-1-(*tert*-butoxycarbonyl)-4-((4-iodobenzyl)oxy)pyrrolidine-2-carboxylic acid (**13ai**): Synthesized following general procedure B, Parts 1 and 2. Yield: 37% (2 steps).

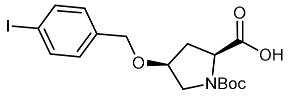

^{1}H NMR (400 MHz, $CDCl_3$) (1:1 mixture of rotamers) δ 10.97 (s, 1H), 7.62 (app. t, J = 6.7 Hz, 2H), 7.08 – 6.92 (m, 2H), 4.53 – 4.29 (m, 3H), 4.13 – 4.09 (m, 1H), 3.69 – 3.50 (m, 2H), 2.65 (d, J = 13.9 Hz, 0.5H), 2.46 – 2.31 (m, 1H), 2.24 – 2.12 (m, 0.5H), 1.46 (app. d, J = 18.7 Hz, 9H). ^{13}C NMR (101 MHz, $CDCl_3$) δ 177.06, 177.04, 177.02, 174.94, 174.89, 174.85, 156.02, 154.06, 137.43, 137.31, 129.62, 129.39, 93.14, 81.48, 80.72, 76.71, 76.04, 70.06, 69.89, 57.84, 57.73, 52.87, 51.39, 35.96, 33.32, 28.38, 28.28. HRMS (ESI$^+$) m/z calculated for $C_{17}H_{22}INO_5$ [M-Boc+H]$^+$ 348.0097; found 348.0104.

(2*S*,4*R*)-1-(*tert*-butoxycarbonyl)-4-((4-iodobenzyl)oxy)pyrrolidine-2-carboxylic acid (**13bi**): Synthesized following general procedure B, Parts 1 and 2. Yield: 54% (2 steps).

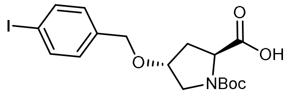

^{1}H NMR (400 MHz, $CDCl_3$) (55:45 mixture of rotamers) δ 9.62 (s, 1H), 7.72 – 7.61 (m, 2H), 7.11 – 7.00 (m, 2H), 4.51 – 4.34 (m, 3H), 4.21 – 4.14 (m, 1H), 3.73 (d, J = 11.8 Hz, 0.55H), 3.63 – 3.51 (m, 1.45H), 2.50 – 2.31 (m, 1H), 2.31 – 2.21 (m, 0.45H), 2.18 – 2.08 (m, 0.55H), 1.44 (app. d, J = 18.8 Hz, 9H). ^{13}C NMR (101 MHz, $CDCl_3$) δ 178.11, 176.03, 155.54, 153.99, 137.56, 137.41, 137.37, 129.51, 129.43, 93.35, 81.29, 80.90, 76.68, 76.21, 70.56, 70.40, 57.91, 57.76, 51.93, 51.31, 36.63, 34.96, 28.39, 28.24.

(2*R*,4*R*)-1-(*tert*-butoxycarbonyl)-4-((4-iodobenzyl)oxy)pyrrolidine-2-carboxylic acid (**13ci**): Synthesized following general procedure B, Parts 1 and 2. Yield: 47% (2 steps).

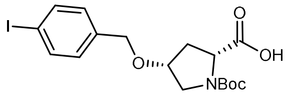

^{1}H NMR (400 MHz, $CDCl_3$) (1:1 mixture of rotamers) δ 11.41 (s, 1H), 7.38 (t, J = 7.2 Hz, 2H), 6.77 (t, J = 7.9 Hz, 2H), 4.30 – 4.08 (m, 3H), 3.90 – 3.84 (m, 1H), 3.45 – 3.31 (m, 2H), 2.31 (d, J = 13.7 Hz, 0.5H), 2.15 (t, J = 5.2 Hz, 1H), 2.06 – 1.93 (m, 0.5H), 1.24 (app. d, J = 18.9 Hz, 9H). ^{13}C NMR (101 MHz, $CDCl_3$) δ 176.89, 175.59, 155.42, 154.14, 137.44, 137.33, 129.49, 129.37, 93.04, 81.01, 80.64, 76.85, 76.03,

69.98, 69.89, 57.63, 52.58, 51.38, 35.95, 33.97, 28.42, 28.28. HRMS (ESI$^+$) m/z calculated for $C_{17}H_{22}INO_5$ [M-Boc+H]$^+$ 348.0097; found 348.0102.

(2*R*,4*S*)-1-(*tert*-butoxycarbonyl)-4-((4-iodobenzyl)oxy)pyrrolidine-2-carboxylic acid (**13di**): Synthesized following general procedure B, Parts 1 and 2. Yield: 42% (2 steps).

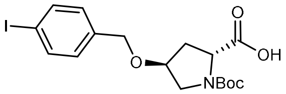

^{1}H NMR (400 MHz, $CDCl_3$) (3:2 mixture of rotamers) δ 10.67 (s, 1H), 7.66 (app. dd, *J* = 8.2, 3.0 Hz, 2H), 7.05 (d, *J* = 8.1 Hz, 2H), 4.50 – 4.34 (m, 3H), 4.21 – 4.13 (m, 1H), 3.73 (d, *J* = 11.7 Hz, 0.6H), 3.63 – 3.52 (m, 1.4H), 2.48 – 2.32 (m, 1H), 2.28 – 2.20 (m, 0.4H), 2.17 – 2.07 (m, 0.6H), 1.44 (app. d, *J* = 17.6 Hz, 9H). ^{13}C NMR (101 MHz, $CDCl_3$) δ 178.04, 176.23, 155.42, 154.01, 137.43, 129.52, 129.43, 93.35, 81.19, 80.91, 76.71, 76.21, 70.54, 70.38, 57.92, 57.73, 51.93, 51.32, 36.63, 35.03, 28.40, 28.25. HRMS (ESI$^+$) m/z calculated for $C_{17}H_{22}INO_5$ [M-Boc+H]$^+$ 348.0097; found 348.0103.

(2*S*,4*S*)-1-(*tert*-butoxycarbonyl)-4-((3-iodobenzyl)oxy)pyrrolidine-2-carboxylic acid (**14aii**): Synthesized following general procedure B, Parts 1 and 2. Yield: 49% (2 steps).

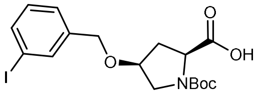

^{1}H NMR (400 MHz, $CDCl_3$) (1:1 mixture of rotamers) δ 9.94 (s, 1H), 7.68 – 7.55 (m, 2H), 7.31 – 7.22 (m, 1H), 7.06 (t, *J* = 7.8 Hz, 1H), 4.55 – 4.30 (m, 3H), 4.12 – 4.09 (m, 1H), 3.60 (d, *J* = 30.9 Hz, 2H), 2.66 (d, *J* = 13.8 Hz, 0.5H), 2.36 (s, 1H), 2.23 – 2.12 (m, 0.5H), 1.46 (app. d, *J* = 21.4 Hz, 9H). ^{13}C NMR (101 MHz, $CDCl_3$) δ 176.75, 174.50, 156.18, 154.04, 140.07, 136.75, 136.53, 136.33, 130.26, 126.98, 126.72, 94.31, 81.61, 80.76, 76.62, 76.00, 69.86, 69.62, 57.87, 57.71, 52.93, 51.41, 35.79, 33.07, 28.37, 28.29. HRMS (ESI$^+$) m/z calculated for $C_{17}H_{22}INO_5$ [M-Boc+H]$^+$ 348.0097; found 348.0125.

(2*S*,4*R*)-1-(*tert*-butoxycarbonyl)-4-((3-iodobenzyl)oxy)pyrrolidine-2-carboxylic acid (**14bii**): Synthesized following general procedure B, Parts 1 and 2. Yield: 56% (2 steps).

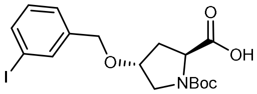

^{1}H NMR (400 MHz, $CDCl_3$) δ 10.12 (s, 1H), 7.66 (s, 1H), 7.63 – 7.57 (m, 1H), 7.26 (d, *J* = 7.7 Hz, 1H), 7.06 (td, *J* = 7.8, 3.3 Hz, 1H), 4.50 – 4.34 (m, 3H), 4.21 – 4.14 (m, 1H), 3.73 (d, *J* = 11.8 Hz, 1H), 3.63 – 3.53 (m, 1H), 2.50 – 2.32 (m, 1H), 2.29 – 2.20 (m, 0H), 2.18 – 2.09 (m, 1H), 1.44 (d, *J* = 17.3 Hz, 9H). ^{13}C NMR (101 MHz, $CDCl_3$) δ 177.89, 176.16, 155.39, 154.05, 140.11, 136.86, 136.82, 136.40, 136.37,

130.25, 126.73, 126.68, 94.46, 81.19, 80.94, 76.79, 76.34, 70.25, 70.07, 57.93, 57.72, 51.92, 51.31, 36.61, 35.02, 28.42, 28.26.

(2*R*,4*R*)-1-(*tert*-butoxycarbonyl)-4-((3-iodobenzyl)oxy)pyrrolidine-2-carboxylic acid (**14cii**): Synthesized following general procedure B, Parts 1 and 2. Yield: 59% (2 steps).

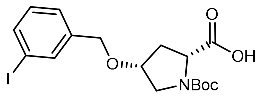

^{1}H NMR (400 MHz, $CDCl_3$) (1:1 mixture of rotamers) δ 11.65 (s, 1H), 7.55 (s, 1H), 7.51 (d, *J* = 8.1 Hz, 1H), 7.22 – 7.16 (m, 1H), 7.02 – 6.96 (m, 1H), 4.44 – 4.24 (m, 3H), 4.07 – 3.99 (m, 1H), 3.61 – 3.45 (m, 2H), 2.44 (d, *J* = 13.7 Hz, 0.5H), 2.32 – 2.27 (m, 1H), 2.23 – 2.08 (m, 0.5H), 1.40 (app. d, *J* = 20.6 Hz, 9H). ^{13}C NMR (101 MHz, $CDCl_3$) δ 176.62, 175.46, 155.36, 154.13, 140.19, 136.58, 136.28, 136.16, 130.25, 126.81, 126.67, 94.30, 80.96, 80.66, 76.81, 76.01, 69.76, 69.62, 57.60, 57.54, 52.57, 51.38, 35.78, 33.89, 28.42, 28.27. HRMS (ESI^{+}) m/z calculated for $C_{17}H_{22}INO_5$ [M-Boc+H]$^{+}$ 348.0097; found 348.0121.

(2*R*,4*S*)-1-(*tert*-butoxycarbonyl)-4-((3-iodobenzyl)oxy)pyrrolidine-2-carboxylic acid (**14dii**): Synthesized following general procedure B, Parts 1 and 2. Yield: 35% (2 steps).

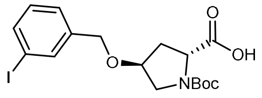

^{1}H NMR (400 MHz, $CDCl_3$) (3:2 mixture of rotamers) δ 10.80 (s, 1H), 7.65 (app. t, *J* = 1.7 Hz, 1H), 7.60 (app. dd, *J* = 7.9, 3.4 Hz, 1H), 7.26 (d, *J* = 7.6 Hz, 1H), 7.06 (app. td, *J* = 7.6, 3.7 Hz, 1H), 4.49 – 4.34 (m, 3H), 4.21 – 4.13 (m, 1H), 3.73 (d, *J* = 11.8 Hz, 0.6H), 3.63 – 3.54 (m, 1.4H), 2.49 – 2.32 (m, 1H), 2.28 – 2.20 (m, 0.4H), 2.13 (ddd, *J* = 13.0, 7.8, 5.0 Hz, 0.6H), 1.44 (app. d, *J* = 17.1 Hz, 9H). ^{13}C NMR (101 MHz, $CDCl_3$) δ 177.87, 176.22, 155.35, 154.04, 140.12, 136.84, 136.81, 136.39, 136.36, 130.24, 126.73, 126.68, 94.45, 81.15, 80.93, 76.79, 76.33, 70.24, 70.06, 57.92, 57.71, 51.91, 51.31, 36.61, 35.03, 28.41, 28.25. HRMS (ESI^{+}) m/z calculated for $C_{17}H_{22}INO_5$ [M-Boc+H]$^{+}$ 348.0097; found 348.0097.

(2*S*,4*S*)-1-(((9H-fluoren-9-yl)methoxy)carbonyl)-4-(4-iodophenoxy)pyrrolidine-2-carboxylic acid (**10ai**): Synthesized following general procedure A, Part 2. Yield: 52%.

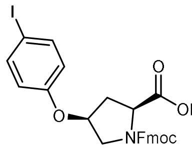

^{1}H NMR (400 MHz, Methanol-d_4) (1:1 mixture of rotamers) δ 7.80 – 7.74 (m, 2H), 7.70 – 7.59 (m, 2H), 7.59 – 7.52 (m, 2H), 7.40 – 7.34 (m, 2H), 7.34 – 7.24 (m, 2H), 6.76 – 6.68 (m, 2H), 4.93 (ddp, *J* = 12.8,

5.0, 2.3 Hz, 1H), 4.49 – 4.18 (m, 4H), 3.87 (dd, *J* = 11.9, 5.5 Hz, 0.5H), 3.76 (dd, *J* = 11.9, 5.3 Hz, 0.5H), 3.71 – 3.60 (m, 1H), 2.59 (ddd, *J* = 14.3, 9.3, 5.2 Hz, 0.5H), 2.49 (ddd, *J* = 14.4, 9.5, 5.2 Hz, 0.5H), 2.45 – 2.32 (m, 1H). ^{13}C NMR (101 MHz, CD_3OD) δ 176.23, 175.85, 157.12, 157.02, 155.44, 155.26, 144.23, 144.15, 143.80, 143.75, 141.28, 141.15, 141.04, 138.10, 138.08, 127.38, 126.85, 126.77, 125.14, 125.04, 124.84, 124.80, 119.51, 119.46, 119.44, 118.08, 118.00, 82.57, 82.48, 75.59, 74.80, 67.74, 67.26, 59.27, 59.18, 52.06, 51.60, 47.03, 36.11, 35.13. HRMS (ESI$^+$) m/z calculated for $C_{26}H_{22}INO_5$ [M+H]$^+$ 556.0621; found 556.0627.

(2*S*,4*R*)-1-(((9H-fluoren-9-yl)methoxy)carbonyl)-4-(4-iodophenoxy)pyrrolidine-2-carboxylic acid (**10bi**): Synthesized following general procedure A, Part 2. Yield: 64%.

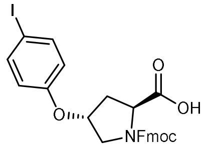

^{1}H NMR (400 MHz, $CDCl_3$) (3:2 mixture of rotamers) δ 11.37 (s, 1H), 7.75 (app. dd, *J* = 7.6, 5.5 Hz, 2H), 7.65 – 7.48 (m, 4H), 7.45 – 7.37 (m, 2H), 7.35 – 7.21 (m, 2H), 6.69 – 6.59 (m, 2H), 4.93 – 4.86 (m, 0.6H), 4.86 – 4.80 (m, 0.4H), 4.64 (t, *J* = 8.0 Hz, 0.6H), 4.57 – 4.38 (m, 2.4H), 4.27 (t, *J* = 7.0 Hz, 0.6H), 4.22 – 4.18 (m, 0.4H), 3.95 – 3.75 (m, 2H), 2.68 – 2.55 (m, 1H), 2.40 – 2.27 (m, 1H). ^{13}C NMR (101 MHz, $CDCl_3$) δ 177.63, 177.48, 156.62, 156.53, 155.41, 154.69, 143.92, 143.68, 143.63, 143.61, 141.32, 141.28, 138.63, 138.54, 127.84, 127.78, 127.15, 125.02, 124.96, 120.06, 117.91, 84.08, 84.01, 75.27, 74.61, 68.04, 58.04, 57.49, 52.49, 51.88, 47.17, 47.07, 36.60, 35.30. HRMS (ESI$^+$) m/z calculated for $C_{26}H_{22}INO_5$ [M+H]$^+$ 556.0621; found 556.0630.

(2*R*,4*R*)-1-(((9H-fluoren-9-yl)methoxy)carbonyl)-4-(4-iodophenoxy)pyrrolidine-2-carboxylic acid (**10ci**): Synthesized following general procedure A, Part 2. Yield: 72%.

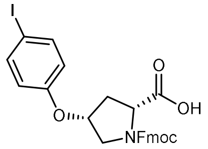

^{1}H NMR (400 MHz, $CDCl_3$) (55:45 mixture of rotamers) δ 11.57 (s, 1H), 7.75 (app. dd, *J* = 7.5, 4.7 Hz, 2H), 7.60 – 7.47 (m, 4H), 7.44 – 7.34 (m, 2H), 7.34 – 7.24 (m, 2H), 6.58 (app. dd, *J* = 22.0, 8.7 Hz, 2H), 4.94 – 4.87 (m, 0.55H), 4.87 – 4.81 (m, 0.45H), 4.69 – 4.32 (m, 3H), 4.28 (t, *J* = 6.9 Hz, 0.55H), 4.21 (t, *J* = 6.5 Hz, 0.45H), 3.82 – 3.69 (m, 2H), 2.64 – 2.51 (m, 1H), 2.45 (app. ddt, *J* = 13.9, 9.2, 4.3 Hz, 1H). ^{13}C NMR (101 MHz, $CDCl_3$) δ 176.90, 176.32, 156.20, 156.17, 155.11, 154.64, 143.91, 143.71, 143.61, 141.36, 141.27, 138.52, 138.46, 127.79, 127.75, 127.14, 127.11, 125.15, 125.01, 124.91, 120.01, 118.19, 118.04, 84.16, 84.06, 75.55, 74.58, 67.85, 67.78, 57.88, 57.48, 52.10, 51.74, 47.16, 36.36, 35.15. HRMS (ESI$^+$) m/z calculated for $C_{26}H_{22}INO_5$ [M+H]$^+$ 556.0621; found 556.0648.

(2*R*,4*S*)-1-(((9H-fluoren-9-yl)methoxy)carbonyl)-4-(4-iodophenoxy)pyrrolidine-2-carboxylic acid (**10di**): Synthesized following general procedure A, Part 2. Yield: 36%.

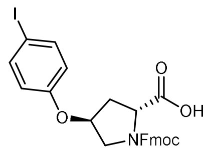

^{1}H NMR (400 MHz, DMSO) δ 7.90 – 7.83 (m, 2H), 7.78 (dd, *J* = 20.8, 7.5 Hz, 1H), 7.65 – 7.53 (m, 3H), 7.44 – 7.36 (m, 2H), 7.36 – 7.29 (m, 1H), 7.23 (dt, *J* = 12.7, 7.5 Hz, 1H), 6.82 – 6.76 (m, 2H), 4.99 – 4.93 (m, 1H), 4.28 – 4.09 (m, 3H), 4.06 – 3.97 (m, 1H), 3.73 (app. dt, *J* = 11.7, 5.0 Hz, 1H), 3.57 (app. ddd, *J* = 16.5, 11.7, 2.0 Hz, 1H), 2.33 (dd, *J* = 7.2, 4.6 Hz, 1H), 2.22 (dd, *J* = 7.4, 4.4 Hz, 1H). ^{13}C NMR (101 MHz, CD_3OD) δ 177.10, 176.99, 157.91, 157.83, 155.47, 155.31, 144.29, 144.17, 143.86, 143.76, 141.27, 141.16, 141.13, 141.03, 130.67, 130.65, 130.04, 129.98, 127.43, 127.40, 126.89, 126.87, 126.82, 126.79, 125.28, 125.12, 124.91, 124.88, 119.58, 119.50, 114.92, 114.84, 93.65, 75.63, 74.94, 67.76, 67.23, 59.80, 52.08, 51.62, 36.33, 35.30. HRMS (ESI$^+$) m/z calculated for $C_{26}H_{22}INO_5$ $[M+H]^+$ 556.0621; found 556.0637.

(2*S*,4*S*)-1-(((9H-fluoren-9-yl)methoxy)carbonyl)-4-(3-iodophenoxy)pyrrolidine-2-carboxylic acid (**10aii**): Synthesized following general procedure A, Part 2. Yield: 29%.

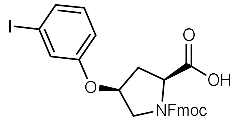

^{1}H NMR (400 MHz, Methanol-d_4) (1:1 mixture of rotamers) δ 7.68 – 7.60 (m, 2H), 7.60 – 7.46 (m, 2H), 7.28 – 7.12 (m, 6H), 6.94 – 6.84 (m, 1H), 6.79 (app. td, *J* = 9.0, 2.4 Hz, 1H), 4.84 – 4.70 (m, 2H), 4.36 (dd, *J* = 9.4, 3.0 Hz, 0.5H), 4.23 (d, *J* = 6.5 Hz, 2H), 4.17 (dd, *J* = 9.4, 3.3 Hz, 0.5H), 4.13 – 4.04 (m, 1H), 3.77 (dd, *J* = 11.9, 5.6 Hz, 0.5H), 3.64 (dd, *J* = 11.8, 5.4 Hz, 0.5H), 3.58 (dd, *J* = 11.9, 2.5 Hz, 0.5H), 3.51 (dd, *J* = 11.7, 2.5 Hz, 0.5H), 2.53 – 2.43 (m, 0.5H), 2.41 – 2.27 (m, 1H), 2.23 (dt, *J* = 13.8, 3.1 Hz, 0.5H). ^{13}C NMR (101 MHz, CD_3OD) δ 177.10, 176.99, 157.91, 157.83, 155.47, 155.31, 144.29, 144.17, 143.86, 143.76, 141.27, 141.16, 141.13, 141.03, 130.67, 130.65, 130.04, 129.98, 127.43, 127.40, 126.89, 126.87, 126.82, 126.79, 125.28, 125.12, 124.91, 124.88, 119.58, 119.50, 114.92, 114.84, 93.65, 75.63, 74.94, 67.76, 67.23, 59.80, 52.08, 51.62, 36.33, 35.30. HRMS (ESI$^+$) m/z calculated for $C_{26}H_{22}INO_5$ $[M+H]^+$ 556.0621; found 556.0634.

(2*S*,4*R*)-1-(((9H-fluoren-9-yl)methoxy)carbonyl)-4-(3-iodophenoxy)pyrrolidine-2-carboxylic acid (**10bii**): Synthesized following general procedure A, Part 2. Yield: 38%.

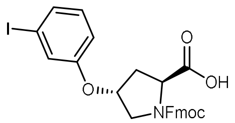

^{1}H NMR (400 MHz, Methanol-d_4) (43:57 mixture of rotamers) δ 7.67 – 7.53 (m, 2.86H), 7.42 (dd, *J* = 7.5, 1.0 Hz, 0.57H), 7.37 (dd, *J* = 7.5, 1.0 Hz, 0.57H), 7.29 – 7.13 (m, 4.86H), 7.11 (td, *J* = 7.5, 1.1 Hz, 0.57H), 7.05 (td, *J* = 7.5, 1.1 Hz, 0.57H), 6.97 – 6.89 (m, 1H), 6.85 – 6.80 (m, 0.43H), 6.78 (ddd, *J* = 8.4, 2.5, 0.9 Hz, 0.57H), 4.92 – 4.86 (m, 0.43H), 4.84 – 4.79 (m, 0.57H), 4.42 (t, *J* = 8.0 Hz, 0.43H), 4.34 (dd, *J* = 10.4, 6.6 Hz, 0.57H), 4.23 – 4.03 (m, 3H), 3.75 (dd, *J* = 12.0, 4.0 Hz, 0.43H), 3.70 (dt, *J* = 12.0, 1.9 Hz, 0.43H), 3.61 (dd, *J* = 12.0, 3.9 Hz, 0.57H), 3.52 (dt, *J* = 12.0, 1.6 Hz, 0.57H), 2.56 – 2.48 (m, 0.43H), 2.38 (ddt, *J* = 13.8, 7.8, 2.0 Hz, 0.57H), 2.22 (ddd, *J* = 13.3, 8.0, 4.8 Hz, 0.43H), 2.07 (ddd, *J* = 13.6, 8.8, 4.7 Hz, 0.57H). ^{13}C NMR (101 MHz, CD_3OD) δ 178.08, 178.07, 157.82, 157.73, 155.73, 155.26, 144.26, 144.01, 143.75, 143.69, 141.18, 141.17, 141.10, 141.00, 130.88, 130.80, 130.17, 130.15, 127.39, 127.36, 126.92, 126.89, 126.74, 126.69, 125.35, 125.17, 124.70, 124.67, 124.61, 124.50, 119.57, 119.50, 119.46, 114.76, 114.72, 93.87, 93.73, 75.76, 75.23, 68.01, 67.03, 60.41, 60.16, 52.25, 51.65, 46.97, 36.82, 35.87. HRMS (ESI$^+$) m/z calculated for $C_{26}H_{22}INO_5$ $[M+H]^+$ 556.0621; found 556.0643.

(2*R*,4*R*)-1-(((9H-fluoren-9-yl)methoxy)carbonyl)-4-(3-iodophenoxy)pyrrolidine-2-carboxylic acid (**10cii**): Synthesized following general procedure A, Part 2. Yield: 29%.

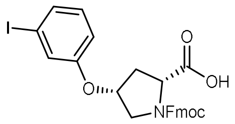

^{1}H NMR (400 MHz, $CDCl_3$) (1:1 mixture of rotamers) δ 7.80 – 7.72 (m, 2H), 7.63 – 7.55 (m, 2H), 7.47 – 7.25 (m, 5H), 7.16 (app. dt, *J* = 19.1, 2.0 Hz, 1H), 6.99 (app. dt, *J* = 21.4, 8.2 Hz, 1H), 6.77 (app. ddd, *J* = 20.6, 8.3, 2.5 Hz, 1H), 4.98 – 4.87 (m, 1H), 4.69 – 4.51 (m, 2H), 4.51 – 4.34 (m, 2H), 4.27 (app. dt, *J* = 25.2, 6.5 Hz, 1H), 3.77 (app. dd, *J* = 22.4, 3.0 Hz, 2H), 2.63 (app. dd, *J* = 25.4, 14.0 Hz, 1H), 2.54 – 2.41 (m, 1H). ^{13}C NMR (101 MHz, $CDCl_3$) δ 177.37, 176.45, 156.86, 156.81, 155.18, 154.54, 143.93, 143.86, 143.76, 143.66, 141.40, 141.29, 131.02, 130.96, 130.88, 130.80, 127.79, 127.71, 127.12, 125.11, 124.96, 124.87, 119.99, 115.26, 115.05, 94.48, 77.26, 75.52, 74.60, 67.85, 67.67, 57.88, 57.41, 52.11, 51.82, 47.19, 36.42, 35.10. HRMS (ESI$^+$) m/z calculated for $C_{26}H_{22}INO_5$ $[M+H]^+$ 556.0621; found 556.0649.

(2*R*,4*S*)-1-(((9H-fluoren-9-yl)methoxy)carbonyl)-4-(3-iodophenoxy)pyrrolidine-2-carboxylic acid (**10dii**): Synthesized following general procedure A, Part 2. Yield: 60%.

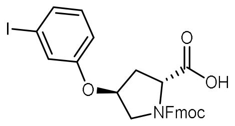

^{1}H NMR (400 MHz, Methanol-*d*$_4$) (55:45 mixture of rotamers) δ 7.68 – 7.54 (m, 3H), 7.44 (d, *J* = 7.5 Hz, 1H), 7.38 (d, *J* = 7.5 Hz, 1H), 7.30 – 7.09 (m, 6H), 7.06 (t, *J* = 7.5 Hz, 1H), 6.99 – 6.90 (m, 1H), 6.87 – 6.82 (m, 0H), 6.79 (dd, *J* = 8.5, 2.5 Hz, 1H), 4.94 – 4.88 (m, 0H), 4.85 – 4.81 (m, 1H), 4.45 – 4.33 (m, 1H), 4.24 – 4.05 (m, 3H), 3.75 (dd, *J* = 11.9, 4.0 Hz, 0H), 3.70 (dt, *J* = 12.2, 1.9 Hz, 0H), 3.61 (dd, *J* = 12.1, 3.9 Hz, 1H), 3.52 (dt, *J* = 12.0, 1.4 Hz, 1H), 2.56 – 2.49 (m, 0H), 2.43 – 2.35 (m, 1H), 2.22 (ddd, *J* = 13.3, 7.9, 4.8 Hz, 0H), 2.08 (ddd, *J* = 13.6, 8.7, 4.7 Hz, 1H). ^{13}C NMR (101 MHz, CD_3OD) δ 178.01, 177.92, 157.83, 157.75, 155.73, 155.25, 144.27, 144.03, 143.75, 143.69, 141.19, 141.17, 141.10, 141.00, 130.87, 130.79, 130.17, 130.14, 127.38, 127.34, 126.91, 126.88, 126.73, 126.68, 125.34, 125.16, 124.71, 124.65, 124.60, 124.49, 119.54, 119.47, 119.43, 114.77, 114.73, 93.81, 93.68, 75.75, 75.23, 68.01, 67.02, 60.31, 60.12, 52.24, 51.62, 46.96, 36.80, 35.88. HRMS (ESI$^+$) m/z calculated for $C_{26}H_{22}INO_5$ [M+H]$^+$ 556.0621; found 556.0643.

(2*S*,4*S*)-1-(((9H-fluoren-9-yl)methoxy)carbonyl)-4-((4-iodobenzyl)oxy)pyrrolidine-2-carboxylic acid (**14ai**): Synthesized following general procedure B, Part 3. Yield: 62%.

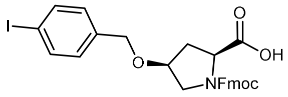

^{1}H NMR (400 MHz, $CDCl_3$) (57:43 mixture of rotamers) δ 7.73 – 7.57 (m, 2H), 7.56 – 7.37 (m, 4H), 7.35 – 7.17 (m, 4H), 6.85 (dd, *J* = 31.3, 7.9 Hz, 2H), 4.50 – 3.99 (m, 7H), 3.67 – 3.42 (m, 2H), 2.54 (d, *J* = 13.7 Hz, 0.57H), 2.38 – 2.05 (m, 1.43H). ^{13}C NMR (101 MHz, $CDCl_3$) δ 176.44, 174.85, 156.03, 154.66, 143.75, 143.61, 141.34, 141.24, 137.46, 137.16, 129.49, 129.29, 127.80, 127.14, 125.12, 124.98, 124.87, 120.01, 93.21, 93.09, 77.26, 75.99, 68.01, 67.68, 58.18, 57.46, 52.48, 52.10, 47.18, 36.24, 33.81. HRMS (ESI$^+$) m/z calculated for $C_{27}H_{24}INO_5$ [M+H]$^+$ 570.0777; found 570.0792.

(2*S*,4*R*)-1-(((9H-fluoren-9-yl)methoxy)carbonyl)-4-((4-iodobenzyl)oxy)pyrrolidine-2-carboxylic acid (**14bi**): Synthesized following general procedure B, Part 3. Yield: 79%.

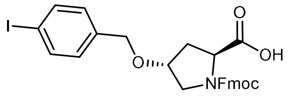

^{1}H NMR (400 MHz, Methanol-*d*$_4$) (55:45 mixture of rotamers) δ 7.65 – 7.57 (m, 2H), 7.52 – 7.37 (m, 4H), 7.25 – 7.17 (m, 2H), 7.12 (qd, *J* = 7.9, 3.0 Hz, 2H), 6.90 (dd, *J* = 8.3, 4.4 Hz, 2H), 4.34 (t, *J* = 7.8 Hz, 0.45H), 4.29 – 4.09 (m, 4.55H), 4.07 – 3.97 (m, 2H), 3.61 (d, *J* = 11.7 Hz, 0.45H), 3.47 – 3.40 (m, 1H),

3.37 (dd, J = 11.7, 4.3 Hz, 0.55H), 2.41 – 2.24 (m, 1H), 2.01 (ddd, J = 13.1, 7.6, 5.0 Hz, 0.45H), 1.92 (ddd, J = 13.9, 8.3, 5.3 Hz, 0.55H). ^{13}C NMR (101 MHz, CD_3OD) δ 174.63, 174.39, 155.30, 155.22, 143.88, 143.85, 143.68, 143.63, 141.20, 141.14, 141.07, 137.99, 137.90, 137.26, 129.32, 129.22, 127.49, 126.86, 124.89, 124.85, 124.75, 119.65, 119.62, 92.45, 77.10, 76.28, 69.89, 69.79, 67.91, 67.30, 57.97, 57.73, 51.94, 51.51, 46.97, 46.91, 36.41, 35.38. HRMS (ESI$^+$) m/z calculated for $C_{27}H_{24}INO_5$ $[M+H]^+$ 570.0777; found 570.0794.

(2*R*,4*R*)-1-(((9H-fluoren-9-yl)methoxy)carbonyl)-4-((4-iodobenzyl)oxy)pyrrolidine-2-carboxylic acid (**14ci**): Synthesized following general procedure B, Part 3. Yield: 75%.

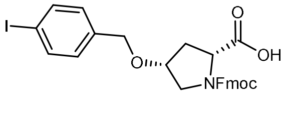

^{1}H NMR (400 MHz, Methanol-d_4) δ 7.58 (d, J = 7.5 Hz, 2H), 7.50 – 7.37 (m, 4H), 7.20 (t, J = 7.5 Hz, 2H), 7.17 – 7.07 (m, 2H), 6.87 (t, J = 7.3 Hz, 2H), 4.29 – 4.06 (m, 5H), 4.01 (dt, J = 13.9, 6.8 Hz, 1H), 3.94 – 3.87 (m, 1H), 3.47 – 3.31 (m, 2H), 2.28 – 2.18 (m, 1H), 2.15 – 2.02 (m, 1H). ^{13}C NMR (101 MHz, CD_3OD) δ 175.80, 175.55, 157.12, 157.05, 145.85, 145.81, 145.61, 145.53, 143.07, 143.02, 142.95, 139.88, 138.98, 138.95, 131.04, 131.02, 129.37, 128.75, 126.78, 126.72, 126.68, 121.52, 94.10, 94.05, 79.11, 78.23, 71.38, 69.50, 69.21, 59.65, 59.54, 53.99, 53.71, 48.90, 48.83, 37.38, 36.39. HRMS (ESI$^+$) m/z calculated for $C_{27}H_{24}INO_5$ $[M+H]^+$ 570.0777; found 570.0791.

(2*R*,4*S*)-1-(((9H-fluoren-9-yl)methoxy)carbonyl)-4-((4-iodobenzyl)oxy)pyrrolidine-2-carboxylic acid (**14di**): Synthesized following general procedure B, Part 3. Yield: 72%.

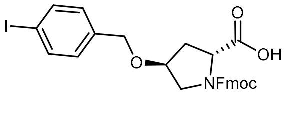

^{1}H NMR (400 MHz, Methanol-d_4) (55:45 mixture of rotamers) δ 7.59 (dd, J = 8.1, 3.4 Hz, 2H), 7.52 – 7.35 (m, 4H), 7.25 – 7.16 (m, 2H), 7.16 – 7.05 (m, 2H), 6.88 (dd, J = 8.2, 3.7 Hz, 2H), 4.34 (t, J = 7.8 Hz, 0.45H), 4.28 – 4.07 (m, 4.55H), 4.05 – 3.95 (m, 2H), 3.60 (d, J = 11.7 Hz, 0.45H), 3.48 – 3.40 (m, 1H), 3.36 (dd, J = 11.7, 4.3 Hz, 0.55H), 2.40 – 2.22 (m, 1H), 2.00 (ddd, J = 13.0, 7.6, 4.9 Hz, 0.45H), 1.91 (ddd, J = 13.5, 8.1, 5.0 Hz, 0.55H). ^{13}C NMR (101 MHz, CD_3OD) δ 174.63, 174.39, 155.29, 155.21, 143.88, 143.84, 143.67, 143.63, 141.19, 141.18, 141.13, 141.07, 137.96, 137.87, 137.26, 129.32, 129.22, 127.51, 126.88, 124.91, 124.87, 124.77, 119.67, 119.64, 92.50, 77.10, 76.27, 69.89, 69.80, 67.91, 67.31, 57.98, 57.75, 51.95, 51.53, 46.97, 46.91, 36.41, 35.37. HRMS (ESI$^+$) m/z calculated for $C_{27}H_{24}INO_5$ $[M+H]^+$ 570.0777; found 570.0788.

(2*S*,4*S*)-1-(((9H-fluoren-9-yl)methoxy)carbonyl)-4-((3-iodobenzyl)oxy)pyrrolidine-2-carboxylic acid (**14aii**): Synthesized following general procedure B, Part 3. Yield: 49%.

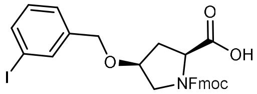

^{1}H NMR (400 MHz, $CDCl_3$) (57:43 mixture of rotamers) δ 10.11 (br. s, 1H), 7.62 (dd, *J* = 25.6, 7.6 Hz, 2H), 7.54 – 7.36 (m, 4H), 7.33 – 7.03 (m, 5H), 6.91 (app. dt, *J* = 13.9, 7.7 Hz, 1H), 4.47 – 4.12 (m, 5.57H), 4.08 – 3.92 (m, 1.43H), 3.62 – 3.45 (m, 2H), 2.44 (d, *J* = 13.7 Hz, 0.57H), 2.30 (d, *J* = 13.7 Hz, 0.43H), 2.24 – 2.05 (m, 1H). ^{13}C NMR (101 MHz, $CDCl_3$) δ 176.65, 175.40, 155.75, 154.73, 143.75, 143.66, 141.33, 141.22, 139.97, 136.77, 136.37, 136.23, 130.28, 127.81, 127.72, 127.18, 126.79, 126.66, 125.18, 125.04, 124.96, 120.03, 94.34, 76.74, 75.88, 69.68, 67.96, 67.79, 58.01, 57.44, 52.37, 52.19, 47.19, 36.04, 34.01. HRMS (ESI$^+$) m/z calculated for $C_{27}H_{24}INO_5$ $[M+H]^+$ 570.0777; found 570.0790.

(2*S*,4*R*)-1-(((9H-fluoren-9-yl)methoxy)carbonyl)-4-((3-iodobenzyl)oxy)pyrrolidine-2-carboxylic acid (**14bii**): Synthesized following general procedure B, Part 3. Yield: 80%.

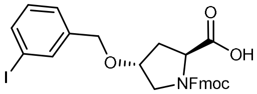

^{1}H NMR (400 MHz, Methanol-d_4) (54:46 mixture of rotamers) δ 7.63 – 7.55 (m, 2H), 7.51 (d, *J* = 5.5 Hz, 1H), 7.48 – 7.35 (m, 3H), 7.24 – 7.16 (m, 2H), 7.16 – 7.06 (m, 3H), 6.90 (q, *J* = 7.6 Hz, 1H), 4.34 (t, *J* = 7.9 Hz, 0.46H), 4.30 – 4.11 (m, 4.54H), 4.06 – 3.95 (m, 2H), 3.62 (dt, *J* = 12.0, 2.1 Hz, 0.46H), 3.43 (dd, *J* = 12.1, 3.4 Hz, 1H), 3.35 (dd, *J* = 11.7, 4.3 Hz, 0.54H), 2.40 – 2.23 (m, 1H), 2.00 (ddd, *J* = 13.0, 7.5, 4.9 Hz, 0.46H), 1.91 (ddd, *J* = 13.2, 7.9, 4.8 Hz, 0.54H). ^{13}C NMR (101 MHz, CD_3OD) δ 174.64, 174.39, 155.29, 155.23, 143.87, 143.84, 143.69, 143.65, 141.19, 141.13, 141.07, 140.72, 140.66, 136.47, 136.19, 136.12, 129.98, 129.96, 127.50, 126.89, 126.53, 126.47, 124.91, 124.89, 124.76, 124.73, 119.65, 119.62, 93.75, 93.71, 77.17, 76.40, 69.61, 69.51, 67.93, 67.30, 57.96, 57.74, 51.92, 51.49, 46.98, 46.93, 36.41, 35.36. HRMS (ESI$^+$) m/z calculated for $C_{27}H_{24}INO_5$ $[M+H]^+$ 570.0777; found 570.0810.

(2*R*,4*R*)-1-(((9H-fluoren-9-yl)methoxy)carbonyl)-4-((3-iodobenzyl)oxy)pyrrolidine-2-carboxylic acid (**14cii**): Synthesized following general procedure B, Part 3. Yield: 86%.

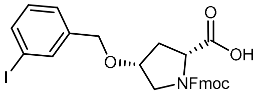

^{1}H NMR (400 MHz, Methanol-d_4) (1:1 mixture of rotamers) δ 7.82 – 7.75 (m, 2H), 7.74 – 7.57 (m, 4H), 7.38 (td, *J* = 7.3, 2.4 Hz, 2H), 7.35 – 7.27 (m, 3H), 7.09 (td, *J* = 7.7, 5.9 Hz, 1H), 4.50 – 4.31 (m, 5H), 4.27 (t, *J* = 6.6 Hz, 0.5H), 4.23 – 4.15 (m, 1.5H), 3.64 (dd, *J* = 11.8, 4.9 Hz, 0.5H), 3.61 – 3.54 (m, 1.5H), 2.47

– 2.28 (m, 2H). ^{13}C NMR (101 MHz, CD_3OD) δ 174.15, 173.87, 155.39, 155.31, 144.05, 144.00, 143.75, 143.72, 141.27, 141.21, 141.19, 141.10, 140.81, 136.31, 136.27, 136.05, 136.03, 129.84, 129.82, 127.43, 126.83, 126.46, 124.85, 124.79, 124.77, 119.52, 119.48, 93.37, 93.32, 77.31, 76.47, 69.38, 69.33, 67.63, 67.38, 57.82, 57.74, 52.08, 51.78, 47.08, 47.02, 35.47, 34.52. HRMS (ESI$^+$) m/z calculated for $C_{27}H_{24}INO_5$ $[M+H]^+$ 570.0777; found 570.0793.

(2*R*,4*S*)-1-(((9H-fluoren-9-yl)methoxy)carbonyl)-4-((3-iodobenzyl)oxy)pyrrolidine-2-carboxylic acid (**14dii**): Synthesized following general procedure B, Part 3. Yield: 90%.

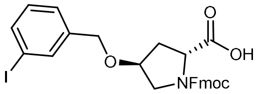

^{1}H NMR (400 MHz, Methanol-d_4) (55:45 mixture of rotamers) δ 7.62 – 7.55 (m, 2H), 7.51 (d, *J* = 5.3 Hz, 1H), 7.47 – 7.36 (m, 3H), 7.24 – 7.15 (m, 2H), 7.15 – 7.06 (m, 3H), 6.90 (q, *J* = 7.7 Hz, 1H), 4.34 (t, *J* = 7.9 Hz, 0.45H), 4.29 – 4.19 (m, 2.55H), 4.19 – 4.09 (m, 2H), 4.05 – 3.95 (m, 2H), 3.65 – 3.57 (m, 0.45H), 3.47 – 3.39 (m, 1H), 3.34 (dd, *J* = 11.7, 4.3 Hz, 0.55H), 2.39 – 2.23 (m, 1H), 2.00 (ddd, *J* = 13.1, 7.6, 4.9 Hz, 0.45H), 1.91 (ddd, *J* = 13.2, 7.9, 4.8 Hz, 0.55H). ^{13}C NMR (101 MHz, CD_3OD) δ 174.64, 174.39, 155.29, 155.23, 143.86, 143.84, 143.69, 143.65, 141.18, 141.12, 141.07, 140.71, 140.65, 136.47, 136.19, 136.11, 129.99, 129.97, 127.50, 126.89, 126.54, 126.47, 124.91, 124.90, 124.76, 124.74, 119.66, 119.63, 93.77, 93.73, 77.17, 76.40, 69.61, 69.52, 67.93, 67.31, 57.96, 57.74, 51.92, 51.49, 46.98, 46.93, 36.41, 35.35. HRMS (ESI$^+$) m/z calculated for $C_{27}H_{24}INO_5$ $[M+H]^+$ 570.0777; found 570.0800.

1.3. Spirocyclic azetidines

(*R*)-4-benzyl-3-((3*S*,4*R*)-1-benzyl-4-(4-iodophenyl)pyrrolidine-3-carbonyl)oxazolidin-2-one **(15)**
(*R*)-4-benzyl-3-((3*R*,4*S*)-1-benzyl-4-(4-iodophenyl)pyrrolidine-3-carbonyl)oxazolidin-2-one **(16)**

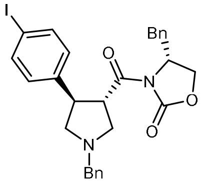

15

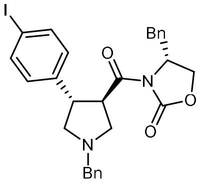

16

To a 250 mL round-bottom flask was added 4-iodobenzaldehyde (11.6 g, 50 mmol, 1 eq) and malonic acid (6.25 g, 60 mmol, 1.2 eq), the mixture was dissolved with pyridine (50 mL), then 0.1 mL of piperidine was added. The resulting mixture was stirred under reflux for 6 h, cooled to rt and poured into 500 mL ice-water, then adjusted the pH to ~3 by adding concentrated HCl. The precipitated white solid was collected by filtration and washed with water for 3 times. (*E*)-3-(4-iodophenyl)acrylic acid (13.8 g, quantitative yield), a white solid, was dried under air and used for next step directly.

To a 250 mL round-bottom flask was added *E*)-3-(4-iodophenyl)acrylic acid (13.7 g, 50 mmol) and dissolved with CH_2Cl_2 (120 mL), 5 drops of DMF was added. To this solution was added oxalyl chloride (6.0 mL, 70 mmol, 1.4 eq) slowly while stirring, after which the reaction mixture was stirred for 2 hr at rt. The volatiles were evaporated under reduced pressure and dried under vacuum to afford (*E*)-3-(4-iodophenyl)acryloyl chloride (14.7 g, quantitative yield, stored under N_2 before use) as a white solid.

To a 250 mL round-bottom flask was added NaH (2.4 g, 60 mmol, 60% wt in mineral oil) and MeCN (120 mL). The mixture was stirred under an ice-bath and (*R*)-4-benzyloxazolidin-2-one (8.9 g, 50 mmol) was added in portions. The reaction mixture was stirred at rt for 2 h, then cooled with an ice-bath again. (*E*)-3-(4-iodophenyl)acryloyl chloride (14.6 g, 50 mmol) was added in portions, then the mixture was stirred at rt overnight. The reaction was quenched with 5 mL of water and the organic solvent was evaporated under reduced pressure. To the resulting solid was added 200 mL of water and stirred for 30 min, the solid was collected by filtration and washed with water twice. (*R*,*E*)-4-benzyl-3-(3-(4-iodophenyl)acryloyl)oxazolidin-2-one (19.0 g, 88% yield) was isolated as an off-white solid and used directly in the next step.

To a 250 mL round-bottom flask was added (*R*,*E*)-4-benzyl-3-(3-(4-iodophenyl)acryloyl)oxazolidin-2-one (17.4 g, 40 mmol, 1 eq) and *N*-benzyl-1-methoxy-*N*-((trimethylsilyl)methyl)methanamine (10.5 g, 44 mmol, 1.1 eq), the mixture was dissolved with anhydrous CH_2Cl_2 (150 mL) and cooled to 0 °C. To the cooled solution was added 5 drops of trifluoroacetic acid and the reaction mixture was stirred for 30 min 0 °C. The reaction was warmed to rt and stirred for another 4 h. The reaction mixture was washed with 1 M aq. $NaHCO_3$ solution, the organic phase was collected and dried with anhydrous Na_2SO_4. The solvent evaporated and the residue was purified by flash column chromatography (hexanes/ethyl acetate: 3:1 to 2:1). The two diastereomers were obtained in 42% yield **15** (Rf: ~0.4, hexanes/ethyl acetate = 2:1) and 43% yield for **16** (Rf: ~0.25, hexanes/ethyl acetate = 2:1) respectively.

(*R*)-4-benzyl-3-((*3S,4R*)-1-benzyl-4-(4-iodophenyl)pyrrolidine-3-carbonyl)oxazolidin-2-one (**15**)

Pale yellow solid, upper spot on TLC, 42% yield. $[\alpha]^{20}_D$ = -68.00 (c=1, $CHCl_3$); ^{1}H NMR (600 MHz, $CDCl_3$) δ 7.63 (d, *J* = 8.4 Hz, 2H), 7.41 (d, *J* = 7.4 Hz, 2H), 7.36 (t, *J* = 7.5 Hz, 2H), 7.32 – 7.28 (m, 4H), 7.22 – 7.18 (m, 2H), 7.11 (d, *J* = 8.4 Hz, 2H), 4.71 – 4.63 (m, 1H), 4.17 (q, *J* = 5.2, 4.6 Hz, 2H), 4.09 (h, *J* = 7.4 Hz, 2H), 3.81 (d, *J* = 13.0 Hz, 1H), 3.70 (d, *J* = 13.5 Hz, 1H), 3.33 (t, *J* = 9.0 Hz, 1H), 3.28 – 3.24 (m, 1H), 3.23 (m, 1H), 2.92-2.86 (m, 1H), 2.81 (dd, *J* = 13.4, 9.3 Hz, 1H), 2.70 (t, *J* = 8.0 Hz, 1H). ^{13}C NMR (151 MHz, $CDCl_3$) δ 173.10, 153.22, 137.80, 135.14, 130.09, 129.62, 129.14, 128.94, 128.59, 127.61, 127.44, 92.13, 66.47, 61.69, 59.90, 58.24, 55.41, 51.47, 44.91, 37.99; HRMS (ESI-TOF) m/z calculated for $C_{28}H_{28}IN_2O_3$ $[M+H]^+$ 567.1139; found: 567.1142.

(*R*)-4-benzyl-3-((*3R,4S*)-1-benzyl-4-(4-iodophenyl)pyrrolidine-3carbonyl)oxazolidin-2-one (**16**)

Pale yellow solid, lower spot on TLC, 43% yield. $[\alpha]^{20}_D$ = -4.10 (c=1, $CHCl_3$); ^{1}H NMR (600 MHz, $CDCl_3$) δ 7.67 (d, *J* = 8.4 Hz, 2H), 7.39 (d, *J* = 7.2 Hz, 2H), 7.34 (t, *J* = 7.5 Hz, 2H), 7.30 – 7.23 (m, 4H), 7.15 (d, *J* = 8.4 Hz, 2H), 7.08 (dd, *J* = 7.6, 1.7 Hz, 2H), 4.73 – 4.66 (m, 1H), 4.26 (ddd, *J* = 9.4, 7.5, 6.1 Hz, 1H), 4.22

– 4.18 (m, 1H), 4.15 (dd, J = 9.0, 2.9 Hz, 1H), 4.05 (q, J = 7.7 Hz, 1H), 3.81 – 3.66 (m, 2H), 3.27 (t, J = 9.6 Hz, 1H), 3.22 – 3.16 (m, 2H), 2.84 (dd, J = 9.7, 6.1 Hz, 1H), 2.78 (dd, J = 13.5, 9.1 Hz, 1H), 2.74 – 2.69 (m, 1H); ^{13}C NMR (151 MHz, $CDCl_3$) δ 173.49, 153.16, 137.79, 135.03, 130.04, 129.54, 129.09, 128.90, 128.52, 127.56, 127.34, 92.13, 66.24, 61.57, 59.95, 58.06, 55.55, 51.08, 45.70, 37.75; HRMS (ESI-TOF) m/z calculated for $C_{28}H_{28}IN_2O_3$ $[M+H]^+$ 567.1139; found 567.1144.

1-(*tert*-butyl) 3-methyl (*3R,4S*)-4-(4-iodophenyl)pyrrolidine-1,3-dicarboxylate (**17**)

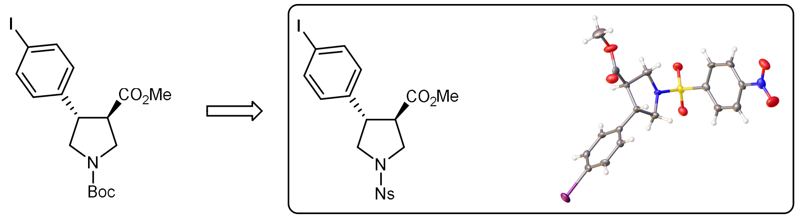

To a 250-mL round-bottom flask was added **15** (8.5 g, 15 mmol, 1 eq), dissolved with toluene (120 mL), then 1-chloroethyl chloroformate (4.3 g, 30 mmol) was added. The resulting solution was stirred under reflux, a lot of solid was precipitated during the reaction, the reflux was continued until the solid disappeared again (2-4 hr). The resulting solution was cooled to rt, and the solvent was evaporated under reduced pressure, the residue was dissolved with 50 mL of MeOH and heated at reflux for 1 h. The solvent was evaporated, and the residue was dissolved with saturated aq. $NaHCO_3$ solution (100 mL) and THF (50 mL), then Boc_2O (4.4 g, 20 mmol, 1.3 eq) was added, the resulting solution was stirred at rt overnight. The reaction solution was extracted with ethyl acetate twice and the combined organic portion was dried with anhydrous Na_2SO_4. After filtration the solution was concentrated, and the residue was purified by flash column chromatography (hexanes/ethyl acetate: 3:1 to 2:1) to obtain *tert*-butyl (3*R*,4*S*)-3-((*R*)-4-benzyl-2-oxooxazolidine-3-carbonyl)-4-(4-iodophenyl)pyrrolidine-1-carboxylate (7.9 g, 13.7 mmol, 91% yield).

To a 250-mL round-bottom flask was added *tert*-butyl (3*R*,4*S*)-3-((*R*)-4-benzyl-2-oxooxazolidine-3-carbonyl)-4-(4-iodophenyl)pyrrolidine-1-carboxylate (7.5 g, 13 mmol, 1 eq), dissolved in THF (50 mL), then aq. LiOH (30 mL, 2.0 M) was added, followed by the addition of aq. H_2O_2 (4 mL, 30%v/v). The resulting solution was stirred at rt overnight. The mixture was acidified with aq. HCl (50 mL, 2.0 M) and extracted with EtOAc (5 times). The organic portions were combined and evaporated under reduced pressure, the residue was dissolved with DMF (50 mL), K_2CO_3 (3.6 g, 26 mmol, 2 eq) and MeI (1.7 mL, 26 mmol, 2 eq) were added sequentially, the resulting mixture was stirred at rt for 8 h. The reaction solution was diluted with 100 mL of water and extracted with EtOAc 3 times. The organic portions were combined and washed with water twice and then dried with anhydrous Na_2SO_4. The solvent was concentrated, and the residue was purified by flash column chromatography (hexanes/ethyl acetate: 3:1) to provide **17** (3.75 g, 8.7 mmol, 67% yield) as a white solid.

1-(*tert*-butyl) 3-methyl (*3R,4S*)-4-(4-iodophenyl)pyrrolidine-1,3-dicarboxylate (**17**)

99% ee, absolute configuration confirmed by x-ray of its Ns analogue, $[\alpha]^{20}D$ = 42.10 (c=1, $CHCl_3$). ^{1}H NMR (600 MHz, $CDCl_3$) δ 7.66 (d, *J* = 8.3 Hz, 2H), 7.02 (d, *J* = 8.4 Hz, 2H), 3.95 – 3.78 (m, 2H), 3.65 (s, 3H), 3.63 – 3.54 (m, 2H), 3.38 (q, *J* = 8.6 Hz, 1H), 3.15 (q, *J* = 9.1 Hz, 1H), 1.48 (s, 9H). ^{13}C NMR (151 MHz, $CDCl_3$) δ 172.93, 154.59, 139.52, 138.47, 129.94, 93.32, 80.49, 52.80, 50.90, 49.98, 49.33, 47.37, 29.09. HRMS (ESI-TOF) m/z calculated for $C_{17}H_{23}INO_4$ $[M+H]^+$ 432.0666; found 432.0669.

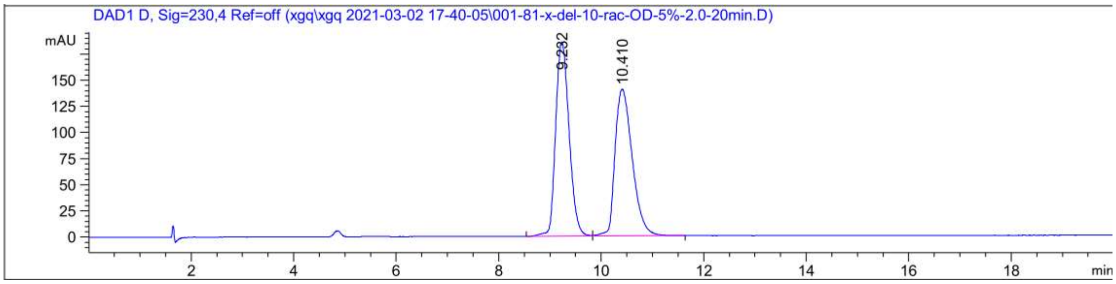

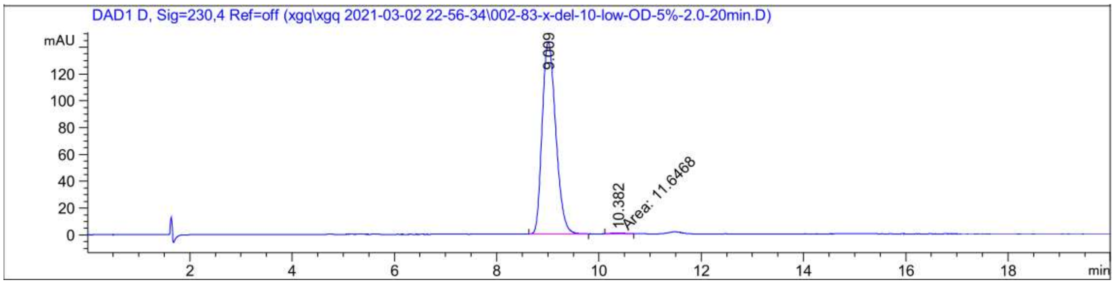

Signal 3: DAD1 D, Sig=230,4 Ref=off

Peak #	RetTime [min]	Type	Width [min]	Area [mAU*s]	Height [mAU]	Area %
1	9.009	BB	0.2908	2634.95508	143.08714	99.5599
2	10.382	MM	0.3021	11.64684	6.42448e-1	0.4401
Totals :				2646.60192	143.72959	

1-(*tert*-butyl) 3-methyl (3*S*,4*R*)-4-(4-iodophenyl)pyrrolidine-1,3-dicarboxylate (**18**) [prepared as for **17**]

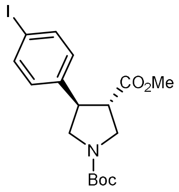

White solid, 96.5% ee. $[\alpha]^{20}_D$ = -41.10 (c=1, $CHCl_3$). ^{1}H NMR (600 MHz, $CDCl_3$) δ 7.66 (d, *J* = 8.3 Hz, 2H), 7.02 (d, *J* = 8.4 Hz, 2H), 3.94 – 3.81 (m, 2H), 3.65 (s, 3H), 3.63 – 3.56 (m, 2H), 3.38 (q, *J* = 8.6 Hz, 1H),

3.15 (q, J = 9.1 Hz, 1H), 1.48 (s, 9H). ^{13}C NMR (151 MHz, $CDCl_3$) δ 172.49, 154.15, 139.08, 138.03, 129.50, 92.88, 80.05, 52.36, 50.46, 49.54, 48.89, 46.93, 28.65. HRMS (ESI-TOF) m/z calculated for $C_{17}H_{23}INO_4$ $[M+H]^+$ 432.0666; found 432.0667.

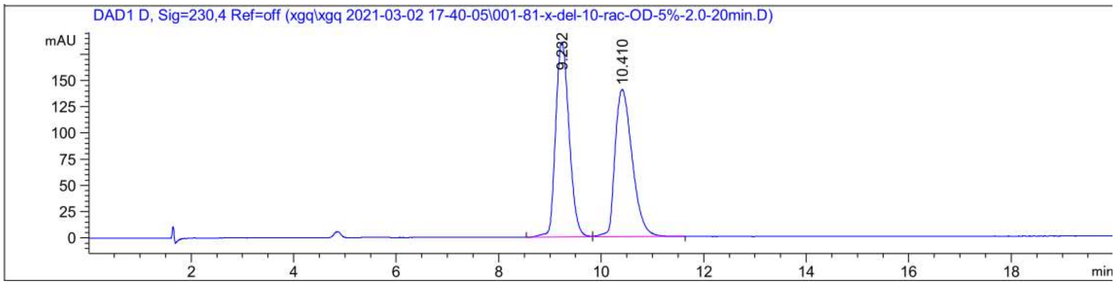

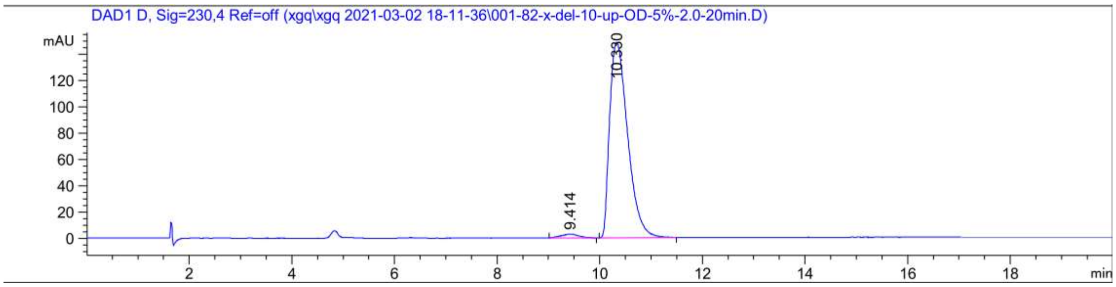

Signal 3: DAD1 D, Sig=230,4 Ref=off

Peak #	RetTime [min]	Type	Width [min]	Area [mAU*s]	Height [mAU]	Area %
1	9.414	BB	0.3026	65.23700	2.77138	1.7703
2	10.330	BB	0.3866	3619.87378	148.36899	98.2297
Totals :				3685.11078	151.14037	

tert-butyl (*R*)-8-(4-iodophenyl)-2-((4-nitrophenyl)sulfonyl)-2,6-diazaspiro[3.4] octane-6-carboxylate (**19**)

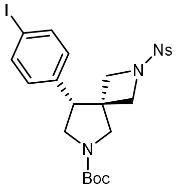

A flame-dried 100 mL round-bottom flask equipped with a magnetic stir bar was evacuated and backfilled with N_2 3 times. To this flask was added diisopropylamine (0.45 mL, 3.2 mmol, 1.6 eq) and anhydrous THF (20 mL), cooled to -78 °C. ^nBuLi solution (1.2 mL, 2.5 M in hexane, 3.0 mmol, 1.5 eq) was added to the reaction slowly via a syringe, after addition the reaction was stirred at -78 °C for 15 min. **17** (0.87 g, 2.0 mmol, 1 eq) was dissolved in anhydrous THF (5 mL) and added to the reaction slowly via a syringe, after addition the reaction was stirred -78 °C for 2 h. 2-(Bromomethyl)isoindoline-1,3-dione (0.96 g, 4

mmol, 2 eq) was dissolved in anhydrous THF (5 mL) and added to the reaction slowly via a syringe, after addition the reaction was warmed up to rt slowly and stirred overnight. Saturated aq. NH_4Cl (50 mL) was added to the reaction and the reaction mixture was extracted with EtOAc (30 mL) 3 times. The combined organic portions were dried with anhydrous Na_2SO_4 and then filtrated, the solvent was removed under reduced pressure and the residue was purified by flash column chromatography (hexanes/ethyl acetate: 3:1) to give the alkylated product 1-(*tert*-butyl) 3-methyl (3*R*,4*R*)-3-((1,3-dioxoisoindolin-2-yl)methyl)-4-(4-iodophenyl)pyrrolidine-1,3-dicarboxylate (602 mg, 51% yield), which was used directly in subsequent steps.

To a 12 mL reaction vial was added 1-(*tert*-butyl) 3-methyl (3*R*,4*R*)-3-((1,3-dioxoisoindolin-2-yl)methyl)-4-(4-iodophenyl)pyrrolidine-1,3-dicarboxylate (590 mg, 1.0 mmol, 1 eq), dissolved with methanol (5 mL), then hydrazine monohydrate (0.1 mL, 2.0 mmol, 2 eq) was added, the vial was capped and stirred at 50 °C for 12 h. The reaction was cooled to rt and diluted with CH_2Cl_2 (15 mL), the white solid precipitate was filtered off and washed with CH_2Cl_2 (10 mL). The combined organic solution was concentrated and re-dissolved in anhydrous THF (5 mL), then lithium borohydride (1.5 mL, 2.0 M in THF, 3 eq) was added, and the resulting solution was stirred at 50 °C for 12 h. After the reaction was cooled to rt the solution was poured into 30 mL of ice-water, the aqueous solution was then extracted with EtOAc (20 mL) 5 times. The organic portions were combined and concentrated to provide *tert*-butyl (3*R*,4*R*)-3-(aminomethyl)-3-(hydroxymethyl)-4-(4-iodophenyl)pyrrolidine-1-carboxylate as a white foam, which was used directly in the next step.

To a 12 mL vial was added *tert*-butyl (3*R*,4*R*)-3-(aminomethyl)-3-(hydroxymethyl)-4-(4-iodophenyl)pyrrolidine-1-carboxylate (~1.0 mmol, 1 eq) which was prepared in the last step and 2-nitrobenzenesufonyl chloride (270 mg, 1.2 mmol, 1.2 eq), dissolved in CH_2Cl_2 (6 mL), then 2,6-lutidine (0.5 mL, 4.3 mmol, 4.3 eq) was added. The resulting solution was stirred at rt for 12 h, after which the reaction mixture was washed with 0.5 M aq. HCl (20 mL), and the aqueous phase was extracted with CH_2Cl_2 (10 mL) 3 times. The combined organic portions were dried with anhydrous Na_2SO_4 and concentrated. The residue was re-dissolved with CH_2Cl_2 (10 mL) and NEt_3 (0.3 mL, 2.2 mmol, 2.2 eq) was added. The resulting solution was cooled to 0 °C and methanesulfonyl chloride (0.15 mL, 2.0 mmol, 2 eq) was added dropwise, then after addition the reaction was stirred at 0 °C for 30 min, then quenched with saturated aq. NH_4Cl (10 mL). The aqueous phase was extracted with CH_2Cl_2 (10 mL) twice and dried with anhydrous Na_2SO_4. The solvent was concentrated, and the residue was re-dissolved MeCN (6 mL), then K_2CO_3 (280 mg, 2.0 mmol, 2 eq) was added. The resulting mixture was stirred at 80 °C for 12 h, cooled to rt and poured into water (20 mL), the aqueous solution was then extracted with EtOAc (20 mL) 3 times. The combined organic portions were dried with anhydrous Na_2SO_4, filtered, and concentrated under reduced pressure. The residue was purified by flash column chromatography (hexanes/ethyl acetate: 3:1 to 2:1) to give the spiro azetidine **19** (366 mg, 51% over 5 steps) as a pale brown foam.

Mixture of 2 rotamers, 97.5% ee. $[\alpha]^{20}_{D}$ = 1.30 (c=1, $CHCl_3$). ^{1}H NMR (600 MHz, $CDCl_3$) δ 7.86-7.77 (m, 1.6H), 7.70-7.69 (m, 0.23H), 7.66-7.64 (m, 1.77H), 7.57 (d, *J* = 8.4 Hz, 1.4H), 7.30 (t, *J* = 7.2 Hz, 0.72H), 7.24 (t, *J* = 7.2 Hz, 0.28H), 7.12 (d, *J* = 7.2 Hz, 0.58H), 6.86 (d, *J* = 7.2 Hz, 1.42H), 4.04-3.97 (m, 2H), 3.89 (d, *J* = 8.4 Hz, 0.29H), 3.81 (d, *J* = 8.4 Hz, 0.71H), 3.74-3.54 (m, 5H), 3.34-3.30 (m, 0.28H), 3.28-3.21 (m, 0.72H), 1.49 (s, 9H). ^{13}C NMR (151 MHz, $CDCl_3$) δ 154.32, 148.49, 138.03, 134.04, 131.95, 130.78, 129.99, 124.34, 93.54, 80.46, 61.15, 60.22, 56.16, 55.96, 55.14, 54.35, 50.15, 49.93, 49.22, 49.05, 42.95, 42.25, 28.61. HRMS (ESI-TOF) m/z calculated for $C_{23}H_{27}IN_3O_6S$ $[M+H]^+$ 600.0660; found 600.0664.

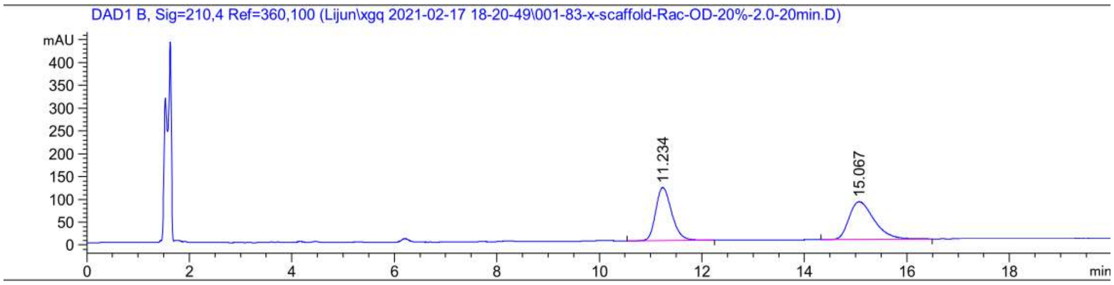

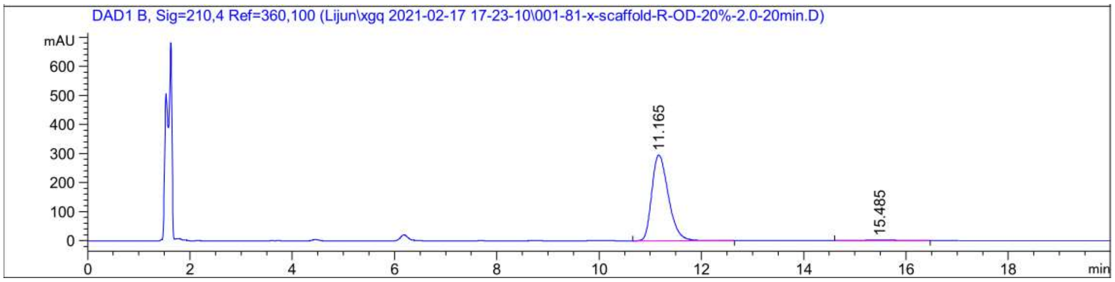

Peak #	RetTime [min]	Type	Width [min]	Area [mAU*s]	Height [mAU]	Area %
1	11.165	BB	0.3482	1069.92212	47.59830	98.7404
2	15.447	MM	0.5913	13.64903	3.84688e-1	1.2596
Totals :				1083.57115	47.98299	

tert-butyl (*S*)-8-(4-iodophenyl)-2-((4-nitrophenyl)sulfonyl)-2,6-diazaspiro[3.4] octane-6-carboxylate (**20**) [prepared as for **19**]

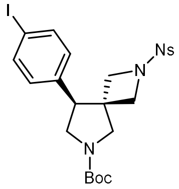

Pale yellow solid, 98.3% ee. $[\alpha]^{20}_D$ = -1.50 (c=1, $CHCl_3$). ^1H NMR (600 MHz, $CDCl_3$) δ 7.90 – 7.75 (m, 2H), 7.67 (d, *J* = 7.8 Hz, 2H), 7.64 – 7.54 (m, 2H), 6.92-6.84 (m, 2H), 4.07 – 3.95 (m, 2H), 3.87-3.80 (m, 1H), 3.76 – 3.55 (m, 5H), 3.33 – 3.20 (m, 1H), 1.52 (s, 9H). ^{13}C NMR (151 MHz, $CDCl_3$) δ 154.39, 154.31, 148.50, 148.47, 138.00, 134.12, 134.02, 132.03, 131.99, 130.78, 130.76, 130.04, 129.03, 128.00, 127.85, 124.36, 124.32, 93.57, 80.41, 80.26, 60.89, 60.06, 56.16, 56.00, 55.16, 54.53, 49.90, 49.08, 42.95, 42.40, 28.64, 28.61. HRMS (ESI-TOF) m/z calculated for $C_{23}H_{27}IN_3O_6S$ [M+H]+ 600.0660; found: 600.0661.

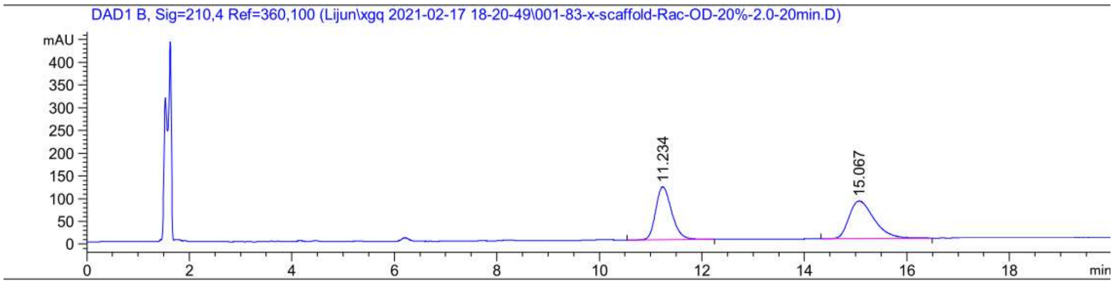

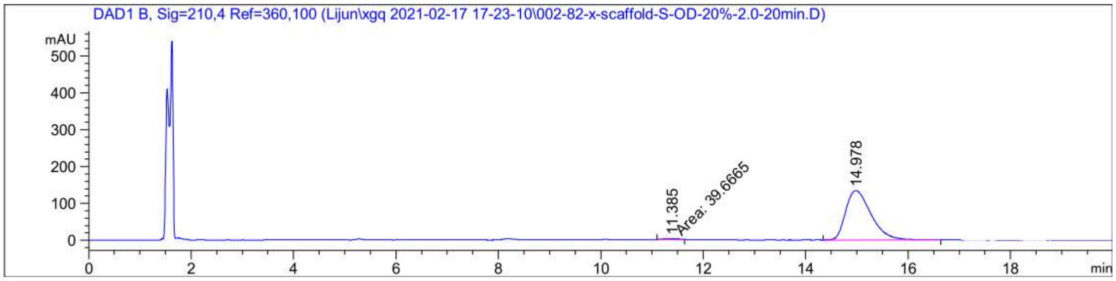

Peak #	RetTime [min]	Type	Width [min]	Area [mAU*s]	Height [mAU]	Area %
1	11.385	MM	0.3543	39.66647	1.86587	0.8539
2	14.978	BB	0.5354	4605.60449	133.87163	99.1461
Totals :				4645.27096	135.73749	

(*R*)-5-(8-(4-iodophenyl)-2-((4-nitrophenyl)sulfonyl)-2,6-diazaspiro[3.4]octan-6-yl)-5-oxopentanoic acid (**21**)

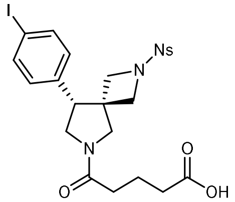

To a 12-mL vial was **19** (60 mg, 100 µmol, 1 eq), dissolved with CH_2Cl_2 (2 mL), then trifluoroacetic acid (0.5 mL) was added. The resulting solution was stirred at rt for 2 hr and then concentrated, the residue was dissolved with CH_2Cl_2 (2 mL), then NEt_3 (100 mg, 1.0 mmol, 10 eq) and glutaric anhydride (35 mg, 300 µmol, 3 eq) were added sequentially, and the resulting solution was stirred at rt overnight. The

solution was washed with 2.0 M aq. HCl (10 mL) and extracted with CH_2Cl_2 (10 mL) 3 times. The combined organic portions were dried over anhydrous Na_2SO_4. The solution was concentrated and purified by preparative TLC chromatography to **21** (58 mg, 95 μmol, 95% yield) as a colorless foam and mixture of rotamers.

$[\alpha]^{20}_{D}$ = -3.20 (c=1, $CHCl_3$); ^1H NMR (600 MHz, DMSO) δ 7.98 – 7.89 (m, 2H), 7.79 – 7.69 (m, 2H), 7.54 (dd, *J* = 13.5, 7.9 Hz, 2H), 6.94 (dd, *J* = 27.3, 7.6 Hz, 2H), 3.97 – 3.85 (m, 2.65H), 3.79 – 3.58 (m, 5.35H), 3.51 (dd, *J* = 19.8, 12.5 Hz, 1H), 2.34 – 2.20 (m, 4H), 1.78 – 1.67 (m, 2H). ^{13}C NMR (151 MHz, DMSO) δ 174.38, 174.36, 170.44, 170.41, 148.18, 137.23, 136.57, 136.28, 134.90, 132.38, 132.36, 130.59, 130.56, 130.54, 127.02, 124.18, 94.04, 93.97, 58.01, 56.64, 56.29, 54.99, 54.79, 48.81, 48.29, 48.23, 46.69, 42.75, 41.15, 33.07, 33.03, 32.61, 32.48, 30.47, 19.82, 19.78. HRMS (ESI-TOF) m/z calculated for $C_{23}H_{25}IN_3O_7S$ $[M+H]^+$ 614.0452; found 614.0457.

(*S*)-5-(8-(4-iodophenyl)-2-((4-nitrophenyl)sulfonyl)-2,6-diazaspiro[3.4]octan-6-yl)-5-oxopentanoic acid (**22**) [prepared as for **21**]

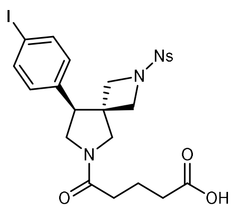

Colorless foam, mixture of rotamers. $[\alpha]^{20}_{D}$ = 3.00 (c=1, $CHCl_3$). ^1H NMR (600 MHz, DMSO) δ 7.97 – 7.89 (m, 2H), 7.78 – 7.70 (m, 2H), 7.54 (dd, *J* = 14.0, 7.9 Hz, 2H), 6.94 (dd, *J* = 27.3, 7.9 Hz, 2H), 3.97 – 3.85 (m, 2.65H), 3.82 – 3.56 (m, 5.35H), 3.51 (dd, *J* = 18.6, 11.4 Hz, 1H), 2.34 – 2.20 (m, 4H), 1.73 (dt, *J* = 11.3, 7.1 Hz, 2H). ^{13}C NMR (151 MHz, DMSO) δ 174.38, 174.36, 170.43, 170.41, 148.17, 139.23, 137.23, 136.56, 136.28, 134.90, 134.88, 132.38, 132.36, 130.59, 130.56, 130.54, 127.02, 124.97, 124.18, 94.04, 93.97, 58.01, 56.64, 56.29, 54.99, 54.79, 48.81, 48.29, 48.23, 46.69, 42.75, 41.15, 34.42, 33.07, 33.03, 32.61, 32.48, 30.47, 21.09, 19.81, 19.78. HRMS (ESI-TOF) m/z calculated for $C_{23}H_{25}IN_3O_7S$ $[M+H]^+$ 614.0452; found 614.0454.

(*R*)-5-(6-(((9H-fluoren-9-yl)methoxy)carbonyl)-8-(4-iodophenyl)-2,6-diazaspiro[3.4]octan-2-yl)-5-oxopentanoic acid (**23**)

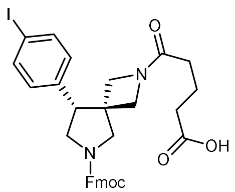

To a 12 mL vial was added **19** (60 mg, 0.1 mmol, 1 eq) and K_2CO_3 (28 mg, 0.2 mmol, 2 eq), dissolved with DMF (1 mL), then thiophenol (13 mg, 0.12 mmol, 1.2 eq) was added. The resulting solution was

stirred at rt for 30 min and then concentrated, the residue was then dissolved with CH_2Cl_2 (2 mL), and NEt_3 (50 mg, 0.5 mmol, 5 eq) and glutaric anhydride (35 mg, 0.3 mmol, 3 eq) were added sequentially, the resulting solution was stirred at rt overnight. The solution was washed with 2.0 M aq. HCl (10 mL) and extracted with CH_2Cl_2 (10 mL) 3 times, then the organic portions were combined and dried over anhydrous Na_2SO_4. The solution was concentrated and purified by preparative TLC chromatography to give (*R*)-5-(6-(*tert*-butoxycarbonyl)-8-(4-iodophenyl)-2,6-diazaspiro[3.4]octan-2-yl)-5-oxopentanoic acid (47 mg, 89 µmol, 88% yield). The above isolated material was dissolved in a 12 mL vial with CH_2Cl_2 (2 mL), then trifluoroacetic acid (0.5 mL) was added. The resulting solution was stirred at rt for 2 hr and then concentrated, the residue was dissolved with CH_2Cl_2 (2 mL), NEt_3 (100 mg, 1.0 mmol, 12 eq) and fluorenylmethyloxycarbonyl chloride (52 mg, 0.2 mmol, 2.2 eq) were added sequentially, the resulting solution was stirred at rt for 4 h. The solution was washed with 1.0 M aq. HCl (10 mL) and extracted with CH_2Cl_2 (10 mL) 3 times. The organic portions were combined and dried over anhydrous Na_2SO_4. The solution was concentrated and purified by preparative TLC chromatography to give **23** (48 mg, 74 µmol, 85% yield).

Colorless foam, mixture of rotamers. $[\alpha]^{20}_D$ = -4.20 (c=1, $CHCl_3$). ^{1}H NMR (600 MHz, $CDCl_3$) δ 7.77 (dd, *J* = 16.9, 7.5 Hz, 2H), 7.67 (q, *J* = 8.3 Hz, 2H), 7.59 (dd, *J* = 14.3, 7.4 Hz, 2H), 7.41 (dt, *J* = 13.2, 7.4 Hz, 2H), 7.36 – 7.29 (m, 2H), 6.92 – 6.83 (m, 2H), 4.58-4.46 (m, 2H), 4.25 (dt, *J* = 12.6, 6.6 Hz, 1H), 4.05 – 3.96 (m, 1H), 3.91 (d, *J* = 10.6 Hz, 1H), 3.87 – 3.50 (m, 6H), 3.33 – 3.24 (m, 1H), 2.45 – 2.32 (m, 2H), 2.15 (q, *J* = 8.2 Hz, 1H), 2.10 – 2.02 (m, 1H), 1.88 (dp, *J* = 39.2, 7.7, 7.2 Hz, 2H); ^{13}C NMR (151 MHz, $CDCl_3$) δ 177.10, 172.98, 154.77, 143.91, 141.49, 138.24, 129.79, 129.75, 127.98, 127.94, 127.28, 127.24, 125.07, 125.04, 120.21, 120.15, 93.48, 93.45, 67.45, 60.97, 58.56, 58.08, 54.89, 54.76, 54.68, 54.57, 54.39, 51.88, 50.50, 50.20, 49.99, 49.87, 49.50, 49.39, 47.50, 43.42, 42.65, 33.16, 30.33, 30.30, 19.92, 19.89, 19.87; HRMS (ESI-TOF) m/z calculated for $C_{32}H_{32}IN_2O_5$ $[M+H]^+$ 651.1350; found 651.1351.

(*S*)-5-(6-(((9H-fluoren-9-yl)methoxy)carbonyl)-8-(4-iodophenyl)-2,6-diazaspiro[3.4]octan-2-yl)-5-oxopentanoic acid (**24**)

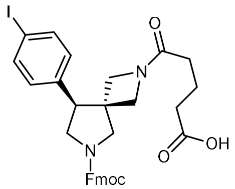

Colorless foam, mixture of rotamers. $[\alpha]^{20}_D$ = 4.10 (c=1, $CHCl_3$); ^{1}H NMR (600 MHz, $CDCl_3$) δ 7.77 (dd, *J* = 16.8, 7.5 Hz, 2H), 7.68 (q, *J* = 8.1 Hz, 2H), 7.59 (dd, *J* = 14.0, 7.3 Hz, 2H), 7.41 (dt, *J* = 13.4, 7.3 Hz, 2H), 7.37 – 7.29 (m, 2H), 6.94 – 6.81 (m, 2H), 4.60 – 4.45 (m, 2H), 4.26 (dt, *J* = 12.5, 6.4 Hz, 1H), 4.05 – 3.96 (m, 1H), 3.91 (d, *J* = 10.6 Hz, 1H), 3.87 – 3.49 (m, 6H), 3.29 (p, *J* = 11.0, 10.3 Hz, 1H), 2.44 – 2.33 (m, 2H), 2.15 (q, *J* = 8.7, 8.3 Hz, 1H), 2.10 – 2.03 (m, 1H), 1.95 – 1.82 (m, 2H); ^{13}C NMR (151 MHz, $CDCl_3$) δ 177.20, 172.95, 154.78, 144.01, 143.96, 143.90, 141.51, 138.31, 138.26, 129.80, 127.96,

127.94, 127.25, 125.05, 120.17, 93.46, 67.46, 60.99, 58.56, 54.40, 51.88, 50.54, 50.01, 49.89, 49.53, 49.42, 47.52, 43.41, 42.68, 33.20, 33.18, 33.12, 30.34, 30.31, 19.93, 19.90, 19.88; HRMS (ESI-TOF) m/z calculated for $C_{32}H_{32}IN_2O_5$ [M+H]$^+$ 651.1350; found 651.1354.

X-ray data for methyl (3*R*,4*S*)-4-(4-iodophenyl)-1-((4-nitrophenyl)sulfonyl)pyrrolidine-3-carboxylate (**25**)

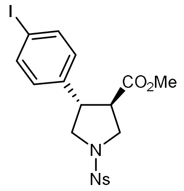

The single crystal X-ray diffraction studies were carried out on a Bruker ApexII-Ultra CCD diffractometer equipped with Mo K_α radiation (λ =0.7107 Å).

Crystals of the subject compound were used as received. A 0.22 x 0.02 x 0.01 mm piece of a colorless crystal was mounted on a Cryoloop with Paratone oil. Data were collected in a nitrogen gas stream at 100(2) K using ϖ scans. Crystal-to-detector distance was 40 mm and exposure time was 30 seconds depending on the 2θ range per frame using a scan width of 0.70°. Data collection was 99.9 % complete to 25.242° in θ. A total of 10995 reflections were collected covering the indices, -6<=h<=6, -14<=k<=14, -17<=l<=20. 4137 reflections were found to be symmetry independent, with a R_{int} of 0.0469. Indexing and unit cell refinement indicated a **P**rimitive, **Monoclinic** lattice. The space group was found to be ***P*2₁2₁2**. The data were integrated using the Bruker SAINT Software program and scaled using the SADABS software program. Solution by direct methods (SHELXT) produced a complete phasing model consistent with the proposed structure.

All nonhydrogen atoms were refined anisotropically by full-matrix least-squares (SHELXL-2014). All carbon bonded hydrogen atoms were placed using a riding model. Their positions were constrained relative to their parent atom using the appropriate HFIX command in SHELXL-2014. Crystallographic data are summarized in Supplementary Table 1.

Absolute stereochemistry was conclusively assigned (Flack = 0.14(12)). There is one copy of the molecule in the asymmetric unit. The chemical formula of the compound is: $C_{18}H_{17}IN_2O_6S$.

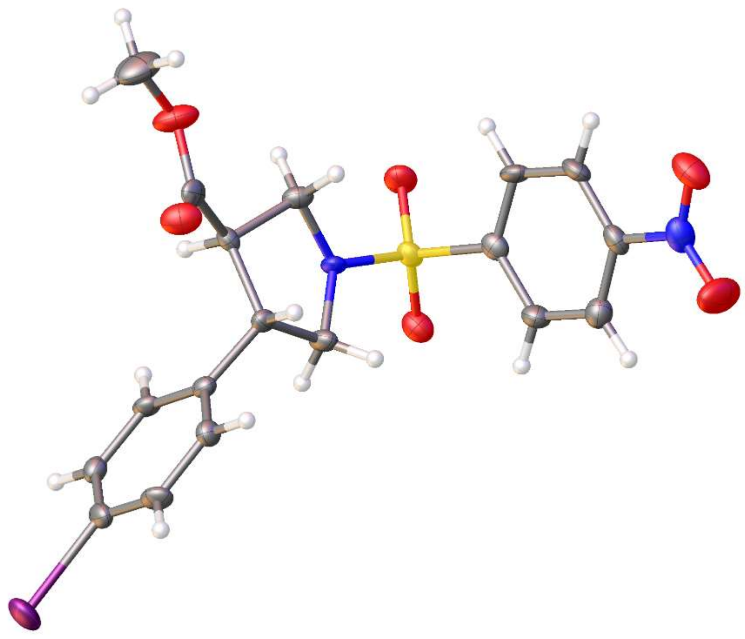

Supplementary Table 1 | Crystal data and structure refinement.

Empirical formula	C18 H17 I N2 O6 S	
Formula weight	516.29	
Temperature	100.0 K	
Wavelength	0.71073 Å	
Crystal system	Monoclinic	
Space group	P 1 21 1	
Unit cell dimensions	a = 5.2498(12) Å	a = 90°.
	b = 11.580(2) Å	b = 96.166(7)°.
	c = 16.099(3) Å	g = 90°.
Volume	973.0(4) Å^3	
Z	2	
Density (calculated)	1.762 Mg/m^3	
Absorption coefficient	1.791 mm-1	
F(000)	512	
Crystal size	0.22 x 0.02 x 0.01 mm^3	
Theta range for data collection	2.171 to 26.732°.	
Index ranges	-6<=h<=6, -14<=k<=14, -17<=l<=20	
Reflections collected	10995	
Independent reflections	4137 [R(int) = 0.0469]	

Completeness to theta = 25.242°	99.90%
Absorption correction	Semi-empirical from eq
Max. and min. transmission	0.4912 and 0.4352
Refinement method	Full-matrix least-squares on F^2
Data / restraints / parameters	4137 / 1 / 254
Goodness-of-fit on F^2	1.032
Final R indices [I>2sigma(I)]	R1 = 0.0278, wR2 = 0.0587
R indices (all data)	R1 = 0.0300, wR2 = 0.0594
Absolute structure parameter	0.014(12)
Largest diff. peak and hole	0.724 and -0.368 e.Å^{-3}

Supplementary Table 2 | Atomic coordinates (x 10^4) and equivalent isotropic displacement parameters (Å^2 x 10^3). U(eq) is defined as one third of the trace of the orthogonalized U^{ij} tensor.

	x	y	z	U(eq)
I(1)	5063(1)	1190(1)	9851(1)	24(1)
S(1)	-90(2)	6773(1)	5621(1)	13(1)
O(1)	8377(7)	7080(3)	2865(2)	24(1)
O(2)	6931(9)	5360(3)	2582(3)	33(1)
O(3)	-1969(6)	5874(3)	5517(2)	17(1)
O(4)	-877(6)	7955(3)	5679(2)	18(1)
O(5)	9161(6)	6565(3)	8201(2)	21(1)
O(6)	7049(7)	8244(3)	8180(3)	24(1)
N(1)	6919(7)	6277(5)	2962(2)	18(1)
N(2)	1753(7)	6485(3)	6459(2)	12(1)
C(1)	3287(9)	7598(4)	4582(3)	15(1)
C(2)	4954(9)	7464(4)	3980(3)	16(1)
C(3)	5074(8)	6409(4)	3589(2)	15(1)
C(4)	3569(9)	5474(4)	3755(3)	16(1)
C(5)	1912(9)	5607(4)	4371(3)	16(1)
C(6)	1816(9)	6667(4)	4777(3)	13(1)
C(7)	3609(9)	7330(4)	6855(3)	13(1)
C(8)	4775(8)	6643(4)	7617(3)	10(1)
C(9)	7245(9)	7113(4)	8024(3)	14(1)
C(10)	9311(10)	8793(5)	8610(4)	31(1)
C(11)	4995(9)	5403(4)	7280(3)	12(1)
C(12)	2671(10)	5291(4)	6608(3)	15(1)

C(13)	5104(8)	4443(4)	7917(3)	11(1)
C(14)	3265(9)	4360(4)	8479(3)	12(1)
C(15)	3284(9)	3448(4)	9035(3)	13(1)
C(16)	5140(9)	2597(4)	9030(3)	15(1)
C(17)	6986(9)	2656(4)	8484(3)	15(1)
C(18)	6965(9)	3591(4)	7931(3)	13(1)

Supplementary Table 3 | Bond lengths [Å] and angles [°].

I(1)-C(16)	2.100(4)	C(16)-C(17)	1.378(6)	C(9)-C(8)-C(7)	114.6(4)
S(1)-O(3)	1.432(3)	C(17)-H(17)	0.95	C(9)-C(8)-H(8)	108.2
S(1)-O(4)	1.435(3)	C(17)-C(18)	1.401(7)	C(9)-C(8)-C(11)	113.5(4)
S(1)-N(2)	1.609(4)	C(18)-H(18)	0.95	C(11)-C(8)-H(8)	108.2
S(1)-C(6)	1.776(5)	O(3)-S(1)-O(4)	120.1(2)	O(5)-C(9)-O(6)	123.6(4)
O(1)-N(1)	1.225(6)	O(3)-S(1)-N(2)	106.98(19)	O(5)-C(9)-C(8)	125.4(4)
O(2)-N(1)	1.227(6)	O(3)-S(1)-C(6)	107.4(2)	O(6)-C(9)-C(8)	111.0(4)
O(5)-C(9)	1.198(5)	O(4)-S(1)-N(2)	107.14(19)	O(6)-C(10)-H(10A)	109.5
O(6)-C(9)	1.339(6)	O(4)-S(1)-C(6)	107.8(2)	O(6)-C(10)-H(10B)	109.5
O(6)-C(10)	1.455(6)	N(2)-S(1)-C(6)	106.8(2)	O(6)-C(10)-H(10C)	109.5
N(1)-C(3)	1.479(5)	C(9)-O(6)-C(10)	116.2(4)	H(10A)-C(10)-H(10B)	109.5
N(2)-C(7)	1.476(6)	O(1)-N(1)-O(2)	123.8(4)	H(10A)-C(10)-H(10C)	109.5
N(2)-C(12)	1.476(6)	O(1)-N(1)-C(3)	118.2(4)	H(10B)-C(10)-H(10C)	109.5
C(1)-H(1)	0.95	O(2)-N(1)-C(3)	117.9(4)	C(8)-C(11)-H(11)	107.9
C(1)-C(2)	1.383(6)	C(7)-N(2)-S(1)	122.3(3)	C(8)-C(11)-C(12)	103.9(4)
C(1)-C(6)	1.381(6)	C(12)-N(2)-S(1)	119.2(3)	C(12)-C(11)-H(11)	107.9
C(2)-H(2)	0.95	C(12)-N(2)-C(7)	111.2(4)	C(13)-C(11)-C(8)	116.5(3)
C(2)-C(3)	1.380(6)	C(2)-C(1)-H(1)	120.6	C(13)-C(11)-H(11)	107.9
C(3)-C(4)	1.383(6)	C(6)-C(1)-H(1)	120.6	C(13)-C(11)-C(12)	112.5(4)
C(4)-H(4)	0.95	C(6)-C(1)-C(2)	118.8(4)	N(2)-C(12)-C(11)	104.7(4)
C(4)-C(5)	1.397(7)	C(1)-C(2)-H(2)	120.6	N(2)-C(12)-H(12A)	110.8
C(5)-H(5)	0.95	C(3)-C(2)-C(1)	118.9(4)	N(2)-C(12)-H(12B)	110.8
C(5)-C(6)	1.394(6)	C(3)-C(2)-H(2)	120.6	C(11)-C(12)-H(12A)	110.8
C(7)-H(7A)	0.99	C(2)-C(3)-N(1)	117.9(4)	C(11)-C(12)-H(12B)	110.8
C(7)-H(7B)	0.99	C(2)-C(3)-C(4)	123.4(4)	H(12A)-C(12)-H(12B)	108.9
C(7)-C(8)	1.532(6)	C(4)-C(3)-N(1)	118.7(4)	C(14)-C(13)-C(11)	120.9(4)
C(8)-H(8)	1	C(3)-C(4)-H(4)	121.2	C(18)-C(13)-C(11)	120.7(4)
C(8)-C(9)	1.492(6)	C(3)-C(4)-C(5)	117.6(4)	C(18)-C(13)-C(14)	118.4(4)

C(8)-C(11)	1.544(6)	C(5)-C(4)-H(4)	121.2	C(13)-C(14)-H(14)	119.5
C(10)-H(10A)	0.98	C(4)-C(5)-H(5)	120.4	C(15)-C(14)-C(13)	121.0(4)
C(10)-H(10B)	0.98	C(6)-C(5)-C(4)	119.1(4)	C(15)-C(14)-H(14)	119.5
C(10)-H(10C)	0.98	C(6)-C(5)-H(5)	120.4	C(14)-C(15)-H(15)	120.2
C(11)-H(11)	1	C(1)-C(6)-S(1)	119.6(4)	C(14)-C(15)-C(16)	119.7(4)
C(11)-C(12)	1.547(6)	C(1)-C(6)-C(5)	122.1(4)	C(16)-C(15)-H(15)	120.2
C(11)-C(13)	1.508(6)	C(5)-C(6)-S(1)	118.1(3)	C(15)-C(16)-I(1)	119.1(3)
C(12)-H(12A)	0.99	N(2)-C(7)-H(7A)	111.6	C(17)-C(16)-I(1)	120.1(3)
C(12)-H(12B)	0.99	N(2)-C(7)-H(7B)	111.6	C(17)-C(16)-C(15)	120.7(4)
C(13)-C(14)	1.396(6)	N(2)-C(7)-C(8)	101.0(4)	C(16)-C(17)-H(17)	120.5
C(13)-C(18)	1.388(6)	H(7A)-C(7)-H(7B)	109.4	C(16)-C(17)-C(18)	119.1(4)
C(14)-H(14)	0.95	C(8)-C(7)-H(7A)	111.6	C(18)-C(17)-H(17)	120.5
C(14)-C(15)	1.384(6)	C(8)-C(7)-H(7B)	111.6	C(13)-C(18)-C(17)	121.2(4)
C(15)-H(15)	0.95	C(7)-C(8)-H(8)	108.2	C(13)-C(18)-H(18)	119.4
C(15)-C(16)	1.386(6)	C(7)-C(8)-C(11)	103.9(4)	C(17)-C(18)-H(18)	119.4

Supplementary Table 4 | Anisotropic displacement parameters (Å^2 x 10^3). The anisotropic displacement factor exponent takes the form: 2π2 [h^2 **a***^{2} U^{11} + ... + 2 h k **a*** **b*** U^{12}].

	U^{11}	U^{22}	U^{33}	U^{23}	U^{13}	U^{12}
I(1)	39(1)	16(1)	18(1)	9(1)	6(1)	4(1)
S(1)	15(1)	11(1)	12(1)	2(1)	2(1)	2(1)
O(1)	20(2)	25(2)	25(2)	10(2)	4(2)	-2(2)
O(2)	46(3)	23(2)	35(2)	-10(2)	22(2)	-4(2)
O(3)	16(2)	18(2)	18(2)	4(1)	2(1)	0(1)
O(4)	23(2)	12(2)	19(2)	1(1)	4(1)	6(1)
O(5)	15(2)	17(2)	30(2)	-1(1)	1(1)	2(1)
O(6)	22(2)	12(2)	37(2)	-7(2)	2(2)	-1(2)
N(1)	19(2)	20(2)	16(2)	3(3)	4(1)	7(2)
N(2)	19(2)	5(2)	12(2)	0(1)	1(1)	1(1)
C(1)	22(2)	6(2)	16(2)	0(2)	0(2)	-1(2)
C(2)	23(3)	11(2)	14(2)	5(2)	2(2)	-4(2)
C(3)	18(2)	16(3)	12(2)	5(2)	3(2)	1(2)
C(4)	19(3)	17(2)	13(2)	0(2)	2(2)	1(2)
C(5)	21(3)	11(2)	15(2)	2(2)	2(2)	-3(2)
C(6)	12(2)	13(2)	13(2)	3(2)	-1(2)	2(2)
C(7)	13(2)	9(2)	17(3)	-1(2)	2(2)	0(2)

C(8)	11(2)	11(2)	10(2)	3(2)	2(2)	1(2)
C(9)	18(2)	13(2)	12(2)	-2(2)	8(2)	1(2)
C(10)	30(3)	23(3)	39(3)	-12(3)	6(2)	-11(3)
C(11)	15(2)	9(2)	11(2)	-1(2)	4(2)	1(2)
C(12)	21(3)	8(2)	14(2)	2(2)	-3(2)	0(2)
C(13)	13(2)	8(2)	10(2)	-1(2)	-1(2)	0(2)
C(14)	16(2)	7(2)	14(2)	-1(2)	-1(2)	2(2)
C(15)	15(2)	14(3)	10(2)	-2(2)	2(2)	-2(2)
C(16)	23(3)	12(2)	10(2)	2(2)	-3(2)	-2(2)
C(17)	18(2)	7(2)	18(2)	1(2)	-2(2)	4(2)
C(18)	16(2)	13(3)	11(3)	0(2)	4(2)	-1(2)

Supplementary Table 5 | Hydrogen coordinates (x 10^4) and isotropic displacement parameters ($Å^2$x 10^3).

	x	y	z	U(eq)
H(1)	3156	8318	4857	18
H(2)	5999	8088	3838	20
H(4)	3659	4767	3460	20
H(5)	864	4984	4512	19
H(7A)	2746	8039	7023	16
H(7B)	4919	7537	6482	16
H(8)	3513	6637	8040	13
H(10A)	10695	8787	8247	46
H(10B)	9857	8369	9125	46
H(10C)	8904	9592	8746	46
H(11)	6587	5360	6992	14
H(12A)	3192	4947	6089	18
H(12B)	1319	4804	6812	18
H(14)	1980	4938	8479	15
H(15)	2032	3405	9418	15
H(17)	8255	2071	8483	18
H(18)	8248	3642	7559	16

1.4. Enumerated DEL targets synthesized off-DNA

The following targets in this section were synthesized by multiple chemists and did not follow consistent protocols. A full synthetic protocol is given for each target compound to provide added clarity.

2-((3'-((2R,3R)-1-(2-(allylamino)thiazole-5-carbonyl)-2-(methylcarbamoyl)pyrrolidin-3-yl)-5-fluoro-[1,1'-biphenyl]-3-yl)oxy)acetic acid (**3**)

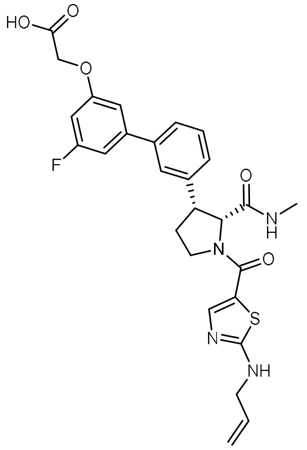

To a vial charged with **6c** (30 mg, 1 eq, 72 µmol), NH_3MeCl (9.7 mg, 2.0 eq) and HATU (32.9 mg, 1.2 eq) were added. The vial was purged with N_2, before addition of anhydrous DMF (750 µL) and DIPEA (63 µL, 5 eq). The reaction was stirred overnight at rt, diluted with ethyl acetate and washed twice with water, then brine, before being dried over anhydrous $MgSO_4$, filtered, and removed solvent under reduced pressure. The crude *tert*-butyl (2*R*,3*R*)-3-(3-iodophenyl)-2-(methylcarbamoyl)pyrrolidine-1-carboxylate (29 mg, 68 µmol, 94%) was used directly in the next step without additional purification.

A vial was charged with *tert*-butyl (2*R*,3*R*)-3-(3-iodophenyl)-2-(methylcarbamoyl)pyrrolidine-1-carboxylate (27 mg, 63 µmol, 1 eq), 2-(3-borono-5-fluorophenoxy)acetic acid (16 mg, 75 µmol, 1.2 eq), $Pd(PPh_3)_4$ (7.3 mg, 6.3 µmol, 0.1 eq), and Na_2CO_3 (27 mg, 250 µmol, 4.0 eq). The vial was sealed and purged with N_2 before addition of water (0.5 mL), DMF (0.5 mL), and MeCN (0.5 mL) were added, and the reaction was stirred 80 °C for 1 hr under microwave irradiation. The crude solution was purified by preparative HPLC to afford 2-((3'-((2*R*,3*R*)-1-(*tert*-butoxycarbonyl)-2-(methylcarbamoyl)pyrrolidin-3-yl)-5-fluoro-[1,1'-biphenyl]-3-yl)oxy)acetic acid (5.9 mg, 12.6 µmol, 20%).

2-(Allylamino)thiazole-5-carboxylic acid (30 mg, 0.16 mmol, 1 eq), NHS (21 mg, 0.18 mmol, 1.1 eq) and EDC (34 mg, 0.18 mmol, 1.1 eq) was dissolved in THF and stirred at rt for 6 hr. The resulting solution was purified by flash column chromatography (0–100% EtOAC in hexanes) to afford 2,5-dioxopyrrolidin-1-yl 2-(allylamino)thiazole-5-carboxylate (29 mg, 0.10 mmol, 65%).

2-((3'-((2*R*,3*R*)-1-(*tert*-Butoxycarbonyl)-2-(methylcarbamoyl)pyrrolidin-3-yl)-5-fluoro-[1,1'-biphenyl]-3-yl)oxy)acetic acid (5 mg, 11 µmol, 1 eq) was dissolved in CH_2Cl_2 (1 mL), followed by addition of TFA (0.2

mL). The mixture was stirred at rt for 2 hr before removal of solvents under reduced pressure, then co-evaporated with toluene. To the residue was added 2,5-dioxopyrrolidin-1-yl 2-(allylamino)thiazole-5-carboxylate (4.46 mg, 15.9 μmol, 1.5 eq) before being dissolved in DMF (250 μL), followed by addition of DIPEA (5 μL, 3 eq). The mixture was stirred overnight, then was purified by preparative HPLC to afford **3** (1.5 mg, 2.75 μmol, 25%).

^{1}H NMR (600 MHz, DMSO) δ 8.39 – 8.19 (m, 1.5H), 7.70 – 7.50 (m, 3.5H), 7.37 (t, *J* = 7.8 Hz, 1H), 7.23 (d, *J* = 8.0 Hz, 1H), 7.05 (d, *J* = 9.9 Hz, 1H), 7.01 (s, 1H), 6.71 (dd, *J* = 10.7, 2.7 Hz, 1H), 5.96 – 5.71 (m, 0.5H), 5.24 (d, *J* = 19.4 Hz, 0.5H), 5.13 (d, *J* = 11.6 Hz, 0.5H), 4.05 (t, *J* = 10.0 Hz, 0.6H), 4.00 – 3.76 (m, 3H), 3.76 – 3.58 (m, 0.6H), 2.79 – 2.63 (m, 0.5H), 2.33 – 2.07 (m, 3.5H). LRMS (ESI): calculated for $C_{27}H_{28}FN_4O_5S$ [M + H]$^+$, 539.18; found, 539.36.

(2*S*,4*S*)-4-(3-((*E*)-2-cyclohexylvinyl)phenoxy)-1-(3-ethynylbenzyl)-*N*-methylpyrrolidine-2-carboxamide (**27**)

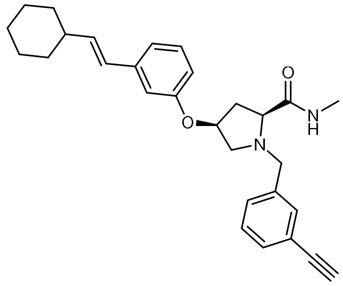

To a solution of (2*S*,4*S*)-4-(3-iodophenoxy)-2-(methoxycarbonyl)pyrrolidin-1-ium 2,2,2-trifluoroacetate (50 mg, 1 eq, 0.11 mmol) di-*tert*-butyl dicarbonate (28 mg, 1.2 eq, 0.13 mmol) in CH_2Cl_2 (1.0 mL) was added NEt_3 (13 mg, 18 μL, 1.2 eq, 0.13 mmol). The resulting mixture was stirred at rt for 16 h. The reaction was then concentrated, and the crude residue purified by column chromatography on silica gel (12 g, 0 to 70% EtOAc in Hexanes), affording the Boc-protected amine, **9aii**. To **9aii** (50 mg, 1 eq, 0.11 mmol) were added THF (3 mL) and LiOH (40 mg, 1.7 mL, 1 M, 15 eq, 1.7 mmol). The resulting reaction mixture was vigorously stirred for 16 h, then diluted with H_2O (15 mL). The aqueous layer was acidified with 1N HCl to pH of ~2 and extracted with CH_2Cl_2 (3 x 20 mL). The combined organics were then dried over Na_2SO_4 and concentrated under reduced pressure to afford the crude acid. This material was dissolved in CH_2Cl_2 (2 mL) and treated with HATU (58 mg, 1.2 eq, 0.15 mmol) and DIPEA (49 mg, 66 μL, 3.0 eq, 0.38 mmol). The resulting reaction mixture was stirred for 15 min at rt and methylamine hydrochloride (13 mg, 1.5 eq, 0.19 mmol) was added. The reaction was stirred for 16 hr at rt. The CH_2Cl_2 was removed under reduced pressure and the crude residue was diluted in EtOAc (25 mL). The organics were washed with water (1 x 25 mL), 10% aq. NaOH (1 x 25 mL) and brine (1 x 25 mL), dried over Na_2SO_4, and concentrated under reduced pressure to afford the cure amide, which was purified by column chromatography on silica gel (4 g, 0 to 80% EtOAc in hexanes). The resulting material was then charged to a 5 mL μW vial equipped with a triangular magnetic stir bar. (*E*)-(2-cyclohexylvinyl)boronic acid (23 mg, 1.2 eq, 0.15 mmol), Pd-Xphos-G2 (19 mg, 0.20 eq, 25 μmol), and DMF (0.93 mL) were added, followed by aq. K_3PO_4 (0.10 g, 0.27 mL,

1.8 molar, 4 eq, 0.49 mmol). The resulting mixture was sparged with argon for 3 min, then the vial was sealed and heated to 60 °C for 1 hour in a µW reactor. The reaction mixture was diluted with EtOAc (40 mL) and washed with H_2O (1 x 15 mL) then brine (2 x 15 mL). The organics were then dried over Na_2SO_4 and concentrated under reduced pressure. The crude residues were purified by column chromatography on silica gel (4 g, 0% to 90% EtOAc in hexanes) to afford the crude product as a colorless oil which was immediately dissolved in methanolic HCl (1.0 mL, 2 M, 2.0 mmol, 29 eq.) and incubated for 3 h, after which time volatiles were removed under reduced pressure to afford (2*S*,4*S*)-4-(3-((*E*)-2-cyclohexylvinyl)phenoxy)-*N*-methylpyrrolidine-2-carboxamide hydrochloride (26 mg, 70 µmol, 52% over five steps).

The above hydrochloride salt (26 mg, 1 eq., 71 µmol) was taken up in DCE (0.5 mL) and treated with 3-ethynylbenzaldehyde (11 mg, 1.2 eq, 86 µmol) and sodium triacetoxyhydroborate (23 mg, 1.5 eq, 0.11 mmol). The resulting mixture was stirred for 1 hr at rt, then concentrated under reduced pressure, re-dissolved in CH_2Cl_2 (25 mL), and washed with water (15 mL). The aqueous layer was then back-extracted with CH_2Cl_2 (2 x 25 mL) and the combined organics dried over Na_2SO_4 and concentrated under reduced pressure. The crude residue was purified by preparative HPLC to afford **27** (15 mg, 33 µmol, 46% over two steps) as a white solid.

^{1}H NMR (400 MHz, CD_3OD) δ 7.53 – 7.49 (m, 1H), 7.44 – 7.29 (m, 3H), 7.16 (t, *J* = 7.9 Hz, 1H), 6.95 – 6.90 (m, 1H), 6.83 (t, *J* = 2.0 Hz, 1H), 6.68 (dd, *J* = 8.1, 2.3 Hz, 1H), 6.31 (d, *J* = 15.9 Hz, 1H), 6.17 (dd, *J* = 16.0, 6.9 Hz, 1H), 4.92 – 4.87 (m, 1H), 3.82 (d, *J* = 12.9 Hz, 1H), 3.66 (d, *J* = 12.8 Hz, 1H), 3.49 (s, 1H), 3.32 – 3.24 (m, 2H), 2.77 (dd, *J* = 10.8, 4.4 Hz, 1H), 2.71 (s, 3H), 2.62 (ddd, *J* = 14.2, 10.3, 5.6 Hz, 1H), 2.18 – 2.02 (m, 2H), 1.87 – 1.65 (m, 5H), 1.44 – 1.14 (m, 5H). ^{13}C NMR (151 MHz, DMSO) δ 173.11, 173.03, 157.82, 139.48, 139.23, 137.11, 132.57, 130.97, 130.15, 130.00, 129.00, 127.40, 122.09, 118.87, 114.34, 113.28, 84.03, 81.18, 75.58, 66.96, 58.89, 58.59, 40.89, 37.13, 32.86, 26.14, 26.03, 25.94, 25.90. HRMS (ESI): calculated for $C_{29}H_{35}N_2O_2$ $[M + H]^+$, 443.2693; found, 443.2689.

(2*R*,4*S*)-4-(3-((*E*)-2-cyclohexylvinyl)phenoxy)-1-(3-ethynylbenzyl)-*N*-methylpyrrolidine-2-carboxamide (**28**)

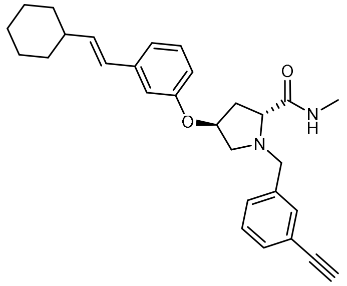

To a 1-dram vial containing (2*R*,4*S*)-1-(*tert*-butoxycarbonyl)-4-(3-iodophenoxy)pyrrolidine-2-carboxylic acid (17 mg, 39 µmol, 1 eq), methylamine hydrochloride (3.2 mg, 47 µmol, 1.2 eq) and HATU (18 mg, 47 µmol, 1.2 eq) were added. Additional CH_2Cl_2 (0.4 mL) was added, followed by DIPEA (34 µL, 200 µmol, 5

eq). The resulting yellow solution was stirred overnight, then diluted with CH_2Cl_2 and washed with water (3 x 1 mL) and brine (1 x 1 mL). The organic phase was dried over anhydrous $MgSO_4$, then concentrated under reduced pressure. The combined crude material from several reactions was pooled to provide crude acid (61 mg, 0.14 mmol), which was charged to a 2 mL μW vial. (*E*)-(2-cyclohexylvinyl)boronic acid (32 mg, 0.21 mmol, 1.5 eq) and XPhos-Pd-G2 (22 mg, 28 μmol, 0.2 eq) were added; the vial was then capped with a septum and purged with N_2 (3 times). Anhydrous DMF (2 mL) was added, followed by aq. K_3PO_4 (47 mL of a 1.8 M solution, 6.00 eq). The reaction was then heated to 60 °C for 50 mins in a μW reactor. The resulting dark brown suspension was transferred to a scintillation vial and concentrated under reduced pressure, then diluted with EtOAc and washed with water and brine. The organics were dried over $MgSO_4$, then concentrated under reduced pressure to provide crude material which was purified by flash column chromatography on silica gel (ethyl acetate/hexanes), then dissolved in CH_2Cl_2 (2 mL) and treated with TFA (0.4 mL), stirring at r.t. for 1.5 hr at which time LCMS showed complete consumption of starting material. Concentration under reduced pressure followed by azeotropic removal of residual TFA with toluene gave the free amine (20 mg, 4% over three steps). Half of this material (10 mgs, 0.03 mmol, 1.00 eq) was charged to a 1-dram vial containing 3-ethynylbenzaldehyde (5 mg, 0.04 mmol, 1.2 eq) and sodium acetoxyborohydride (10 mg, 0.06 mmol, 2.0 eq). The vial was purged with N_2, then charged with DCE (0.2 mL) and NEt_3 (4 μL, 0.03 mmol, 1.0 eq), yielding a yellow suspension which was stirred overnight. The reaction solution was then concentrated under reduced pressure, dissolved in DMSO and purified by preparative HPLC, affording **28** as a white solid (9 mg, 70%).

^{1}H NMR (400 MHz, $CDCl_3$) δ 7.46 – 7.38 (m, 2H), 7.30 (t, *J* = 7.5 Hz, 1H), 7.20 – 7.10 (m, 2H), 6.94 (d, *J* = 7.6 Hz, 1H), 6.80 (t, *J* = 2.0 Hz, 1H), 6.64 (dd, *J* = 8.1, 2.5 Hz, 1H), 6.28 (d, *J* = 16.0 Hz, 1H), 6.14 (dd, *J* = 16.0, 6.8 Hz, 1H), 4.84 – 4.76 (m, 1H), 3.92 (d, *J* = 13.4 Hz, 1H), 3.70 – 3.55 (m, 2H), 3.41 (dd, *J* = 12.0, 4.9 Hz, 1H), 3.09 (s, 1H), 2.81 (d, *J* = 5.0 Hz, 3H), 2.78 – 2.69 (m, 1H), 2.59 – 2.48 (m, 1H), 2.23 – 2.04 (m, 2H), 1.85 – 1.72 (m, 4H), 1.72 – 1.62 (m, 1H), 1.30 (tt, *J* = 12.1, 3.1 Hz, 2H), 1.24 – 1.11 (m, 3H). ^{13}C NMR (101 MHz, $CDCl_3$) δ 173.8, 157.9, 139.8, 138.7, 132.1, 131.2, 129.5, 129.1, 128.6, 126.9, 122.4, 119.1, 113.6, 113.1, 83.4, 77.5, 77.2, 76.1, 67.0, 60.1, 58.9, 41.1, 37.6, 32.9, 26.2, 26.0, 25.8. HRMS (ESI): calculated for $C_{29}H_{35}N_2O_2$ $[M + H]^+$, 443.2693; found, 443.2697.

(2*S*,4*S*)-4-(4-((*E*)-2-cyclohexylvinyl)phenoxy)-1-(3-cyclopropyl-1-ethyl-1H-pyrazole-5-carbonyl)-*N*-methylpyrrolidine-2-carboxamide (**29**)

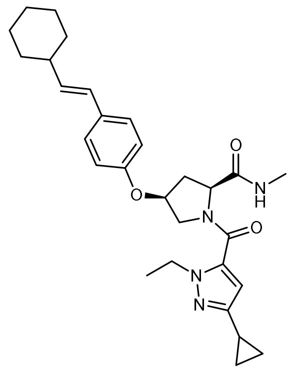

To a solution of (2*S*,4*S*)-4-(4-iodophenoxy)-2-(methoxycarbonyl)pyrrolidin-1-ium 2,2,2-trifluoroacetate (55 mg, 1 eq, 0.12 mmol) and di-*tert*-butyl dicarbonate (31 mg, 1.2 eq, 0.14 mmol) in CH_2Cl_2 (1.0 mL) was added NEt_3 (0.025 mL, 0.17 mmol). The resulting mixture was stirred at rt for 16 h, then concentrated under reduced pressure. The crude residue was purified by column chromatography on silica gel (12 g, 0 to 70% EtOAc in hexanes) to afford the desired product as a clear oil which was immediately dissolved in THF (3 mL) and treated with aq. LiOH (40 mg, 1.7 mL of a 1 M solution, 15 eq, 1.7 mmol). The resulting mixture was vigorously stirred 16 h, then diluted with water (15 mL) and acidified with 1 N aq. HCl to pH ~2. The aqueous mixture was extracted with CH_2Cl_2 (3 x 20 mL); organics were then combined dried over Na_2SO_4 and concentrated under reduced pressure to reveal the crude acid. This material was immediately dissolved in CH_2Cl_2 (2 mL) and treated with HATU (58 mg, 1.2 eq, 0.15 mmol) and DIPEA (49 mg, 66 µL, 3.0 eq, 0.38 mmol). The resulting mixture was stirred 15 min at rt, then methylamine hydrochloride (13 mg, 1.5 eq, 0.19 mmol) was added and the reaction stirred an additional 3 hr at rt. Volatiles were removed under reduced pressure and the crude residue diluted with EtOAc (25 mL), washed with water (25 mL), 10% aq. NaOH (25 mL), and brine (25 mL), then dried over Na_2SO_4. Volatiles were evaporated under reduced pressure and the residue purified by column chromatography on silica gel (4 g, EtOAc in hexanes, 0% to 80% EtOAc) to the amide. This material was then charged to a 5 mL µW vial equipped with a magnetic stir bar, followed by (*E*)-(2-cyclohexylvinyl)boronic acid (24 mg, 1.2 eq, 0.15 mmol), Palladium-Xphos (20 mg, 0.20 eq, 26 µmol), DMF (0.93 mL) and aq. K_3PO_4 (0.11 g, 0.29 mL of a 1.8 M solution, 4 eq, 0.51 mmol). The resulting mixture was sparged with argon for 3 min, then the vial was sealed, and the reaction mixture heated to 60 °C for 1 hr in a µW reactor. The mixture was diluted with EtOAc (40 mL), washed with H_2O (1 x 15 mL) and brine (2 x 15 mL), then dried over Na_2SO_4. Volatiles were removed under reduced pressure and the crude residue purified by column chromatography on silica gel (EtOAc in hexanes gradient, 0% to 90%) to afford *tert*-butyl (2*S*,4*S*)-4-(4-((*E*)-2-cyclohexylvinyl)phenoxy)-2-(methylcarbamoyl)pyrrolidine-1-carboxylate, which was immediately dissolved in methanolic HCl (1.0 mL of a 2 M solution, 29 eq, 2.0 mmol) and incubated for 3 h, at which point volatiles were removed under reduced pressure to give (2*S*,4*S*)-4-(4-((*E*)-2-cyclohexylvinyl)phenoxy)-*N*-methylpyrrolidine-2-carboxamide hydrochloride (44 mg, 0.06 mmol, 50% over five steps).

A portion (4.7 mg, 1.2 eq, 26 μmol) of the above hydrochloride salt was treated with 3-cyclopropyl-1-ethyl-1H-pyrazole-5-carboxylic acid (4.7 mg, 1.2 eq, 26 μmol), HATU (10 mg, 1.2 eq, 26 μmol) and DIPEA (7.1 mg, 9.5 μL, 2.5 eq, 55 μmol) which had all been pre-stirred for 15 min in CH_2Cl_2 (3 mL). The resulting solution was stirred for 4 hr at rt, then diluted with CH_2Cl_2 (25 mL) and washed with water (1 x 25 mL), sat. aq. $NaHCO_3$ (1 x 25 mL) and brine (1 x 25 mL). The combined organics were dried over Na_2SO_4 and concentrated under reduced pressure, affording crude material which was purified by HPLC to afford **29** (8.8 mg, 18 μmol, 69%) as a white solid.

^{1}H NMR (400 MHz, CD_3OD) (~3:2 mixture of rotamers) δ 7.28 (d, *J* = 8.3 Hz, 2H), 6.82 (d, *J* = 8.4 Hz, 2H), 6.41 (s, 0.6H), 6.30 (d, *J* = 15.9 Hz, 1H), 6.07 (app.dd, *J* = 15.9, 7.0 Hz, 1.4H), 5.11 – 5.01 (m, 1H), 4.74 (dd, *J* = 9.4, 3.7 Hz, 0.6H), 4.58 (d, *J* = 8.8 Hz, 0.4H), 4.27 (dh, *J* = 27.8, 7.1 Hz, 2H), 4.10 (ddd, *J* = 29.6, 12.6, 5.2 Hz, 1H), 3.90 (dt, *J* = 11.7, 5.0 Hz, 1H), 2.79 (s, 2H), 2.72 (s, 1H), 2.58 (ddd, *J* = 14.0, 9.2, 5.1 Hz, 1H), 2.47 (s, 0.4H), 2.36 (dd, *J* = 10.4, 6.9 Hz, 0.6H), 2.12 (dtt, *J* = 10.8, 7.2, 3.4 Hz, 1H), 1.92 (dtt, *J* = 11.7, 8.3, 4.3 Hz, 1H), 1.77 (d, *J* = 3.1 Hz, 4H), 1.75 – 1.67 (m, 1H), 1.45 – 1.13 (m, 8H), 0.94 (dt, *J* = 9.5, 3.1 Hz, 2H), 0.76 – 0.62 (m, 2H). ^{13}C NMR (151 MHz, DMSO) δ 171.61, 171.52, 171.25, 171.17, 161.69, 160.78, 156.23, 156.04, 152.51, 152.49, 136.25, 135.62, 134.78, 131.08, 131.03, 127.52, 127.49, 126.90, 126.87, 116.18, 116.00, 104.36, 102.35, 100.00, 75.34, 74.15, 61.29, 59.49, 59.45, 54.27, 52.59, 45.91, 45.44, 40.89, 40.54, 36.99, 34.80, 33.06, 26.30, 26.22, 26.16, 26.09, 25.99, 16.27, 16.03, 9.36, 9.31, 8.39, 8.33. MS (ESI): calculated for $C_{29}H_{39}N_4O_3$ $[M + H]^+$ 491.30; found, 491.42.

(2*S*,4*R*)-4-(4-((*E*)-2-cyclohexylvinyl)phenoxy)-1-(3-cyclopropyl-1-ethyl-1H-pyrazole-5-carbonyl)-*N*-methylpyrrolidine-2-carboxamide (**30**)

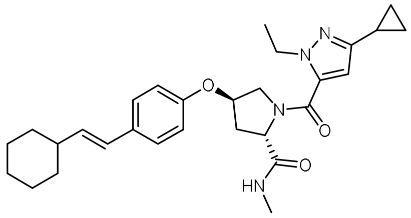

To a suspension of (2*S*,4*R*)-1-(*tert*-butoxycarbonyl)-4-(4-iodophenoxy)pyrrolidine-2-carboxylic acid (25 mg, 1 eq, 58 μmol) in CH_2Cl_2 (3.0 mL), was added DIPEA (22 mg, 30 μL, 3.0 eq, 0.17 mmol). The reaction was stirred for 15 min at rt before addition of methanamine hydrochloride (5.8 mg, 1.5 eq, 87 μmol) and continued stirring for 16 hr at rt. The volatiles were removed under reduced pressure and the crude residue was diluted in EtOAc (25 mL). The organic layer was washed with water (1 x 25 mL), 10% aq. NaOH (1 x 25 mL) and brine (1 x 25 mL), dried over Na_2SO_4 and concentrated under reduced pressure. The crude residue was purified by column chromatography (EtOAc/Hexanes gradient from 0% to 90% EtOAc, 4 g column) to afford the intermediate material *tert*-butyl (2*S*,4*R*)-4-(4-iodophenoxy)-2-(methylcarbamoyl)pyrrolidine-1-carboxylate which was then charged to a 5 mL μW vial equipped with a triangular magnetic stir bar. To this vial was charged (*E*)-(2-cyclohexylvinyl)boronic acid, Palladium-

Xphos (9.5 mg, 0.20 eq, 12 µmol), DMF (0.5 mL) and 1.8 M aq. K_3PO_4 (77 mg, 0.20 mL, 6.0 eq, 0.36 mmol). The mixture was purged with Ar for 3 min. The vial was then sealed and heated to 60 °C for 1 hr using a µW reactor. The reaction mixture was diluted with EtOAc (40 mL) and washed with H_2O (1 x 15mL) and brine (2 x 15 mL) before being dried over Na_2SO_4 and concentrated under reduced pressure. The crude residue was purified by column chromatography (EtOAc/Hexanes; 0% to 90% EtOAc, 4 g column) to afford *tert*-butyl (2*S*,4*R*)-4-(4-((*E*)-2-cyclohexylvinyl)phenoxy)-2-(methylcarbamoyl)pyrrolidine-1-carboxylate as a clear oil (20 mg, 47 µmol, 81% over 2 steps).

tert-butyl (2*S*,4*R*)-4-(4-((*E*)-2-cyclohexylvinyl)phenoxy)-2-(methylcarbamoyl)pyrrolidine-1-carboxylate (20 mg, 1 eq, 47 µmol) was dissolved in HCl (1.5 g, 1.0 mL, 2 M solution in MeOH, 43 eq, 2.0 mmol). The reaction was allowed to stand for 3 hr then volatiles were removed under reduced pressure to afford (2*S*,4*R*)-4-(4-((*E*)-2-cyclohexylvinyl)phenoxy)-*N*-methylpyrrolidine-2-carboxamide hydrochloride (quant.).

3-Cyclopropyl-1-ethyl-1H-pyrazole-5-carboxylic acid (10 mg, 1.2 eq, 56 µmol), and 2-(3H-[1,2,3]triazolo[4,5-*b*]pyridin-3-yl)-1,1,3,3-tetramethylisouronium hexafluorophosphate(V) (21 mg, 1.2 eq, 56 µmol) were dissolved in CH_2Cl_2 (1.0 mL) and DIPEA (15 mg, 20 µL, 2.5 eq, 0.12 mmol). The reaction was stirred for 15 min at rt before addition of (2*S*,4*R*)-4-(4-((*E*)-2-cyclohexylvinyl)phenoxy)-*N*-methylpyrrolidine-2-carboxamide hydrochloride (17 mg, 1 eq, 47 µmol) in CH_2Cl_2 (1.0 mL). The reaction was stirred for 16 hr at rt. The volatiles were removed under reduced pressure and the crude residue was diluted in EtOAc (25 mL). The organic layer was washed with water (1 x 25 mL), sat. aq. $NaHCO_3$ (1 x 25 mL) and brine (1 x 25 mL), dried over Na_2SO_4 and concentrated under reduced pressure. The crude residue was purified by preparative HPLC to afford **30** (16 mg, 33 µmol, 70%).

^{1}H NMR (400 MHz, CD_3OD) (~3:1 mixture of rotamers) δ 7.34 – 7.23 (m, 2H), 6.89 (d, *J* = 8.6 Hz, 0.5H), 6.85 – 6.78 (m, 1.5H), 6.30 (app.dd, *J* = 15.8, 8.0 Hz, 1H), 6.19 (s, 0.75H), 6.14 – 6.01 (m, 1.25H), 5.09 (s, 0.25H), 5.07 – 5.02 (m, 0.75 H), 4.70 (dd, *J* = 9.4, 7.7 Hz, 0.75H), 4.60 (t, *J* = 8.2 Hz, 0.25H), 4.34 – 4.11 (m, 2H), 4.09 – 4.02 (m, 0.25H), 3.96 – 3.81 (m, 1.75H), 2.80 (s, 2.25H), 2.65 – 2.56 (m, 1H), 2.55 (s, 0.75H), 2.25 (ddt, *J* = 13.6, 8.4, 4.2 Hz, 1H), 2.11 (dtd, *J* = 10.8, 7.2, 3.3 Hz, 1H), 1.89 (tt, *J* = 8.4, 5.0 Hz, 1H), 1.84 – 1.75 (m, 4H), 1.75 – 1.66 (m, 1H), 1.46 – 1.13 (m, 8H), 0.92 (td, *J* = 8.4, 2.5 Hz, 2H), 0.67 (dtt, *J* = 7.6, 5.2, 2.5 Hz, 2H). ^{13}C NMR (101 MHz, CD_3OD) δ 172.84, 172.39, 162.48, 161.76, 155.76, 155.48, 153.95, 135.84, 135.49, 134.69, 134.61, 131.94, 131.85, 126.88, 126.49, 126.44, 115.46, 115.40, 103.39, 101.81, 75.51, 74.14, 60.61, 59.00, 54.96, 52.72, 45.25, 45.12, 41.13, 37.29, 35.04, 32.86, 25.89, 25.77, 25.03, 24.87, 14.87, 14.42, 8.24, 7.02, 7.00, 6.94. HRMS (ESI): calculated for $C_{29}H_{39}N_3O_3$ $[M + H]^+$, 491.3017; found, 491.3026.

(2*R*,4*R*)-4-(4-((*E*)-2-Cyclohexylvinyl)phenoxy)-1-(3-cyclopropyl-1-ethyl-1H-pyrazole-5-carbonyl)-*N*-methylpyrrolidine-2-carboxamide (**31**)

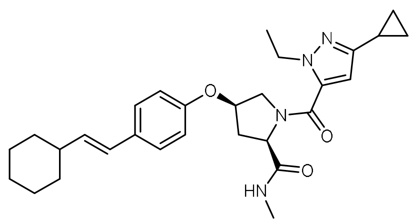

10ci (65 mg, 1 eq, 0.12 mmol), and 2-(3H-[1,2,3]triazolo[4,5-*b*]pyridin-3-yl)-1,1,3,3-tetramethylisouronium hexafluorophosphate(V) (53 mg, 1.2 eq, 0.14 mmol) were dissolved in CH_2Cl_2 (2 mL) and DIPEA (30 mg, 2.0 eq, 0.23 mmol). The reaction was stirred for 15 min at rt and methanamine hydrochloride (12 mg, 1.5 eq, 0.18 mmol) was added. The reaction was stirred for 16 hr at rt. The CH_2Cl_2 was removed under a stream of N_2, and the crude residue was diluted in EtOAc (25 mL). The organic layer was washed with water (1 x 25 mL), sat. aq. $NaHCO_3$ (1 x 25 mL) and brine (1 x 25 mL), dried over Na_2SO_4 and concentrated under reduced pressure. The crude residue was purified by flash chromatography (EtOAc/Hexanes gradient from 0% to 40% EtOAc, 4g column) to afford (9H-fluoren-9-yl)methyl (2*R*,4*R*)-4-(4-iodophenoxy)-2-(methylcarbamoyl)pyrrolidine-1-carboxylate (60 mg, 0.11 mmol, 90%) as a white solid.

A 5 mL µW vial equipped with a triangular magnetic stir bar was charged with (9H-fluoren-9-yl)methyl (2*R*,4*R*)-4-(4-iodophenoxy)-2-(methylcarbamoyl)pyrrolidine-1-carboxylate (60 mg, 1 eq, 0.11 mmol), (*E*)-(2-cyclohexylvinyl)boronic acid (16 mg, 1 eq, 0.11 mmol), Palladium-Xphos (17 mg, 0.20 eq, 21 µmol), DMF (1.0 mL) and 1.8 M aq. K_3PO_4 (0.13 g, 0.35 mL, 6.0 eq, 0.63 mmol). The mixture was sparged with Ar for 3 min before heating to 60 °C for 1 hr using a µW reactor. The reaction mixture was diluted with EtOAc (40 mL) and washed with H_2O (1 x 15mL) and brine (2 x 15 mL). The organic layer was dried over Na_2SO_4 and concentrated under reduced pressure. The crude residue was purified by flash chromatography (EtOAc/Hexanes gradient from 0% to 90% EtOAc, 4g column) to afford (9H-fluoren-9-yl)methyl (2*R*,4*R*)-4-(4-((*E*)-2-cyclohexylvinyl)phenoxy)-2-(methylcarbamoyl)pyrrolidine-1-carboxylate (26 mg, 47 µmol, 45%) as a light brown solid.

(9H-fluoren-9-yl)methyl (2*R*,4*R*)-4-(4-((*E*)-2-cyclohexylvinyl)phenoxy)-2-(methylcarbamoyl)pyrrolidine-1-carboxylate (26 mg, 1 eq, 47 µmol) was dissolved in DMF (0.52 mL) and piperidine (121 mg, 140 µL, 30 eq, 1.42 mmol) was added. The reaction mixture was allowed to stand for 45 min. *n*-Pentane (50 mL) was added and the volatiles were removed under reduced pressure. The process was repeated 2 additional times and the crude solid was purified by flash chromatography (0–10% MeOH [with 1% NH4OH] in CH_2Cl_2, 4 g silica column) to afford (2*R*,4*R*)-4-(4-((*E*)-2-cyclohexylvinyl)phenoxy)-*N*-methylpyrrolidine-2-carboxamide (12 mg, 37 µmol, 77%) as a white solid.

3-Cyclopropyl-1-ethyl-1H-pyrazole-5-carboxylic acid (16 mg, 1.2 eq, 91 µmol), and 2-(3H-[1,2,3]triazolo[4,5-*b*]pyridin-3-yl)-1,1,3,3-tetramethylisouronium hexafluorophosphate(V) (35 mg, 1.2 eq, 91 µmol) were dissolved in CH_2Cl_2 (2 mL) and DIPEA (25 mg, 33 µL, 2.5 eq, 0.19 mmol). The reaction

was stirred for 15 min at rt and (2*R*,4*R*)-4-(4-((*E*)-2-cyclohexylvinyl)phenoxy)-*N*-methylpyrrolidine-2-carboxamide (25 mg, 1 eq, 76 μmol) was added. The reaction was stirred for 16 hr at rt. The CH_2Cl_2 was removed under a stream of N_2, and the crude residue was diluted in EtOAc (25 mL). The organic layer was washed with water (1 x 25 mL), sat. aq. $NaHCO_3$ (1 x 25 mL) and brine (1 x 25 mL), dried over Na_2SO_4 and concentrated under reduced pressure. The crude residue was purified by preparative HPLC to afford **31** (10.5 mg, 58%).

^{1}H NMR (400 MHz, CD_3OD) (~3:2 mixture of rotamers) δ 7.28 (d, *J* = 8.3 Hz, 2H), 6.82 (d, *J* = 8.4 Hz, 2H), 6.41 (s, 0.6H), 6.30 (d, *J* = 15.9 Hz, 1H), 6.07 (app. dd, *J* = 15.9, 7.0 Hz, 1.4H), 5.12 – 4.99 (m, 1H), 4.74 (dd, *J* = 9.5, 3.7 Hz, 0.6H), 4.66 – 4.52 (m, 0.4H), 4.38 – 4.20 (m, 2H), 4.19 – 4.02 (m, 1H), 3.95 – 3.85 (m, 1H), 2.79 (s, 1.8H), 2.72 (s, 1.2H), 2.64 – 2.53 (m, 1H), 2.45 (d, *J* = 14.0 Hz, 0.4H), 2.35 (dt, *J* = 13.8, 3.6 Hz, 0.6H), 2.11 (dtt, *J* = 10.7, 6.9, 3.4 Hz, 1H), 2.00 – 1.85 (m, 1H), 1.85 – 1.75 (m, 4H), 1.75 – 1.66 (m, 1H), 1.45 – 1.15 (m, 8H), 0.99 – 0.89 (m, 2H), 0.77 – 0.62 (m, 2H). ^{13}C NMR (101 MHz, CD_3OD) δ 172.54, 162.12, 161.65, 155.74, 153.80, 135.31, 134.55, 131.76, 126.80, 126.51, 115.36, 103.72, 102.01, 75.26, 73.92, 61.57, 59.59, 54.42, 52.57, 45.59, 45.37, 41.13, 36.54, 34.30, 32.87, 25.89, 25.78, 25.17, 14.85, 14.67, 8.27, 6.99. HRMS (ESI): calculated for $C_{29}H_{39}N_3O_3$ $[M + H]^+$, 491.3017; found, 491.3014.

(2*R*,4*S*)-4-(4-((*E*)-2-cyclohexylvinyl)phenoxy)-1-(3-(2,3-dihydro-1H-inden-1-yl)propanoyl)-*N*-methylpyrrolidine-2-carboxamide (**32**)

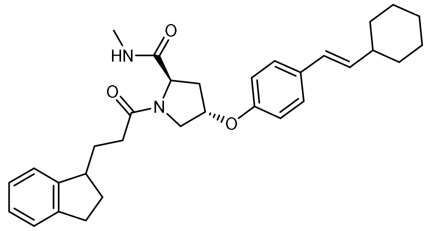

A solution of (2*R*,4*S*)-4-(4-iodophenoxy)-2-(methoxycarbonyl)pyrrolidin-1-ium 2,2,2-trifluoroacetate (80 mg, 1 eq, 0.17 mmol) and di-*tert*-butyl dicarbonate (45 mg, 1.2 eq, 0.21 mmol) in CH_2Cl_2 (1 mL) was added NEt_3 (21 mg, 1.2 eq, 0.21 mmol), and was stirred at rt for 16 hr. The reaction was concentrated, and the crude residue was purified by flash chromatography (0–70% EtOAc in hexanes, 12 g silica column) to afford 1-(*tert*-butyl) 2-methyl (2*R*,4*S*)-4-(4-iodophenoxy)pyrrolidine-1,2-dicarboxylate (52 mg, 0.12 mmol, 68%).

1-(*tert*-butyl) 2-methyl (2*R*,4*S*)-4-(4-iodophenoxy)pyrrolidine-1,2-dicarboxylate (50 mg, 1 eq, 0.11 mmol) was dissolved in THF:H_2O (3 mL) and LiOH (40 mg, 15 eq, 1.7 mmol) was added. The reaction mixture was stirred vigorously for 16 hr, and the reaction mixture was diluted with H_2O (15 mL). The aqueous layer was acidified with 1 N HCl to pH ~2 and extracted with CH_2Cl_2 (3 x 20 mL). The combined organics were dried over Na_2SO_4 and concentrated under reduced pressure to afford (2*R*,4*S*)-1-(*tert*-butoxycarbonyl)-4-(4-iodophenoxy)pyrrolidine-2-carboxylic acid (44 mg, 0.10 mmol, 93%) as a white solid.

(2*R*,4*S*)-1-(*tert*-butoxycarbonyl)-4-(4-iodophenoxy)pyrrolidine-2-carboxylic acid (43 mg, 1 eq, 99 μmol), and 2-(3H-[1,2,3]triazolo[4,5-*b*]pyridin-3-yl)-1,1,3,3-tetramethylisouronium hexafluorophosphate(V) (45 mg, 1.2 eq, 0.12 mmol) were dissolved in DMF (0.3 mL) and DIPEA (38 mg, 52 μL, 3.0 eq, 0.30 mmol). The reaction was stirred for 15 min at rt and methanamine hydrochloride (10 mg, 1.5 eq, 0.15 mmol) was added. The reaction was stirred for 16 hr at rt. The DMF was removed under a stream of N_2, and the crude residue was diluted in EtOAc (25 mL). The organic layer was washed with water (1 x 25 mL), sat. aq. $NaHCO_3$ (1 x 25 mL) and brine (1 x 25 mL), dried over Na_2SO_4 and concentrated under reduced pressure. The crude was purified by flash chromatography (0-40% EtOAc in hexanes, 4 g column) to afford *tert*-butyl (2*R*,4*S*)-4-(4-iodophenoxy)-2-(methylcarbamoyl)pyrrolidine-1-carboxylate (20 mg, 45 μmol, 45%).

A 5 mL μW vial equipped with a triangular magnetic stir bar was charged with *tert*-butyl (2*R*,4*S*)-4-(4-iodophenoxy)-2-(methylcarbamoyl)pyrrolidine-1-carboxylate (20 mg, 1 eq, 45 μmol), (*E*)-(2-cyclohexylvinyl)boronic acid (6.9 mg, 1 eq, 45 μmol), Palladium-Xphos (7.1 mg, 0.20 eq, 9.0 μmol), DMF (1.0 mL) and 1.8 M aq. K_3PO_4 (57 mg, 0.15 mL, 6.0 eq, 0.27 mmol). The mixture was purged with Ar for 3 min. The vial was then sealed and heated to 60 °C for 1 hr using a μW reactor. The reaction mixture was diluted with EtOAc (40 mL) and washed with H_2O (1 x 15mL) and brine (2 x 15 mL). dried over Na_2SO_4 and concentrated under reduced pressure. The crude residue was purified by column chromatography (0–90% EtOAc in hexanes, 4 g column) to afford *tert*-butyl (2*R*,4*S*)-4-(4-((*E*)-2-cyclohexylvinyl)phenoxy)-2-(methylcarbamoyl)pyrrolidine-1-carboxylate (18 mg, 42 μmol, 94%) as a white solid.

tert-Butyl (2*R*,4*S*)-4-(4-((*E*)-2-cyclohexylvinyl)phenoxy)-2-(methylcarbamoyl)pyrrolidine-1-carboxylate (20 mg, 1 eq, 47 μmol) was dissolved in HCl (1.5 g, 1.0 mL, 2 M solution in MeOH, 43 eq, 2.0 mmol). The reaction was stood at rt for 3 hr and volatiles were removed under reduced pressure to afford (2*R*,4*S*)-4-(4-((*E*)-2-cyclohexylvinyl)phenoxy)-*N*-methylpyrrolidine-2-carboxamide (quant.)

3-(2,3-Dihydro-1H-inden-1-yl)propanoic acid (4.0 mg, 1 eq, 21 μmol), and 2-(3H-[1,2,3]triazolo[4,5-*b*]pyridin-3-yl)-1,1,3,3-tetramethylisouronium hexafluorophosphate(V) (9.6 mg, 1.2 eq, 25 μmol) were dissolved in CH_2Cl_2 (3 mL) and *N*-ethyl-*N*-isopropylpropan-2-amine (6.8 mg, 9.1 μL, 2.5 eq, 53 μmol). The reaction was stirred for 15 min at rt and (2*R*,4*S*)-4-(4-((*E*)-2-cyclohexylvinyl)phenoxy)-*N*-methylpyrrolidine-2-carboxamide hydrochloride (7.7 mg, 1 eq, 21 μmol) was added. The reaction was stirred for 16 hr at rt. The reaction was diluted with CH_2Cl_2 (25 mL). The organic layer was washed with water (1 x 25 mL), sat. aq. $NaHCO_3$ (1 x 25 mL) and brine (1 x 25 mL), dried over Na_2SO_4 and concentrated under reduced pressure. The crude residue was purified by preparative HPLC to afford **32** (5.1 mg, 10 μmol, 49%) as a white solid.

^1H NMR (400 MHz, CD_3OD) (~2:1 mixture of rotamers) δ 7.33 – 7.24 (m, 2H), 7.23 – 7.05 (m, 4H), 6.88 – 6.81 (m, 2H), 6.31 (dd, *J* = 15.9, 9.5 Hz, 1H), 6.13 – 6.03 (m, 1H), 5.09 (td, *J* = 3.9, 1.9 Hz, 0.8H), 5.03 – 4.99 (m, 0.2H), 4.53 (dt, *J* = 19.0, 8.1 Hz, 1H), 4.05 (dt, *J* = 12.8, 1.9 Hz, 0.2H), 3.92 – 3.80 (m, 1.6H),

3.75 – 3.68 (m, 0.2H), 3.20 – 3.03 (m, 1H), 3.00 – 2.71 (m, 5H), 2.69 – 2.00 (m, 7H), 1.87 – 1.76 (m, 4H), 1.76 – 1.54 (m, 3H), 1.45 – 1.14 (m, 5H). ^{13}C NMR (101 MHz, CD_3OD) δ 173.52, 173.49, 155.70, 155.67, 146.29, 143.60, 143.54, 134.65, 134.62, 134.60, 131.85, 131.82, 126.94, 126.90, 126.83, 126.53, 126.51, 126.06, 125.78, 125.74, 123.99, 123.96, 123.19, 123.11, 115.48, 115.36, 75.59, 75.53, 58.96, 52.71, 44.09, 44.03, 41.15, 35.20, 35.15, 32.89, 32.87, 32.24, 32.07, 31.39, 31.34, 30.72, 30.68, 29.81, 25.89, 25.77, 24.96. HRMS (ESI): calculated for $C_{32}H_{41}N_2O_3$ [M + H]$^+$, 501.3122; found, 501.3127.

(2*R*,4*S*)-4-(4-((*E*)-2-cyclohexylvinyl)phenoxy)-1-(3-cyclopropyl-1-ethyl-1H-pyrazole-5-carbonyl)-*N*-methylpyrrolidine-2-carboxamide (**33**)

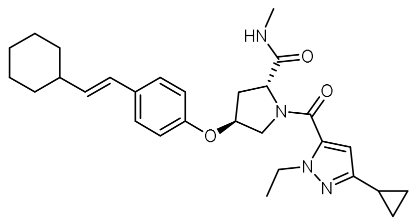

3-Cyclopropyl-1-ethyl-1H-pyrazole-5-carboxylic acid (4.5 mg, 1.2 eq, 25 µmol), and 2-(3H-[1,2,3]triazolo[4,5-*b*]pyridin-3-yl)-1,1,3,3-tetramethylisouronium hexafluorophosphate(V) (9.6 mg, 1.2 eq, 25 µmol) were dissolved in DMF (0.3 mL) and DIPEA (6.8 mg, 9.1 µL, 2.5 eq, 53 µmol). The reaction was stirred for 15 min at rt before the addition of (2*R*,4*S*)-4-(4-((*E*)-2-cyclohexylvinyl)phenoxy)-*N*-methylpyrrolidine-2-carboxamide hydrochloride (7.7 mg, 1 eq, 21 µmol) [*cf.* preparation of **32**]. The reaction was stirred for 16 hr at rt. The DMF was removed under a stream of N_2, and the crude residue was diluted in EtOAc (25 mL). The organic layer was washed with water (1 x 25 mL), sat. aq. $NaHCO_3$ (1 x 25 mL) and brine (1 x 25 mL), dried over Na_2SO_4 and concentrated under reduced pressure. The crude residue was purified by preparative HPLC to afford **33** (5.08 mg, 10.4 µmol, 49%) as a white solid.

^{1}H NMR (400 MHz, CD_3OD) (~3:1 mixture of rotamers) δ 7.31 (d, *J* = 8.6 Hz, 0.5H), 7.30 – 7.25 (m, 1.5H), 6.90 (d, *J* = 8.6 Hz, 0.5H), 6.86 – 6.78 (m, 1.5H), 6.31 (dd, *J* = 15.9, 7.9 Hz, 1H), 6.19 (s, 0.75H), 6.14 – 6.02 (m, 1.25H), 5.09 (t, *J* = 3.8 Hz, 0.25H), 5.05 (t, *J* = 3.8 Hz, 0.75H), 4.70 (dd, *J* = 9.4, 7.7 Hz, 0.75H), 4.60 (t, *J* = 8.2 Hz, 0.25H), 4.35 – 4.11 (m, 2H), 4.11 – 4.03 (m, 0.25H), 3.97 – 3.80 (m, 1.75H), 2.80 (s, 2.25H), 2.66 – 2.56 (m, 1H), 2.55 (s, 0.75H), 2.31 – 2.20 (m, 1H), 2.11 (dtd, *J* = 10.8, 7.3, 3.4 Hz, 1H), 1.98 – 1.85 (m, 1H), 1.85 – 1.75 (m, 4H), 1.75 – 1.67 (m, 1H), 1.45 – 1.17 (m, 8H), 0.97 – 0.85 (m, 2H), 0.73 – 0.61 (m, 2H). ^{13}C NMR (101 MHz, CD_3OD) δ 172.84, 161.78, 155.48, 153.96, 135.50, 134.69, 131.94, 126.87, 126.44, 115.46, 103.37, 101.98, 75.51, 59.00, 54.95, 52.38, 41.14, 37.28, 35.05, 32.86, 25.88, 25.77, 25.01, 24.86, 14.86, 14.52, 8.22, 7.00, 6.98. HRMS (ESI): calculated for $C_{29}H_{39}N_3O_3$ [M + H]$^+$, 491.3017; found, 491.3024.

(6*R*,7*S*,8*S*)-4-((3-Chloro-4-fluorophenyl)sulfonyl)-7-(4-((*E*)-2-cyclohexylvinyl)phenyl)-8-(hydroxymethyl)-1,4-diazabicyclo[4.2.0]octan-2-one (**34**)

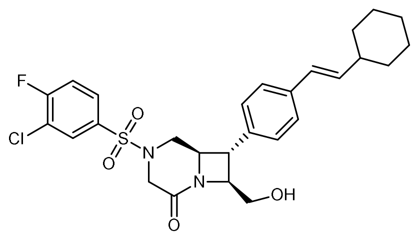

A 1-dram vial equipped with a magnetic stir bar was charged with (6*R*,7*S*,8*S*)-7-(4-bromophenyl)-8-(hydroxymethyl)-4-((2-nitrophenyl)sulfonyl)-1,4-diazabicyclo[4.2.0]octan-2-one (60 mg, 1 eq, 0.12 mmol). CH_2Cl_2 (1 mL) was added to give a yellow-tinted solution. 2,6-Lutidine (0.028 mL, 2.0 eq, 0.24 mmol) was added, followed by TBSOTf (0.042 mL, 1.5 eq, 0.18 mmol). The vial was blown out with nitrogen, tightly capped, stirred 10 hr, at which time the reaction was quenched by the addition of MeOH (0.1 mL) and allowed to stir for 30 min. Volatiles were evaporated under reduced pressure to reveal a viscous, opaque yellow oil which was dried under high vacuum overnight. To this crude material were added Cs_2CO_3 (0.20 g, 5.0 eq, 0.60 mmol) and DMF (1 mL), followed by 4-methoxythiophenol (0.059 mL, 4.0 eq, 0.48 mmol). The resulting yellow mixture was stirred for 2 hr, then transferred to a 100 mL round bottom flask (rinsing the reaction flask with ethyl acetate). Volatiles were removed under reduced pressure; DMF was removed by co-evaporation with heptane, affording a yellow residue, which was suspended in CH_2Cl_2 and washed with water. The organic portion was separated, dried over Na_2SO_4 and purified directly by flash chromatography (0–100% MeOH [containing 1% NH_4OH] in CH_2Cl_2, 4 g silico column), giving (6*R*,7*S*,8*S*)-7-(4-bromophenyl)-8-(((tert-butyldimethylsilyl)oxy)methyl)-1,4-diazabicyclo[4.2.0]octan-2-one (33 mg, 64% over two steps) as a pale yellow oil.

A 20 mL glass scintillation vial containing a magnetic stir bar and (6*R*,7*S*,8*S*)-7-(4-bromophenyl)-8-(((tert-butyldimethylsilyl)oxy)methyl)-1,4-diazabicyclo[4.2.0]octan-2-one (29 mg, 1.00 eq, 68 μmol) was charged with CH_2Cl_2 (0.75 mL), giving a colorless solution. 4-DMAP (0.075 mL of a 0.1 M solution in CH_2Cl_2, 0.11 eq, 7.5 μmol) and *N*,*N*-diisopropylethylamine (0.82 mL of a 0.1 M solution in CH_2Cl_2, 1.20 eq, 82 μmol) were then added, followed by 3-chloro-4-fluorobenzenesulfonyl chloride (0.75 mL of a 0.1 M solution in CH_2Cl_2, 1.10 eq, 75 μmol) positive N_2 pressure. The reaction vial was then capped, and the colorless reaction solution stirred for 2 hr. The reaction mixture was purified directly by flash chromatography (0–10% MeOH [containing 1% NH_4OH] in CH_2Cl_2, 4 g silica column) giving (6*R*,7*S*,8*S*)-7-(4-bromophenyl)-8-(((*tert*-butyldimethylsilyl)oxy)methyl)-4-((3-chloro-4-fluorophenyl)sulfonyl)-1,4-diazabicyclo[4.2.0]octan-2-one (40 mg, 95%) as a colorless solid.

A 2 mL μW vial containing a magnetic stir bar was charged with (*E*)-(2-cyclohexylvinyl)boronic acid (15 mg, 1.50 eq, 97 μmol) and Pd-Xphos-G2 (10 mg, 0.20 eq, 13 μmol). The vial was capped with a rubber septum and (6*R*,7*S*,8*S*)-7-(4-bromophenyl)-8-(((*tert*-butyldimethylsilyl)oxy)methyl)-4-((3-chloro-4-fluorophenyl)sulfonyl)-1,4-diazabicyclo[4.2.0]octan-2-one (40 mg, 1.00 eq, 65 μmol) was added as a solution in DMF (0.5 mL + 2 x 0.5 mL washes). K_3PO_4 (0.24 mL of a 1.6 M aq. solution, 6.00 eq, 0.39 mmol) was added, to afford an off-white cloudy suspension. The vial was then evacuated and backfilled with N_2

(5x), then heated to 60 °C for 1 hr under μW irradiation. The reaction mixture was concentrated under reduced pressure, then residual DMF was removed by azeotropic drying with heptane. The resulting residue was purified by flash chromatography (EtOAc in hexanes, 4 g silica column) to give (7*S*,8*S*)-8-(((*tert*-butyldimethylsilyl)oxy)methyl)-4-((3-chloro-4-fluorophenyl)sulfonyl)-7-(4-((*E*)-2-cyclohexylvinyl)phenyl)-1,4-diazabicyclo[4.2.0]octan-2-one (20 mg, 31 μmol, 48%).

This resulting material was dissolved in DMF (250 μL), followed by addition of water (3 μL, 5 eq, 155 μmol,). TAS-F (21 mg, 2.5 eq, 77.5 μmol) was added in one portion and the resulting solution was stirred at 50 °C for 40 min. Upon completion, the reaction mixture was purified by preparative HPLC to afford **34** (1.5 mg, 2.8 μmol, 9%).

^{1}H NMR (400 MHz, $CDCl_3$) δ 7.88 (dd, *J* = 6.6, 2.3 Hz, 1H), 7.70 (ddd, *J* = 8.6, 4.3, 2.3 Hz, 1H), 7.38 – 7.32 (m, 3H), 7.15 (d, *J* = 8.0 Hz, 2H), 6.33 (d, *J* = 15.9 Hz, 1H), 6.20 (dd, *J* = 16.0, 6.8 Hz, 1H), 4.82 – 4.66 (m, 2H), 4.25 (d, *J* = 16.8 Hz, 1H), 4.17 (dd, *J* = 11.6, 4.5 Hz, 1H), 4.10 – 4.00 (m, 1H), 3.97 (dd, *J* = 9.7, 3.4 Hz, 1H), 3.79 – 3.67 (m, 1H), 3.48 (t, *J* = 7.6 Hz, 1H), 3.41 (d, *J* = 16.8 Hz, 1H), 2.70 (t, *J* = 10.9 Hz, 1H), 2.19 – 2.07 (m, 1H), 1.85 – 1.74 (m, 4H), 1.69 (d, *J* = 12.6 Hz, 1H), 1.39 – 1.13 (m, 6H). ^{13}C NMR (101 MHz, $CDCl_3$) δ 164.39, 138.28, 137.98, 134.74, 130.35, 127.98, 127.89, 127.10, 126.62, 126.32, 117.98, 117.76, 77.21, 74.73, 65.33, 63.08, 48.43, 47.59, 44.72, 41.17, 32.88, 26.15, 26.01. LRMS (ESI): calculated for $C_{27}H_{31}ClFN_2O_4S$ $[M + H]^+$, 533.17; found 533.35.

N-(((4*R*,5*R*)-8-((*E*)-2-Cyclohexylvinyl)-2-((*R*)-1-hydroxypropan-2-yl)-4-methyl-1,1-dioxido-2,3,4,5-tetrahydrobenzo[*b*][1,4,5]oxathiazocin-5-yl)methyl)-*N*-methyl-2-phenylcyclopent-1-ene-1-carboxamide (**35**)

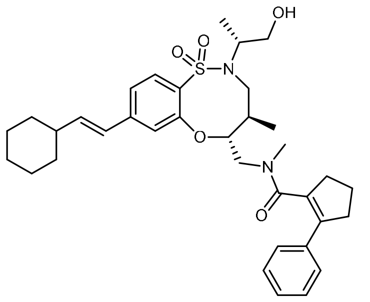

A 20 mL scintillation vial equipped with a magnetic stir bar was charged with (9H-fluoren-9-yl)methyl (((4*R*,5*R*)-8-bromo-2-((*R*)-1-hydroxypropan-2-yl)-4-methyl-1,1-dioxido-2,3,4,5-tetrahydrobenzo[*b*][1,4,5]oxathiazocin-5-yl)methyl)(methyl)carbamate (65.3 mg, 1.00 eq, 104 μmol), dry DMF (3.5 mL) and dry piperidine (0.16 g, 0.18 mL, 18 eq, 1.8 mmol) at rt. The clear, colorless solution was stirred for 20 min. It was then concentrated under reduced pressure to afford a white solid. The material was absorbed on silica gel and purified by flash chromatography (4–8% MeOH [containing 1% NH_4OH] in CH_2Cl_2) affording (4*R*,5*R*)-8-bromo-2-((*R*)-1-hydroxypropan-2-yl)-4-methyl-5-

((methylamino)methyl)-2,3,4,5-tetrahydrobenzo[*b*][1,4,5]oxathiazocine 1,1-dioxide as a colorless oil (28.2 mg, 69.2 µmol, 67%).

A 2-dram scintillation vial equipped with a magnetic stir bar was charged with (4*R*,5*R*)-8-bromo-2-((*R*)-1-hydroxypropan-2-yl)-4-methyl-5-((methylamino)methyl)-2,3,4,5-tetrahydrobenzo[*b*][1,4,5]oxathiazocine 1,1-dioxide (28.2 mg, 1.00 eq, 69.2 µmol), dry DMF (0.69 mL), DIPEA (25 µL, 2.1 eq, 0.15 mmol), 2-phenylcyclopent-1-ene-1-carboxylic acid (95% wt, 15.5 mg, 1.13 eq, 78.2 µmol) and PyBOP (39.6 mg, 1.1 eq, 76.2 µmol) at rt. The clear, faint brown solution was stirred for 25 min. EtOAc and water were added, and the aqueous phase was extracted twice with EtOAc. The organic layers were washed with water and were then combined, dried over Na_2SO_4 and concentrated under reduced pressure. The crude material was purified by flash chromatography (0–8% MeOH in CH_2Cl_2), affording *N*-(((4*R*,5*R*)-8-bromo-2-((*R*)-1-hydroxypropan-2-yl)-4-methyl-1,1-dioxido-2,3,4,5-tetrahydrobenzo[*b*][1,4,5]oxathiazocin-5-yl)methyl)-*N*-methyl-2-phenylcyclopent-1-ene-1-carboxamide (35.3 mg, 61.1 µmol, 88%) as a colorless oil.

A 5 mL cone-shaped µW vial equipped with a magnetic stir bar was charged with *N*-(((4*R*,5*R*)-8-bromo-2-((*R*)-1-hydroxypropan-2-yl)-4-methyl-1,1-dioxido-2,3,4,5-tetrahydrobenzo[*b*][1,4,5]oxathiazocin-5-yl)methyl)-*N*-methyl-2-phenylcyclopent-1-ene-1-carboxamide (35.3 mg, 1.00 eq, 61.1 µmol) in dry DMF (0.61 mL) at rt. (*E*)-(2-Cyclohexylvinyl)boronic acid (98% wt, 14.4 mg, 1.5 eq, 91.7 µmol), XPhos-Pd-G2 (9.6 mg, 20 mol%, 12 µmol) and aq. K_3PO_4 (1.8 M, 0.20 mL, 5.9 eq) were successively added and the biphasic mixture was sparged with Ar for 3 min. The vial was then sealed, and the reaction mixture was heated to 60 °C for 3 hr using µW irradiation. More XPhos-Pd-G2 (4.8 mg, 10 mol%, 6.1 µmol) and aq. K_3PO_4 (1.8 M, 0.10 mL, 0.18 mmol, 3.0 eq) were added after 2 hr. EtOAc was added, and the mixture was filtered over cotton. The filtrate was concentrated under reduced pressure and the obtained crude material was purified by flash chromatography (20–80% EtOAc in hexanes) affording the product as a yellow oil. The material was further purified by preparative HPLC to afford **35** (19.6 mg, 32.3 µmol, 53%) as a white foam.

^{1}H NMR (400 MHz, $CDCl_3$) (~3:2 mixture of rotamers) δ 7.73 (dd, *J* = 8.3, 6.1 Hz, 1H), 7.31 – 7.13 (m, 5H), 7.08 (ddd, *J* = 13.5, 8.2, 1.7 Hz, 1H), 6.95 (d, *J* = 1.7 Hz, 0.6H), 6.86 (d, *J* = 1.7 Hz, 0.4H), 6.36 (dd, *J* = 16.0, 6.8 Hz, 0.4H), 6.28 – 6.18 (m, 1.6H), 4.50 (td, *J* = 8.7, 2.6 Hz, 0.6H), 4.28 – 4.08 (m, 1H), 3.80 – 3.67 (m, 1H), 3.66 – 3.38 (m, 4H), 3.34 (dd, *J* = 15.8, 4.9 Hz, 0.4H), 3.23 (dd, *J* = 14.1, 2.6 Hz, 0.6H), 2.99 – 2.36 (m, 8.75H), 2.23 – 1.93 (m, 4.25H), 1.83 – 1.62 (m, 5.2H), 1.34 – 1.07 (m, 8.8H), 0.91 (d, *J* = 6.9 Hz, 1.75H), 0.61 (d, *J* = 6.9 Hz, 1.25H). ^{13}C NMR (101 MHz, $CDCl_3$) δ 172.08, 171.69, 155.99, 155.34, 144.96, 144.30, 141.80, 141.15, 140.15, 139.88, 136.22, 136.16, 133.02, 132.91, 130.82, 130.51, 130.02, 129.92, 128.58, 127.98, 127.91, 126.78, 126.72, 125.66, 125.32, 122.27, 121.40, 120.77, 120.73, 85.10, 84.69, 77.36, 64.96, 64.86, 57.47, 57.34, 53.96, 49.76, 49.50, 49.33, 41.38, 37.42, 37.13, 36.92, 36.65, 36.44, 36.34, 36.17, 33.21, 32.79, 32.76, 32.67, 32.65, 26.18, 26.06, 22.61, 22.58, 15.97, 15.83, 15.75, 15.31. HRMS (ESI): calculated for $C_{35}H_{47}N_2O_5S$ $[M + H]^+$, 607.3200; found, 607.3219.

N-(((4*S*,5*S*)-8-((*E*)-2-Cyclohexylvinyl)-2-((*R*)-1-hydroxypropan-2-yl)-4-methyl-1,1-dioxido-2,3,4,5-tetrahydrobenzo[*b*][1,4,5]oxathiazocin-5-yl)methyl)-*N*-methyl-2-(3-methylthiophen-2-yl)cyclopropane-1-carboxamide (**36**)

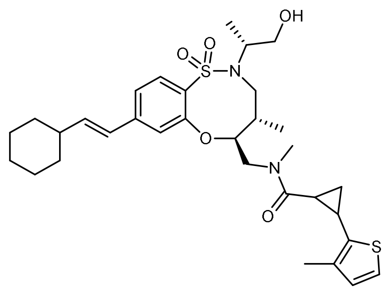

A 2 dram scintillation vial equipped with a magnetic stir bar was charged with (9H-fluoren-9-yl)methyl (((4*S*,5*S*)-8-bromo-2-((*R*)-1-hydroxypropan-2-yl)-4-methyl-1,1-dioxido-2,3,4,5-tetrahydrobenzo[*b*][1,4,5]oxathiazocin-5-yl)methyl)(methyl)carbamate (52.1 mg, 1.00 eq, 82.8 μmol), dry DMF (2.8 mL) and dry piperidine (0.14 mL, 17 eq, 1.4 mmol) at rt. The colorless solution was stirred for 40 min. It was then concentrated under reduced pressure and the obtained white solid was purified by flash chromatography (0–10% MeOH [containing 1% NH_4OH] in CH_2Cl_2) to afford (4*S*,5*S*)-8-bromo-2-((*R*)-1-hydroxypropan-2-yl)-4-methyl-5-((methylamino)methyl)-2,3,4,5-tetrahydrobenzo[*b*][1,4,5]oxathiazocine 1,1-dioxide (31.0 mg, 76.1 μmol, 92%) as a colorless oil.

A 2 dram scintillation vial equipped with a magnetic stir bar was charged with (4*S*,5*S*)-8-bromo-2-((*R*)-1-hydroxypropan-2-yl)-4-methyl-5-((methylamino)methyl)-2,3,4,5-tetrahydrobenzo[*b*][1,4,5]oxathiazocine 1,1-dioxide (31.0 mg, 1 eq, 76.1 μmol), *racemic* *trans*-2-(3-methylthiophen-2-yl)cyclopropane-1-carboxylic acid (0.22 M in DMF, 0.45 mL, 1.3 eq, 99 μmol), DIPEA (21 mg, 28 μL, 2.1 eq, 0.16 mmol) and PyBOP (0.22 M in DMF, 0.45 mL, 0.22 molar, 1.3 eq, 99 μmol) at r.t. The light brown solution was stirred for 50 min. EtOAc and water (each 30 mL) were added and the aqueous phase was extracted twice with EtOAc. The organic layers were washed two more times with water. They were combined, dried over $MgSO_4$ and concentrated under reduced pressure. Purification of the crude material by flash chromatography (0–10% MeOH [containing 1% NH_4OH] in CH_2Cl_2) afforded *N*-(((4*S*,5*S*)-8-bromo-2-((*R*)-1-hydroxypropan-2-yl)-4-methyl-1,1-dioxido-2,3,4,5-tetrahydrobenzo[*b*][1,4,5]oxathiazocin-5-yl)methyl)-*N*-methyl-2-(3-methylthiophen-2-yl)cyclopropane-1-carboxamide (34 mg, 0.060 mmol, 78%) as a mixture of both diastereomers and rotamers.

A 5 mL cone-shaped μW vial equipped with a magnetic stir bar was charged with *N*-(((4*S*,5*S*)-8-bromo-2-((*R*)-1-hydroxypropan-2-yl)-4-methyl-1,1-dioxido-2,3,4,5-tetrahydrobenzo[*b*][1,4,5]oxathiazocin-5-yl)methyl)-*N*-methyl-2-(3-methylthiophen-2-yl)cyclopropane-1-carboxamide (34 mg, 1.0 eq, 59 μmol) in dry DMF (0.59 mL), (*E*)-(2-cyclohexylvinyl)boronic acid (14 mg, 1.5 eq, 89 μmol), XPhos-Pd-G2 (9.4 mg, 20 mol%, 12 μmol) and aq. K_3PO_4 (1.8 M, 0.10 mL, 3.0 eq, 0.18 mmol) at r.t. The reaction mixture was purged with Argon for two min and the vial was sealed. The mixture was heated to 60 °C for 1 hour using

μW irradiation. More aq. K_3PO_4 (0.26 g, 0.10 mL, 1.8 molar, 3.0 eq, 0.18 mmol) was added after 30 min. EtOAc was added and the mixture was filtered over Celite. The filtrate was concentrated under reduced pressure and the crude material was purified by flash chromatography (25–75% EtOAc in hexanes) to afford a yellow oil, that was further purified by preparative HPLC to **36** (19.6 mg, 32.6 μmol, 55%) as a white solid and as a mixture of rotamers and diastereomers (ratio approximately 1:1:1:1, according to analysis by ^{1}H NMR spectroscopy).

^{1}H NMR (400 MHz, CD_3OD) δ 7.72 (dt, *J* = 8.2, 2.1 Hz, 1H), 7.24 – 7.09 (m, 1H), 7.07 – 6.90 (m, 2H), 6.75 (dd, *J* = 12.6, 5.1 Hz, 1H), 6.43 – 6.14 (m, 2H), 4.72 – 4.63 (m, 0.5H), 4.58 (ddd, *J* = 10.7, 9.0, 2.9 Hz, 0.25H), 4.44 (ddd, *J* = 11.3, 8.9, 2.8 Hz, 0.25H), 4.28 (td, *J* = 15.0, 10.4 Hz, 0.5H), 4.18 – 4.05 (m, 0.5H), 3.85 – 3.49 (m, 5.75H), 3.46 (dd, *J* = 14.1, 2.8 Hz, 0.25H), 3.36 (dd, *J* = 13.9, 2.7 Hz, 0.25H), 3.22 (s, 0.75H), 3.08 (s, 0.75H), 2.86 (s, 0.75H), 2.77 (s, 1H), 2.58 – 2.38 (m, 1.25H), 2.35 – 2.03 (m, 5.25H), 2.02 – 1.92 (m, 0.5H), 1.86 – 1.63 (m, 5.5H), 1.50 – 1.03 (m, 10H), 0.95 – 0.90 (m, 2.25H), 0.86 (d, *J* = 6.9 Hz, 0.75H). ^{13}C NMR (101 MHz, methanol-d^4) δ 174.66, 174.60, 174.46, 174.19, 157.18, 157.16, 157.01, 156.66, 145.78, 145.58, 145.37, 145.34, 142.20, 142.17, 141.70, 141.62, 138.53, 138.43, 138.37, 138.27, 136.32, 136.28, 136.21, 133.99, 133.64, 133.59, 133.51, 131.40, 131.31, 131.26, 130.47, 130.41, 130.21, 126.95, 126.91, 126.75, 126.66, 123.22, 123.15, 122.18, 122.16, 122.07, 122.00, 121.89, 121.66, 121.38, 86.34, 85.22, 85.13, 84.66, 65.73, 65.64, 65.52, 58.86, 58.51, 58.46, 58.38, 54.90, 54.61, 53.32, 51.28, 51.19, 51.04, 50.98, 49.71, 42.65, 42.58, 42.55, 42.42, 38.19, 38.02, 37.98, 37.92, 37.23, 36.69, 34.88, 34.35, 33.89, 33.81, 33.67, 33.64, 27.20, 27.14, 27.07, 27.02, 26.97, 24.69, 24.22, 23.96, 23.66, 21.30, 20.80, 20.40, 20.08, 18.62, 17.74, 17.66, 17.57, 15.92, 15.71, 15.27, 15.22, 15.10, 14.83, 14.10, 13.99, 13.88, 13.78. HRMS (ESI): calculated for $C_{32}H_{45}N_2O_5S_2$ $[M + H]^+$, 601.2764; found, 601.2778.

N-(((4*S*,5*S*)-8-(2-Chloro-5-(hydroxymethyl)phenyl)-2-((*R*)-1-hydroxypropan-2-yl)-4-methyl-1,1-dioxido-2,3,4,5-tetrahydrobenzo[*b*][1,4,5]oxathiazocin-5-yl)methyl)-6-ethyl-*N*-methyl-2-oxo-1,2-dihydropyridine-4-carboxamide (**37**)

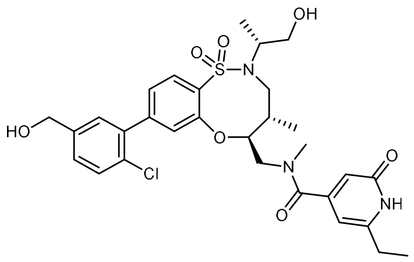

A 2 dram scintillation vial equipped with a magnetic stir bar was charged with (4*S*,5*S*)-8-bromo-2-((*R*)-1-hydroxypropan-2-yl)-4-methyl-5-((methylamino)methyl)-2,3,4,5-tetrahydrobenzo[*b*][1,4,5]oxathiazocine 1,1-dioxide [*cf.* synthesis of **36**] (30.8 mg, 1 eq, 75.6 μmol) at r.t. Dry DMF (0.76 mL), DIPEA (28 μL, 2.1 eq, 0.16 mmol), 6-ethyl-2-oxo-1,2-dihydropyridine-4-carboxylic acid (16.4 mg, 1.3 eq, 98.3 μmol) and PyBOP (51.2 mg, 1.3 eq, 98.3 μmol) were successively added and the obtained brown solution was stirred for 50 min at rt. EtOAc and water–brine (9:1) (each 30 mL) were added and the aqueous phase

was extracted twice with EtOAc. The organic layers were washed two more times with water:brine (9:1). They were combined, dried over Na_2SO_4 and concentrated under reduced pressure to afford a yellow oil. The crude material was purified by flash chromatography (0–10% MeOH in CH_2Cl_2), then further purified by preparative HPLC to afford *N*-(((4*S*,5*S*)-8-bromo-2-((*R*)-1-hydroxypropan-2-yl)-4-methyl-1,1-dioxido-2,3,4,5-tetrahydrobenzo[*b*][1,4,5]oxathiazocin-5-yl)methyl)-6-ethyl-*N*-methyl-2-oxo-1,2-dihydropyridine-4-carboxamide (22.8 mg, 41.0 µmol, 54%) as a white foam.

A 5 mL cone-shaped µW vial equipped with a magnetic stir bar was charged with *N*-(((4*S*,5*S*)-8-bromo-2-((*R*)-1-hydroxypropan-2-yl)-4-methyl-1,1-dioxido-2,3,4,5-tetrahydrobenzo[*b*][1,4,5]oxathiazocin-5-yl)methyl)-6-ethyl-*N*-methyl-2-oxo-1,2-dihydropyridine-4-carboxamide (22.8 mg, 1.00 eq, 41.0 µmol) in dry DMF (0.41 mL) at rt. (2-chloro-5-(hydroxymethyl)phenyl)boronic acid (11.5 mg, 1.5 eq, 61.5 µmol), XPhos-Pd-G2 (6.5 mg, 20 mol%, 8.2 µmol) and aq. K_3PO_4 (1.8 M, 0.14 mL, 6.2 eq, 0.25 mmol) were successively added. The mixture was sparged with Ar for 3 min, the vial was sealed, and the reaction mixture was heated to 60 °C for 4 hr using µW irradiation. EtOAc was added and the mixture was filtered over cotton. The filtrate was concentrated under reduced pressure and the crude material was purified by flash chromatography (0–10% MeOH in CH_2Cl_2), then further purified by preparative HPLC to afford **37** (7.4 mg, 12 µmol, 29%) as a white foam.

^1H NMR (400 MHz, CD_3OD) (1:1 mixture of rotamers) δ 7.94 (d, *J* = 8.2 Hz, 1H), 7.52 – 7.30 (m, 4H), 7.28 (d, *J* = 1.8 Hz, 0.5H), 7.13 (d, *J* = 1.8 Hz, 0.5H), 6.41 (s, 0.5H), 6.10 (s, 0.5H), 6.01 (s, 0.5H), 5.85 (s, 0.5H), 4.63 (d, *J* = 21.1 Hz, 2H), 4.59 – 4.52 (m, 0.5H), 4.18 (dd, *J* = 14.2, 8.8 Hz, 0.5H), 3.81 – 3.55 (m, 6.5H), 3.05 (s, 1.5H), 2.93 (s, 1.5H), 2.58 (q, *J* = 7.6 Hz, 1H), 2.43 (q, *J* = 7.6 Hz, 1H), 2.34 (p, *J* = 7.5 Hz, 0.5H), 2.22 – 2.09 (m, 0.5H), 1.36 – 1.08 (m, 6.5H), 1.02 (d, *J* = 6.9 Hz, 1.5H), 0.83 (d, *J* = 6.9 Hz, 1.5H). ^{13}C NMR (101 MHz, CD_3OD) δ 171.24, 171.16, 165.76, 165.55, 156.57, 156.08, 154.30, 154.27, 154.26, 151.19, 151.06, 146.68, 146.35, 142.71, 139.25, 139.20, 135.31, 135.20, 131.58, 131.21, 131.17, 130.88, 130.59, 130.24, 129.31, 129.26, 126.74, 126.28, 125.68, 125.43, 115.30, 114.84, 104.18, 103.39, 86.17, 84.85, 65.62, 65.49, 64.20, 64.17, 59.00, 58.57, 55.90, 52.36, 51.18, 50.91, 49.28, 39.11, 38.10, 37.94, 34.05, 27.17, 27.15, 27.12, 16.12, 15.90, 15.52, 14.84, 13.35, 13.05. HRMS (ESI): calculated for $C_{30}H_{37}ClN_3O_7S$ $[M + H]^+$, 618.2035; found, 618.2053

N-(((4*R*,5*R*)-8-((*E*)-2-Cyclohexylvinyl)-2-((*S*)-1-hydroxypropan-2-yl)-4-methyl-1,1-dioxido-2,3,4,5-tetrahydrobenzo[*b*][1,4,5]oxathiazocin-5-yl)methyl)-*N*-methyl-2-phenylcyclopent-1-ene-1-carboxamide (**38**)

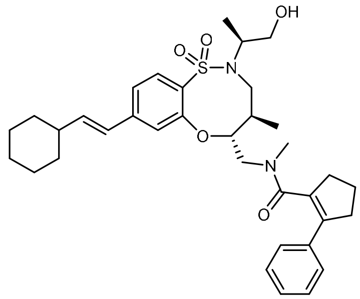

To a 0.5 mL µW vial was added (9H-fluoren-9-yl)methyl (((4*R*,5*R*)-8-bromo-2-((*S*)-1-hydroxypropan-2-yl)-4-methyl-1,1-dioxido-2,3,4,5-tetrahydrobenzo[*b*][1,4,5]oxathiazocin-5-yl)methyl)(methyl)carbamate (20 mg, 1 eq, 32 µmol), (*E*)-(2-cyclohexylvinyl)boronic acid (7.3 mg, 1.50 eq, 48 µmol) and Palladium-Xphos-G2 (5.0 mg, 0.20 eq, 6.4 µmol). The vial was equipped with a magnetic stir bar, capped with a rubber septum and charged with DMF (0.3 mL), giving, after light stirring, a colorless, homogenous solution. This solution was degassed by evacuating at high vacuum and backfilling with N_2 (3 x). 1.6 M aq. K_3PO_4 (0.12 mL, 6 eq, 0.19 mmol) was then added through the septum. A yellow color evolved quickly; the reaction vessel was heated under µW irradiation at 60 °C for 45 min. The mixture was allowed to reach rt before addition of piperidine (0.06 mL) and stirring for 6 hr. The mixture was then concentrated under reduced pressure and purified by flash chromatography (0–100% MeOH [containing 1% NH_4OH] in CH_2Cl_2, 4 g silica column) giving (4*R*,5*R*)-8-((*E*)-2-cyclohexylvinyl)-2-((*S*)-1-hydroxypropan-2-yl)-4-methyl-5-((methylamino)methyl)-2,3,4,5-tetrahydrobenzo[*b*][1,4,5]oxathiazocine 1,1-dioxide (15 mg) as a brown residue. The residue was charged to a 20 mL glass scintillation vial containing a magnetic stir bar, then treated with a pre-incubated (10 min) solution of 2-phenylcyclopent-1-ene-1-carboxylic acid (10 mg, 1.5 eq, 53 µmol), PyBOP (30 mg, 1.7 eq, 58 µmol) and DIPEA (15 µL, 2.50 eq, 86 µmol) in DMF (0.1 mL). The walls of the vial were rinsed with additional DMF (0.3 mL), and the resulting brown solution was stirred for 1.25 hr. The reaction solution was partitioned between EtOAc and water and the resulting layers separated. The aqueous phase was extracted with EtOAc (4 x 1 mL) and the combined organics dried over Na_2SO_4. The organics were decanted, concentrated under reduced pressure and purified by flash chromatography (0–100% MeOH [containing 1% NH_4OH] in CH_2Cl_2, 4 g silica column), followed by preparative HPLC to afford **38** (10.5 mg, 50%, 17 µmol) as a white solid.

^1H NMR (400 MHz, $CDCl_3$) 1 : 1 mixture of rotamers δ 7.79 (dd, *J* = 8.3, 4.3 Hz, 1H), 7.30 (t, *J* = 3.9 Hz, 3.7H), 7.26 – 7.22 (m, 0.8H), 7.15 (ddd, *J* = 15.8, 8.3, 1.7 Hz, 1H), 7.01 (d, *J* = 1.7 Hz, 0.5H), 6.96 (d, *J* = 1.7 Hz, 0.5H), 6.40 (dd, *J* = 16.0, 6.8 Hz, 0.5H), 6.29 – 6.19 (m, 1.5H), 4.70 (td, *J* = 8.8, 2.6 Hz, 0.5H), 4.46 – 4.35 (m, 1H), 3.95 – 3.75 (m, 2H), 3.65 – 3.56 (m, 1H), 3.56 – 3.43 (m, 1.5H), 3.43 – 3.20 (m, 2H), 3.20 – 3.12 (m, 0.5H), 3.06 – 2.69 (m, 4H), 2.67 (s, 1.4H), 2.63 (s, 1.5H), 2.62 – 2.53 (m, 0.6H), 2.21 – 2.07 (m, 2.5H), 2.06 – 1.92 (m, 1.5H), 1.87 – 1.73 (m, 4H), 1.73 – 1.64 (m, 1H), 1.64 – 1.57 (m, 0.5H), 1.38 – 1.10 (m, 8H), 0.95 (d, *J* = 6.8 Hz, 1.5H), 0.64 (d, *J* = 6.8 Hz, 1.5H). ^{13}C NMR (101 MHz, $CDCl_3$) δ 171.89, 171.40, 154.38, 153.64, 144.70, 143.95, 141.78, 141.01, 140.21, 139.67, 136.18, 136.05, 132.88, 132.87, 131.49, 131.16, 129.54, 129.33, 128.43, 128.41, 127.84, 127.71, 126.58, 125.45, 125.07, 122.78, 121.87, 120.79,

84.16, 82.81, 77.20, 64.51, 64.48, 57.59, 57.47, 53.51, 49.35, 47.55, 47.10, 41.22, 41.20, 37.19, 37.02, 36.90, 36.44, 36.31, 36.19, 35.82, 32.65, 32.59, 32.51, 32.43, 26.02, 25.91, 25.89, 22.50, 22.43, 15.69, 15.62, 15.09, 14.53. HRMS (ESI): calculated for $C_{35}H_{47}N_2O_5S$ [M + H]$^+$, 607.3200; found, 607.3196.

N-(((4*R*,5*R*)-8-((*E*)-2-Cyclohexylvinyl)-2-((*S*)-1-hydroxypropan-2-yl)-4-methyl-1,1-dioxido-2,3,4,5-tetrahydrobenzo[*b*][1,4,5]oxathiazocin-5-yl)methyl)-2-(2,3-dihydrobenzofuran-3-yl)-*N*-methylacetamide (**39**)

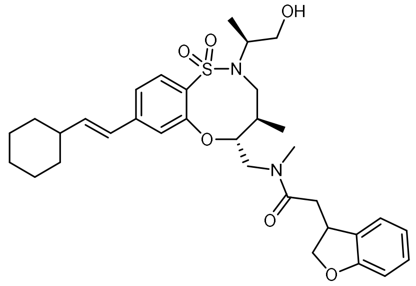

In a 2 mL microwave reaction vial, a solution of (9H-fluoren-9-yl)methyl (((4*R*,5*R*)-8-bromo-2-((*S*)-1-hydroxypropan-2-yl)-4-methyl-1,1-dioxido-2,3,4,5-tetrahydrobenzo[*b*][1,4,5]oxathiazocin-5-yl)methyl)(methyl)carbamate (36 mg, 1.0 eq, 0.057 mmol), XPhos-Pd-G2 (9 mg, 0.2 eq, 0.011 mmol) (*E*)-(2-cyclohexylvinyl)boronic acid (13.2 mg, 1.5 eq, 0.085 mmol) and 1.8 M aq. K_3PO_4 (95 µL, 3 eq, 0.171 mmol) in DMF (0.6 mL) was deaerated with Ar at rt for 2 min. The vial was sealed and heated under µW irradiation at 60 °C for 50 min. The solvent was evaporated under the reduced pressure, and the residue was diluted with EtOAc (50 mL), washed with water (10 mL), and brine (10 mL), then was dried over anhydrous. Na_2SO_4, was filtered and concentrated under reduced pressure. The residue was purified by flash chromatography (0–60% EtOAc in hexanes) to afford (9H-fluoren-9-yl)methyl (((4*R*,5*R*)-8-((*E*)-2-cyclohexylvinyl)-2-((*S*)-1-hydroxypropan-2-yl)-4-methyl-1,1-dioxido-2,3,4,5-tetrahydrobenzo[*b*][1,4,5]oxathiazocin-5-yl)methyl)(methyl)carbamate (29 mg, 0.044 mmol, 77%) as an off-white foam.

In a 4 mL vial, piperidine (50 µL) was added to a solution of (9H-fluoren-9-yl)methyl (((4*R*,5*R*)-8-((*E*)-2-cyclohexylvinyl)-2-((*S*)-1-hydroxypropan-2-yl)-4-methyl-1,1-dioxido-2,3,4,5-tetrahydrobenzo[*b*][1,4,5]oxathiazocin-5-yl)methyl)(methyl)carbamate (19 mg, 1.0 eq, 0.029 mmol) in DMF (1 mL) at rt and the reaction was stirred at rt for 30 min. The solvent was evaporated, and the residue was purified by flash chromatography (0–10% MeOH [with 1% NH_4OH] in CH_2Cl_2) to afford (4*R*,5*R*)-8-((*E*)-2-cyclohexylvinyl)-2-((*S*)-1-hydroxypropan-2-yl)-4-methyl-5-((methylamino)methyl)-2,3,4,5-tetrahydrobenzo[*b*][1,4,5]oxathiazocine 1,1-dioxide (9 mg, 0.021 mmol, 71%) as an off-white foam.

In an 8 mL vial DIPEA (0.019 g, 2.1 eq, 0.15 mmol) was added to a solution of 2-(2,3-dihydrobenzofuran-3-yl)acetic acid (4 mg, 1.0 eq, 0.021 mmol), (4*R*,5*R*)-8-((*E*)-2-cyclohexylvinyl)-2-((*S*)-1-hydroxypropan-2-yl)-4-methyl-5-((methylamino)methyl)-2,3,4,5-tetrahydrobenzo[*b*][1,4,5]oxathiazocine 1,1-dioxide (8 mg,

1.0 eq, 0.058 mmol), and PyBOP (12 mg, 1.1 eq, 0.022 mmol) in DMF (1 mL) at rt and stirred for 30–45 min. The solvent was evaporated under reduced pressure and the residue was purified by flash chromatography (0–10% MeOH [with 1% NH_4OH] in CH_2Cl_2) to afford a clear oil, which was further purified by preparative HPLC to afford **39** (8 mg, 0.038 mmol, 65%) as an off-white solid, and as a mixture of rotamers and diastereomers (ratio approximately 1:1:1:1, according to analysis by ^{1}H NMR spectroscopy).

1H NMR (400 MHz, $CDCl_3$) δ 7.81 (ddd, *J* = 8.0, 6.5, 1.4 Hz, 1H), 7.25 – 7.08 (m, 3H), 6.93 – 6.75 (m, 3H), 6.41 – 5.99 (m, 2H), 4.96 – 4.57 (m, 2H), 4.36 – 4.13 (m, 2H), 4.12 – 3.69 (m, 4H), 3.62 (dt, *J* = 13.1, 9.6 Hz, 1H), 3.56 – 3.44 (m, 1H), 3.38 (dt, *J* = 15.8, 5.9 Hz, 1H), 3.33 – 3.13 (m, 1H), 2.84 (s, 2H), 2.80 – 2.61 (m, 2H), 2.60 – 2.30 (m, 2H), 2.29 – 1.88 (m, 4H), 1.83 – 1.59 (m, 5H), 1.40 – 0.99 (m, 9H), 0.92 (dd, *J* = 23.0, 7.8 Hz, 3H). ^{13}C NMR (101 MHz, $CDCl_3$) δ 171.94, 171.59, 162.68, 160.17, 160.15, 160.06, 154.84, 154.75, 153.98, 144.91, 144.86, 144.04, 144.01, 141.99, 141.88, 141.30, 131.81, 131.56, 129.92, 129.77, 129.73, 129.68, 129.62, 129.52, 128.78, 128.76, 128.72, 128.68, 125.46, 125.40, 125.37, 124.59, 124.50, 124.36, 122.80, 122.74, 121.87, 121.78, 120.69, 120.61, 120.53, 120.37, 109.94, 109.86, 109.82, 83.91, 83.70, 83.04, 82.66, 77.99, 77.89, 77.68, 77.57, 77.36, 64.75, 57.78, 57.71, 53.31, 50.86, 50.76, 47.90, 47.88, 47.65, 41.38, 41.32, 41.27, 40.12, 40.01, 38.83, 38.78, 38.48, 37.47, 37.39, 36.91, 36.86, 36.62, 35.91, 35.83, 32.79, 32.74, 32.65, 32.59, 31.58, 26.13, 26.07, 25.98, 15.81, 15.79, 14.89, 14.86. HRMS (ESI): calculated for $C_{33}H_{44}N_2O_6S$ $[M + H]^+$, 597.2998; found, 597.3000.

3-(Benzo[*d*][1,3]dioxol-5-yl)-*N*-(((4*R*,5*R*)-2-((*S*)-1-hydroxypropan-2-yl)-4-methyl-1,1-dioxido-8-(1-oxo-2,3,4,5-tetrahydro-1H-benzo[*c*]azepin-7-yl)-2,3,4,5-tetrahydrobenzo[*b*][1,4,5]oxathiazocin-5-yl)methyl)-*N*-methylpropiolamide (**40**)

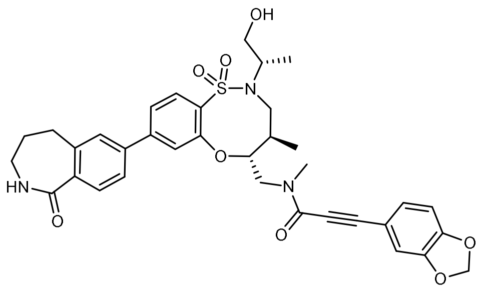

A 5 mL cone-shaped μW vial equipped with a magnetic stir bar was charged with (9H-fluoren-9-yl)methyl (((4*R*,5*R*)-8-bromo-2-((*S*)-1-hydroxypropan-2-yl)-4-methyl-1,1-dioxido-2,3,4,5-tetrahydrobenzo[*b*][1,4,5]oxathiazocin-5-yl)methyl)(methyl)carbamate (31.8 mg, 1 eq, 50.5 μmol), 7-(4,4,5,5-tetramethyl-1,3,2-dioxaborolan-2-yl)-2,3,4,5-tetrahydro-1*H*-benzo[*c*]azepin-1-one (22.9 mg, 95% *wt*, 75.8 μmol, 1.5 eq), XPhos Pd G3 (9.0 mg, 95% Wt, 0.010 mmol, 20 mol%), dry DMF (0.51 mL) and aq. K_3PO_4 (1.8 M, 84 μL, 0.15 mmol, 3.0 eq) at rt. The reaction mixture was purged with Ar for 3 min. The vial was then sealed, and the mixture was heated to 60 °C for 2 hr using μW irradiation. EtOAc was added and the mixture was filtered over cotton. The filtrate was concentrated under reduced pressure and

the obtained crude material was purified by flash chromatography (0–20% MeOH [with 1% NH_4OH] in CH_2Cl_2), affording 7-((4*R*,5*R*)-2-((*S*)-1-hydroxypropan-2-yl)-4-methyl-5-((methylamino)methyl)-1,1-dioxido-2,3,4,5-tetrahydrobenzo[*b*][1,4,5]oxathiazocin-8-yl)-2,3,4,5-tetrahydro-1H-benzo[*c*]azepin-1-one (4.8 mg, 9.8 µmol, 19%) as a cloudy oil.

A 2 dram scintillation vial equipped with a magnetic stir bar was charged with 7-((4*R*,5*R*)-2-((*S*)-1-hydroxypropan-2-yl)-4-methyl-5-((methylamino)methyl)-1,1-dioxido-2,3,4,5-tetrahydrobenzo[*b*][1,4,5]oxathiazocin-8-yl)-2,3,4,5-tetrahydro-1H-benzo[*c*]azepin-1-one (4.8 mg, 9.8 µmol, 1.0 eq) at rt. A solution of 3-(benzo[*d*][1,3]dioxol-5-yl)propiolic acid (2.2 mg, 12 µmol, 1.2 eq), COMU (5.2 mg, 12 µmol, 1.2 eq) and DIPEA (3.5 µL, 20 µmol, 2.0 eq) in dry DMF (0.24 mL) was then added and the resulting orange solution was stirred for 14 min. Water and EtOAc were added, and the aqueous phase was extracted twice with EtOAc. The organic layers were combined, dried over $MgSO_4$ and concentrated under reduced pressure. The material was then purified by preparative HPLC to afford **40**(4.0 mg, 6.1 µmol, 62%) as a pale yellow oil.

^{1}H NMR (400 MHz, CD_3OD) (~3:2 ratio of rotamers) δ 7.94 (d, *J* = 0.8 Hz, 0.4H), 7.92 (d, *J* = 0.8 Hz, 0.6H), 7.70 (d, *J* = 1.1 Hz, 1H), 7.65 (dd, *J* = 7.9, 1.8 Hz, 1H), 7.57 (t, *J* = 2.0 Hz, 0.6H), 7.55 (t, *J* = 2.0 Hz, 0.4H), 7.52 (d, *J* = 1.8 Hz, 0.6H), 7.47 (d, *J* = 8.0 Hz, 0.6H), 7.42 (d, *J* = 1.8 Hz, 0.6H), 7.38 (d, *J* = 1.8 Hz, 0.4H), 7.19 (dd, *J* = 8.1, 1.6 Hz, 0.4H), 7.06 (d, *J* = 1.6 Hz, 0.4H), 6.87 (d, *J* = 8.1 Hz, 0.4H), 6.83 (dd, *J* = 8.1, 1.6 Hz, 0.6H), 6.77 (d, *J* = 8.0 Hz, 0.6H), 6.68 (d, *J* = 1.5 Hz, 0.6H), 6.02 (s, 2H), 4.83 – 4.75 (m, 1H), 4.44 (ddd, *J* = 24.7, 14.2, 10.5 Hz, 1H), 3.87 – 3.56 (m, 5.4H), 3.37 – 3.33 (m, 0.6H), 3.16 (s, 1.75H), 3.10 (t, *J* = 6.6 Hz, 0.8H), 2.95 (t, *J* = 7.1 Hz, 0.8H), 2.89 (t, *J* = 6.6 Hz, 1.2H), 2.87 (s, 1.25H), 2.84 – 2.68 (m, 1.2H), 2.44 – 2.26 (m, 1H), 2.14 – 2.05 (m, 0.8H), 2.00 – 1.86 (m, 1.2H), 1.27 (dd, *J* = 6.7, 1.8 Hz, 3H), 1.00 (d, *J* = 6.9 Hz, 1.25H), 0.96 (d, *J* = 7.0 Hz, 1.75H). ^{13}C NMR (101 MHz, CD_3OD) δ 175.46, 175.07, 157.88, 157.62, 157.10, 156.90, 151.55, 151.51, 149.38, 149.20, 147.17, 146.81, 142.91, 142.58, 140.87, 140.78, 136.40, 136.23, 134.93, 134.92, 134.92, 131.16, 131.11, 130.26, 130.16, 129.19, 128.86, 128.34, 126.83, 126.53, 123.76, 123.64, 123.33, 122.89, 114.19, 113.72, 112.76, 112.72, 109.87, 109.74, 103.38, 93.99, 93.57, 84.80, 84.10, 81.35, 80.88, 65.57, 58.50, 58.38, 56.34, 51.14, 51.09, 40.36, 40.22, 38.16, 38.05, 37.47, 33.11, 31.68, 31.59, 31.23, 31.20, 15.87, 15.85, 14.95, 14.81. HRMS (ESI): calculated for $C_{35}H_{38}N_3O_8S$ $[M + H]^+$, 660.2374; found, 660.2408.

3-(Benzo[*d*][1,3]dioxol-5-yl)-*N*-(((4*R*,5*R*)-8-bromo-2-((*R*)-1-hydroxypropan-2-yl)-4-methyl-1,1-dioxido-2,3,4,5-tetrahydrobenzo[*b*][1,4,5]oxathiazocin-5-yl)methyl)-*N*-methylpropiolamide (**41**)

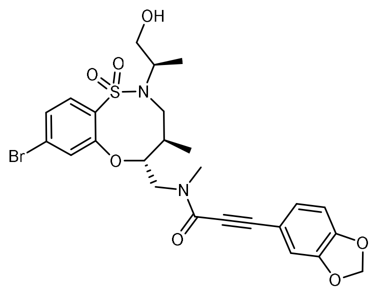

A 2 dram scintillation vial equipped with a magnetic stir bar was charged with (4*R*,5*R*)-8-bromo-2-((*R*)-1-hydroxypropan-2-yl)-4-methyl-5-((methylamino)methyl)-2,3,4,5-tetrahydrobenzo[*b*][1,4,5]oxathiazocine 1,1-dioxide [*cf.* synthesis of **35**] (8.0 mg, 1.0 eq, 20 µmol), dry DMF (0.20 mL), DIPEA (5.3 mg, 7.1 µL, 2.1 eq, 41 µmol), 3-(benzo[*d*][1,3]dioxol-5-yl)propiolic acid (4.8 mg, 1.3 eq, 25 µmol) and PyBOP (13 mg, 1.3 eq, 25 µmol) under N_2 atmosphere and at rt. The faint yellow reaction mixture was stirred for 35 min. The sample was then concentrated under reduced pressure and purified by preparative HPLC (optimal gradient: 30–70% MeCN in H_2O) to afford **41** (7.3 mg, 13 µmol, 64%) as a white solid.

^{1}H NMR (400 MHz, $CDCl_3$) (~3:1 ratio of rotamers) δ 7.76 (dd, *J* = 8.5, 2.2 Hz, 1H), 7.39 – 7.27 (m, 2H), 7.13 (ddd, *J* = 8.1, 3.9, 1.6 Hz, 1H), 7.00 (dd, *J* = 4.3, 1.6 Hz, 1H), 6.80 (dd, *J* = 8.0, 2.3 Hz, 1H), 6.02 (s, 2H), 4.79 – 4.61 (m, 1H), 4.45 (dd, *J* = 14.0, 9.9 Hz, 0.7H), 4.27 (dd, *J* = 14.6, 9.7 Hz, 0.3H), 3.89 – 3.46 (m, 5.3H), 3.18 (dd, *J* = 14.0, 2.7 Hz, 0.7H), 3.12 (s, 2.1H), 2.87 (s, 0.9H), 2.41 – 2.22 (m, 2H), 1.24 (t, *J* = 7.0 Hz, 3H), 0.96 (t, *J* = 7.6 Hz, 3H). ^{13}C NMR (101 MHz, $CDCl_3$) δ 156.16, 156.05, 155.71, 155.49, 149.75, 147.81, 147.73, 132.24, 131.83, 131.32, 131.16, 128.25, 128.00, 127.71, 127.52, 127.13, 127.08, 113.54, 113.51, 112.32, 112.16, 108.91, 108.83, 101.86, 101.83, 92.41, 91.60, 84.21, 83.64, 80.62, 80.23, 64.88, 56.58, 56.44, 54.89, 49.50, 48.78, 48.55, 37.34, 37.06, 36.85, 33.17, 15.95, 15.92, 15.30, 15.19. HRMS (ESI): calculated for $C_{25}H_{28}{}^{81}BrN_2O_7S$ $[M + H]^+$, 581.0775 found, 581.0788.

3-(Benzo[*d*][1,3]dioxol-5-yl)-*N*-(((4*R*,5*R*)-8-bromo-2-((*S*)-1-hydroxypropan-2-yl)-4-methyl-1,1-dioxido-2,3,4,5-tetrahydrobenzo[*b*][1,4,5]oxathiazocin-5-yl)methyl)-*N*-methylpropiolamide (**42**)

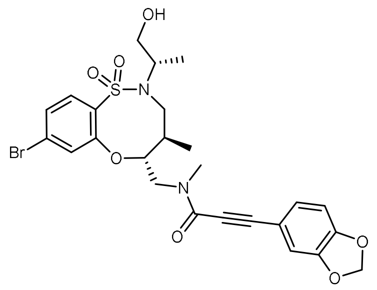

(9H-Fluoren-9-yl)methyl (((4*R*,5*R*)-8-bromo-2-((*S*)-1-hydroxypropan-2-yl)-4-methyl-1,1-dioxido-2,3,4,5-tetrahydrobenzo[*b*][1,4,5]oxathiazocin-5-yl)methyl)(methyl)carbamate (60 mg, 1 eq, 95 µmol) was charged to a 1-dram glass vial. DMF (0.56 mL) and piperidine (0.14 mL) were then added and the colorless solution was stirred at rt for 45 min. The reaction solution was concentrated under reduced

pressure, then azeotropically dried with heptane (4 x 50 mL) to provide the crude product as a white powder, a portion of which was carried on without further purification.

In a 1-dram vial containing a magnetic stir bar were combined 3-(benzo[*d*][1,3]dioxol-5-yl)propiolic acid (15 mg, 1.30 eq, 77 µmol) and PyBOP (40 mg, 1.30 eq, 77 µmol). DMF (0.2 mL) was added, giving a light brown solution. Addition of DIPEA (26 µL, 2.50 eq, 0.15 mmol) then afforded a thick, off-white slurry. After stirring for 5 min at rt, (4*R*,5*R*)-8-bromo-2-((*S*)-1-hydroxypropan-2-yl)-4-methyl-5-((methylamino)methyl)-2,3,4,5-tetrahydrobenzo[*b*][1,4,5]oxathiazocine 1,1-dioxide (24 mg, 1.00 eq, 59 µmol) was added and the walls of the flask were rinsed with DMF (0.8 mL), giving a brown solution. The reaction solution was stirred for 10 min before dilution with EtOAc and water, giving a biphasic mixture. Layers were separated, and the aqueous phase was extracted with EtOAc (3 x 1 mL); the combined organics were then dried over Na_2SO_4, decanted and concentrated under reduced pressure to reveal a yellow oil. Purification by flash chromatography (0–100% MeOH [with 1% NH_4OH] in CH_2Cl_2, 4 g silica column), followed by (0–100% EtOAc in hexanes) followed by preparative HPLC afforded **42** (8.7 mg, 15 µmol, 26%) as a white solid.

^{1}H NMR (400 MHz, $CDCl_3$) (~2:1 ratio of rotamers) δ 7.77 (dd, *J* = 8.8, 2.9 Hz, 1H), 7.40 – 7.27 (m, 2H), 7.13 (ddd, *J* = 8.1, 2.7, 1.6 Hz, 1H), 7.00 (dd, *J* = 5.7, 1.6 Hz, 1H), 6.80 (dd, *J* = 8.1, 5.5 Hz, 1H), 6.02 (s, 2H), 4.82 – 4.74 (m, 1H), 4.54 (dd, *J* = 14.0, 10.2 Hz, 0.7H), 4.36 (dd, *J* = 14.6, 10.0 Hz, 0.3H), 3.97 – 3.81 (m, 2H), 3.67 – 3.36 (m, 3.3H), 3.13 (dd, *J* = 14.1, 2.8 Hz, 0.7H), 3.10 (s, 2H), 2.83 (s, 1H), 2.33 – 2.18 (m, 1H), 1.22 (dd, *J* = 6.8, 5.5 Hz, 3H), 0.97 (dd, *J* = 13.8, 6.9 Hz, 3H). ^{13}C NMR (101 MHz, $CDCl_3$) δ 156.24, 155.55, 154.72, 154.32, 149.59, 147.52, 147.46, 133.11, 132.86, 130.42, 128.11, 128.05, 127.95, 127.77, 127.48, 127.01, 126.89, 113.02, 112.92, 112.05, 111.95, 108.70, 108.61, 101.63, 92.77, 92.09, 83.65, 82.92, 79.96, 79.73, 77.20, 64.36, 64.24, 57.53, 57.41, 54.59, 48.38, 48.27, 36.69, 36.59, 36.47, 32.72, 15.39, 14.65, 14.43. HRMS (ESI): calculated for $C_{25}H_{28}{}^{81}BrN_2O_7S$ $[M + H]^+$, 581.0775 found, 581.0797.

3-(Benzo[*d*][1,3]dioxol-5-yl)-1-((8*R*,9*R*,10*S*)-9-(4-bromophenyl)-10-(hydroxymethyl)-1,6-diazabicyclo[6.2.0]decan-6-yl)prop-2-yn-1-one (**43**)

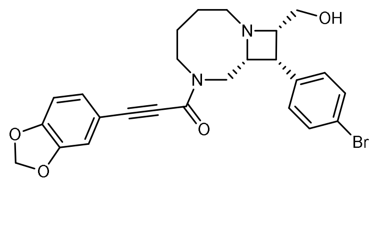

A 1-dram scintillation vial equipped with a magnetic stir bar was charged with ((8*R*,9*R*,10*S*)-9-(4-bromophenyl)-6-((4-nitrophenyl)sulfonyl)-1,6-diazabicyclo[6.2.0]decan-10-yl)methanol (8.5 mg, 1 eq, 17 µmol). Dry DMF (0.28 mL), K_2CO_3 (12 mg, 5.0 eq, 83 µmol) and 4-methoxythiophenol (9.6 mg, 8.4 µL, 97% Wt, 4.0 eq, 67 µmol) were successively added and the yellow suspension was heated to 50 °C and

stirred for 50 min. The obtained yellow/orange suspension was concentrated under reduced pressure. EtOAc (30 mL) and 0.3 M aq. HCl (30 mL) were added, phases separated, and aqueous portion was extracted twice with EtOAc (2 x 30 mL). The aqueous layer was then basified with 40% aq. KOH solution and extracted EtOAc (3 x 30 mL). The organic layers were combined, dried over Na_2SO_4, filtered and solvent was removed under reduced pressure to afford crude ((8*R*,9*R*,10*S*)-9-(4-bromophenyl)-1,6-diazabicyclo[6.2.0]decan-10-yl)methanol as a faintly yellow, grey solid. To this solid was added dry DMF (0.34 mL), DIPEA (4.5 mg, 6.1 µL, 2.1 eq, 35 µmol), 3-(benzo[*d*][1,3]dioxol-5-yl)propiolic acid (3.5 mg, 1.1 eq, 18 µmol) and COMU (8.1 mg, 97%wt, 1.1 eq, 18 µmol) at rt. The orange/brown solution was stirred for 50 min, then a drop of water and DMSO were added, and the mixture was filtered prior to purification by preparative HPLC to afford **43** (3.64 mg, 17 µmol, 44%) as a faint yellow oil.

^{1}H NMR (400 MHz, CD_3OD) (~55:45 ratio of rotamers) δ 7.53 – 7.40 (m, 4H), 7.12 (dd, *J* = 8.0, 1.6 Hz, 0.45H), 7.03 (dd, *J* = 8.0, 1.6 Hz, 0.55H), 7.00 (d, *J* = 1.6 Hz, 0.45H), 6.91 (d, *J* = 1.6 Hz, 0.55H), 6.88 (dd, *J* = 8.0, 5.3 Hz, 1H), 6.04 (d, *J* = 6.3 Hz, 2H), 4.30 (ddd, *J* = 14.9, 6.7, 3.4 Hz, 0.45H), 4.03 (ddd, *J* = 13.8, 6.5, 4.0 Hz, 0.55H), 3.76 – 3.54 (m, 3.75H), 3.50 – 3.32 (m, 5.25H), 3.22 – 3.09 (m, 1.55H), 3.01 – 2.93 (m, 0.45H), 2.41 – 2.28 (m, 1H), 2.11 (ddd, *J* = 14.6, 7.0, 3.5 Hz, 0.45H), 1.94 – 1.57 (m, 3.55H). ^{13}C NMR (101 MHz, CD_3OD) δ 156.54, 156.19, 151.47, 151.46, 151.45, 149.38, 136.79, 136.71, 133.83, 132.25, 132.09, 129.16, 129.01, 121.95, 121.84, 114.26, 114.16, 112.76, 112.69, 109.86, 109.84, 103.38, 103.36, 93.03, 92.57, 81.13, 80.86, 69.86, 69.56, 68.07, 67.19, 62.01, 61.91, 60.06, 59.72, 54.14, 52.39, 50.39, 43.97, 43.68, 29.91, 28.29, 28.03, 27.58. HRMS (ESI): calculated for $C_{25}H_{26}{}^{81}BrN_2O_4$ $[M + H]^+$, 499.1050 found, 499.1056.

3-(Benzo[*d*][1,3]dioxol-5-yl)-1-((8*S*,9*S*,10*S*)-9-(4-bromophenyl)-10-(hydroxymethyl)-1,6-diazabicyclo[6.2.0]decan-6-yl)prop-2-yn-1-one (**44**)

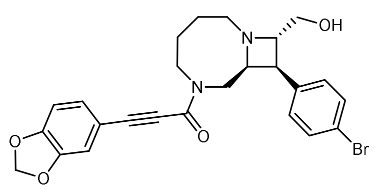

A 20 mL scintillation vial equipped with a magnetic stir bar was charged with ((8*S*,9*S*,10*S*)-9-(4-bromophenyl)-6-((2-nitrophenyl)sulfonyl)-1,6-diazabicyclo[6.2.0]decan-10-yl)methanol (49 mg, 1.0 eq, 96 µmol), dry DMF (1.6 mL), 4-methoxy-benzenethiol (54 mg, 47 µL, 4 eq, 0.38 mmol) and K_2CO_3 (66 mg, 5 eq, 0.48 mmol) at rt. The suspension was heated to 50 °C and stirred for 80 min. The obtained yellow suspension was concentrated under reduced pressure to afford a yellow solid. EtOAc (30 mL) and 0.3 M aq. HCl (30 mL) were added, phases separated, and the aqueous phase was extracted twice with EtOAc. The aqueous portion then had pH adjusted to 13–14 with 40% aq. KOH solution and was extracted three times with EtOAc. The organic layers were combined, dried over Na_2SO_4 and concentrated under reduced pressure to afford crude ((8*S*,9*S*,10*S*)-9-(4-bromophenyl)-1,6-diazabicyclo[6.2.0]decan-10-

yl)methanol (27 mg, 83 µmol) as a pale-yellow solid. To this residue was added dry DMF (1.6 mL), 3-(benzo[*d*][1,3]dioxol-5-yl)propiolic acid (19 mg, 1.2 eq, 0.10 mmol), DIPEA (21 mg, 2 eq, 0.17 mmol) and COMU (39 mg, 1.1 eq, 91 µmol) at rt and the mixture was stirred for 30 min. The mixture was concentrated under reduced pressure, then purified by preparative HPLC to afford **44** (10 mg, 20 µmol, 24 %) as a colorless oil.

^{1}H NMR (400 MHz, CD_3OD) (~55:45 ratio of rotamers) δ 7.54 (dd, *J* = 33.9, 8.5 Hz, 2H), 7.34 (t, *J* = 8.1 Hz, 2H), 7.13 (dd, *J* = 8.1, 1.6 Hz, 0.55H), 7.00 (d, *J* = 1.6 Hz, 0.55H), 6.94 – 6.89 (m, 0.9H), 6.87 (d, *J* = 8.0 Hz, 0.55H), 6.83 (t, *J* = 1.0 Hz, 0.45H), 6.07 (s, 0.9H), 6.03 (s, 1.1H), 4.68 – 4.55 (m, 1.55H), 4.47 (dt, *J* = 8.0, 4.1 Hz, 0.45H), 4.30 (dt, *J* = 15.5, 4.8 Hz, 0.56H), 4.23 – 3.66 (m, 5H), 3.57 – 3.25 (m, 3H), 3.19 (ddd, *J* = 14.4, 8.2, 3.6 Hz, 0.45H), 2.11 – 1.60 (m, 4H). ^{13}C NMR (101 MHz, CD_3OD) δ 156.77, 156.20, 151.66, 151.58, 149.38, 135.07, 134.37, 133.25, 133.15, 131.68, 131.48, 129.39, 129.03, 123.05, 122.98, 113.84, 113.71, 112.80, 112.66, 109.89, 109.88, 103.46, 103.41, 93.31, 93.25, 80.61, 80.34, 71.21, 70.87, 68.79, 67.42, 60.22, 59.82, 50.24, 46.03, 45.16, 40.67, 40.07, 27.55, 26.11, 24.86, 23.46. HRMS (ESI): calculated for $C_{25}H_{26}{}^{81}BrN_2O_4$ [M + H]$^+$, 499.1050 found, 499.1059.

(2*R*,4*R*)-*N*-Methyl-1-((4-sulfamoylphenyl)sulfonyl)-4-(3-(1,2,3,6-tetrahydropyridin-4-yl)phenoxy)pyrrolidine-2-carboxamide (**45**)

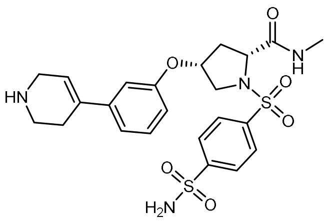

(2*R*,4*R*)-4-(3-Iodophenoxy)-2-(methoxycarbonyl)pyrrolidin-1-ium 2,2,2-trifluoroacetate (43 mg, 93 µmol, 1 eq) and 4-sulfamoylbenzenesulfonyl chloride (2.9 mg, 0.11 mmol, 1.2 eq) were dissolved in anhydrous CH_2Cl_2 (500 µL, 0.2 M) in a 1-dram vial. NEt_3 (39 µL, 0.28 mmol, 3 eq) was added and the reaction was allowed to stir at rt for 4 h. The crude reaction mixture was washed with 1:1 mixture of water:brine (1x), the organic phase was concentrated under reduced pressure, and the crude product was dissolved in THF (2 mL), followed by addition of 1 N aq. LiOH solution (1 mL). The reaction was stirred overnight, then was acidified to pH 2 with dilute aq. HCl and extracted with CH_2Cl_2 five times. The pooled organics were dried over anhydrous $MgSO_4$ and concentrated under reduced pressure. The residue was used for the next step without further purification.

To a 1-dram vial containing crude (2*R*,4*R*)-4-(3-iodophenoxy)-1-((4-sulfamoylphenyl)sulfonyl)pyrrolidine-2-carboxylic acid (26 mg, 47 µmol, 1 eq), NH_3MeCl (6.4 mg, 94 µmol, 2 eq) and HATU (27 mg, 71 µmol, 1.5 eq) was added anhydrous DMF (450 µL), followed by DIPEA (41 µL, 0.24 mmol, 5 eq). The resulting yellow solution was stirred overnight at rt. The solution was diluted with CH_2Cl_2 and washed with water (3 x 1 mL), followed by brine (1 x 1 mL). The organic phase was dried over anhydrous $MgSO_4$ and

concentrated under reduced pressure. The resulting crude mixture was added to a 2 mL μW vial and 4-(4,4,5,5-tetramethyl-1,3,2-dioxaborolan-2-yl)-1,2,3,6-tetrahydropyridine (15 mg, 71 μmol, 1.5 eq) and XPhos-Pd-G2 (7.4 mg, 9.4 μmol, 0.2 eq) was added. The vial was capped with a septum and purged 3 times with N_2 gas. Anhydrous DMF (0.5 mL) was added, followed by 1.8 M aq. K_3PO_4 (0.16 mL, 6 eq). The reaction was stirred at 60 °C for 2 hr under μW irradiation. The resulting dark brown suspension was diluted with DMSO, filtered through a syringe filter, and purified by preparative to afford **45** (3.9 mg, 7.5 μmol, 16%) as a white solid.

^{1}H NMR (400 MHz, CD_3OD) δ 8.55 (s, 1H), 8.12 (dd, *J* = 16.7, 2.6 Hz, 4H), 7.27 (t, *J* = 8.0 Hz, 1H), 7.07 (dd, *J* = 7.7, 1.7 Hz, 1H), 6.87 (t, *J* = 2.1 Hz, 1H), 6.80 (dd, *J* = 8.2, 2.4 Hz, 1H), 6.13 (t, *J* = 1.8 Hz, 1H), 4.91 (t, *J* = 4.1 Hz, 1H), 4.27 (dd, *J* = 9.9, 1.7 Hz, 1H), 3.87 – 3.73 (m, 3H), 3.56 (dd, *J* = 11.5, 4.5 Hz, 1H), 3.41 (t, *J* = 6.1 Hz, 2H), 2.83 (s, 3H), 2.77 – 2.69 (m, 2H), 2.37 (d, *J* = 14.1 Hz, 1H), 1.91 (ddd, *J* = 14.2, 9.9, 4.2 Hz, 1H). ^{13}C NMR (101 MHz, CD_3OD) δ 174.24, 158.09, 149.94, 142.38, 140.35, 136.73, 130.90, 129.86, 128.42, 119.53, 118.19, 116.10, 114.10, 76.88, 62.96, 55.81, 43.63, 42.31, 36.61, 26.76, 25.23. HRMS (ESI): calculated for $C_{23}H_{29}N_4O_6S_2$ $[M + H]^+$, 521.1523 found, 521.1536.

(*R*)-5-((2-Acetyl-8-(3'-((dimethylamino)methyl)-4'-fluoro-[1,1'-biphenyl]-4-yl)-2,6-diazaspiro[3.4]octan-6-yl)methyl)furan-2-sulfonamide (**46**)

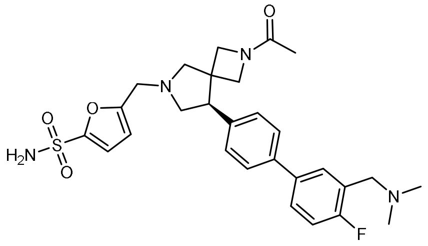

tert-Butyl (*R*)-8-(4-iodophenyl)-2-((2-nitrophenyl)sulfonyl)-2,6-diazaspiro[3.4]octane-6-carboxylate (30 mg, 55 μmol, 1 eq) and K_2CO_3 (14 mg, 0.1 mmol, 2 eq) was suspended in anhydrous DMF (400 μL). The resulting suspension was cooled to 0 °C in an ice bath, followed by addition of thiophenol (5.7 μL, 55 μmol, 1.1 eq). The resulting mixture was stirred for 1 hr at 0 °C, then diluted with EtOAc and filtered through a pad of celite. The filtrate was concentrated under reduced pressure and used for the next step without further purification.

The crude mixture from previous reaction (55 μmol, 1 eq) was dissolved in CH_2Cl_2 (0.5 mL), followed by addition of Ac_2O (10 μL, 0.11 mmol, 2 eq) and NEt_3 (15.0 μL, 0.11 μmol, 2 eq). The solution was stirred at rt for 2 hr and concentrated under reduced pressure. This crude mixture was added to a 2 mL μW vial and (3-((dimethylamino)methyl)-4-fluorophenyl)boronic acid (16 mg, 83 μmol, 1.5 eq) and XPhos-Pd-G2 (8.7 mg, 11 μmol, 0.2 eq) were added. The vial was capped with a septum and purged 3 times with N_2 gas. Anhydrous DMF (550 μL) was added, followed by 1.8 M aq. K_3PO_4 (0.18 mL, 330 μmol, 6 eq). The reaction was stirred at 60 °C for 120 min under μW irradiation, then the solution was washed with brine

twice and product was purified by flash chromatography (MeOH [with 1% NH_4OH] in CH_2Cl_2) to afford *tert*-butyl (*R*)-2-acetyl-8-(3'-((dimethylamino)methyl)-4'-fluoro-[1,1'-biphenyl]-4-yl)-2,6-diazaspiro[3.4]octane-6-carboxylate (15 mg, 31 µmol, 57% over 3 steps).

tert-butyl (*R*)-2-acetyl-8-(3'-((dimethylamino)methyl)-4'-fluoro-[1,1'-biphenyl]-4-yl)-2,6-diazaspiro[3.4]octane-6-carboxylate (15 mg, 31 µmol, 1 eq) was dissolved in CH_2Cl_2 (0.5 mL), and TFA (100 µL) was added. After 1.5 hr at rt the solution was concentrated under reduced pressure and to the resulting crude residue was added 5-formylfuran-2-sulfonamide (6.0 mg, 34 µmol, 1.1 eq) and sodium acetoxyborohydride (13 mg, 62 µmol, 2 eq). Anhydrous DCE (300 µL) and anhydrous DMF (100 µL) were added, followed by NEt_3 (4.3 µL, 31 µmol, 1 eq). The reaction was stirred at rt for 1 hr and concentrated under reduced pressure. The residue was diluted with DMSO and purified directly by preparative HPLC to afford (*R*)-5-((2-acetyl-8-(3'-((dimethylamino)methyl)-4'-fluoro-[1,1'-biphenyl]-4-yl)-2,6-diazaspiro[3.4]octan-6-yl)methyl)furan-2-sulfonamide (2.2 mg, 4 µmol, 13% over 2 steps).

^{1}H NMR (400 MHz, CD_3OD) (~1:1 ratio of rotamers) δ 7.78 (ddq, *J* = 7.5, 4.9, 2.6 Hz, 2H), 7.72 – 7.58 (m, 2H), 7.45 – 7.25 (m, 3H), 6.93 (d, *J* = 3.4 Hz, 1H), 6.49 (d, *J* = 3.4 Hz, 1H), 4.29 (d, *J* = 8.8 Hz, 2H), 4.21 (d, *J* = 2.2 Hz, 1H), 3.98 (s, 1H), 3.93 – 3.81 (m, 2.5H), 3.67 (d, *J* = 9.2 Hz, 0.5H), 3.58 (d, *J* = 10.4 Hz, 0.5H), 3.52 – 3.44 (m, 1.5H), 3.30 – 3.22 (m, 1.5H), 3.19 (d, *J* = 9.8 Hz, 0.5H), 3.13 (d, *J* = 9.8 Hz, 0.5H), 3.07 (d, *J* = 9.9 Hz, 0.5H), 2.93 (ddd, *J* = 20.5, 9.7, 7.6 Hz, 1H), 2.80 (d, *J* = 6.4 Hz, 6H), 1.80 (s, 1.6H), 1.61 (s, 1.4H). ^{13}C NMR (101 MHz, CD_3OD) δ 155.42, 151.27, 128.83, 126.95, 116.18, 115.96, 113.86, 109.56, 64.93, 64.66, 62.52, 59.90, 59.13, 54.52, 54.27, 51.61, 51.49, 50.36, 43.53, 43.35, 42.28, 42.23, 17.07. LRMS (ESI): calculated for $C_{28}H_{34}FN_4O_4S$ [M + H]$^+$, 541.23 found, 541.26.

(2*S*,3*S*)-3-(3'-(Ethylcarbamoyl)-[1,1'-biphenyl]-4-yl)-*N*-methyl-1-(4-sulfamoylbenzyl)pyrrolidine-2-carboxamide (**47**)

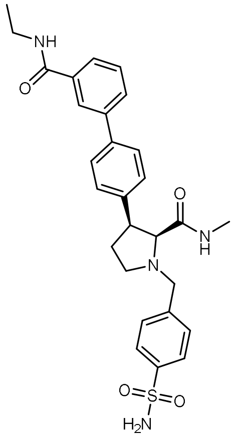

A 20 mL vial was charged with (2S,3S)-1-(tert-butoxycarbonyl)-3-(4-iodophenyl)pyrrolidine-2-carboxylic acid (20.9 mg, 1.00 eq, 50.0 µmol) and HATU (22.8 mg, 1.20 eq, 60.0 µmol). DMF (5 mL) was added followed by NEt_3 (15.2 mg, 20.9 µL, 3.00 eq, 150 µmol) and solution was stirred for 15 min. Methanamine,

HCl (6.75 mg, 2.00 eq, 100 µmol) was then added as a solid and reaction was further stirred for 3 hr. The solution was diluted with CH_2Cl_2 (50 mL), washed with 1 M aq. citric acid (2 x 50 mL), dried over $MgSO_4$, filtered, and solvent removed under reduced pressure. *tert*-Butyl (2*S*,3*S*)-3-(4-iodophenyl)-2-(methylcarbamoyl)pyrrolidine-1-carboxylate (89%) was isolated after flash chromatography (0–20% MeOH in CH_2Cl_2) as a yellow oil.

tert-Butyl (2*S*,3*S*)-3-(4-iodophenyl)-2-(methylcarbamoyl)pyrrolidine-1-carboxylate (21.5 mg, 1.00 eq, 50.0 µmol) was dissolved by CH_2Cl_2 (5 mL). TFA (114 mg, 77.0 µL, 20.0 eq, 1.00 mmol) was added via syringe and reaction stirred for 30 min. Volatiles were removed under reduced pressure, the residue was dissolved in CH_2Cl_2 (50 mL) and washed with 1M K_2CO_3 (3 x 50 mL). Aqueous layers were combined and extracted with CH_2Cl_2 (3 x 100 mL). Organic portions were combined, dried over Na_2SO_4, filtered, and solvent was removed under reduced pressure to afford crude (2*S*,3*S*)-3-(4-iodophenyl)-*N*-methylpyrrolidine-2-carboxamide, which was used directly in the next step.

Crude (2*S*,3*S*)-3-(4-iodophenyl)-*N*-methylpyrrolidine-2-carboxamide (30.4 mg, 1.00 eq, 92.0 µmol), 4-formylbenzenesulfonamide (25.6 mg, 1.50 eq, 138 µmol), and sodium triacetoxyborohydride (97.5 mg, 5.00 eq, 460 µmol) were stirred in DCE (8 mL) for 1.5 hr at rt. The reaction mixture was diluted with CH_2Cl_2 (50 mL) and washed with saturated aq. $NaHCO_3$ (3 x 50 mL). Aqueous layers were combined and extracted with CH_2Cl_2 (3 x 100 mL). Organic portions were combined, dried over $MgSO_4$, filtered, and volatiles removed under reduced pressure prior to purification by preparative HPLC to afford (2*S*,3*S*)-3-(4-iodophenyl)-*N*-methyl-1-(4-sulfamoylbenzyl)pyrrolidine-2-carboxamide (22 mg, 43 µmol, 47% over two steps) as an off-white solid.

A 5 mL µW vial was charged with (2*S*,3*S*)-3-(4-iodophenyl)-*N*-methyl-1-(4-sulfamoylbenzyl)pyrrolidine-2-carboxamide (12.5 mg, 1.00 eq, 25.0 µmol), (3-(ethylcarbamoyl)phenyl)boronic acid (7.24 mg, 1.50 eq, 37.5 µmol), and Palladium-Xphos (3.93 mg, 0.20 eq, 5.00 µmol). DMF was added followed by 1.8 M aq. K_3PO_4 (15.9 mg, 41.7 µL, 3.00 eq, 75.0 µmol). The vial was capped and sparged with N_2 for 5 min before heating at 60 °C for 1 hr under µW irradiation. After cooing the solution was diluted with 1:4 MeOH:CH_2Cl_2 (20 mL), filtered through celite and rinsed with flushing 1:4 MeOH:CH_2Cl_2 (5 x 50 mL). Volatiles were removed under reduced pressure and crude product was purified by preparative HPLC to afford **47** (7.4 mg, 14.3 µmol, 57%).

^1H NMR (400 MHz, DMSO) δ 8.57 (t, *J* = 5.5 Hz, 1H), 8.10 (t, *J* = 1.8 Hz, 1H), 7.83 – 7.77 (m, 4H), 7.61 (dd, *J* = 8.4, 2.0 Hz, 4H), 7.59 – 7.51 (m, 2H), 7.34 (d, *J* = 7.8 Hz, 4H), 3.90 (d, *J* = 13.7 Hz, 1H), 3.70 – 3.57 (m, 2H), 3.46 (d, *J* = 9.3 Hz, 1H), 3.10 (ddd, *J* = 9.0, 6.5, 3.2 Hz, 1H), 2.58 – 2.52 (m, 1H), 2.33 (d, *J* = 4.7 Hz, 3H), 2.16 (tq, *J* = 7.9, 3.3 Hz, 2H), 1.16 (t, *J* = 7.2 Hz, 3H). ^{13}C NMR (101 MHz, DMSO) δ 170.79, 165.82, 142.88, 142.75, 140.64, 139.96, 137.40, 135.30, 129.08, 128.99, 128.94, 128.91, 126.09, 125.95, 125.53, 124.99, 71.70, 57.67, 52.25, 46.85, 34.07, 31.19, 25.18, 14.81. HRMS (ESI): calculated for $C_{28}H_{33}N_4O_4S$ $[M + H]^+$, 522.2217 found, 521.2228.

(2*R*,3*S*)-3-(3'-Ethoxy-[1,1'-biphenyl]-3-yl)-*N*-methyl-1-((5-sulfamoylfuran-3-yl)methyl)pyrrolidine-2-carboxamide (**48**)

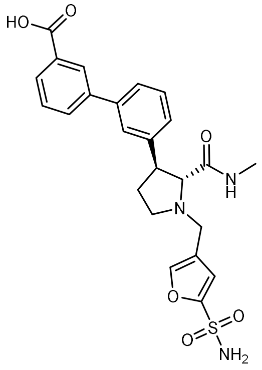

A 20 mL vial was charged with (2*R*,3*S*)-1-(*tert*-butoxycarbonyl)-3-(3-iodophenyl)pyrrolidine-2-carboxylic acid (83.4 mg, 1 eq, 200 µmol) and HATU (91.3 mg, 1.2 eq, 240 µmol). DMF was added followed by NEt_3 (101 mg, 139 µL, 5 eq, 1.00 mmol) and the solution was stirred for 15 min. Methanamine, HCl (40.5 mg, 3 eq, 600 µmol) was then added as a solid and reaction was further stirred 3 hr. Solvent was removed under reduced pressure, residue was dissolved as far as possible in CH_2Cl_2 (100 mL), washed with 1 M aq. citric acid (2 x 50 mL), dried over $MgSO_4$, filtered, and solvent removed under reduced pressure. *tert*-Butyl (2*R*,3*S*)-3-(3-iodophenyl)-2-(methylcarbamoyl)pyrrolidine-1-carboxylate (63 mg, 146 µmol, 73%) was obtained as a yellow oil after flash chromatography (0–20% MeOH in CH_2Cl_2).

A 20 mL vial was charged with *tert*-butyl (2*S*,3*S*)-3-(4-iodophenyl)-2-(methylcarbamoyl)pyrrolidine-1-carboxylate (22 mg, 1 eq, 50 µmol) followed by CH_2Cl_2 . TFA (0.11 g, 77 µL, 20 eq, 1.0 mmol) was added by syringe and the mixture was stirred for 30 min. Solvent and TFA were then removed under reduced pressure. Crude residue was taken up in CH_2Cl_2 (50 mL) and washed 1M K_2CO_3 (3 x 50 mL). Combined aqueous layers were extracted with CH_2Cl_2 (3 x 100 mL). Organic portions were combined and dried over Na_2SO_4, filtered, and (2*R*,3*S*)-3-(3-iodophenyl)-*N*-methylpyrrolidine-2-carboxamide was isolated under reduced pressure in sufficient purity to carry to next step.

A 20 mL vial was charged with (2*R*,3*S*)-3-(3-iodophenyl)-*N*-methylpyrrolidine-2-carboxamide (132 mg, 1.00 eq, 400 µmol), 4-formylfuran-2-sulfonamide (105 mg, 1.50 eq, 600 µmol), and sodium triacetoxyborohydride (424 mg, 5.00 eq, 2.00 mmol). DCE (10 mL) was added, and the reaction was stirred for 1.5 hr at rt. The reaction solution was diluted with CH_2Cl_2 (50 mL) and washed with saturated $NaHCO_3$ (3 x 50 mL). Combined aqueous portions were extracted with CH_2Cl_2 (3 x 100 mL). Then combined organics were dried over $MgSO_4$, filtered, and solvent removed under reduced pressure. (2*R*,3*S*)-3-(3-Iodophenyl)-*N*-methyl-1-((5-sulfamoylfuran-3-yl)methyl)pyrrolidine-2-carboxamide (145 mg, 296 µmol, 74% over two steps) was isolated as a white solid following purification by preparative HPLC.

A 5 mL µW vial was charged with (2*R*,3*S*)-3-(3-iodophenyl)-*N*-methyl-1-((5-sulfamoylfuran-3-yl)methyl)pyrrolidine-2-carboxamide (7.3 mg, 1.00 eq, 15 µmol), 3-boronobenzoic acid (3.1 mg, 1.25 eq, 19

μmol), and Palladium-Xphos (2.4 mg, 0.20 eq, 3.0 μmol). DMF (2.5 mL) was added followed by 1.8 M aq. K_3PO_4 (16 mg, 42 μL, 5.00 eq, 75 μmol). The vial was capped and sparged with N_2 for 5 min, then the solution was heated at 60 °C for 1 hr under μW irradiation. The solution was diluted with 1:4 MeOH:CH_2Cl_2 (20 mL), filtered through celite and rinsed with 1:4 MeOH:CH_2Cl_2 (5 x 50 mL). Solvents were removed under reduced pressure and product was purified by preparative HPLC to afford **48** (3.3 mg, 6.9 μmol, 46%) as an off-white solid.

^{1}H NMR (400 MHz, DMSO) δ 8.18 (t, *J* = 1.8 Hz, 1H), 7.95 (dt, *J* = 7.7, 1.4 Hz, 1H), 7.92 – 7.84 (m, 3H), 7.70 (s, 2H), 7.59 (t, *J* = 7.7 Hz, 1H), 7.56 – 7.51 (m, 2H), 7.43 (t, *J* = 7.6 Hz, 1H), 7.28 (dt, *J* = 7.6, 1.4 Hz, 1H), 7.10 (d, *J* = 1.0 Hz, 1H), 3.63 (dd, *J* = 13.7, 1.2 Hz, 1H), 3.47 (d, *J* = 13.7 Hz, 1H), 3.40 (dt, *J* = 9.2, 6.8 Hz, 1H), 3.19 (d, *J* = 7.4 Hz, 1H), 3.09 (ddd, *J* = 9.3, 7.6, 4.4 Hz, 1H), 2.70 – 2.59 (m, 4H), 2.35 – 2.22 (m, 1H), 1.97 – 1.87 (m, 1H). ^{13}C NMR (101 MHz, DMSO) δ 172.38, 167.55, 151.98, 144.59, 143.36, 140.45, 139.52, 132.69, 130.70, 129.14, 128.23, 127.35, 126.81, 126.06, 124.83, 123.74, 114.24, 74.64, 52.10, 49.22, 47.80, 32.14, 25.57. HRMS (ESI): calculated for $C_{24}H_{26}N_3O_6S$ [M + H]$^+$, 484.1537 found, 484.1552.

(2*R*,3*S*)-3-(3'-Ethoxy-[1,1'-biphenyl]-4-yl)-*N*-methyl-1-((5-sulfamoylfuran-3-yl)methyl)pyrrolidine-2-carboxamide (**49**)

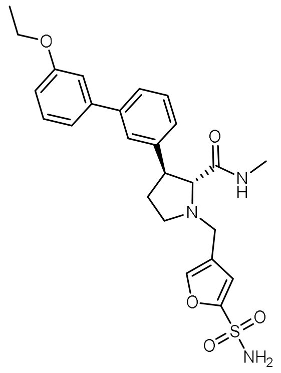

A 5 mL μW vial was charged with (2*R*,3*S*)-3-(3-iodophenyl)-*N*-methyl-1-((5-sulfamoylfuran-3-yl)methyl)pyrrolidine-2-carboxamide [*cf.* synthesis of **48**] (7.3 mg, 1.00 eq, 15 μmol), (3-ethoxyphenyl)boronic acid (3.1 mg, 1.25 eq, 19 μmol), and Palladium-Xphos (2.4 mg, 0.20 eq, 3.0 μmol). DMF (2.5 mL) was added followed by 1.8 M aq. K_3PO_4 (16 mg, 42 μL, 5.00 eq, 75 μmol). The vial was capped and sparged with N_2 for 5 min. Solution was placed in a μwave reactor and μwaved at 60 °C for 1 hour. Solution was diluted with 1:4 MeOH:CH_2Cl_2 (20 mL), filtered through celite and rinsed with flushing 1:4 MeOH:CH_2Cl_2 (5 x 50 mL). Solvents were removed under reduced pressure, and product was purified by preparative HPLC to **49** (3.7 mg, 7.7 μmol, 51%) as an off-white solid.

^{1}H NMR (400 MHz, DMSO) δ 7.93 – 7.83 (m, 2H), 7.69 (s, 2H), 7.53 – 7.47 (m, 2H), 7.38 (td, *J* = 7.8, 3.6 Hz, 2H), 7.24 (dt, *J* = 7.6, 1.5 Hz, 1H), 7.20 (dt, *J* = 7.9, 1.1 Hz, 1H), 7.16 (t, *J* = 2.1 Hz, 1H), 7.10 (d, *J* = 1.0 Hz, 1H), 6.94 (ddd, *J* = 8.3, 2.5, 0.9 Hz, 1H), 4.11 (q, *J* = 7.0 Hz, 2H), 3.63 (dd, *J* = 13.6, 1.2 Hz, 1H),

3.46 (d, *J* = 13.7 Hz, 1H), 3.37 (dt, *J* = 9.3, 6.7 Hz, 1H), 3.17 (d, *J* = 7.4 Hz, 1H), 3.09 (ddd, *J* = 9.2, 7.6, 4.3 Hz, 1H), 2.70 – 2.63 (m, 1H), 2.62 (d, *J* = 4.8 Hz, 3H), 2.27 (ddt, *J* = 12.5, 9.2, 7.6 Hz, 1H), 1.96 – 1.86 (m, 1H), 1.37 (t, *J* = 6.9 Hz, 3H). ^{13}C NMR (101 MHz, DMSO) δ 172.41, 158.99, 151.96, 144.37, 143.34, 141.82, 140.15, 129.97, 128.90, 126.55, 126.08, 124.80, 123.72, 119.01, 114.23, 113.31, 112.85, 74.62, 63.03, 52.08, 49.21, 47.80, 32.15, 25.55, 14.69. HRMS (ESI): calculated for $C_{25}H_{30}N_3O_5S$ $[M + H]^+$, 484.1901 found, 484.1957.

(2*S*,3*R*)-3-(3'-Ethoxy-[1,1'-biphenyl]-4-yl)-*N*-methyl-1-((5-sulfamoylfuran-3-yl)methyl)azetidine-2-carboxamide (**50**)

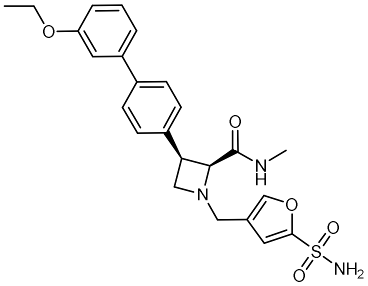

(2*S*,3*R*)-3-(3'-Ethoxy-[1,1'-biphenyl]-4-yl)-*N*-methyl-1-((5-sulfamoylfuran-3-yl)methyl)azetidine-2-carboxamide (7.2 mg, 15.4 µmol, 77%, final step) was prepared as a white solid following an analogous synthesis to **48**. Intermediate yields are provided below:

tert-Butyl (2*S*,3*R*)-3-(4-iodophenyl)-2-(methylcarbamoyl)azetidine-1-carboxylate (88%) as yellow oil. (2*S*,3*R*)-3-(4-Iodophenyl)-*N*-methyl-1-((4-sulfamoylfuran-2-yl)methyl)azetidine-2-carboxamide (66% over 2 steps).

^{1}H NMR (400 MHz, DMSO) δ 7.91 (s, 1H), 7.71 (s, 2H), 7.66 (q, *J* = 4.7 Hz, 1H), 7.59 – 7.54 (m, 2H), 7.49 – 7.44 (m, 2H), 7.36 (t, *J* = 7.9 Hz, 1H), 7.28 (d, *J* = 1.0 Hz, 1H), 7.20 (dt, *J* = 7.8, 1.2 Hz, 1H), 7.17 – 7.13 (m, 1H), 6.91 (ddd, *J* = 8.3, 2.6, 0.9 Hz, 1H), 4.10 (q, *J* = 7.0 Hz, 2H), 3.97 (d, *J* = 9.1 Hz, 1H), 3.82 (td, *J* = 8.4, 3.2 Hz, 1H), 3.63 (dd, *J* = 13.2, 1.2 Hz, 1H), 3.45 (d, *J* = 13.1 Hz, 1H), 3.36 (t, *J* = 7.7 Hz, 1H), 2.35 (d, *J* = 4.8 Hz, 3H), 1.36 (t, *J* = 7.0 Hz, 3H). ^{13}C NMR (101 MHz, DMSO) δ 169.20, 159.01, 151.99, 143.54, 141.45, 139.20, 138.20, 129.96, 128.73, 126.10, 122.67, 118.73, 114.41, 113.20, 112.52, 69.66, 63.00, 55.72, 50.98, 48.60, 37.86, 25.08, 14.69. HRMS (ESI): calculated for $C_{24}H_{28}N_3O_5S$ $[M + H]^+$, 470.1744 found, 470.1806.

(2*S*,3*S*)-3-(5'-Chloro-2'-methyl-[1,1'-biphenyl]-3-yl)-*N*-methyl-1-((5-sulfamoylfuran-3-yl)methyl)piperidine-2-carboxamide (**51**)

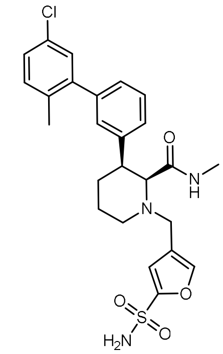

An 8 mL vial was charged with (2*S*,3*S*)-1-(((9H-fluoren-9-yl)methoxy)carbonyl)-3-(3-iodophenyl)piperidine-2-carboxylic acid (14 mg, 1.00 eq, 25 μmol) and HATU (10 mg, 1.10 eq, 28 μmol). CH_2Cl_2 (2 mL) was added followed by DIPEA (3.6 mg, 4.8 μL, 1.10 eq, 28 μmol) and the solution was stirred for 15 min. Methanamine, HCl (0.85 mg, 1.10 eq, 28 μmol) was then added as a solid and reaction was further stirred 2 hr. The solution was diluted with CH_2Cl_2 (50 mL), washed with 1 M aq. citric acid (2 x 50 mL), dried over $MgSO_4$, filtered, and solvent removed under reduced pressure. Crude product was purified by flash chromatography (0–20% MeOH in CH_2Cl_2) to afford (9*H*-fluoren-9-yl)methyl (2*S*,3*S*)-3-(3-iodophenyl)-2-(methylcarbamoyl)piperidine-1-carboxylate (13 mg, 23 μmol, 93%) as a yellow solid.

An 8 mL vial was charged with (9H-fluoren-9-yl)methyl (2*S*,3*S*)-3-(3-iodophenyl)-2-(methylcarbamoyl)piperidine-1-carboxylate (11 mg, 1 eq, 20 μmol) followed by DMF (2 mL). Piperidine (17 mg, 20 μL, 10 eq, 0.20 mmol) was added and the reaction stirred for 30 min. Solvent was removed under reduced pressure, and product purified by preparative HPLC to afford (2*S*,3*S*)-3-(3-iodophenyl)-*N*-methylpiperidine-2-carboxamide (5.3 mg, 15 μmol, 77%) as a colorless oil.

An 8 mL vial was charged with (2*S*,3*S*)-3-(3-iodophenyl)-*N*-methylpiperidine-2-carboxamide (5.2 mg, 1 eq, 15 μmol), 4-formylfuran-2-sulfonamide (3.9 mg, 1.5 eq, 23 μmol), and sodium triacetoxyborohydride (32 mg, 10 eq, 0.15 mmol). DCE (3 mL) was added, and the reaction stirred for 1.5 hr at rt. The solution was then diluted with CH_2Cl_2 (50 mL) and washed with saturated $NaHCO_3$ (3 x 50 mL). Combined aqueous washes were extracted with CH_2Cl_2 (3 x 100 mL). Organic portions were combined, dried over $MgSO_4$, filtered, and solvent removed under reduced pressure. Product was purified by preparative HPLC to afford (2*S*,3*S*)-3-(3-iodophenyl)-*N*-methyl-1-((5-sulfamoylfuran-3-yl)methyl)piperidine-2-carboxamide (6.2 mg, 12 μmol, 82%) as a white solid.

A 5 mL μW vial was charged with (2*S*,3*S*)-3-(3-iodophenyl)-*N*-methyl-1-((5-sulfamoylfuran-3-yl)methyl)piperidine-2-carboxamide (5.0 mg, 1 eq, 10 μmol), (5-chloro-2-methylphenyl)boronic acid (1.9 mg, 1.1 eq, 11 μmol), and Pd-Xphos (1.6 mg, 0.2 eq, 2.0 μmol). DMF (2 mL) was added followed by 1.8 M aq. K_3PO_4 (11 mg, 28 μL, 5 eq, 50 μmol). The vial was capped and sparged with N_2 for 5 min. The reaction was heated at 60 °C for 1 hr. After cooing the solution was diluted with 1:4 MeOH:CH_2Cl_2 (20 mL), filtered through celite and rinsed with flushing 1:4 MeOH:CH_2Cl_2 (5 x 50 mL). Volatiles were removed under reduced

pressure and crude product was purified by preparative HPLC to afford **51** (2.4 mg, 5 μmol, 48%) as an off-white solid.

^{1}H NMR (400 MHz, DMSO) δ 7.71 (d, *J* = 1.0 Hz, 1H), 7.64 (s, 2H), 7.39 (q, *J* = 4.5 Hz, 1H), 7.30 – 7.24 (m, 3H), 7.19 (dt, *J* = 7.8, 1.5 Hz, 1H), 7.10 (dq, *J* = 10.0, 1.4 Hz, 3H), 6.81 (d, *J* = 1.0 Hz, 1H), 3.47 – 3.38 (m, 2H), 3.25 – 3.20 (m, 1H), 3.09 – 2.97 (m, 2H), 2.24 (d, *J* = 4.5 Hz, 3H), 2.11 (s, 3H), 1.74 – 1.67 (m, 1H), 1.56 – 1.45 (m, 2H). ^{13}C NMR (101 MHz, DMSO) δ 170.80, 152.02, 143.49, 143.37, 142.68, 139.39, 133.92, 132.08, 130.14, 128.83, 128.28, 127.83, 127.29, 126.94, 126.79, 124.06, 113.78, 66.21, 49.17, 45.15, 44.49, 25.07, 24.62, 24.19, 19.54. HRMS (ESI): calculated for $C_{25}H_{29}ClN_3O_4S$ [M + H]$^+$, 502.1562 found, 502.1635.

(2*R*,4*S*)-1-((6,7-Dihydro-5H-pyrrolo[1,2-a]imidazol-2-yl)methyl)-*N*-methyl-4-((4'-sulfamoyl-[1,1'-biphenyl]-3-yl)oxy)pyrrolidine-2-carboxamide (**52**)

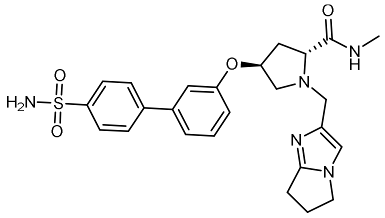

To a 1-dram vial containing (2*R*,4*S*)-1-(*tert*-butoxycarbonyl)-4-(3-iodophenoxy)pyrrolidine-2-carboxylic acid (23 mg, 53 μmol, 1 eq), NH_3MeCl (4.3 mg, 64 μmol, 1.2 eq) and HATU (24 mg, 64 μmol, 1.2 eq) were added. Anhydrous CH_2Cl_2 (400 μL) was added, followed by addition of DIPEA (46 μL, 0.27 mmol, 5 eq). The resulting yellow solution was stirred overnight and then diluted with CH_2Cl_2 and washed with water (3 x 1 mL), followed by brine (1mL). The organic phase was dried over anhydrous $MgSO_4$ and concentrated under reduced pressure to around 2 mL volume. To the resulting crude mixture was added TFA (300 μL) and after 1.5 hr the solution was concentrated under reduced pressure, then co-evaporated with toluene (1 mL). This crude mixture was used without further purification.

Crude (2*R*,4*S*)-4-(3-iodophenoxy)-*N*-methylpyrrolidine-2-carboxamide (53 μmol, 1 eq), 6,7-dihydro-5H-pyrrolo[1,2-*a*]imidazole-2-carbaldehyde (8.7 mg, 64 μmol, 1.2 eq) and sodium triacetoxyborohydride (22 mg, 0.11 mmol, 2 eq) were dissolved in DCE (500 μL), followed by addition of NEt_3 (7.4 μL, 53 μmol, 1 eq). The mixture was stirred at rt overnight, then was washed with sat. aq. $NaHCO_3$ twice and brine once. The combined organic portions were dried over anhydrous $MgSO_4$ and concentrated under reduced pressure. To the resulting residue was added (4-sulfamoylphenyl)boronic acid (16 mg, 80 μmol, 1.5 eq) and XPhos-Pd-G2 (8.3 mg, 11 μmol, 0.2 eq). The vial was capped with a septum and purged 3 times with N_2 gas. Then anhydrous DMF (2 mL) was added, followed by 1.8 M aq. K_3PO_4 (0.18 mL, 318 μmol, 6 eq). The reaction was stirred at 60 °C for 50 min under μW irradiation, then the resulting solution was diluted with DMSO and purified by preparative HPLC to afford **52** (3.4 mg, 6.9 μmol, 13%) as a white solid.

^{1}H NMR (400 MHz, CD_3OD) δ 8.00 – 7.92 (m, 2H), 7.81 – 7.73 (m, 2H), 7.38 (t, *J* = 8.0 Hz, 1H), 7.28 – 7.21 (m, 1H), 7.13 (t, *J* = 2.1 Hz, 1H), 6.94 – 6.88 (m, 2H), 4.95 (dt, *J* = 5.4, 2.8 Hz, 1H), 3.96 (t, *J* = 7.1 Hz, 2H), 3.75 (d, *J* = 13.4 Hz, 1H), 3.63 – 3.41 (m, 3H), 2.88 – 2.77 (m, 3H), 2.75 (s, 3H), 2.57 (p, *J* = 7.4 Hz, 2H), 2.46 – 2.36 (m, 1H), 2.15 (ddd, *J* = 13.6, 9.0, 5.9 Hz, 1H). ^{13}C NMR (101 MHz, CD_3OD) δ 176.86, 159.32, 155.88, 145.92, 143.95, 142.73, 142.41, 131.30, 128.53, 127.73, 121.02, 116.24, 115.61, 115.12, 77.44, 67.31, 59.14, 53.32, 46.17, 38.80, 26.97, 26.05, 23.78. HRMS (ESI): calculated for $C_{25}H_{30}N_5O_4S$ $[M + H]^+$, 496.2013 found, 496.2009.

(*R*)-4'-(6-Acetyl-2-(3-methyl-5,6,7,8-tetrahydroimidazo[1,5-*a*]pyridine-1-carbonyl)-2,6-diazaspiro[3.4]octan-8-yl)-[1,1'-biphenyl]-3-sulfonamide (**53**)

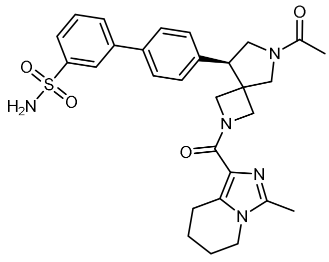

A 1-dram scintillation vial equipped with a magnetic stir bar was charged with **19** (40 mg, 67 μmol, 1.0 eq) and K_2CO_3 (18 mg, 130 μmol, 2.0 eq) were dissolved with anhydrous DMF (600 μL, 0.1 M), cooled with an ice-water bath, then thiophenol (7.6 μL, 73 μmol 1.1 eq) was added in one portion. The resulting mixture was stirred for 1 hr in the ice-water bath, then diluted with EtOAc and filtered through a pad of celite. The filtrate was concentrated under reduced pressure, and to the residue was added 3-methyl-5,6,7,8-tetrahydroimidazo[1,5-*a*]pyridine-1-carboxylic acid hydrochloride (17 mg, 80 μmol, 1.2 eq) and HATU (31 mg, 80 μmol, 1.2 eq). DMF (1 mL, 65 mM) and DIPEA (29 μL, 0.17 mmol, 2.5 eq) were added and the resulting yellow solution was stirred overnight. The reaction was diluted with EtOAc and washed three times with a 50% brine solution. The organic phase was dried over anhydrous $MgSO_4$, filtered, and concentrated under reduced pressure. Flash chromatography (0–50% EtOAc in hexanes) afforded *tert*-butyl (*R*)-8-(4-iodophenyl)-2-(3-methyl-5,6,7,8-tetrahydroimidazo[1,5-*a*]pyridine-1-carbonyl)-2,6-diazaspiro[3.4]octane-6-carboxylate (19 mg, 33 μmol, 49% over 2 steps).

To a solution of *tert*-butyl (*R*)-8-(4-iodophenyl)-2-(3-methyl-5,6,7,8-tetrahydroimidazo[1,5-*a*]pyridine-1-carbonyl)-2,6-diazaspiro[3.4]octane-6-carboxylate (19 mg, 33 μmol, 1 eq) in CH_2Cl_2 (400 μL), TFA (100 μL) was added, and the resulting solution was stirred for 1 hr at rt. The solution was concentrated under reduced pressure, and co-evaporated with MeOH three times. To the residue was added in CH_2Cl_2 (350 μL), DIPEA (17 μL, 99 μmol, 3 eq) and Ac_2O (3.7 μL, 40 μmol, 1.2 eq). After stirring for 5 hr at rt the solution was diluted with CH_2Cl_2, washed 3 times with water and solvent was removed into a 2–5 mL μW vial. 3-(4,4,5,5-Tetramethyl-1,3,2-dioxaborolan-2-yl)benzenesulfonamide (14 mg, 49 μmol, 1.5 eq) and XPhos-Pd-G2 (5.2 mg, 6.6 μmol, 0.2 eq) were added, the vial capped with a septum and purged 3 times with N_2 gas. Anhydrous DMF (0.5 mL) was added, followed by 1.8 M aq. K_3PO_4 (0.12 mL, 215 μmol, 6.5

eq). The reaction was stirred at 60 °C for 50 min under microwave irradiation. Upon cooling the dark brown suspension was diluted with water and extracted five times with CH_2Cl_2. The organic phase was dried over $MgSO_4$, and solvents were removed under reduced pressure. The residue was purified by preparative HPLC to afford **53** (4.9 mg, 8.9 µmol, 27% over 3 steps) as a colorless oil.

^{1}H NMR (400 MHz, CD_3OD) δ 8.14 (s, 1H), 7.86 (dd, *J* = 14.6, 7.8 Hz, 2H), 7.71 – 7.64 (m, 2H), 7.64 – 7.57 (m, 1H), 7.47 – 7.38 (m, 2H), 4.63 – 4.49 (m, 1H), 4.43 – 3.71 (m, 10H), 3.62 (dt, *J* = 32.3, 7.0 Hz, 1H), 2.94 – 2.74 (m, 2H), 2.26 (d, *J* = 27.4 Hz, 3H), 2.15 (d, *J* = 7.3 Hz, 3H), 1.88 (d, *J* = 23.7 Hz, 2H), 1.75 – 1.53 (m, 2H). ^{13}C NMR (101 MHz, CD_3OD) δ 170.90, 165.41, 144.42, 143.46, 141.25, 137.67, 137.35, 135.09, 130.02, 129.28, 128.67, 128.60, 126.99, 124.52, 123.96, 54.73, 50.72, 48.52, 42.52, 22.15, 20.70, 20.52, 19.04, 10.96. HRMS (ESI): calculated for $C_{29}H_{34}N_5O_4S$ $[M + H]^+$, 548.2326 found, 548.2425.

4'-((8*S*)-6-Acetyl-2-(2-(3-cyanophenoxy)propanoyl)-2,6-diazaspiro[3.4]octan-8-yl)-[1,1'-biphenyl]-4-sulfonamide (**54**)

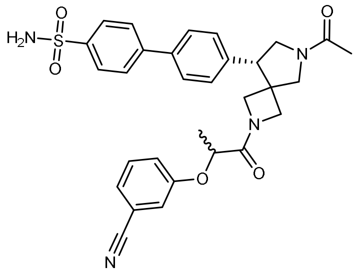

20 (20 mg, 33 µmol, 1 eq) and K_2CO_3 (9.2 mg, 67 µmol, 2 eq) were suspended in anhydrous DMF (300 µL) and cooled to 0 °C in an ice bath, followed by addition of thiophenol (3.8 µL, 37 µmol, 1.1 eq). The resulting mixture was stirred for 1 hr at 0 °C, then diluted with EtOAc and filtered through a pad of celite. The filtrate was concentrated under reduced pressure and 2-(3-cyanophenoxy)propanoic acid (7.6 mg, 40 µmol, 1.2 eq) and PyBOP (21 mg, 40 µmol, 1.2 eq) were added. The vial was purged with N_2, followed by addition of DMF (300 µL) to dissolve the reactants. DIPEA (17 µL, 99 µmol, 3 eq) was added, and the solution was stirred at rt overnight. The solution was diluted with EtOAc and washed with water (1 mL) 3 times, followed by brine (1 mL). Combined organic portions were dried over anhydrous $MgSO_4$ and concentrated under reduced pressure to approximately 2 mL volume. The product was purified by flash chromatography (EtOAc in hexanes) to afford *tert*-butyl (8*S*)-2-(2-(3-cyanophenoxy)propanoyl)-8-(4-iodophenyl)-2,6-diazaspiro[3.4]octane-6-carboxylate (7.8 mg, 13 µmol, 40% over 2 steps).

tert-Butyl (8*S*)-2-(2-(3-cyanophenoxy)propanoyl)-8-(4-iodophenyl)-2,6-diazaspiro[3.4]octane-6-carboxylate (7.0 mg, 12 µmol, 1 eq) was dissolved in CH_2Cl_2 (0.5 mL), and TFA (100 µL) was added. After 1.5 hr the solution was concentrated under reduced pressure, then co-evaporated to constant mass with toluene (1 mL). The resulting crude mixture was dissolved in CH_2Cl_2 (0.5 mL), followed by addition of Ac_2O (1.2 µL, 13 µmol, 1.05 eq), NEt_3 (5.0 µL, 36 µmol, 3 eq) and pyridine (0.97 µL, 12 µmol, 1.0 eq).

The solution was stirred at rt overnight and concentrated under reduced pressure. The resulting residue was added to a 2 mL μW vial along with (4-sulfamoylphenyl)boronic acid (3.6 mg, 18 μmol, 1.5 eq) and XPhos-Pd-G2 (1.9 mg, 2.4 μmol, 0.2 eq). The vial was capped with a septum and purged 3 times with N_2. Anhydrous DMF (2 mL) was added, followed by addition of 1.8 M aq. K_3PO_4 (40 μL, 72 μmol, 6 eq) and the reaction was stirred at 60 °C for 2 hr under μW irradiation. The resulting solution was diluted with DMSO and purified by preparative HPLC to afford **54** (3.5 mg, 6.2 μmol, 52%) as a white solid.

^{1}H NMR (400 MHz, CD_3OD) δ 8.05 – 7.93 (m, 2H), 7.85 – 7.69 (m, 3H), 7.62 – 7.55 (m, 1H), 7.55 – 7.40 (m, 1H), 7.41 – 7.18 (m, 3H), 7.16 – 6.95 (m, 2H), 4.78 – 3.40 (m, 10H), 2.18 – 2.06 (m, 3H), 1.49 (dd, *J* = 6.7, 5.6 Hz, 1H), 1.44 – 1.36 (m, 2H), 1.20 (dd, *J* = 6.7, 5.3 Hz, 1H). HRMS (ESI): calculated for $C_{30}H_{31}N_4O_5S$ [M + H]$^+$, 559.2010 found, 559.2082.

3-(3'-((2S,3R)-2-(Methylcarbamoyl)-1-(4-sulfamoylbenzoyl)pyrrolidin-3-yl)-[1,1'-biphenyl]-3-yl)propanoic acid (**55**)

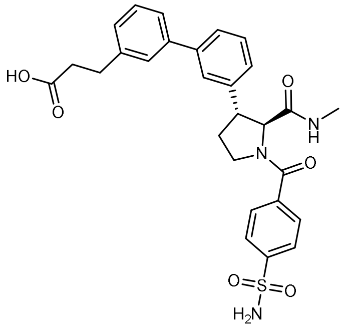

A 20 mL vial was charged with (2*S*,3*R*)-1-(*tert*-butoxycarbonyl)-3-(3-iodophenyl)pyrrolidine-2-carboxylic acid (209 mg, 1.00 eq, 500 μmol) and HATU (228 mg, 1.20 eq, 600 μmol). DMF (5 mL) was added followed by NEt_3 (126 mg, 174 μL, 2.50 eq, 1.25 mmol) and the solution was stirred for 15 min. Methanamine, HCl (67.5 mg, 2.00 eq, 1.00 mmol) was then added as a solid and the reaction was further stirred for 3 hr. The mixture was concentrated under reduced pressure and the resulting residue was dissolved as far as possible in CH_2Cl_2 (100 mL), washed with 1 M aq. citric acid (2 x 100 mL), dried over $MgSO_4$, filtered, and had solvent removed under reduced pressure. Crude product was purified by flash chromatography (0–20% MeOH in CH_2Cl_2) to afford *tert*-butyl (2*S*,3*R*)-3-(3-iodophenyl)-2-(methylcarbamoyl)pyrrolidine-1-carboxylate (153 mg, 355 μmol, 71%) as a yellow oil.

A 20 mL vial was charged with *tert*-butyl (2*S*,3*R*)-3-(3-iodophenyl)-2-(methylcarbamoyl)pyrrolidine-1-carboxylate (116 mg, 1.00 eq, 270 μmol) followed by CH_2Cl_2 (10 mL). TFA (308 mg, 208 μL, 10.0 eq, 2.70 mmol) was added via syringe and reaction stirred for 30 min. Volatiles were removed under reduced pressure. Crude was taken up in CH_2Cl_2 (50 mL) and washed 1 M K_2CO_3 (3 x 50 mL). Aqueous portions were combined and extracted with CH_2Cl_2 (3 x 100 mL). Organics were then combined, dried over Na_2SO_4, filtered, and the residue was used directly in the next step without further purification.

A 20 mL vial was charged with 4-sulfamoylbenzoic acid (7.04 mg, 1.05 eq, 35.0 μmol) and CDI (5.94 mg, 1.10 eq, 36.6 μmol). DMF (5 mL) was added, and the solution was stirred for 15 min. (2*S*,3*R*)-3-(3-iodophenyl)-*N*-methylpyrrolidine-2-carboxamide (11.0 mg, 1.00 eq, 33.3 μmol) was then added and the reaction was further stirred for 3 hr. The mixture was diluted with EtOAc (50 mL), washed with 0.5 M aq. citric acid (2 x 50 mL), dried over $MgSO_4$, filtered, and solvent was removed under reduced pressure. Purification was performed by flash chromatography (0–20% MeOH in CH_2Cl_2) to afford (*2S,3R*)-3-(3-iodophenyl)-*N*-methyl-1-(4-sulfamoylbenzoyl)pyrrolidine-2-carboxamide (42%) as a yellow solid.

A μW vial was charged with (2*S*,3*R*)-3-(3-iodophenyl)-*N*-methyl-1-(4-sulfamoylbenzoyl)pyrrolidine-2-carboxamide (26 mg, 1.00 eq, 50 μmol), 3-(3-boronophenyl)propanoic acid (12 mg, 1.25 eq, 63 μmol), and Palladium-Xphos (7.9 mg, 0.20 eq, 10 μmol). DMF (5 mL) was added followed by 1.8 M aq. K_3PO_4 (53 mg, 0.14 mL, 5.00 eq, 0.25 mmol). The vial was capped and sparged with N_2 for 5 min. The reaction was heated at 60 °C for 1 hr by μW irradiation. Once cooled the solution was diluted with 1:4 MeOH:CH_2Cl_2 (20 mL), filtered through celite and rinsed with 1:4 MeOH:CH_2Cl_2 (5 x 50 mL). Solvent was removed under reduced pressure, and crude product was purified by preparative HPLC to afford **55** (20 mg, 37 μmol, 74%) as a white solid.

^1H NMR (400 MHz, DMSO) δ 8.00 – 7.88 (m, 2H), 7.80 – 7.68 (m, 2H), 7.66 – 7.12 (m, 10H), 4.37 (dd, *J* = 98.1, 6.5 Hz, 1H), 3.93 – 3.43 (m, 4H), 2.89 (t, *J* = 7.6 Hz, 2H), 2.61 – 2.57 (m, 3H), 2.37 – 2.21 (m, 2H), 2.16 – 2.04 (m, 1H). ^{13}C NMR (101 MHz, DMSO) δ 173.92, 171.01, 170.72, 167.40, 145.28, 144.78, 141.72, 141.63, 140.58, 140.20, 139.36, 129.09, 128.85, 127.92, 127.43, 127.20, 126.89, 126.39, 126.09, 125.77, 125.67, 125.45, 125.35, 124.51, 68.04, 66.55, 49.99, 49.59, 48.28, 46.54, 35.61, 33.36, 30.60, 29.01, 28.80, 28.69, 25.67, 25.46. HRMS (ESI): calculated for $C_{28}H_{30}N_3O_6S$ $[M + H]^+$, 536.1850 found, 536.1871.

4-Cyano-*N*-(((4*S*,5*R*)-2-((*R*)-1-hydroxypropan-2-yl)-4-methyl-1,1-dioxido-8-(3-sulfamoylphenyl)-2,3,4,5-tetrahydrobenzo[*b*][1,4,5]oxathiazocin-5-yl)methyl)-*N*-methylbenzenesulfonamide (**56**)

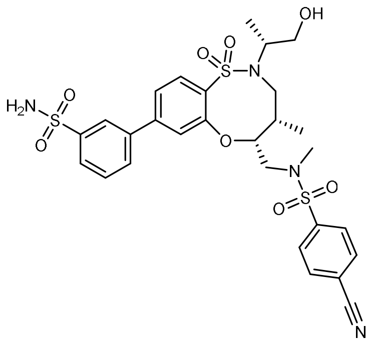

A 5 mL cone-shaped μW vial equipped with a magnetic stir bar was charged with (9H-fluoren-9-yl)methyl (((4*S*,5*R*)-8-bromo-2-((*R*)-1-hydroxypropan-2-yl)-4-methyl-1,1-dioxido-2,3,4,5-tetrahydrobenzo[*b*][1,4,5]oxathiazocin-5-yl)methyl)(methyl)carbamate (44.1 mg, 1.00 eq, 70.0 μmol), (3-

sulfamoylphenyl)boronic acid (21.1 mg, 1.5 eq, 105 µmol), XPhos-Pd-G2 (11.0 mg, 0.20 eq, 14.0 µmol), dry DMF (0.70 mL) and aq. K_3PO_4 (1.8 M, 0.12 mL, 0.22 mmol, 3.0 eq). The mixture was purged with Ar for 2 min, the vial was sealed, and the reaction mixture was heated to 60 °C for 75 min using µW irradiation. The reaction mixture was concentrated under reduced pressure and then purified by flash chromatography (0–20% MeOH [with 1% NH_4OH] in CH_2Cl_2) to afford 3-((4*S*,5*R*)-2-((*R*)-1-hydroxypropan-2-yl)-4-methyl-5-((methylamino)methyl)-1,1-dioxido-2,3,4,5-tetrahydrobenzo[*b*][1,4,5]oxathiazocin-8-yl)benzenesulfonamide (29.5 mg, 61.0 µmol, 87%) as a white solid.

A 1 dram scintillation vial equipped with a magnetic stir bar was charged with 3-((4*S*,5*R*)-2-((*R*)-1-hydroxypropan-2-yl)-4-methyl-5-((methylamino)methyl)-1,1-dioxido-2,3,4,5-tetrahydrobenzo[*b*][1,4,5]oxathiazocin-8-yl)benzenesulfonamide (10 mM in THF, 1.0 mL, 1.0 eq, 10 µmol), NEt_3 (2.0 mg, 2.8 µL, 2.0 eq, 20 µmol), 4-dimethylaminopyridine (0.1 mg, 0.1 eq, 1 µmol) and 4-cyanobenzenesulfonyl chloride (2.4 mg, 1.20 eq, 12 µmol). The reaction mixture was stirred for 40 min. CH_2Cl_2 and silica gel were added, and the suspension was concentrated under reduced pressure. The material was purified by flash chromatography (0–10% MeOH in CH_2Cl_2) affording **56** (5.1 mg, 7.9 µmol, 78%) as a colorless oil.

^{1}H NMR (400 MHz, CD_3OD) δ 8.19 (t, *J* = 1.8 Hz, 1H), 8.07 – 7.80 (m, 7H), 7.71 – 7.60 (m, 2H), 7.57 (dd, *J* = 8.2, 1.8 Hz, 1H), 4.68 (dt, *J* = 8.2, 3.8 Hz, 1H), 4.12 (dd, *J* = 15.8, 10.0 Hz, 1H), 4.01 (dq, *J* = 13.9, 6.7 Hz, 1H), 3.84 (dd, *J* = 14.0, 8.5 Hz, 1H), 3.59 – 3.47 (m, 2H), 3.38 (dd, *J* = 15.7, 5.4 Hz, 1H), 3.11 (dd, *J* = 14.0, 3.7 Hz, 1H), 2.75 (s, 3H), 2.32 – 2.20 (m, 1H), 1.23 (d, *J* = 6.9 Hz, 3H), 1.04 (d, *J* = 6.9 Hz, 3H). ^{13}C NMR (101 MHz, CD_3OD) δ 158.29, 146.05, 145.91, 142.26, 141.12, 138.49, 134.35, 132.00, 130.88, 129.75, 129.57, 126.95, 125.81, 124.67, 124.00, 118.37, 117.60, 84.74, 66.03, 58.71, 53.63, 36.33, 36.25, 15.91, 12.65. HRMS (ESI): calculated for $C_{28}H_{33}N_4O_8S_3$ $[M + H]^+$, 649.1455 found, 649.1464.

5-(Furan-2-yl)-*N*-(((4*S*,5*R*)-2-((*S*)-1-hydroxypropan-2-yl)-4-methyl-1,1-dioxido-8-(4-sulfamoylphenyl)-2,3,4,5-tetrahydrobenzo[*b*][1,4,5]oxathiazocin-5-yl)methyl)-*N*-methylisoxazole-4-carboxamide (**57**)

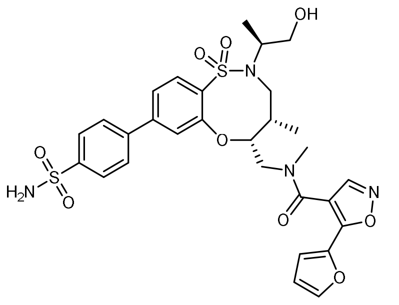

A 5 mL cone-shaped µW vial equipped with magnetic stir bar was charged with (9H-fluoren-9-yl)methyl (((4*S*,5*R*)-8-bromo-2-((*S*)-1-hydroxypropan-2-yl)-4-methyl-1,1-dioxido-2,3,4,5-tetrahydrobenzo[*b*][1,4,5]oxathiazocin-5-yl)methyl)(methyl)carbamate (52.5 mg, 1.00 eq, 83.4 µmol), (4-

sulfamoylphenyl)boronic acid (25.1 mg, 1.5 eq, 125 μmol), XPhos-Pd-G2 (13.1 mg, 0.20 eq, 16.7 μmol), aq. K_3PO_4 (1.8 M, 0.14 mL, 0.25 mmol, 3.0 eq) and dry DMF (0.83 mL). The reaction mixture was sparged with Ar for 3 min, the vial was sealed, and the reaction mixture was heated to 60 °C for 80 min using μW irradiation. The orange reaction mixture was concentrated under reduced pressure and absorbed on silica gel. The material was then purified by flash chromatography (0–20% MeOH [with 1% NH_4OH] in CH_2Cl_2) to afford 4-((4*S*,5*R*)-2-((*S*)-1-hydroxypropan-2-yl)-4-methyl-5-((methylamino)methyl)-1,1-dioxido-2,3,4,5-tetrahydrobenzo[*b*][1,4,5]oxathiazocin-8-yl)benzenesulfonamide (14.8 mg, 30.6 μmol, 37%) as a cloudy white oil.

A 2 dram scintillation vial equipped with a magnetic stir bar was charged with 4-((4*S*,5*R*)-2-((*S*)-1-hydroxypropan-2-yl)-4-methyl-5-((methylamino)methyl)-1,1-dioxido-2,3,4,5-tetrahydrobenzo[*b*][1,4,5]oxathiazocin-8-yl)benzenesulfonamide (14.8 mg, 1.00 eq, 30.6 μmol) at rt. 5-(Furan-2-yl)isoxazole-4-carboxylic acid (0.22 M in DMF, 0.17 mL, 1.2 eq, 37 μmol), DIPEA (11.2 μL, 2.1 eq, 64.3 μmol) and PyBOP (0.22 M in DMF, 0.17 mL, 1.2 eq, 37 μmol) were successively added and the brown solution was stirred for 1 hr. EtOAc and water (each ca 30 mL) were added and the aqueous phase was extracted twice with EtOAc. The organic layers were washed two more times with water, then they were combined, dried over Na_2SO_4 and concentrated under reduced pressure to afford a brown oil. The crude material was purified by preparative HPLC to afford **57** (7.9 mg, 12 μmol, 40%) as a white powder.

^{1}H NMR (400 MHz, CD_3OD) δ 8.75 (s, 0.45H), 8.45 (s, 0.55H), 8.07 – 7.83 (m, 4H), 7.76 (d, *J* = 8.2 Hz, 1H), 7.62 – 7.53 (m, 2.55H), 7.40 (s, 0.45H), 7.07 (dd, *J* = 22.4, 3.5 Hz, 1H), 6.59 – 6.49 (m, 1H), 4.94 – 4.88 (m, 0.55H), 4.79 – 4.71 (m, 0.45H), 4.17 (dd, *J* = 15.9, 8.3 Hz, 0.55H), 4.06 (dd, *J* = 14.0, 4.6 Hz, 0.55H), 3.94 (dd, *J* = 14.1, 8.5 Hz, 2H), 3.88 – 3.82 (m, 0.45H), 3.82 – 3.71 (m, 1H), 3.63 – 3.52 (m, 1H), 3.52 – 3.44 (m, 0.55H), 3.36 (dd, *J* = 15.3, 3.1 Hz, 0.45H), 3.23 – 3.12 (m, 2H), 3.08 (s, 1.55H), 2.42 – 2.30 (m, 0.55H), 2.27 – 2.17 (m, 0.45H), 1.17 (d, *J* = 7.1 Hz, 1.7H), 1.13 (d, *J* = 7.1 Hz, 3H), 0.85 (d, *J* = 7.1 Hz, 1.3H). ^{13}C NMR (101 MHz, CD_3OD) δ 165.43, 164.92, 159.81, 159.19, 157.63, 156.99, 151.01, 150.82, 147.09, 146.94, 146.44, 146.27, 145.02, 145.01, 144.99, 143.73, 143.66, 143.19, 143.05, 138.58, 129.70, 129.06, 128.78, 127.89, 124.43, 124.24, 123.70, 123.60, 114.70, 114.53, 113.38, 113.23, 110.71, 86.08, 85.16, 65.27, 65.12, 59.06, 58.85, 52.63, 49.82, 49.00, 48.79, 39.25, 36.75, 36.17, 34.10, 16.89, 13.68, 13.53. HRMS (ESI): calculated for $C_{29}H_{33}N_4O_9S_2$ $[M + H]^+$, 645.1683 found, 645.1699.

N-(((4*S*,5*S*)-2-((*R*)-1-Hydroxypropan-2-yl)-4-methyl-1,1-dioxido-8-(4-sulfamoylphenyl)-2,3,4,5-tetrahydrobenzo[*b*][1,4,5]oxathiazocin-5-yl)methyl)-*N*-methyl-5-(methylsulfonyl)furan-3-carboxamide (**58**)

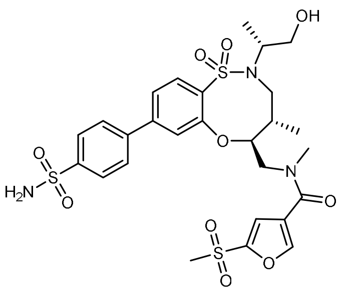

A 2-dram scintillation vial equipped with a magnetic stir bar was charged with (4*S*,5*S*)-8-bromo-2-((*R*)-1-hydroxypropan-2-yl)-4-methyl-5-((methylamino)methyl)-2,3,4,5-tetrahydrobenzo[*b*][1,4,5]oxathiazocine 1,1-dioxide [*cf.* synthesis of **36**] (29.2 mg, 1 eq, 71.7 µmol), dry DMF (0.72 mL), DIPEA (26 µL, 2.1 eq, 0.15 mmol), 5-(methylsulfonyl)furan-3-carboxylic acid (15.0 mg, 1.1 eq, 78.9 µmol) and PyBOP (41.0 mg, 1.1 eq, 78.9 µmol). The light brown solution was stirred for 18 min at rt. EtOAc and water–brine (2:1) were added and the aqueous phase was extracted twice with EtOAc. The organic layers were washed with more water–brine (2:1) and water. The organic layers were then combined and dried over Na_2SO_4. Concentration under reduced pressure afforded the crude material, which was purified by flash chromatography (0–10% MeOH in CH_2Cl_2) to afford *N*-(((4*S*,5*S*)-8-bromo-2-((*R*)-1-hydroxypropan-2-yl)-4-methyl-1,1-dioxido-2,3,4,5-tetrahydrobenzo[*b*][1,4,5]oxathiazocin-5-yl)methyl)-*N*-methyl-5-(methylsulfonyl)furan-3-carboxamide (32.5 mg, 56.1 µmol, 78%) as a colorless oil.

A 5 mL cone-shaped µW vial equipped with a magnetic stir bar was charged with *N*-(((4*S*,5*S*)-8-bromo-2-((*R*)-1-hydroxypropan-2-yl)-4-methyl-1,1-dioxido-2,3,4,5-tetrahydrobenzo[*b*][1,4,5]oxathiazocin-5-yl)methyl)-*N*-methyl-5-(methylsulfonyl)furan-3-carboxamide (32.4 mg, 1.00 eq, 55.9 µmol) in dry DMF (0.56 mL) at rt. (4-Sulfamoylphenyl)boronic acid (16.9 mg, 1.5 eq, 83.9 µmol), XPhos-Pd-G2 (8.8 mg, 20 mol%, 11 µmol) and 1.8 M aq. K_3PO_4 (0.19 mL, 6.0 eq, 0.34 mmol) were successively added and the reaction mixture was purged with Ar for 3 min. The vial was sealed, and the reaction mixture was heated to 60 °C for 1 hr using µW irradiation. EtOAc was added to the dark orange, cloudy solution and the mixture was filtered over cotton. It was concentrated under reduced pressure to afford an orange oil. The material was purified by flash chromatography (0–10% MeOH in CH_2Cl_2) affording a yellow solid. The material was further purified by preparative HPLC to give **58** (23.8 mg, 36.3 µmol, 65 %) as a white foam.

^{1}H NMR (400 MHz, $CDCl_3$) δ 8.34 (s, 0.45H), 8.03 – 7.75 (m, 4.55H), 7.65 (d, *J* = 8.2 Hz, 1H), 7.58 (td, *J* = 8.6, 1.8 Hz, 1H), 7.46 (s, 0.45H), 7.31 (d, *J* = 1.9 Hz, 0.55H), 7.20 (d, *J* = 1.8 Hz, 0.45H), 7.14 (s, 0.55H), 4.91 – 4.85 (m, 0.55H), 4.77 – 4.65 (m, 0.45H), 4.59 – 4.47 (m, 0.55H), 4.15 (dd, *J* = 14.8, 11.0 Hz, 0.45H), 3.86 – 3.73 (m, 2H), 3.73 – 3.55 (m, 3.45H), 3.43 (dd, *J* = 14.1, 2.5 Hz, 0.55H), 3.25 (s, 1.35H), 3.11 (s, 1.65H), 3.07 (s, 1.65H), 2.89 (s, 1.35H), 2.42 – 2.26 (m, 1H), 1.27 (d, *J* = 6.6 Hz, 3H), 0.98 (d, *J* = 6.9 Hz, 1.65H), 0.91 (d, *J* = 6.9 Hz, 1.35H). ^{13}C NMR (101 MHz, $CDCl_3$) δ 165.87, 165.42, 157.28, 156.61, 151.91, 151.46, 149.96, 149.30, 146.49, 145.72, 145.03, 144.93, 143.50, 143.04, 135.69, 135.53, 131.35, 131.30, 128.93, 128.42, 127.98, 124.20, 123.96, 123.84, 123.66, 123.39, 122.87, 118.37,

118.01, 85.13, 84.48, 65.62, 65.51, 58.59, 55.99, 52.98, 51.26, 43.44, 43.41, 38.47, 37.90, 37.87, 34.10, 15.94, 15.86, 15.05, 14.70. HRMS (ESI): calculated for $C_{27}H_{34}N_3O_{10}S_3$ $[M + H]^+$, 656.1401 found, 656.1414.

5-(Furan-2-yl)-*N*-(((4*R*,5*R*)-2-((*S*)-1-hydroxypropan-2-yl)-4-methyl-1,1-dioxido-8-(4-sulfamoylphenyl)-2,3,4,5-tetrahydrobenzo[*b*][1,4,5]oxathiazocin-5-yl)methyl)-*N*-methylisoxazole-4-carboxamide (**59**)

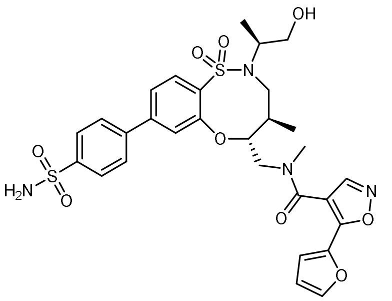

In a µW vial were combined (9H-fluoren-9-yl)methyl (((4*S*,5*R*)-8-bromo-2-((*S*)-1-hydroxypropan-2-yl)-4-methyl-1,1-dioxido-2,3,4,5-tetrahydrobenzo[*b*][1,4,5]oxathiazocin-5-yl)methyl)(methyl)carbamate (49 mg, 1 eq, 78 µmol), (4-sulfamoylphenyl)boronic acid (23 mg, 1.5 eq, 0.12 mmol), XPhos-Pd-G2 (12 mg, 0.20 eq, 16 µmol) and DMF (0.85 mL) to give an off-white cloudy mixture. The vial was capped with a rubber septum and 1.8 M aq. K_3PO_4 (0.13 mL, 3.0 eq, 0.23 mmol) was added. The mixture was degassed by evacuating at high vacuum and backfilling with N_2 five times; the vial was then capped and heated to 60 °C for 1 hr under µW irradiation. Piperidine (0.2 mL) was added, and the orange reaction mixture stirred at rt 30 min. The vial was then opened, and the reaction mixture transferred to a 100 mL round bottom flask, rinsing with EtOAc and water. Volatiles were removed under reduced pressure and the crude residue was purified by flash chromatography (0–30% MeOH [with 1% NH_4OH] in CH_2Cl_2), giving 4-((4*R*,5*R*)-2-((*S*)-1-hydroxypropan-2-yl)-4-methyl-5-((methylamino)methyl)-1,1-dioxido-2,3,4,5-tetrahydrobenzo[*b*][1,4,5]oxathiazocin-8-yl)benzenesulfonamide (28 mg, 58 µmol, 74%) as a colorless residue.

In a 20 mL glass scintillation vial 4-((4*R*,5*R*)-2-((*S*)-1-hydroxypropan-2-yl)-4-methyl-5-((methylamino)methyl)-1,1-dioxido-2,3,4,5-tetrahydrobenzo[*b*][1,4,5]oxathiazocin-8-yl)benzenesulfonamide (28 mg, 58 µmol, 1 eq) was stirred with DIPEA (0.021 mL, 2.1 eq, 0.12 mmol), 5-(furan-2-yl)isoxazole-4-carboxylic acid (12 mg, 1.20 eq, 69 µmol) and PyBOP (36 mg, 1.20 eq, 69 µmol) in DMF (0.2 mL). Additional DMF (0.4 mL) was added, giving a dark brown solution, the vial was sealed under N_2, and the reaction was stirred for 3 hr. The reaction was then quenched by diluting with additional DMF (0.6 mL), filtered through a micron filter and purified by preparative HPLC to give **59** (8.7 mg, 13.5 µmol, 23%) as a colorless solid.

^{1}H NMR (400 MHz, CD_3OD, 330 K) δ 8.16 – 7.64 (m, 7H), 7.52 (dd, *J* = 8.3, 1.8 Hz, 1H), 7.42 – 7.17 (m, 2H), 6.61 (dd, *J* = 3.7, 1.7 Hz, 1H), 4.84 (td, *J* = 9.8, 2.7 Hz, 1H), 4.45 – 4.31 (m, 1H), 3.86 – 3.55 (m, 6H),

3.15 (s, 3H), 2.41 – 2.27 (m, 1H), 1.27 (d, *J* = 6.7 Hz, 3H), 0.96 (d, *J* = 6.9 Hz, 3H). ^{13}C NMR (151 MHz, DMSO) δ 163.36, 155.73, 144.39, 144.34, 141.39, 134.65, 129.92, 127.91, 126.66, 123.06, 122.18, 113.06, 63.90, 57.42, 50.22, 49.07, 40.51, 36.51, 16.24, 15.17. LRMS (ESI): calculated for $C_{29}H_{33}N_4O_9S_2$ $[M + H]^+$, 645.17 found, 645.23.

(*R*,*E*)-4-(4-(2-Cyclohexylvinyl)phenyl)-2-((3,5-dichlorophenyl)sulfonyl)-3-(2-hydroxyethyl)-*N*-methyl-2,3-dihydro-1H-pyrrolo[3,4-*c*]pyridine-6-carboxamide (**60**)

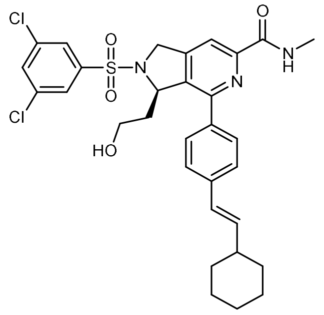

A 5 mL µW vial was charged with ethyl (*R*)-4-(4-bromophenyl)-2-((*R*)-tert-butylsulfinyl)-3-(2-hydroxyethyl)-2,3-dihydro-1H-pyrrolo[3,4-*c*]pyridine-6-carboxylate (39.6 mg, 1.00 eq, 80.0 µmol), (*E*)-(2-cyclohexylvinyl)boronic acid (19.7 mg, 1.60 eq, 128 µmol), and Palladium-Xphos (12.6 mg, 0.20 eq, 16.0 µmol). DMF (2.0 mL) was then added to the vial followed by 1.8 M aq. K_3PO_4 (84.9 mg, 222 µL, 5.00 eq, 400 µmol). The reaction vial was sparged with N_2 and capped before heating at 60 °C for 75 min. After the solution had cooled, DMF was removed under reduced pressure and the crude material was taken up in CH_2Cl_2 (40 mL). The organic layer was washed H_2O (40 mL) and brine (40 mL). After removal of solvent under reduced pressure the crude material was purified by flash chromatography (0–100% EtOAc in hexanes). Ethyl (*R*)-2-((*S*)-*tert*-butylsulfinyl)-4-(4-((*E*)-2-cyclohexylvinyl) phenyl)-3-(2-hydroxyethyl)-2,3-dihydro-1H-pyrrolo[3,4-*c*]pyridine-6-carboxylate (35 mg, 66.4 µmol, 83%) was isolated as a yellow solid.

Ethyl (*R*)-2-((*R*)-*tert*-butylsulfinyl)-4-(4-((*E*)-2-cyclohexylvinyl)phenyl)-3-(2-hydroxyethyl)-2,3-dihydro-1H-pyrrolo[3,4-*c*]pyridine-6-carboxylate (40.0 mg, 1.00 eq, 76.2 µmol) was added to an 8 mL vial and dissolved in EtOH (1.5 mL). HCl (13.9 mg, 95.3 µL, 4 M in 1,4-dioxane, 5.00 eq, 381 µmol) was then added and the solution was stirred at rt for 30 min. Volatiles were removed under reduced pressure, the residue was suspended in Et_2O (30 mL), filtered, and washed with Et_2O (2 x 30 mL). Ethyl (*R*,*E*)-4-(4-(2-cyclohexylvinyl)phenyl)-3-(2-hydroxyethyl)-2,3-dihydro-1H-pyrrolo[3,4-*c*]pyridine-6-carboxylate, HCl (34.8 mg, 76.2 µmol, 100%) was collected as an off-white solid.

An 8 mL vial was charged with 3,5-dichlorobenzenesulfonyl chloride (28 mg, 1.50 eq, 114 µmol) and a small stir bar. The vial was then capped with a septum and purged with N_2. Dry CH_2Cl_2 (4 mL) was added, and the solution was chilled to 0 °C in an ice-water bath. In a separate vial, ethyl (*R,E*)-4-(4-(2-cyclohexylvinyl)phenyl)-3-(2-hydroxyethyl)-2,3-dihydro-1H-pyrrolo[3,4-c]pyridine-6-carboxylate, HCl (34.8 mg, 1.00 eq, 76.2 µmol) was dissolved in a solution of dry CH_2Cl_2 (2 mL) and NEt_3 (116 mg, 159 µL, 15.0

eq, 1.14 mmol). This solution was then added dropwise to the reaction vial over the course of 5 min and the reaction was stirred for 18 hr with gradual warming from 0 °C to rt. The reaction solution was diluted with CH_2Cl_2 (40 mL) and washed with 0.5 M aq. citric acid (2 x 40 mL). The organic layer was dried over $MgSO_4$, filtered, and the solvent was removed under reduced pressure. The residue was then purified by flash chromatography (0–100% EtOAC in hexanes) to afford ethyl (*R*,*E*)-4-(4-(2-cyclohexylvinyl)phenyl)-2-((3,5-dichlorophenyl)sulfonyl)-3-(2-hydroxyethyl)-2,3-dihydro-1*H*-pyrrolo[3,4-*c*]pyridine-6-carboxylate (46 mg, 73.2 µmol, 96%) as a yellow powder.

Ethyl (*R*,*E*)-4-(4-(2-cyclohexylvinyl)phenyl)-2-((3,5-dichlorophenyl)sulfonyl)-3-(2-hydroxyethyl)-2,3-dihydro-1H-pyrrolo[3,4-*c*]pyridine-6-carboxylate (31.5 mg, 1.00 eq, 50.0 µmol) was weighed into an 8 mL vial containing a small stir bar, dissolved in THF (2 mL), and chilled to 0 °C in an ice-water bath. A solution of LiOH (24.0 mg, 20.0 eq, 1.00 mmol) in water (2 mL) was added drop-wise over 5 min, and the reaction was stirred for 18 hr with gradual warming from 0 °C to rt. The basic solution was acidified using a 0.5 M aq. citric acid, diluted with water (30 mL), then extracted with Et_2O (3 x 50 mL). Combined organic portions were dried over $MgSO_4$, filtered, and (*R,E*)-4-(4-(2-cyclohexylvinyl)phenyl)-2-((3,5-dichlorophenyl)sulfonyl)-3-(2-hydroxyethyl)-2,3-dihydro-1*H*-pyrrolo[3,4-*c*]pyridine-6-carboxylic acid was isolated under reduced pressure. To the resulting residue was added HATU (22.8 mg, 1.20 eq, 60.0 µmol, CH_2Cl_2 (3 mL), and NEt_3 (15.2 mg, 20.9 µL, 3.00 eq, 150 µmol). The mixture was stirred for 15 min at rt before addition of methanamine, HCl (6.75 mg, 2.00 eq, 100 µmol) and continued stirring for 2 hr. The solution was diluted with CH_2Cl_2 (50 mL), washed with 0.5 M aq. citric acid (2 x 50 mL), and dried over $MgSO_4$. After filtration solvent was removed under reduced pressure, and the residue was purified by preparative HPLC to afford **60** (24.5 mg, 40 µmol, 80% over two steps) as an off-white solid.

^1H NMR (400 MHz, DMSO) δ 8.60 (q, *J* = 4.8 Hz, 1H), 7.99 (d, *J* = 1.8 Hz, 2H), 7.88 – 7.82 (m, 2H), 7.73 (d, *J* = 8.4 Hz, 2H), 7.54 (d, *J* = 8.4 Hz, 2H), 6.48 (d, *J* = 16.1 Hz, 1H), 6.40 (dd, *J* = 16.1, 6.4 Hz, 1H), 5.63 – 5.55 (m, 1H), 5.13 (d, *J* = 17.4 Hz, 1H), 4.76 (dt, *J* = 17.3, 1.3 Hz, 1H), 4.36 (t, *J* = 5.0 Hz, 1H), 3.46 – 3.36 (m, 1H), 3.30 – 3.23 (m, 1H), 2.83 (d, *J* = 4.9 Hz, 3H), 2.19 (tdt, *J* = 10.2, 6.5, 3.4 Hz, 1H), 1.85 – 1.71 (m, 4H), 1.70 – 1.54 (m, 3H), 1.37 – 1.15 (m, 6H). ^{13}C NMR (101 MHz, DMSO) δ 163.85, 150.85, 149.59, 149.52, 139.72, 138.28, 137.72, 135.71, 135.42, 135.36, 132.91, 128.61, 126.58, 126.10, 125.84, 114.69, 62.65, 57.04, 52.36, 40.56, 35.78, 32.37, 32.33, 26.06, 25.67, 25.49. HRMS (ESI): calculated for $C_{31}H_{34}Cl_2N_3O_4S$ $[M + H]^+$, 614.1642 found, 614.1695.

((8*R*,9*R*,10*S*)-9-(4-((*E*)-2-Cyclohexylvinyl)phenyl)-6-((2-methoxyphenyl)sulfonyl)-1,6-diazabicyclo[6.2.0]decan-10-yl)methanol (**61**)

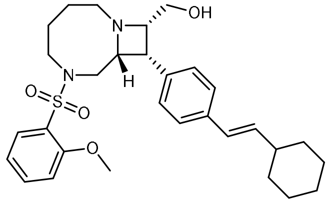

A 1-dram scintillation vial equipped with a magnetic stir bar was charged with ((8*R*,9*R*,10*S*)-9-(4-bromophenyl)-6-((2-nitrophenyl)sulfonyl)-1,6-diazabicyclo[6.2.0]decan-10-yl)methanol (12.5 mg, 24.5 µmol, 1 eq), dry DMF (0.3 mL), K_2CO_3 (16.9 mg, 122 µmol, 5 eq) and 4-methoxybenzenethiol (12.4 µL, 98 µmol, 4 eq). The yellow suspension was heated to 50 °C and stirred for 50 min. The obtained yellow/orange suspension was concentrated under reduced pressure. EtOAc (10 mL) and 0.3 M aq. HCl (10 mL) were added, and the aqueous phase was extracted twice with EtOAc. The aqueous layer was then basified with 40% aq. KOH solution and extracted three times with EtOAc. The organic layers were combined and dried over Na_2SO_4. After filtration the resulting solution was concentrated under reduced pressure and the crude ((8*R*,9*R*,10*S*)-9-(4-bromophenyl)-1,6-diazabicyclo[6.2.0]decan-10-yl)methanol (24.5 µmol, 1 eq) was dissolved in CH_2Cl_2 (0.5 mL), followed by addition of NEt_3 (6.83 µL, 49 µmol, 2.0 eq). 2-Methoxybenzenesulfonyl chloride (5.57 mg, 27.0 µmol, 1.1 eq) was added and the reaction was stirred for 1 hr before concentrating under reduced pressure. The resulting residue was added to a 2 mL µW vial along with (*E*)-(2-cyclohexylvinyl)boronic acid (4.5 mg, 29 µmol, 1.2 eq) and XPhos-Pd-G2 (3.9 mg, 4.9 µmol, 0.2 eq). The vial was capped with a septum and purged 3 times with N_2 gas and vacuum. Anhydrous DMF (0.5 mL) was added, followed by addition of 1.8 M aq. K_3PO_4 (0.16 mL, 294 µmol, 12 eq). The reaction was stirred at 60 °C for 2 hr under µW irradiation, then the resulting dark brown suspension was filtered through celite. Solvents were removed under reduced pressure, and target compound was purified by flash chromatography (30–60% EtOAc in hexanes) followed by further purification by preparative HPLC to afford **61** (2.3 mg, 4.4 µmol, 18% over 3 steps) as a white solid.

^1H NMR (400 MHz, CD_3OD) δ 7.77 (dd, *J* = 7.8, 1.7 Hz, 1H), 7.53 (ddd, *J* = 8.8, 7.5, 1.7 Hz, 1H), 7.40 (d, *J* = 8.1 Hz, 2H), 7.32 (d, *J* = 8.0 Hz, 2H), 7.07 (d, *J* = 8.4 Hz, 1H), 7.02 (t, *J* = 7.6 Hz, 1H), 6.36 (d, *J* = 16.0 Hz, 1H), 6.21 (dd, *J* = 16.0, 6.9 Hz, 1H), 3.74 – 3.40 (m, 6H), 3.53 (s, 3H), 3.16 (td, *J* = 9.2, 4.7 Hz, 1H), 2.97 – 2.86 (m, 2H), 2.50 (ddd, *J* = 12.2, 7.0, 3.2 Hz, 1H), 2.14 (dtd, *J* = 11.0, 7.3, 3.4 Hz, 1H), 2.02 – 1.91 (m, 1H), 1.88 – 1.77 (m, 6H), 1.74 – 1.60 (m, 2H), 1.45 – 1.17 (m, 6H). ^{13}C NMR (101 MHz, CD_3OD) δ 156.53, 136.78, 136.10, 134.64, 134.35, 131.00, 130.87, 126.84, 126.75, 125.03, 119.75, 112.17, 69.43, 61.10, 58.55, 54.52, 52.75, 49.47, 42.23, 41.16, 32.76, 28.35, 25.88, 25.76. HRMS (ESI): calculated for $C_{30}H_{41}N_2O_4S$ $[M + H]^+$, 525.2782 found, 525.2802.

(2*R*,4*S*)-4-(3-((*E*)-2-Cyclohexylvinyl)phenoxy)-1-((3,5-dichlorophenyl)sulfonyl)-*N*-methylpyrrolidine-2-carboxamide (**62**)

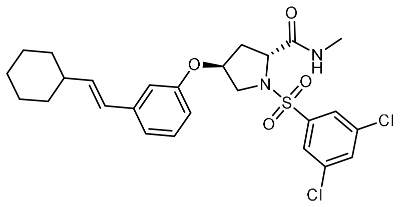

To a 1-dram vial containing (2*R*,4*S*)-1-(tert-butoxycarbonyl)-4-(3-iodophenoxy)pyrrolidine-2-carboxylic acid (17 mg, 39 µmol), NH_3MeCl (3.2 mg, 47 µmol, 1.2 eq) and HATU (18 mg, 47 µmol, 1.2 eq)

anhydrous CH_2Cl_2 (400 μL) was added, followed by DIPEA (34 μL, 200 μmol, 5 eq). The resulting yellow solution was stirred overnight at rt, diluted with CH_2Cl_2 and washed with water (1 mL) 3 times and brine (1 mL). The combined organic portions were dried over anhydrous $MgSO_4$, filtered, and concentrated under reduced pressure. The resulting crude *tert*-butyl (2*R*,4*S*)-4-(3-iodophenoxy)-2-(methylcarbamoyl)pyrrolidine-1-carboxylate was combined with analogous material from additional reactions and used for subsequent steps without further purification.

To a 2 mL μW vial was added crude *tert*-butyl (2*R*,4*S*)-4-(3-iodophenoxy)-2-(methylcarbamoyl)pyrrolidine-1-carboxylate (0.14 mmol, 1 eq), (*E*)-(2-cyclohexylvinyl)boronic acid (32 mg, 0.21 mmol, 1.5 eq) and XPhos-Pd-G2 (22 mg, 28 μmol, 0.2 eq). The vial was capped with a septum and purged 3 times with N_2 gas. Anhydrous DMF (2 mL) was added, followed by 1.8 M aq. K_3PO_4 (0.47 mL, 0.84 mmol, 6 eq). The reaction was stirred at 60 °C for 50 min under μW irradiation, then the resulting dark brown suspension was transferred to a scintillation vial and concentrated under reduced pressure. The mixture was diluted with EtOAc, followed by washing with water 3 times and brine. The combined organic portions were dried over anhydrous $MgSO_4$ and concentrated under reduced pressure. Purification by flash chromatography (50–70% EtOAc in hexanes) afforded *tert*-butyl (2*R*,4*S*)-4-(3-((*E*)-2-cyclohexylvinyl)phenoxy)-2-(methylcarbamoyl)pyrrolidine-1-carboxylate (53 mg, 0.12 mmol, 88% over 2 steps).

tert-Butyl (2*R*,4*S*)-4-(3-((*E*)-2-cyclohexylvinyl)phenoxy)-2-(methylcarbamoyl)pyrrolidine-1-carboxylate (53 mg, 0.12 mmol, 1 eq) was dissolved in CH_2Cl_2 (2 mL) before addition of TFA (400 μL) with stirring. After 1.5 hr the solution was concentrated under reduced pressure, and co-evaporated with toluene (2 mL). A portion of the resulting crude mixture was used in subsequent steps.

To a 1-dram vial charged with crude (2*R*,4*S*)-4-(3-((*E*)-2-cyclohexylvinyl)phenoxy)-*N*-methylpyrrolidine-2-carboxamide (0.03 mmol, 1 eq), 3,5-dichlorobenzenesulfonyl chloride (9 mg, 0.04 mmol, 1.2 eq) was added. This mixture was dissolved in anhydrous CH_2Cl_2 (200 μL) and chilled to 0 °C with an ice bath. NEt_3 (0.01 mL, 0.09 mmol, 3 eq) was added and the reaction was allowed to warm to rt with stirring overnight. The resulting solution was concentrated under reduced pressure, dissolved in DMSO, and directly purified by preparative HPLC to afford **62** (9 mg, 17 μmol, 60%) as a white solid.

^1H NMR (400 MHz, $CDCl_3$) δ 7.67 (d, *J* = 1.8 Hz, 2H), 7.52 (t, *J* = 1.9 Hz, 1H), 7.11 (td, *J* = 7.6, 1.1 Hz, 1H), 6.90 (dt, *J* = 7.6, 1.2 Hz, 1H), 6.70 (q, *J* = 5.1 Hz, 1H), 6.32 – 6.21 (m, 3H), 6.11 (dd, *J* = 16.0, 6.9 Hz, 1H), 4.77 – 4.70 (m, 1H), 4.31 (t, *J* = 8.3 Hz, 1H), 3.81 (dt, *J* = 13.0, 1.6 Hz, 1H), 3.75 (dd, *J* = 13.0, 3.4 Hz, 1H), 2.91 (d, *J* = 4.9 Hz, 3H), 2.52 (ddt, *J* = 14.2, 8.0, 2.0 Hz, 1H), 2.33 (ddd, *J* = 13.7, 8.7, 4.5 Hz, 1H), 2.14 (dtt, *J* = 10.7, 6.9, 3.5 Hz, 1H), 1.86 – 1.75 (m, 4H), 1.74 – 1.66 (m, 1H), 1.37 – 1.18 (m, 5H). ^{13}C NMR (101 MHz, $CDCl_3$) δ 170.91, 155.86, 139.93, 138.98, 137.71, 136.20, 133.30, 129.47, 126.70, 126.46, 120.01, 112.66, 112.13, 77.20, 74.75, 62.04, 55.00, 41.16, 37.07, 32.97, 32.95, 26.45, 26.14, 26.02. HRMS (ESI): calculated for $C_{26}H_{31}ClN_2O_4S$ $[M + H]^+$, 537.1376 found, 537.1381.

(2*S*,4*S*)-4-(4-((*E*)-2-Cyclohexylvinyl)phenoxy)-1-(3-ethynylbenzyl)-*N*-methylpyrrolidine-2-carboxamide (**63**)

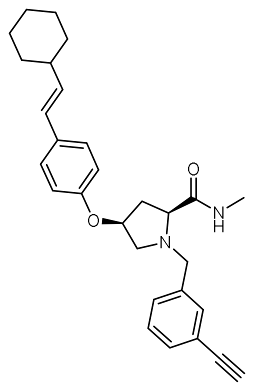

To a solution of (2*S*,4*S*)-4-(4-((*E*)-2-cyclohexylvinyl)phenoxy)-*N*-methylpyrrolidine-2-carboxamide hydrochloride [*cf*. synthesis of **29**] (15 mg, 1 eq, 41 µmol) in DCE (2 mL) was added 3-ethynylbenzaldehyde (6.4 mg, 1.2 eq, 49 µmol) and sodium triacetoxyhydroborate (13 mg, 1.5 eq, 62 µmol). The reaction mixture was stirred for 16 hr at rt and concentrated under reduced pressure. The crude residue was dissolved in CH_2Cl_2 (25 mL) and was washed with H_2O (15 mL). The aqueous layer was back-extracted with CH_2Cl_2 (2 x 25 mL), and the combined organics were dried over Na_2SO_4 and concentrated under reduced pressure. The crude residue was purified by preparative to afford **63** (10.5 mg, 58%) as a white solid.

^{1}H NMR (400 MHz, CD_3OD) δ 7.51 (d, *J* = 1.7 Hz, 1H), 7.40 (ddt, *J* = 10.9, 7.7, 1.6 Hz, 2H), 7.33 (t, *J* = 7.6 Hz, 1H), 7.27 – 7.22 (m, 2H), 6.80 – 6.74 (m, 2H), 6.28 (dd, *J* = 16.0, 1.1 Hz, 1H), 6.04 (dd, *J* = 16.0, 7.0 Hz, 1H), 4.88 (t, *J* = 5.1 Hz, 1H), 3.82 (d, *J* = 12.9 Hz, 1H), 3.67 (d, *J* = 12.9 Hz, 1H), 3.49 (s, 1H), 3.32 – 3.25 (m, 2H), 2.78 (dd, *J* = 10.8, 4.4 Hz, 1H), 2.71 (s, 3H), 2.62 (ddd, *J* = 14.2, 10.3, 5.6 Hz, 1H), 2.15 – 2.03 (m, 2H), 1.79 (ddq, *J* = 9.1, 7.0, 3.5 Hz, 4H), 1.70 (ddt, *J* = 12.4, 3.5, 1.6 Hz, 1H), 1.45 – 1.11 (m, 6H). ^{13}C NMR (151 MHz, DMSO) δ 173.04, 156.58, 139.19, 134.55, 132.57, 130.96, 130.63, 130.15, 129.00, 127.48, 126.90, 122.08, 115.86, 84.02, 81.20, 75.64, 66.98, 58.83, 58.55, 40.89, 37.13, 33.05, 26.15, 26.03, 25.98. HRMS (ESI): calculated for $C_{29}H_{35}N_2O_2$ $[M + H]^+$, 443.2693 found, 443.2696.

2. Synthesis: on-DNA

2.1. General protocols

2.1.1. Ethanol precipitation

To a reaction of a given volume (100% v/v), aq. NaCl (5 M, 10% v/v) and EtOH (300% v/v) were added. The reaction vessel was briefly vortexed and incubated on dry ice for 30 to 60 min. After centrifugation (10–20 min at 3750 x g), the supernatant was removed by decanting into another vessel. To wash the pellet, 80% aq. EtOH (100-200% v/v) was added. The mixture was vortexed for approximately 2 min and chilled again on dry ice for approximately 10 min. After centrifugation (10–20 min at 3750 x g), the supernatant was removed by decanting into another vessel. The washing procedure was repeated with 100% EtOH (100-200% v/v) and the obtained pellet was dried under vacuum.

2.2. Optimization prior to library synthesis

2.2.1. Attachment of skeletons to headpiece

2.2.1.1. Amide formation

A small number of skeletons were used for development of effective skeleton-linkage protocols. Supplementary Table 6 shows the effect of skeleton and DMTMM concentration, as well as organic solvent choice and reaction concentration. Blank data cells indicate that no peak/mass in the LCMS data could confidently be assigned to the noted species, in some cases due to a failed injection at the LCMS. General trends highlighted include:

1) Higher molar equivalents of skeleton relative to AOP-HP tended to afford greater conversion to desired product.
2) Higher organic content and/or higher dilution tended to afford greater conversion to desired product.
3) Higher molar equivalents of DMTMM relative to AOP-HP tended to afford more of the undesired acetylated AOP-HP product, which could be further augmented, though not to a dramatic extent, by a lower relative loading of skeleton.

A more focused set of reactions were performed for one enantiomer of each pair from the *meta*-iodo β-arylated Fmoc-protected amino acids. A summary of these experiments can be found in

Supplementary Table 7, and the associated learnings could be summarized as follows:

1) The order of skeleton reactivity towards acylation follows azetidine>pyrrolidine>piperidine.
2) *trans*-Configured azetidines and pyrrolidines give marginally improved conversion to desired product than their *cis*-diastereomers, with no preference between *cis*- and *trans*-piperidines.
3) High organic content affords greater conversion to desired product, regardless of ring size.

Further focusing of conditions with %organic solvent mixtures of >70%, as shown in

Supplementary Table 8, led to conditions generally affording >80%AUC product for all ring sizes and relative stereochemistry.

Supplementary Table 6 | Initial acylation conditions tested for skeleton attachment to AOP-HP

Skeleton	Conc, DMF, mM	Eq to AOP-HP	DMTMM solvent	DMTMM eq rel to AOP-HP	% org	DNA conc, mM	% AOP-HP	% P	% DMT-add	% Ac-add
Fmoc Pro	100	100	water	100	33	0.33		81		9
				10	33	0.33		97		3
			MeOH	100	66	0.33		75		15

				10	66	0.33		100		0
			DMSO	100	66	0.33		100		0
				10	66	0.33		89		3
		10	water	100	5	0.48		0		100
				10	5	0.48		50		50
			MeOH	100	52	0.48		0		100
				10	52	0.48	60	40		
			DMSO	100	52	0.48		38	10	
				10	52	0.48				
	10	100	water	100	83	0.08		74		8
				10	83	0.08		100		
			MeOH	100	92	0.08		50		44
				10	92	0.08	49	51		0
			DMSO	100	92	0.08		66		25
				10	92	0.08		90		6
		10	water	100	33	0.33		66		20
				10	33	0.33		50		50
			MeOH	100	66	0.33		47	10	31
				10	66	0.33	100			
			DMSO	100	66	0.33				5
				10	66	0.33				
Fmoc β-arylPro	100	100	water	100	33	0.33		55	6	31
				10	33	0.33		97		3
			MeOH	100	66	0.33		48	5	41
				10	66	0.33		97		3
			DMSO	100	66	0.33		89		8
				10	66	0.33		97		3
		10	water	100	5	0.48				60
				10	5	0.48	69	14		13
			MeOH	100	52	0.48				
				10	52	0.48	64			27
			DMSO	100	52	0.48				39
				10	52	0.48	55		17	28
	10	100	water	100	83	0.08	96			
				10	83	0.08		100		
			MeOH	100	92	0.08		100		

				10	92	0.08	20	62		10
			DMSO	100	92	0.08		100		
				10	92	0.08		89		7
		10	water	100	33	0.33		62	7	19
				10	33	0.33	59	33		5
			MeOH	100	66	0.33		17	8	37
				10	66	0.33	96			4
			DMSO	100	66	0.33				9
				10	66	0.33		90		10

Supplementary Table 7 **|** Screening of acylation conditions for linkage of *meta*-iodo β-arylated Fmoc-protected amino acids to AOP-HP

Skeleton (100 mM, DMF, 50 eq)	DMTMM solvent	DMTMM eq	DMTMM conc, mM	% org	final [DNA], mM	% AUC_product
cis-Aze	DMSO	50	100	50	0.50	70
			50	60	0.40	85
		30	100	44	0.56	57
			50	52	0.48	82
	water	50	100	25	0.50	27
			50	20	0.40	8
		30	100	28	0.56	20
			50	24	0.48	24
trans-Aze	DMSO	50	100	50	0.50	90
			50	60	0.40	94
		30	100	44	0.56	90
			50	52	0.48	96
	water	50	100	25	0.50	45
			50	20	0.40	12
		30	100	28	0.56	48
			50	24	0.48	9
cis-Pyr	DMSO	50	100	50	0.50	10
			50	60	0.40	30
		30	100	44	0.56	9
			50	52	0.48	13
	water	50	100	25	0.50	
			50	20	0.40	0

		30	100	28	0.56	0
			50	24	0.48	0
trans-Pyr	DMSO	50	100	50	0.50	42
			50	60	0.40	65
		30	100	44	0.56	33
			50	52	0.48	57
	water	50	100	25	0.50	5
			50	20	0.40	4
		30	100	28	0.56	
			50	24	0.48	5
cis-Pip	DMSO	50	100	50	0.50	10
			50	60	0.40	30
		30	100	44	0.56	9
			50	52	0.48	13
	water	50	100	25	0.50	0
			50	20	0.40	0
		30	100	28	0.56	4
			50	24	0.48	0
trans-Pip	DMSO	50	100	50	0.50	18
			50	60	0.40	33
		30	100	44	0.56	14
			50	52	0.48	18
	water	50	100	25	0.50	4
			50	20	0.40	5
		30	100	28	0.56	7
			50	24	0.48	5

Supplementary Table 8 | Focused screening of acylation conditions for linkage of *meta*-iodo β-arylated Fmoc-protected amino acids to AOP-HP

Skeleton (10 mM, DMF, 50 eq)	[DNA], mM	DMTMM solvent (50 mM, 50 eq)	% org	% AUC_product	% AUC_AOP-HP	% AUC_Ac-AOP-HP
cis-Aze	0.14	DMSO	86	90	2	0
		water	71	94	0	0
trans-Aze		DMSO	86	99	0	0
		water	71	92	0	0

cis-Pyr		DMSO	86	44	38	3
		water	71	72	16	5
trans-Pyr		DMSO	86	98	2	0
		water	71	100	0	0
cis-Pip		DMSO	86	63	28	8
		water	71	87	10	3
trans-Pip		DMSO	86	89	9	2
		water	71	83	13	4

2.2.1.2. Carbamylation

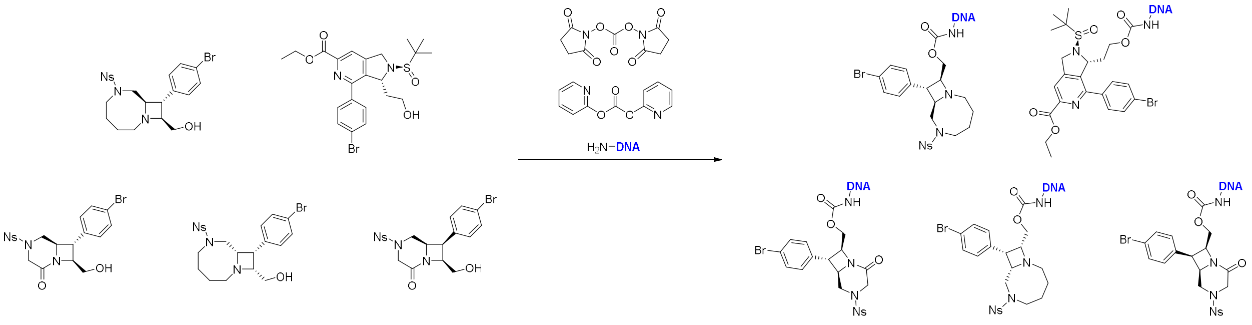

Supplementary Fig. 4 | Initial DNA-carbamate formation reactions.

An initial experiment sought to compare the efficiency of carbamate formation following skeleton incubation with two different activating dicarbonates (Supplementary Fig. 4). One of the two dicarbonates (0.5 µL 100 mM, dry DMSO, 1 eq) was mixed with a skeleton (1 µL, 50 mM, dry DMSO, 1 eq) and NEt_3 (0.5 µL, 100 mM, dry DMSO). Each reaction was set up in triplicate with the first being heated at 40 °C for 1 hr, the second being heated at 40 °C for 4 hr, and the third being incubated at rt for 24 hr. After incubation as indicated, AOP-HP (1 µL, 1 mM solution in 0.1 M PB pH 8, 1 nmol, *assumed* 0.02 eq) was added. After 24 hr at rt each reaction underwent EtOH precipitation and analysis by LCMS.

Analysis of the resulting LCMS data showed that a longer preactivation period was beneficial, DSC was more effective than dipyridin-2-yl carbonate in leading to product formation, but in most cases, unreacted SM was the result.

Then the reaction sequence represented in Supplementary Fig. 4 was repeated as a two-step sequence. First, activated carbonates were prepared by mixing a skeleton (1 eq) in dry DCM (0.1 M), to which was added NEt_3 (3 eq) followed by DSC (3 eq, 0.25 M solution in dry MeCN). The resulting solutions were

stirred until LCMS indicated conversion to desired activated carbonate (typically 24 hr), then reactions mixtures were purified by flash chromatography (4 g silica; 0–100% EtOAc in heptane). Isolated active carbonates were assumed to be 80% pure and at 80% recovery before dissoution in DMF (50 mM).

To AOP-headpiece (1 eq, 0.5 mM in 0.1 M PB pH 8) was added an active carbonate (5–10 eq, 50 mM in DMF) and additional DMF was added to ensure organic content of the reaction was 50%. Solutions were incubated at rt overnight and then underwent EtOH precipitation to afford crude products of typically >80 %AUC with respect to desired product. Example outcomes can be seen in Supplementary Fig. 5.

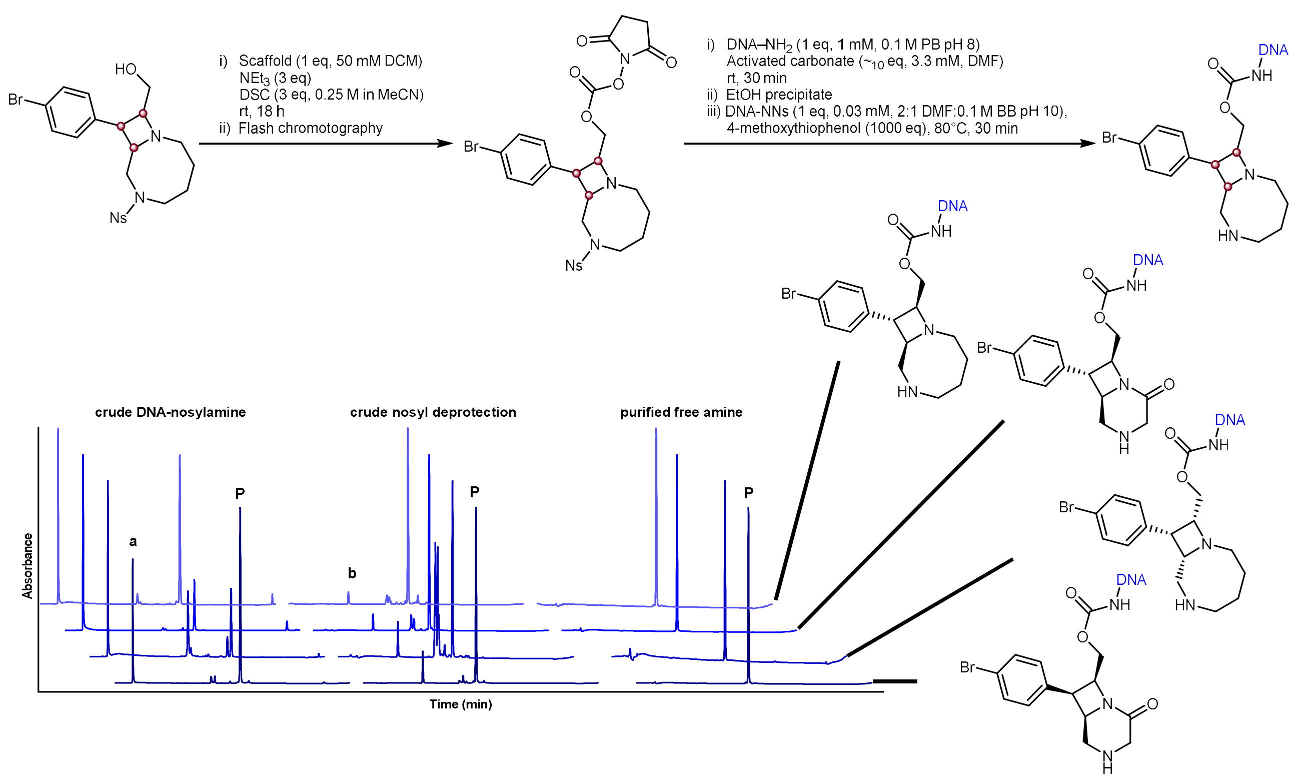

Supplementary Fig. 5 | Optimized process for carbamylation and solution-phase nosyl deprotection. Example traces showing UV absorbance at 260 nm during ion-pairing chromatography for four example bicyclic azetidine skeletons. a: excess non-DNA linked skeleton, b: residual 4-methoxy thiophenol following EtOH precipitation after nosyl deprotection, P – the desired product of each step.

2.2.2. Nosyl deprotection

An operationally facile nosyl deprotection was investigated using the skeleton-DNA construct shown in Supplementary Fig. 6.

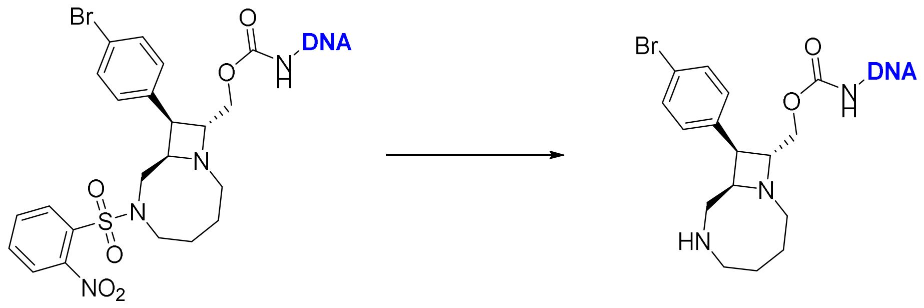

Supplementary Fig. 6 | Model skeleton employed for optimization of a nosyl deprotection.

A set of four thiols were used for initial tests (mercaptoacetic acid, mecaptoethanol, 4-methoxy thiophenol, and SiliaMetS thiol). All thiols were dissolved/suspended in DMF to a concentration of 100 mM, and 10/50/100/500/1000 eq was added a nosyl-amine-DNA construct (0.5 nmol in 5 µL of 0.1 M bicarbonate buffer pH 10.4). Samples were briefly vortexed, centrifuged and stood at rt for either 1 hr or 24 hr before analyzing by LCMS. At the 1 hr time point only 4-methoxythiophenol afforded >10% conversion at 1000 eq. At the 24 hr time point the following observations could be made:

Mercaptoacetic acid	up to 13%AUC deprotection (at 1000 eq)
Mercaptoethanol	deprotection levels between 7–41%AUC observed (10–1000 eq)
4-methoxythiophenol	deprotection levels between 11–75%AUC observed (10–1000 eq)
SiliaMetS Thiol	minimal levels of deprotection could be observed, the extent of which appeared unrelated to 'stoichiometry'

Following the observation that 4-methoxythiophenol led to the highest levels of deprotection we next investigated a set of temperature and time combinations as outlined in Supplementary Table 9. Identifying that suitable loading of 4-methoxythiophenol at elevated temperatures could afford a clean reaction profile in relatively short reaction times.

Supplementary Table 9 | Optimization of a nosyl deprotection

temp, °C	time, hr	4-methoxythiophenol (100 mM, DMF), eq	%AUC_amine	%AUC_nosyl amine
21	70	500	36	64
		1000	73	27
		2000	70	30
40	22	500	40	60

		1000	79	21
		2000	83	17
60	5	500	67	33
		1000	92	8
		2000	94	6
80	1	500	78	22
		1000	86	14
		2000	96	4

2.2.3. Suzuki coupling

A screen was performed using four model skeletons at a single concentration in a single buffer with a single concentration and number of molar equivalents of coupling partner. The skeleton electronic properties varied slightly, but not dramatically – we sought to ensure reaction conditions gave consistent outcomes without major influence from small differences in electronic effects. To that end we screened four palladium catalysts (all dissolved to 1 mM in degassed MeCN), with two boronic acid pinacol esters which were dissolved in either EtOH, MeCN, or DMA. Reactions were run at three different temperature-time combinations in heat-sealed Eppendorf twin.tec PCR plates (skirted; 951020619), then LCMS analysis was performed, and the resulting data was analyzed at the level of individual samples as well as on aggregate for each property of interest (Supplementary Fig. 7).

In total, 288 reactions were performed, of which 11 afforded complex mixtures; all involving 4-(4,4,5,5-tetramethyl-1,3,2-dioxaborolan-2-yl)-1H-indole dissolved in EtOH, and employing either $PdCl_2$(dppf).CH_2Cl_2 or $PdCl_2$(dippf) as catalyst, with 8 of those 11 reactions being from the 60 °C/5 hr conditions set. Of the four catalysts tested, $PdCl_2$(dppf).CH_2Cl_2 afforded the highest conversion to desired product at all temperatures and in all co-solvents relative to matched reaction sets. In addition, $PdCl_2$(dppf).CH_2Cl_2 usage had the least variable outcomes across a range of temperature and time combinations. We also found that MeCN was the preferred solvent for this reaction in combination with any of the catalysts tested.

a

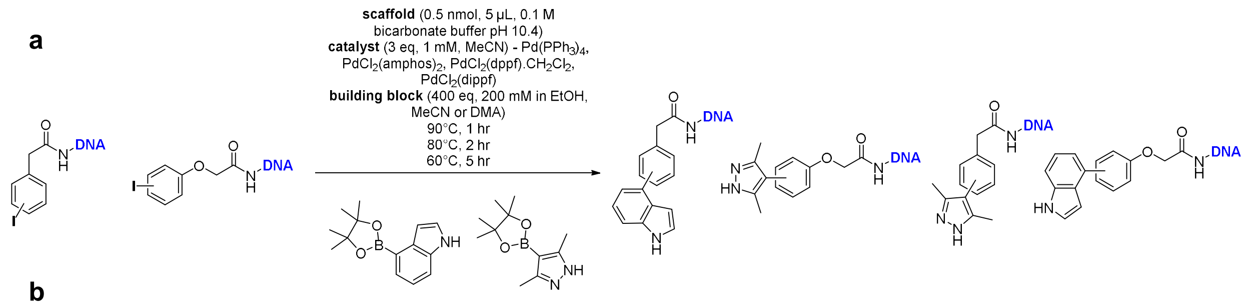

b

Catalyst	Average %AUC yields at given temperatures with various co-solvents															
solvent	all				EtOH				MeCN				DMA			
temp, °C	all	90	80	60	all	90	80	60	all	90	80	60	all	90	80	60
$Pd(PPh_3)_4$	48	61	43	40	41	44	51	29	58	81	43	48	45	58	35	43
$PdCl_2(amphos)_2$	25	32	26	17	13	9	22	9	32	42	35	19	28	41	22	21
$PdCl_2(dppf).CH_2Cl_2$	67	77	58	65	55	65	58	45	81	93	64	84	67	74	59	67
$PdCl_2(dppf)$	46	65	43	29	28	46	24	13	66	85	69	43	43	65	37	31

Supplementary Fig. 7 | Optimization of Suzuki coupling conditions. **a**, Schematic overview of reaction conditions under investigation, representing 288 individual reactions. **b**, table showing mean %AUC yields for each reaction series, corrected for residual non-DNA species with absorbance at 260 nm, and treating all reactions equally (i.e., a reaction where %AUC w.r.t product was noted as "0", such as due to ambiguous deconvoluted mass, would not be discounted from the averaging). Conditional formatting was applied to specified temperature columns corresponding to average yield (green to red corresponding to high to low, respectively), where each of these cells is the mean %AUC of desired product from 8 reactions. The conditional formatting applied to "temp: all" columns corresponds to the standard deviation across all temperature/time combinations (as % of the mean value for the given solvent).

We also investigated the performance of Suzuki couplings on skeletons bearing free amines, using multiple stereoisomers of several skeleton series (Supplementary Fig. 8). For these test reactions, three boronic acids/esters that had previously displayed consistently good performance with related substrates (with %AUC product in the 80–100% range and minimal unknown species evident in the LCMS data). We found that, as free amines, these skeletons displayed suboptimal performance, with low conversions to

desired product and high variability dependent on either the boronic acid/ester, the skeleton series, or skeleton stereochemistry.

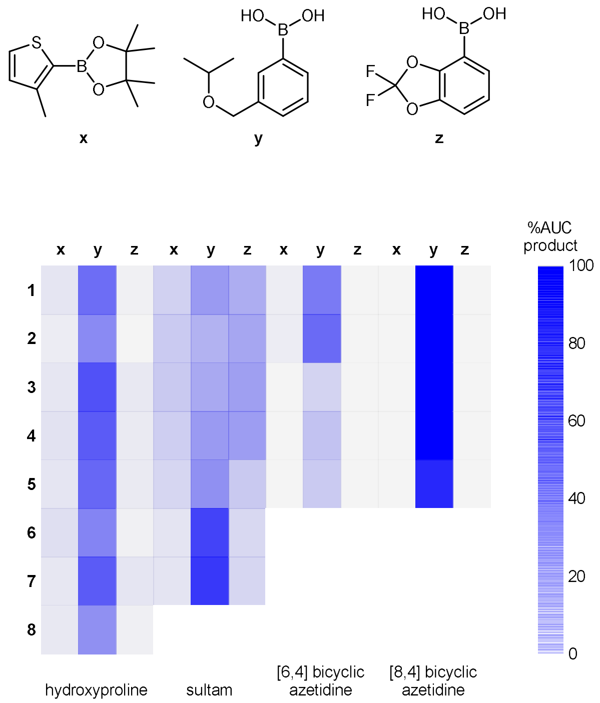

Supplementary Fig. 8 | A heat map representation of %AUC product for a subset of test Suzuki coupling reactions with a subset of skeletons bearing free amines, with blue intensity corresponding to %AUC product.

2.2.4. Skeleton validation experiments

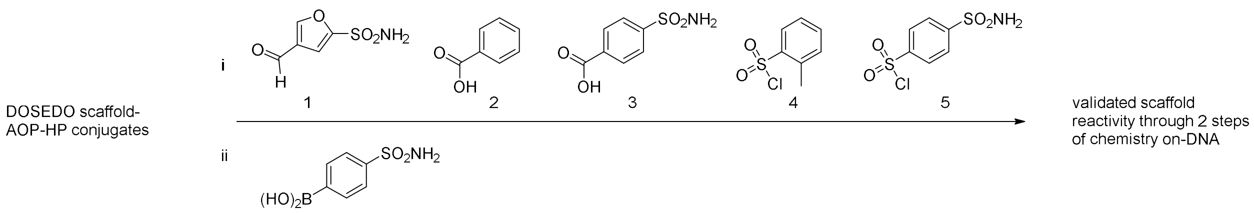

Supplementary Fig. 9 | Overview of skeleton validation reactions performed.

All skeletons to be included in the DOSEDO library (after attachment to AOP-HP, amine deprotection, purification by micropreparative HPLC, desalting by ultrafiltration and normalization of concentration in nuclease free water) required a general assessment of reactivity and stability under all conditions to be employed in the DEL synthesis (overview in Supplementary Fig. 9). Each skeleton was first assessed for

purity by LCMS (Supplementary Section 8.1), then reacted with five different amine capping building blocks followed by a single Suzuki coupling of each resulting product, under the following conditions:

Reductive amination conditions:

Skeleton-AOP-HP (2 µL, 0.9 nmol, ~0.45 mM, 1 eq, water), acetate buffer (8 µL, 1 M, pH 5) and aldehyde (2 µL, 200 mM, 444 eq, MeCN) were added to a 96-well plate (Eppendorf twin.tech lo-bind), mixing the components by three aspirate-dispense cycles. After 5 min of standing at rt $NaCNBH_3$ (1.5 µL, 333 eq, 200 mM, water) was added. The plate was sealed with heat-sealing foil, vortexed briefly, spun down, and incubated at 22 °C for 18 hr.

Acylation conditions:

DMTMM (1 µL, 100 mM, 111 eq, water, freshly prepared) and carboxylic acid (2 µL, 50 mM, 111 eq, DMF) were added to a 96-well plate (Eppendorf twin.tech, lo-bind), mixing the components by three aspirate-dispense cycles. After 5 min of standing at rt skeleton-AOP-HP (2 µL, 0.9 nmol, ~0.45 mM, 1 eq, water) was added. The plate was sealed with heat-sealing foil, vortexed briefly, spun down, and incubated at 22 °C for 18 hr.

Sulfonylation conditions:

Skeleton-AOP-HP (2 µL, 0.9 nmol, ~0.45 mM, 1 eq, water) was diluted with phosphate buffer (3 µL, 0.1M, pH 8) in a 96-well plate (Eppendorf twin.tech, lo-bind) before the addition of sulfonylchloride (1 µL, 200 mM, 222 eq, MeCN) and mixing the components by three aspirate-dispense cycles. The plate was sealed with heat-sealing foil, vortexed briefly, spun down, and incubated at 22 °C for 18 hr.

Amine capping common work-up:

Piperidine (2 µL, 10% solution in nuclease free water), was added to every reaction. After 2 min at rt each reaction was purified by EtOH precipitation, inverting supernatants into a pad of paper towels, and drying final pellets in air for several h. Pellets were dissolved in 10 µL of nuclease free water, 7 µL (~0.5 nmol) of which was transferred to a new plate for subsequent Suzuki coupling; allowing the water to evaporate overnight. The residual 3 µL was diluted with 50 µL of water and analyzed by ion-pairing LCMS

Suzuki coupling conditions:

N-capped-skeleton-AOP-HP-conjugate (0.5 nmol) was dissolved in 5 µL of 0.1 M bicarb buffer pH 10 (for the aryl iodides), or 5 µL of 0.1 M phosphate buffer pH 8 (for the aryl bromides). The added boronic acid/ester (1.5 µL, 200 mM, 600 eq1:1 mixture of MeCN and EtOH), followed by $PdCl_2$(dppf).CH_2CL_2 (1 µL [ArI] or 2.5 µL [ArBr], 2 eq [ArI] or 5 eq [ArBr], 1 mM in MeCN). Plate was sealed with heat-sealing foil, vortexed briefly, spun down, and incubated at 90 °C for 2 hr before cooling to 4 °C. Each well was treated with diethylthiocarbamic acid sodium salt (2 µL, 100 mM, water), plate sealed again, vortexed, spun down briefly, and stood at rt for approximately 1 hr. Each reaction was EtOH precipitated by addition of 5 M

NaCl (1 µL) and cold EtOH (50 µL) per well, then chilled on dry ice for ~20 min, centrifuged at 3750 x g for 15 min before removing supernatant by inversion into a pad of fresh paper towels). Another portion of cold EtOH (100 µL) was added to each well, chilled on dry ice for ~20 min, spun down at 3750 x g for 15 min before removing supernatant by inversion into a pad of fresh paper towels. The plate was then dried in air and the pellets were dissolved in water (50 µL) sealed thoroughly, vortexed briefly and centrifuged at 1000 x g for 1 min before performing LCMS analysis.

2.2.5. Building block validation experiments

2.2.5.1. Amine capping reactions

Proline was attached to AOP-HP and purified following the general procedures in Sections 2.3.1.1 and 2.3.2.1. AOP-HP (1 µmol) and Fmoc-proline (100 eq) were used, resulting in proline-AOP-HP (637 nmol [Nanodrop], 64%) after acylation, Fmoc deprotection and purification. An LCMS trace following purification can be found in Supplementary Fig. 10. The proline-AOP-HP was subjected to acylation, reductive amination and sulfonylation conditions according to the below noted quantities and orders of addition.

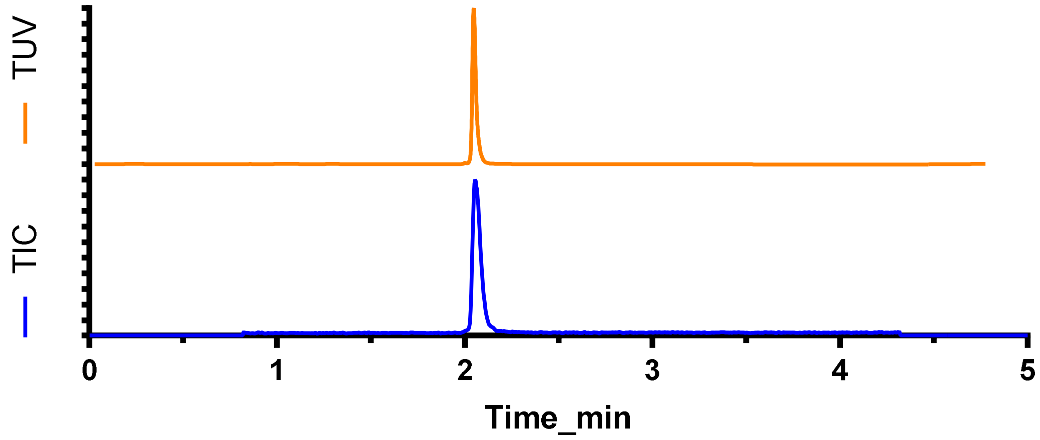

Supplementary Fig. 10 | Isolated proline-AOP-HP used for amine capping reaction validation. Orange line corresponds to tunable ultraviolet (TUV); blue line corresponds to total ion chromatogram (TIC).

<u>Reductive amination conditions:</u>

In 96-well plates proline-AOP-HP (2 µL, 0.5 nmol, 0.25 mM, 1 eq, 1 mM pH 8 PB) was diluted in acetate buffer (8 µL, 1 M, pH 5) before the addition of aldehyde (2 µL, 400 nmol, 200 mM, 800 eq, MeCN). The mixture was mixed by pipetting 3 times and stood for <5 min before the addition of $NaCNBH_3$ (1.5 µL, 300

nmol, 200 mM, 600 eq, water). The plate was heat-sealed, vortexed briefly, spun down and incubated at 25 °C for 16 hr.

Acylation conditions:

In 96-well plates freshly prepared DMTMM (0.5 μL, 50 nmol, 100 mM, 100 eq) and carboxylic acid (2 μL, 50 mM, 50 nmol, 100 eq, DMF) were spun to the bottom of each well. Proline-AOP-HP (2 μL, 0.5 nmol, 0.25 mM, 1 eq, 1 mM pH 8 PB) was added, the plate heat-sealed, vortexed briefly, spun down and incubated at 25 °C for 16 hr.

Sulfonylation conditions:

In 96-well plates proline-AOP-HP (2 μL, 0.5 nmol, 0.25 mM, 1 eq, 1 mM pH 8 PB) was diluted with PB (3 μL, 0.1 M, pH 8) before the addition of sulfonylchloride (1 μL, 200 nmol, 200 mM, 400 eq, MeCN). The plate was heat-sealed, vortexed briefly, spun down and incubated at 25 °C for 16 hr.

All three reaction types were worked-up in parallel according to the below protocol:

To each well piperidine was added (5 μL, 20%v/v in water), and plates were spun down. After <5 min the reactions underwent EtOH precipitation, and dried pellets were dissolved in 40 μL of nuclease free water for LCMS analysis.

2.2.5.2. Suzuki coupling

All boronic acids and esters available were assessed for their performance in Suzuki coupling under conditions optimized for representative ArBr and ArI-bearing skeletons of the planned DOSEDO library.

The on-DNA skeletons employed for these assessments (provided as smiles strings) were: O=C(OC[C@H]1[C@H](C2=CC=C(Br)C=C2)[C@@]3([H])CN(C(C)=O)CC(N13)=O)NC[3H] and O=C([C@H]1N(C(C)=O)CC[C@@H]1C2=CC=CC(I)=C2)NC[3H]. In both cases the smiles strings utilize a tritium atom to indicate the DNA attachment point. Also note that the amines of both skeletons were acetylated. The relevant skeleton-AOP-HP constructs were prepared according to general procedures outlined in Supplementary Sections 2.3.1.1 and 2.3.2.1, with LCMS data for isolated constructs shown in Supplementary Fig. 11.

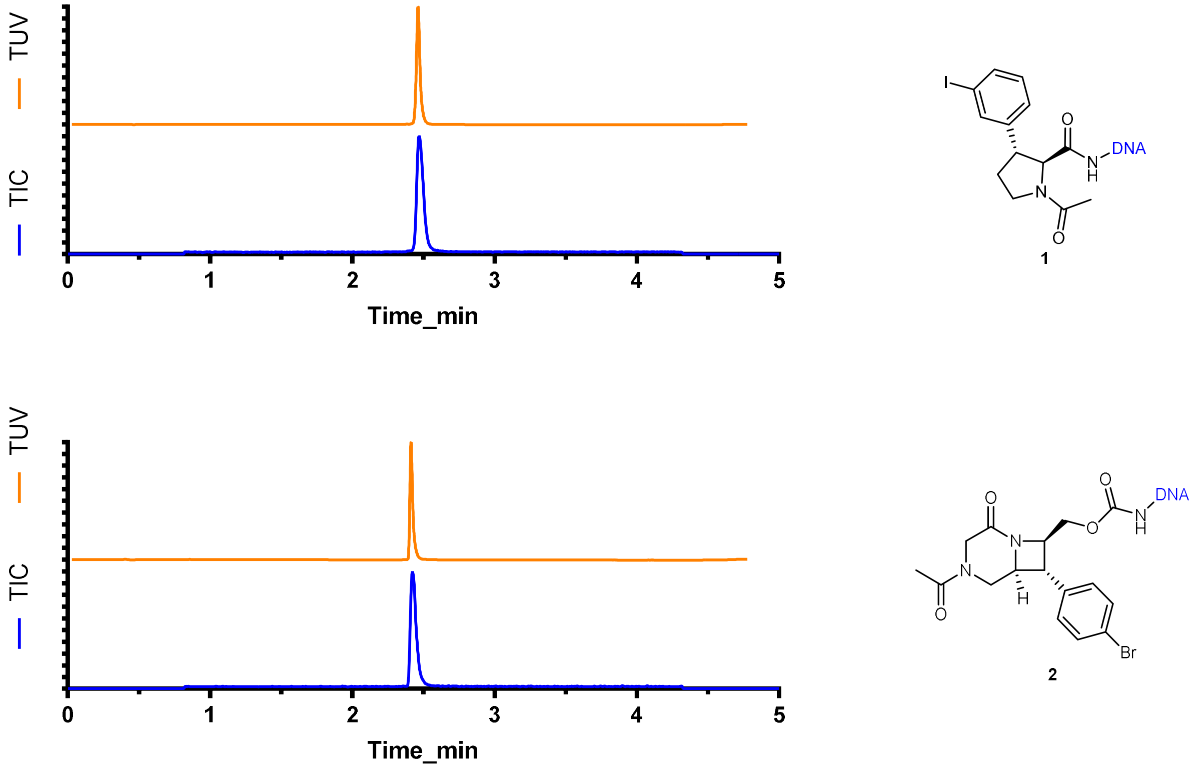

Supplementary Fig. 11 | LCMS analysis of the example ArI and ArBr skeletons used for Suzuki coupling building block validation. Orange line corresponds to tunable ultraviolet (TUV); blue line corresponds to total ion chromatogram (TIC).

In 96-well plates, a relevant skeleton-AOP-HP construct (1 µL, 0.5 nmol, 0.5 mM, nuclease free water) was diluted with bicarbonate buffer (4 µL, pH 10, 0.1 M) for the ArI-containing construct, or phosphate buffer (4 µL, pH 8, 0.1 M) for the ArBr-containing construct. This was followed by the addition of boronic acid/ester (1 µL, 200 nmol, 200 mM, 400 eq, 1:1 MeCN:EtOH) and $PdCl_2$(dppf) (1 µL, 1 nmol, 2 eq, 1 mM, MeCN, freshly prepared). Plates were heat-sealed, vortexed briefly, spun down and heated to 90 °C for 1 hr before cooling to 4 °C. Each well was treated with diethylthiocarbamic acid sodium salt, (1 µL, 100 eq w.r.t. Pd, 100 mM, water). Plates were heat-sealed again, vortexed gently, spun down and stood at rt overnight. Reactions were EtOH precipitated, pellets dried in air and dissolved in water (50 µL) before analysis by LCMS.

ArBr couplings were less efficient than expected, with only ~20% meeting the criteria of >70% conversion to product). Generally, the non-product peaks corresponded to unreacted starting material and protodebromination. To increase the ArBr Suzuki validation rate, a second set of validation reactions were performed, adapting the conditions above to 667 eq of boronic acid/ester, 5 eq of $PdCl_2$(dppf).CH_2Cl_2 with

heating at 90 °C for 2 h. This adaptation greatly improved the overlap between ArI and ArBr couplings which met criteria for inclusion in the final library build.

2.3. Library synthesis

2.3.1. Attachment of skeletons to headpiece

2.3.1.1. General acylation protocol

Part 1: An appropriate 'skeleton' [Fmoc, Boc or Ns protected amine also bearing a carboxylic acid moiety] (1 eq) was dissolved to either 10 or 50 mM in DMF and mixed with a freshly prepared solution of DMTMM (0.9 eq, 100 mM, H_2O). The mixture was vortexed briefly, centrifuged, and stood at rt for approximately 2 min.

Part 2: A solution of AOP-HP (0.03–0.07 eq, 1 mM in 0.1 M Ph 8 PB) was added to the intermediate mixture from part 1, vortexed briefly, centrifuged, and stood at rt until LCMS analysis indicated completion of reaction (typically 30 min – 20 hr). Skeletons were deprotected as outlined in Supplementary Section 2.3.2, then purified by microprep-HPLC.

2.3.1.2. General carbamoylation protocol

Part 1: An appropriate 'skeleton' [Fmoc or Ns protected amine with a free hydroxyl group] (10 – 20 mg, 15-25 µmol, 1 eq) was dissolved in dry CH_2Cl_2 to a concentration of 50 mM. To the resulting solution NEt_3 (3 eq) was charged, followed by a 0.25 M solution of DSC (3 eq) in dry MeCN. The resulting solution was stirred overnight at rt in a 1-dram vial. Crude reaction mixture was loaded directly onto a 4 g prepacked silica column and eluted with heptane/EtOAc mixtures (to remove residual DSC and NHS). The major UV-absorbing species was collected, and in all cases recovery and purity was assumed to be 80%. The activated carbamates were dissolved in DMF to a 50 mM concentration.

Part 2: AOP-HP (1 mM in 0.1 M PB pH 8, 1 eq) was diluted with DMF (2.5 volumes relative to AOP-HP) before the addition of an activated carbamate (50 mM, 10 eq), such that the final reaction mixture contained 75% DMF by volume. Reactions were briefly mixed by benchtop vortexer and centrifuged. Upon indication of completion by LCMS analysis (10 min – 1 hr) the reactions underwent EtOH precipitation and crude product amines were deprotected according to the protocols in Supplementary Section 2.3.2. Following deprotection the desired products were purified by microprep-HPLC.

2.3.2. Deprotection steps

2.3.2.1. Fmoc

For a pellet of Fmoc-protected amine-DNA conjugate (derived from an EtOH precipitation): The pellet was treated with piperidine (10% in water), vortexed briefly, centrifuged, and stood at room temp for 10–30 min. For a crude reaction mixture containing Fmoc-protected amine-DNA conjugate (100%v/v): A 20%

solution of piperidine in water (100%v/v) was added, vortexed briefly, centrifuged, and stood at rt for 10–30 min.

Another EtOH precipitation was performed, and the resulting pellet was dissolved in nuclease-free water at a concentration appropriate for microprep-HPLC purification (no more than 120 nmol per 100 µL; otherwise, UV detector signal saturation was highly likely). If any particulates presented from the solution these were removed by centrifugal filtration using Millipore ULTRAFREE-MC centrifugal filter units 0.22 µm GV DURAPORE (according to manufacturer guidelines) prior to microprep-HPLC purification.

2.3.2.2. Nosyl

A pellet of Ns-protected amine-DNA conjugate (derived from an EtOH precipitation) was dissolved in bicarbonate buffer (0.1 M, pH 10) to a concentration of approximately 0.1 mM with respect to the crude DNA-conjugate. A solution of 4-methyoxythiophenol (100 mM in DMF, 1000 eq) was then added and the mixture was heated at 80 °C for 30–60 min (when LCMS indicated complete reaction) with vigorous stirring by magnet. The mixture was cooled, then performed EtOH precipitation. The pellet was dissolved as much as possible in an appropriate volume of nuclease-free water for micro prep-HPLC purification (no more than 120 nmol per 100 µL; otherwise, UV detector signal saturation was highly likely). If any particulates presented from the solution these were removed by centrifugal filtration (as above) prior to microprep-HPLC purification.

2.3.3. Tandem ligation of forward primer binding site and cycle1_tag

Target structures:

HP FWD primer binding site Cy1_tag

5′ TGACTCCC AGGCCTGCTTCAATGTCGGATAGTG NNNNNNNCT 3′

3′ ACTGAG GGTCCGGACGAAGTTACAGCCTATC ACNNNNNNN 5′

Skeleton-AOP-HP conjugates that were previously purified by microprep-HPLC followed by ultrafiltration had their concentrations normalized to 0.45 mM with nuclease free water; guided by photospectrometry. In a 2 mL deep well plate (Costar 2 mL Assay Block (3960, 96-well, square V-bottom, Sterile, Rnase/Dnase free), purified skeleton-AOP-HP conjugates (100 nmol; 0.45 mM; 222 µL; 1.15 eq) were added.

Forward primer binding site (86 nmol; 0.86 mM; 100 µL; 1.0 eq) was added as an annealed duplex in nuclease free water, followed by the appropriate skeleton tag/cycle 1 tag duplex (174 nmol; 1 mM; 174 µL; 2.0 eq) in nuclease free water. A freshly prepared ligation mixture* (300 µL) was then added. The deep-well plate was heat-sealed, gently vortexed, quickly centrifuged and stood at rt for 16 hr.

A 0.5 µL sample was taken from each reaction, diluted in 80 µL of water and analyzed alongside primer binding site duplex and ultralow weight DNA ladder (Invitrogen, 10488096) on 4% agarose E-gel

(Invitrogen, G401004). All ligations were observed to be complete; with no detectable FWD primer binding site remaining and a solid band at ~40–45 base pairs relative to the ladder. Results are shown in Supplementary Fig. 12.

*Ligation mixture was composed of: 375 µL of 30 Weiss Units T4 ligase/µL (Invitrogen, EL0013), 3 mL of nuclease-free water (Ambion, AM9932) and 20 mL of 10x ligation buffer (Invitrogen).

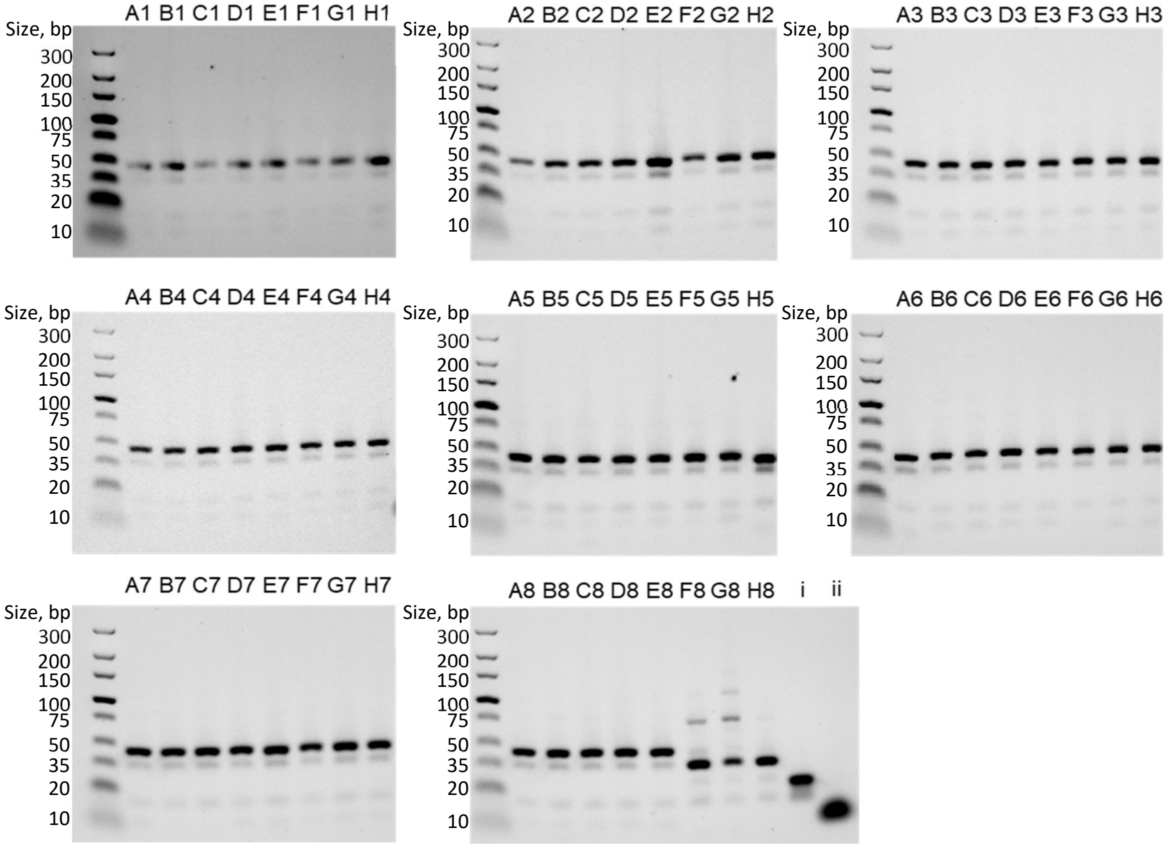

Supplementary Fig. 12 | Analysis of tandem FWD primer binding site and Cy1_tag ligation products by 4% agarose electrophoresis. F8–H8: no skeleton-headpiece construct present; i: FWD primer binding site duplex; ii: unmodified AOP-HP. The gel images represent single experiments; the experiments were not repeated.

2.3.4. Cycle 2 encoding tag ligations

Target structures:

HP FWD primer binding site Cy1_tag Cy2_tag

5′ TGACTCCC AGGCCTGCTTCAATGTCGGATAGTG NNNNNNNCT NNNNNNNAC 3′

3′ ACTGAG GGTCCGGACGAAGTTACAGCCTATC ACNNNNNNN GANNNNNNN 5′

Following ligation of FWD primer binding site and Cy1_tags, 174 nmol (2 eq relative to FWD primer binding site) of potentially ligation-competent species could be present in the mixture for each skeleton; therefore, >2 eq of Cy2_tag were required skeleton.

40 ArI-containing skeletons and 21 ArBr-containing skeletons were to be pooled separately for downstream synthetic steps, with results shown in Supplementary Fig. 13.

40 x 86 nmol (maximum quantity of each encoded ArI) = 3.44 µmol of DNA

split between 300 wells = 11.5 nmol per well; requiring 25 nmol of Cy2 tag (2.2 eq) per ligation

21 x 86 nmol (maximum quantity of each encoded ArBr) = 1.801 µmol of DNA

split between 300 wells = 6 nmol per well; requiring 13 nmol of Cy2 tag (2.2 eq) per ligation

NOTE: initially planned to perform 300 Cy2_tag ligations, however available stocks of Cy2_tags were unexpectedly depleted, so 295 ligations were performed instead.

The two encoded skeleton pools were concentrated and desalted by ultrafiltration, then dissolved in nuclease free water to an assumed concentration of 0.9 mM with respect to pooled encoded skeleton constructs (3.8 mL and 2 mL for ArI and ArBr pools respectively). These 0.9 mM solutions were treated as 1.0 mM solutions for downstream processing to allow a 10% excess volume for pipetting purposes. A fresh ligation mixture was prepared comprising: 300 µL of 30 Weiss Units T4 ligase/µL, 3000 µL of nuclease free water and 15000 µL of 10x ligation buffer. Ligations were performed in Eppendorf Deepwell plates (951032808) with the indicated volumes of components per well:

ArI: 12 µL DNA (assuming ~1.0 mM wrt DNA conjugate of interest), 25 µL tag (1 mM, ~2.2 eq), ligation mixture (30 µL) as prepared above.

ArBr: 6 µL DNA (assuming ~1.0 mM wrt DNA conjugate of interest), 13 µL tag (1 mM, ~2.2 eq), ligation mixture (16 µL) as prepared above.

After plates were heat-sealed, mixed by benchtop vortexer and spun down briefly, the ligations were stored for 16 hr at 22 °C. A 1.0 µL sample was taken from each ligation and, in a fresh 96-well plate, was diluted in 45 µL (for ArI-pooled constructs) or 32 µL (for ArBr-containing constructs) of nuclease-free water. Differential dilutions were performed to maintain equivalent absolute concentration with respect to total DNA in each sample. From these sample plates each row of 12 ligations was pooled into a single well (5 µL taken from each of the diluted ligations) to allow for a whole plate of ligations to be analyzed on a single 10-lane e-gel. Of these pooled mixtures, 2 µL was mixed with 18 µL of e-gel loading buffer (Invitrogen, 10482055) for analysis.

All crude ligations (after sampling) underwent EtOH precipitation (6 µL of 5 M NaCl, followed by 400 µL EtOH, and incubation at -20 °C for 2 days). After centrifugation at 3750 x g for 20 min and removal of supernatant, an additional 400 µL of cold 80% aq. EtOH was added to the pellets. The plates heat-sealed, mixed by benchtop vortexer, spun down quickly and chilled at -20 °C overnight. Plates were centrifuged again at 3750 x g for 20 min, supernatant was discarded, and plates were dried in air.

Concurrent with EtOH precipitation ligation performance was analyzed for both sets of ArI and ArBr plates. A faint band under the desired 'product' band, co-running with the starting material, was present in all lanes, suggestive of residual Cy1_tag ligation product. However, this had been observed numerous times in other experiments [data not reported] and is believed to be the product of a single strand ligation (due to these species not being competent in further ligations, and LCMS analysis of un-pooled ligations indicating a deconvoluted mass concurrent with ssDNA ligation). To further assess this possibility, denaturing PAGE was also performed (15% PAGE with 6 M urea). Denaturing PAGE showed additional faint bands when compared to the native agarose gel images (Supplementary Fig. 14), but the [assumed] ssDNA ligation product still could not be clearly resolved from the starting material. After denaturing PAGE, the bands were quantified using Bio-Rad Image Lab to show ~1-3% of the faint band (either SM or ssDNA ligation). 3% starting material, in the worst case, would suggest that a single ligation in the pool of 12 contained ~36% unligated material. To assess the variability, a lane containing ~3% of the faint band was used for further study (Supplementary Fig. 13 ArI-1 row H [lane 8]). Each ligation from this pool was run individually on 4% agarose gel, imaged, and quantified as for denaturing PAGE, to show very consistent ligations, with generally only ~2% of this faint band present (Supplementary Fig. 14).

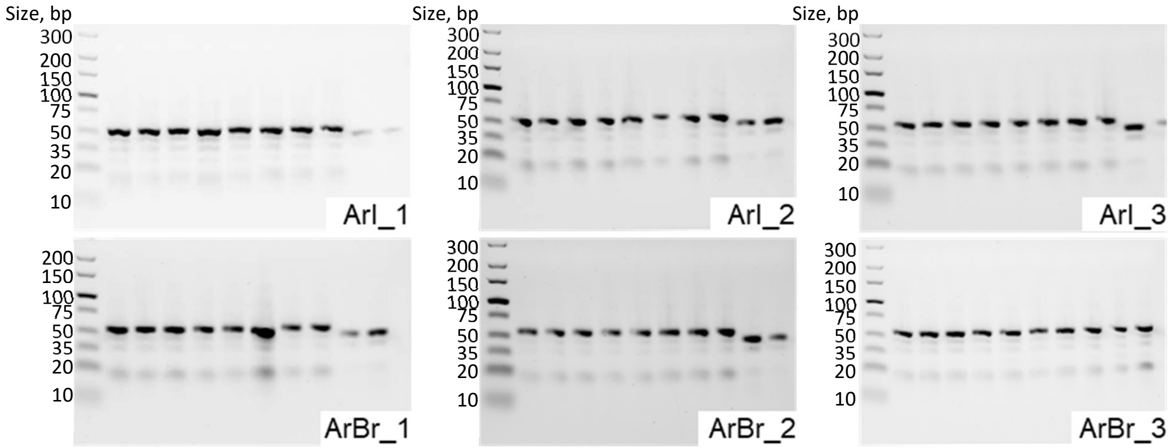

Supplementary Fig. 13 | Analysis of Cy2_tag ligations by 4% agarose e-gel. One lane contains a pooled sample from a single row of a 96-well plate. The gel image labelled as "ArBr_3" also contains the remaining 7 pooled ligations from ArI-plate4 in lane 9 and the remaining 7 pooled ligations from ArBr-plate4 in lane 10. Otherwise, lanes 9 and 10 of each gel both contain the product of tandem Cy1_tag and

FWD primer binding site ligations for comparison. The gel images represent single experiments; the experiments were not repeated.

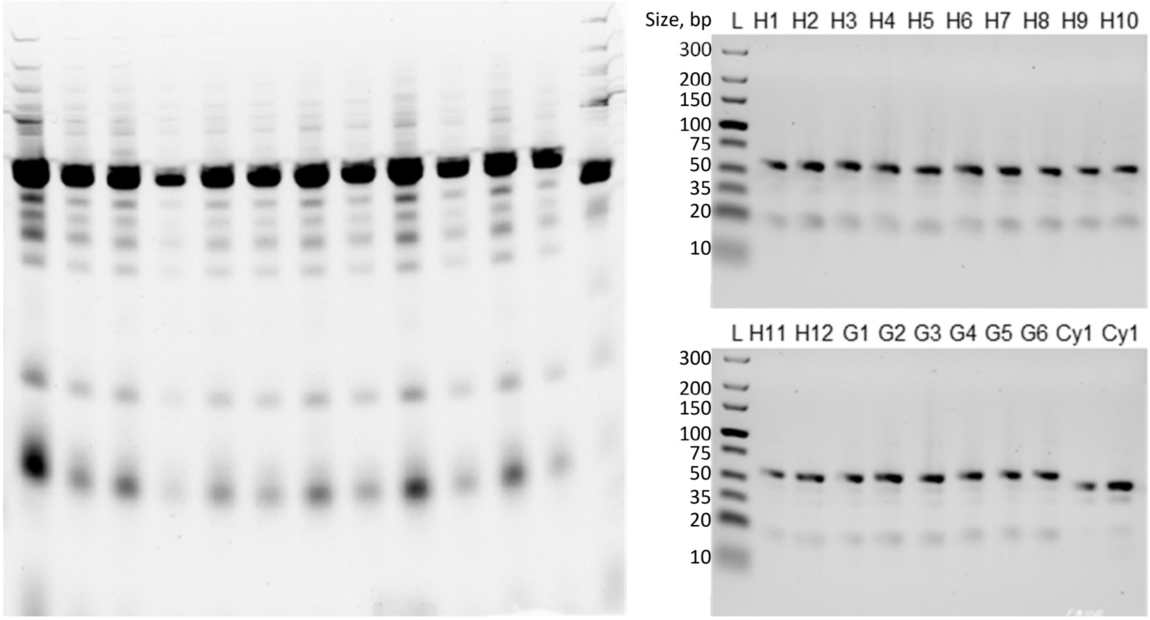

Supplementary Fig. 14 **|** Examples of pooled samples denaturing PAGE and subsequent individual sample analysis by native 4% agarose electrophoresis for cycle 2 tag ligations. Left: Denaturing PAGE of ArI_1 (rows A-H) and ArI_2 (rows A-D) [lanes 1-12], lane 13 = product of CY1_tag ligation. Gel does not include molecular weight markers, as the experiment identified relative differences between samples. Right: 4% agarose e-gel analysis of individual ligations from the ArI_1 plate, row H and some examples of row G (pooled samples of which are shown in lanes 8 and 7 of left denaturing PAGE gel respectively). The gel images represent single experiments; the experiments were not repeated.

2.3.5. Amine capping reactions

Following ligation of the Cy2_tags, EtOH precipitation, and thorough drying of pellets in air, amine-capping reactions were performed; at two different scales for the compounds derived from ArI and ArBr containing skeletons.

<u>Acylation conditions (ArI):</u>

DMTMM (10.3 µL, 100 mM, water, freshly prepared, 90 eq) and carboxylic acid (23 µL, 50 mM, DMF, 100 eq) were mixed in a 96-well twin.tec plate, then after approximately 5 min, DNA-amine (11.5 µL, 11.5

nmol, ~1 mM, 0.1 M pH 8 PB, 1 eq) was added. The plate was heat-sealed, gently vortexed, then spun down before incubating at 22 °C for 16 hr.

Reductive amination conditions (ArI):

DNA-amine (11.5 µL, 11.5 nmol, ~1 mM, 0.1 M pH 8 PB, 1 eq) was diluted with acetate buffer (58.5 µL, 1 M, pH 5) in a 96-well twin.tech plate, and aldehyde (23 µL, 200 mM, MeCN, 400 eq) was then added. After approximately 5 min, $NaCNBH_3$ (17.3 µL, 200 mM, H_2O, 300 eq) was added. The plate was heat-sealed, gently vortexed, then spun down before incubating at 22 °C for 16 hr.

Sulfonylation conditions (ArI):

To a 96-well twin.tech plate was added DNA-amine (11.5 µL, 11.5 nmol, ~1 mM, 0.1 M pH 8 PB, 1 eq), phosphate buffer (23 µL, 0.1 M, pH 8) and sulfonylchloride (11.5 µL, 200 mM, MeCN, 200 eq). The plate was heat-sealed, gently vortexed, then spun down before incubating at 22 °C for 16 hr.

Common work-up (ArI):

Piperidine (20 µL, 10%v/v in H_2O) was added to each well, then after approximately 2 min each reaction was pooled and concentrated by ultrafiltration (10 kDa MWCO). After centrifugation for 4 hr at 3750 x g, membranes were washed twice with 15 mL of nuclease free water, centrifuging at 3750 x g for 1 hr at each wash.

Acylation conditions (ArBr):

DMTMM (5.4 µL, 100 mM, water, freshly prepared, 90 eq) and carboxylic acid (12 µL, 50 mM, DMF, 100 eq) were mixed in a 96-well twin.tech plate, then after approximately 5 min, DNA-amine (6 µL, 6 nmol, ~1 mM, 0.1 M pH 8 PB, 1 eq) was added. The plate was heat-sealed, gently vortexed, then spun down before incubating at 22 °C for 16 hr.

Reductive amination conditions (ArBr):

DNA-amine (6 µL, 6 nmol, ~1 mM, 0.1 M pH 8 PB, 1 eq) was diluted with acetate buffer (34 µL, 1 M, pH 5) in a 96-well twin.tech plate, and aldehyde (12 µL, 200 mM, MeCN, 400 eq) was then added. After approximately 5 min, $NaCNBH_3$ (9 µL, 200 mM, H_2O, 300 eq) was added. The plate was heat-sealed, gently vortexed, then spun down before incubating at 22 °C for 16 hr.

Sulfonylation conditions (ArBr):

To a 96-well twin.tech plate was added DNA-amine (6 µL, 6 nmol, ~1 mM, 0.1 M pH 8 PB, 1 eq), phosphate buffer (12 µL, 0.1 M, pH 8) and sulfonylchloride (6 µL, 200 mM, MeCN, 200 eq). The plate was heat-sealed, gently vortexed, then spun down before incubating at 22 °C for 16 hr.

Common work-up ArBr:

Piperidine (10 µL, 10%v/v in H_2O) was added to each well, then after approximately 2 min, each reaction was pooled and concentrated by ultrafiltration (10 kDa MWCO). After centrifugation for 4 hr at 3750 x g, membranes were washed twice with 15 mL of nuclease free water, centrifuging at 3750 x g for 1 hr at each wash.

Following the common work up procedures two pools were obtained which contained some insoluble yellow particulates. These was filtered by centrifugation (Millipore ULTRAFREE-MC centrifugal filter units 0.22 µm GV DURAPORE). EtOH precipitation was then performed and resulting DNA pellets were dried under high vacuum for 1 hr before dissolution in nuclease free water (7.57 mL [ArI pool] and 3.97 mL [ArBr pool], ~0.5 mM [with 10% excess to enable pipetting]).

2.3.6. Cycle 3 encoding tag ligations

Target structures:

HP FWD primer binding site Cy1_tag Cy2_tag Cy3_tag

5′ TGACTCCC AGGCCTGCTTCAATGTCGGATAGTG NNNNNNNCT NNNNNNNAC NNNNNNNTT 3′

3′ ACTGAG GGTCCGGACGAAGTTACAGCCTATC ACNNNNNNN GANNNNNNN TGNNNNNNN 5′

207 ligations were required for Cy3_tag/Suzuki coupling, including the encoding of null reactions (i.e., without boronic acid or ester) in the presence of Pd-catalyst and heating, or in the absence of Pd-catalyst without heating.

ArI: 3.44 µmol of DNA (assuming no loss of material at any stage of synthesis), split between 207 wells = 16.6 nmol per well; requiring 38 nmol of Cy3_tag (2.3 eq)

ArBr: 1.801 µmol of DNA (assuming no loss of material at any stage of synthesis), split between 207 wells = 8.7 nmol per well; requiring 20 nmol of Cy3_tag (2.3 eq).

A fresh ligation mixture was prepared comprising: 300 µL of 30 Weiss Units T4 ligase/µL, 3000 µL of nuclease free water and 15000 µL of 10x ligation buffer. Ligations were performed in Eppendorf Deepwell plates (951032808) with the indicated volumes of components per well:

ArI: DNA (33.2 µL, 16.6 nmol, ~1.0 mM wrt DNA conjugate of interest), Cy3_tag (38 µL, 38 nmol, 1 mM, ~2.3 eq), ligation mixture (35 µL) as prepared above.

ArBr: DNA (17.4 µL, 8.7 nmol, ~1.0 mM wrt DNA conjugate of interest), Cy3_tag (20 µL, 20 nmol, 1 mM, ~2.3 eq), ligation mixture (19 µL) as prepared above.

After plates were heat-sealed, mixed by benchtop vortexer and spun down briefly, the ligations were stored for 16 hr at 22 °C. A 1.0 µL sample was taken from each ligation and, in a fresh 96-well plate, was diluted in 45 µL (for ArI-pooled constructs) or 32 µL (for ArBr-containing constructs) of nuclease-free water.

Samples were taken for ligation efficiency analysis by electrophoresis as described in Section 2.3.4 (gel images shown below in Supplementary Fig. 15).

All crude ligations were EtOH precipitated by the addition of 5 M NaCl (15 µL), followed by EtOH (400 µL), then storing at -20° for 2 days. Two washes with 80% EtOH (400 µL) in water were performed, and the pellets were dried thoroughly in air.

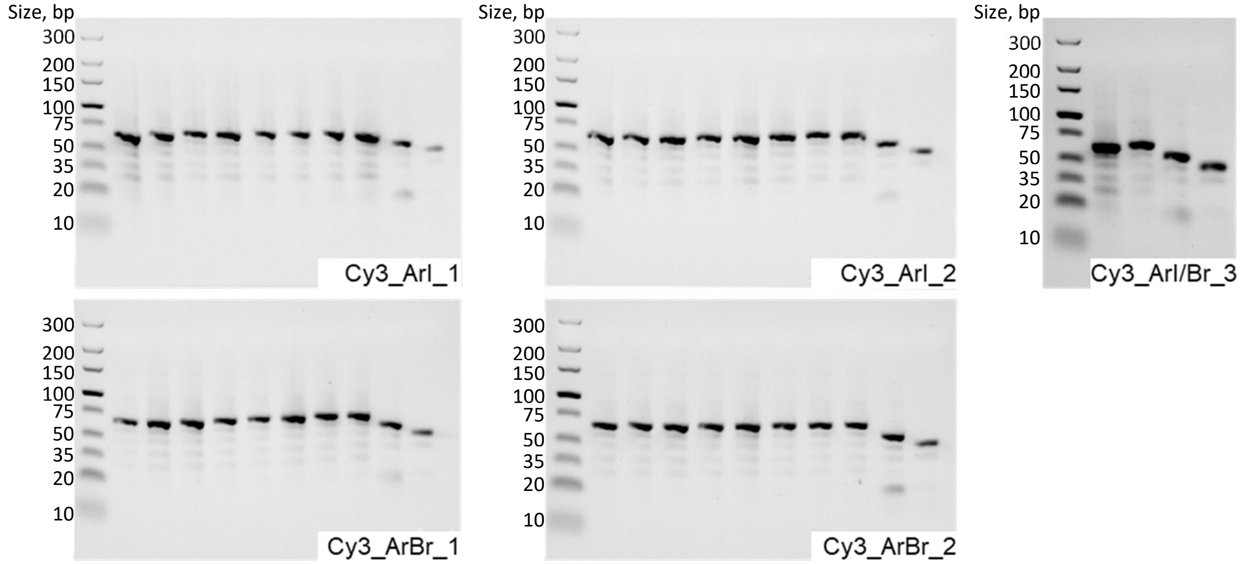

Supplementary Fig. 15 **|** Analysis of Cy3_tag ligations by 4% agarose e-gel. One lane contains a pooled sample from a single row of a standard 96-well plate. The gel image labelled as "Cy3_ArI/ArBr_3" contains the pooled ligations from ArI-plate3 in lane 1 and the pooled ligations from ArBr-plate3 in lane 2. Otherwise, the penultimate and final lanes shown on the right of each gel image contain the product of Cy2_tag ligation and product of tandem Cy1_tag and FWD primer binding site ligations, respectively, for comparison. The gel images represent single experiments; the experiments were not repeated.

2.3.7. Suzuki coupling

Product pellets derived from Cy3_tag ligations outlined in Section 2.3.6 were dissolved in the reaction buffers below (in their existing deepwell plates) and spun down before transferring to Eppendorf twin.tec plates and adding the other components. For stoichiometry/amount calculation, 100% recovery across all steps of synthesis up to this point was assumed. After addition of all components plates were sealed with heat-sealing foils, mixed by benchtop vortexer, spun down and heated to 90 °C for 1 hr (ArI) or 2 hr (ArBr), then cooled to 4 °C.

ArI:

DNA (83 µL, 16.6 nmol, 0.2 mM, 0.1 M bicarb buffer pH 10)
Boronic ester/acid (33.2 µL, 200 eq, 100 mM, 1:1 MeCN:EtOH)
$PdCl_2$(dppf) (33.2 µL, 2 eq, 1 mM, MeCN)

ArBr:

DNA(43.5 µL, 8.7 nmol, 0.2 mM, 0.1 M phosphate buffer pH 8)
Boronic ester/acid (29 µL, 333 eq, 100 mM, 1:1 MeCN:EtOH)
$PdCl_2$(dppf) (43.5 µL, 5 eq, 1 mM, MeCN)

To each well was added diethylthiocarbamic acid sodium salt (30 µL, 200 mM, water), plates were sealed, vortexed, and quickly spun down before standing at rt for 16 hr. All reactions were pooled (still maintaining ArI and ArBr-derived constructs as separate pools) before performing the following steps:

1) Performed centrifugation at 3750 x g for 2 hr and collected supernatant

2) Washed pellet with water, vortexed briefly, performed centrifugation again at 3750 x g for 1 hr then collected supernatant again

3) EtOH precipitation (added 5% v/v 5M NaCl, then 300%v/v EtOH, chilled in dry ice for 1 hr, centrifugation at 3750 x g for 30 min at 4 °C and removed supernatant)

4) Washed pellet with cold 80% EtOH in water, chilled on dry ice for 1 hr, performed centrifugation at 3750 x g for 30 min at 4 °C, removed supernatant and dried pellet under high vacuum.

5) Dissolved dried DNA pellet (still as separate ArI and ArBr-derived pools) in 1.5 mL of water and centrifuged at 14000 x g, collecting supernatant. Pellet was then treated with 400 µL water, mixed vigorously by benchtop vortexer, and centrifuged at 14000 x g again, combining supernatant with that of step 4.

The two pooled product solutions were diluted to 3.44 mL (iodides) and 1.80 mL (bromides) with nuclease free water to afford 1 mM solutions with respect to the desired encoded constructs; assuming no loss of material during library synthesis.

2.4. Small scale library closure

Prior to large scale completion of the DOSEDO library (i.e., pooling the ArI and ArBr-derived sub-libraries, ligation of a REV primer binding site, and purification), a small-scale test was first performed as outlined below. Results are shown in Supplementary Fig. 16.

Target structures ("|" indicating ligation sites):

HP FWDprimerBS Cy1_tag Cy2_tag Cy3_tag Cy4-UMI-REVprimerBS

5′TGACTCCC|AGGCCTGCTTCAATGTCGGATAGTG|NNNNNNNCT|NNNNNNNAC|NNNNNNNTT|CCGTGCTTGA NNNNNNNNNNNNCTGATGGAGGTAGAAGCCGC 3′

3′ACTGAG|GGTCCGGACGAAGTTACAGCCTATC|ACNNNNNNN|GANNNNNNN|TGNNNNNNN|AAGGCACGAACT 5′

ArI-derived pool (3.44 µL, 3.44 nmol) and ArBr-derived pool (1.80 µL, 1.80 nmol) were mixed in a 1.5 mL lo-bind tube (Eppendorf). To the pooled library (5.24 µL, 5.24 nmol) nuclease free water was added (5 µL), 10x ligation buffer (9 µL, Invitrogen), and annealed Cy4-UMI-REVprimerBS duplex [see above] (13.1 µL, 1 mM, 2.5 eq). Then was added 30 U/µL T4 DNA ligase (0.5 µL, Invitrogen), the mixture was vortexed, quickly spun down and stood at rt for 16 hr. A 0.5 µL sample of the crude ligation was diluted in 70 µL nuclease free water and analyzed alongside the pooled ligation samples from during library synthesis (Supplementary Fig. 17).

A 28 mm internal diameter Model 491 prep cell gel tube assembly was cast with 4% agarose in 1X TBE buffer to 9.25 cm and left to cure over 2 hr with circulating room temp water through the cold finger at ~100 mL/min. The prep cell was assembled according to manufacturer's instructions. After assembly, and using 1x TBE as running buffer, sample was loaded in 1x Novex TBE high density loading buffer with xylene cyanol and bromophenol blue as dyes. Electrophoresis was performed at 12 W constant power. Fraction collection was started after approximately 1 hr (when the faster-running dye reached the end of the gel column), with a flow rate of approximately 1 mL/min and 2 min fractionation. Each fraction was analyzed by 4% agarose e-gel (4 µL samples of each fraction diluted in 16 µL of e-gel loading buffer). Acceptably clean product containing fractions (25–30) were pooled before concentration and desalting by ultrafiltration, washing twice with nuclease free water. The purified desalted concentrate (250 µL) was collected and quantified by Qbit and NanoDrop indicating a yield of 1.0–1.7 nmol (19–37% assuming quantitative recovery through all steps of synthesis and purification).

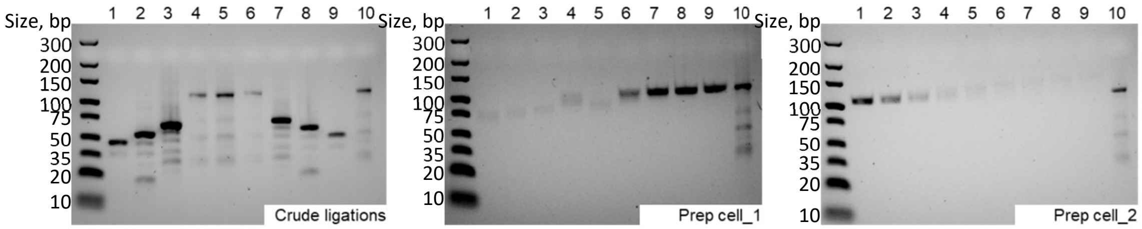

Supplementary Fig. 16 **|** Crude ligations: lanes 1/9, 2/8 and 3/7 show the crude products of Cy1_tag, Cy2_tag and Cy3_tag ligations. Lanes 4, 5, 6 and 10 show the crude product of Cy4-UMI-REVprimerBS ligation. Prep cell_1 and Prep cell_2 show individual fraction contents after prep cell purification alongside the crude Cy4-UMI-REVprimerBS duplex ligation product in Lane 10. Prep cell_1 lanes 6, 7, 8 and 9 plus Prep cell_2 lanes 1, 2 and 3 are fractions 25 – 30, which were pooled for further work (note: lanes 1 and 2

of Prep cell_2 gel are the same samples [at a slightly lower concentration] as those in lanes 8 and 9 of Prep Cell_1 gel image). The gel images represent single experiments; the experiments were not repeated.

2.5. Large scale library closure

Target structures ("|" indicating ligation sites):

HP FWDprimerBS Cy1_tag Cy2_tag Cy3_tag Cy4-UMI-REVprimerBS

5′TGACTCCC|AGGCCTGCTTCAATGTCGGATAGTG|NNNNNNNCT|NNNNNNNAC|NNNNNNNTT|CCGTGCTTGA NNNNNNNNNNNNCTGATGGAGGTAGAAGCCGC 3′

3′ACTGAG|GGTCCGGACGAAGTTACAGCCTATC|ACNNNNNNN|GANNNNNNN|TGNNNNNNN|AAGGCACGAACT 5′

Based on input sequence analysis of the small scale DOSEDO_v1 library closure, the assumed 1:1 pooling of ArI- and ArBr-derived sub-libraries afforded a 1.31:1 (ArI:ArBr) ratio of sequences at the individual skeleton level (on average). To correct for the imperfect pooling of sub-libraries 407.5 µL of the previously assumed 1 mM crude ArBr sub-library sample was pooled with 592.5 µL of the ArI sub-library (also previously assumed to be 1 mM) to give 1 mL of a new pool. Based on there being 40 ArI skeletons and 21 ArBr skeletons included in the DOSEDO library, this suggests a 1.31-fold correction, as desired.

Assuming the 1 mL pool of sub-libraries was at a concentration of 1 mM (as initially intended), annealed closing duplexes (2.5 mL, 1 mM, 2.5 eq) were added followed by 30 U/µL T4 DNA ligase (100 µL, ~3 Weiss units per nmol template DNA, Invitrogen) in 10 x ligation buffer (3 mL, Invitrogen). The mixture was vortexed, quickly centrifuged and stood at rt until analysis by 4% agarose e-gel electrophoresis indicated complete ligation (approximately 1 hr). EtOH precipitation was performed, with pellets being dried under high vacuum before dissolving in minimal nuclease free water.

A 28 mm internal diameter Model 491 prep cell gel tube assembly was cast with 4% agarose in 1X TBE buffer to 9.0 cm and left to set over 2 hr with circulating room temp water through the cold finger at ~100 mL/min. a thin layer of 1:1 water:IPA was overlaid on the gel, which was stood overnight without further buffer recirculation through the cold finger. The prep cell was assembled according to manufacturer's instructions. After assembly, and using 1x TBE as running buffer, sample was loaded in Novex TBE high density loading buffer (5x, LC6678, Invitrogen) with xylene cyanol and bromophenol blue as dyes. Electrophoresis was performed at 12 W constant power. Fraction collection was started after approximately 1 hr (when the faster-running dye reached the end of the gel column), with a flow rate of approximately 1 mL/min and 2 min fractionation. Total electrophoresis run time was 4.5 hr. fractions were analyzed by 4% agarose e-gel. Acceptably clean product containing fractions (94–120) were pooled

before concentration and desalting by ultrafiltration, washing twice with nuclease free water. The purified and desalted concentrate was collected and quantified by Qbit and NanoDrop indicating a yield of 167–247 nmol (17-25% assuming quantitative recovery through all steps of synthesis and purification). Results are shown in Supplementary Fig. 17.

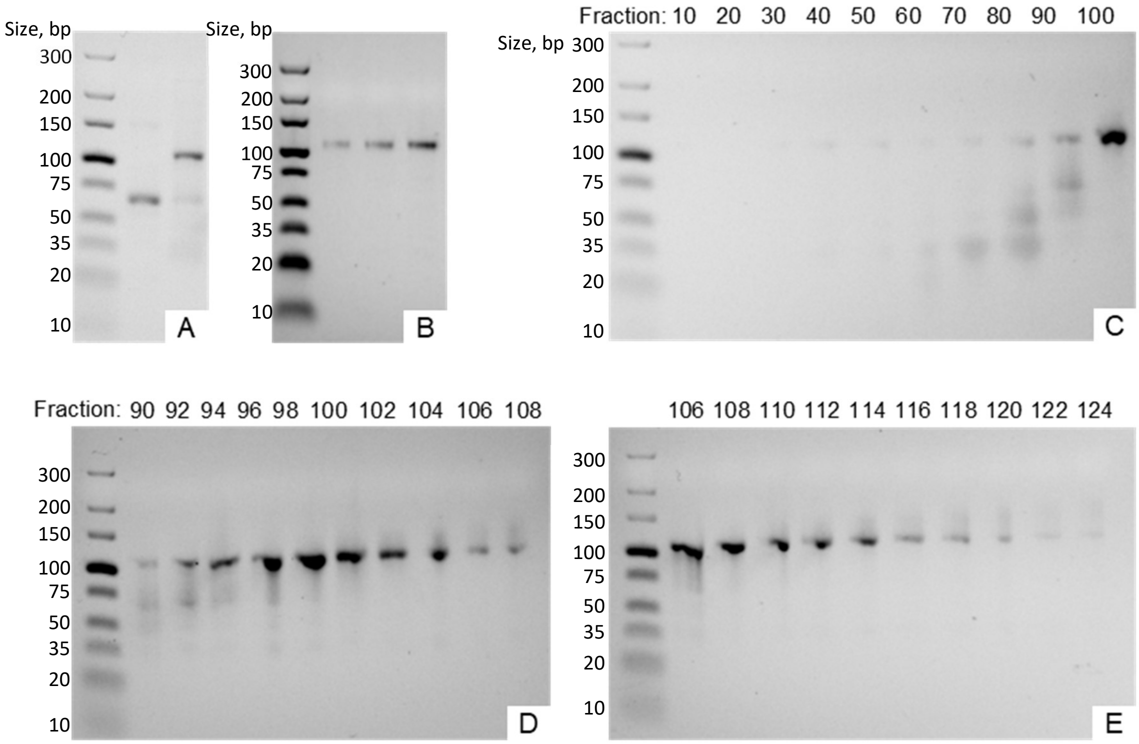

Supplementary Fig. 17 | 4% agarose e-gel analysis of ligation samples and fractions from prep cell purification. A: lane 1 – crude product of Cy3_tag ligation, lane 2 – crude product of Cy4-UMI-REVprimerBS ligation; B: increasing concentration of the prep cell purified closed DOSEDO library (pooled fractions); C: Assessment of every 10th fraction during prep cell purification of the closed DOSEDO library; D and E: Assessment of every 2nd fraction for the region containing desired DOSEDO library product. The gel images represent single experiments; the experiments were not repeated.

3. Preparation of next generation sequencing libraries – Supplementary Figures

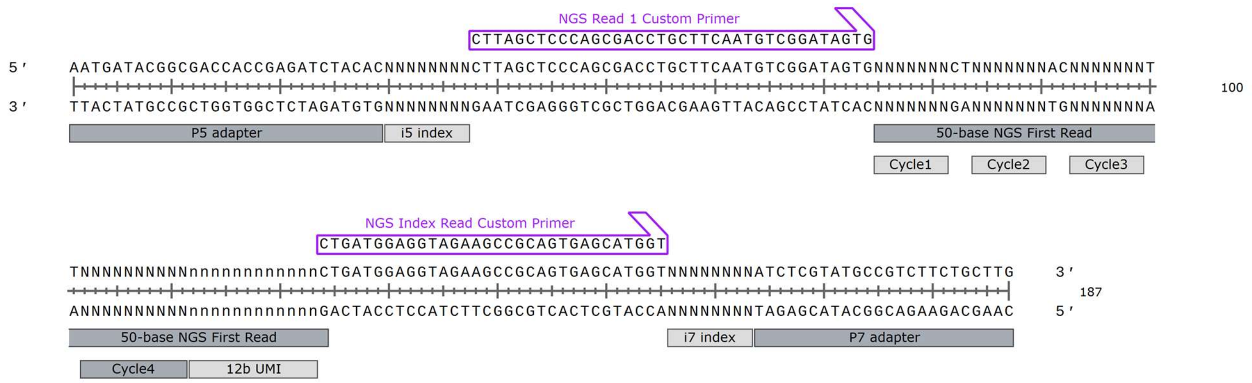

Supplementary Fig. 18 | Overview of DOSEDO coding scheme as prepared for NGS. Prepared with SnapGene Viewer.

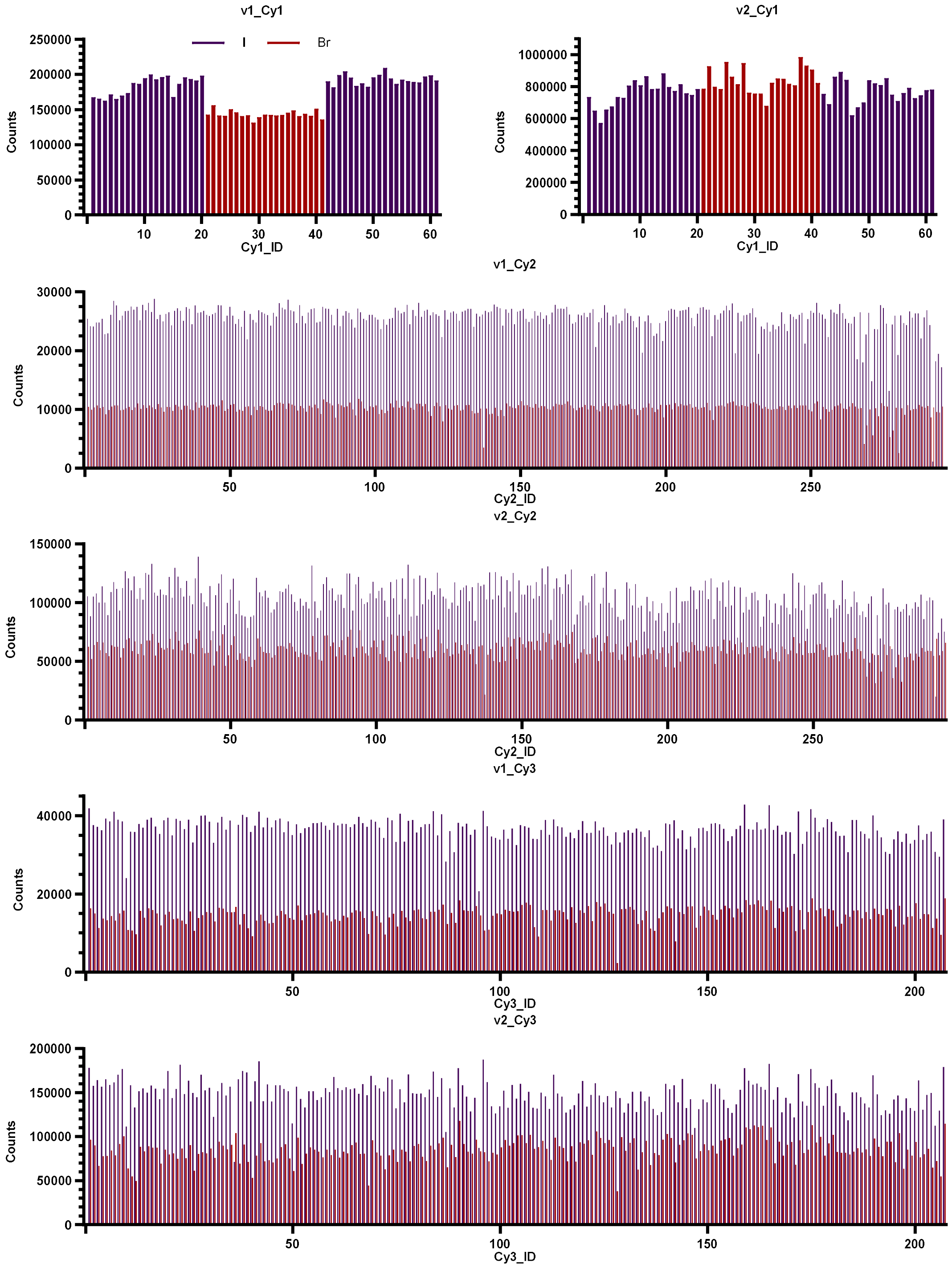

Supplementary Fig. 19 | DOSEDO v1 and v2 input analysis aggregating on cycle 1, 2 or 3 tags. Ordered as plated during synthesis. Purple, iodide-derived sub-library; orange, bromide-derived sub-library.

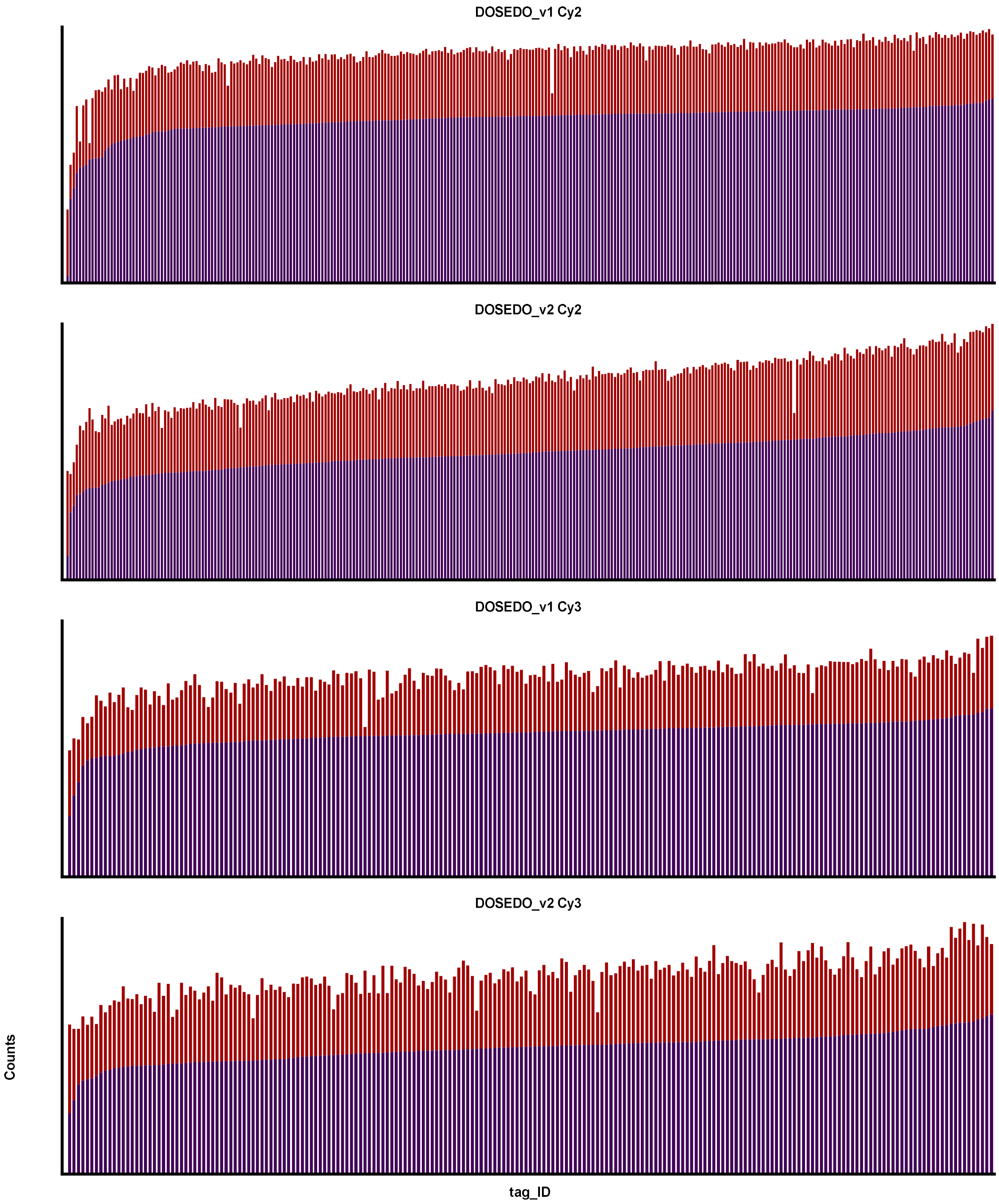

Supplementary Fig. 20 | Comparison of DOSEDO v1 and v2 cycle 2 and 3 tag ligation product representation**.** Sorted from least to most represented in the iodide-derived sub-library (purple) with corresponding aggregated count for the bromide-derived sub-library stacked (orange).

4. DEL screening – Supplementary Figures

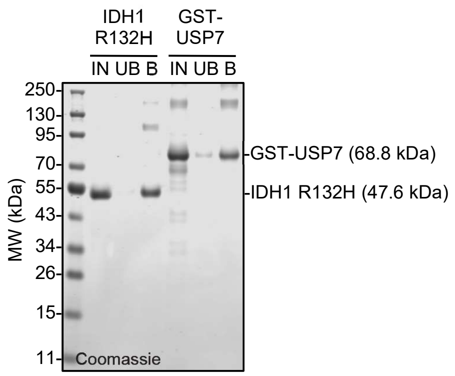

Supplementary Fig. 21 | SDS-PAGE gel to access protein immobilization to the beads during the DEL screen. IDH1 R132H and GST-USP7 fully bound the beads with little unbound. IN: input, 1.2 μM protein; UB: unbound, protein remaining in the supernatant after incubation of protein and beads; B: bound, beads after the screen heated and treated with NuPAGE™ LDS Sample Buffer to release the protein that bound the beads. All lanes are comparable.

5. Assay details – Supplementary Figures and Tables

Supplementary Table 10 | Summary of carbonic anhydrase inhibition data obtained according to method highlighted above

cpd_id	beads_ppm	caix_ppm	er	er_lb	IC50, nM
**59**	2	2248	1020	504	16
**45**	0	11	70	9	26
**46**	2	0	0	0	279
**52**	3	0	0	0	64
**54**	0	9	58	8	34
**56**	0	207	1348	198	2718
**57**	0	264	1716	253	31
**58**	0	922	5997	886	17
**53**	4	0	0	0	> 10000

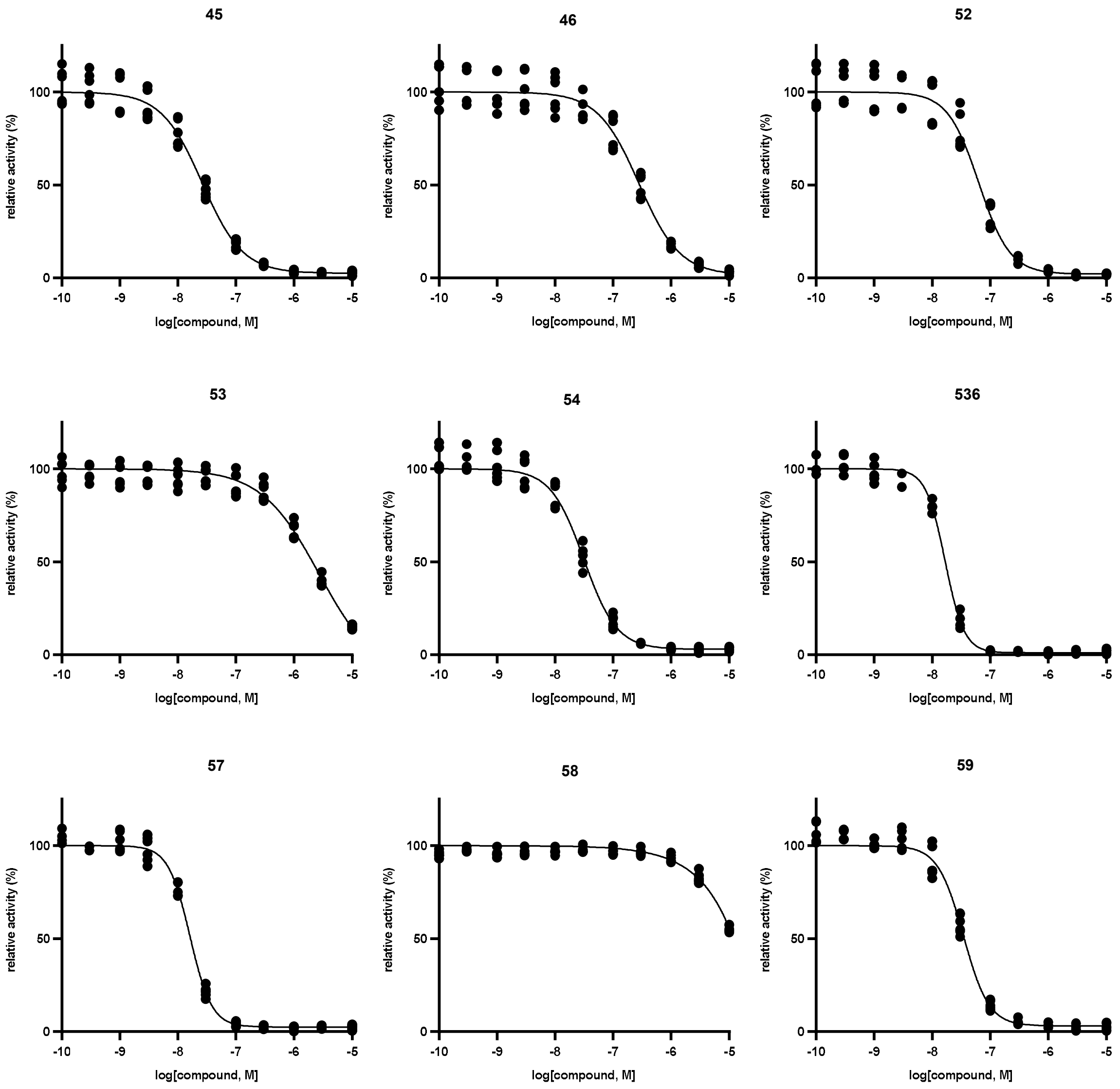

Supplementary Fig. 22 | Carbonic anhydrase dose-response curves generated as described in the Methods section. For each graph, n = 2 biologically independent samples, with 3 technical replicates per sample, with the exception of compound **53**, where n = 1 biologically independent sample, with 6 technical replicates per sample. Source data are provided as a Source Data file.

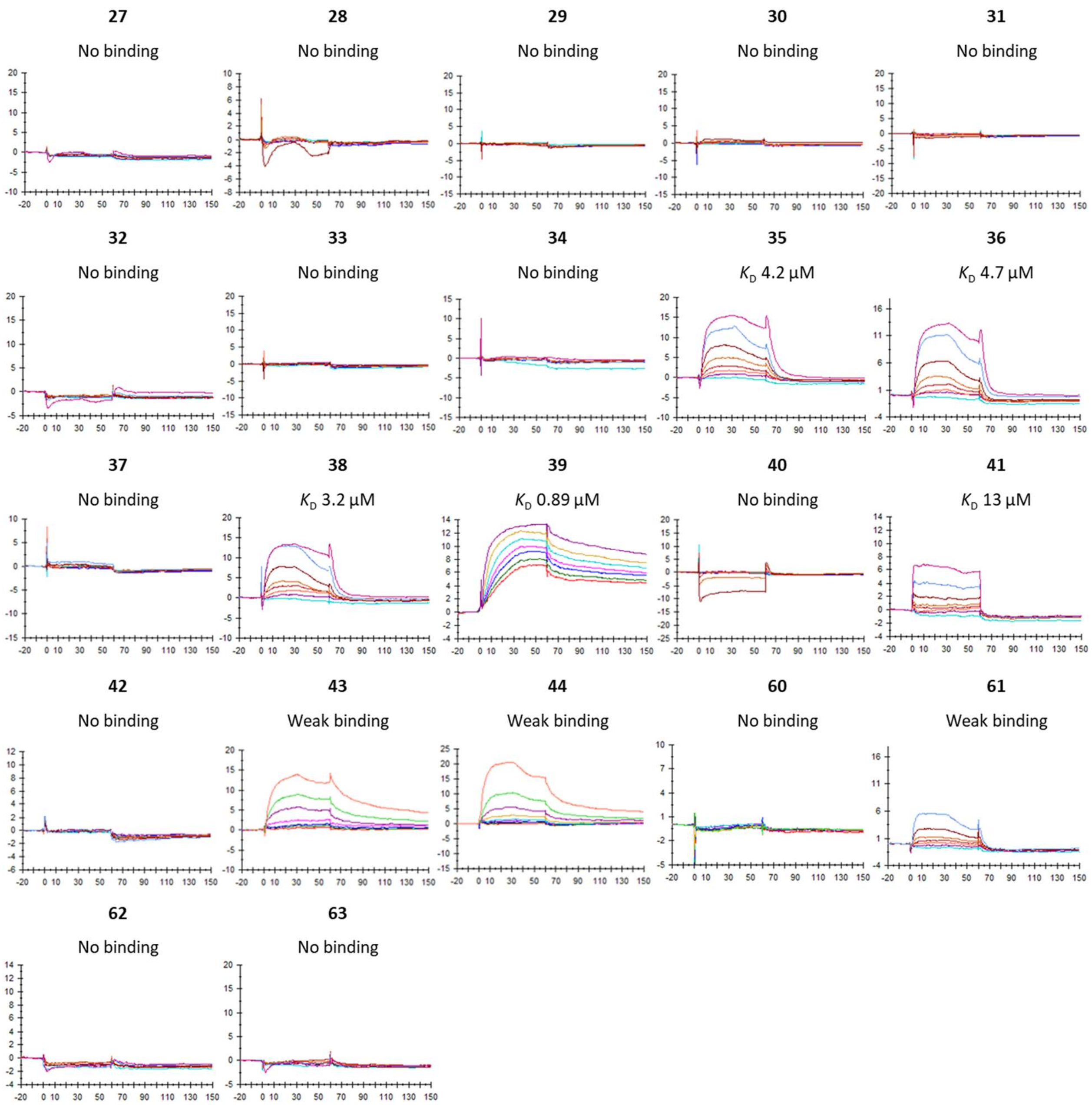

Supplementary Fig. 23 | SPR sensograms of off-DNA DEL compounds binding to biotinylated IDH1 R132H. The binding results were shown with K_D values, weak binding (indicating signs of binding but KD >20 µM) or no binding at the tested concentrations.

3 binding to GST-tagged USP7

K_D 7.7 μM

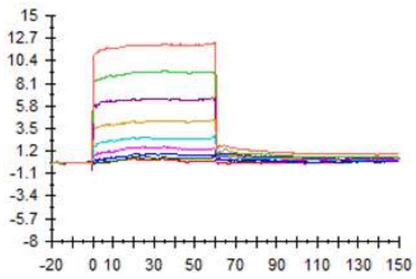

3 binding to GST

K_D 10 μM

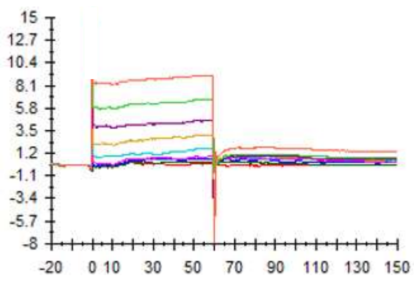

Supplementary Fig. 24 **|** SPR sensograms of compound **3** binding to GST-tagged USP7 and GST with KDs of 7.7 μM and 10 μM, respectively.

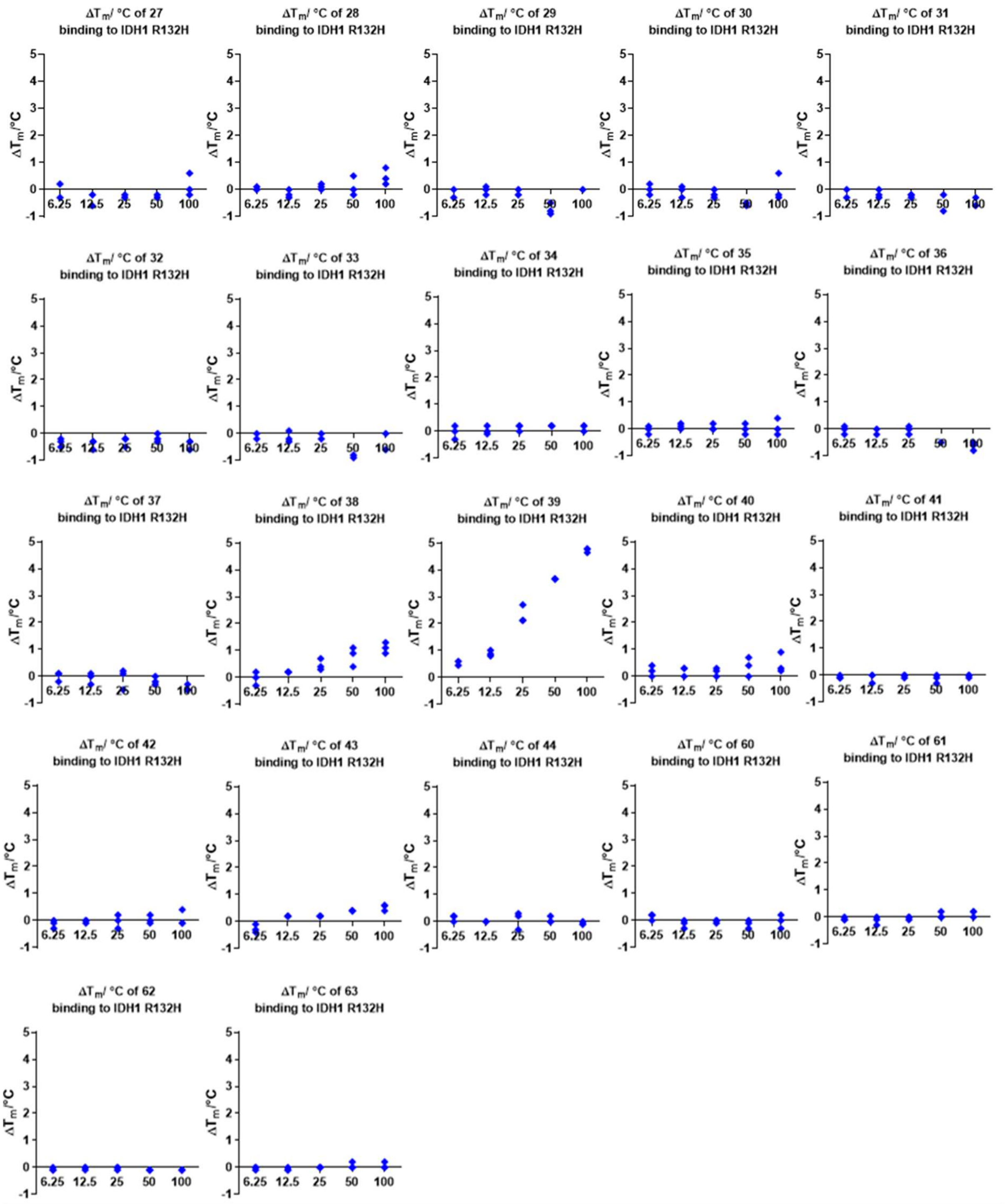

Supplementary Fig. 25 | DSF results for off-DNA DOSEDO DEL compounds with IDH1 R132H in 50 mM Tris-HCl, pH 7.4. x-axis is concentration of names compound in all cases, in μM. Compound **39** induces thermal stabilization of IDH1 R132H in a dose dependent manner, up to 4.7 °C at 100 μM. All other

compounds showed no or weak thermal stabilization of IDH1 R132H. N = 3 technical replicates. Source data are provided as a Source Data file.

a

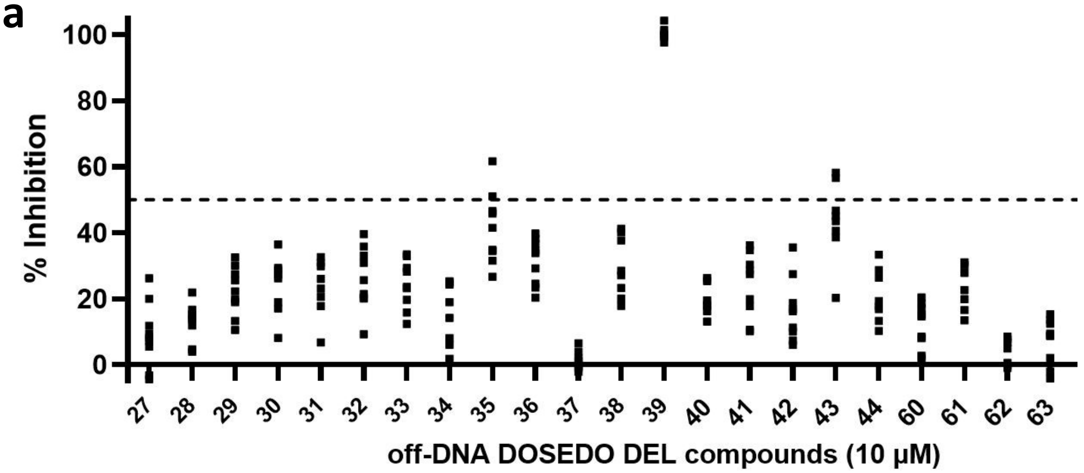

b

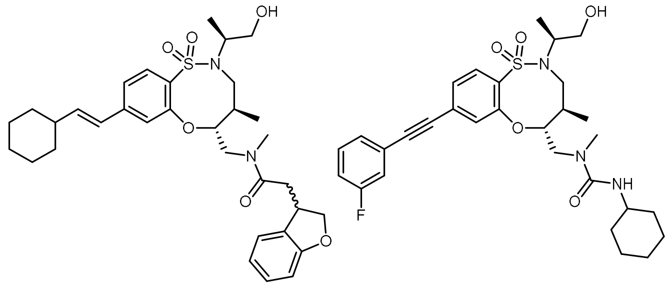

39 BRD2879

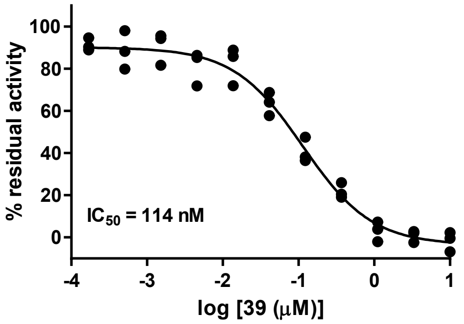

Supplementary Fig. 26 | Summary of IDH1 R132H inhibition assays**. a.** % inhibition of IDH1 R132H catalyzed conversion of 2OG to 2HG by off-DNA DOSEDO DEL compounds, as measured by absorbance assays. Compound **39** fully inhibits IDH1 R132H at 10 µM while all other compounds have an IC50 of >10 µM. All compounds have been tested for IC_{50} with 3-fold serial dilution from 10 µM to confirm their weak (except **39**) inhibitory activity at all concentrations below 10 µM. Data are mean ± SEM, n= 9 consisting of 3 technical replicates from 3 independent replicates; **b.** Structure of compound **39** and BRD2879 alongside IC_{50} of compound **39** against IDH1 R132H, as measured by absorbance assays. N = 3 technical replicates. Source data are provided as a Source Data file.

6. In silico – Supplementary Figures

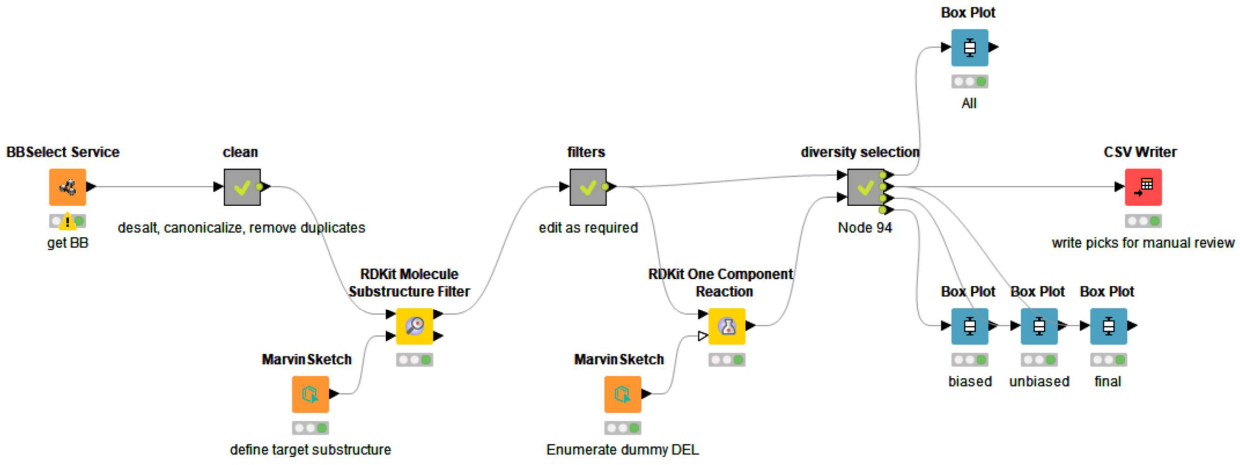

Supplementary Fig. 27 | Overview of building block selection tool. “BBSelect Service” is a tool internal to Novartis allowing for rapid searching of multiple curated building block catalogs, internally available materials, as well as commercial building block sources.

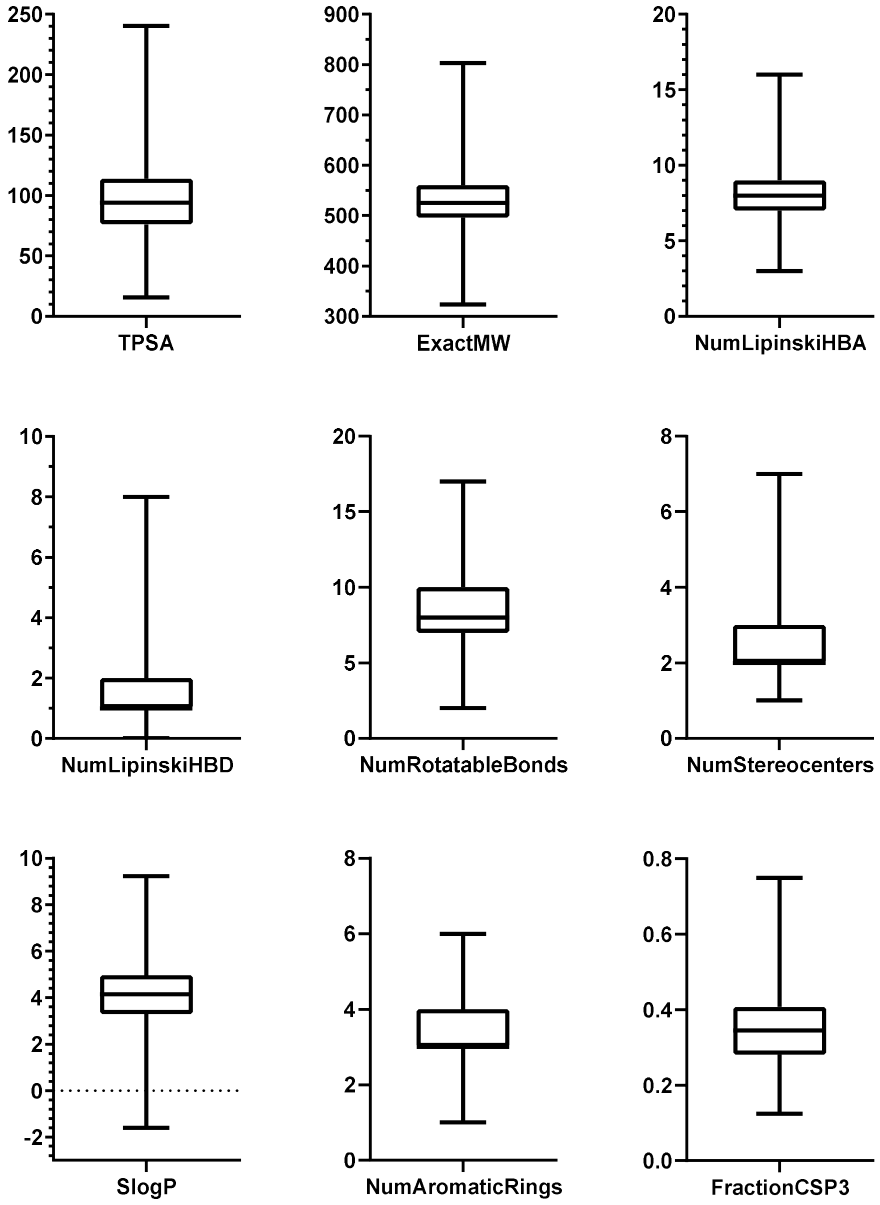

Supplementary Fig. 28 | Box and whisker plots for selected calculated properties of the entire enumerated DOSEDO library. Central bars show the median value, the box encapsulates the median 50% of compounds, and the whiskers show the upper and lower quartiles up to and including the maximum and minimum values for each property. N = 3,688,975 unique compounds.

7. Small molecule NMR data

7.1. β-arylated cyclic amino acids

(2*R*,3*S*)-1-(((9H-fluoren-9-yl)methoxy)carbonyl)-3-(3-iodophenyl)azetidine-2-carboxylic acid (**6a**)

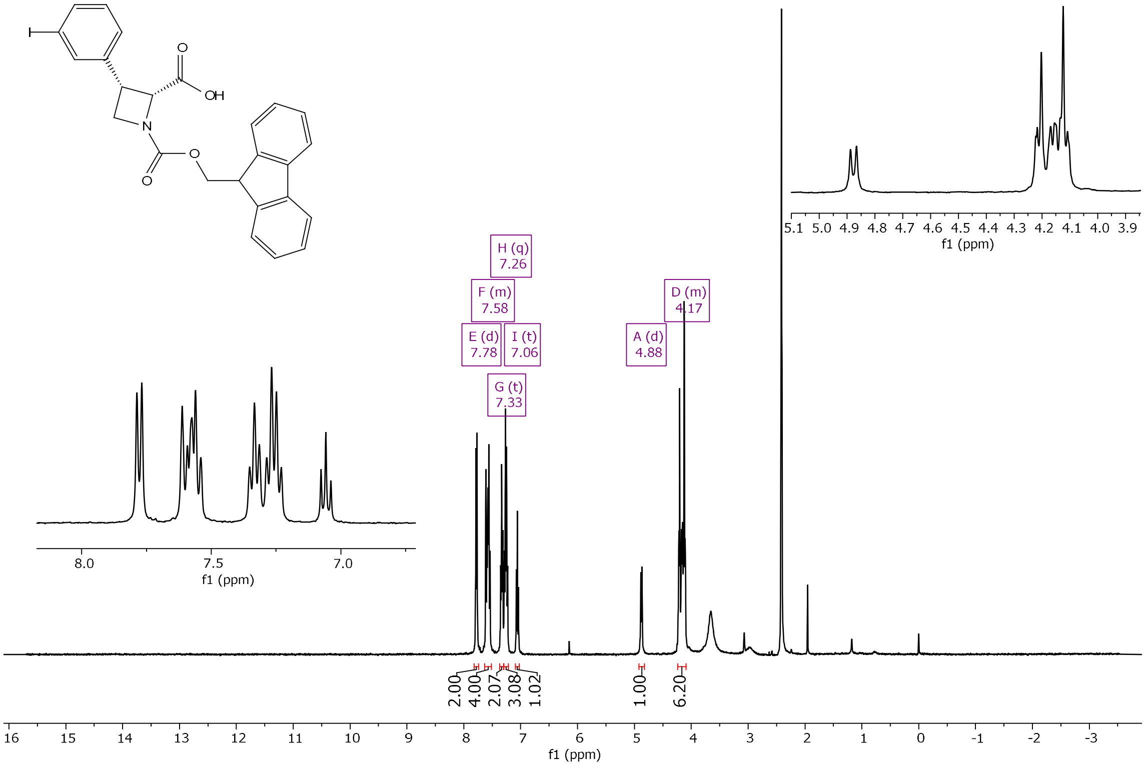

1, 2, 3 and 4 are spectra collected at 25, 40, 60 and 80°C, respectively:

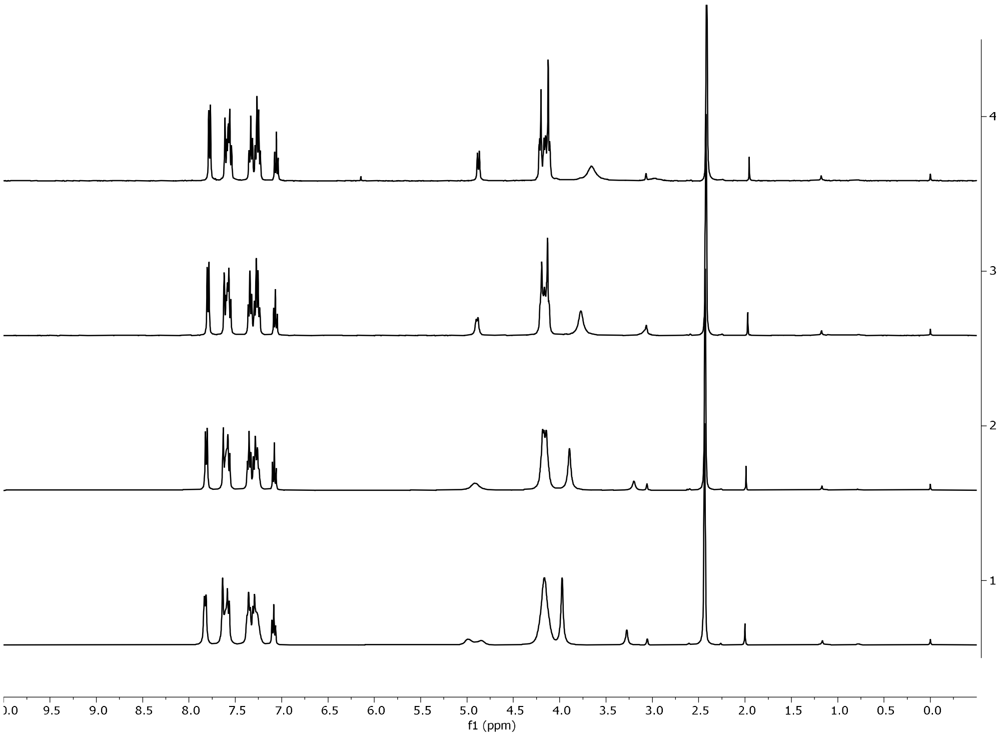

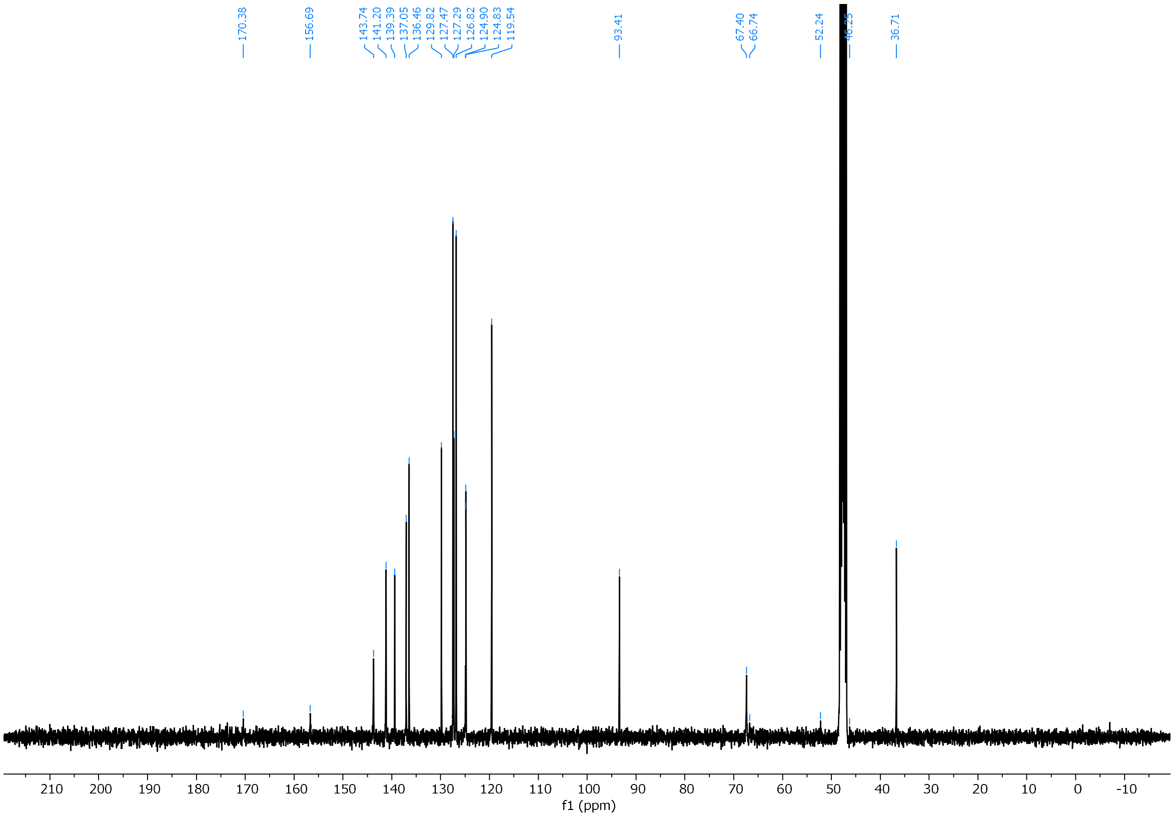

(2*S*,3*R*)-1-(((9H-fluoren-9-yl)methoxy)carbonyl)-3-(3-iodophenyl)azetidine-2-carboxylic acid (**6b**)

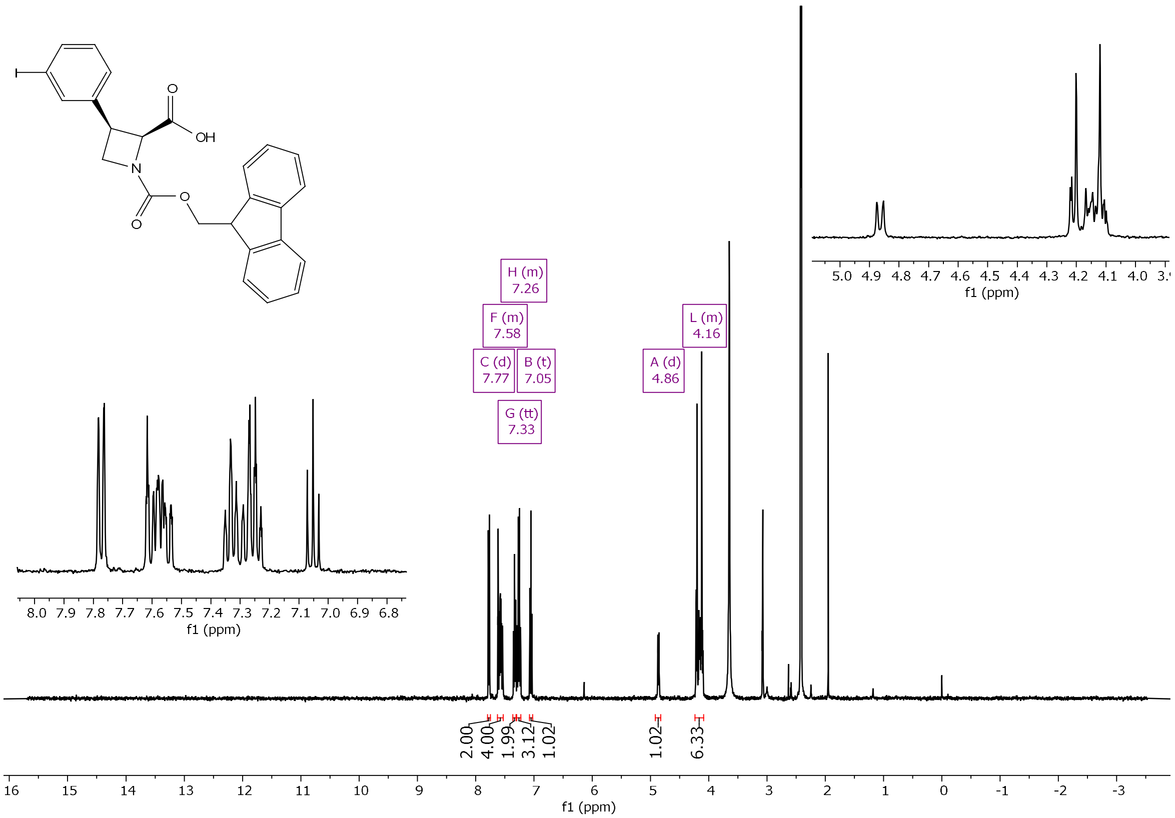

1, 2, 3 and 4 are spectra collected at 25, 40, 60 and 80°C, respectively:

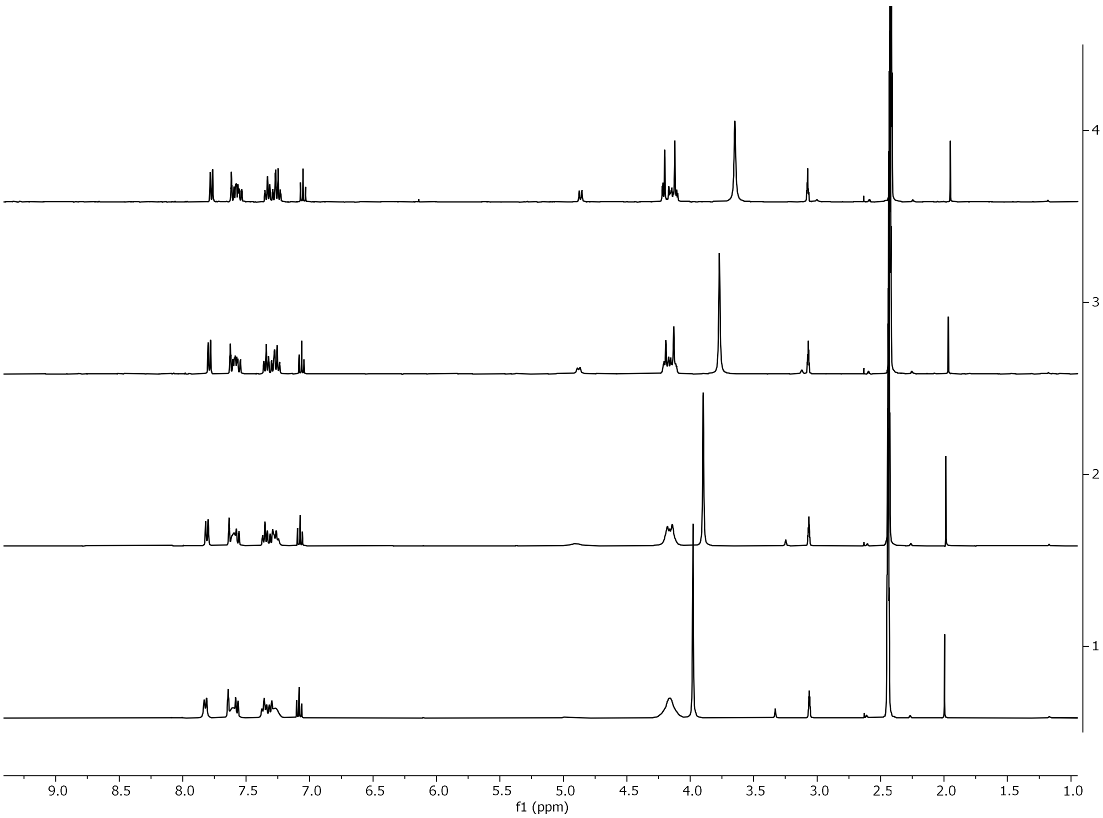

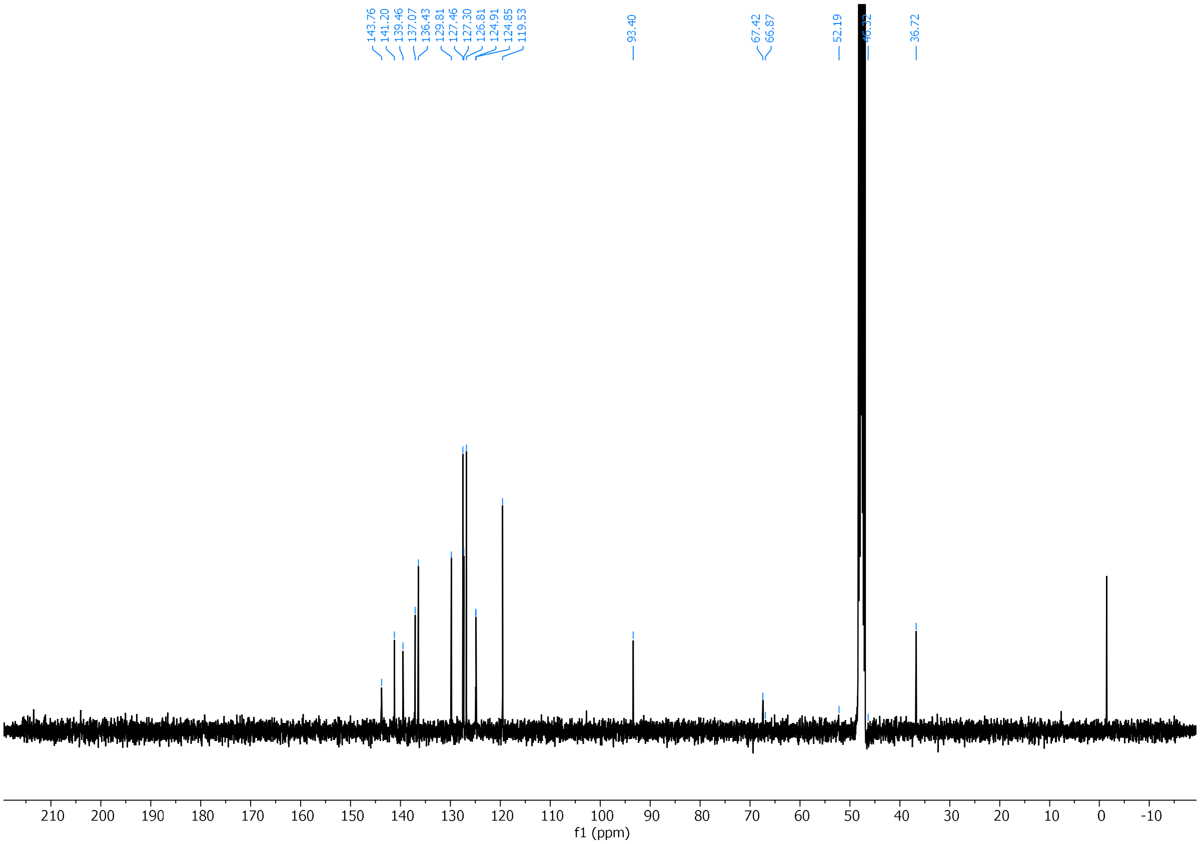

(2*R*,3*R*)-1-(((9H-fluoren-9-yl)methoxy)carbonyl)-3-(3-iodophenyl)pyrrolidine-2-carboxylic acid (**6c**)

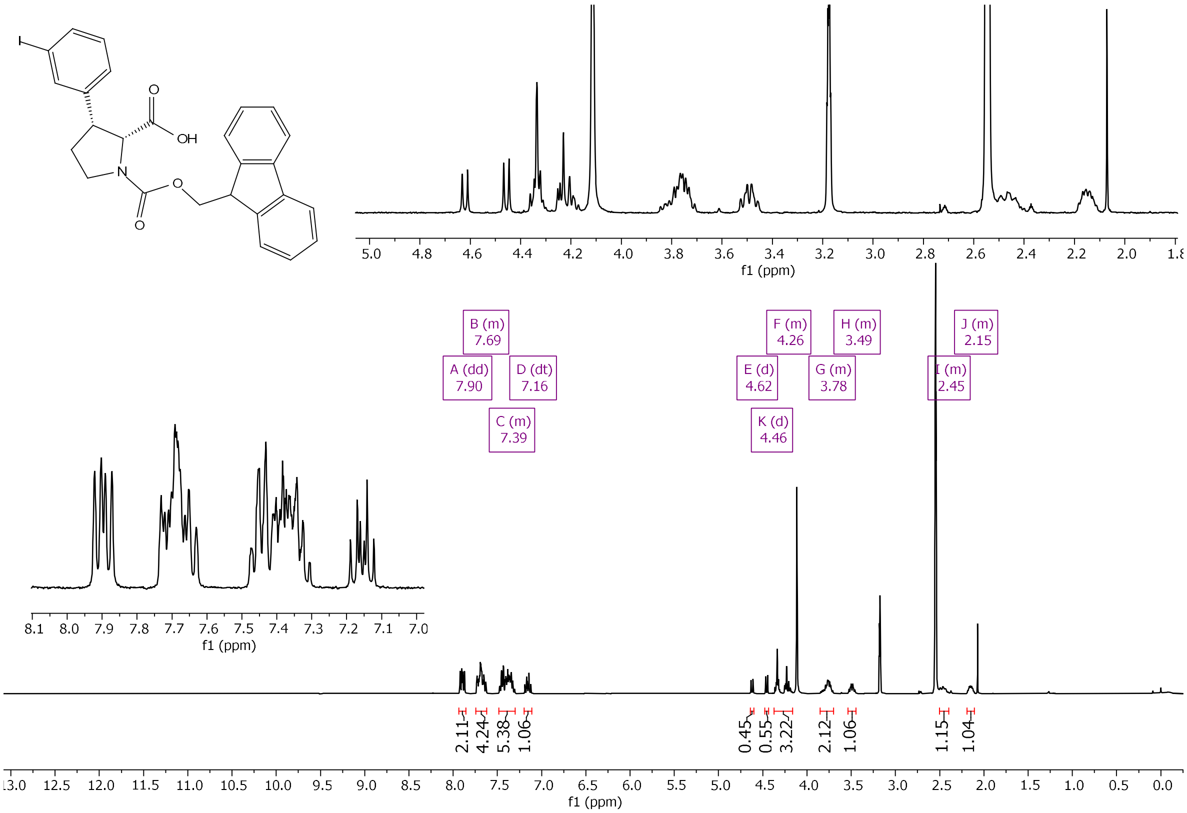

1, 2, 3 and 4 are spectra collected at 25, 40, 60 and 80°C, respectively:

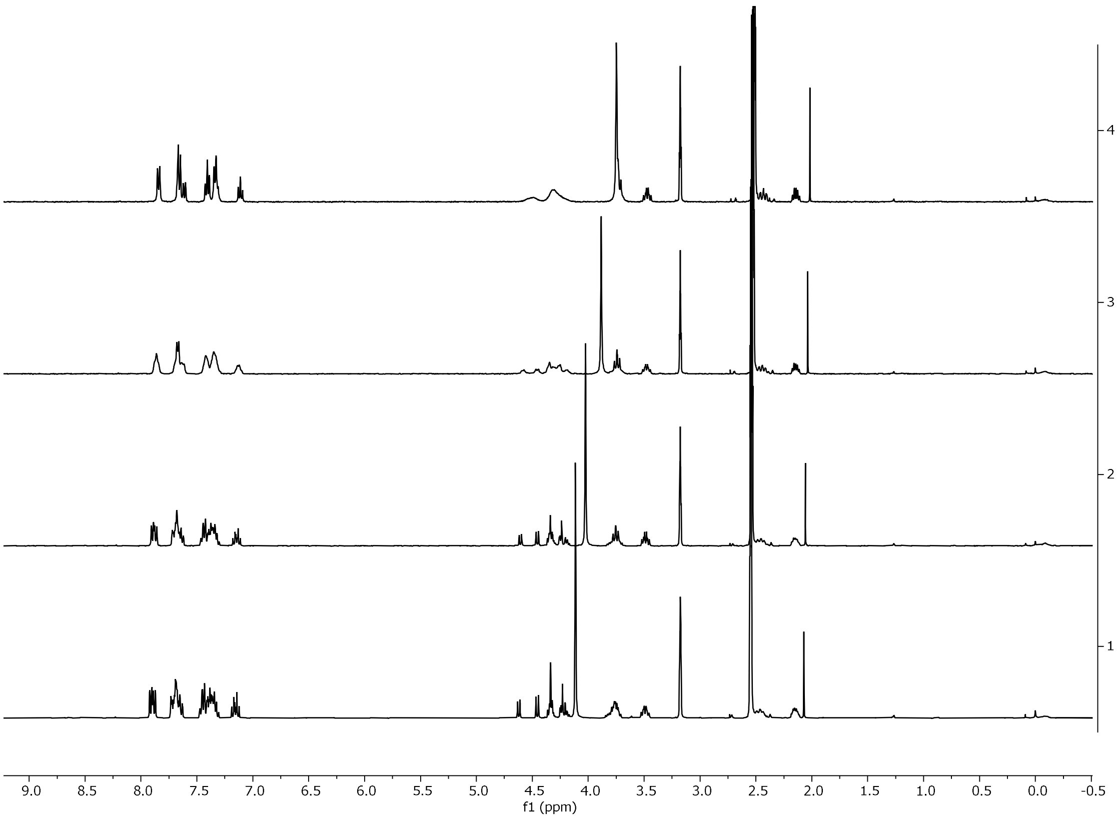

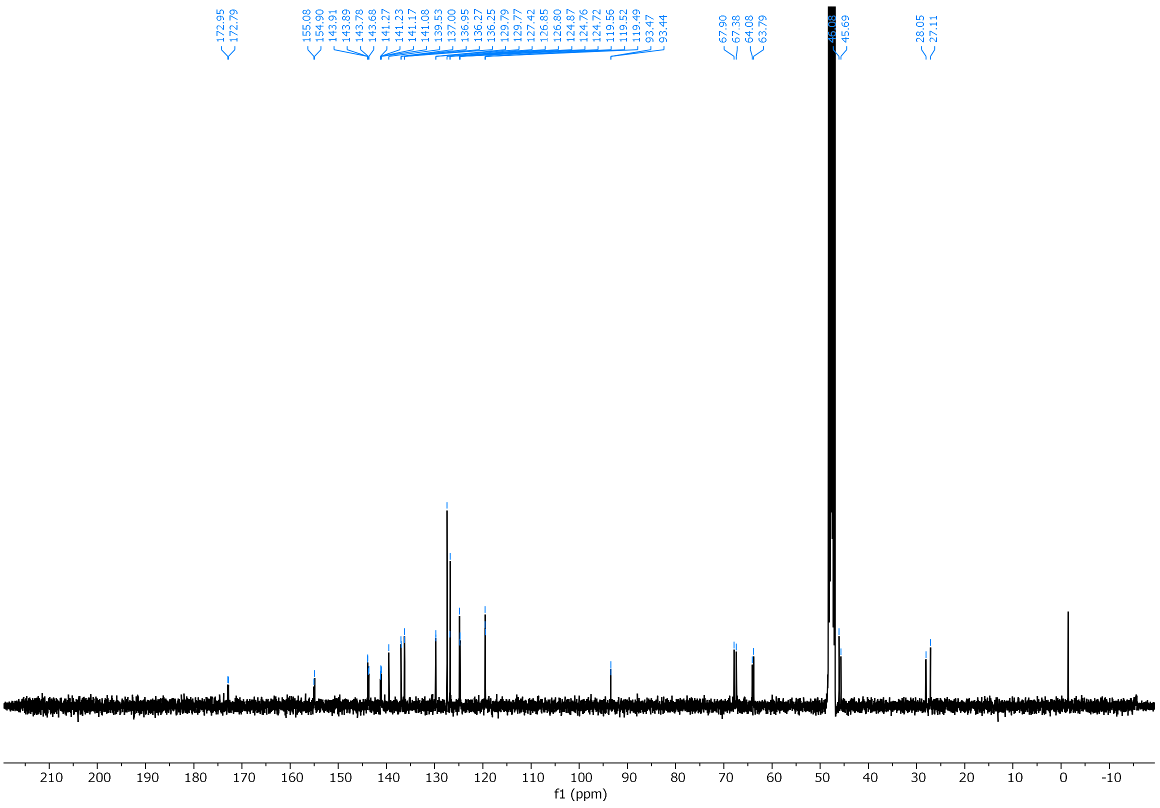

(2*S*,3*S*)-1-(((9H-fluoren-9-yl)methoxy)carbonyl)-3-(3-iodophenyl)pyrrolidine-2-carboxylic acid (**6d**)

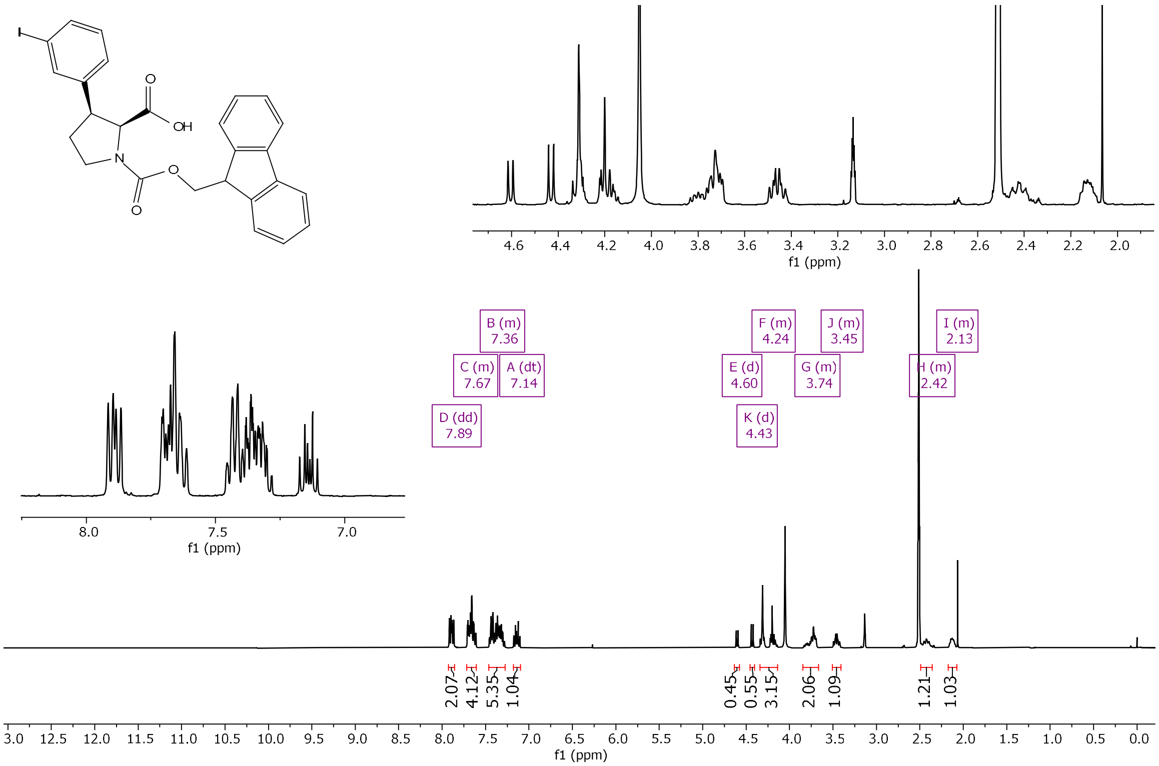

1, 2, 3 and 4 are spectra collected at 25, 40, 60 and 80°C, respectively:

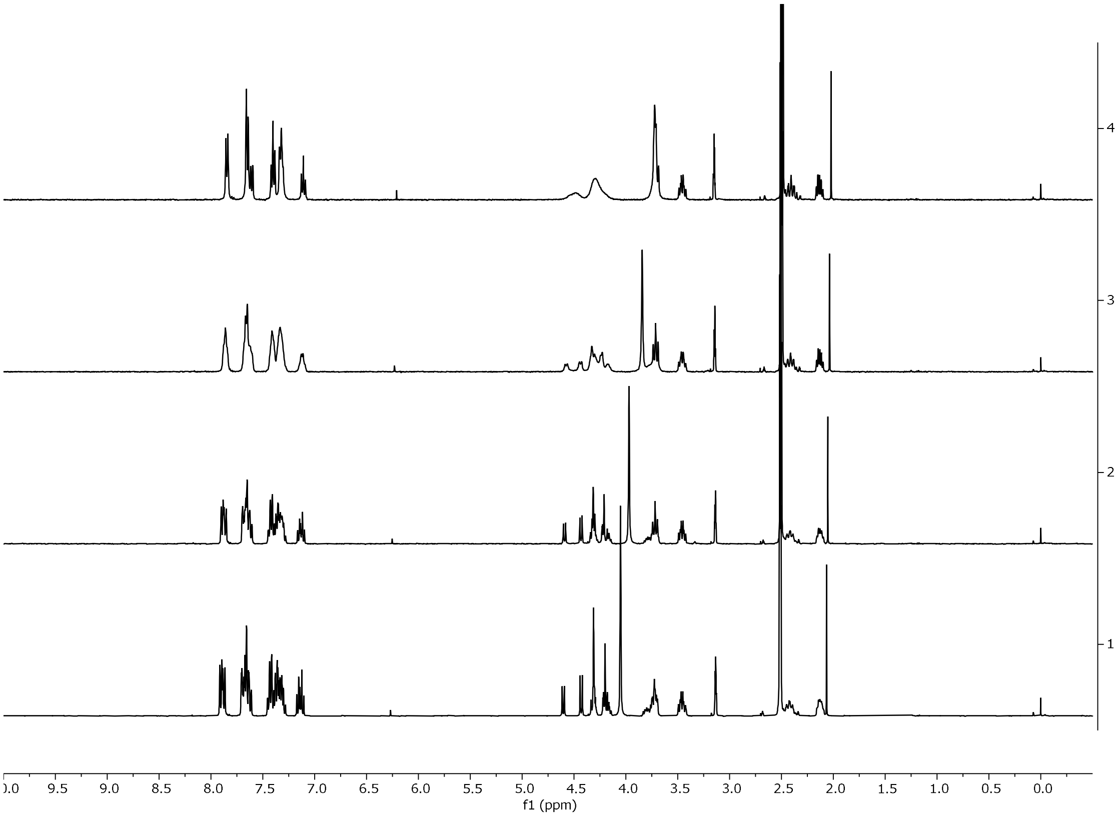

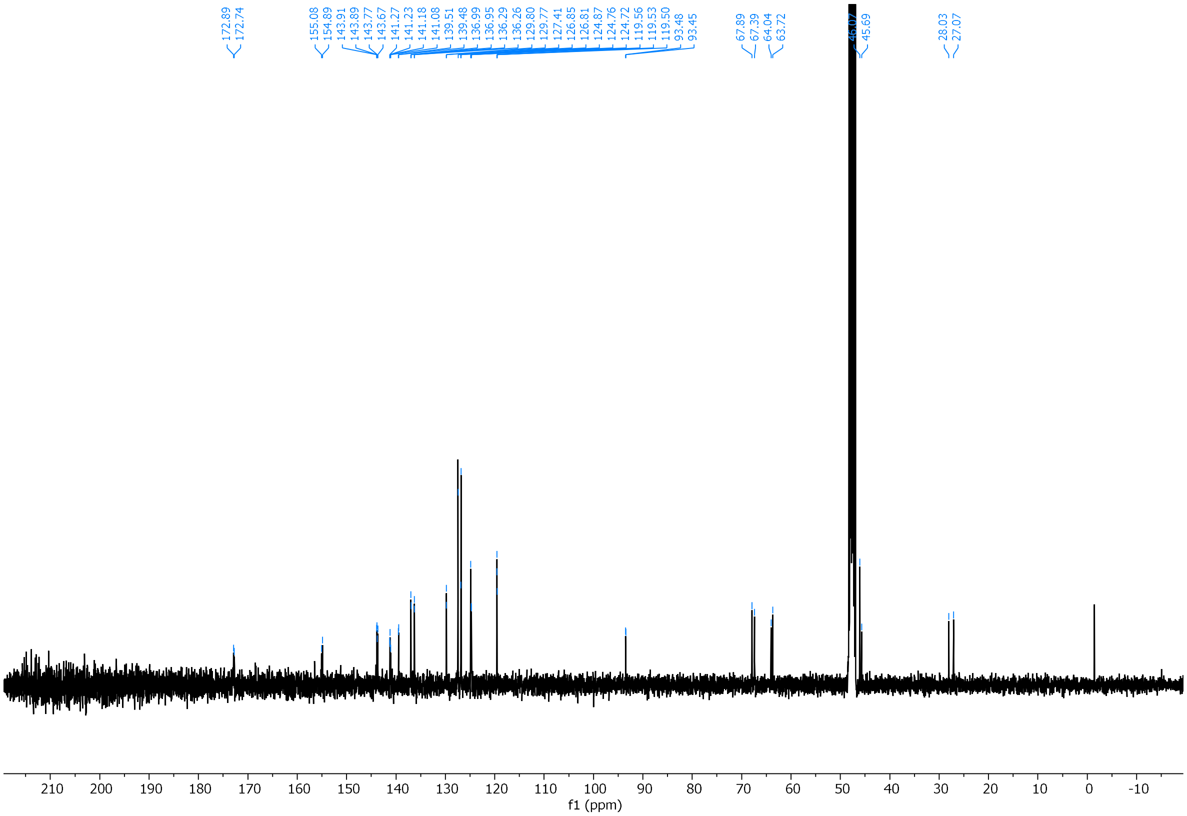

(2*R*,3*R*)-1-(((9H-fluoren-9-yl)methoxy)carbonyl)-3-(3-iodophenyl)piperidine-2-carboxylic acid (**6e**)

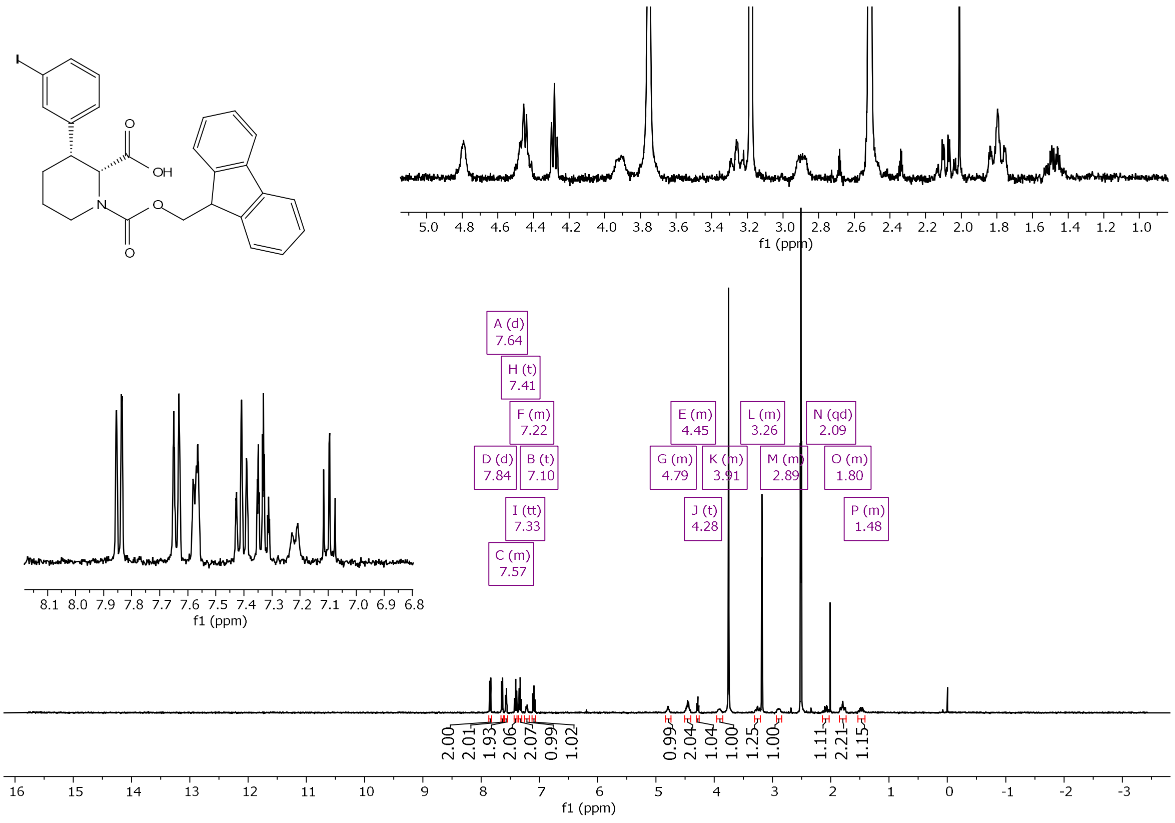

1, 2, 3 and 4 are spectra collected at 25, 40, 60 and 80°C, respectively:

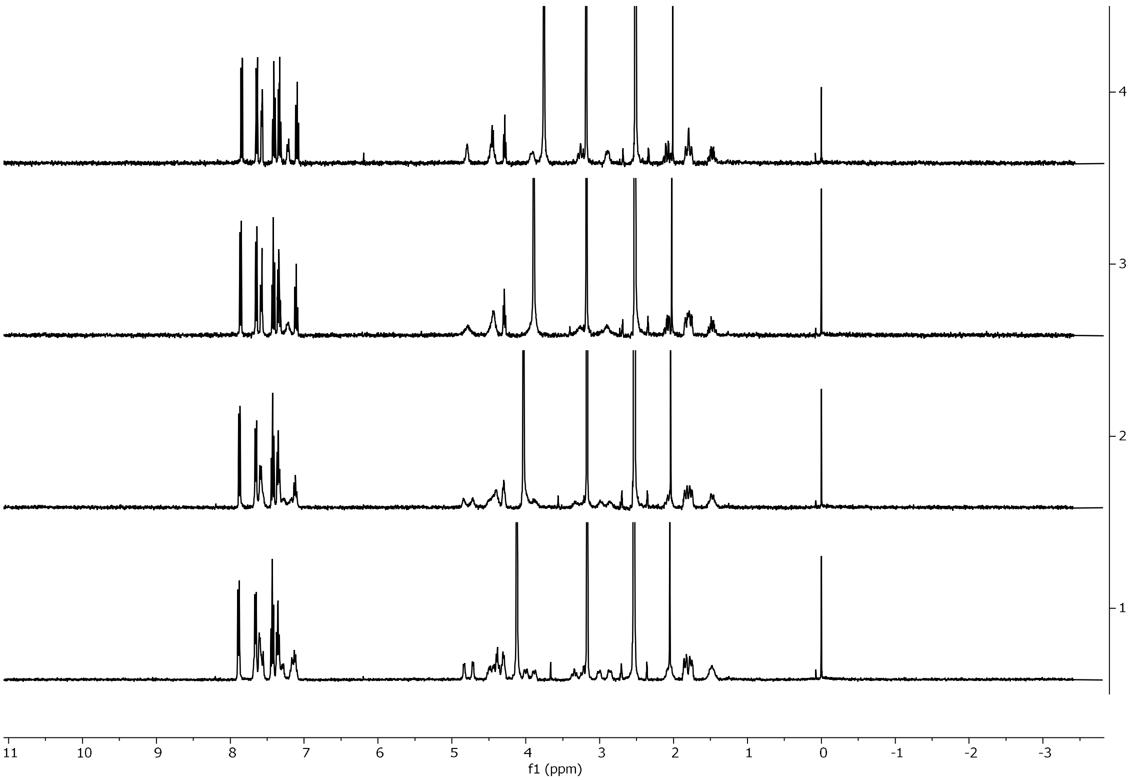

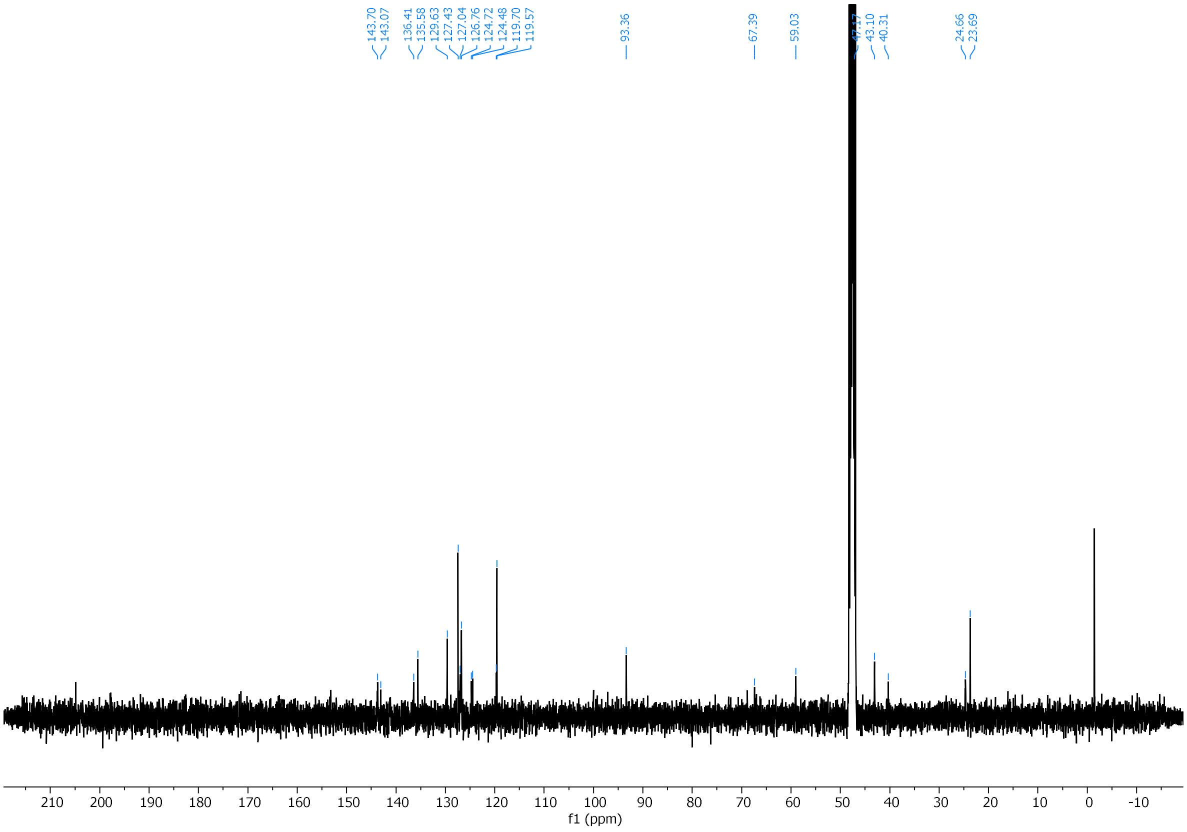

Overlay of HSQC and HMBC (rt in CD_3OD):

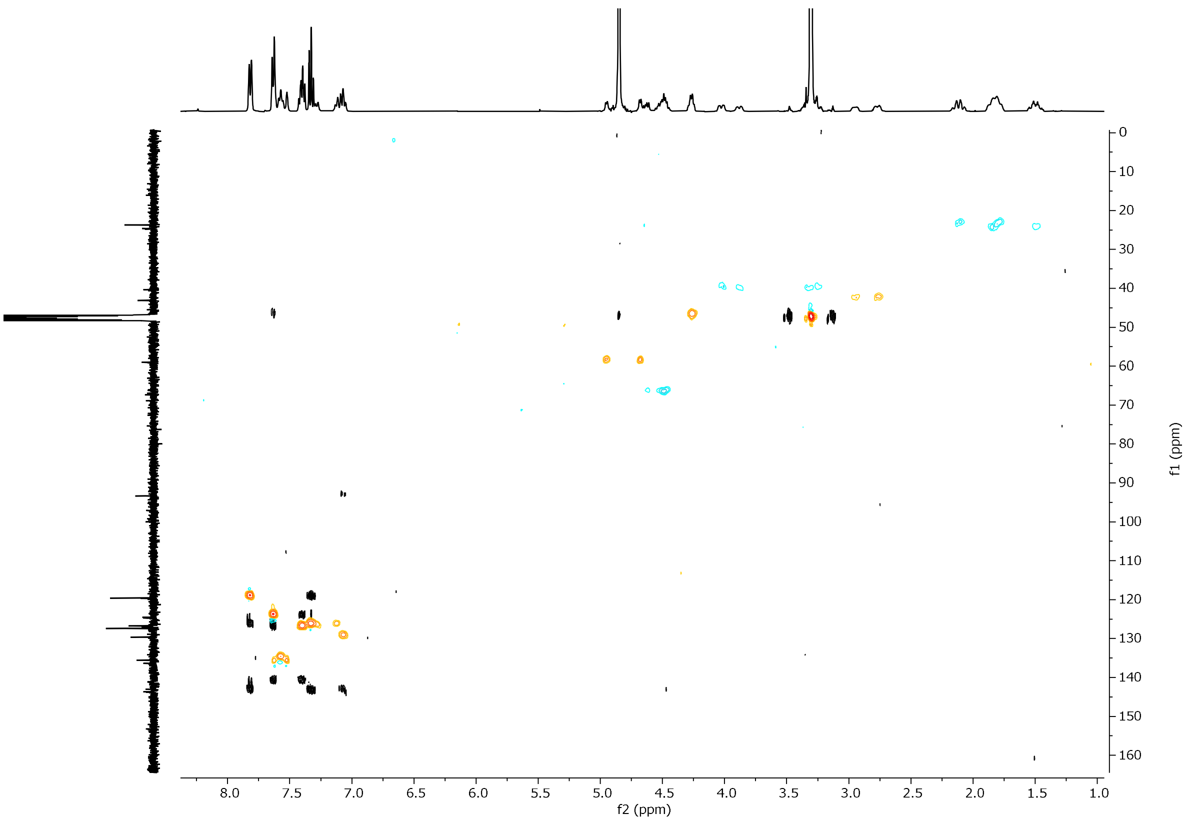

(2*S*,3*S*)-1-(((9H-fluoren-9-yl)methoxy)carbonyl)-3-(3-iodophenyl)piperidine-2-carboxylic acid (**6f**)

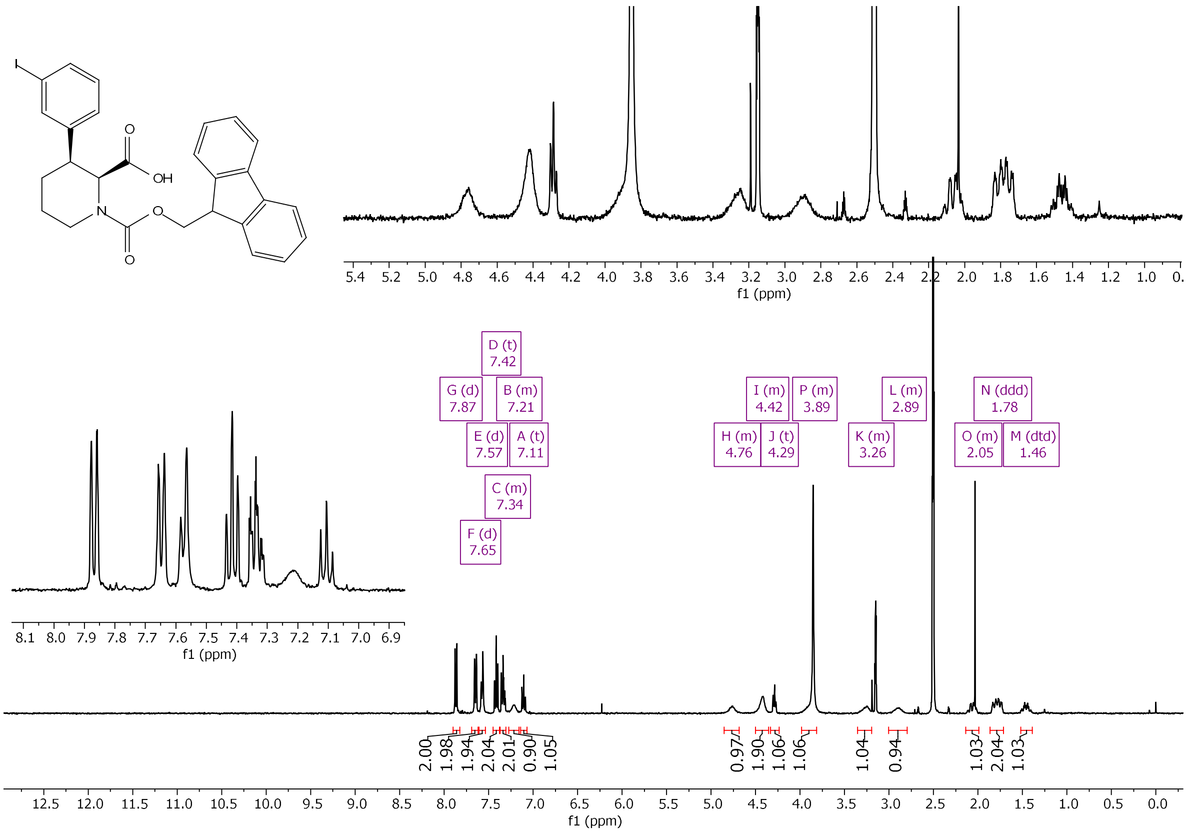

1, 2, 3, 4 and 5 are spectra collected at 25, 40, 60, 80, and then again at 25°C in sequence, respectively:

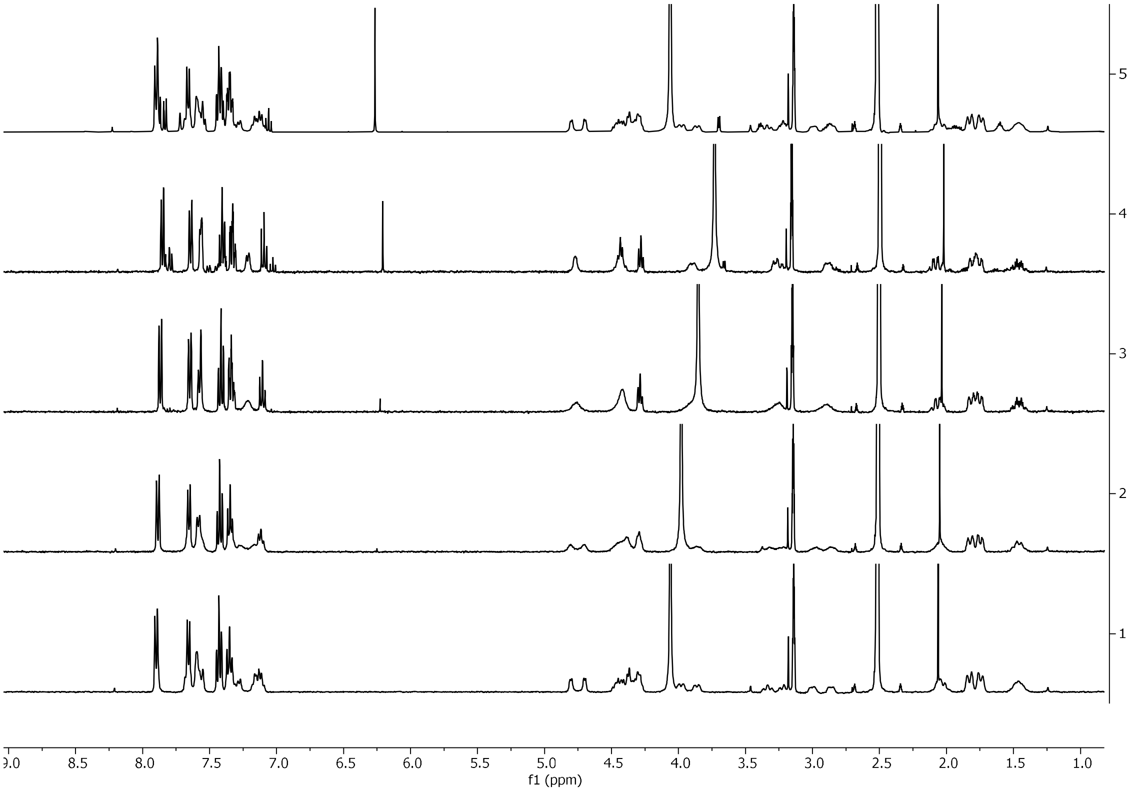

5
4
3
2
1
9.0 8.5 8.0 7.5 7.0 6.5 6.0 5.5 5.0 4.5 4.0 3.5 3.0 2.5 2.0 1.5 1.0
f1 (ppm)

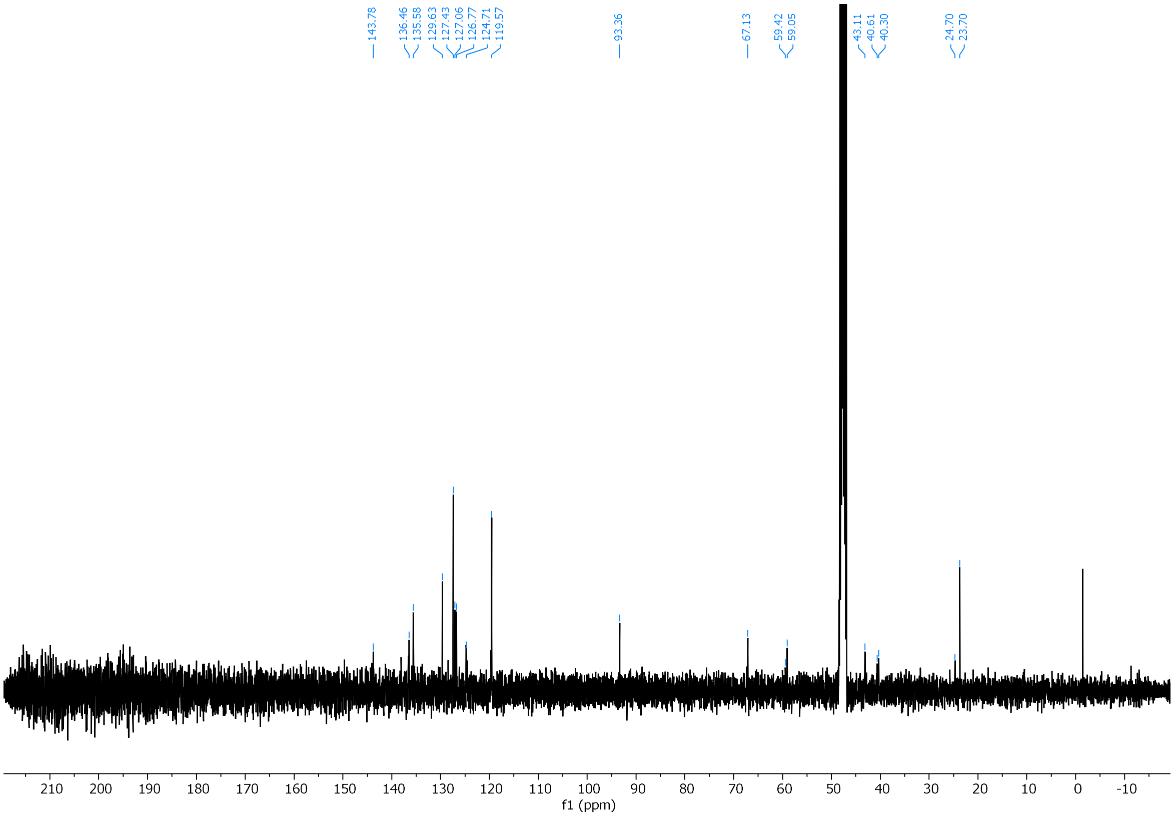

143.78
136.46
135.58
129.63
127.43
127.06
126.77
124.71
119.57
93.36
67.13
59.42
59.05
43.11
40.61
40.30
24.70
23.70
210 200 190 180 170 160 150 140 130 120 110 100 90 80 70 60 50 40 30 20 10 0 -10
f1 (ppm)

Overlay of HSQC and HMBC (rt in CD_3OD):

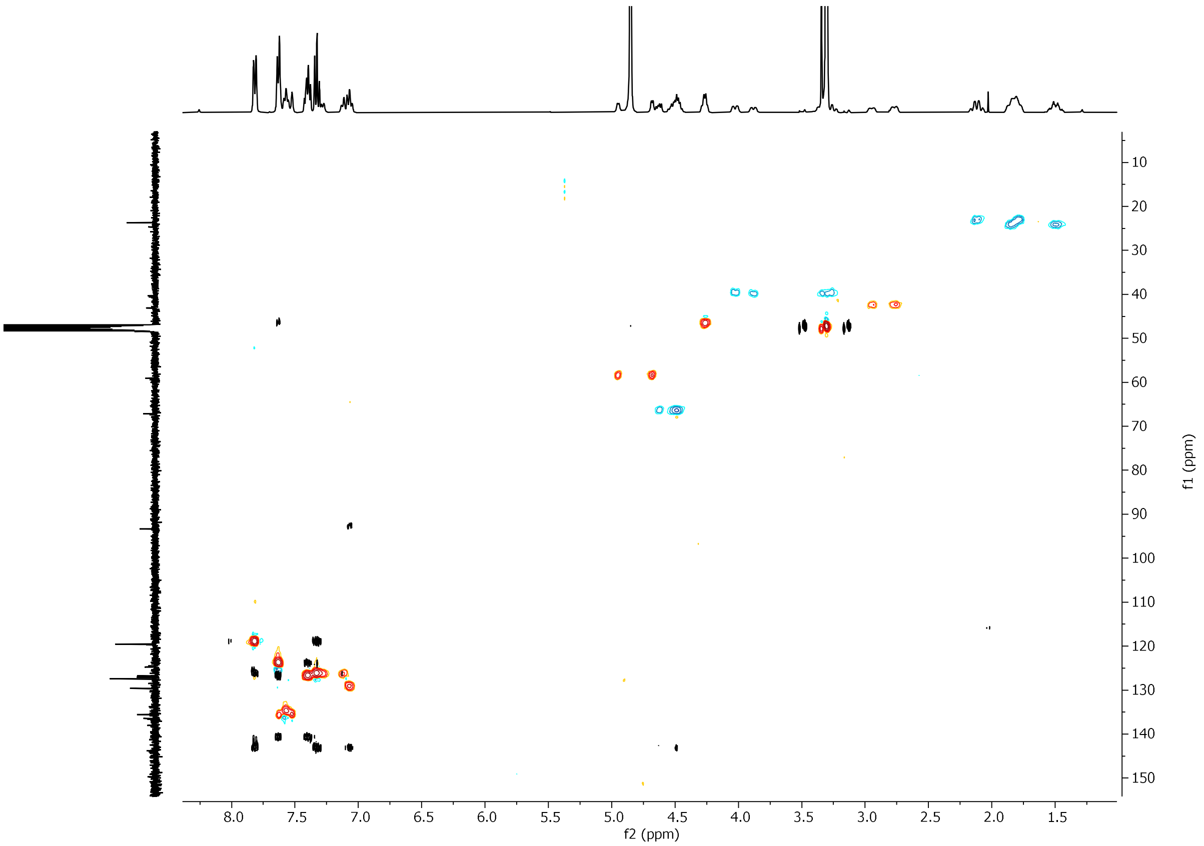

(2*S*,3*S*)-1-(((9H-fluoren-9-yl)methoxy)carbonyl)-3-(3-iodophenyl)azetidine-2-carboxylic acid (**7a**)

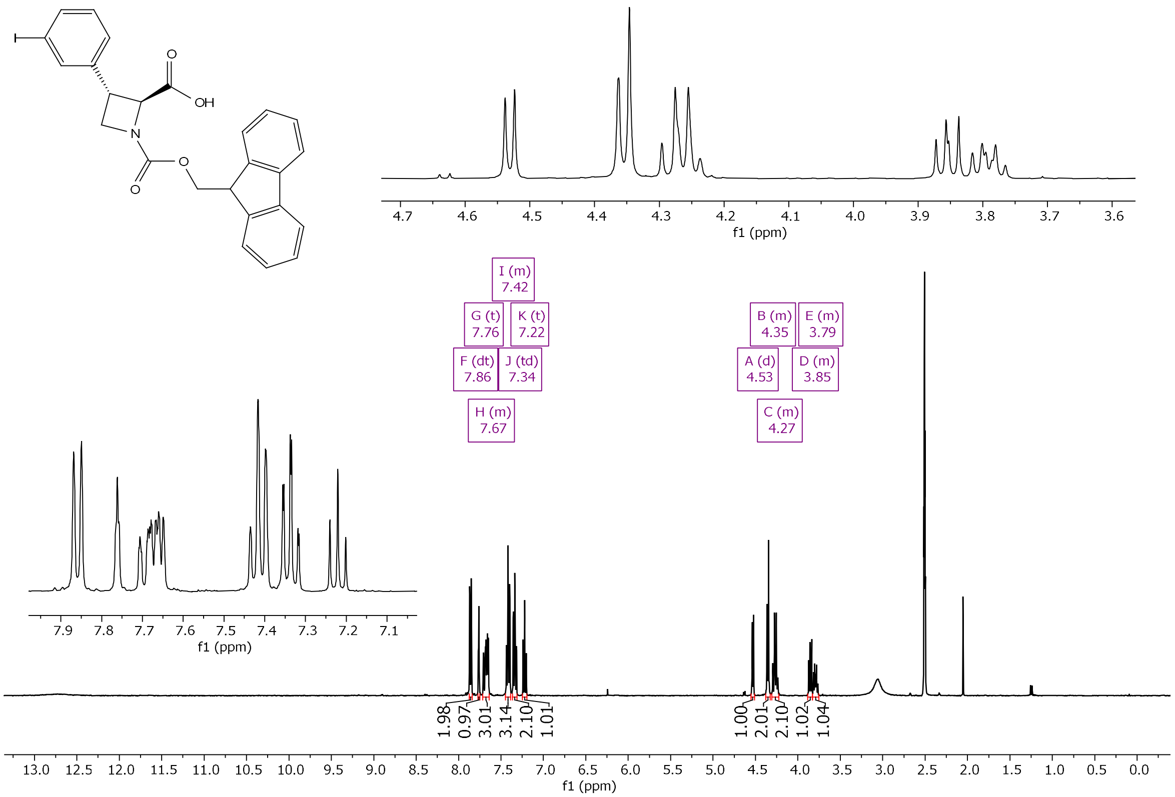

1, 2, 3 and 4 are spectra collected at 25, 40, 60 and 80°C, respectively:

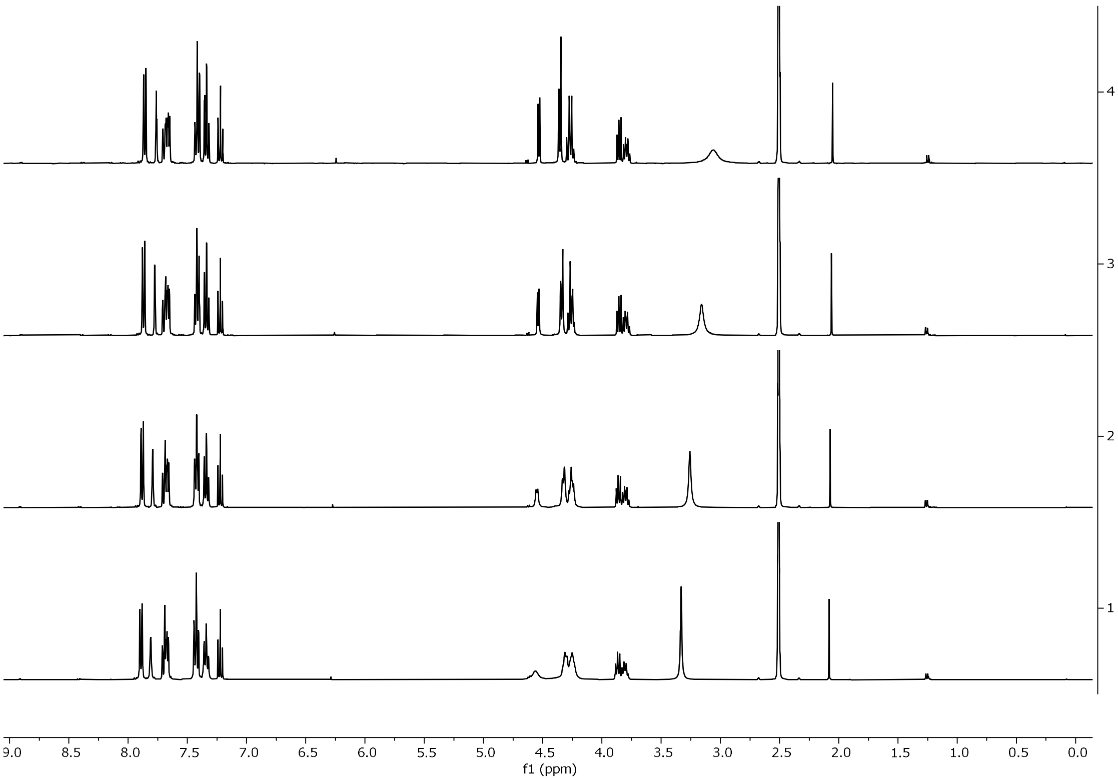

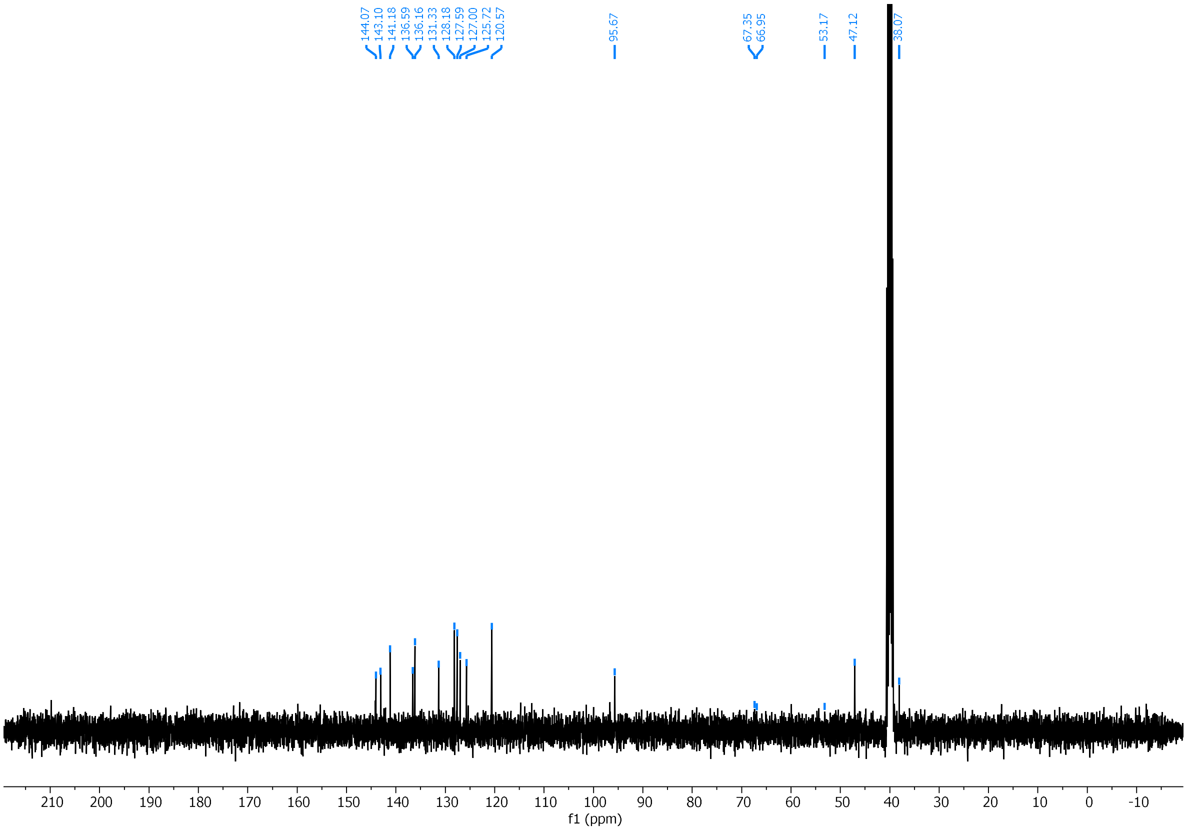

Overlay of HSQC and HMBC (rt in DMSO):

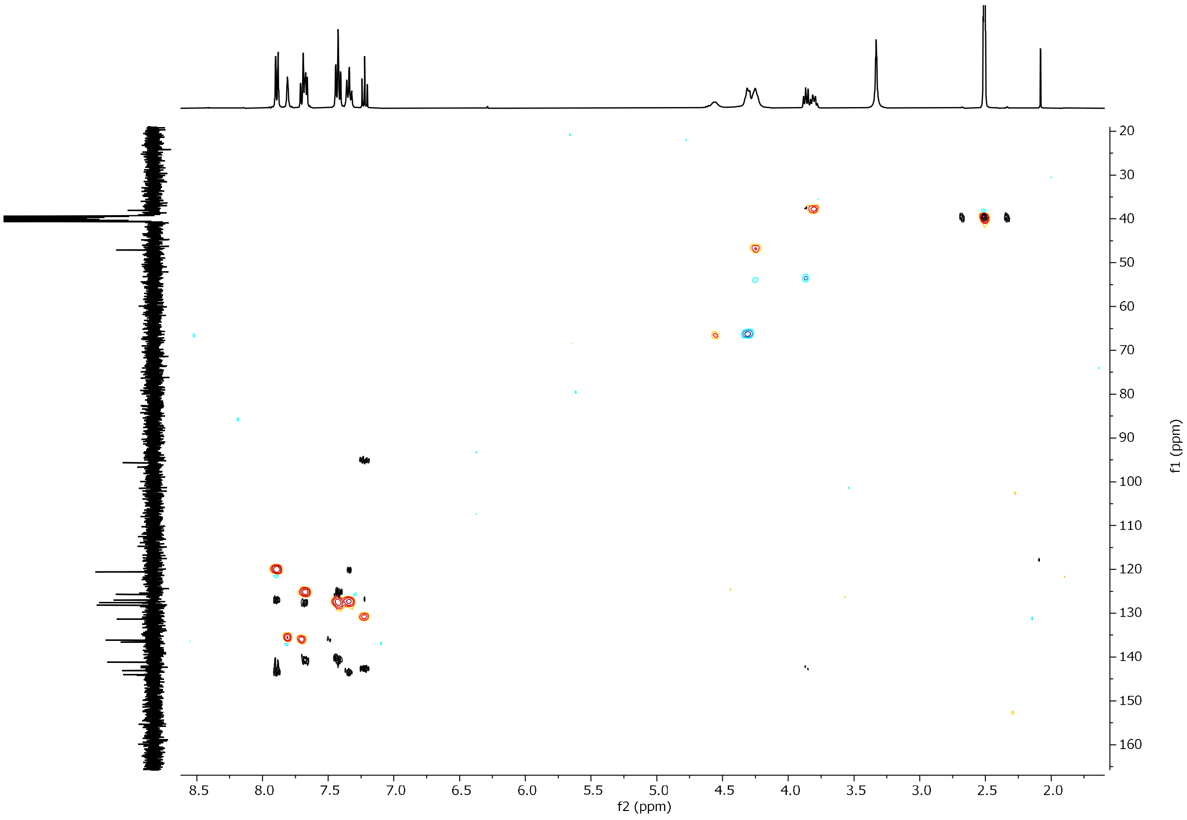

(2*R*,3*R*)-1-(((9H-fluoren-9-yl)methoxy)carbonyl)-3-(3-iodophenyl)azetidine-2-carboxylic acid (**7b**)

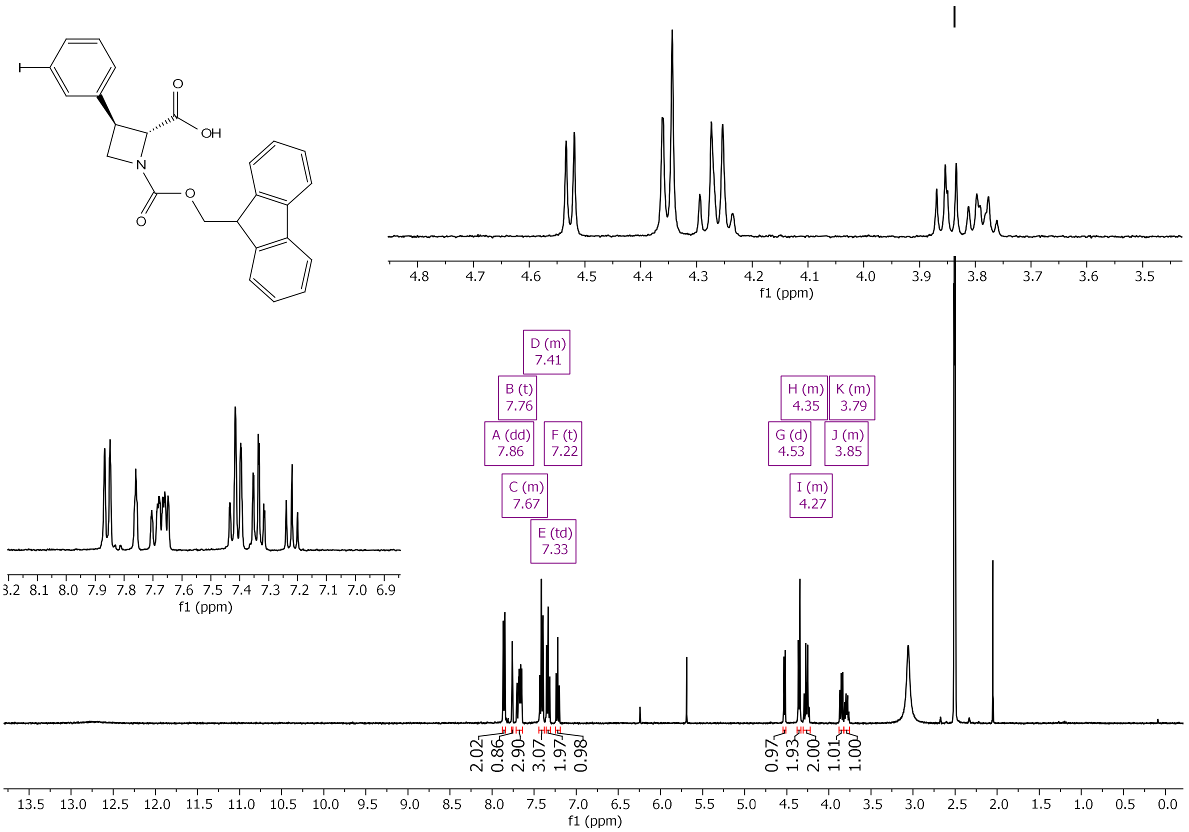

1, 2, 3 and 4 are spectra collected at 25, 40, 60 and 80°C, respectively:

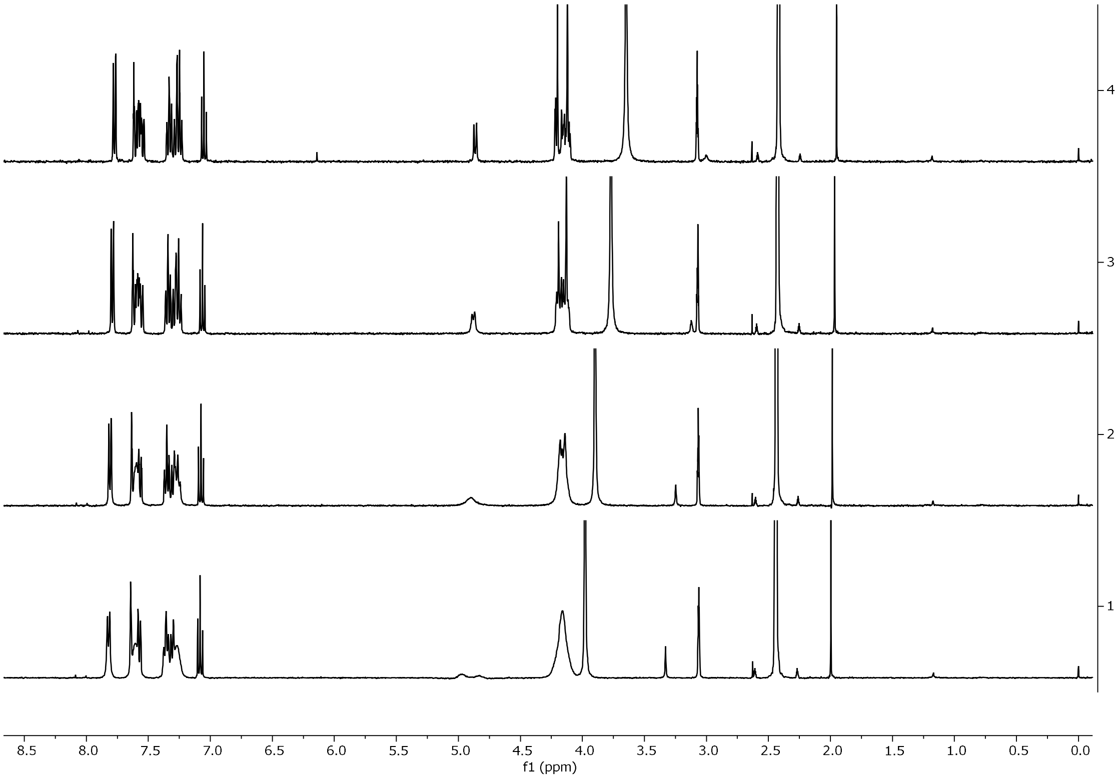

Overlay of HSQC and HMBC (rt in DMSO):

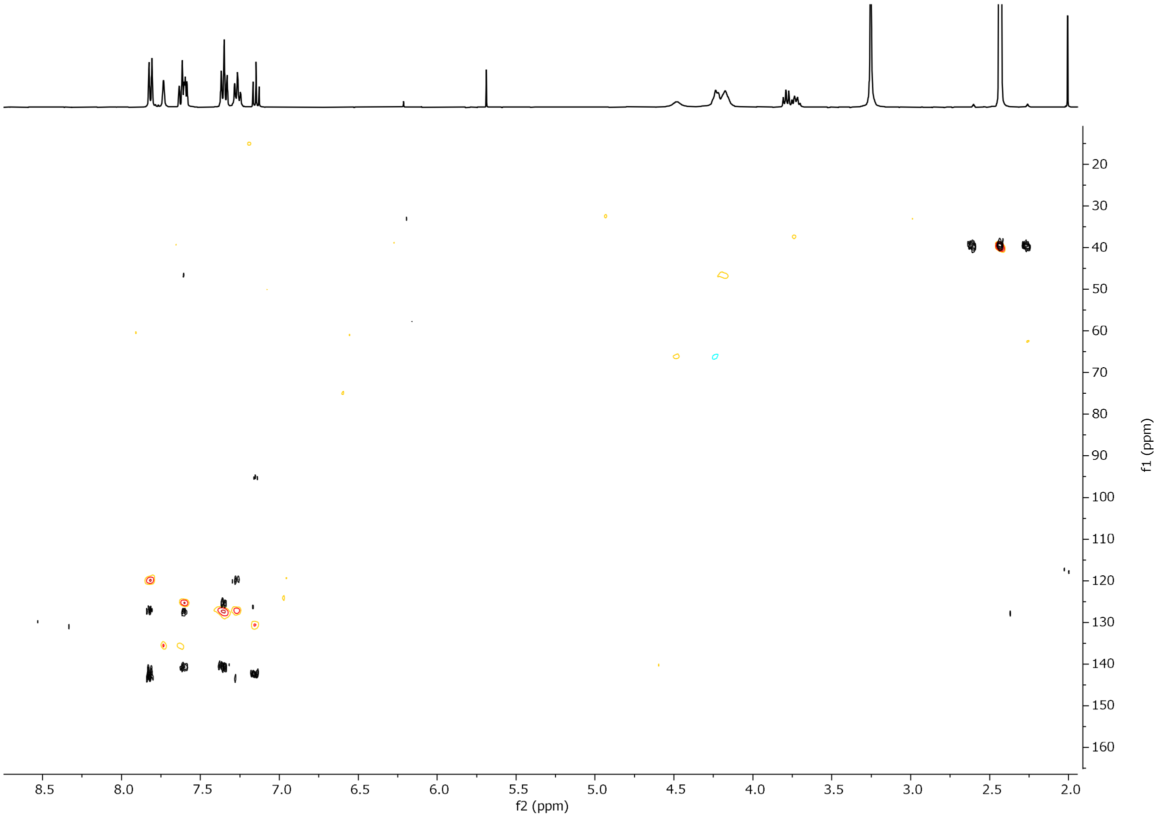

(2*S*,3*R*)-1-(((9H-fluoren-9-yl)methoxy)carbonyl)-3-(3-iodophenyl)pyrrolidine-2-carboxylic acid (**7c**)

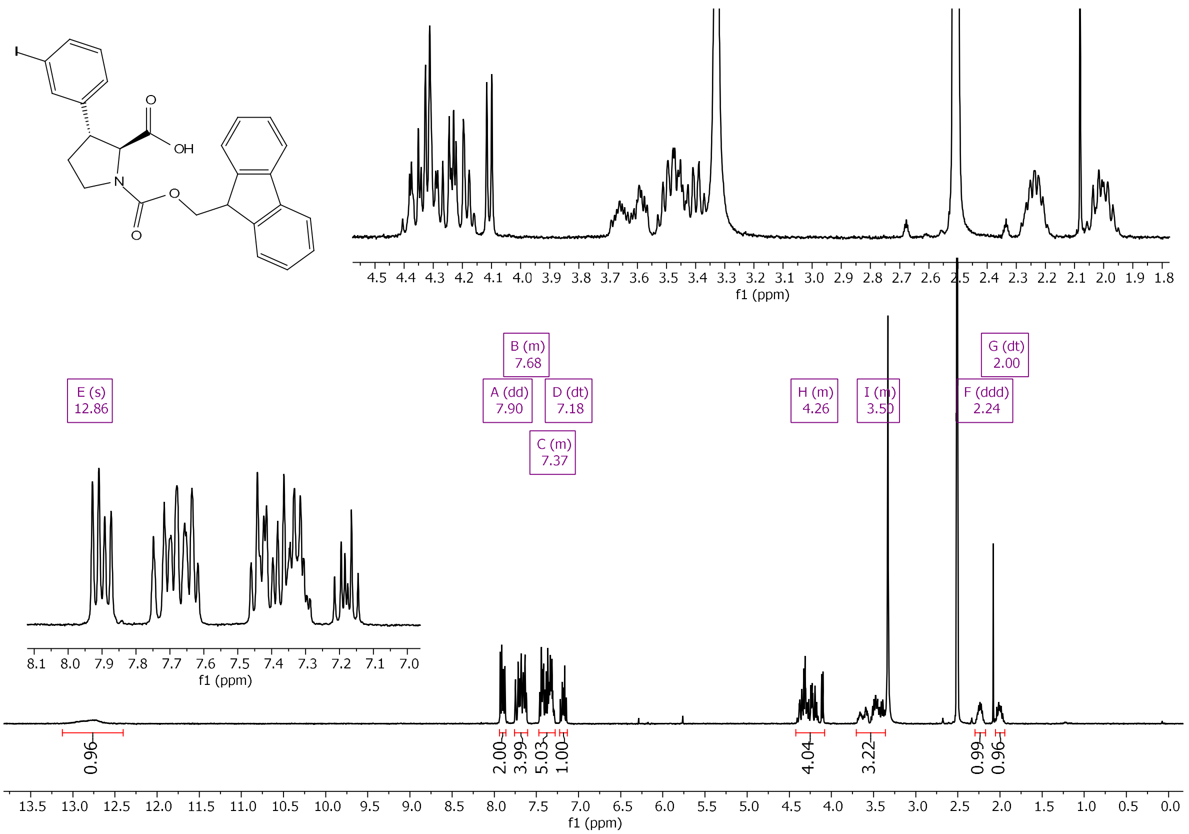

1, 2, 3 and 4 are spectra collected at 25, 40, 60 and 80°C, respectively:

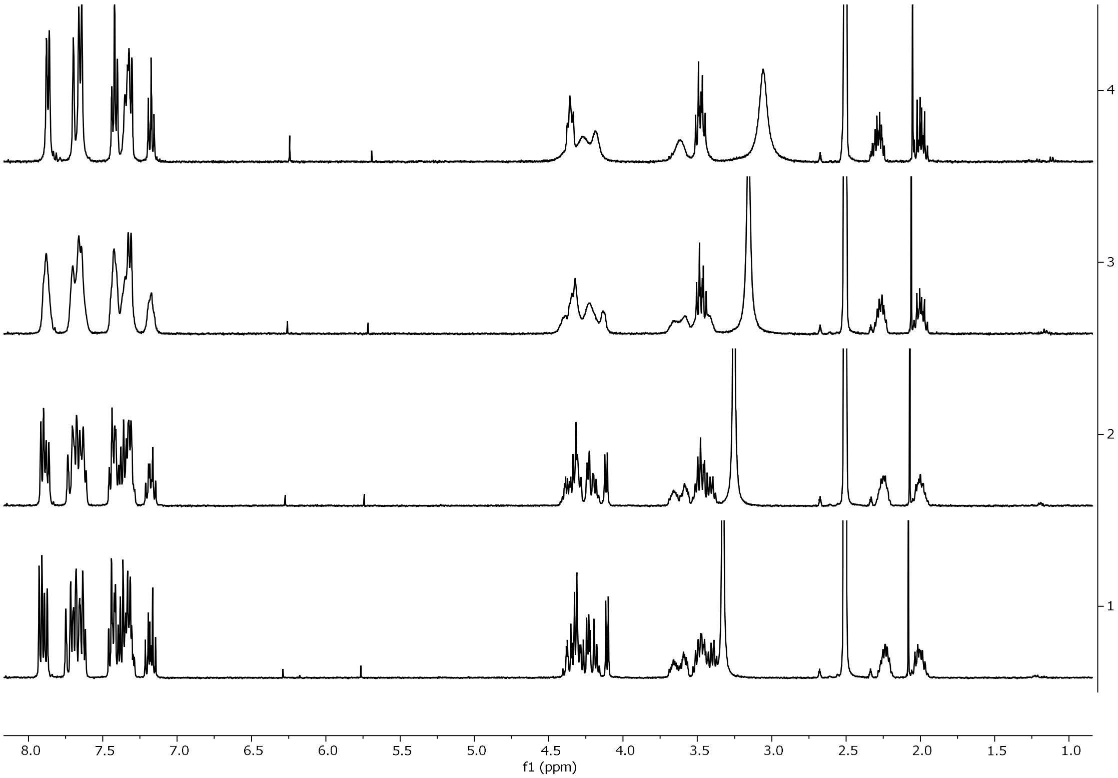

Overlay of HSQC and HMBC (rt in DMSO):

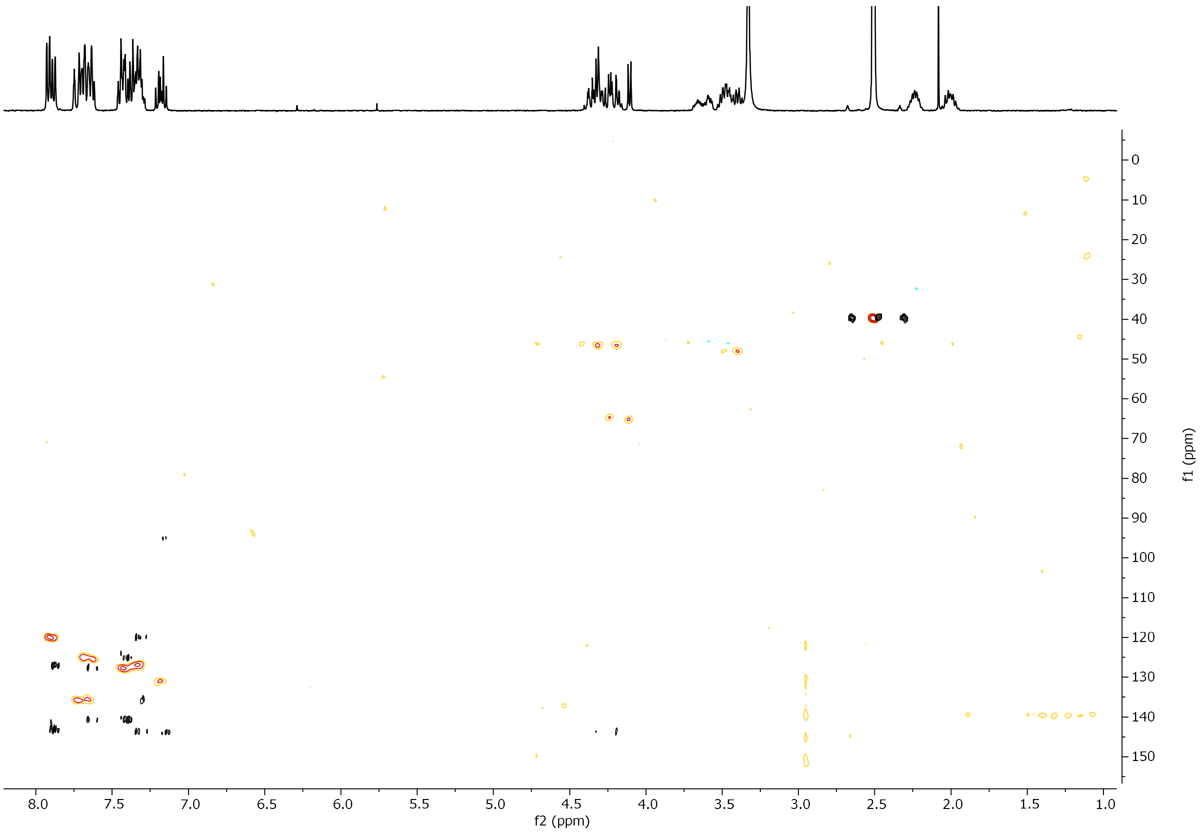

(2*R*,3*S*)-1-(((9H-fluoren-9-yl)methoxy)carbonyl)-3-(3-iodophenyl)pyrrolidine-2-carboxylic acid (**7d**)

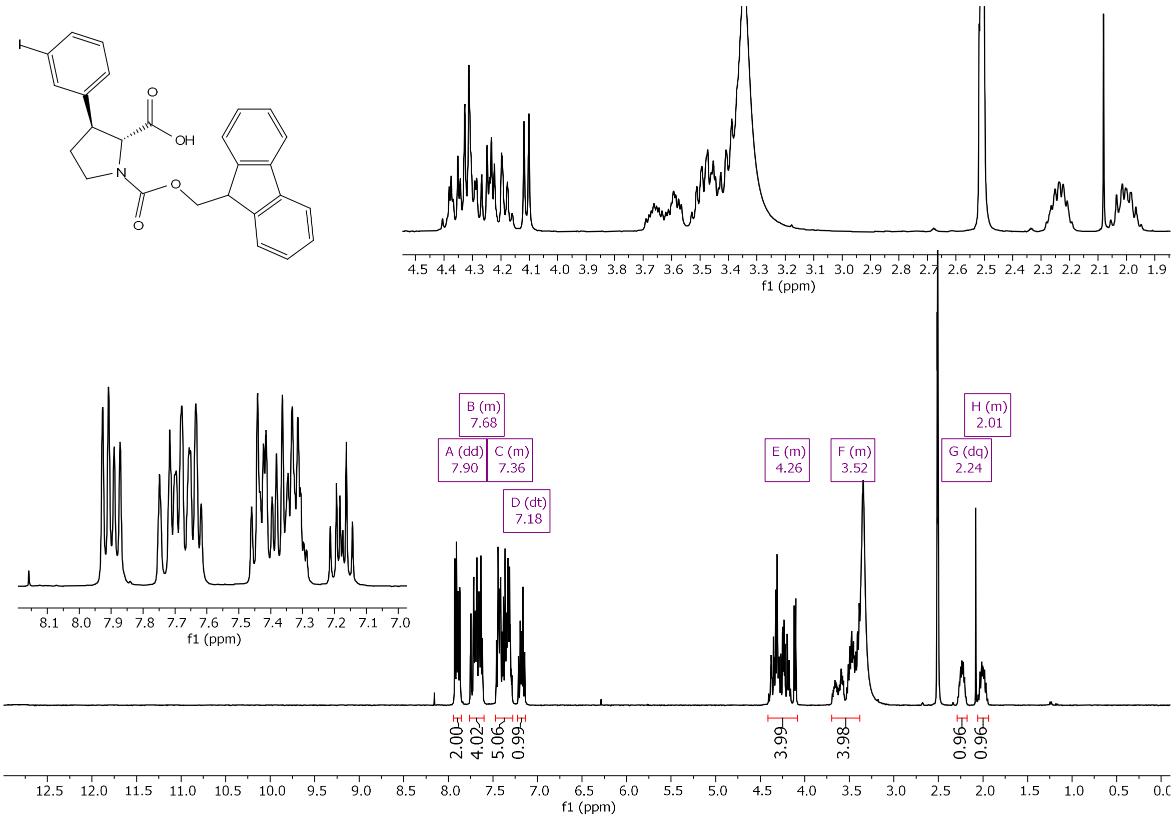

1, 2, 3 and 4 are spectra collected at 25, 40, 60 and 80°C, respectively:

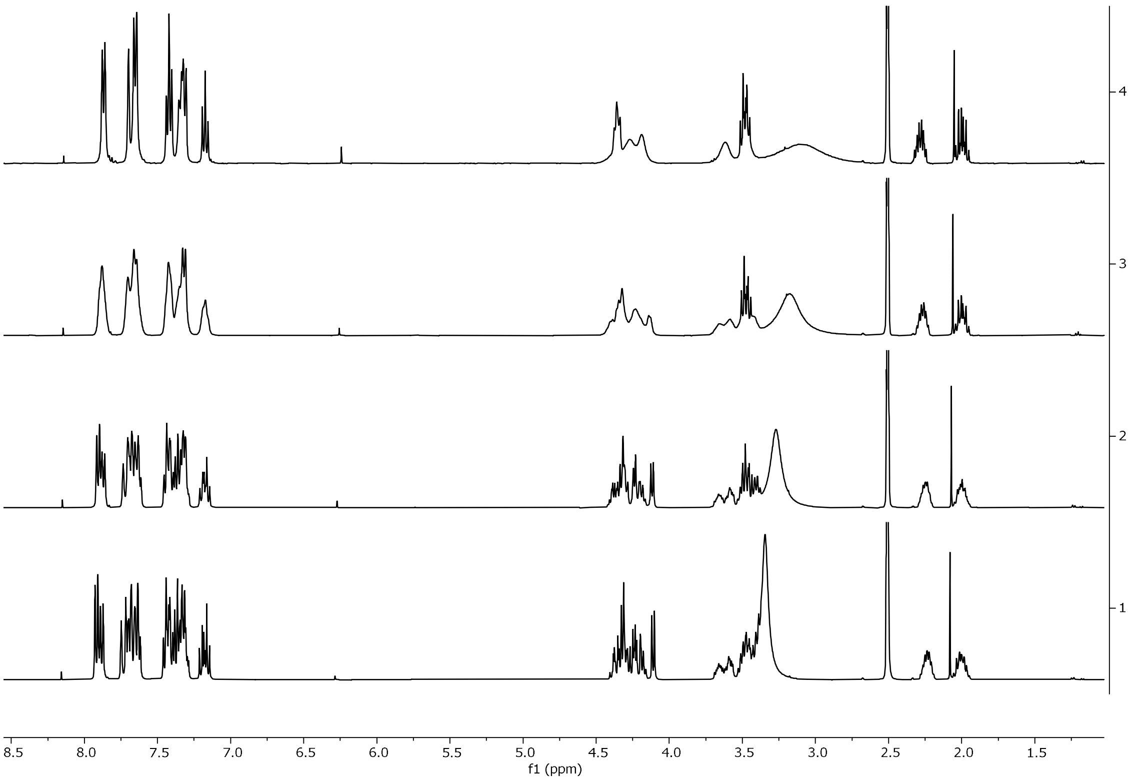

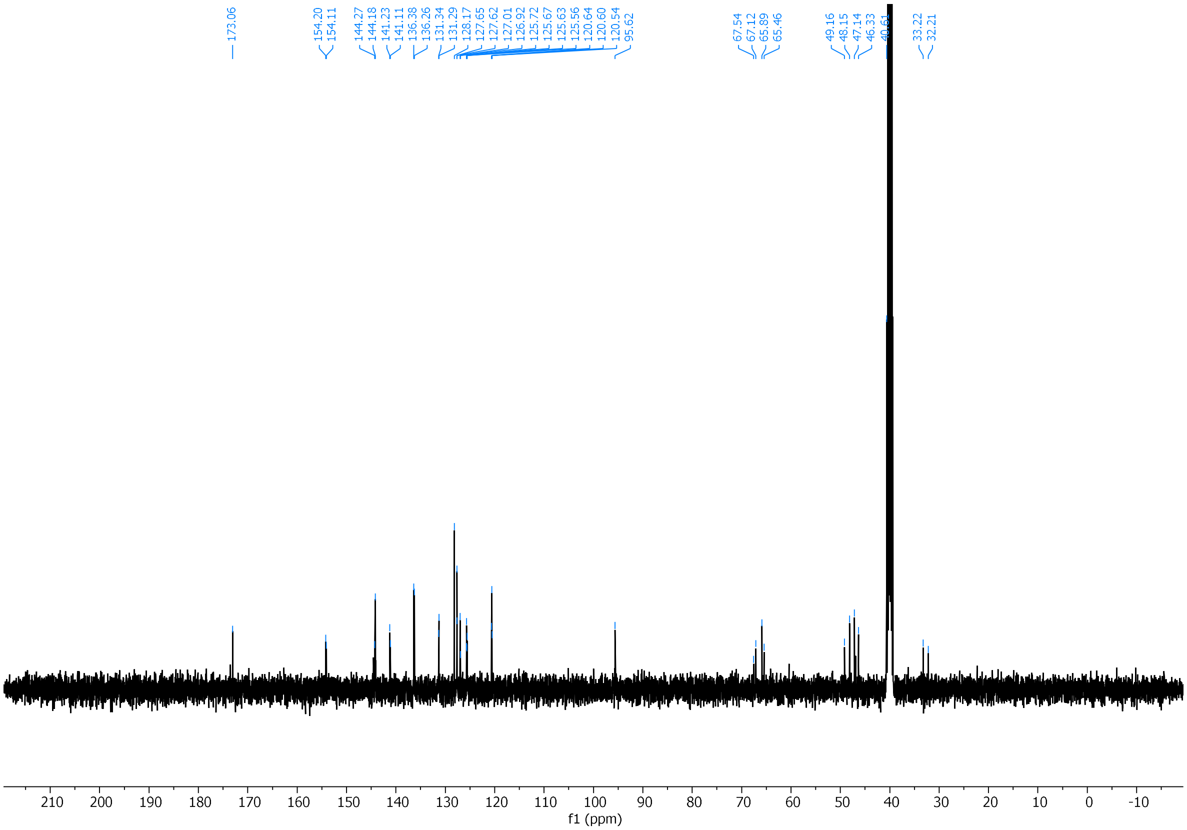

Overlay of HSQC and HMBC in DMSO:

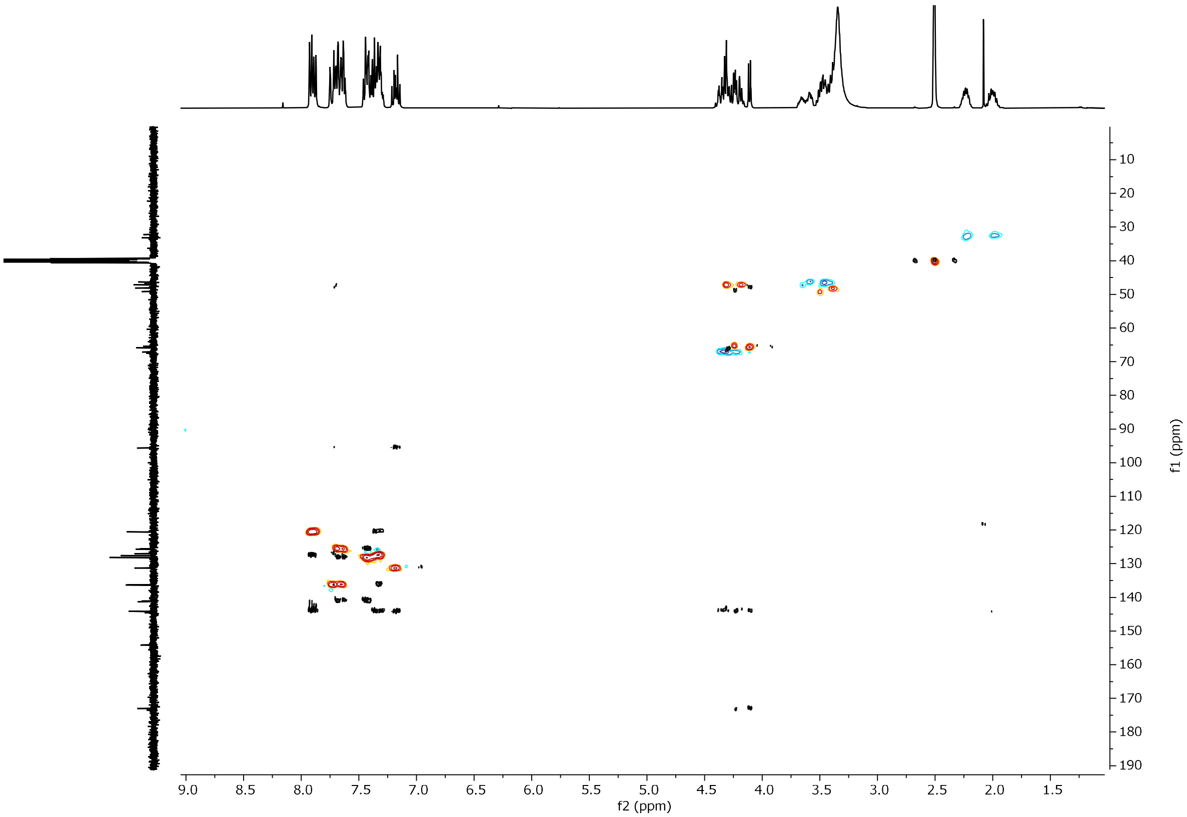

(2*S*,3*S*)-1-(((9H-fluoren-9-yl)methoxy)carbonyl)-3-(3-iodophenyl)piperidine-2-carboxylic acid (**7e**)

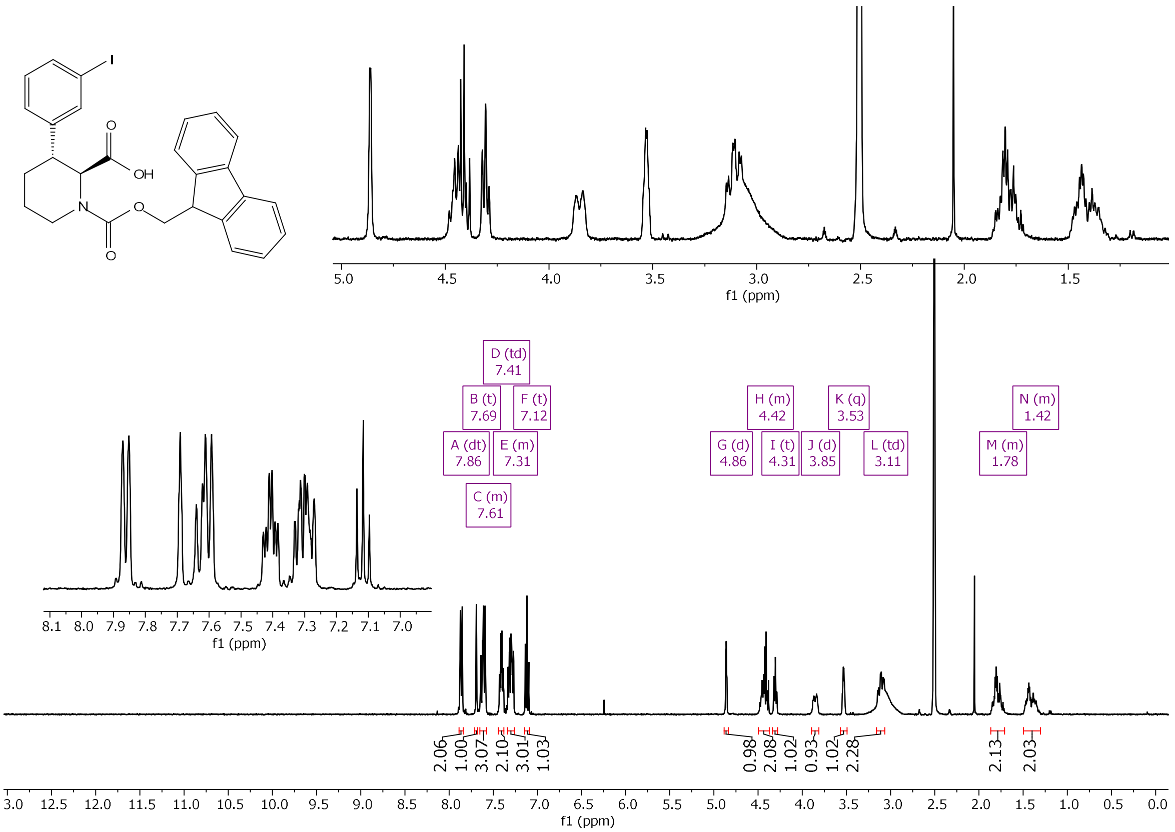

1, 2, 3 and 4 are spectra collected at 25, 40, 60 and 80°C, respectively:

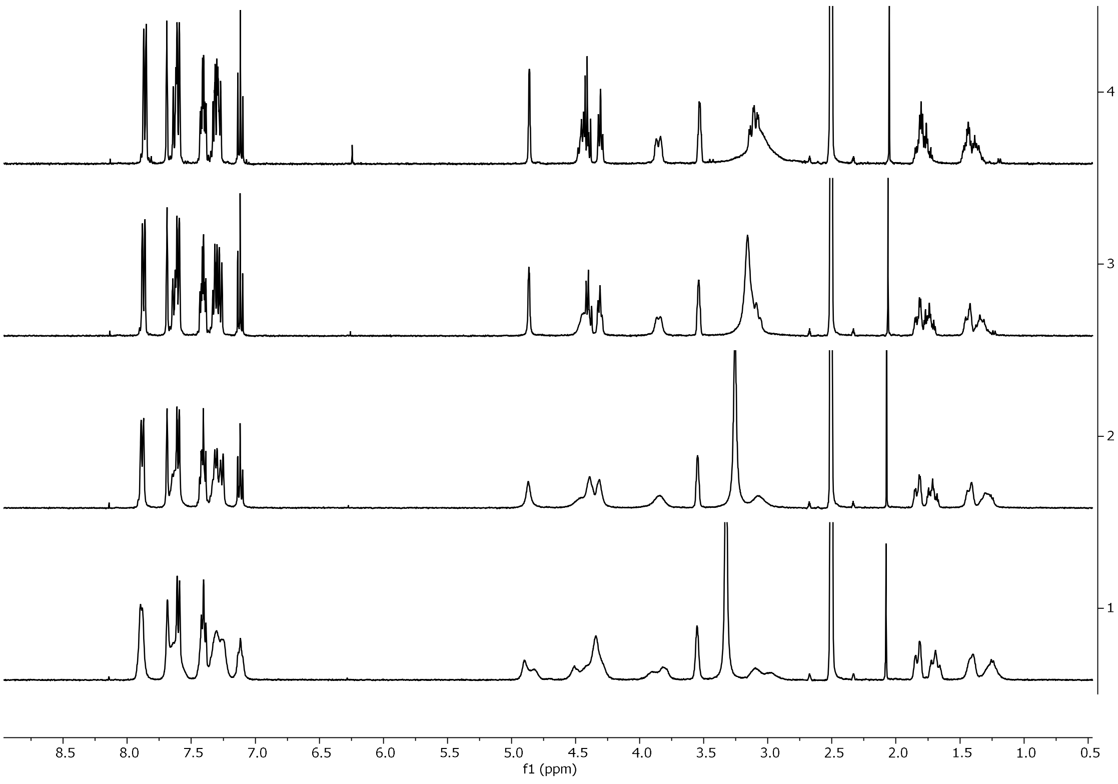

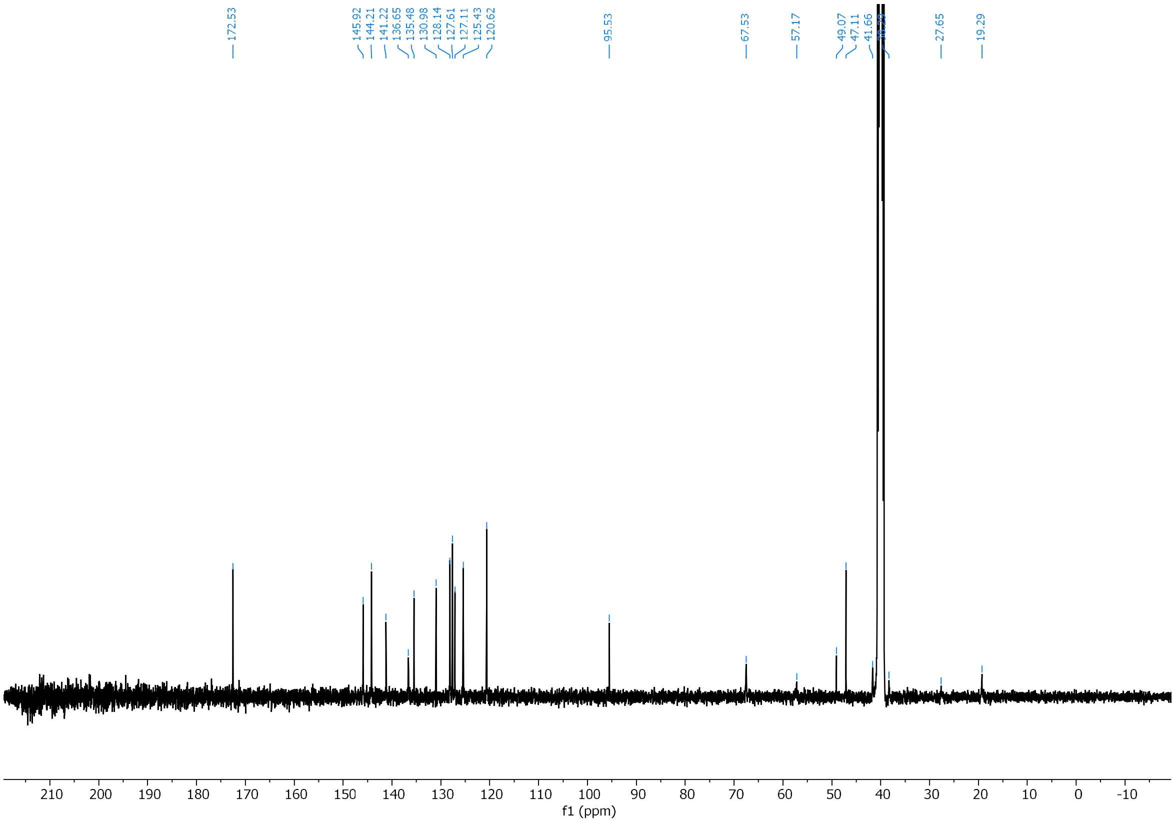

(2*R*,3*S*)-1-(((9H-fluoren-9-yl)methoxy)carbonyl)-3-(3-iodophenyl)piperidine-2-carboxylic acid (**7f**)

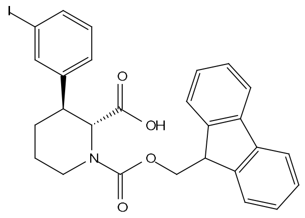

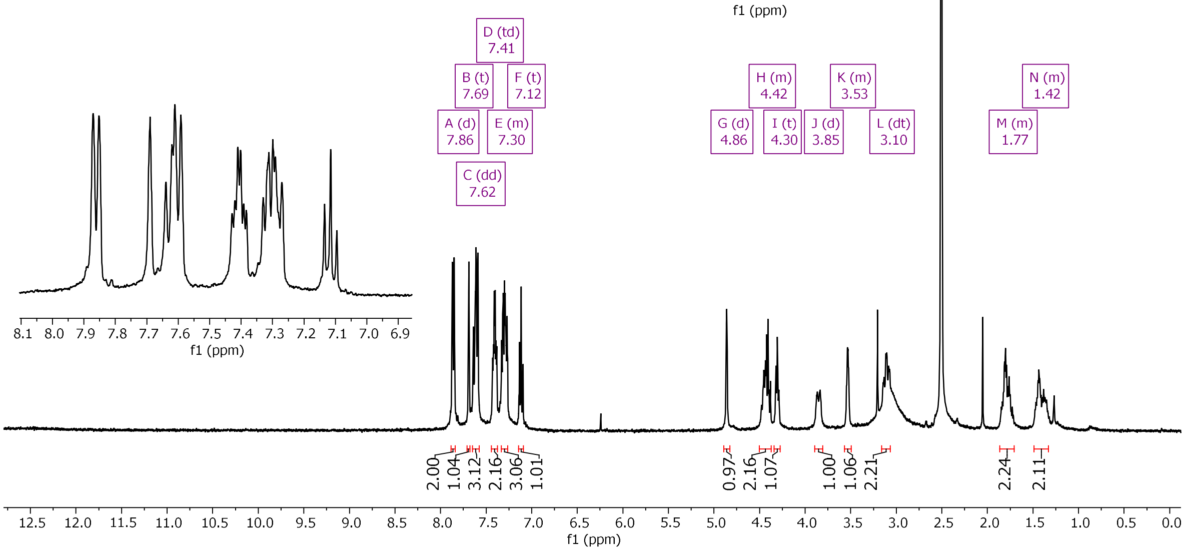

1, 2, 3 and 4 are spectra collected at 25, 40, 60 and 80°C, respectively:

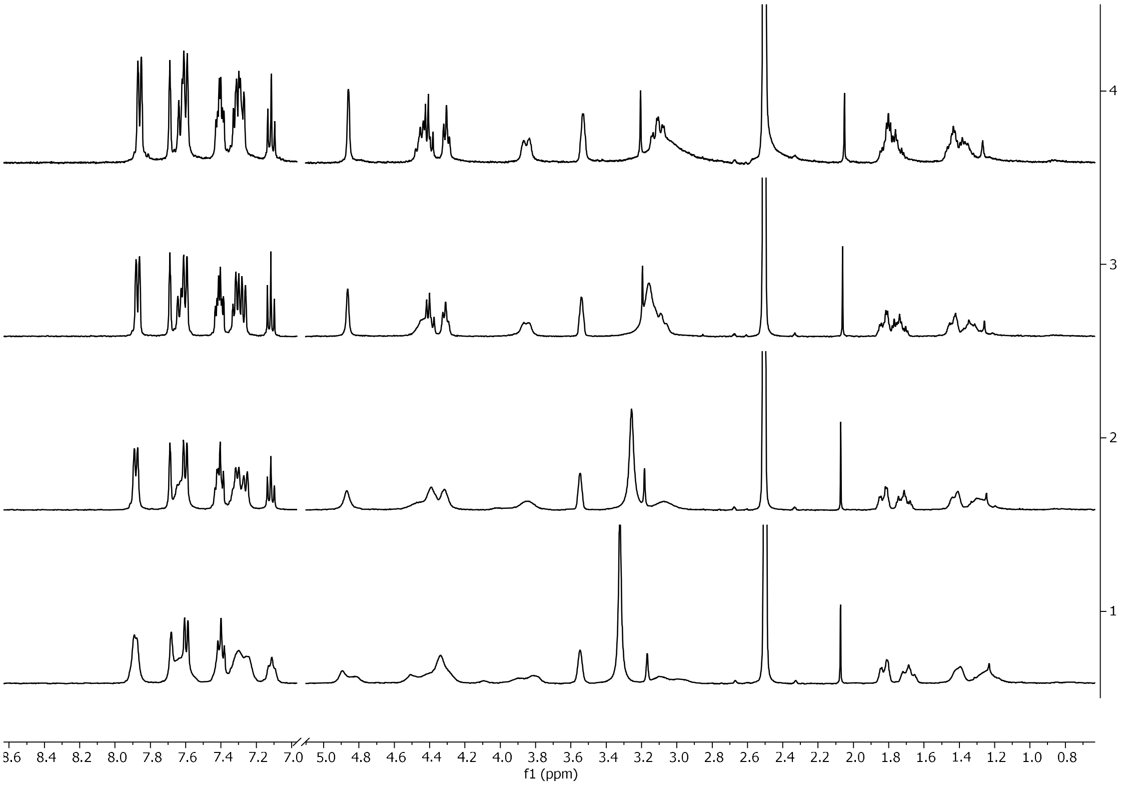

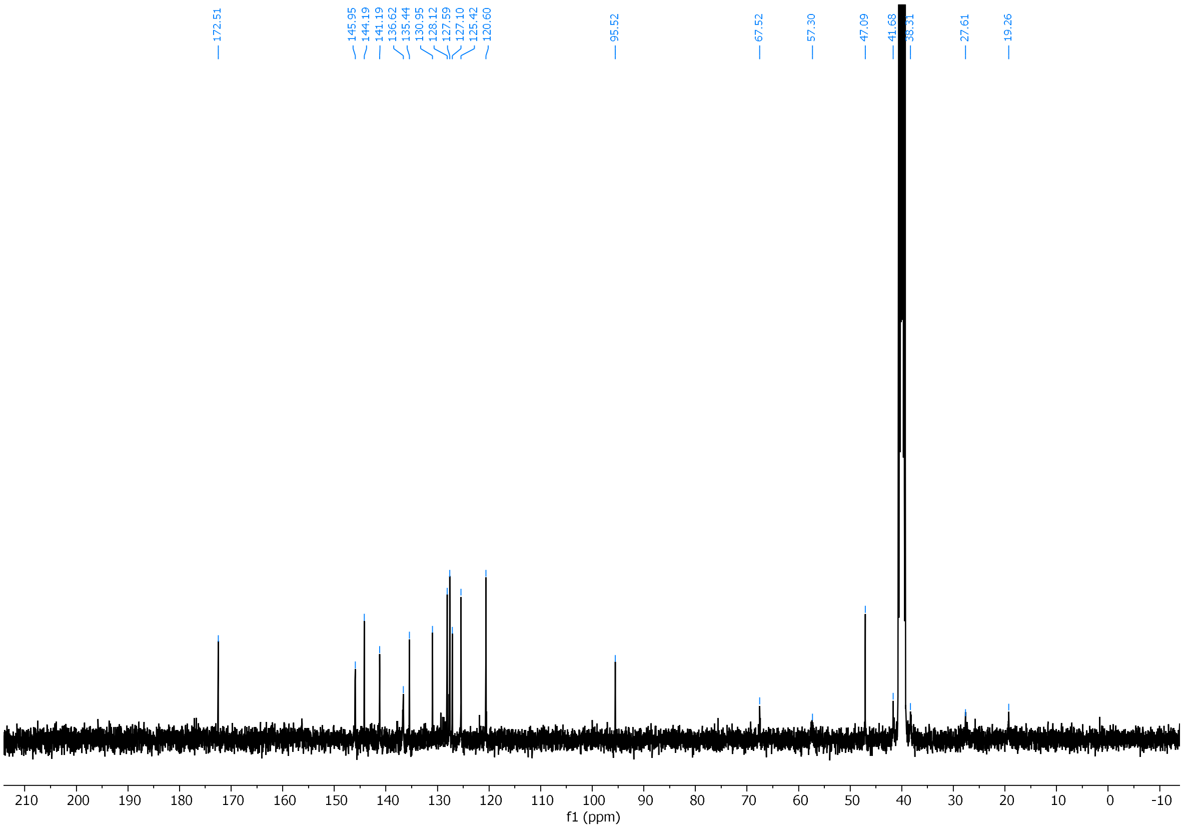

7.2. Hydroxyproline-derived skeletons

(2*S*,4*S*)-1-tert-butyl 2-methyl 4-(4-iodophenoxy)pyrrolidine-1,2-dicarboxylate (**9ai**)

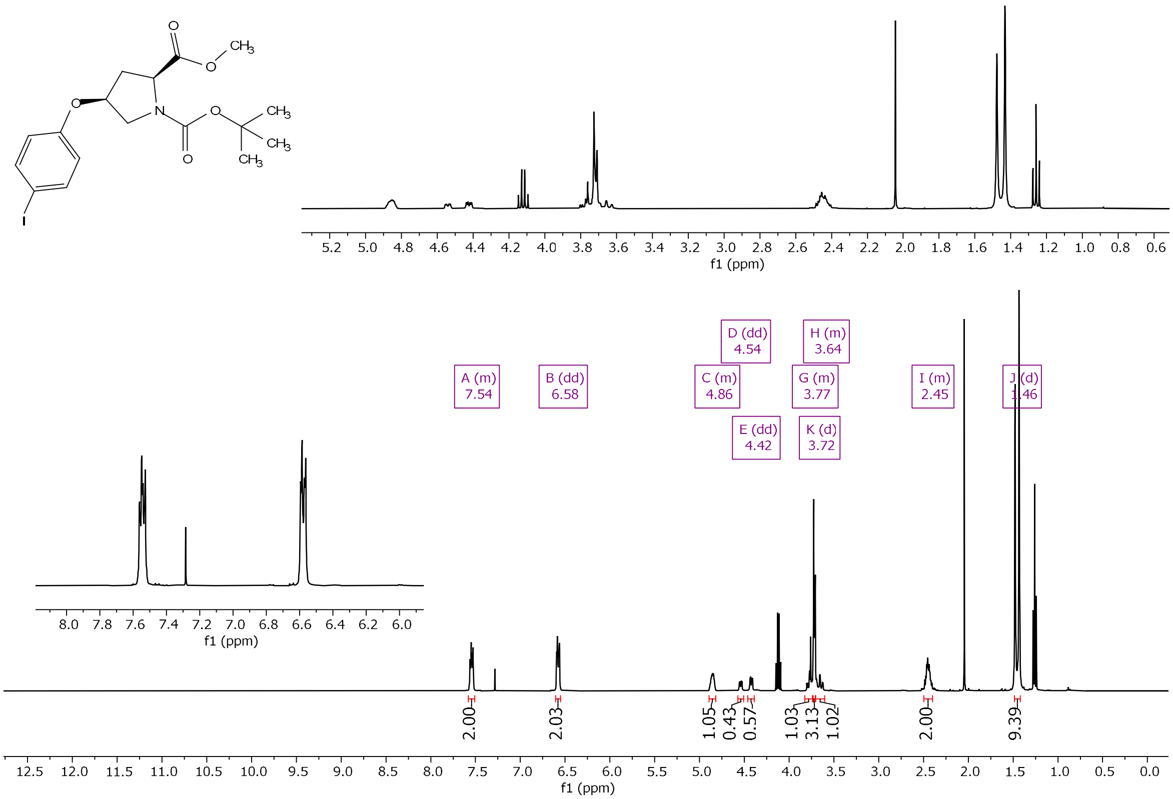

(2*S*,4*R*)-1-tert-butyl 2-methyl 4-(4-iodophenoxy)pyrrolidine-1,2-dicarboxylate (**9bi**)

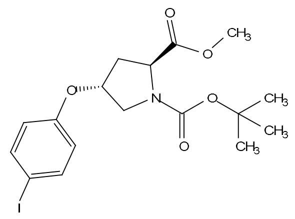

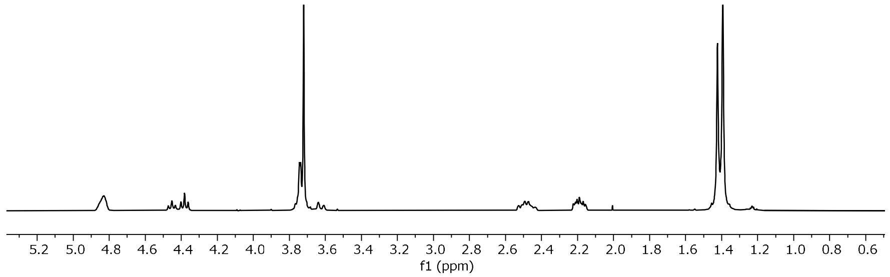

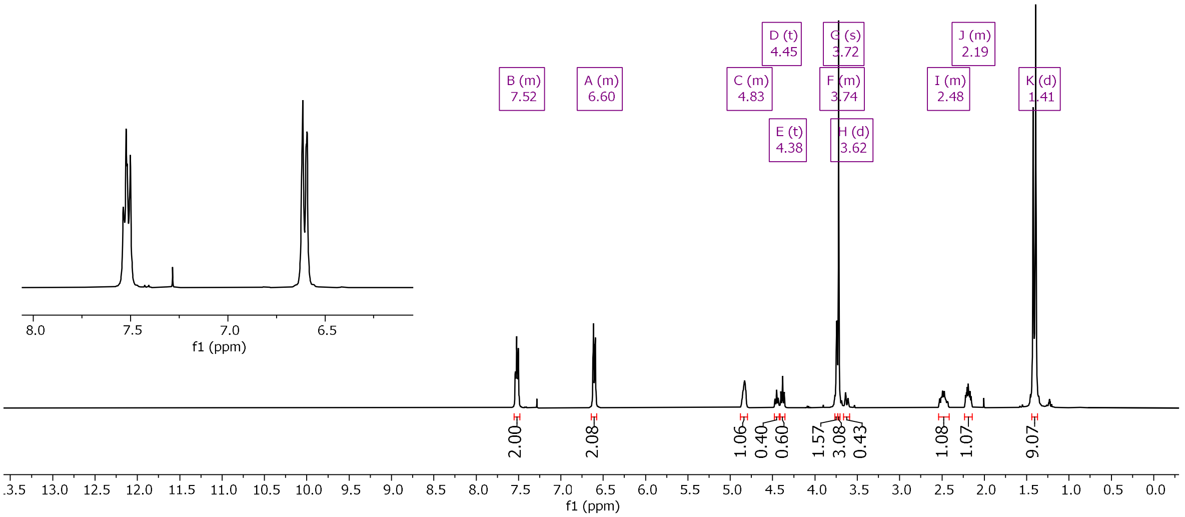

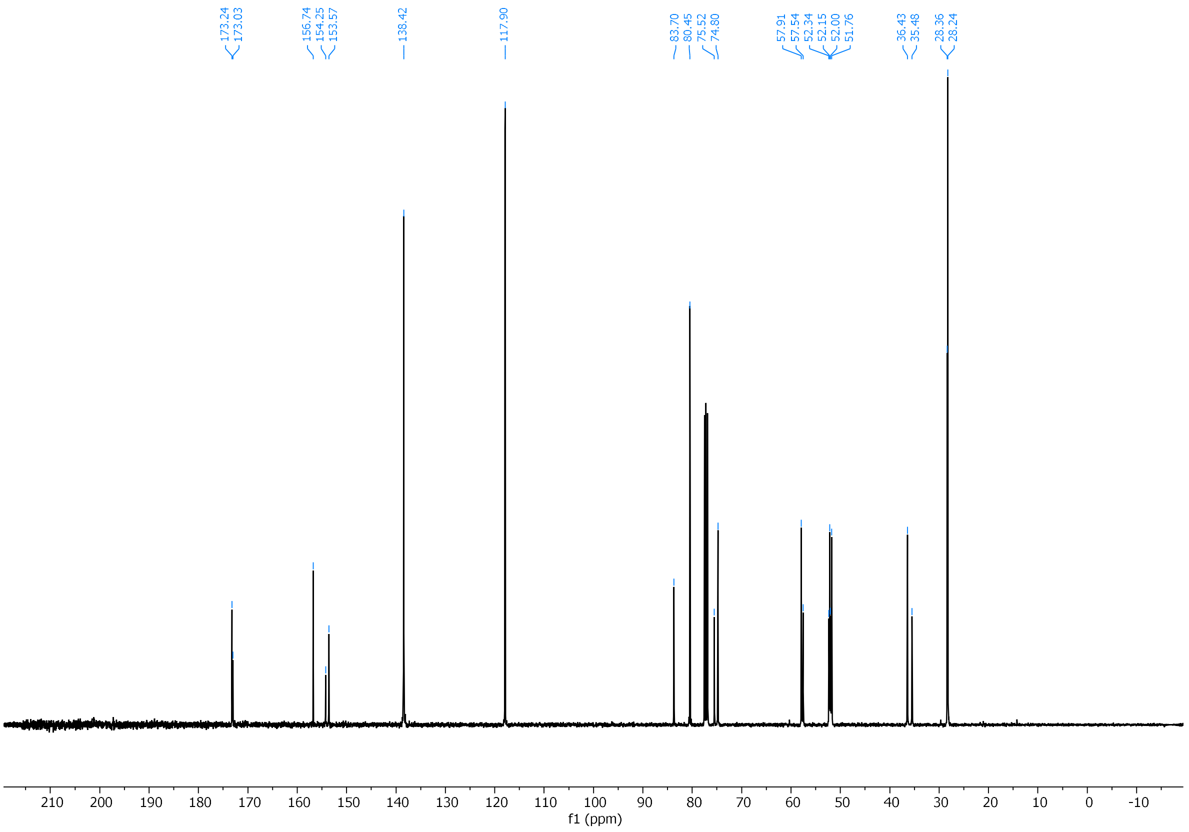

(2*R*,4*R*)-1-tert-butyl 2-methyl 4-(4-iodophenoxy)pyrrolidine-1,2-dicarboxylate (**9ci**)

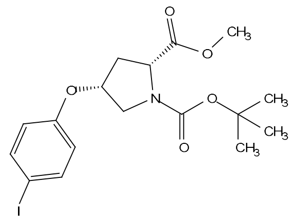

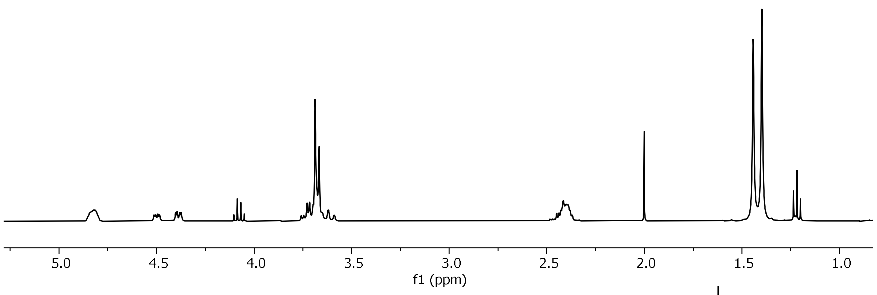

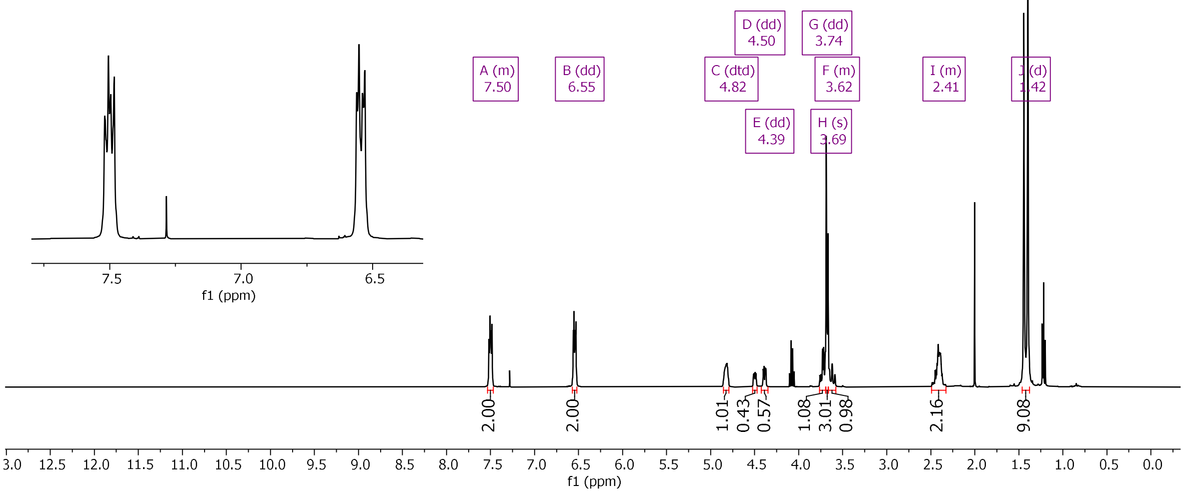

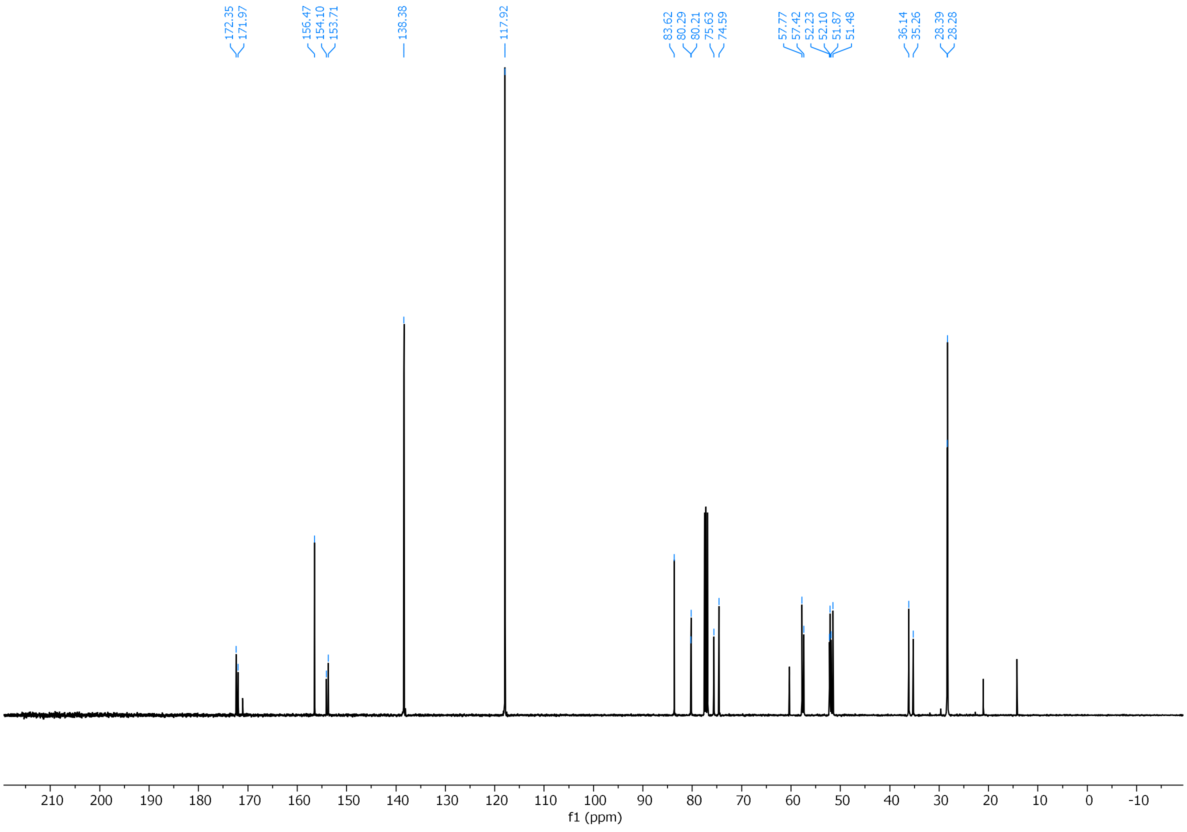

(2*R*,4*S*)-1-tert-butyl 2-methyl 4-(4-iodophenoxy)pyrrolidine-1,2-dicarboxylate (**9di**)

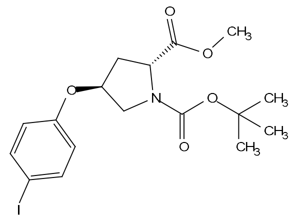

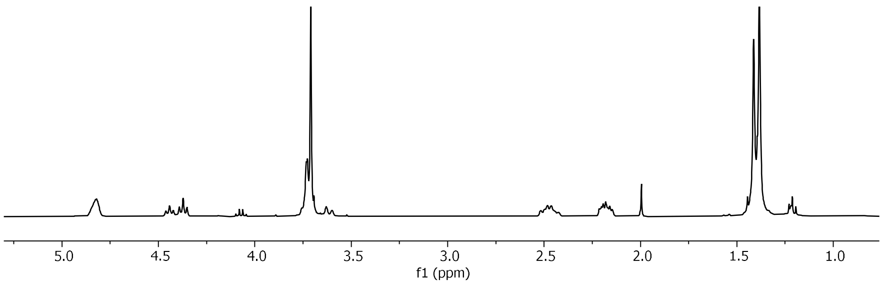

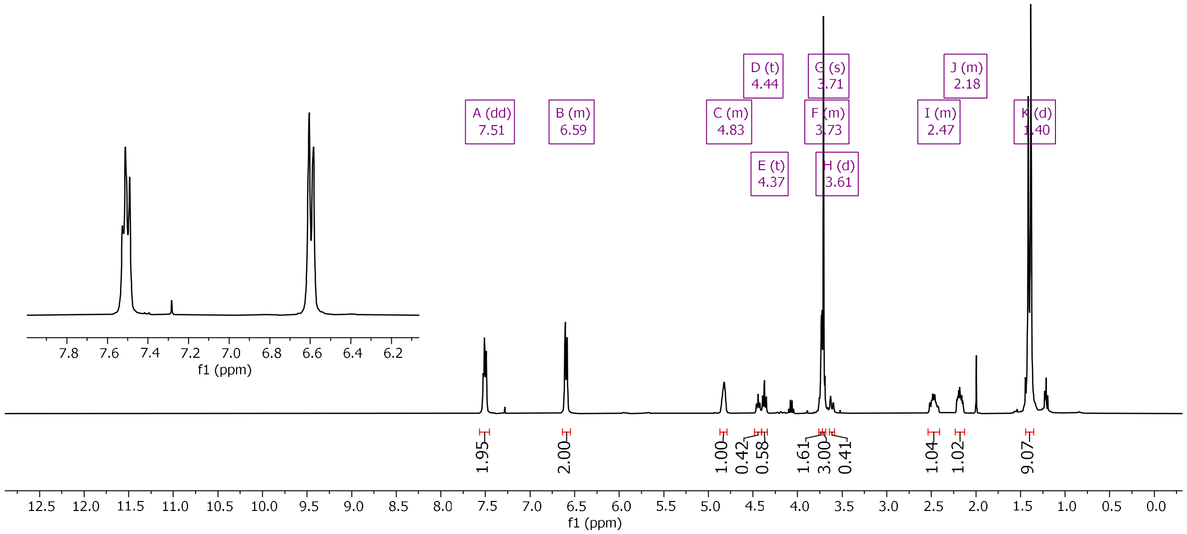

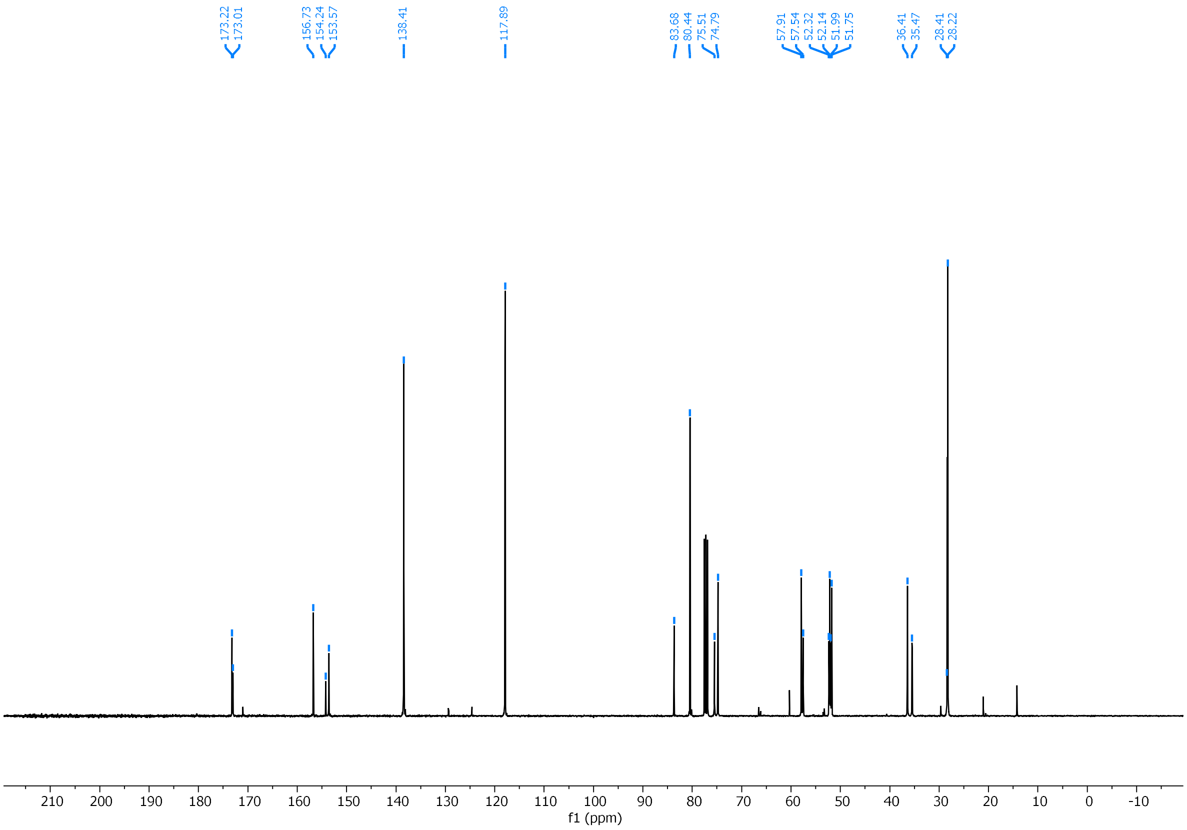

(2*S*,4*S*)-1-tert-butyl 2-methyl 4-(3-iodophenoxy)pyrrolidine-1,2-dicarboxylate (**9aii**)

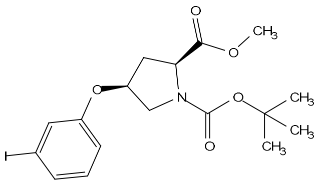

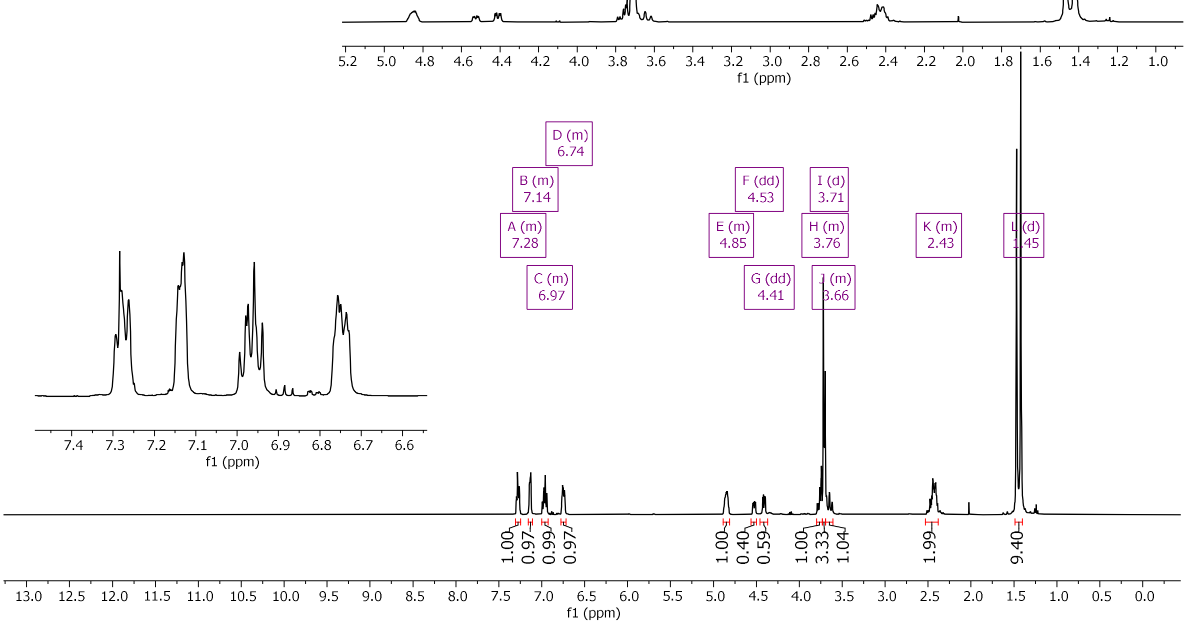

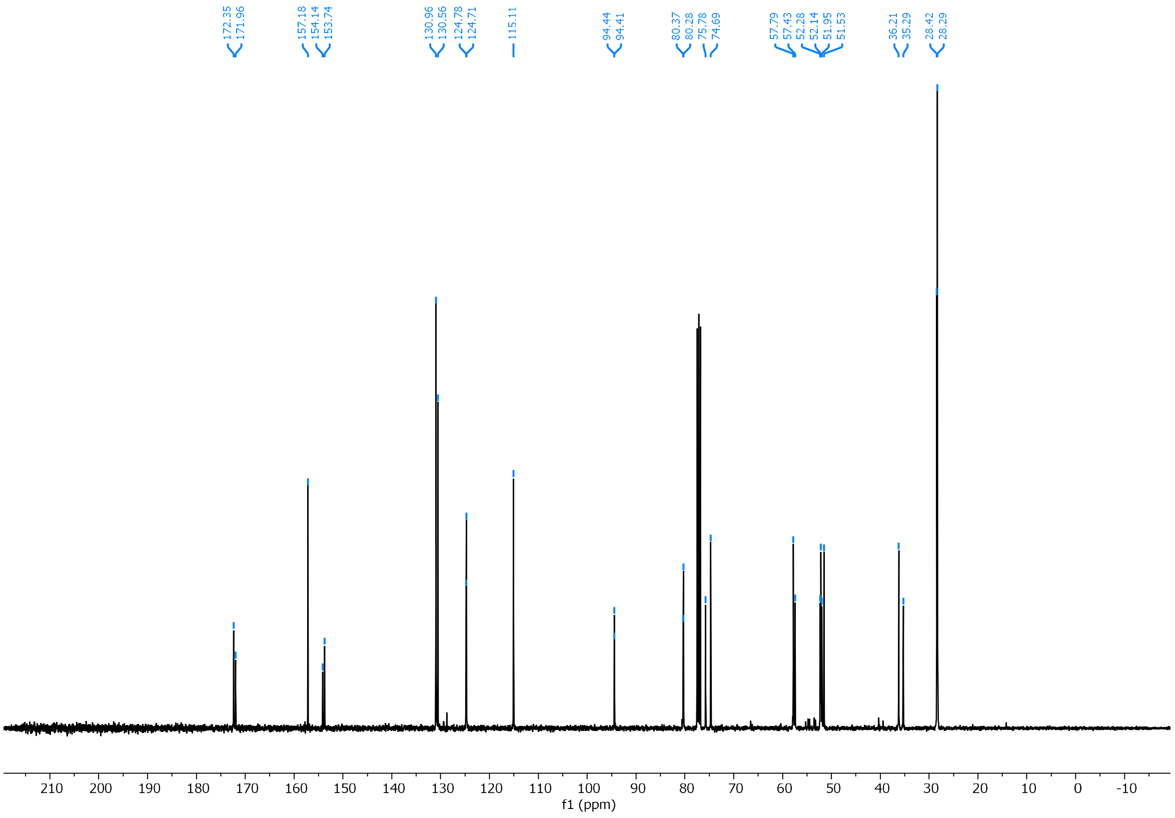

(2*S*,4*R*)-1-tert-butyl 2-methyl 4-(3-iodophenoxy)pyrrolidine-1,2-dicarboxylate (**9bii**)

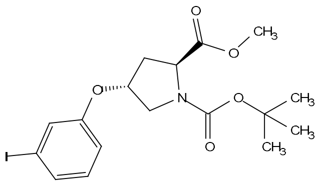

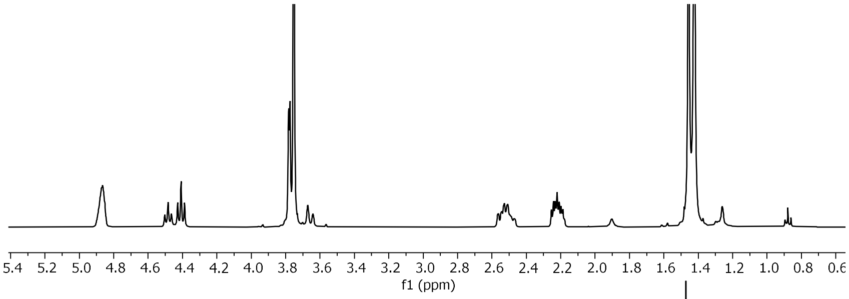

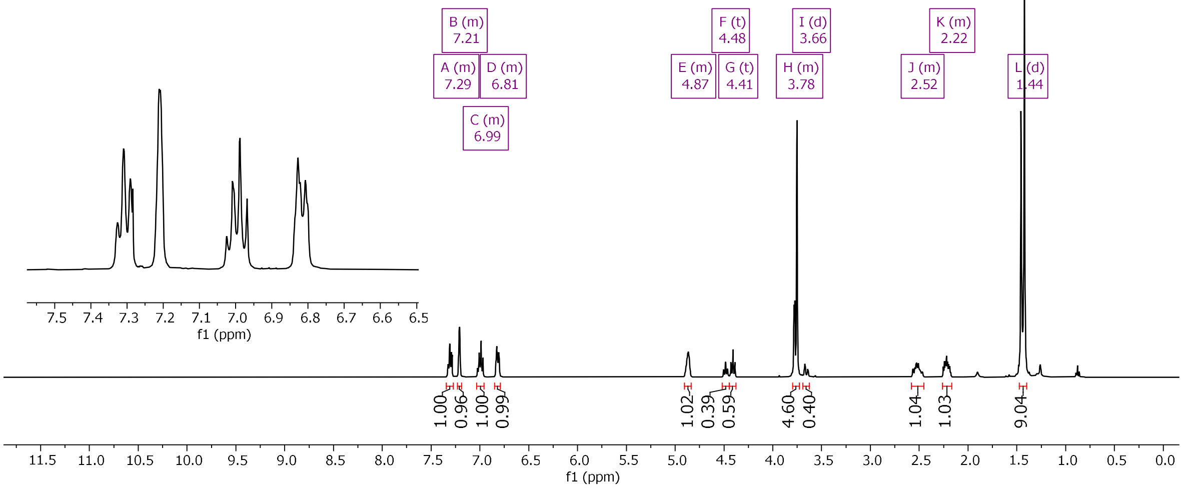

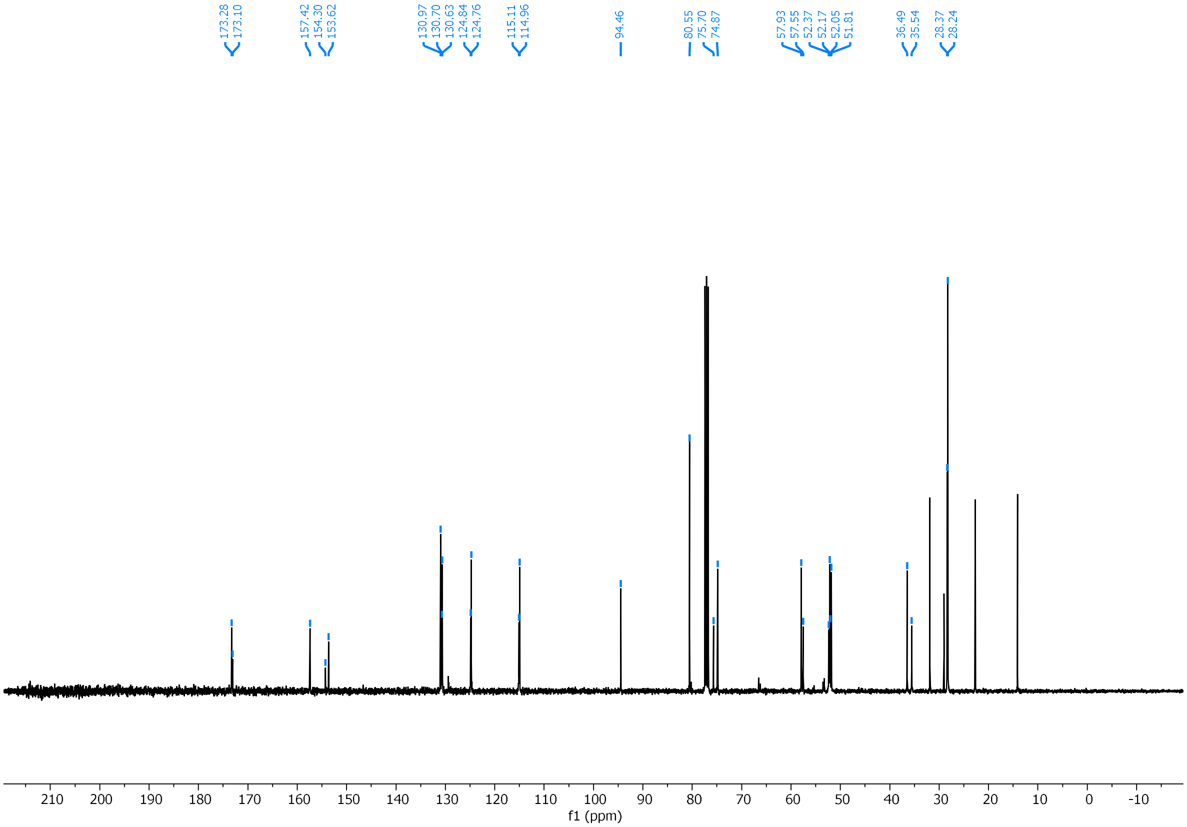

(2*R*,4*R*)-1-tert-butyl 2-methyl 4-(3-iodophenoxy)pyrrolidine-1,2-dicarboxylate (**9cii**)

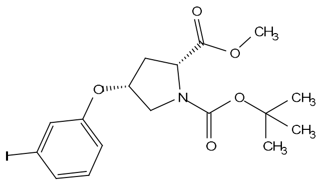

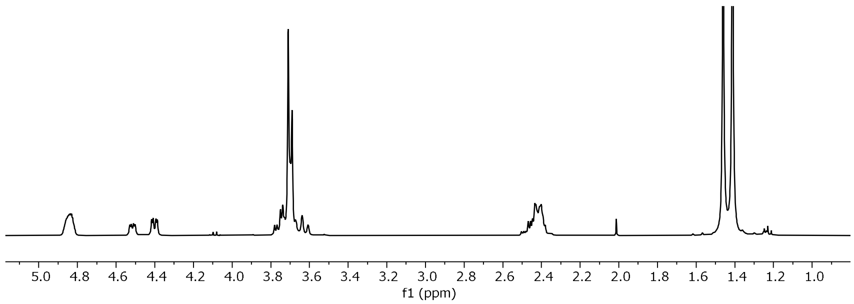

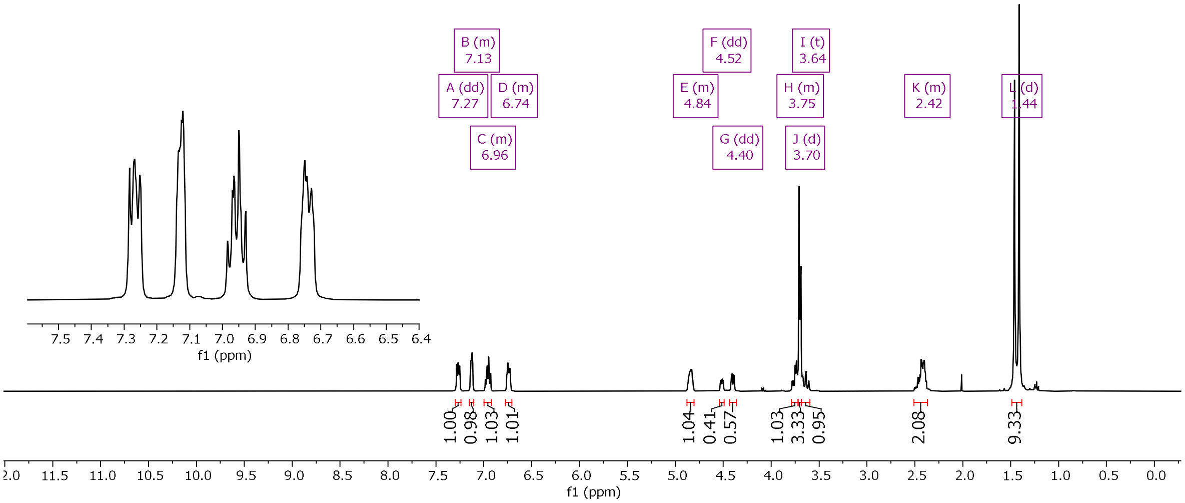

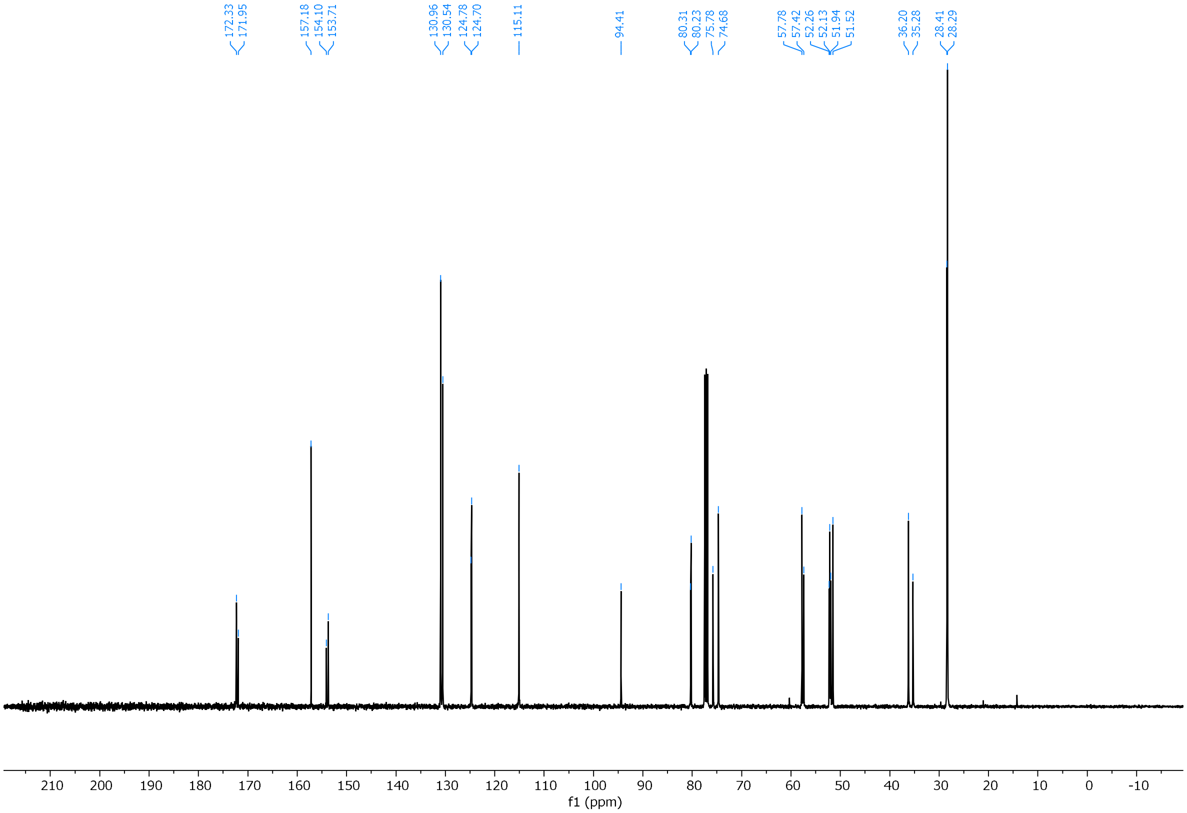

(2*R*,4*S*)-1-tert-butyl 2-methyl 4-(3-iodophenoxy)pyrrolidine-1,2-dicarboxylate (**9dii**)

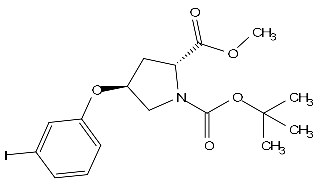

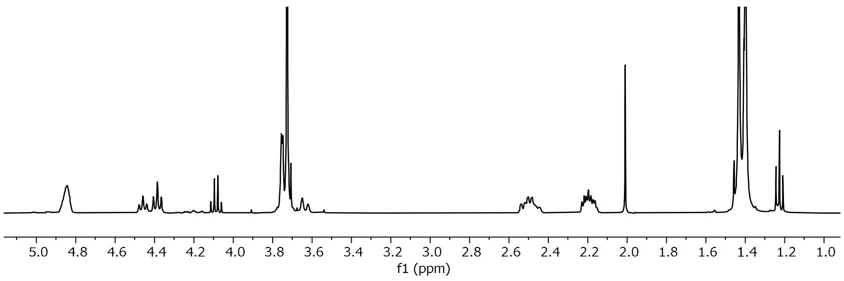

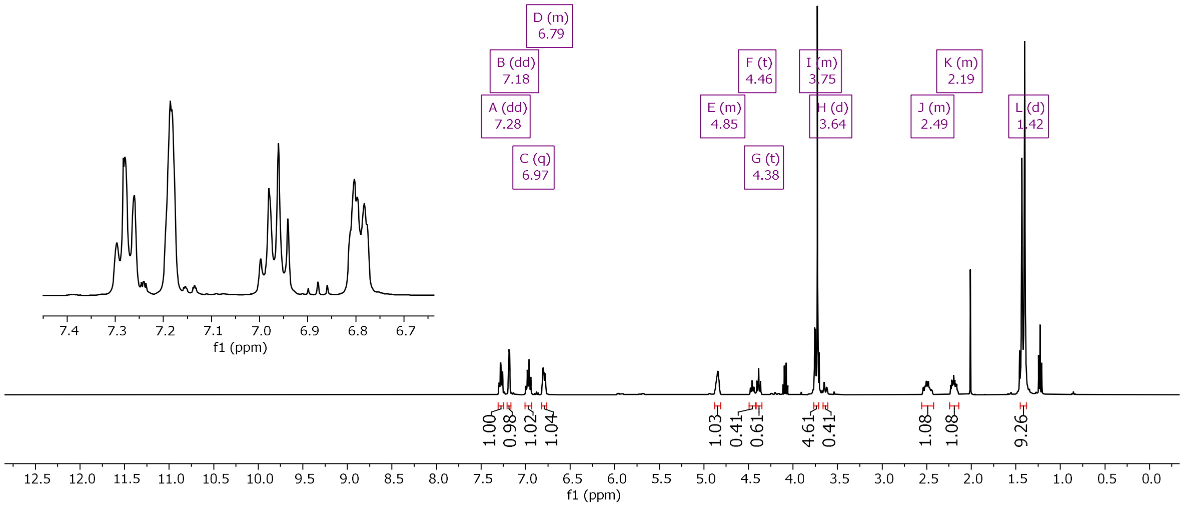

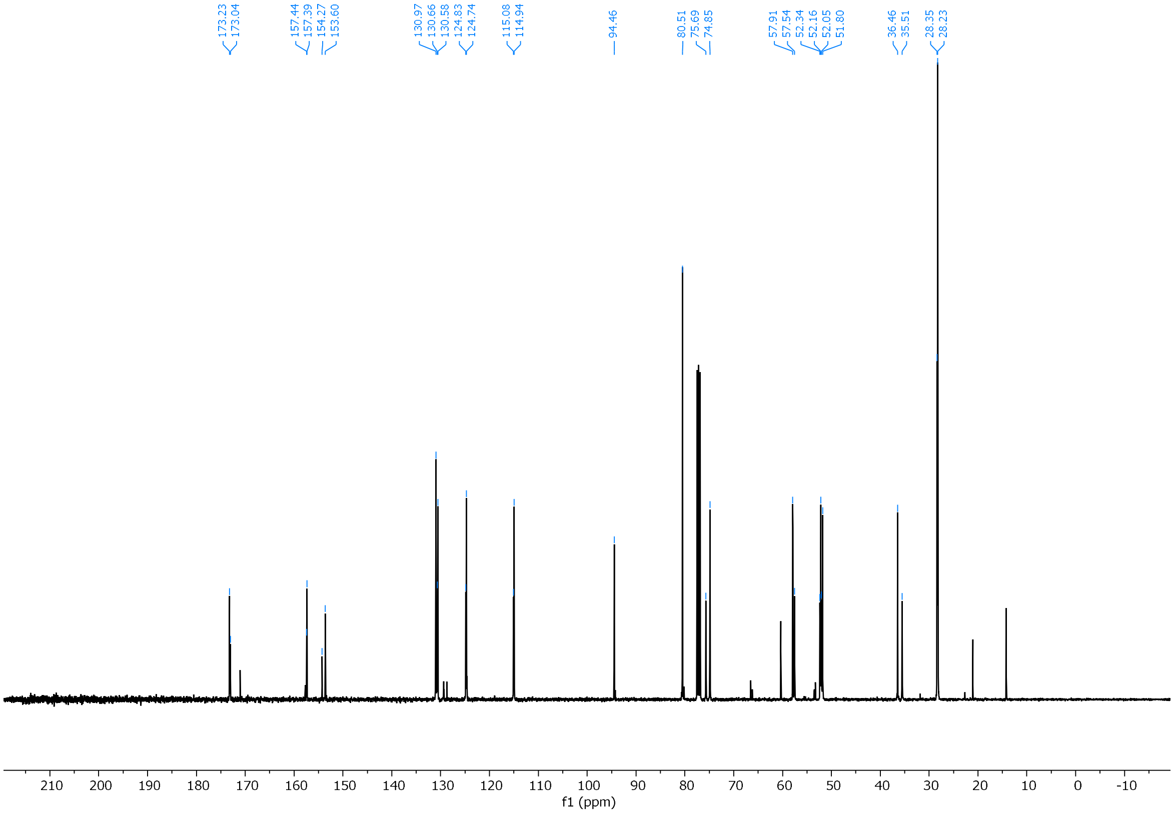

(2*S*,4*S*)-1-(tert-butoxycarbonyl)-4-((4-iodobenzyl)oxy)pyrrolidine-2-carboxylic acid (**13ai**)

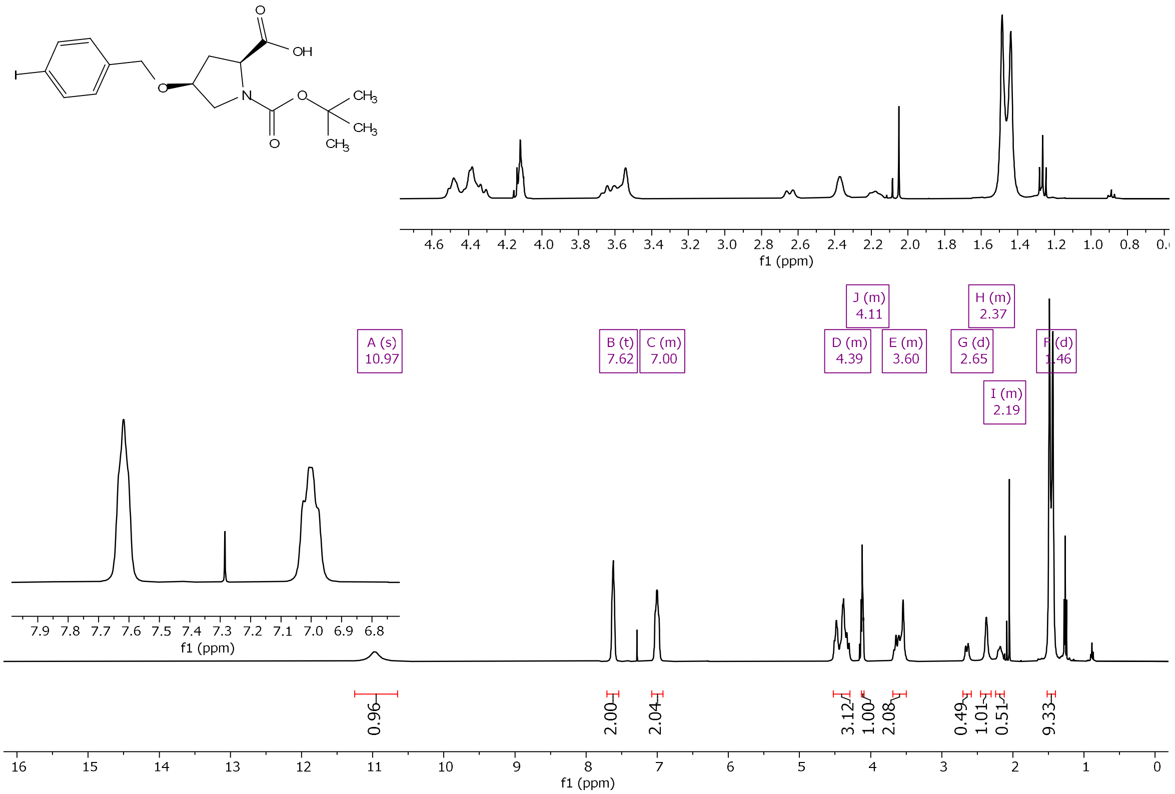

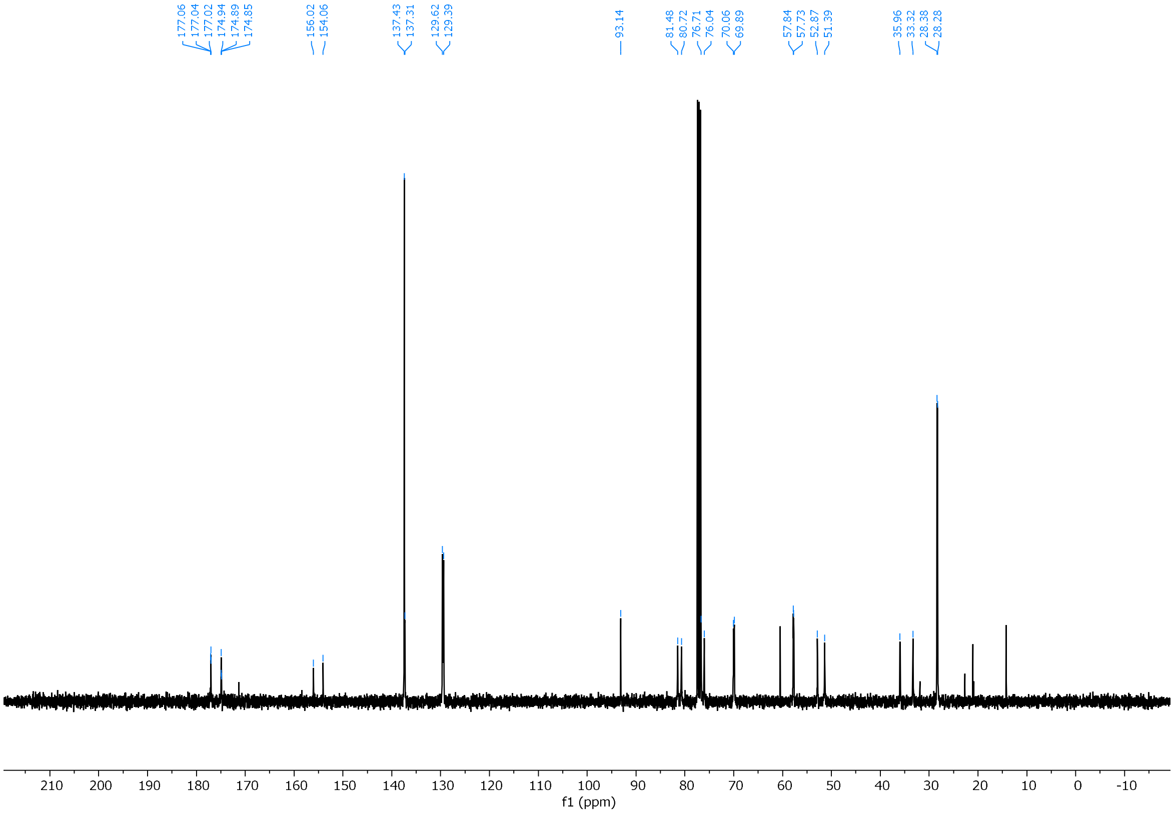

(2*S*,4*R*)-1-(tert-butoxycarbonyl)-4-((4-iodobenzyl)oxy)pyrrolidine-2-carboxylic acid (**13bi**)

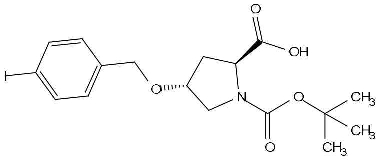

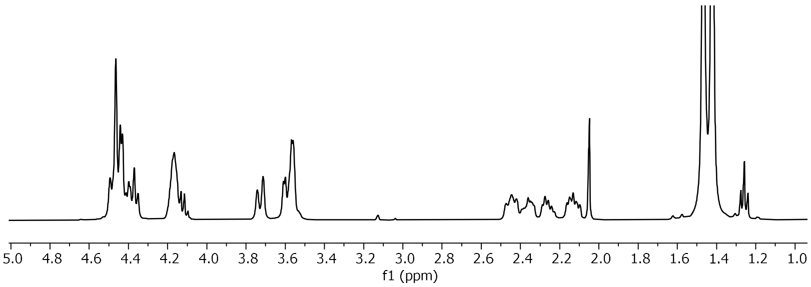

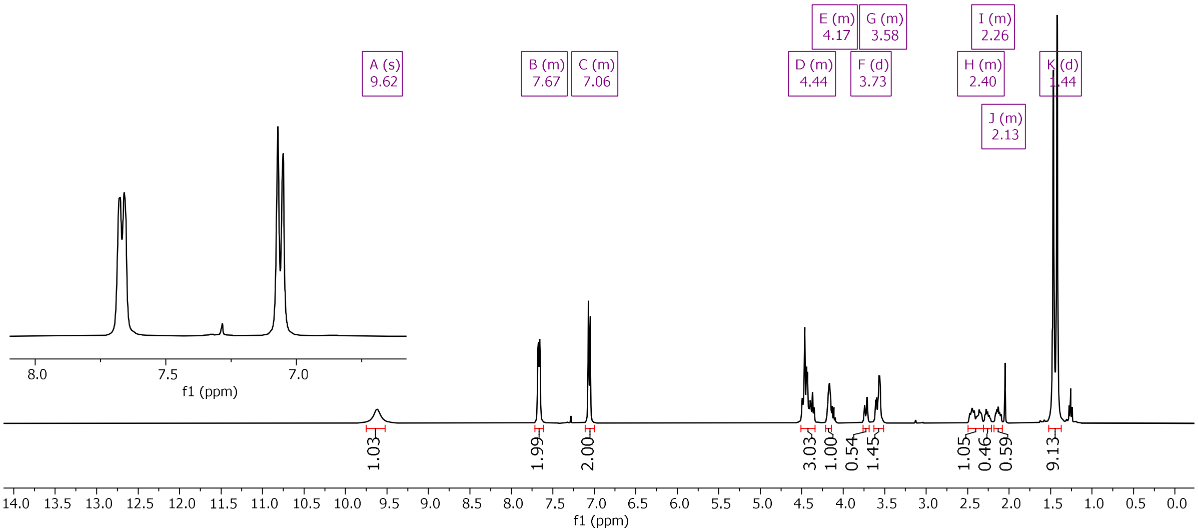

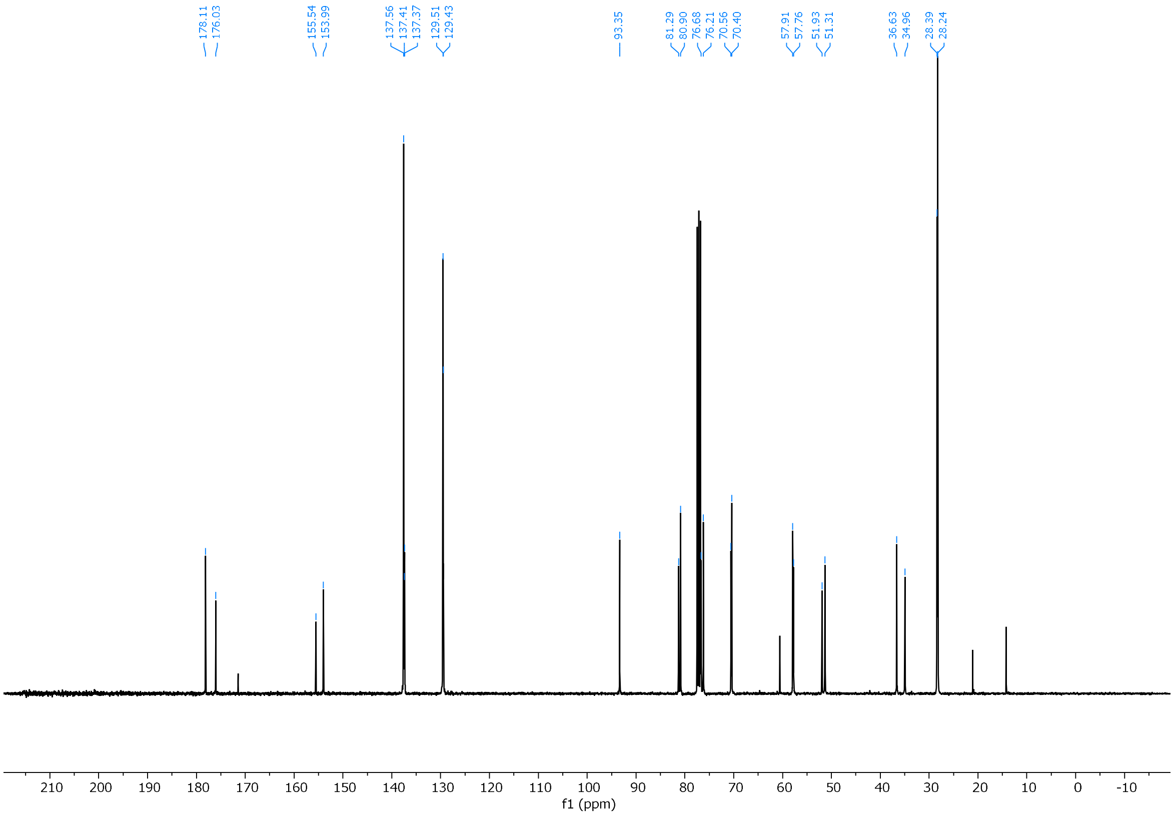

(2*R*,4*R*)-1-(tert-butoxycarbonyl)-4-((4-iodobenzyl)oxy)pyrrolidine-2-carboxylic acid (**13ci**)

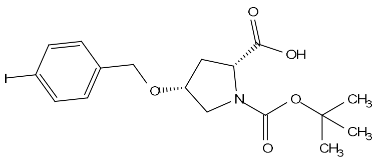

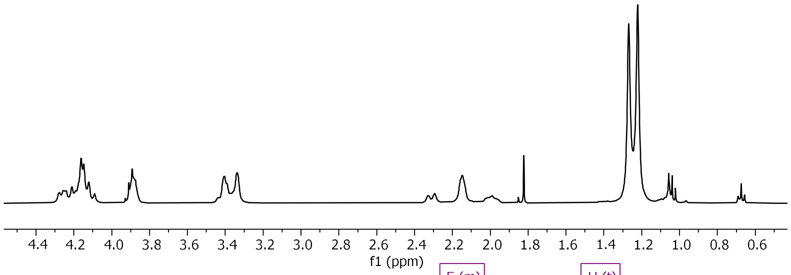

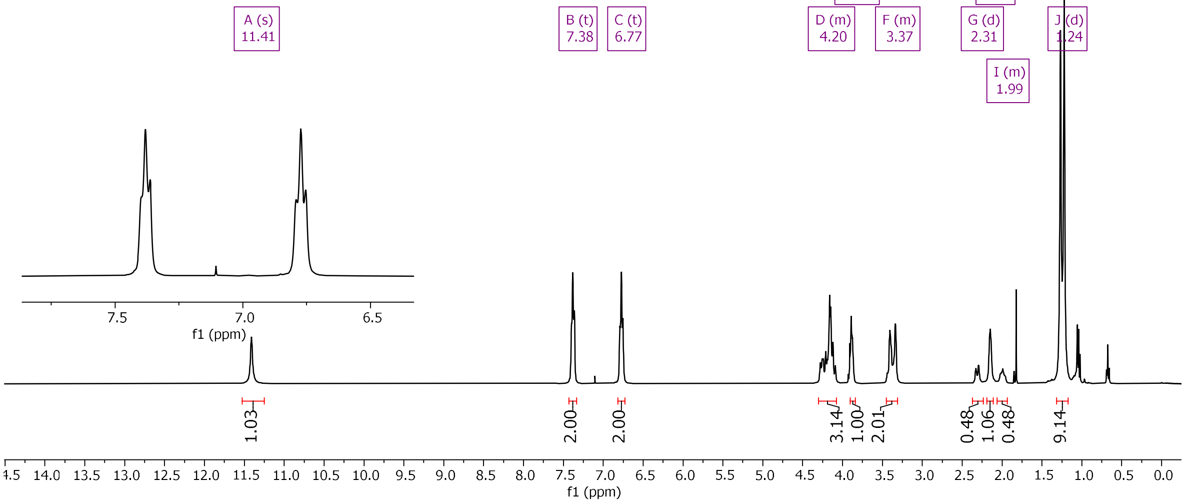

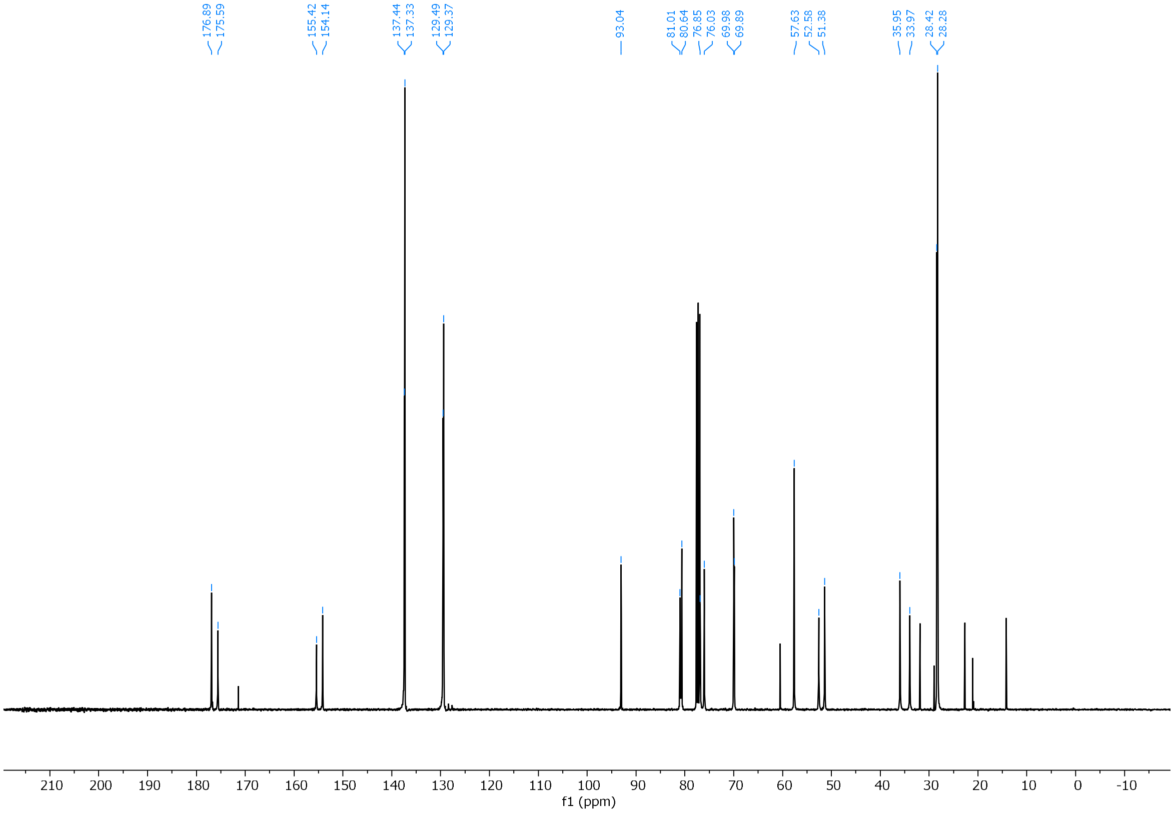

(2*R*,4*S*)-1-(tert-butoxycarbonyl)-4-((4-iodobenzyl)oxy)pyrrolidine-2-carboxylic acid (**13di**)

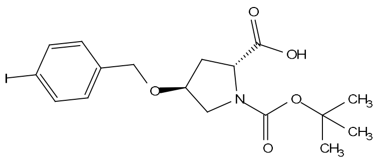

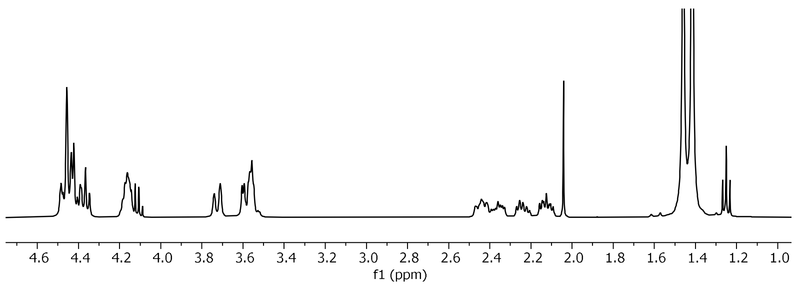

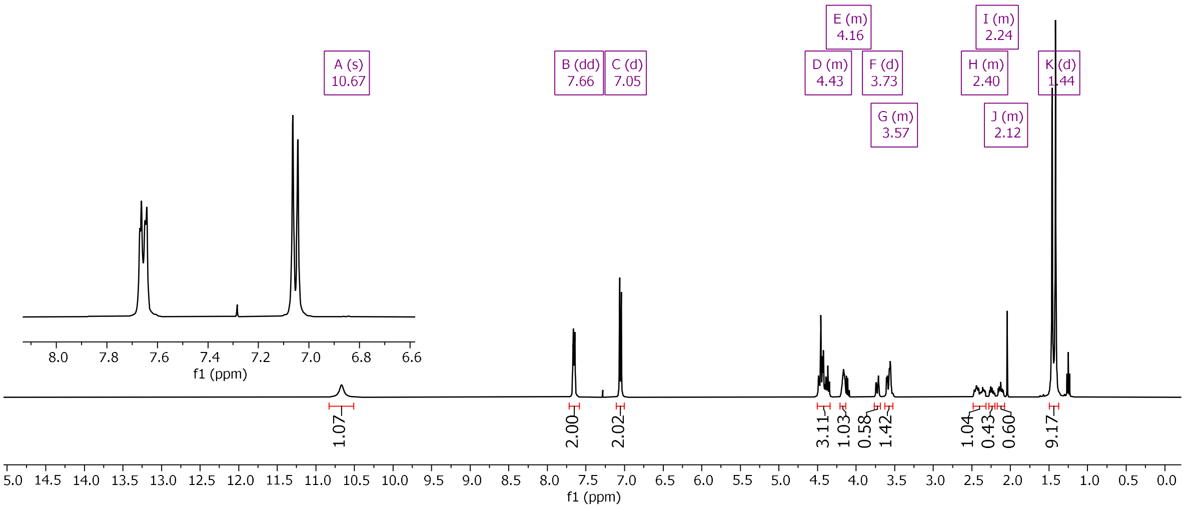

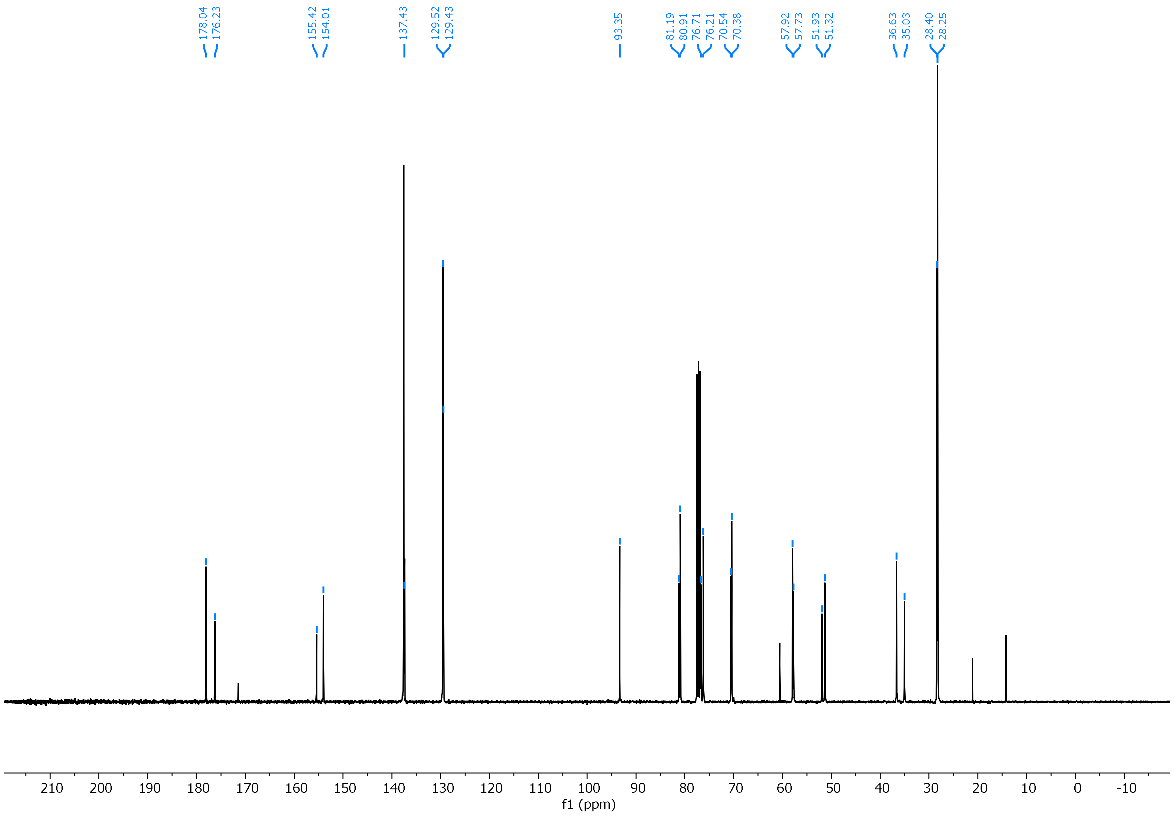

(2*R*,4*S*)-1-(*tert*-butoxycarbonyl)-4-((3-iodobenzyl)oxy)pyrrolidine-2-carboxylic acid (**14aii**)

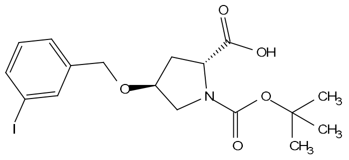

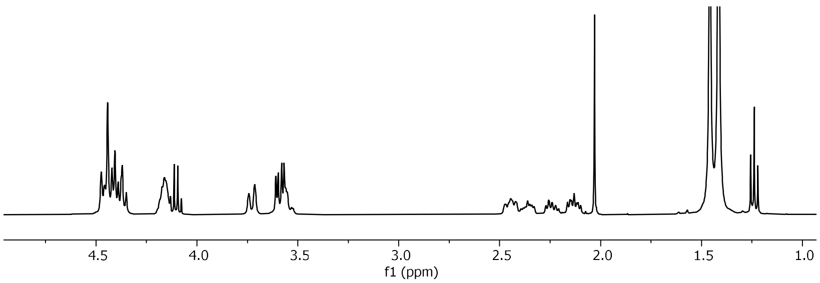

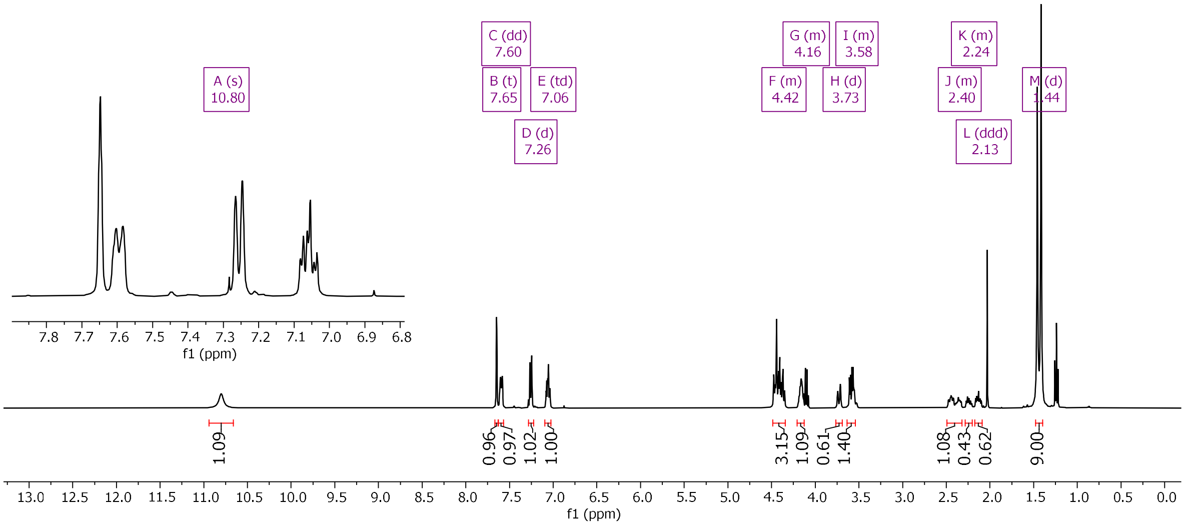

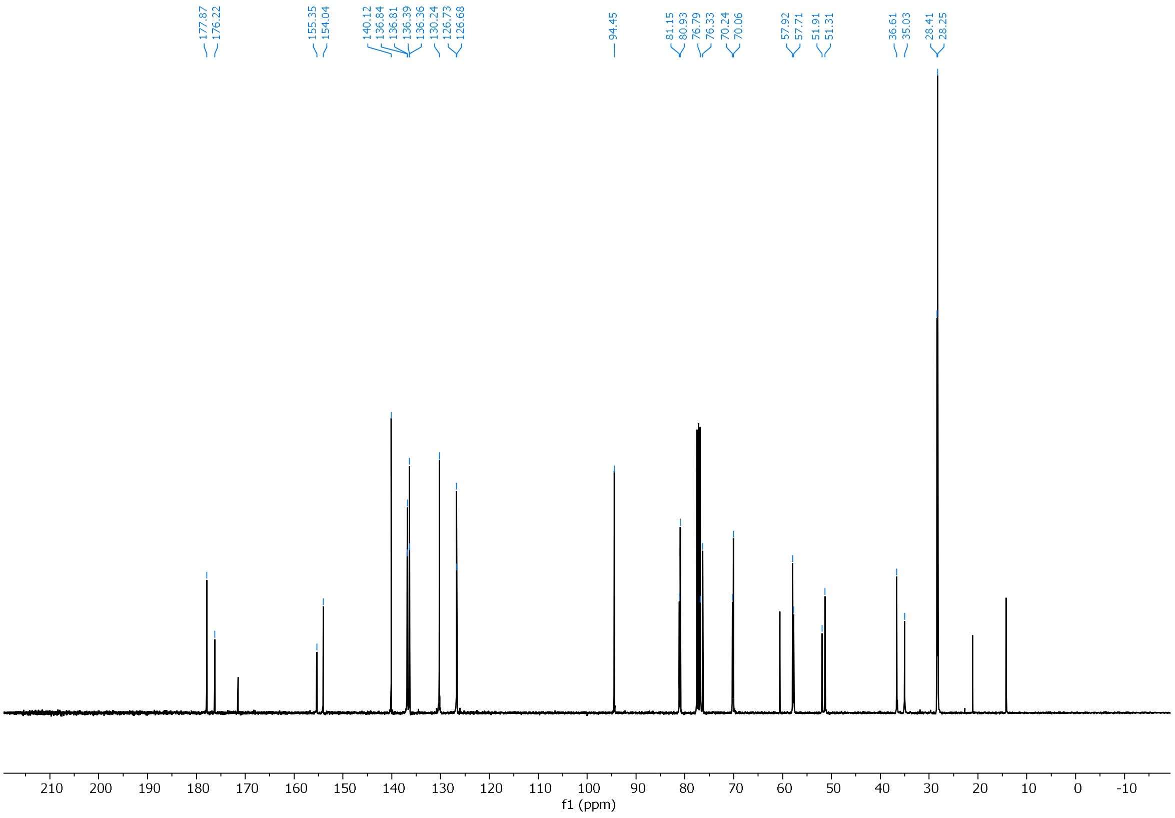

(2*S*,4*S*)-1-(*tert*-butoxycarbonyl)-4-((3-iodobenzyl)oxy)pyrrolidine-2-carboxylic acid (**14bii**)

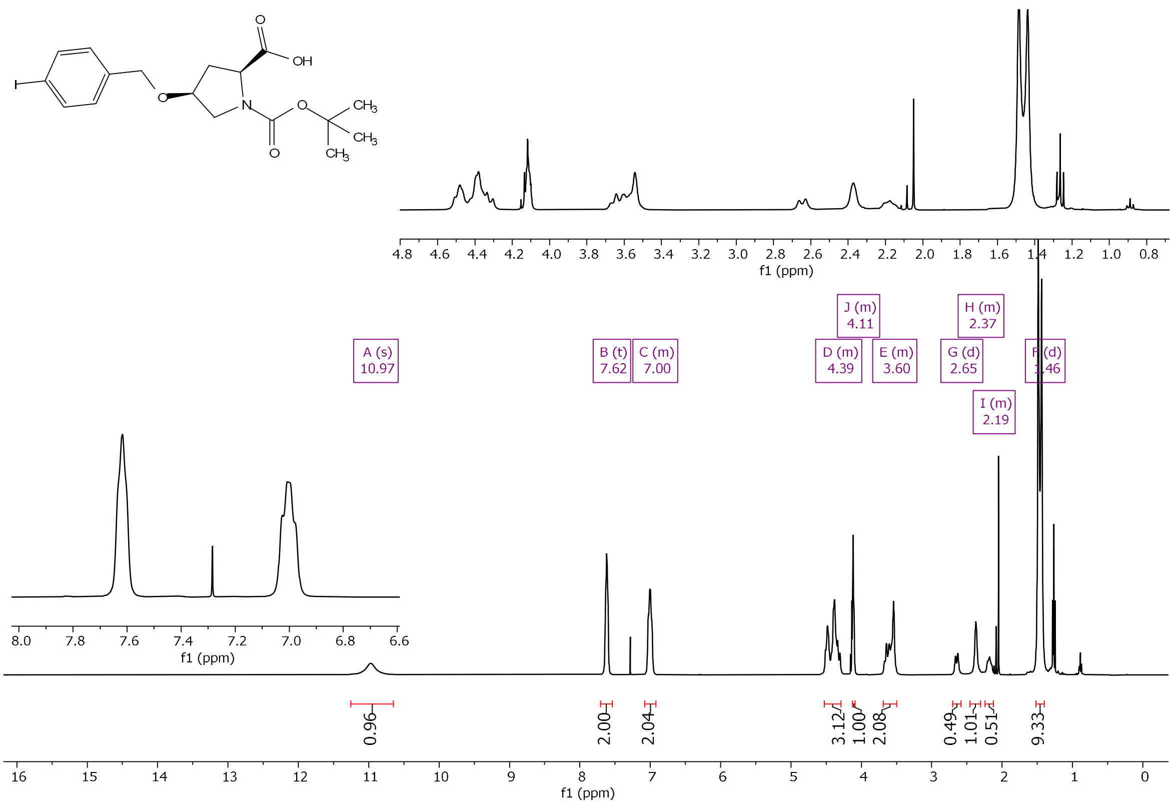

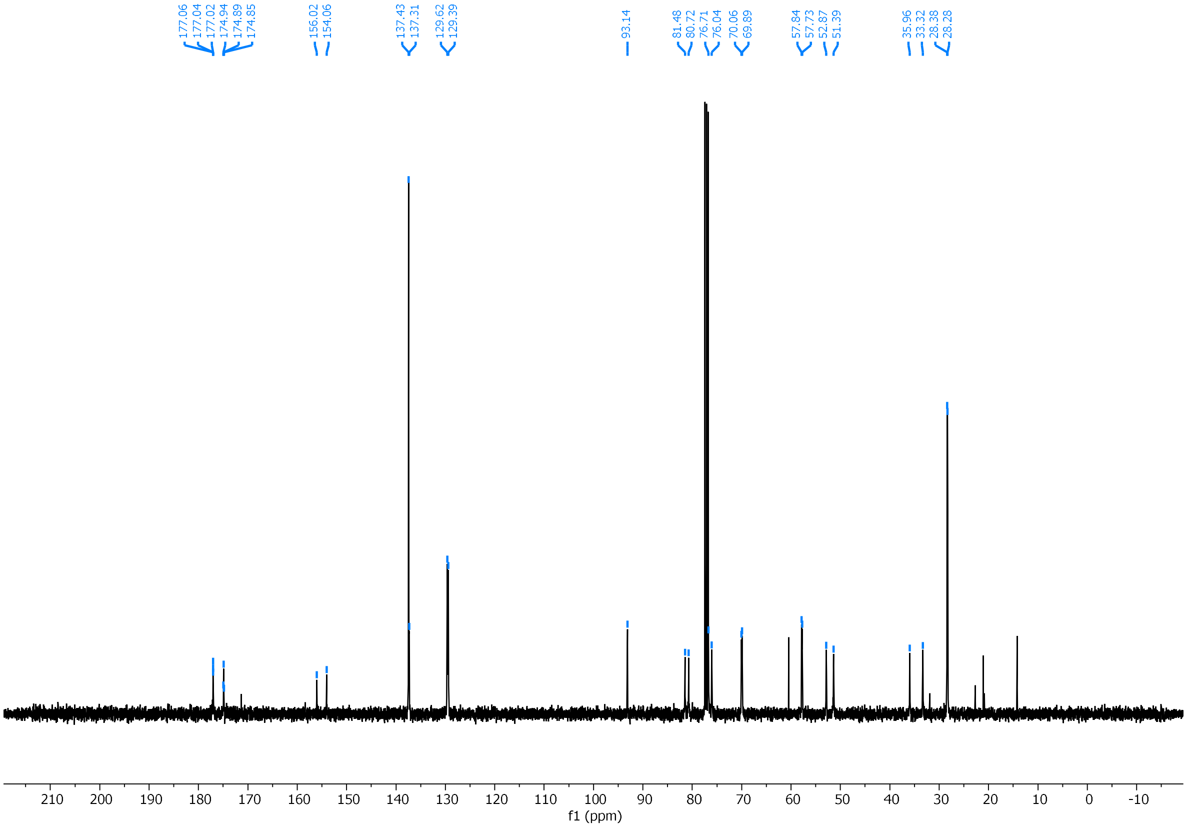

(2*S*,4*R*)-1-(*tert*-butoxycarbonyl)-4-((3-iodobenzyl)oxy)pyrrolidine-2-carboxylic acid (**14cii**)

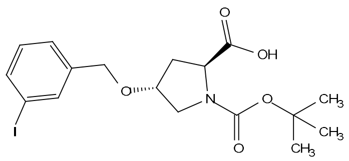

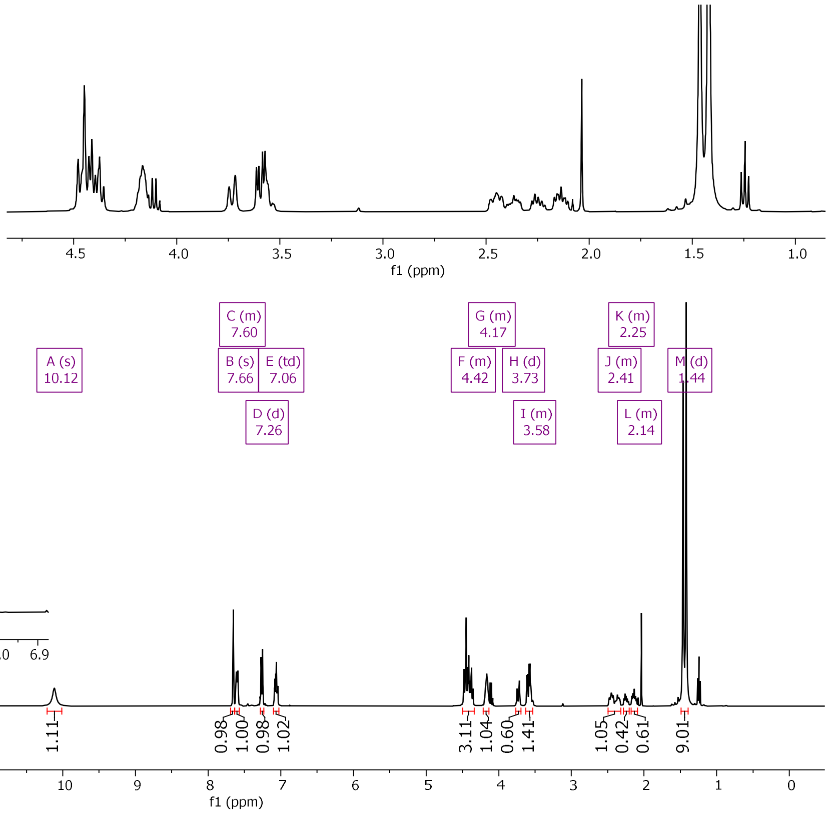

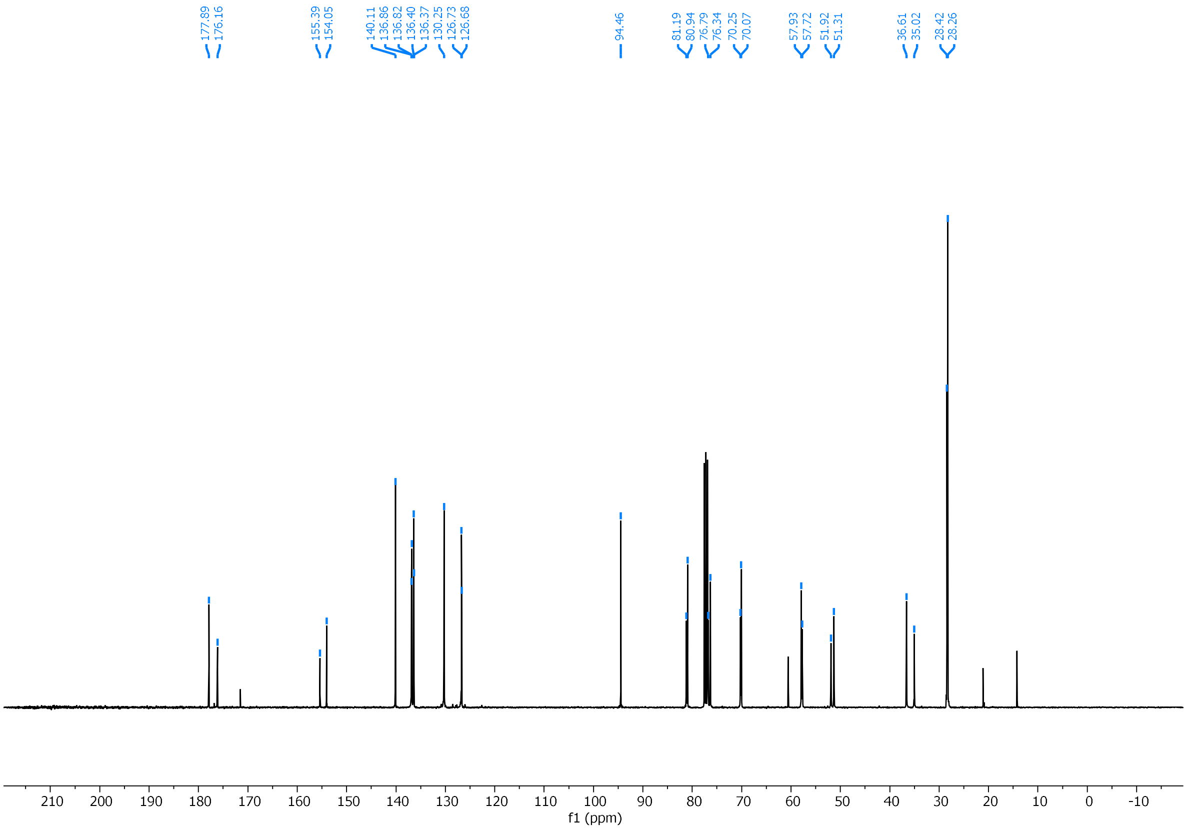

(2*R*,4*R*)-1-(*tert*-butoxycarbonyl)-4-((3-iodobenzyl)oxy)pyrrolidine-2-carboxylic acid (**14dii**)

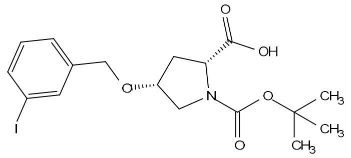

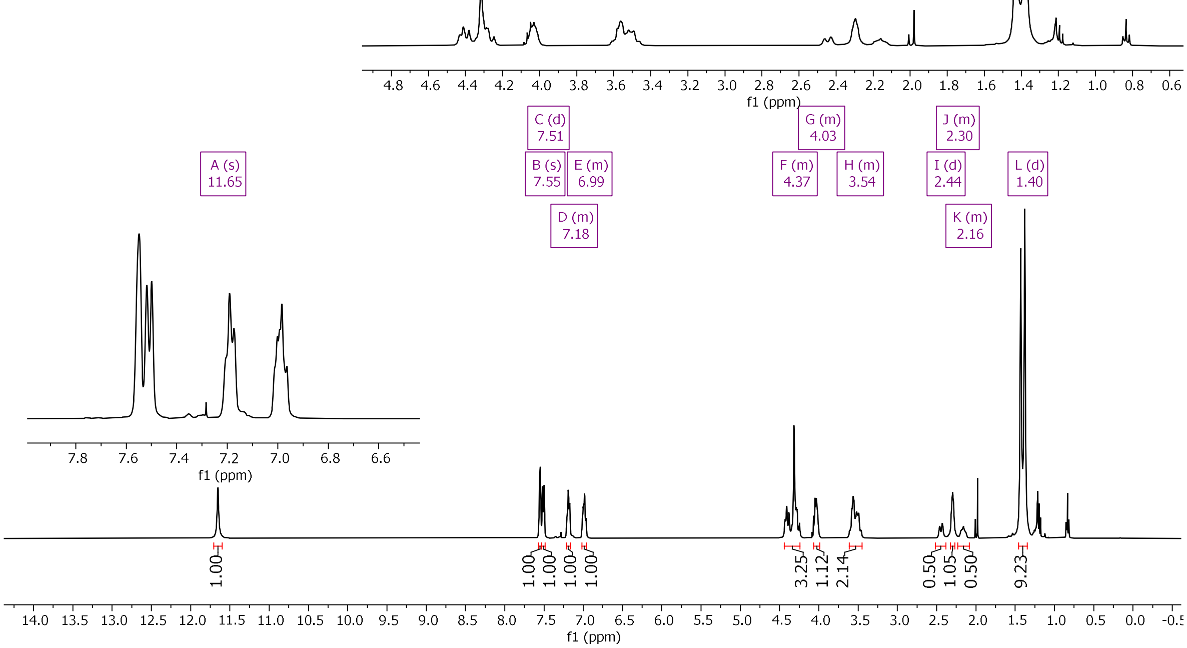

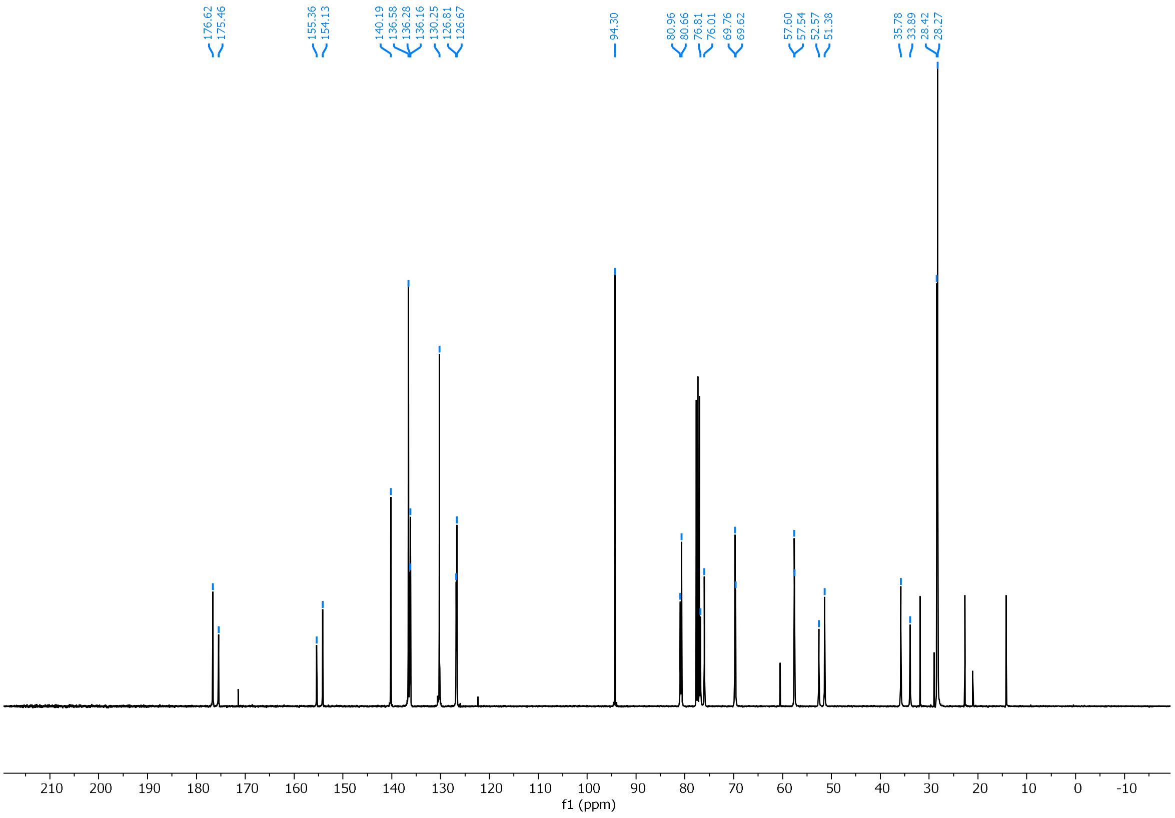

(2*S*,4*S*)-1-(((9H-fluoren-9-yl)methoxy)carbonyl)-4-(4-iodophenoxy)pyrrolidine-2-carboxylic acid (**10ai**)

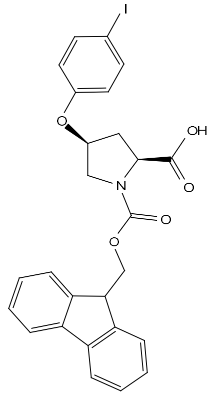

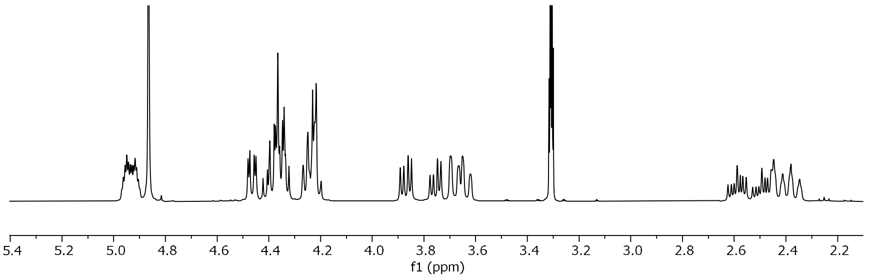

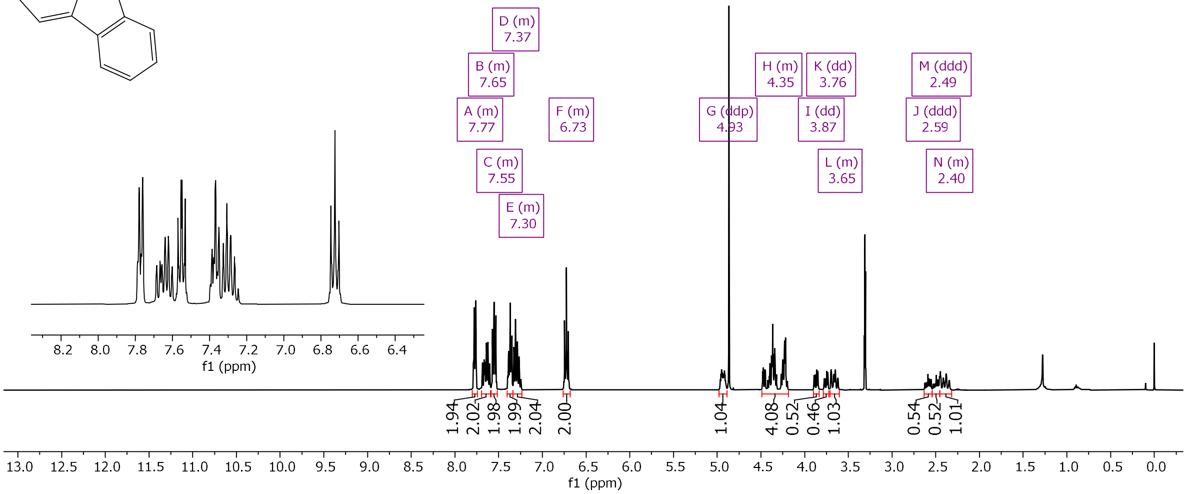

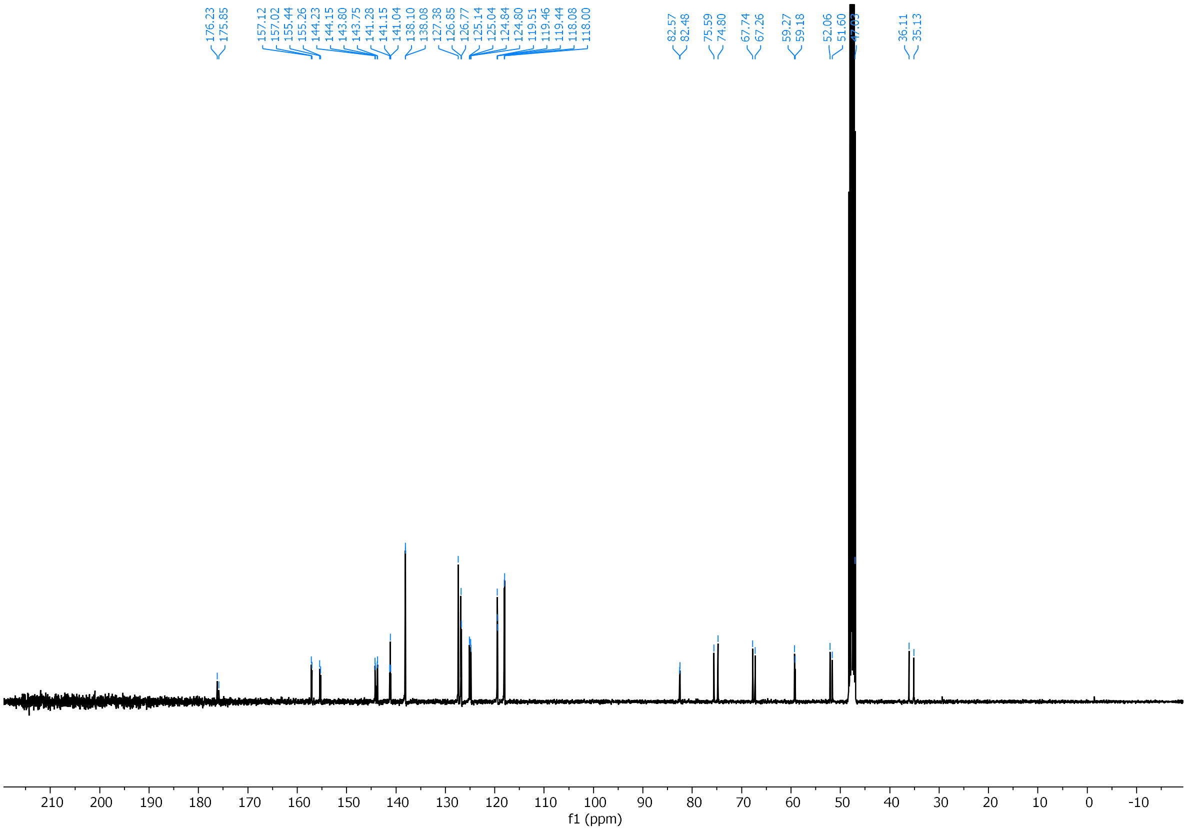

(2*S*,4*R*)-1-(((9H-fluoren-9-yl)methoxy)carbonyl)-4-(4-iodophenoxy)pyrrolidine-2-carboxylic acid (**10bi**)

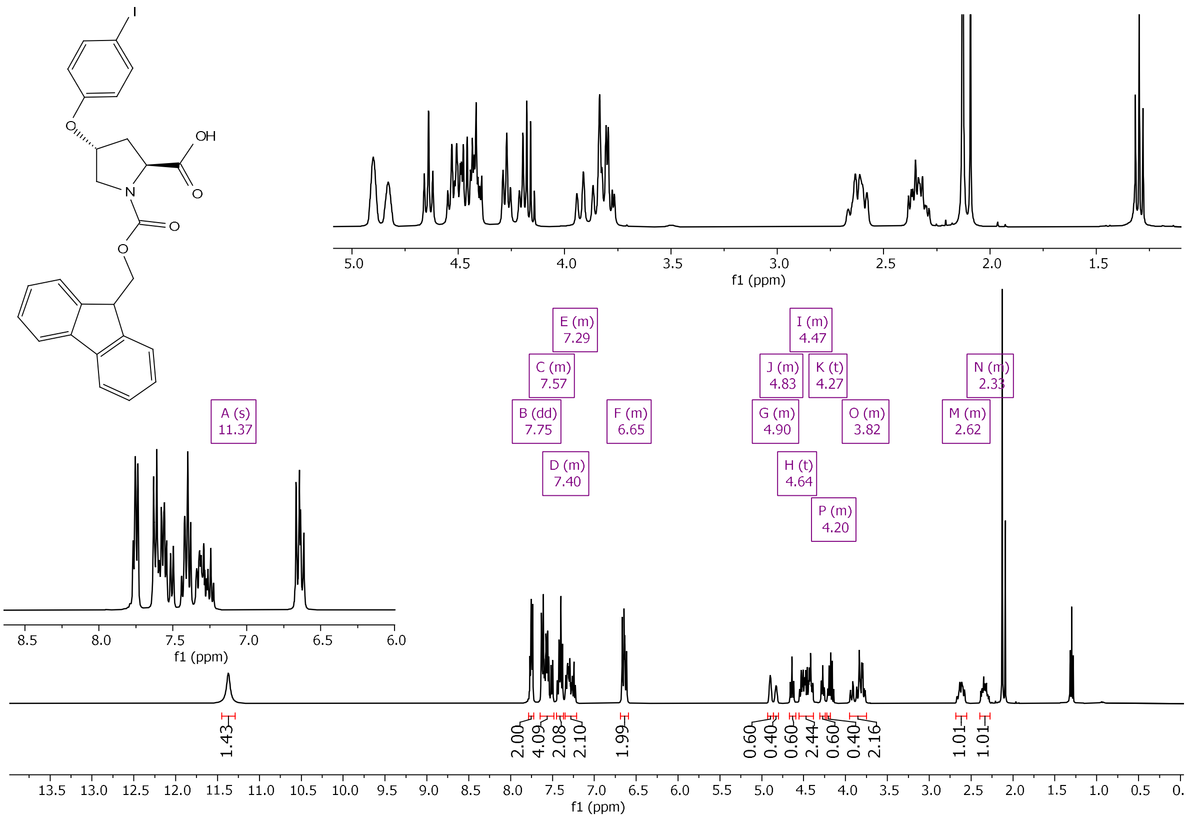

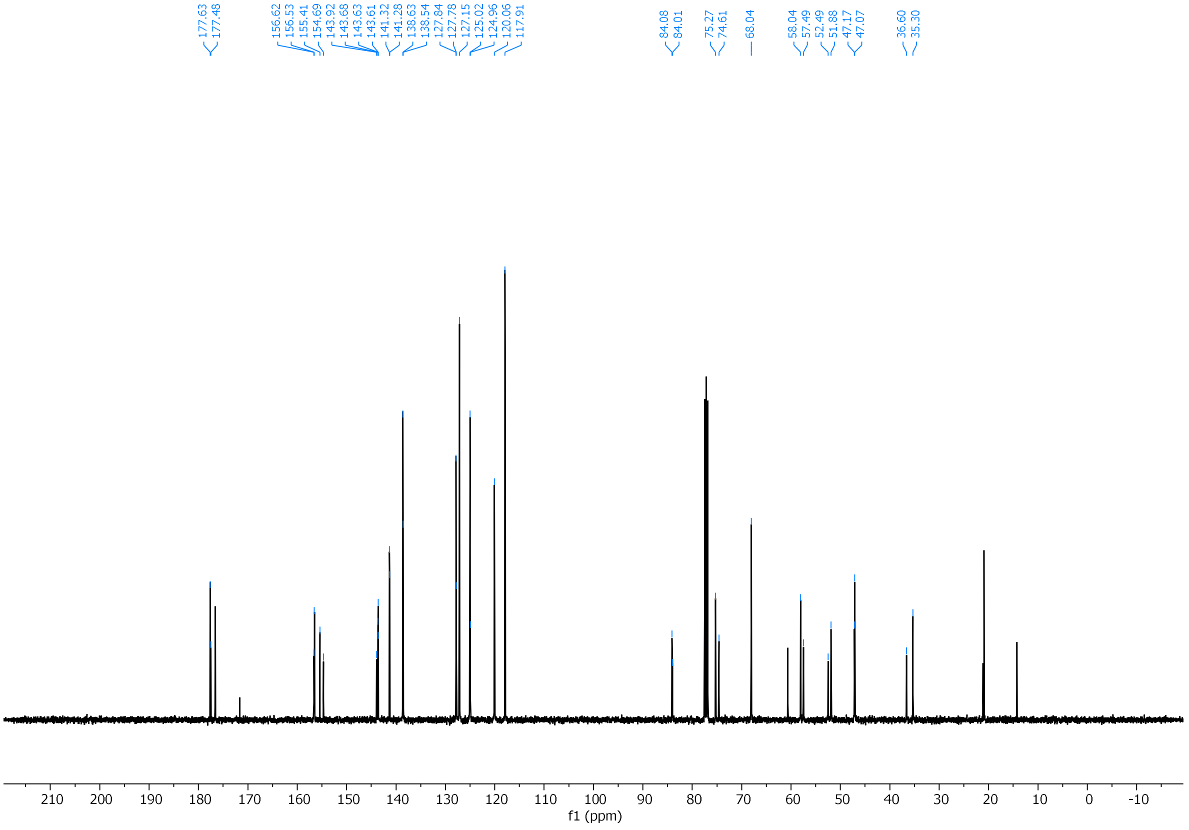

(2*R*,4*R*)-1-(((9H-fluoren-9-yl)methoxy)carbonyl)-4-(4-iodophenoxy)pyrrolidine-2-carboxylic acid (**10ci**)

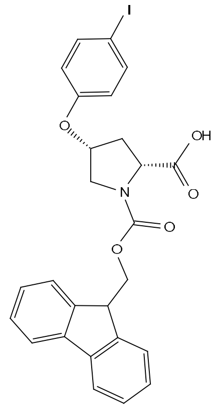

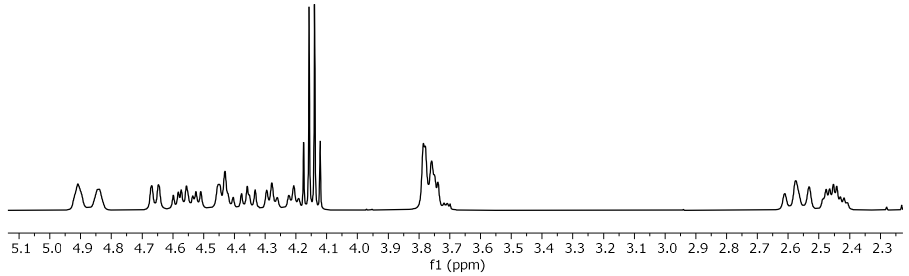

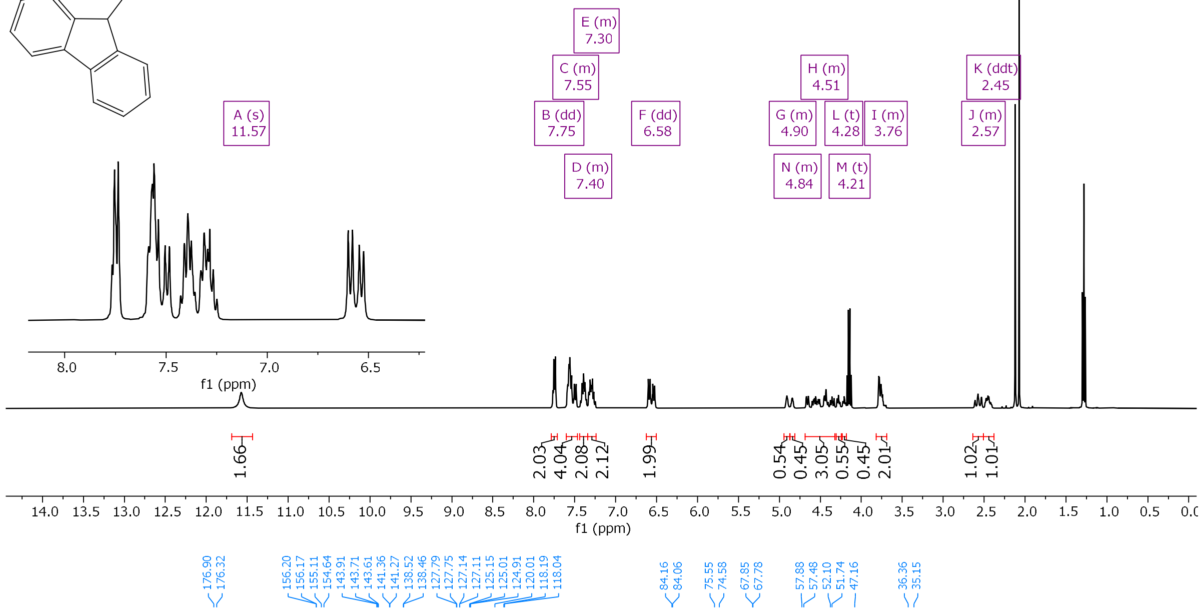

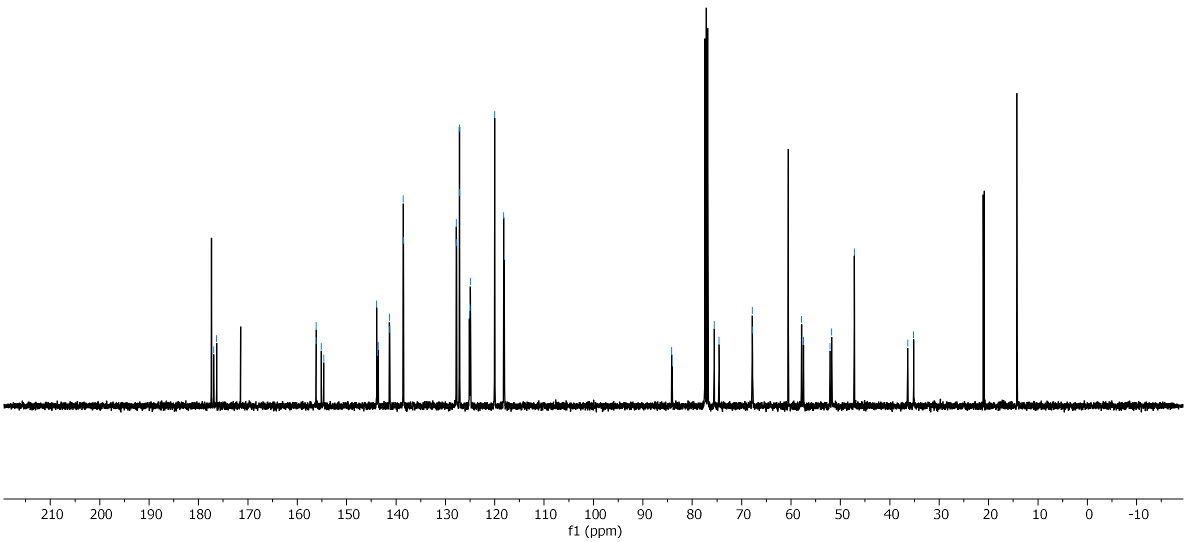

(2*R*,4*S*)-1-(((9H-fluoren-9-yl)methoxy)carbonyl)-4-(4-iodophenoxy)pyrrolidine-2-carboxylic acid (**10di**)

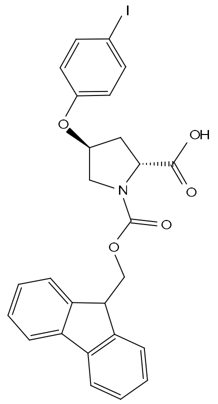

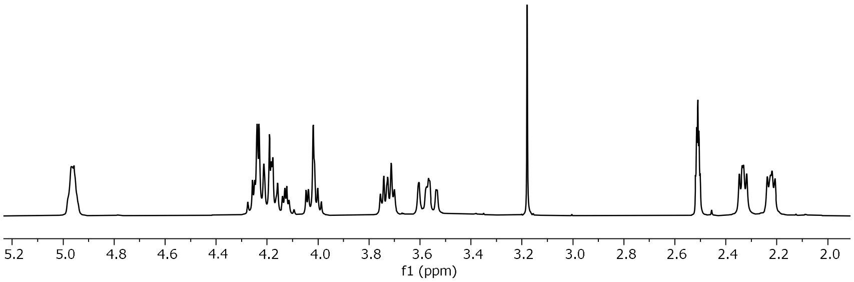

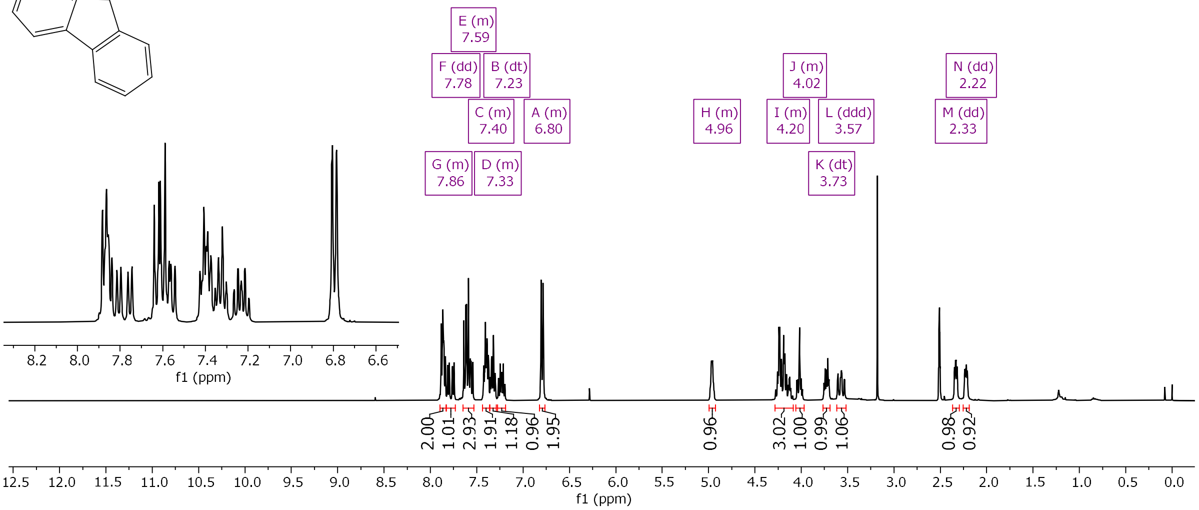

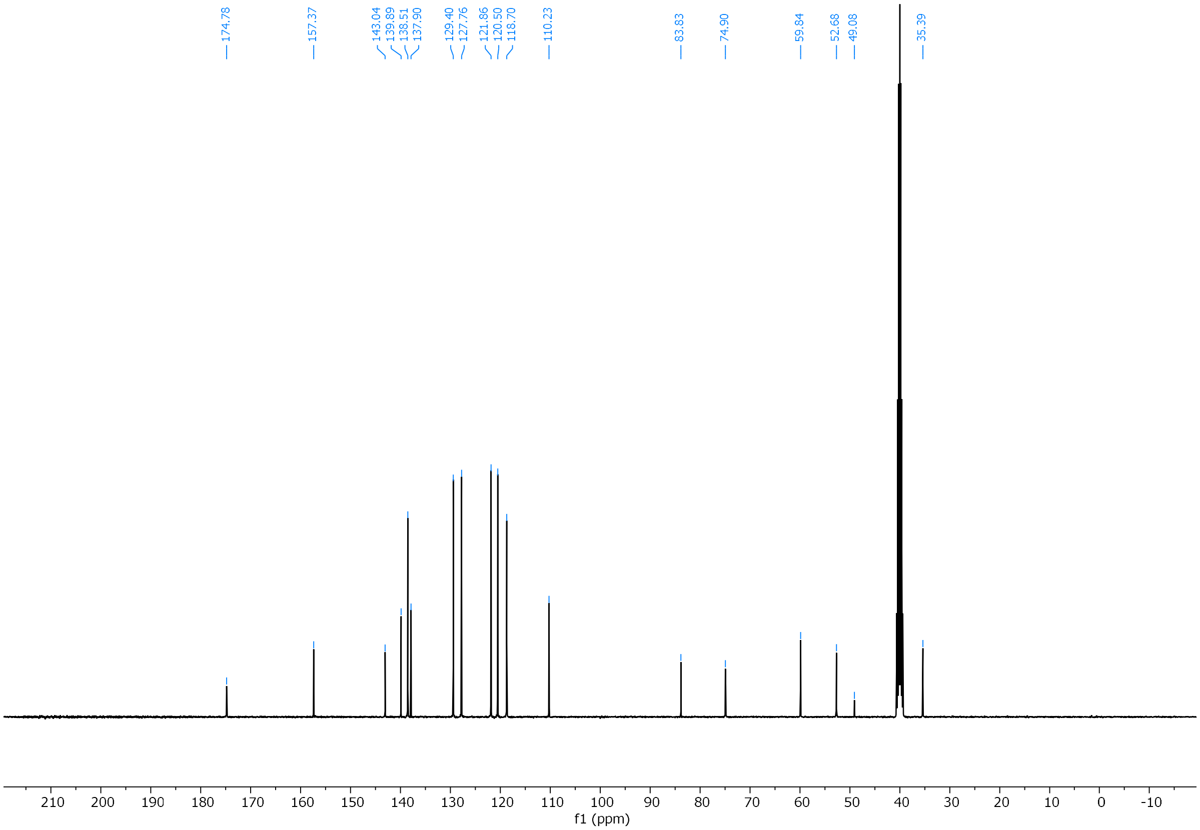

(2*R*,4*S*)-1-(((9H-fluoren-9-yl)methoxy)carbonyl)-4-(4-iodophenoxy)pyrrolidine-2-carboxylic acid (**10aii**)

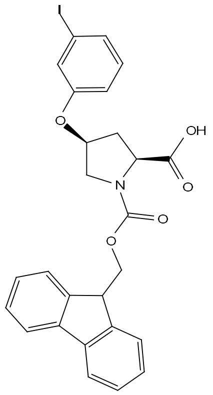

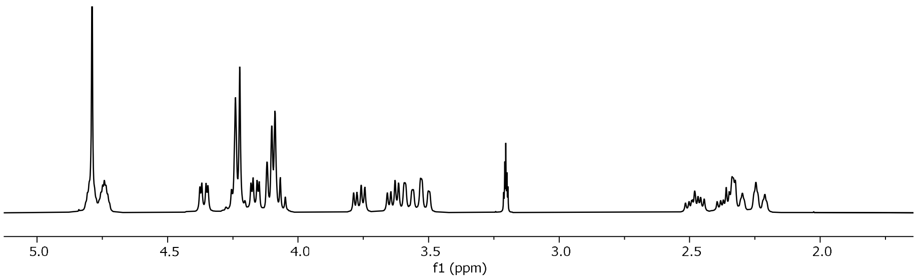

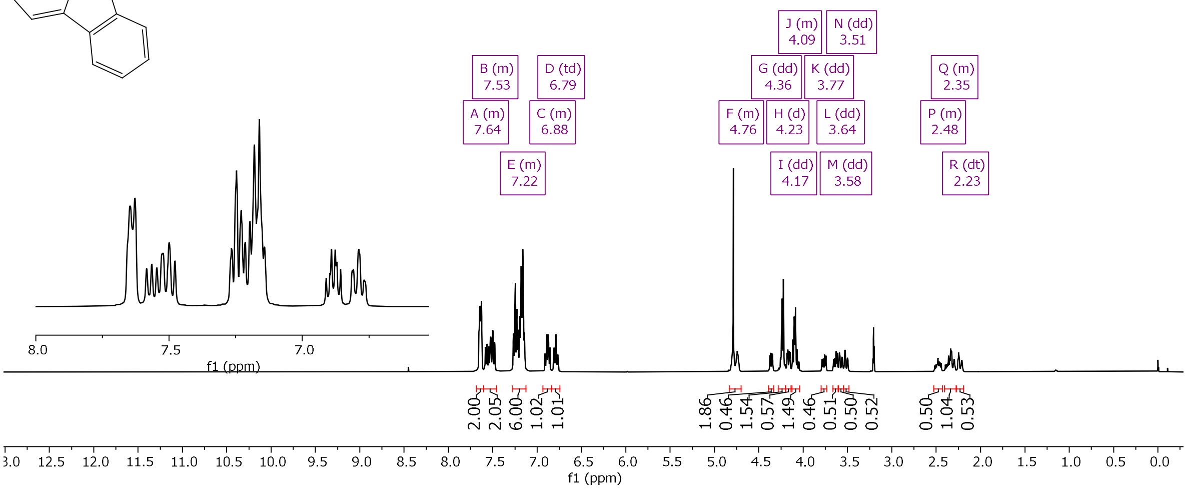

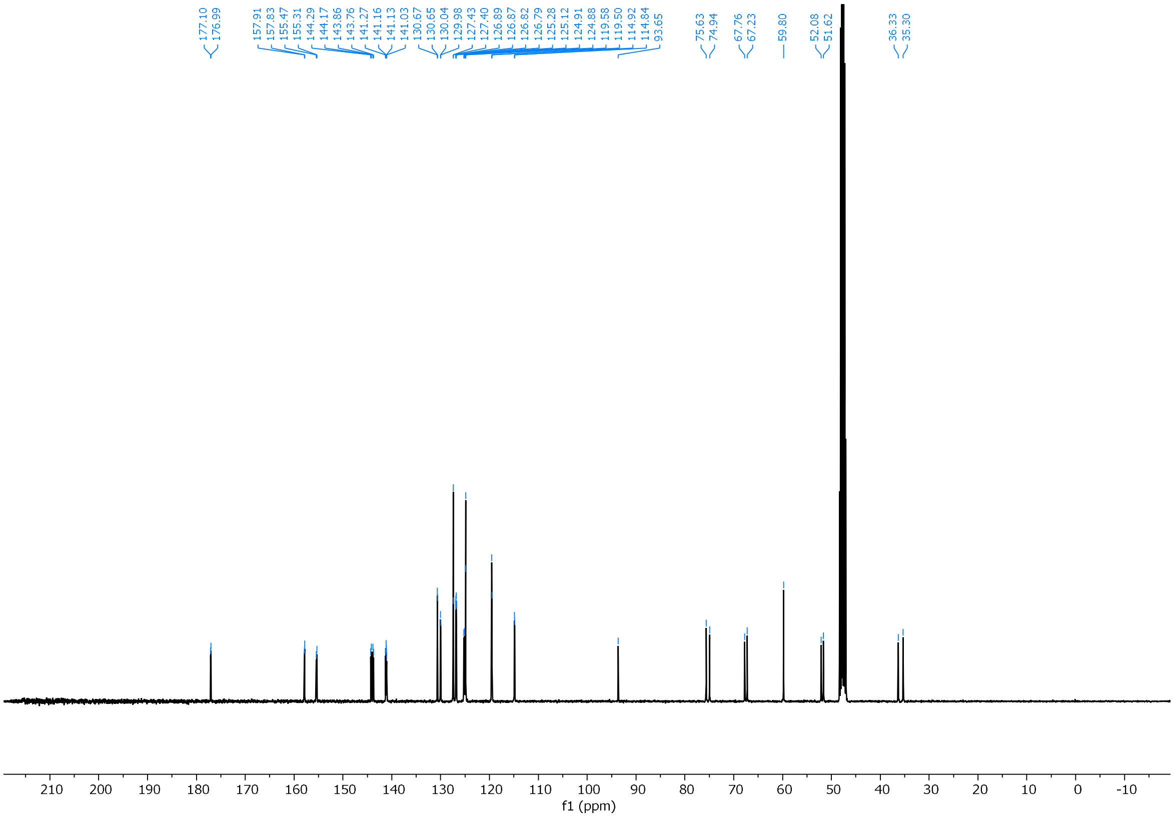

(2*S*,4*R*)-1-(((9H-fluoren-9-yl)methoxy)carbonyl)-4-(3-iodophenoxy)pyrrolidine-2-carboxylic acid (**10bii**)

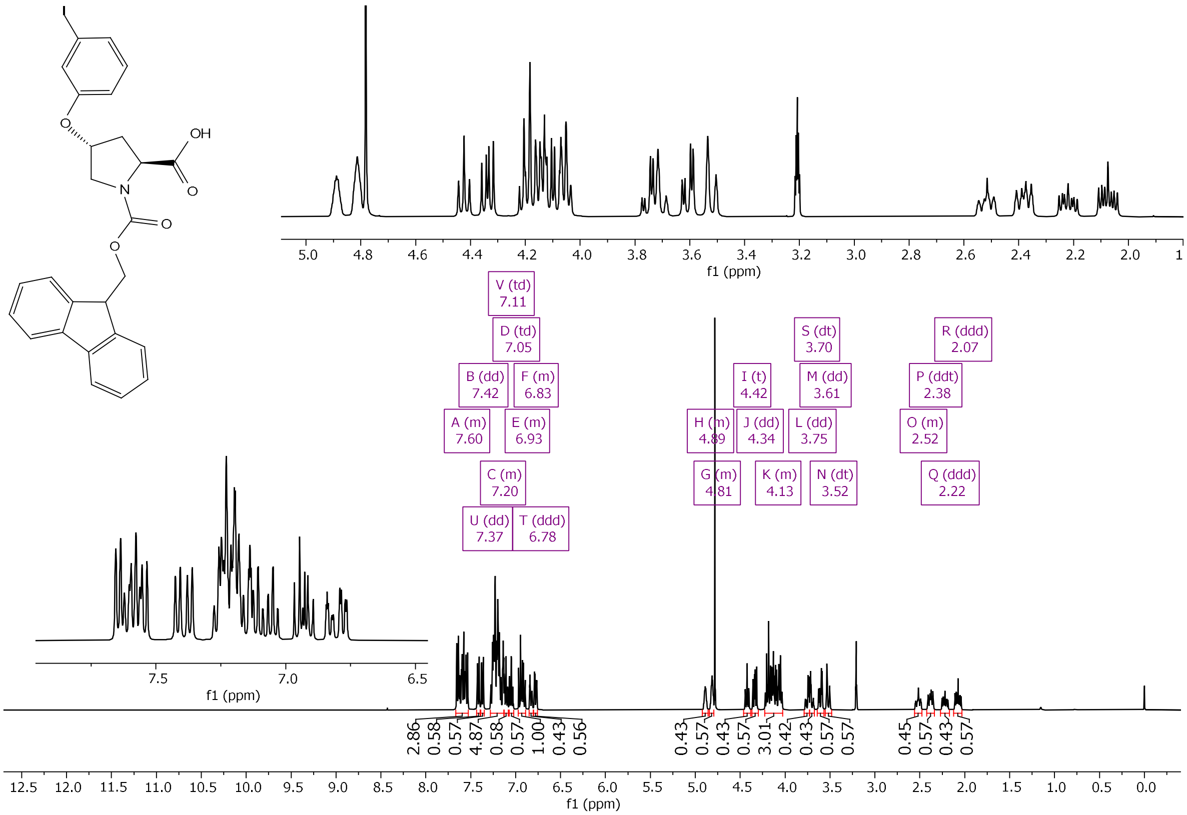

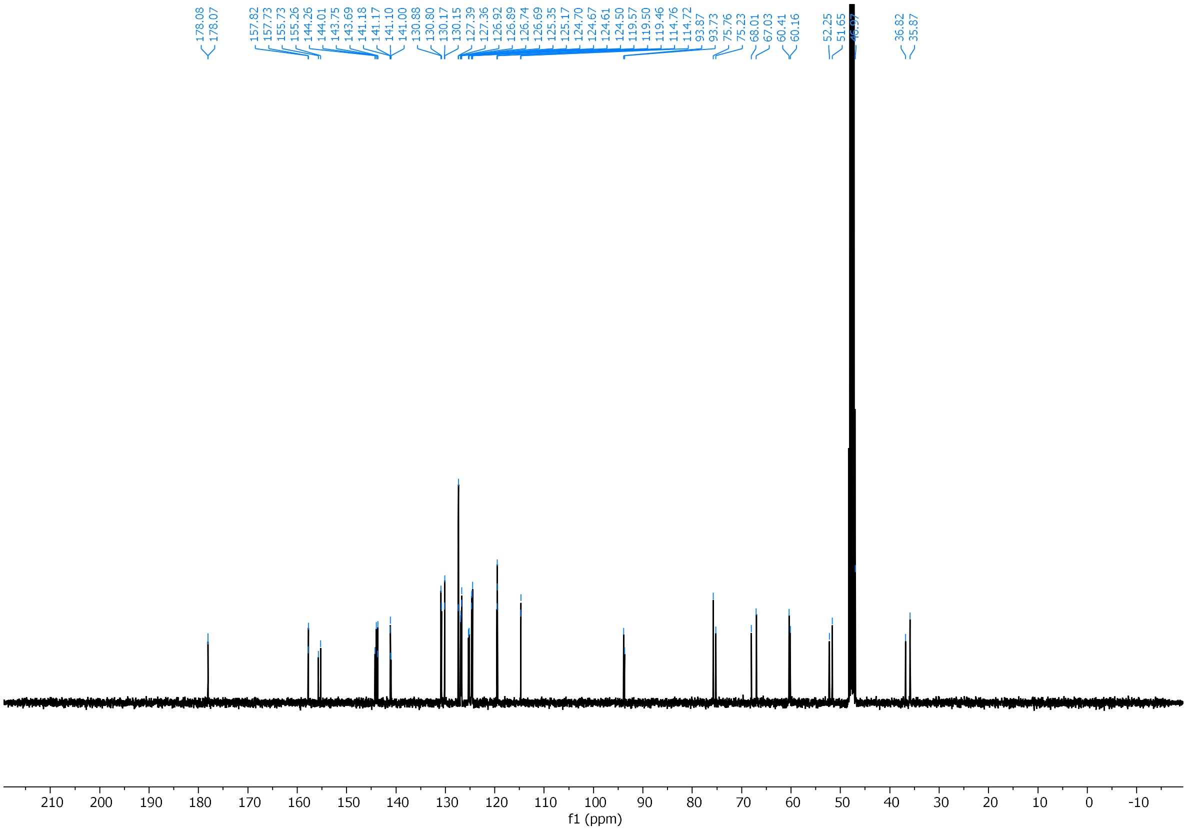

(2*R*,4*R*)-1-(((9H-fluoren-9-yl)methoxy)carbonyl)-4-(3-iodophenoxy)pyrrolidine-2-carboxylic acid (**10cii**)

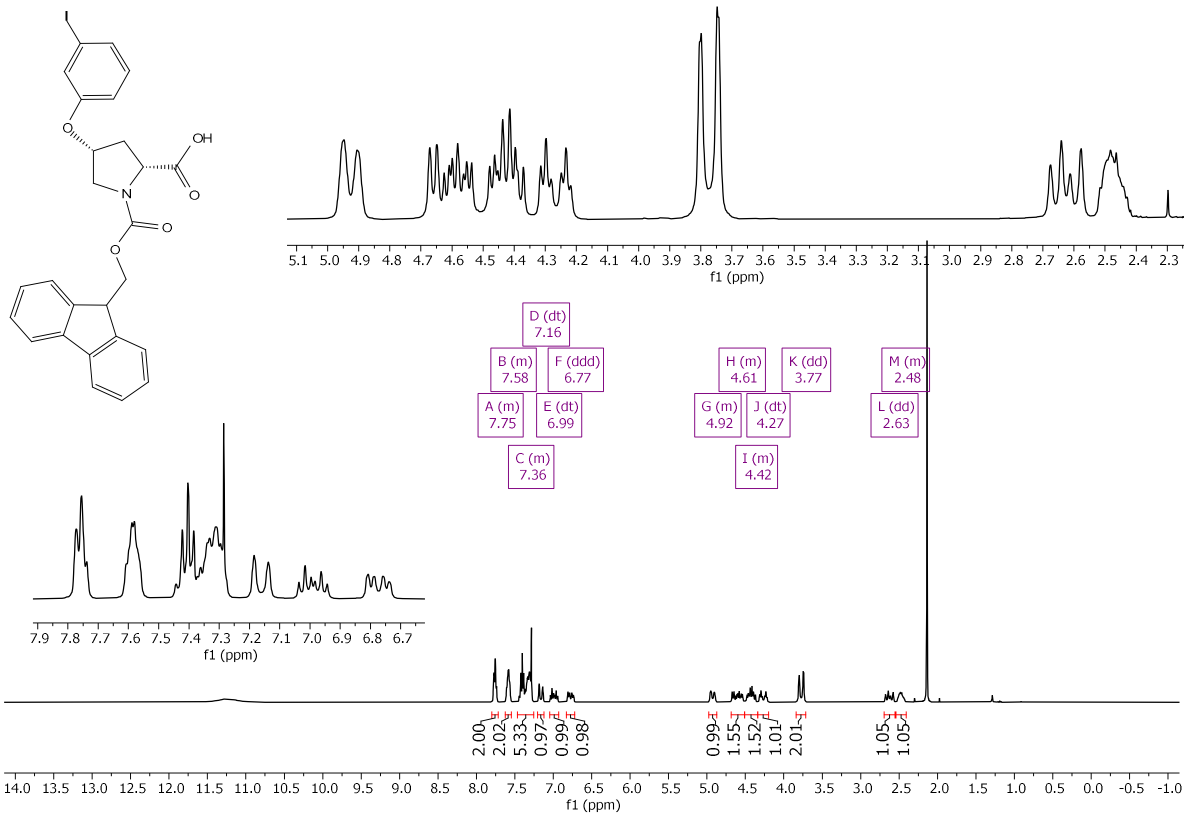

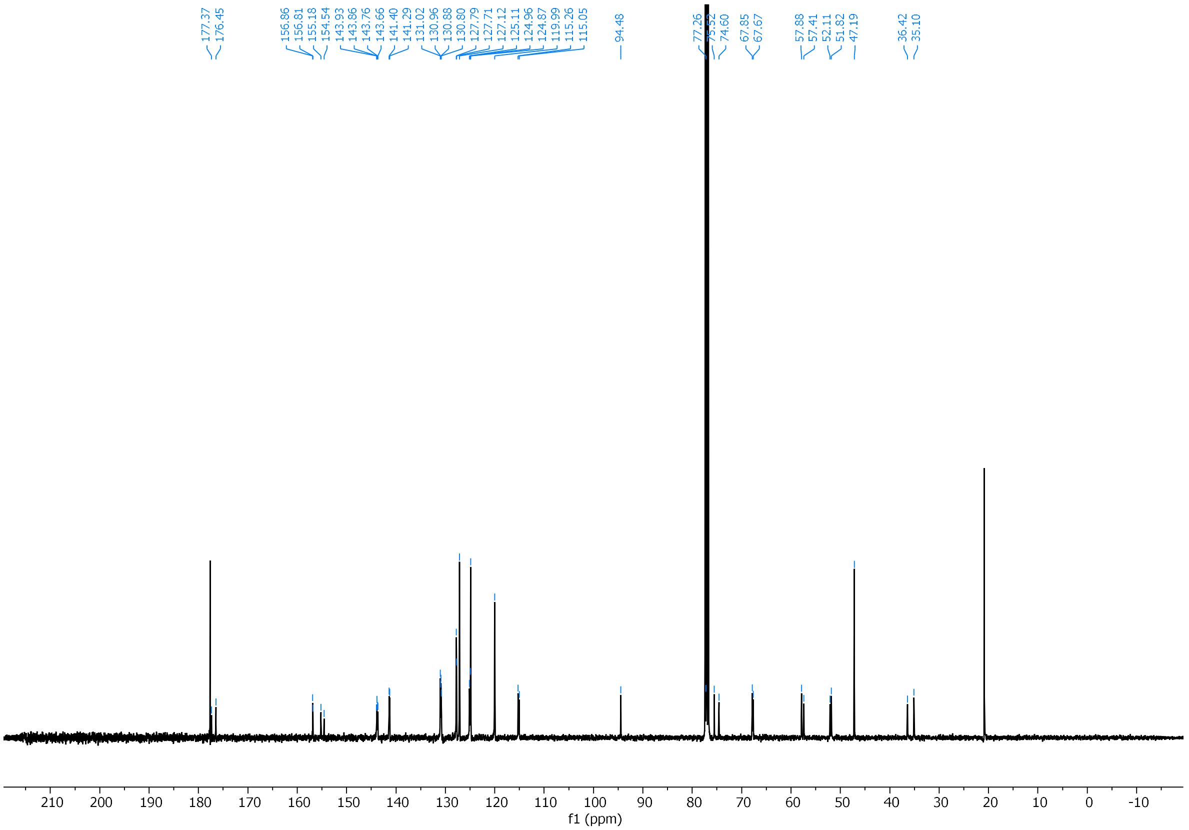

(2*R*,4*S*)-1-(((9H-fluoren-9-yl)methoxy)carbonyl)-4-(3-iodophenoxy)pyrrolidine-2-carboxylic acid (**10dii**)

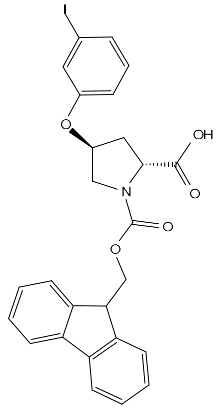

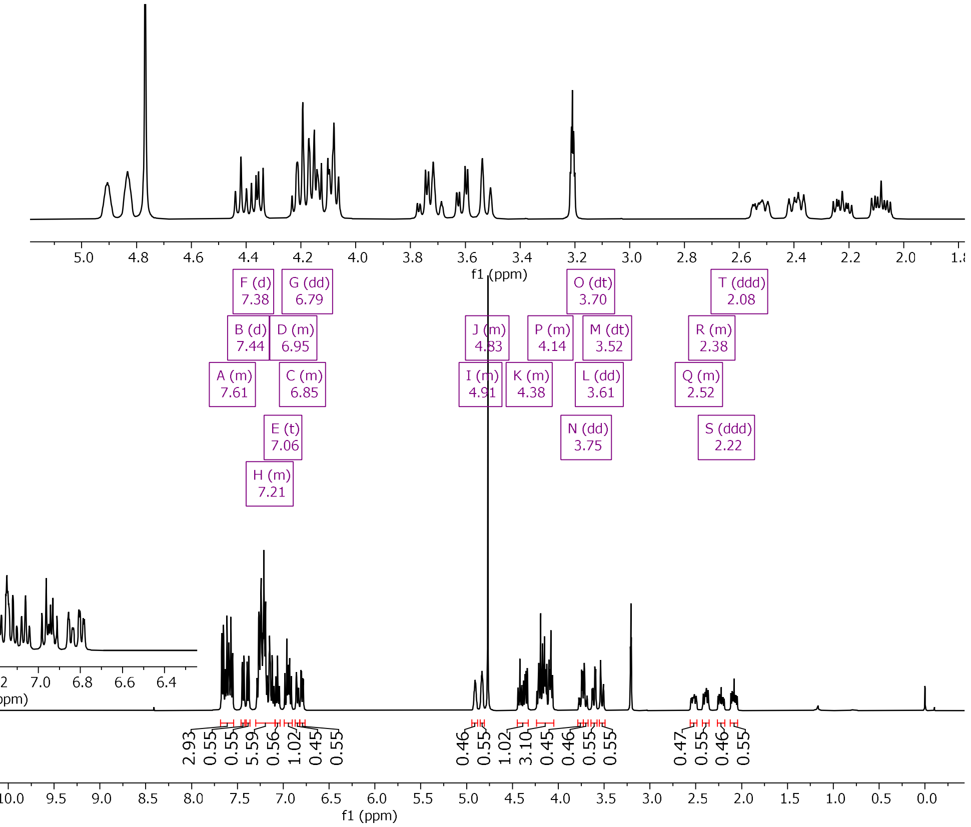

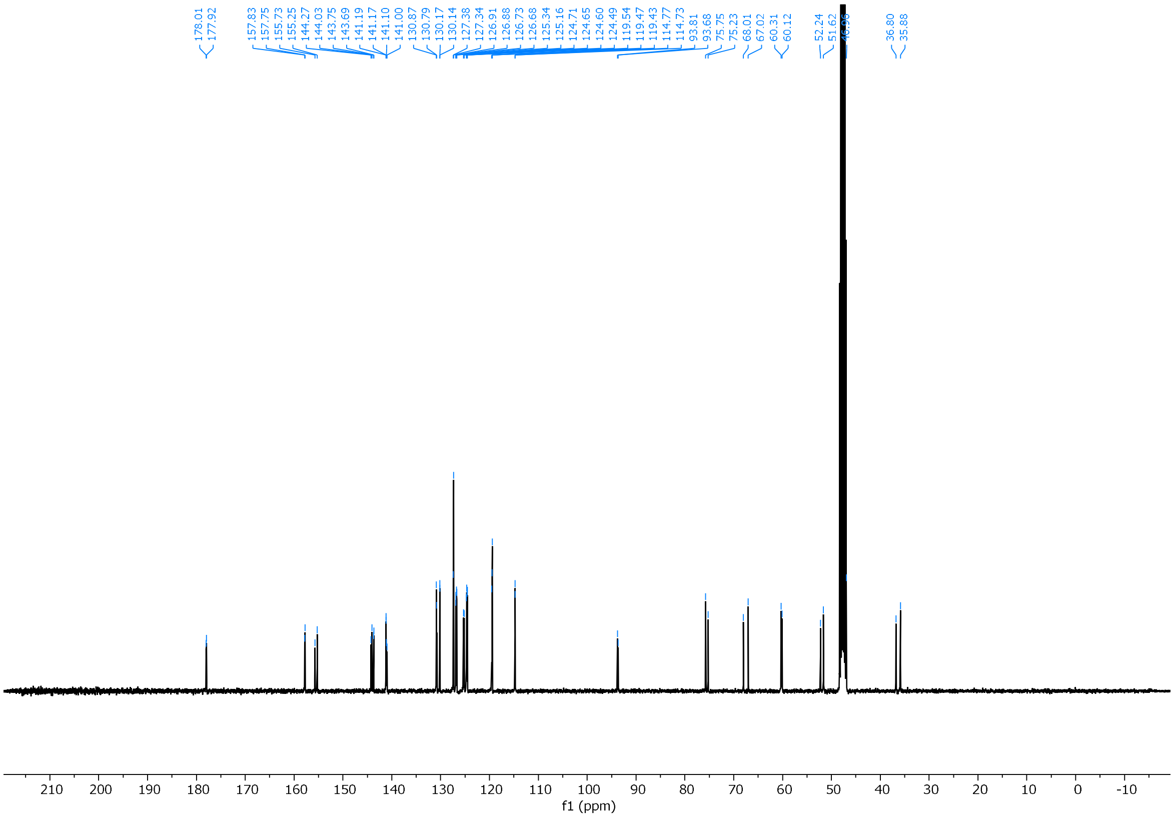

(2*S*,4*S*)-1-(((9H-fluoren-9-yl)methoxy)carbonyl)-4-((4-iodobenzyl)oxy)pyrrolidine-2-carboxylic acid (**14ai**)

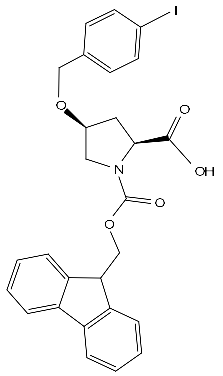

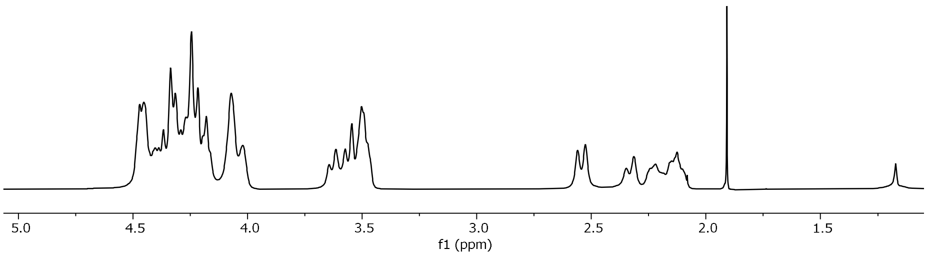

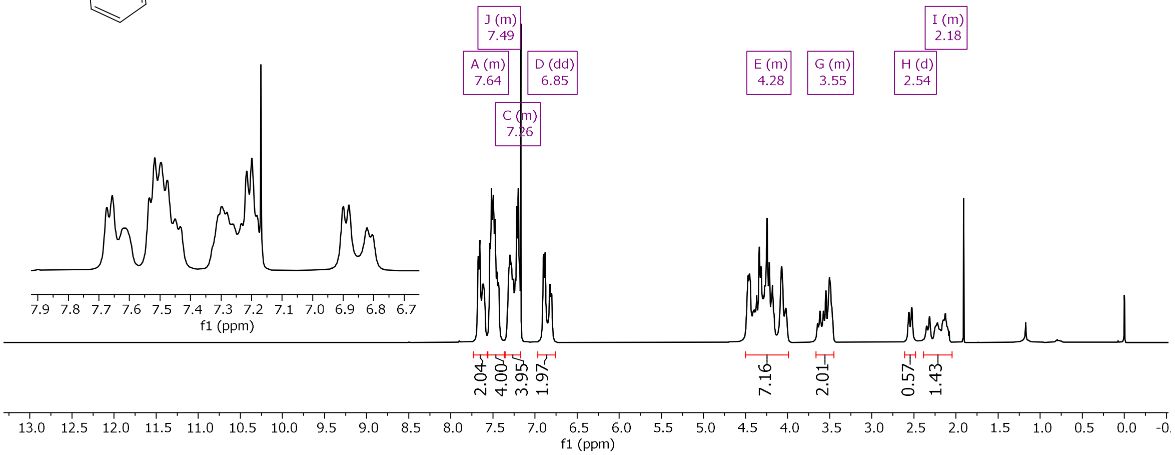

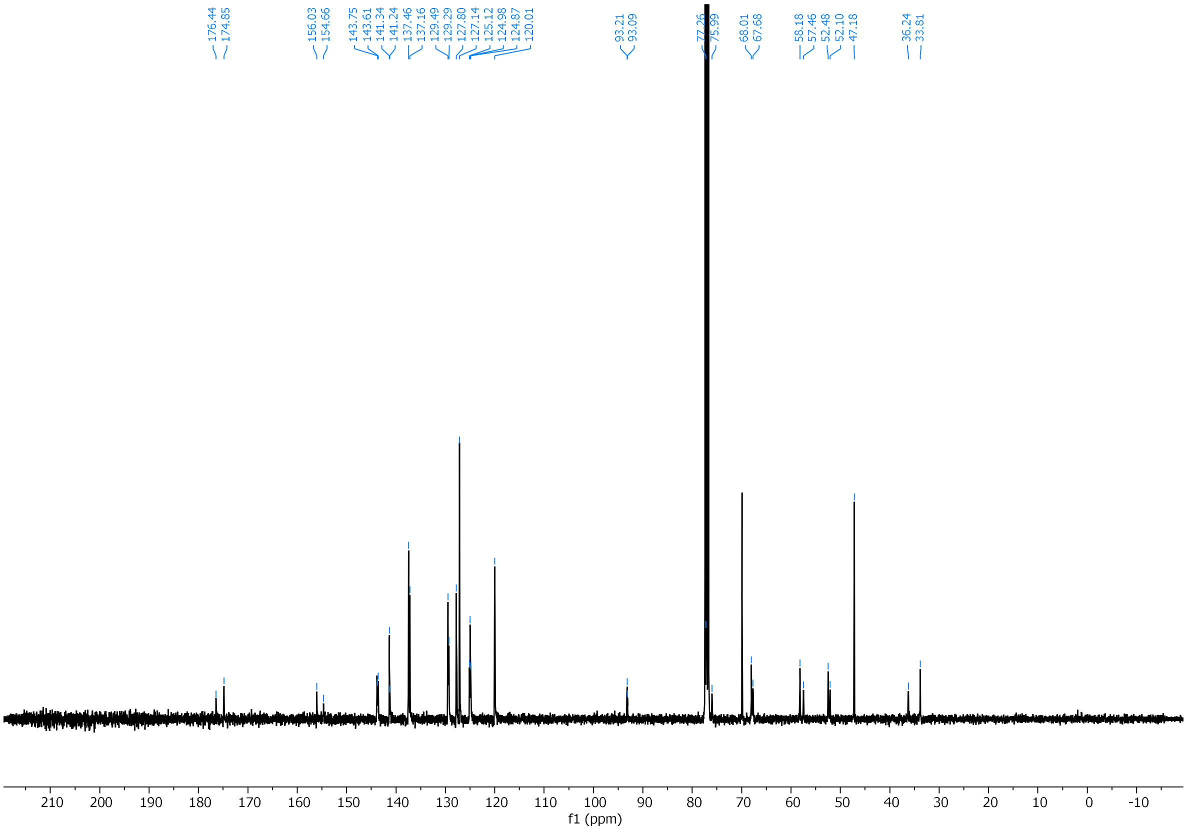

(2*S*,4*R*)-1-(((9H-fluoren-9-yl)methoxy)carbonyl)-4-((4-iodobenzyl)oxy)pyrrolidine-2-carboxylic acid (**14bi**)

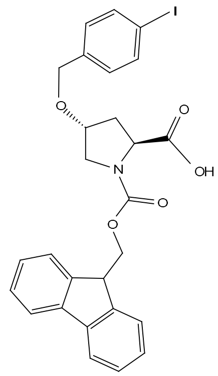

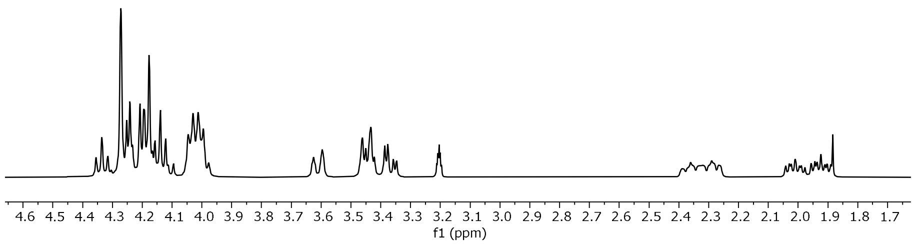

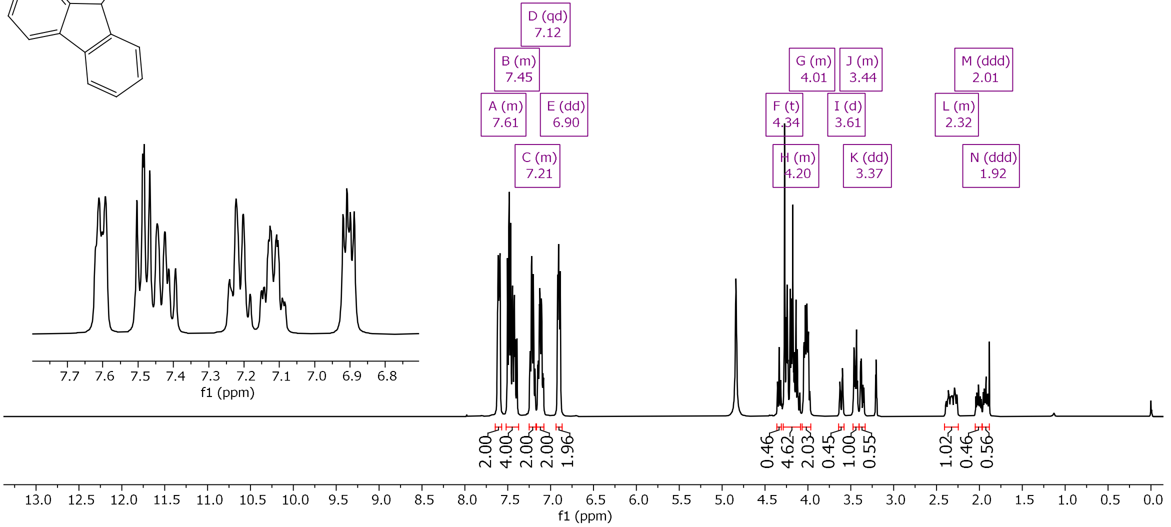

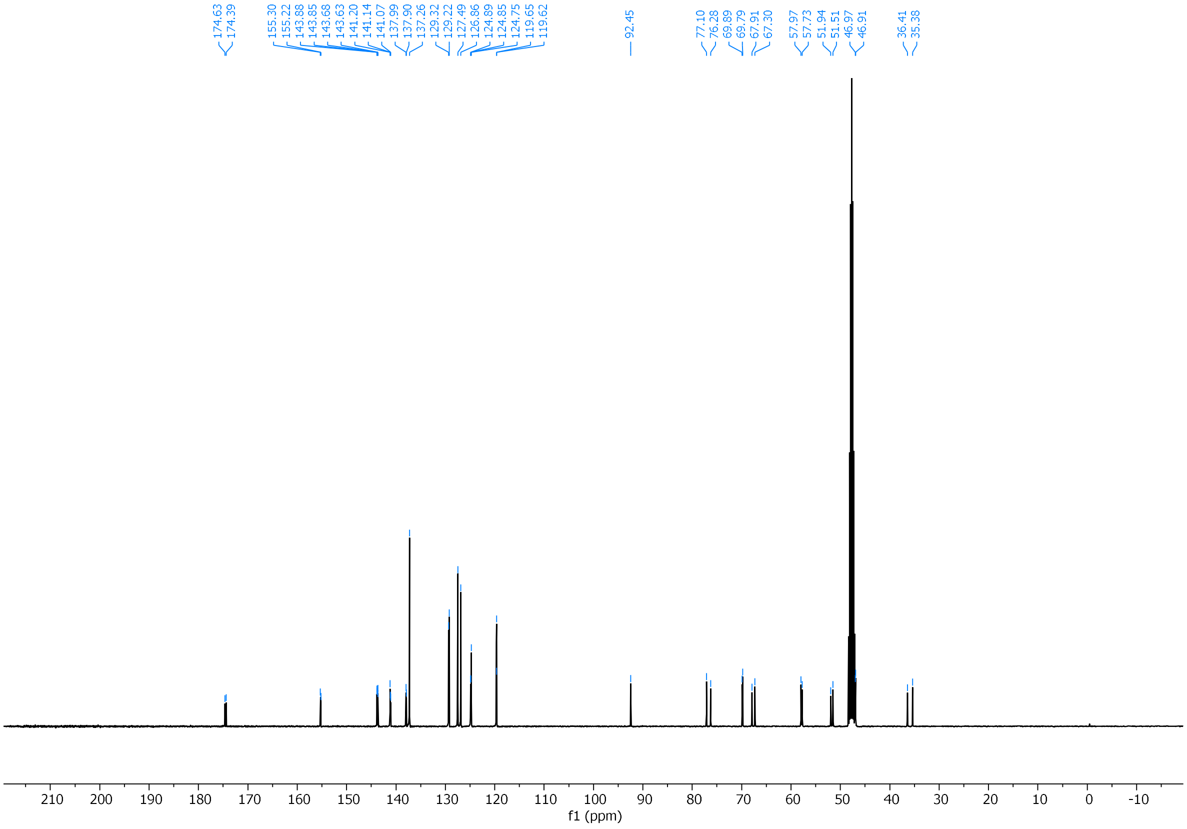

(2*R*,4*R*)-1-(((9H-fluoren-9-yl)methoxy)carbonyl)-4-((4-iodobenzyl)oxy)pyrrolidine-2-carboxylic acid (**14ci**)

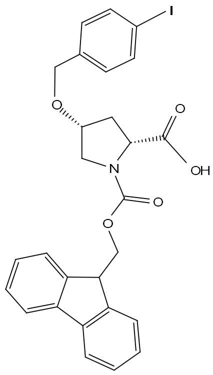

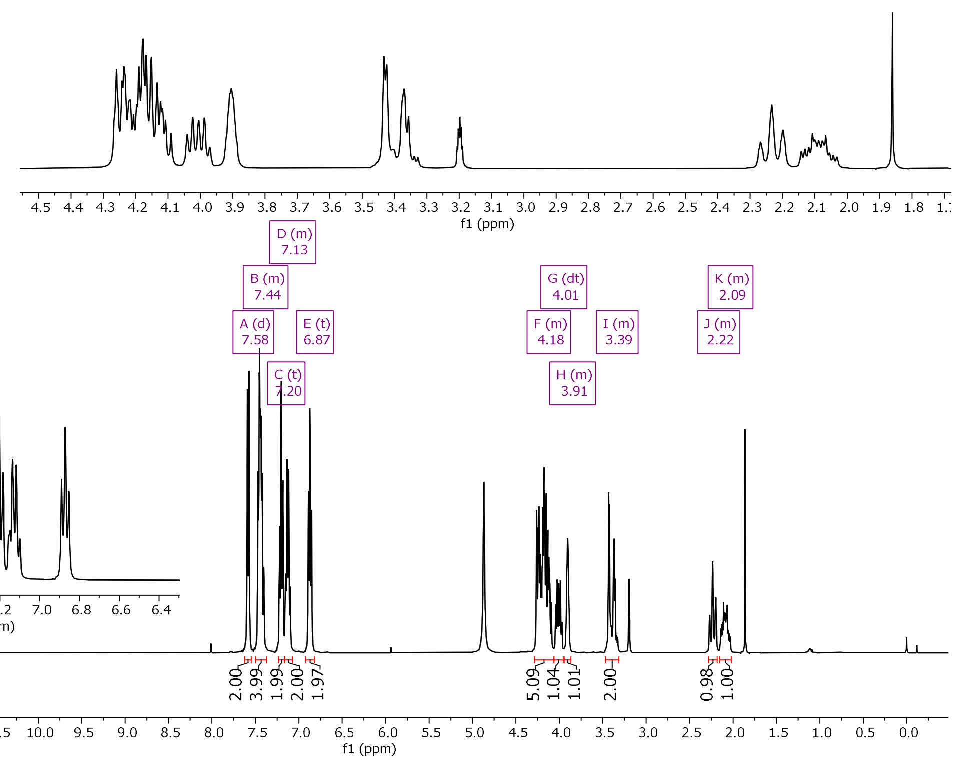

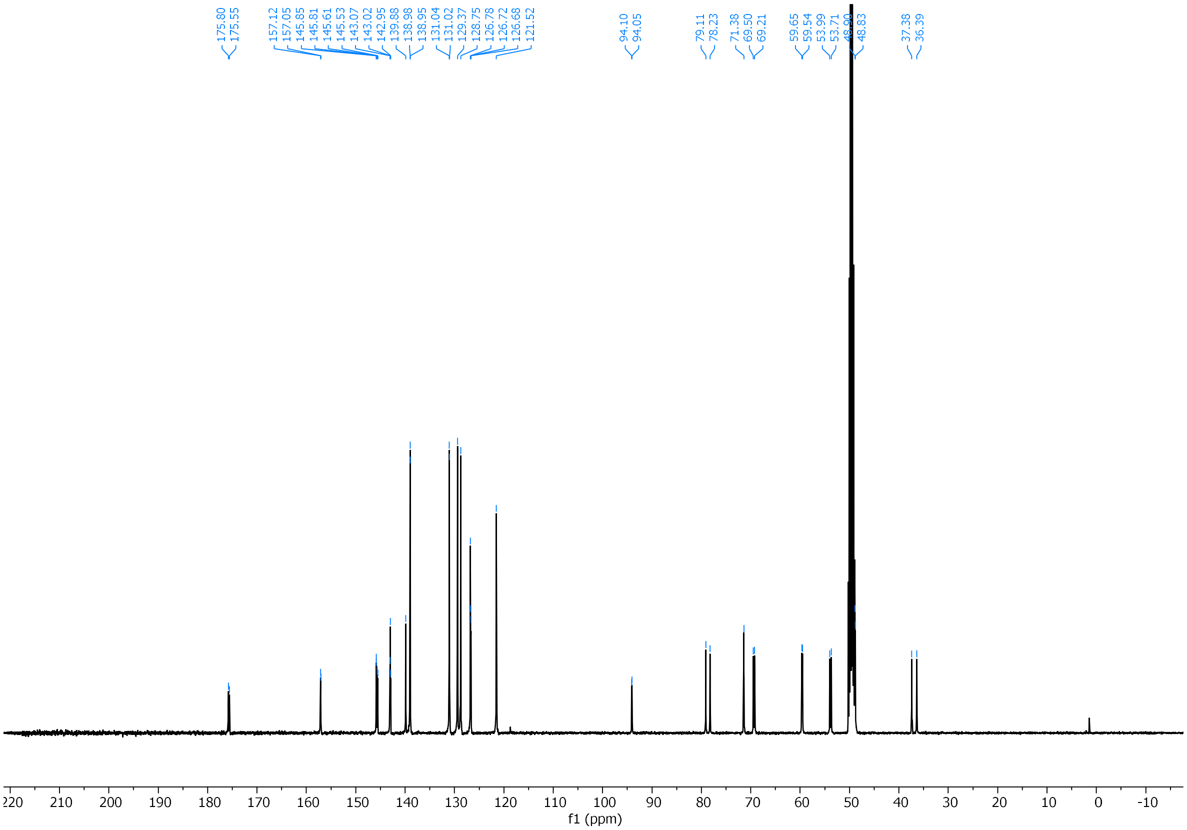

(2*R*,4*S*)-1-(((9H-fluoren-9-yl)methoxy)carbonyl)-4-((4-iodobenzyl)oxy)pyrrolidine-2-carboxylic acid (**14di**)

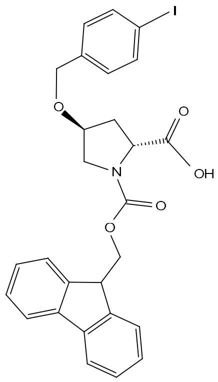

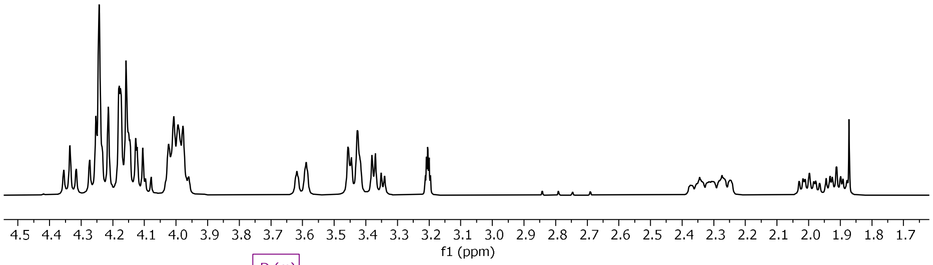

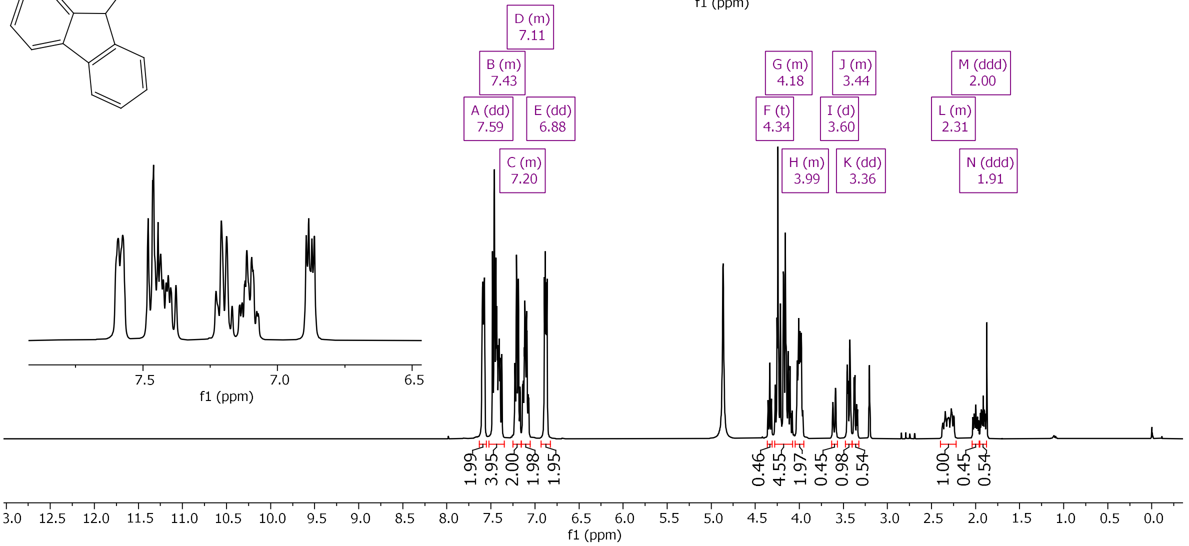

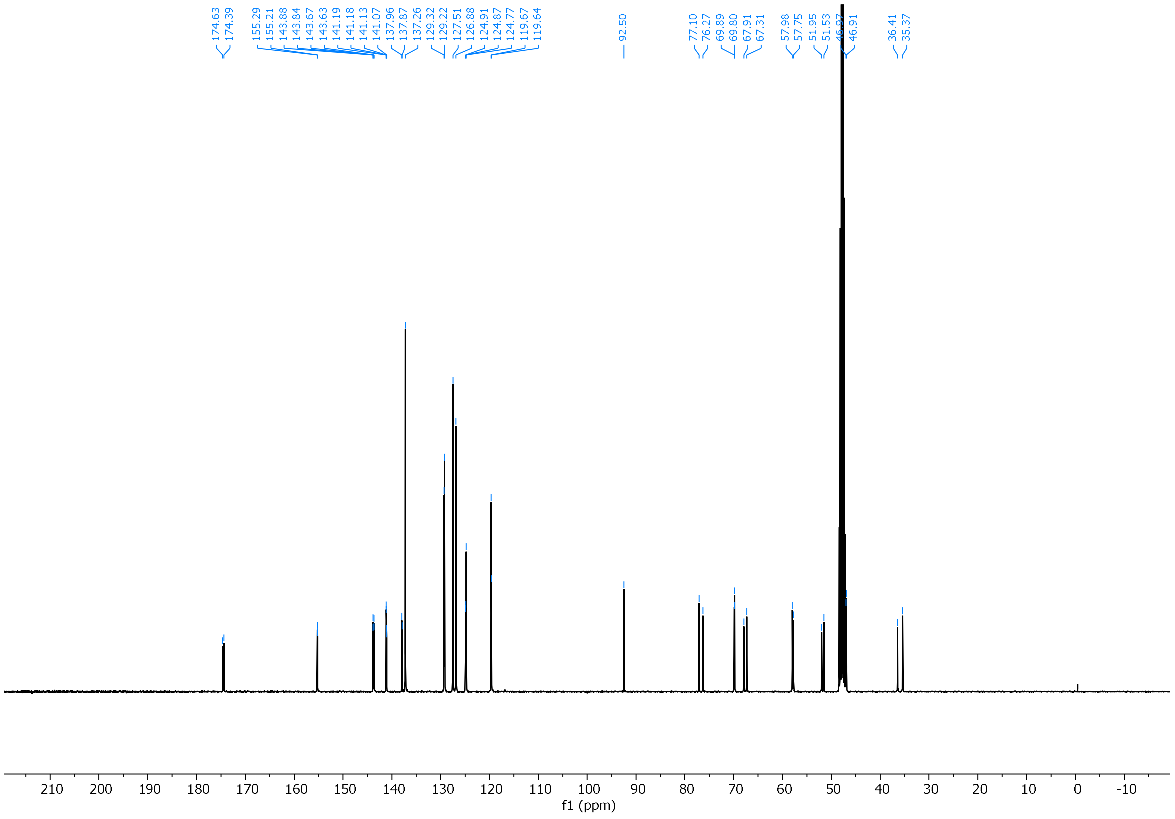

(2*S*,4*S*)-1-(((9H-fluoren-9-yl)methoxy)carbonyl)-4-((3-iodobenzyl)oxy)pyrrolidine-2-carboxylic acid (**14aii**)

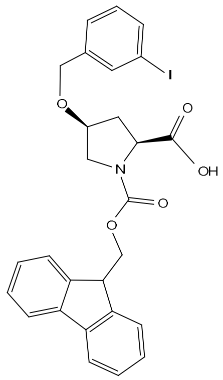

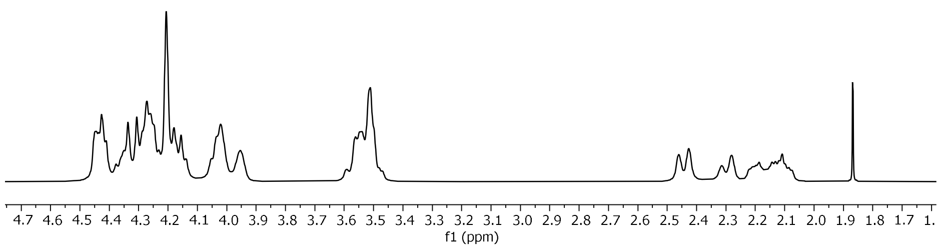

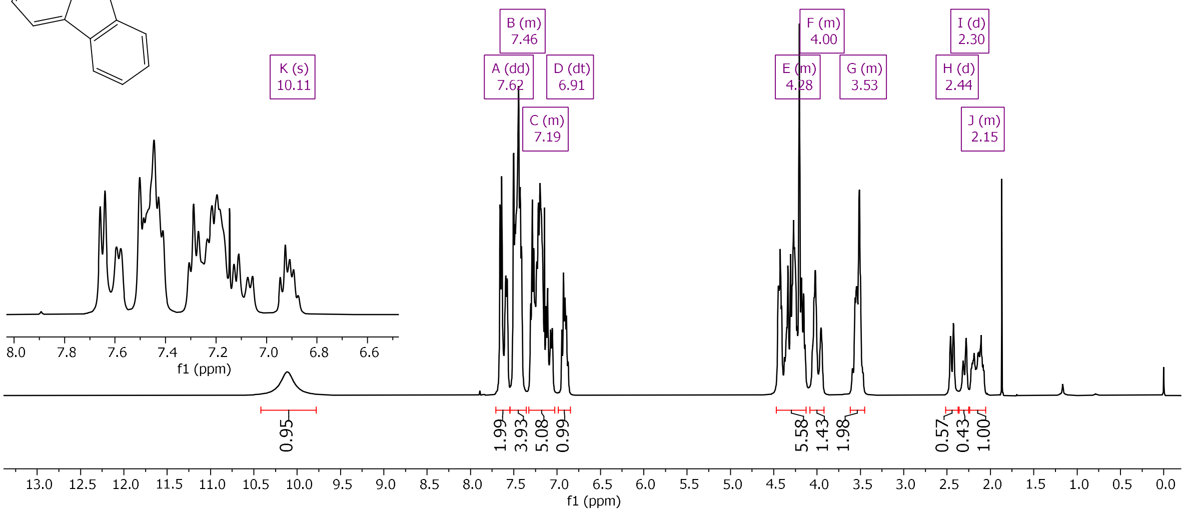

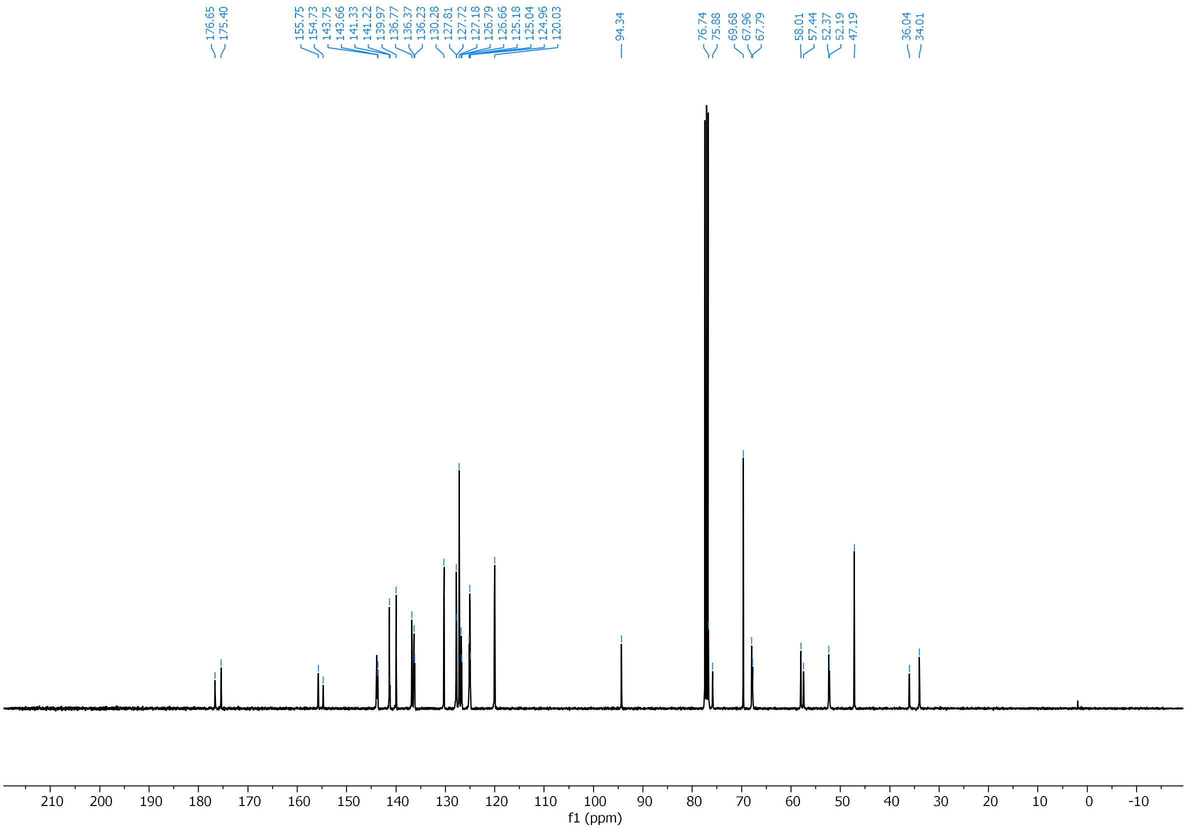

(2*S*,4*R*)-1-(((9H-fluoren-9-yl)methoxy)carbonyl)-4-((3-iodobenzyl)oxy)pyrrolidine-2-carboxylic acid (**14bii**)

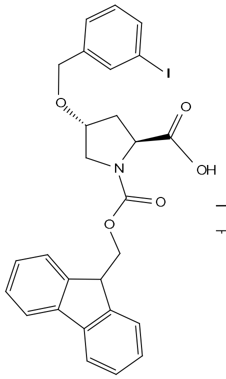

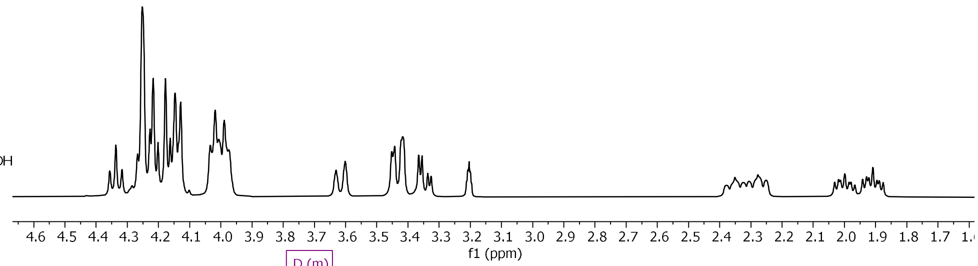

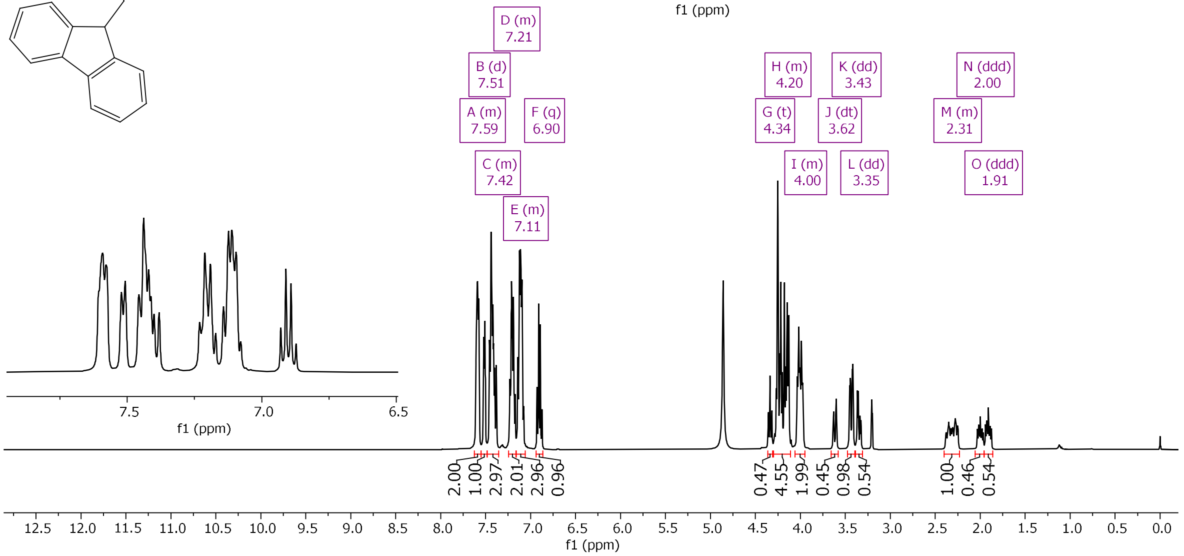

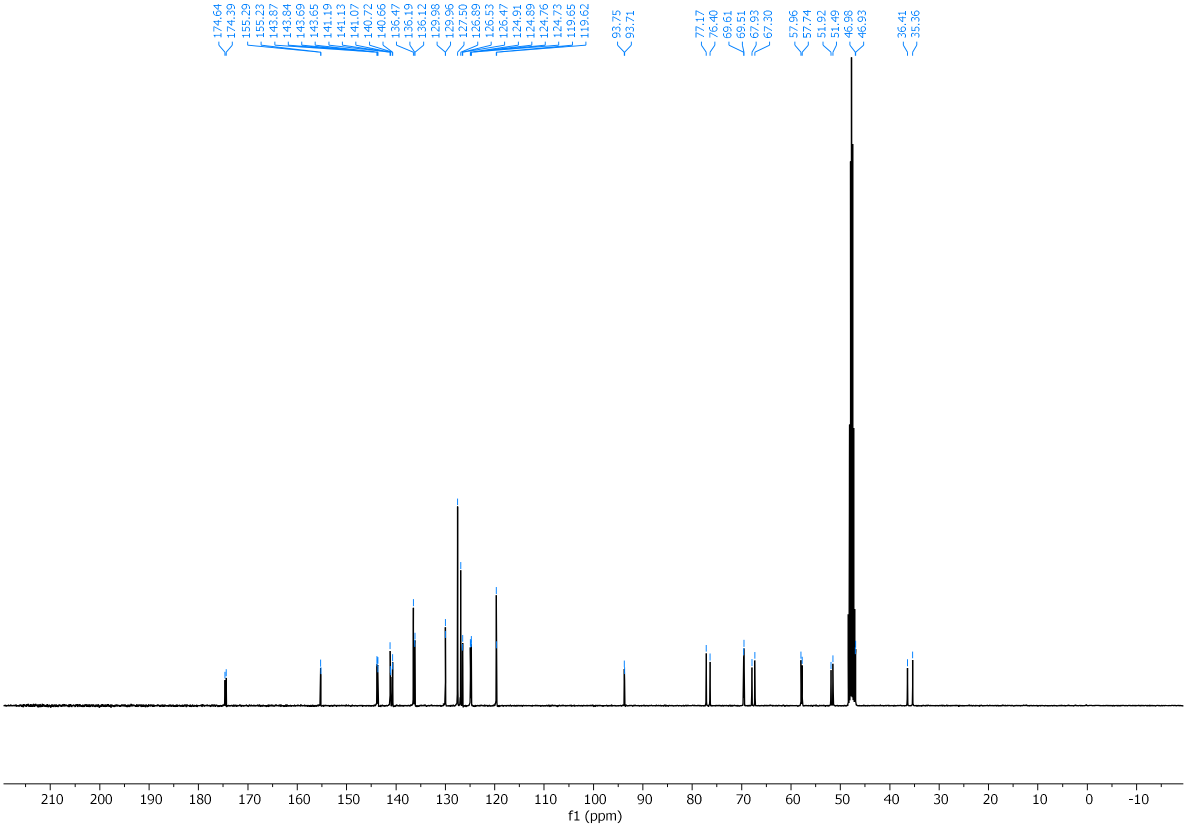

(2*R*,4*R*)-1-(((9H-fluoren-9-yl)methoxy)carbonyl)-4-((3-iodobenzyl)oxy)pyrrolidine-2-carboxylic acid (**14cii**)

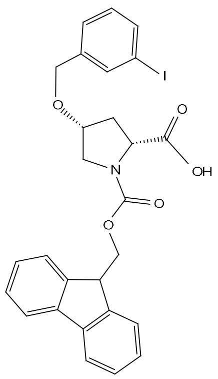

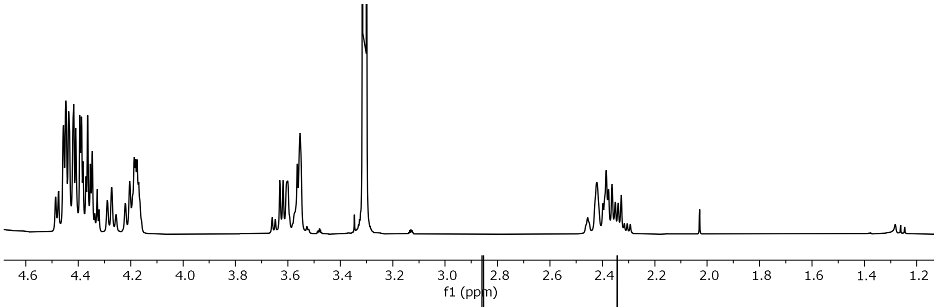

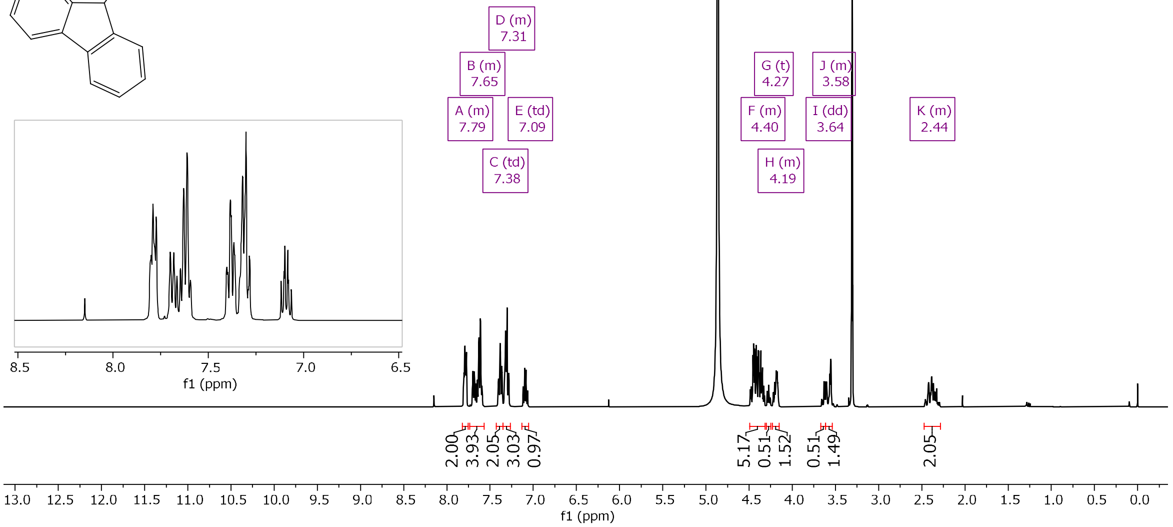

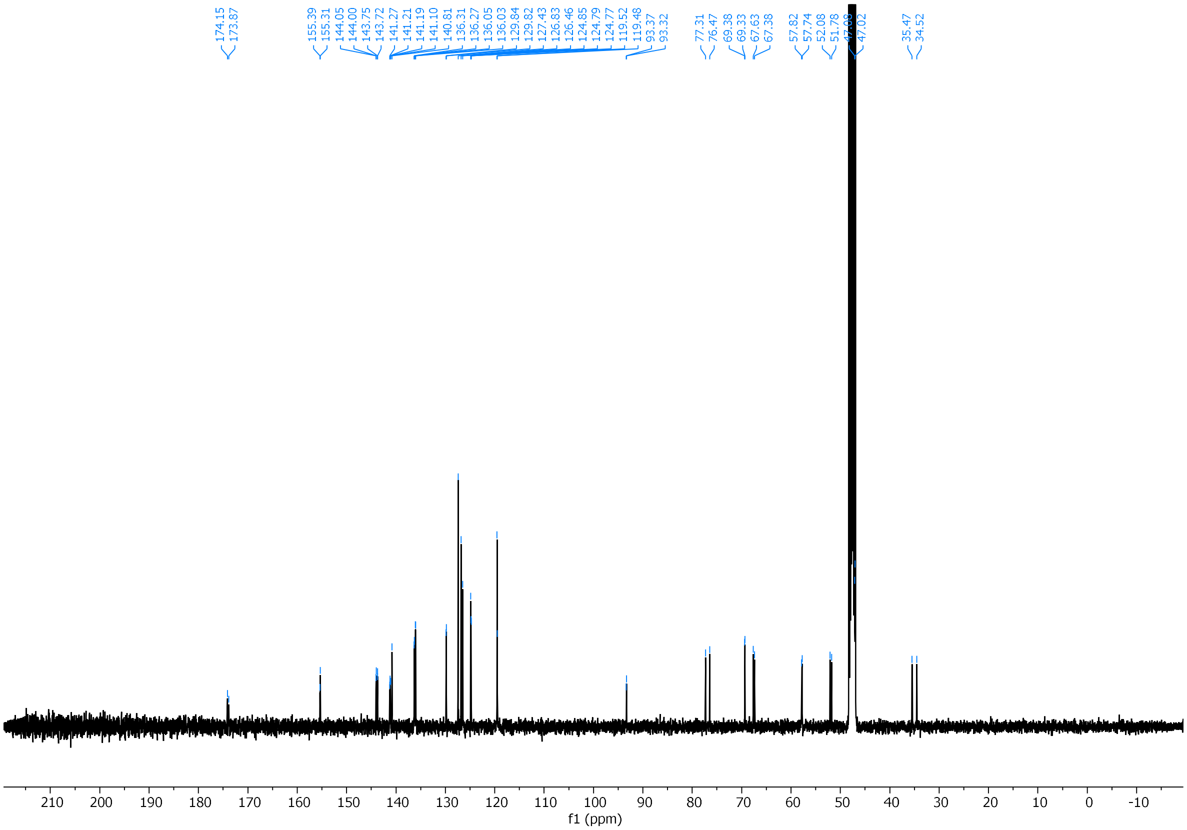

(2*R*,4*S*)-1-(((9H-fluoren-9-yl)methoxy)carbonyl)-4-((3-iodobenzyl)oxy)pyrrolidine-2-carboxylic acid (**14dii**)

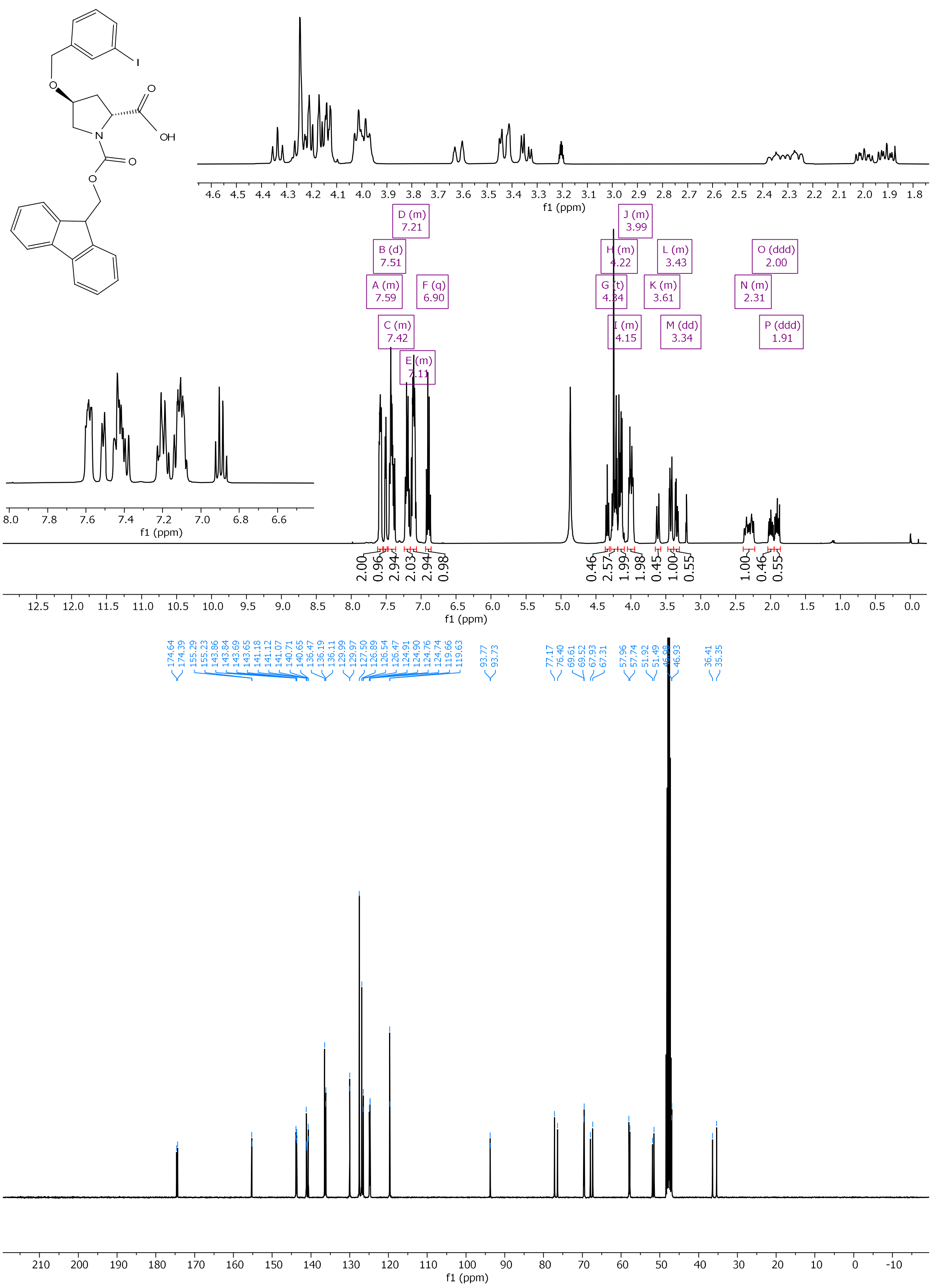

7.3. Spiro-azetdine skeletons

(*R*)-4-benzyl-3-((3*S*,4*R*)-1-benzyl-4-(4-iodophenyl)pyrrolidine-3-carbonyl)oxazolidin-2-one (**15**)

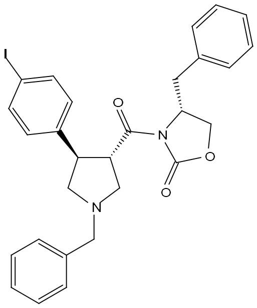

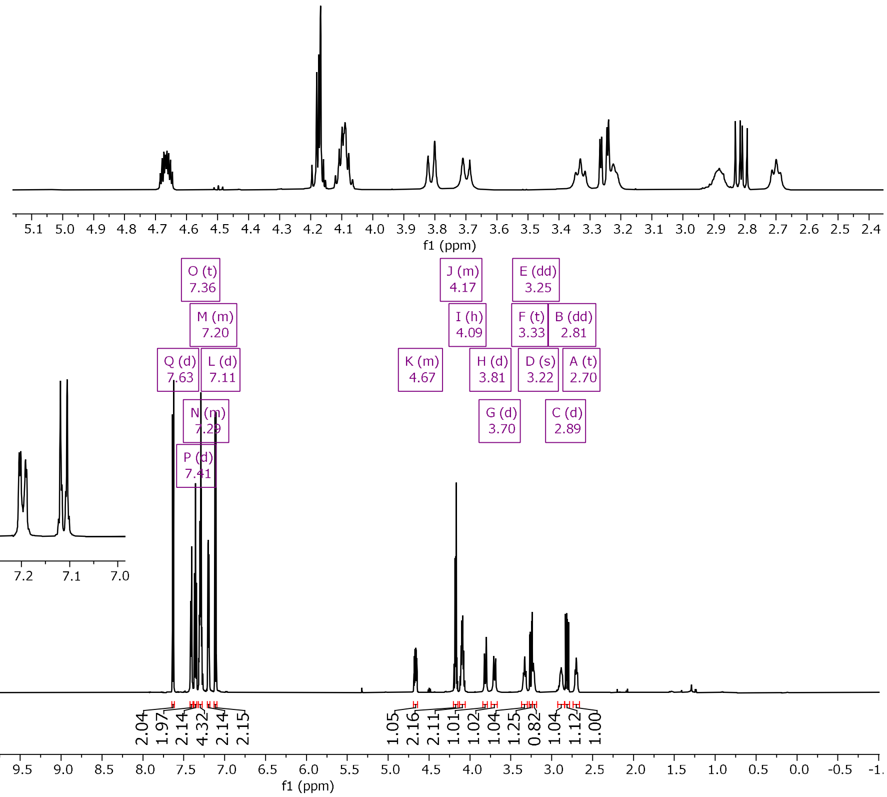

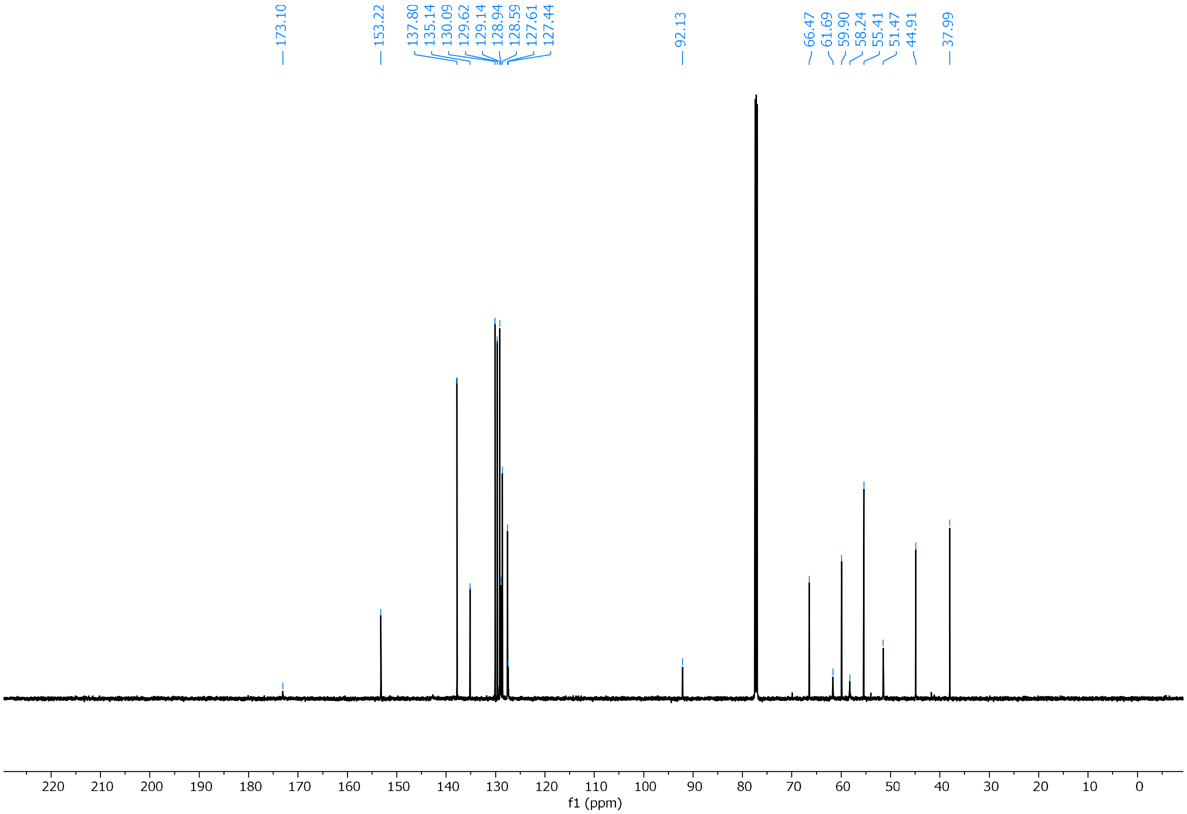

(*R*)-4-benzyl-3-((3*R*,4*S*)-1-benzyl-4-(4-iodophenyl)pyrrolidine-3-carbonyl)oxazolidin-2-one (**16**)

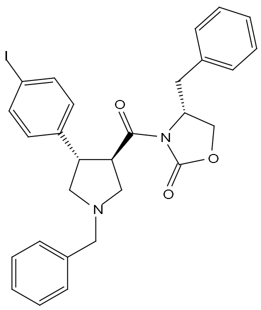

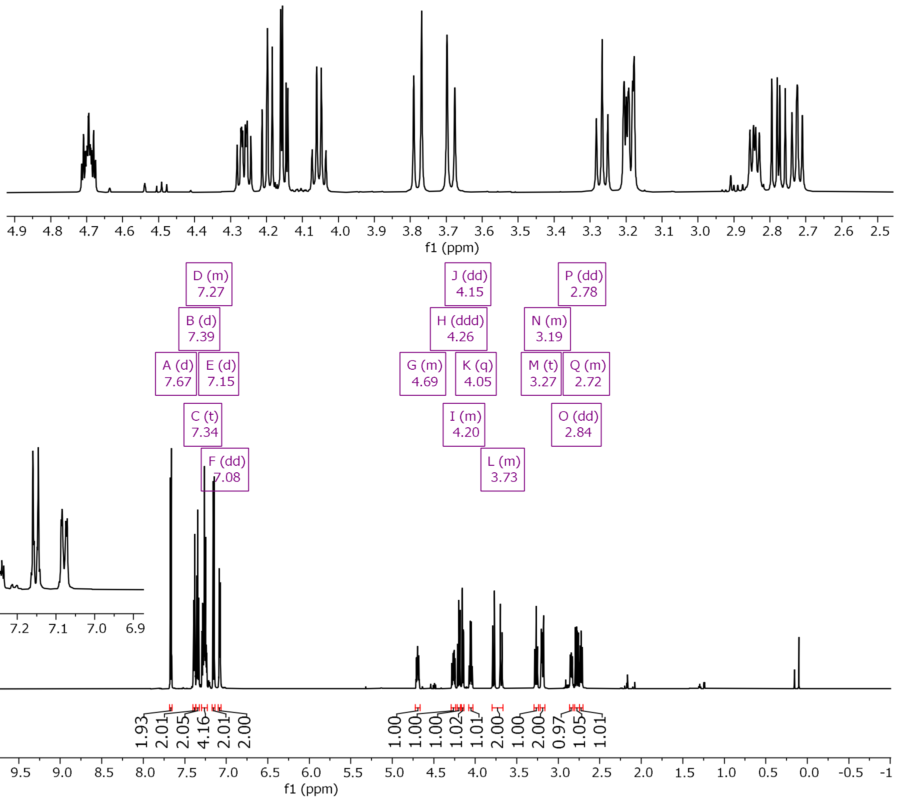

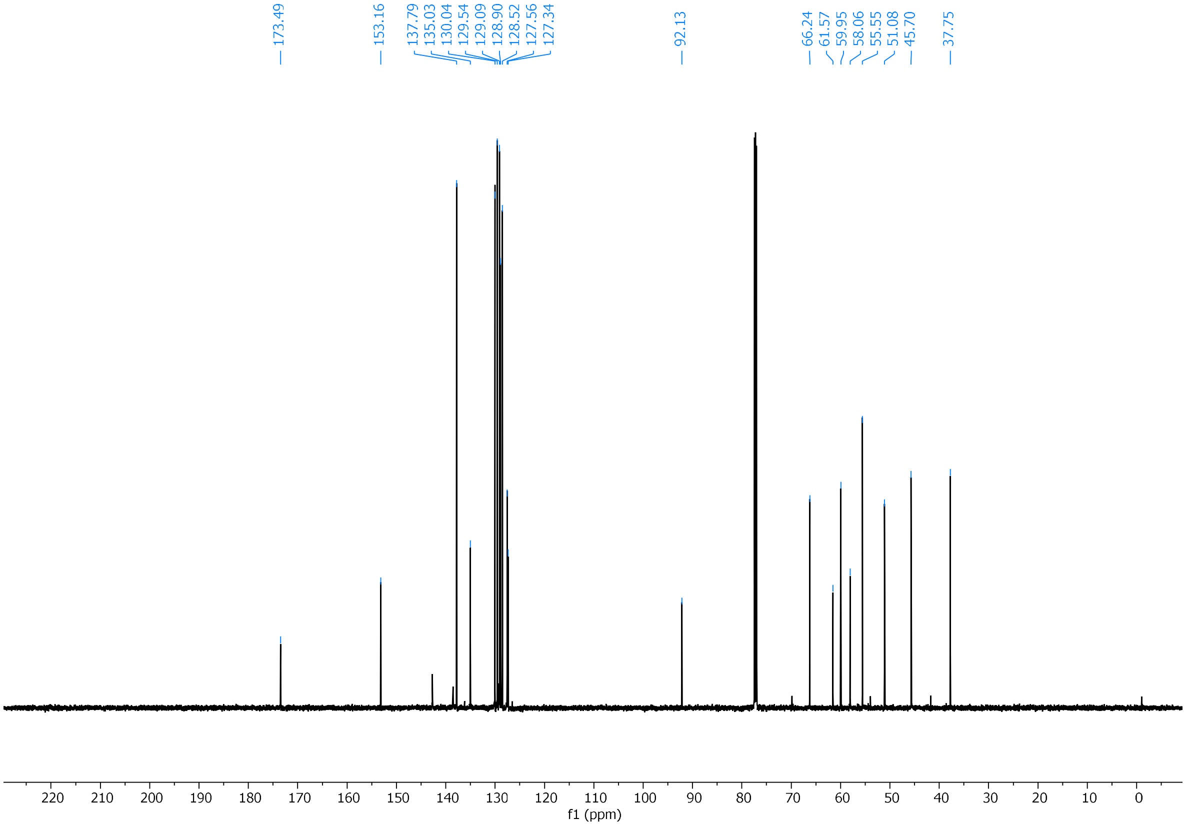

1-(*tert*-butyl) 3-methyl (3*R*,4*S*)-4-(4-iodophenyl)pyrrolidine-1,3-dicarboxylate (**17**)

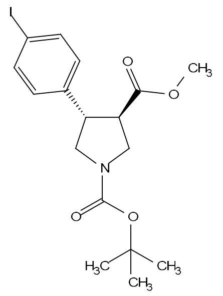

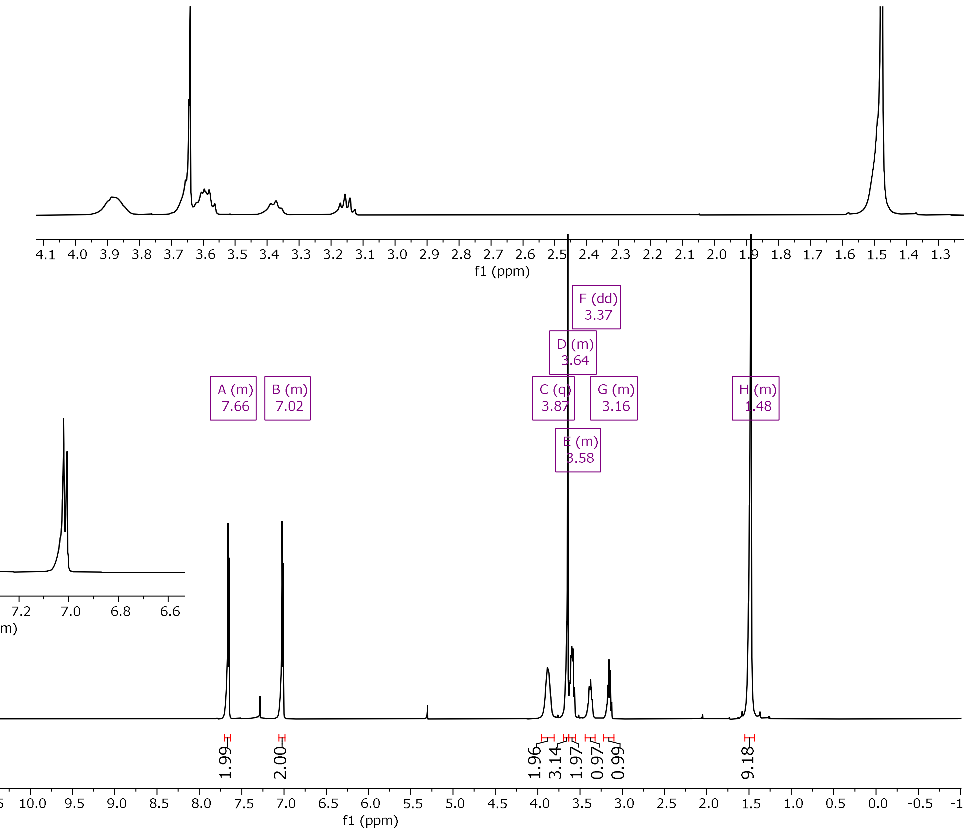

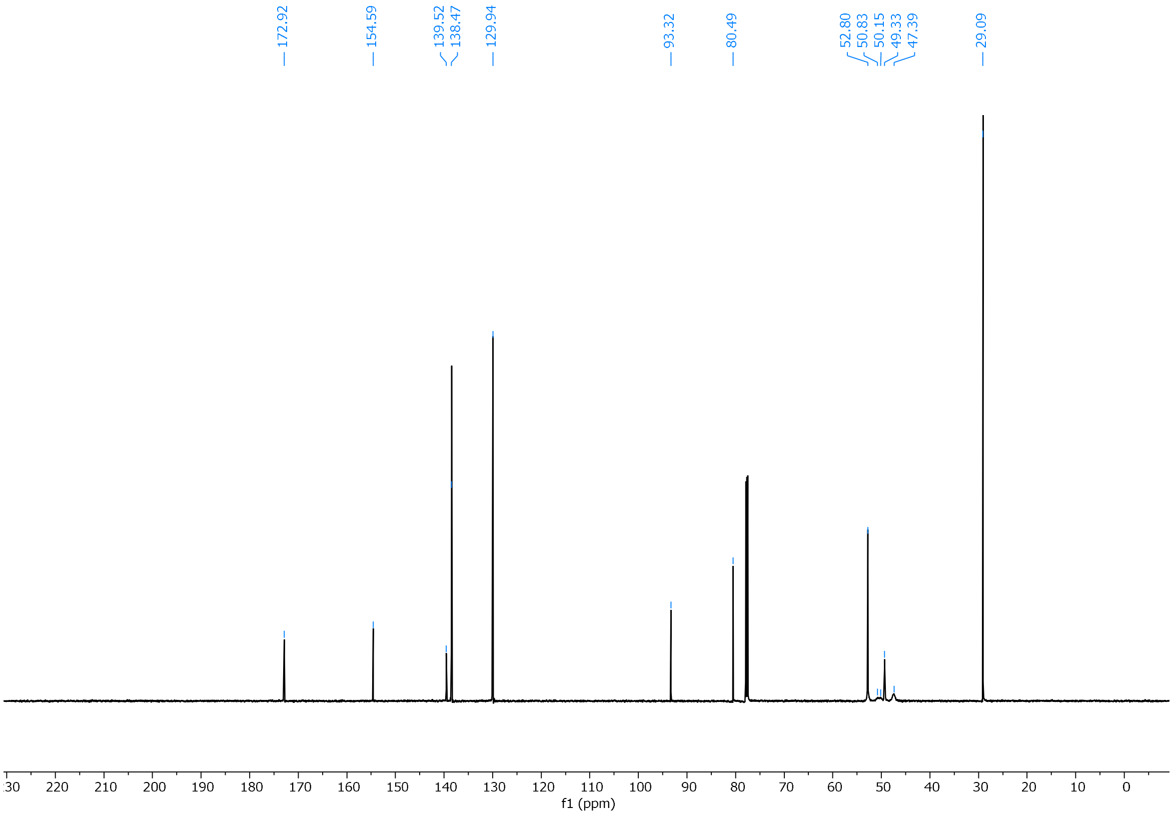

1-(*tert*-butyl) 3-methyl (3*S*,4*R*)-4-(4-iodophenyl)pyrrolidine-1,3-dicarboxylate (**18**)

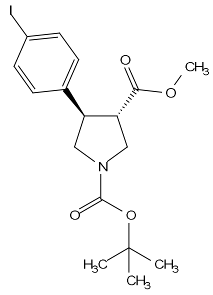

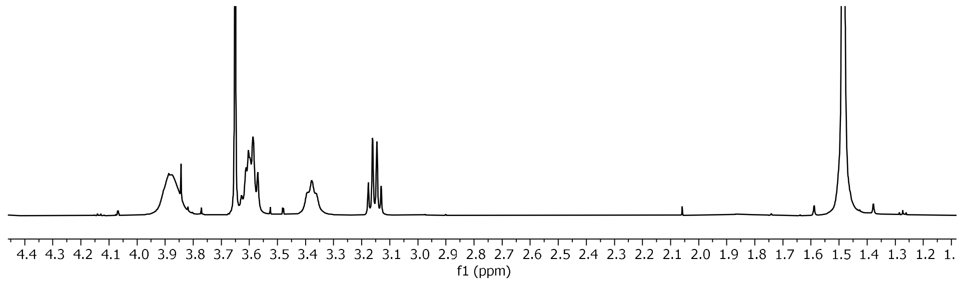

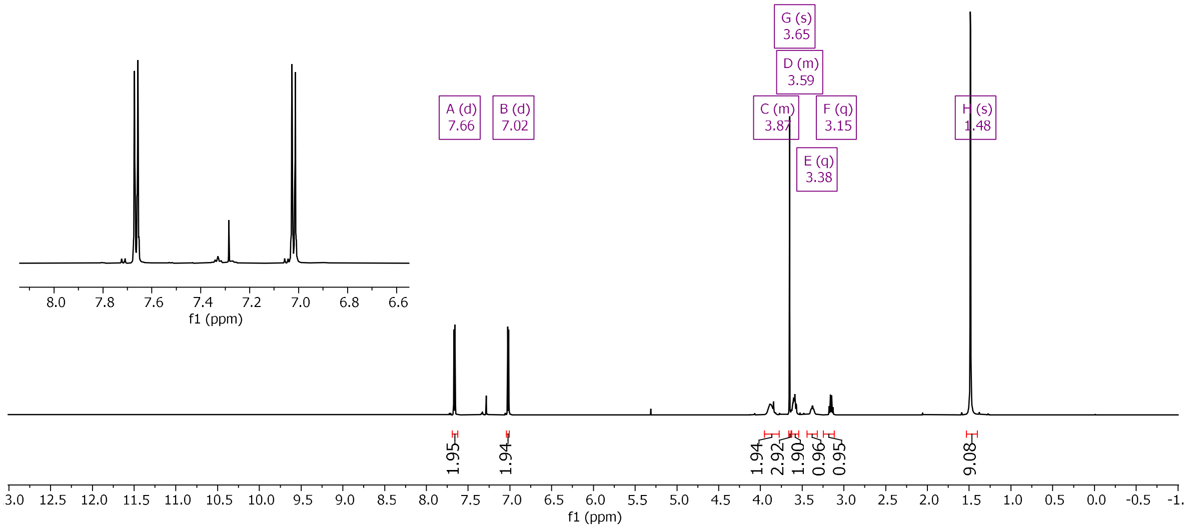

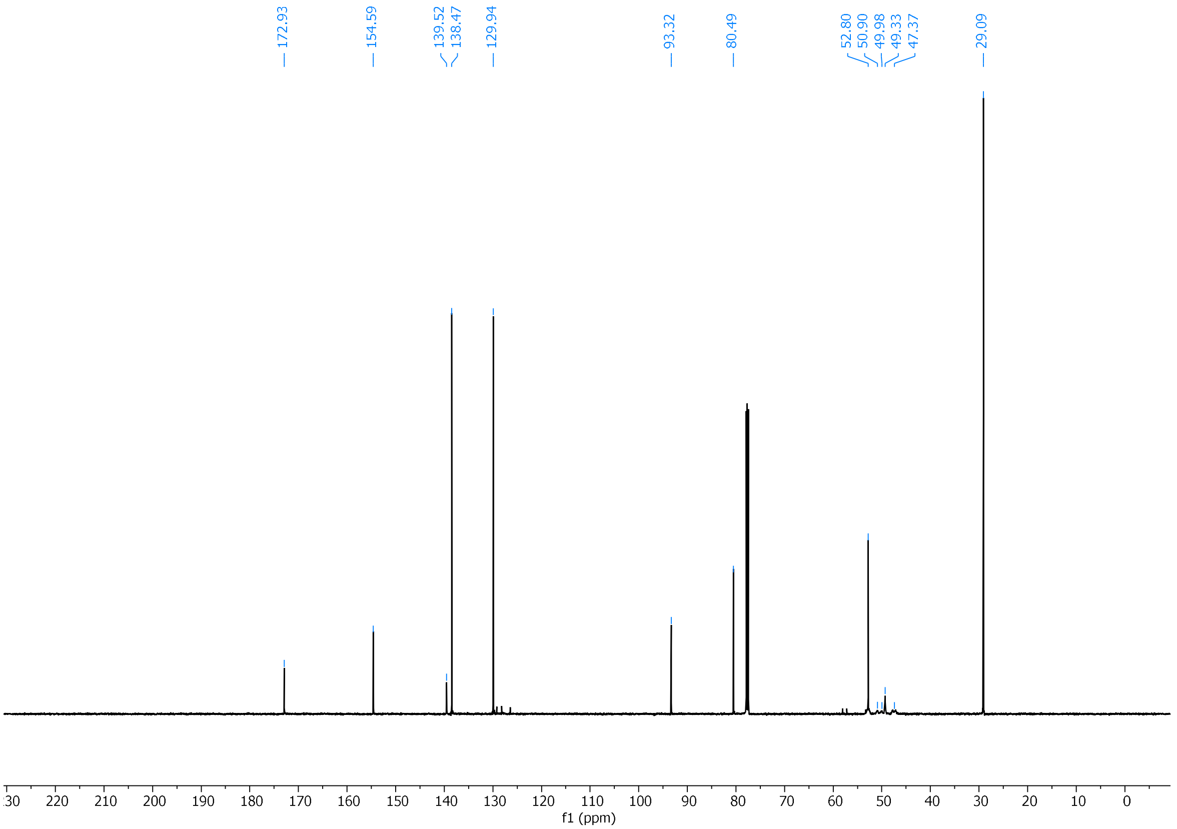

tert-butyl (*R*)-8-(4-iodophenyl)-2-((4-nitrophenyl)sulfonyl)-2,6-diazaspiro[3.4]octane-6-carboxylate (**19**)

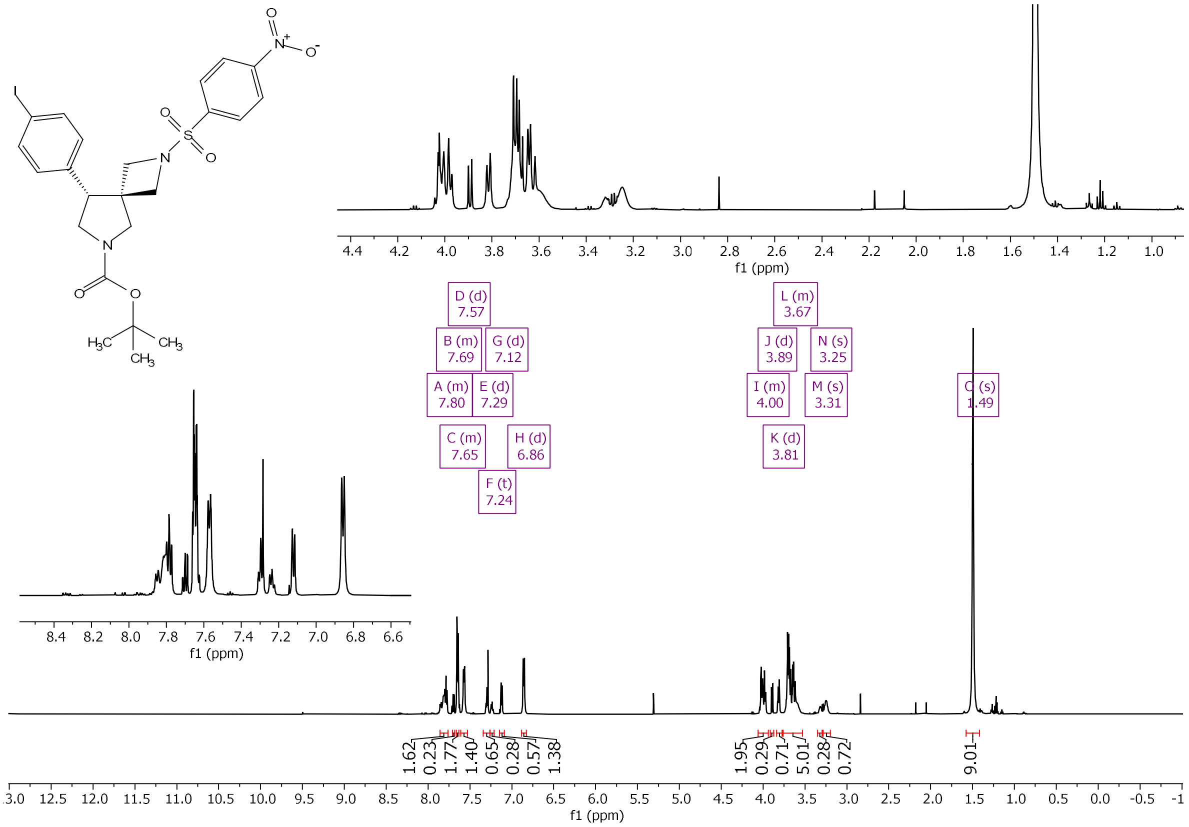

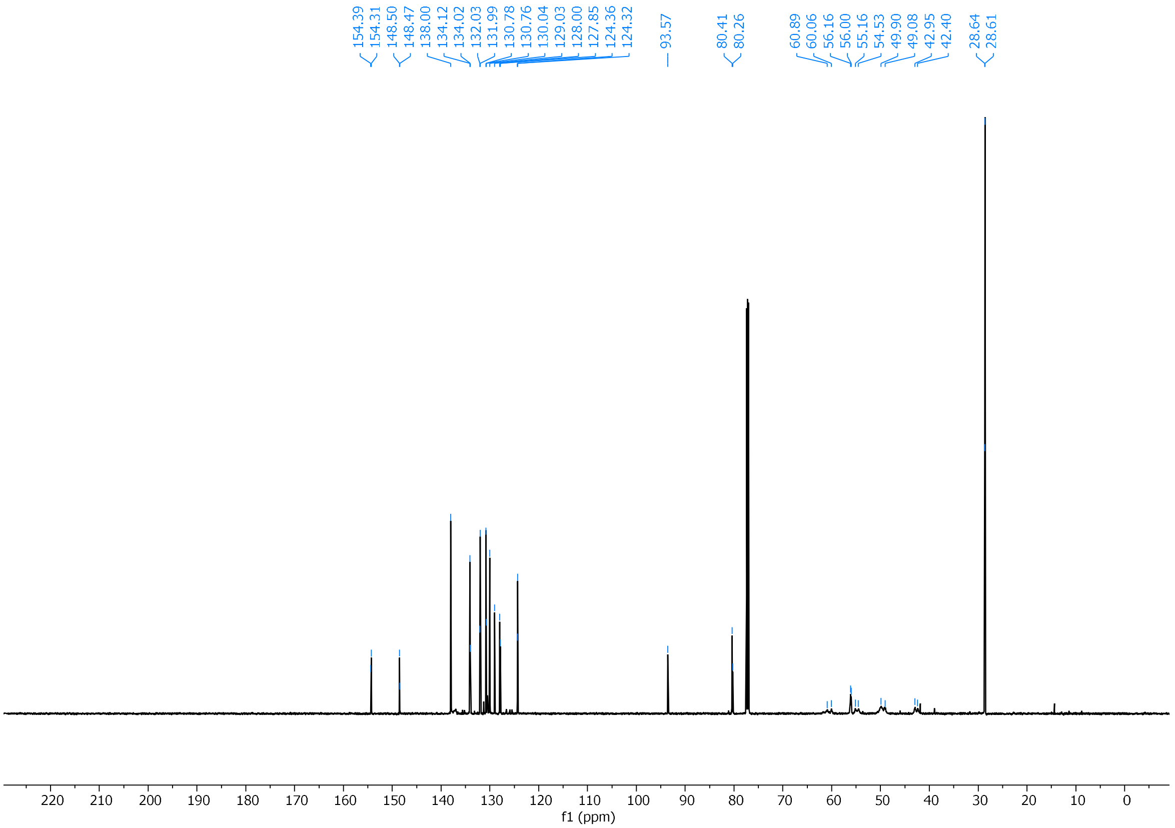

tert-butyl (*S*)-8-(4-iodophenyl)-2-((4-nitrophenyl)sulfonyl)-2,6-diazaspiro[3.4]octane-6-carboxylate (**20**)

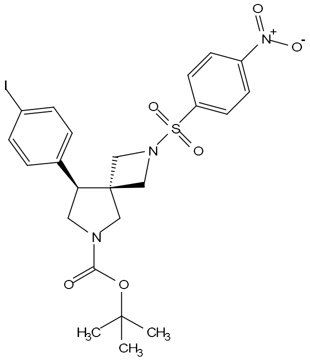

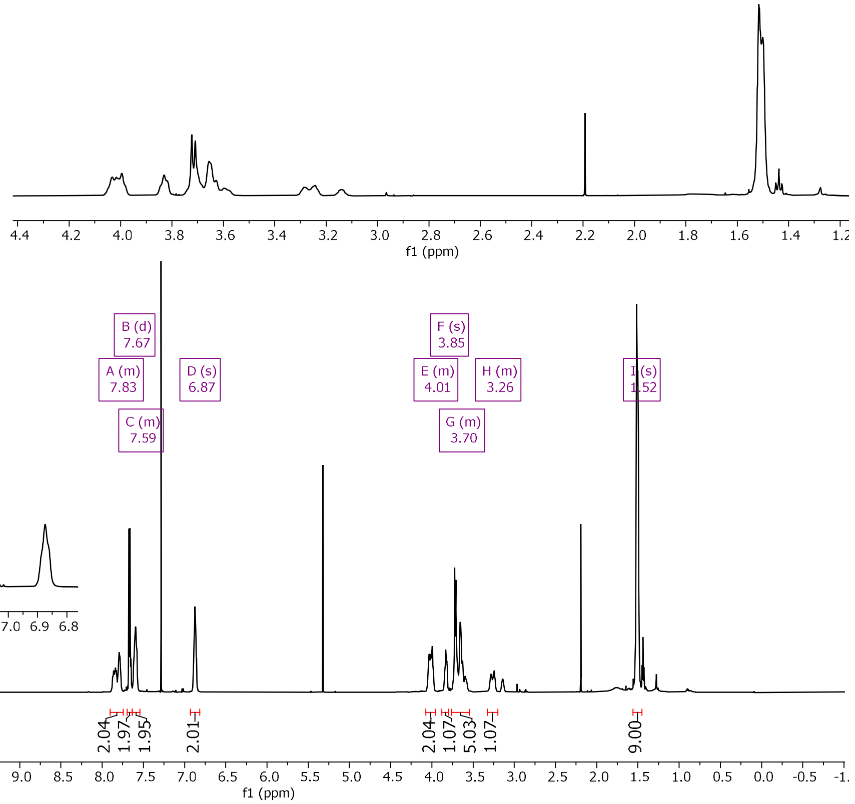

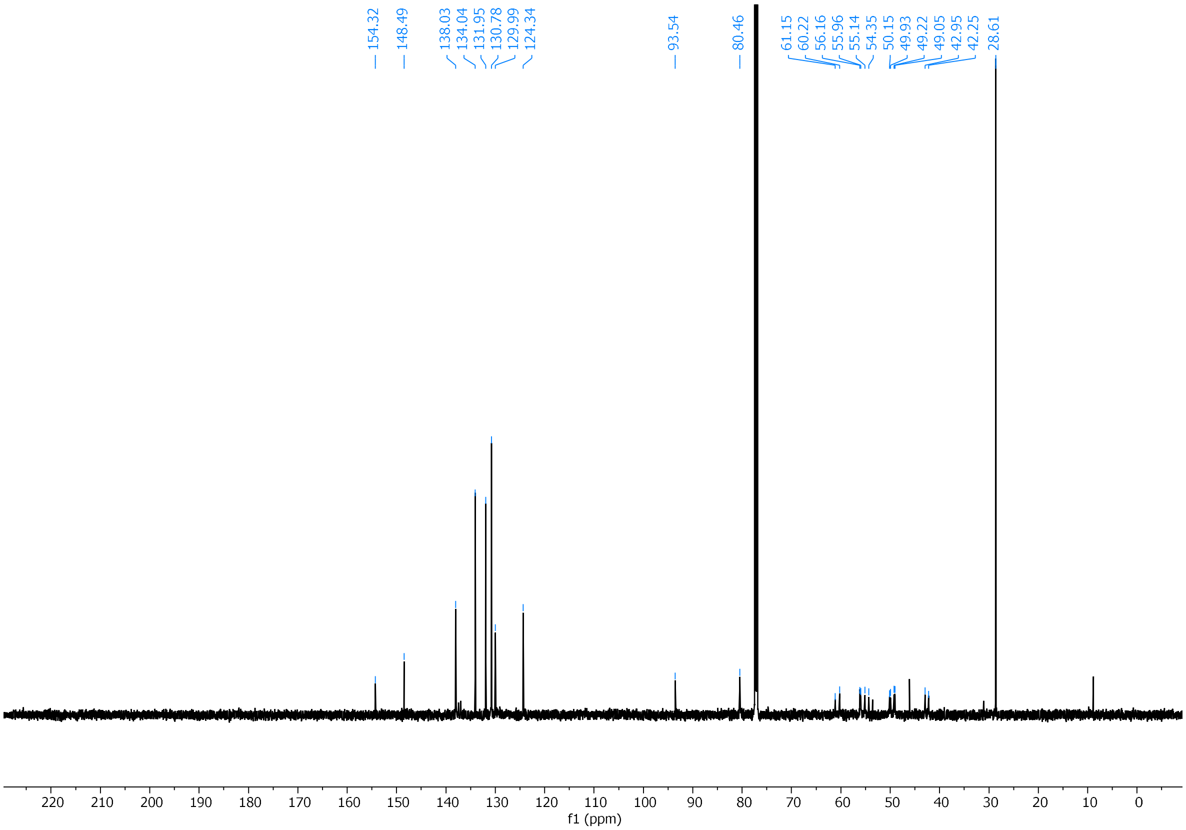

(*R*)-5-(8-(4-iodophenyl)-2-((4-nitrophenyl)sulfonyl)-2,6-diazaspiro[3.4]octan-6-yl)-5-oxopentanoic acid (**21**)

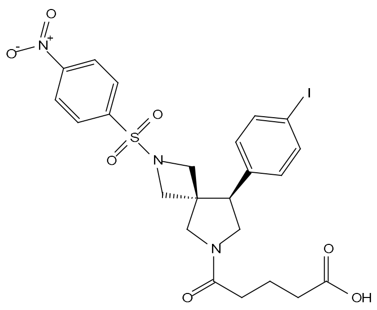

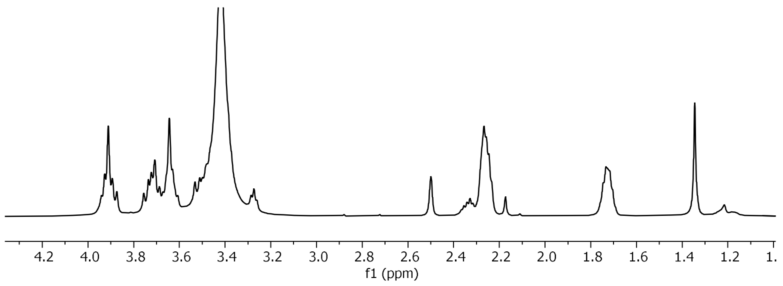

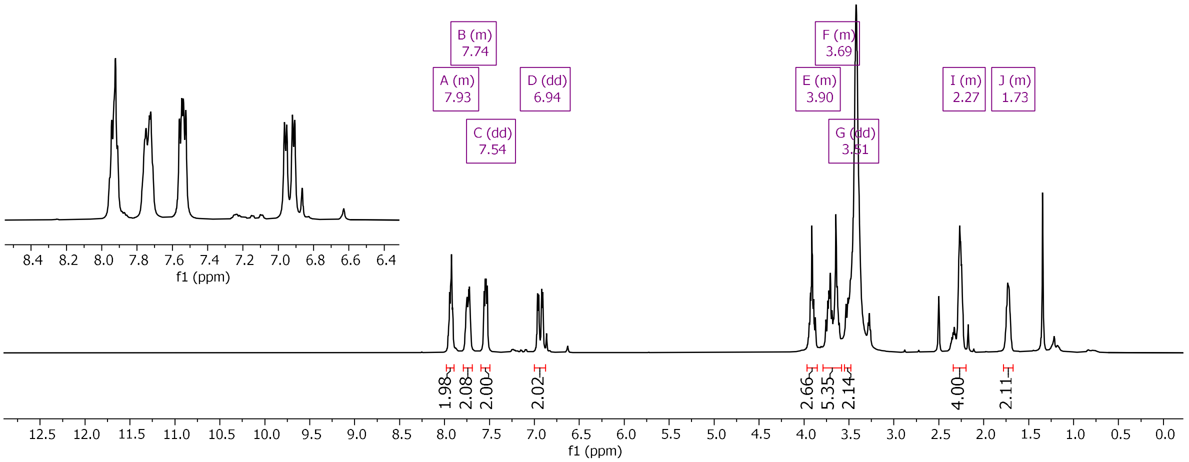

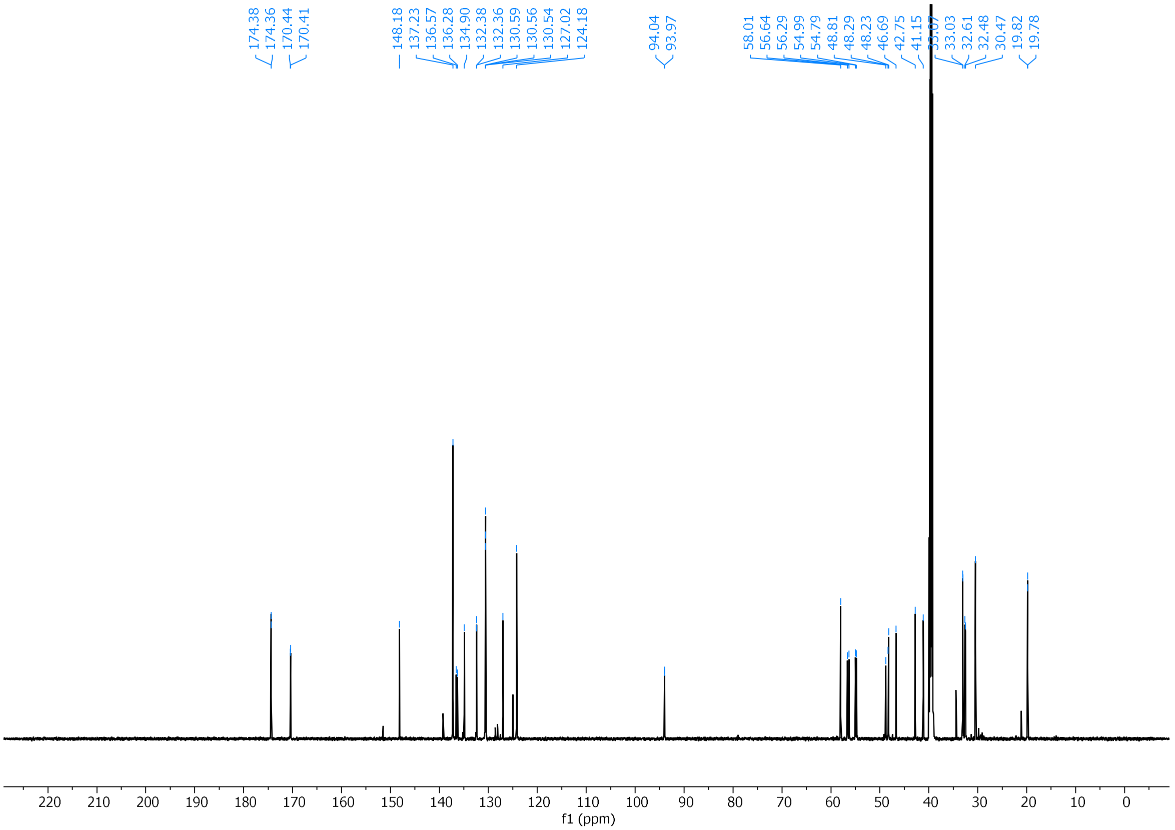

(*S*)-5-(8-(4-iodophenyl)-2-((4-nitrophenyl)sulfonyl)-2,6-diazaspiro[3.4]octan-6-yl)-5-oxopentanoic acid (**22**)

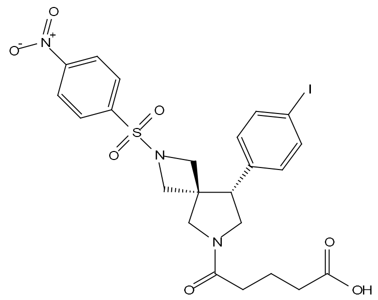

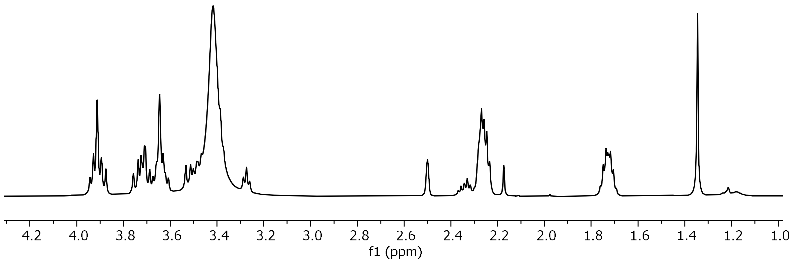

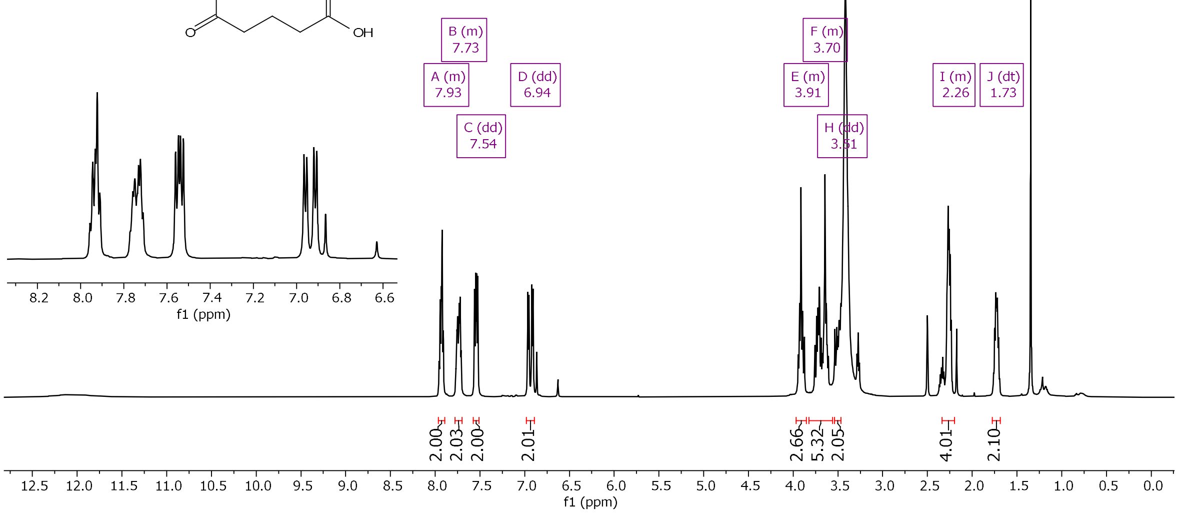

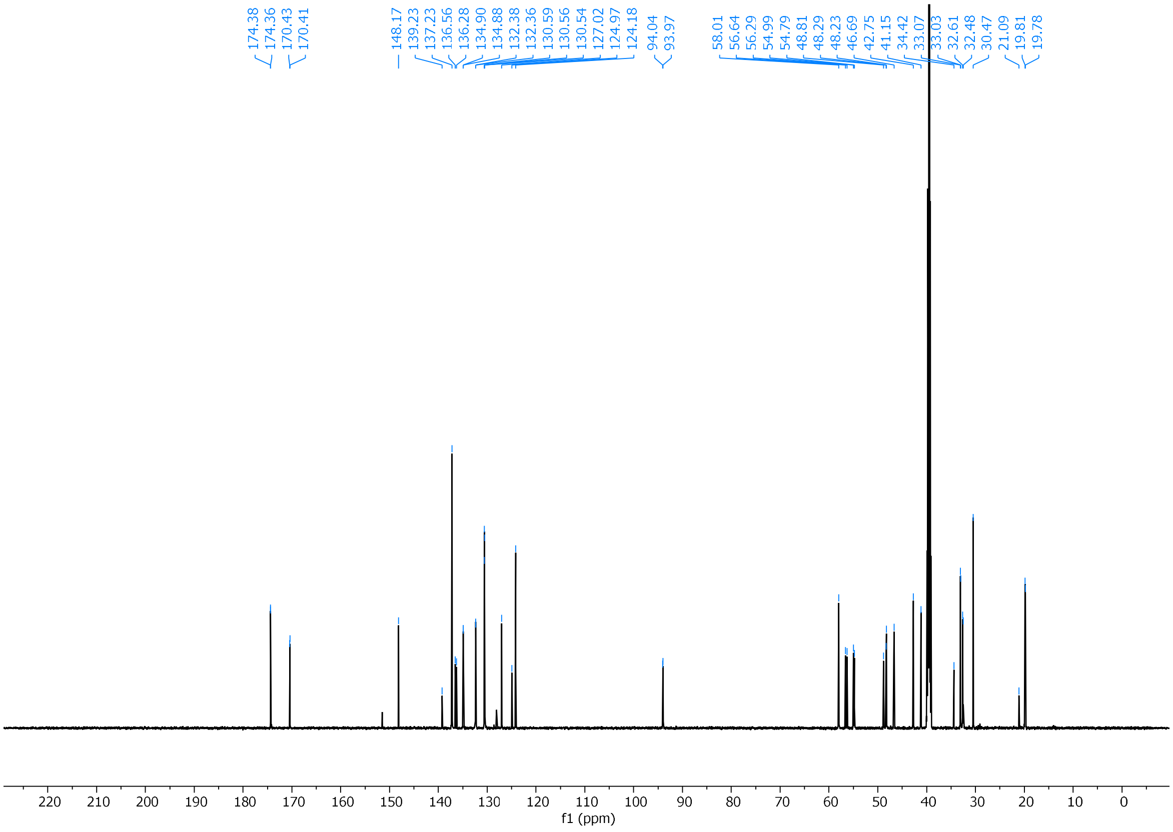

(*R*)-5-(6-(((9H-fluoren-9-yl)methoxy)carbonyl)-8-(4-iodophenyl)-2,6-diazaspiro[3.4]octan-2-yl)-5-oxopentanoic acid (**23**)

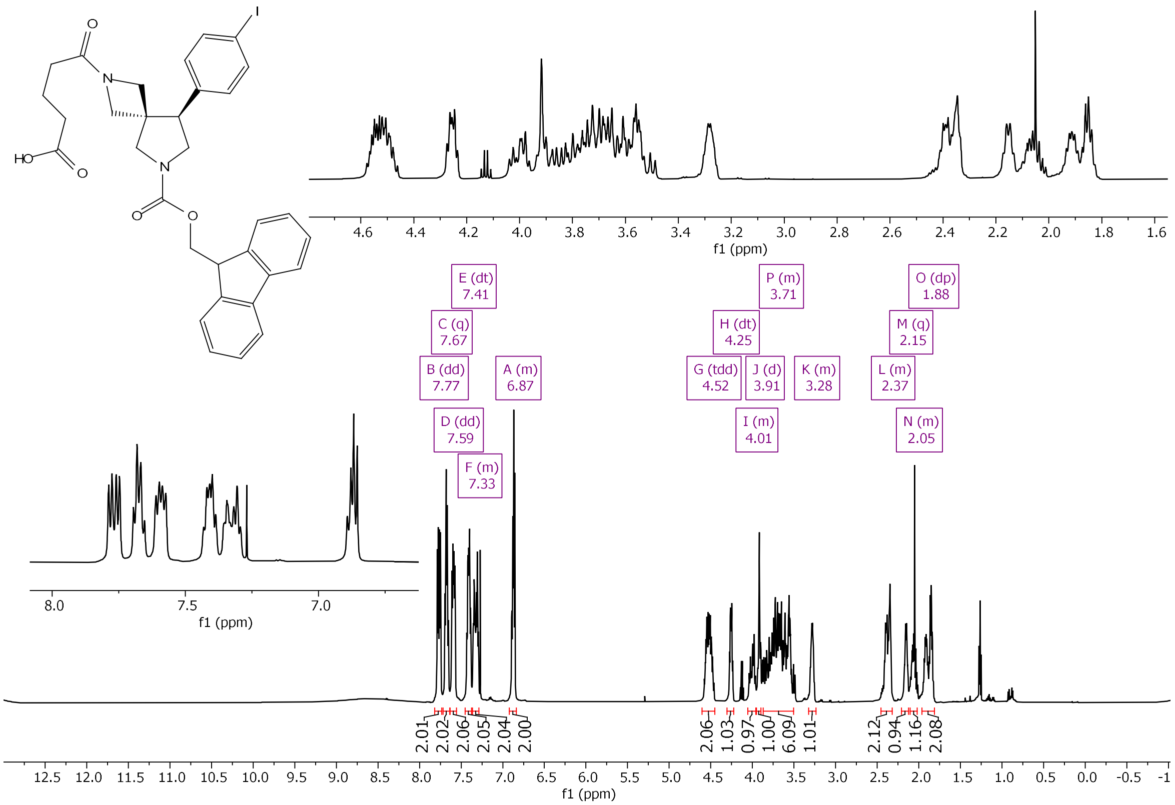

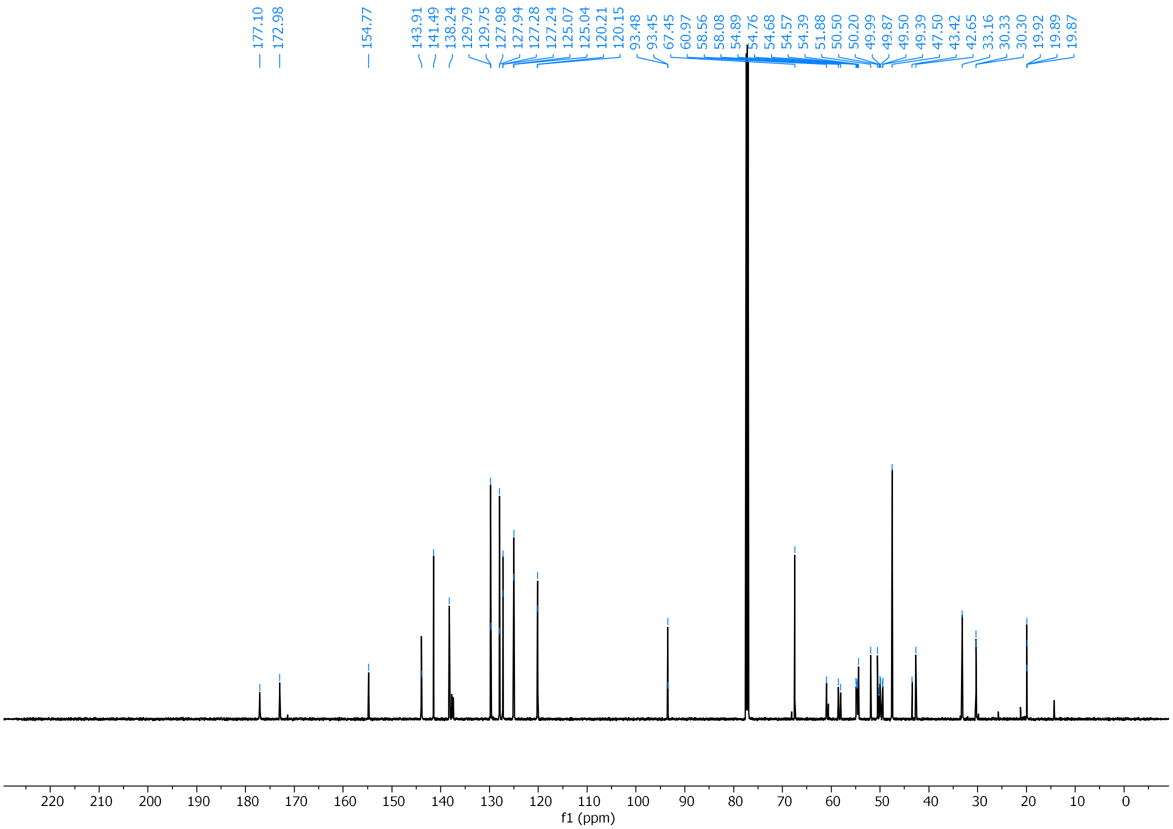
177.10
172.98
154.77
143.91
141.49
138.24
129.79
129.75
127.98
127.94
127.28
127.24
125.07
125.04
120.21
120.15
93.48
93.45
67.45
60.97
58.56
58.08
54.89
54.76
54.68
54.57
54.39
51.88
50.50
50.20
49.99
49.87
49.50
49.39
47.50
43.42
42.65
33.16
30.33
30.30
19.92
19.89
19.87
220 210 200 190 180 170 160 150 140 130 120 110 100 90 80 70 60 50 40 30 20 10 0
f1 (ppm)

(*S*)-5-(6-(((9H-fluoren-9-yl)methoxy)carbonyl)-8-(4-iodophenyl)-2,6-diazaspiro[3.4]octan-2-yl)-5-oxopentanoic acid (**24**)

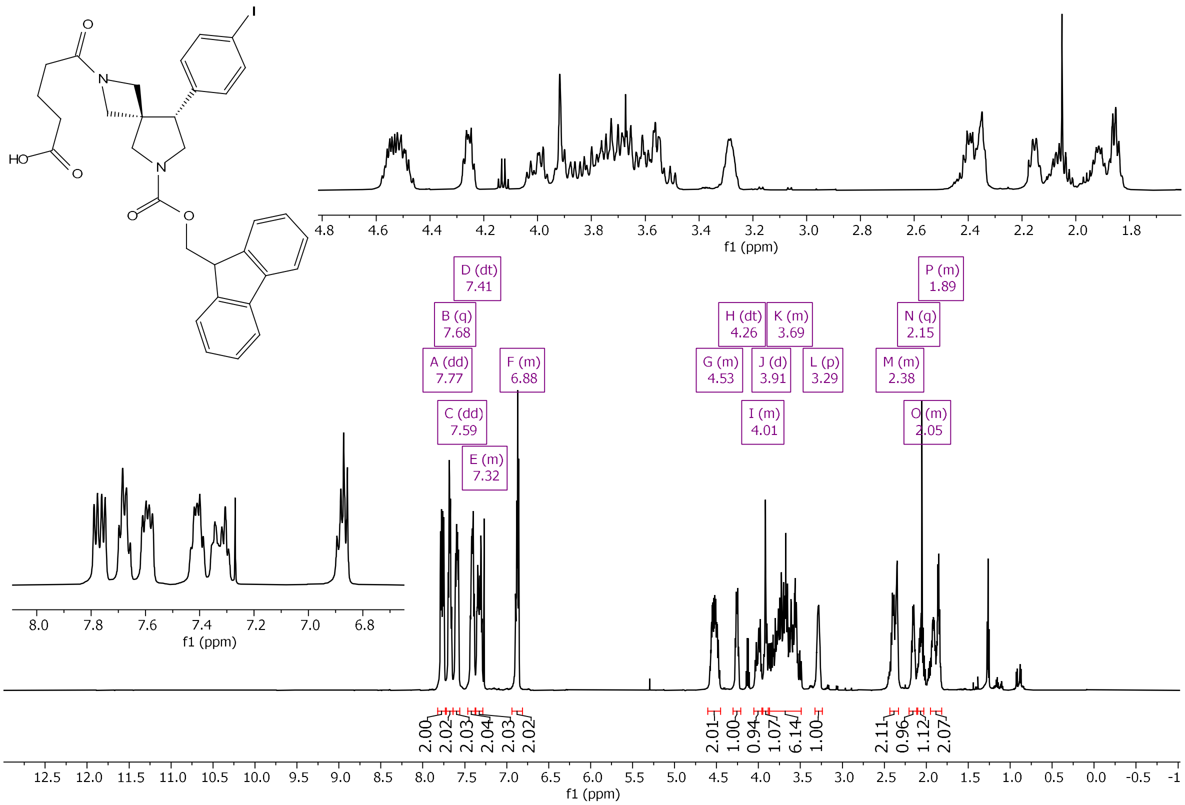

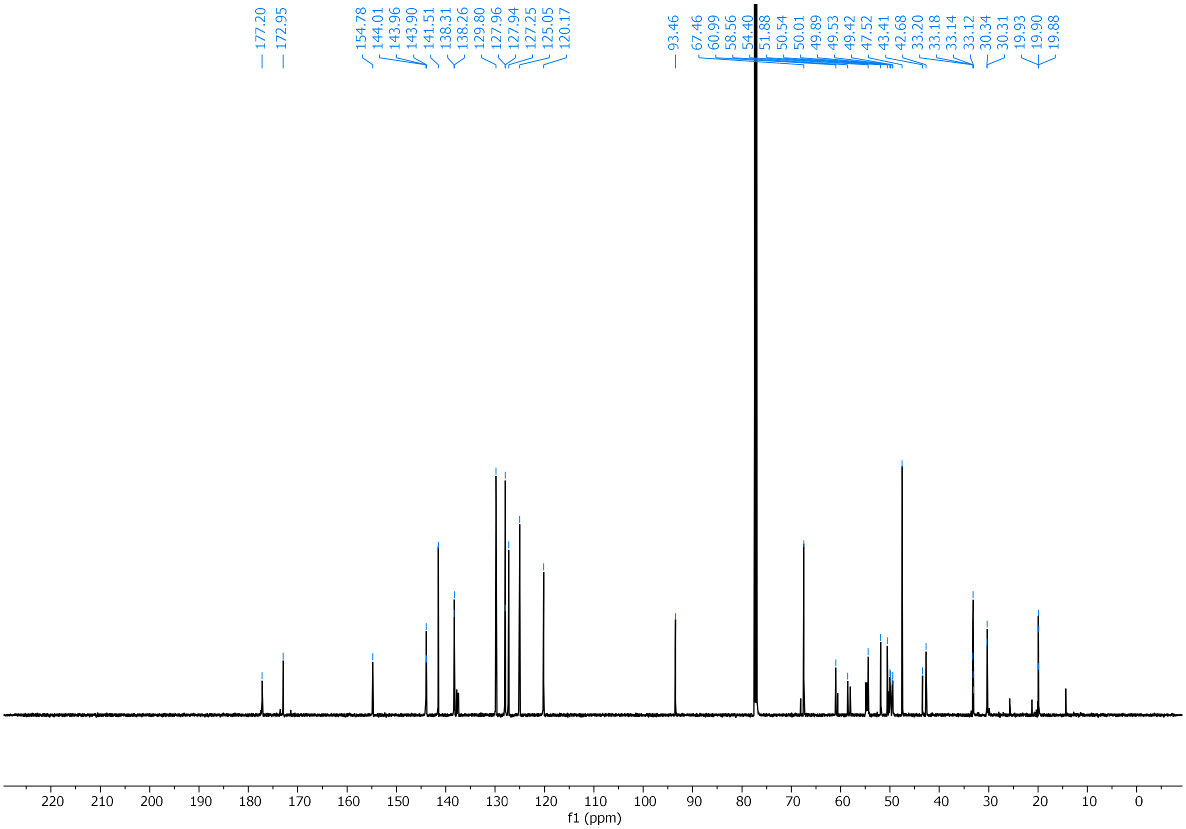

7.4. Enumerated DEL compounds off-DNA

(2*S*,4*S*)-4-(3-((*E*)-2-cyclohexylvinyl)phenoxy)-1-(3-ethynylbenzyl)-*N*-methylpyrrolidine-2-carboxamide (**27**)

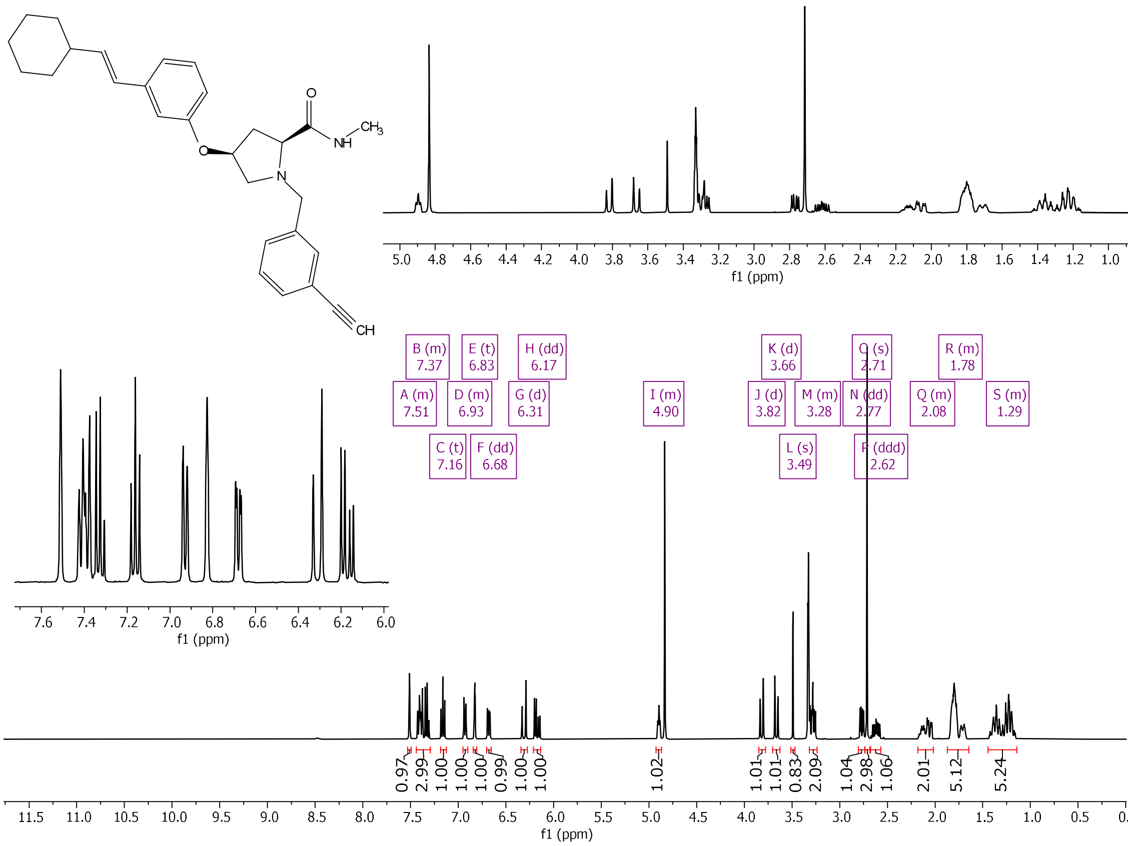

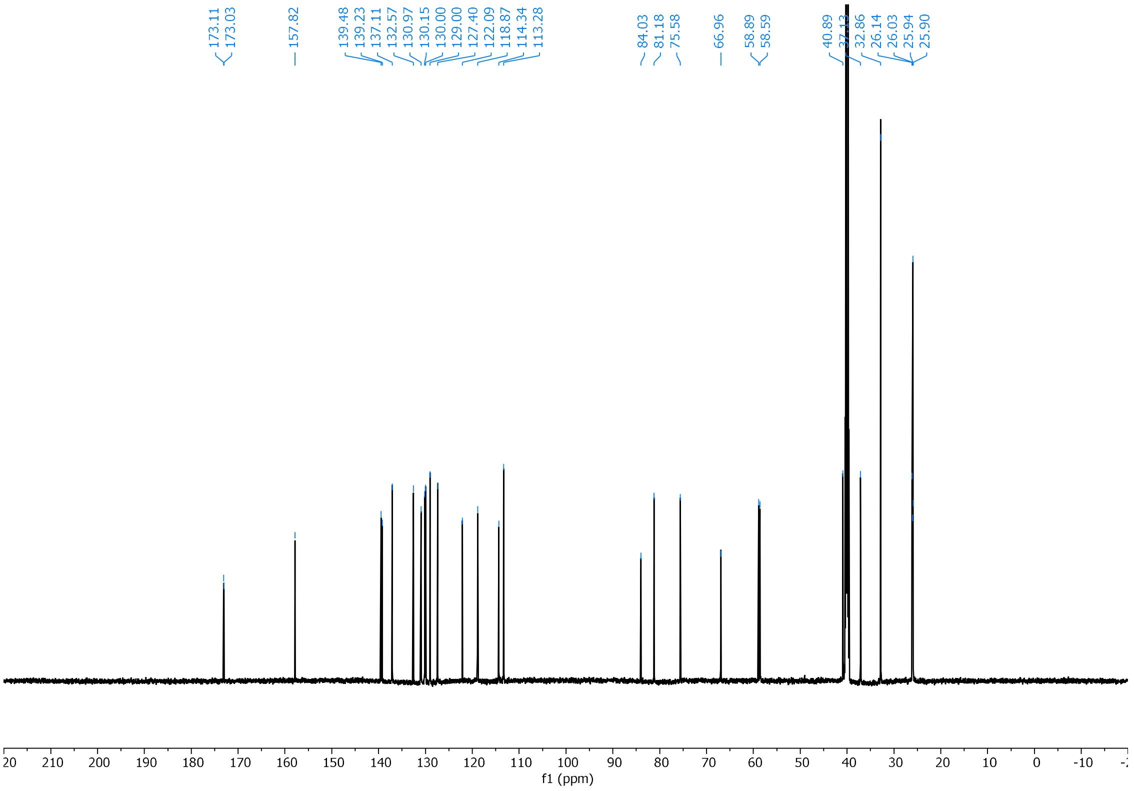

(2*R*,4*S*)-4-(3-((*E*)-2-cyclohexylvinyl)phenoxy)-1-(3-ethynylbenzyl)-*N*-methylpyrrolidine-2-carboxamide (**28**)

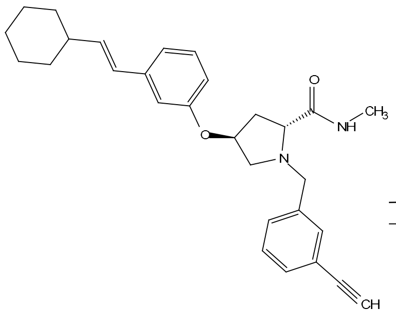

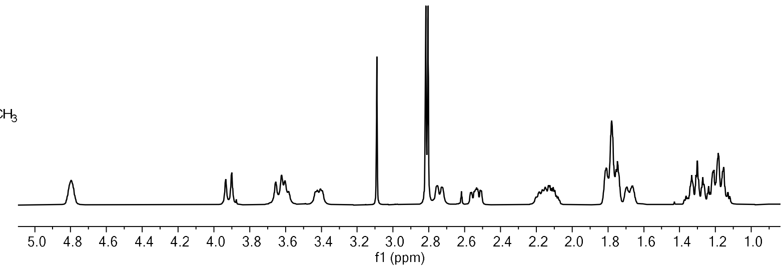

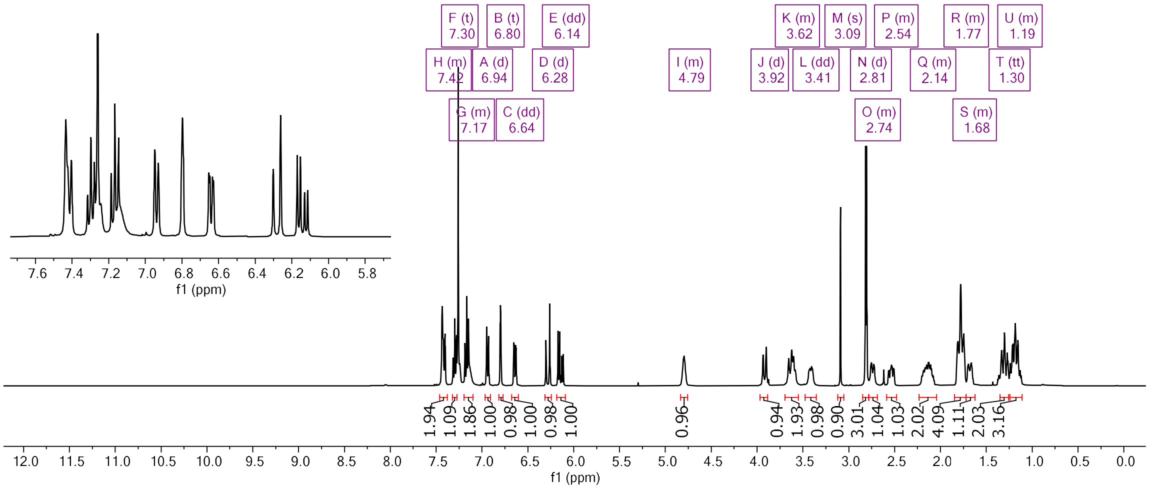

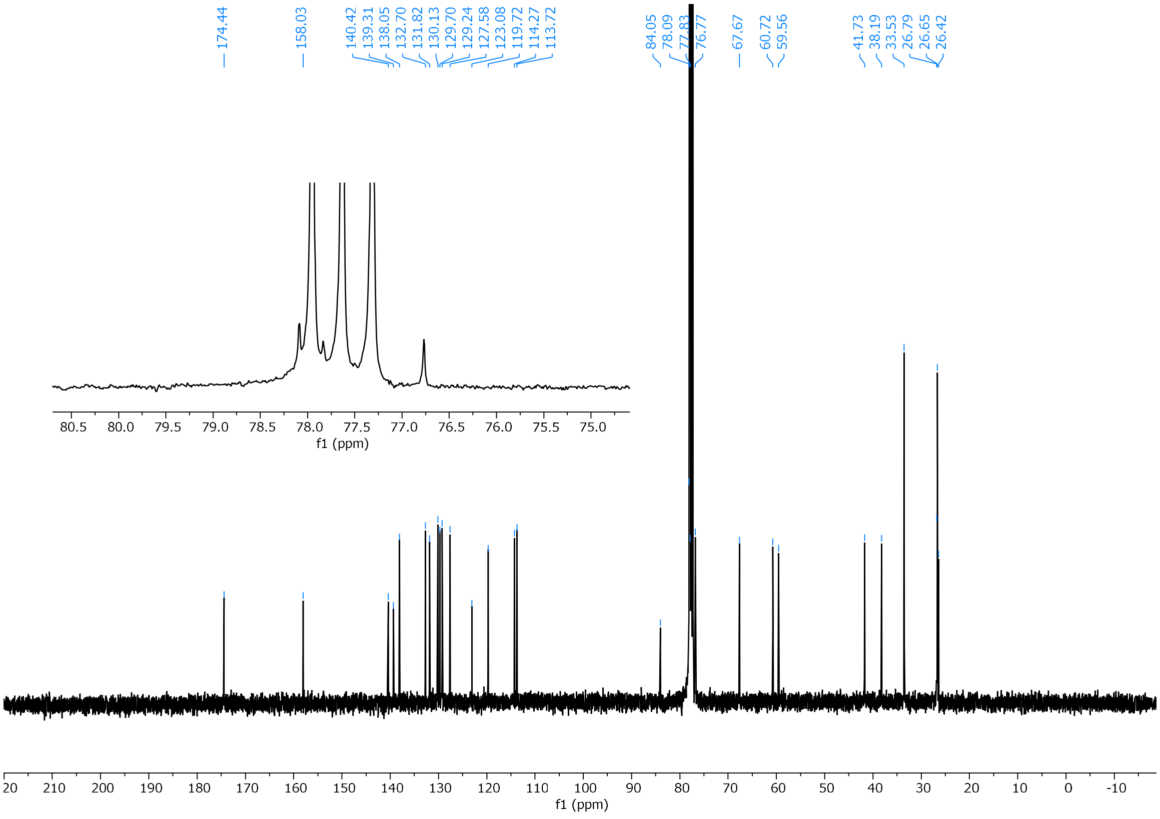

(2*S*,4*S*)-4-(4-((*E*)-2-cyclohexylvinyl)phenoxy)-1-(3-cyclopropyl-1-ethyl-1H-pyrazole-5-carbonyl)-*N*-methylpyrrolidine-2-carboxamide (**29**)

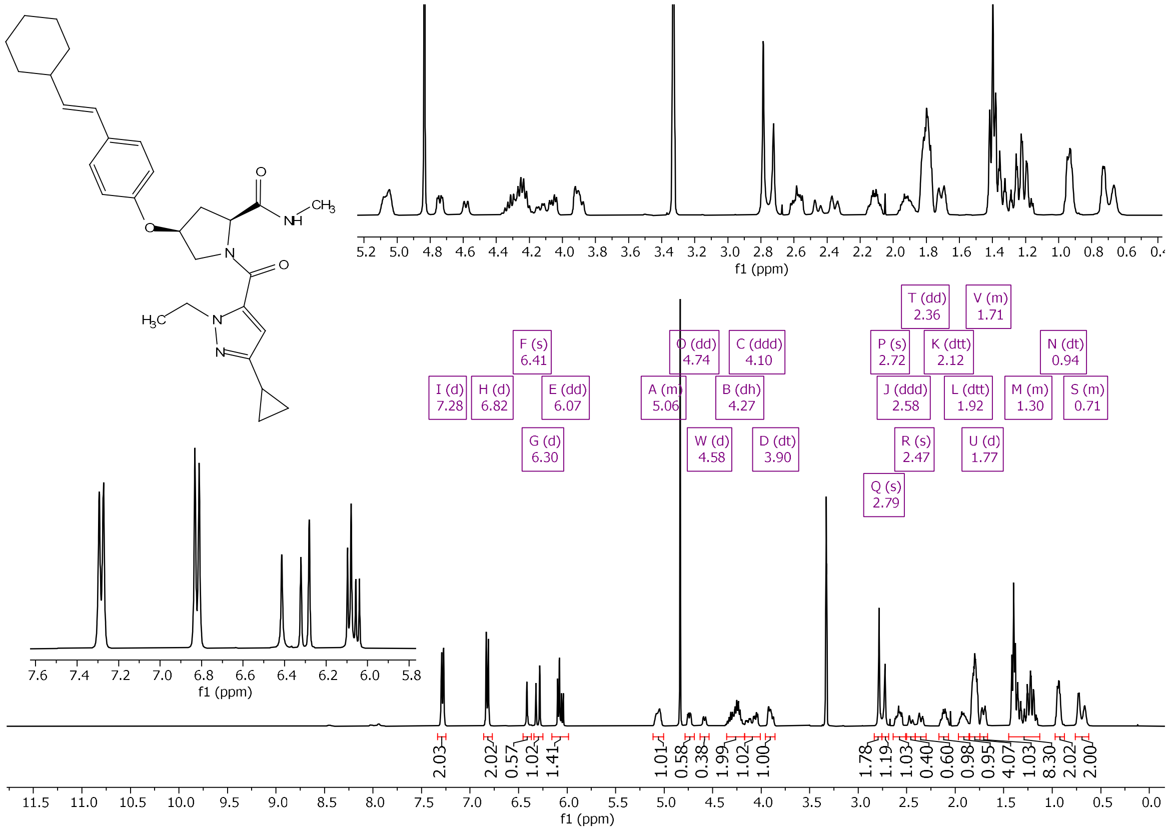

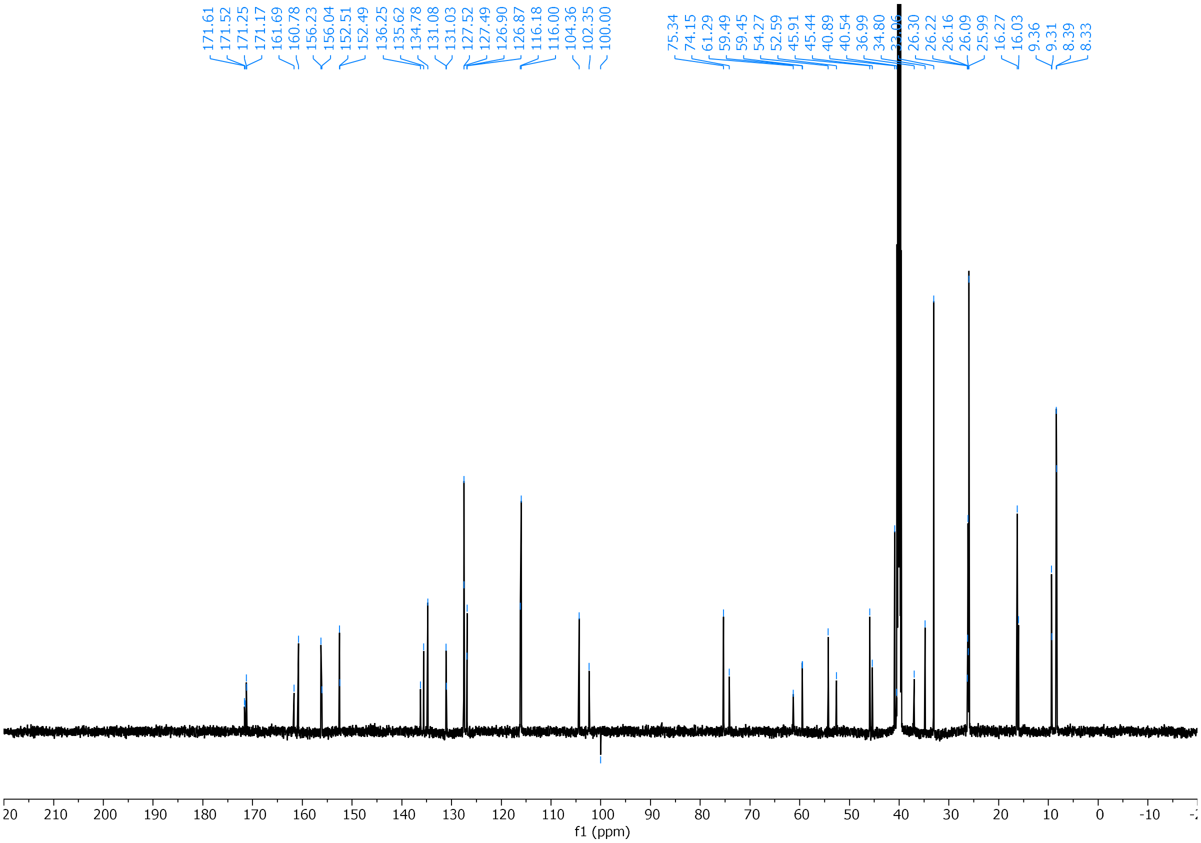
171.61
171.52
171.25
171.17
161.69
160.78
156.23
156.04
152.51
152.49
136.25
135.62
134.78
131.08
131.03
127.52
127.49
126.90
126.87
116.18
116.00
104.36
102.35
100.00
75.34
74.15
61.29
59.49
59.45
54.27
52.59
45.91
45.44
40.89
40.54
36.99
34.80
26.30
26.22
26.16
26.09
25.99
16.27
16.03
9.36
9.31
8.39
8.33
210
200
190
180
170
160
150
140
130
120
110
100
90
80
70
60
50
40
30
20
10
0
-10
f1 (ppm)

(2*S*,4*R*)-4-(4-((*E*)-2-cyclohexylvinyl)phenoxy)-1-(3-cyclopropyl-1-ethyl-1H-pyrazole-5-carbonyl)-*N*-methylpyrrolidine-2-carboxamide (**30**)

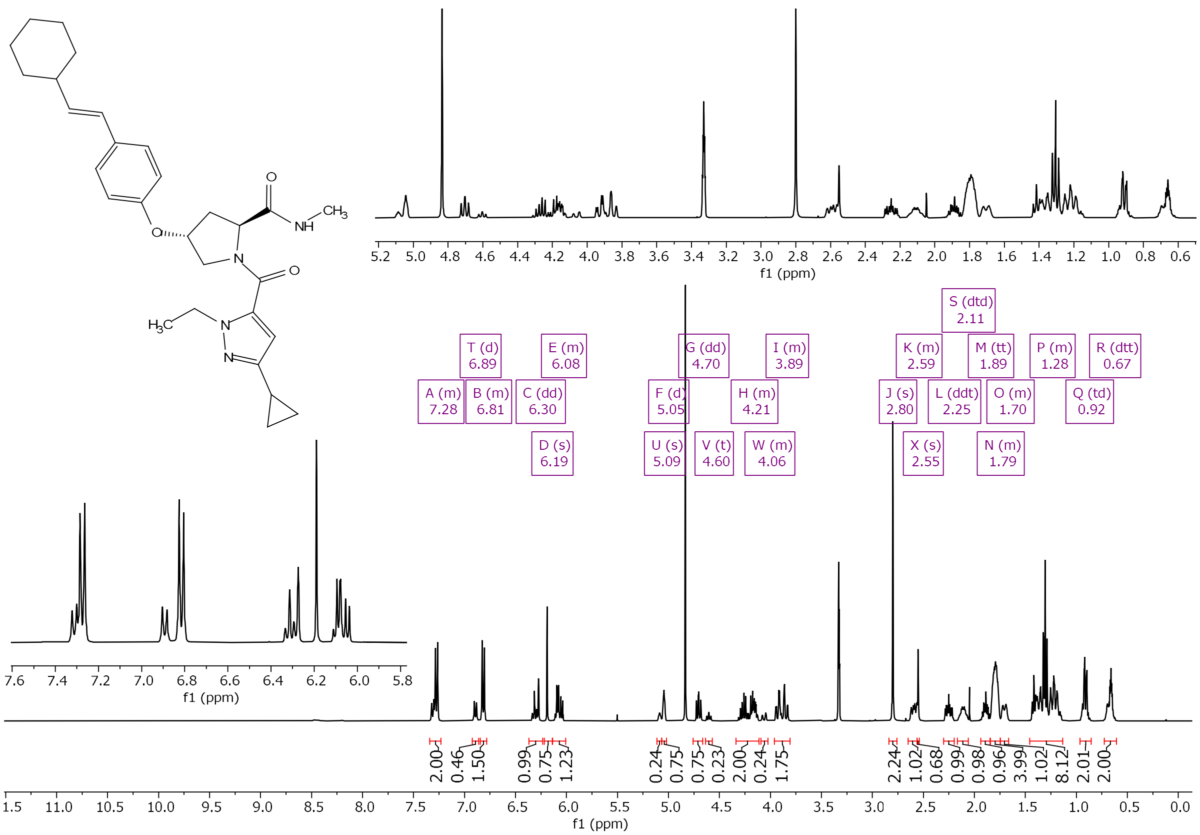

O
NH
CH3
O
N
O
H3C
N
N
A (m) 7.28
B (m) 6.81
C (dd) 6.30
D (s) 6.19
E (m) 6.08
F (d) 5.05
G (dd) 4.70
H (m) 4.21
I (m) 3.89
J (s) 2.80
K (m) 2.59
L (ddt) 2.25
M (tt) 1.89
N (m) 1.79
O (m) 1.70
P (m) 1.28
Q (td) 0.92
R (dtt) 0.67
S (dtd) 2.11
T (d) 6.89
U (s) 5.09
V (t) 4.60
W (m) 4.06
X (s) 2.55
2.00
0.46
1.50
0.99
0.75
1.23
0.24
0.75
0.75
0.23
2.00
0.24
1.75
2.24
1.02
0.68
0.99
0.98
0.96
3.99
1.02
8.12
2.01
2.00
f1 (ppm)
f1 (ppm)
f1 (ppm)

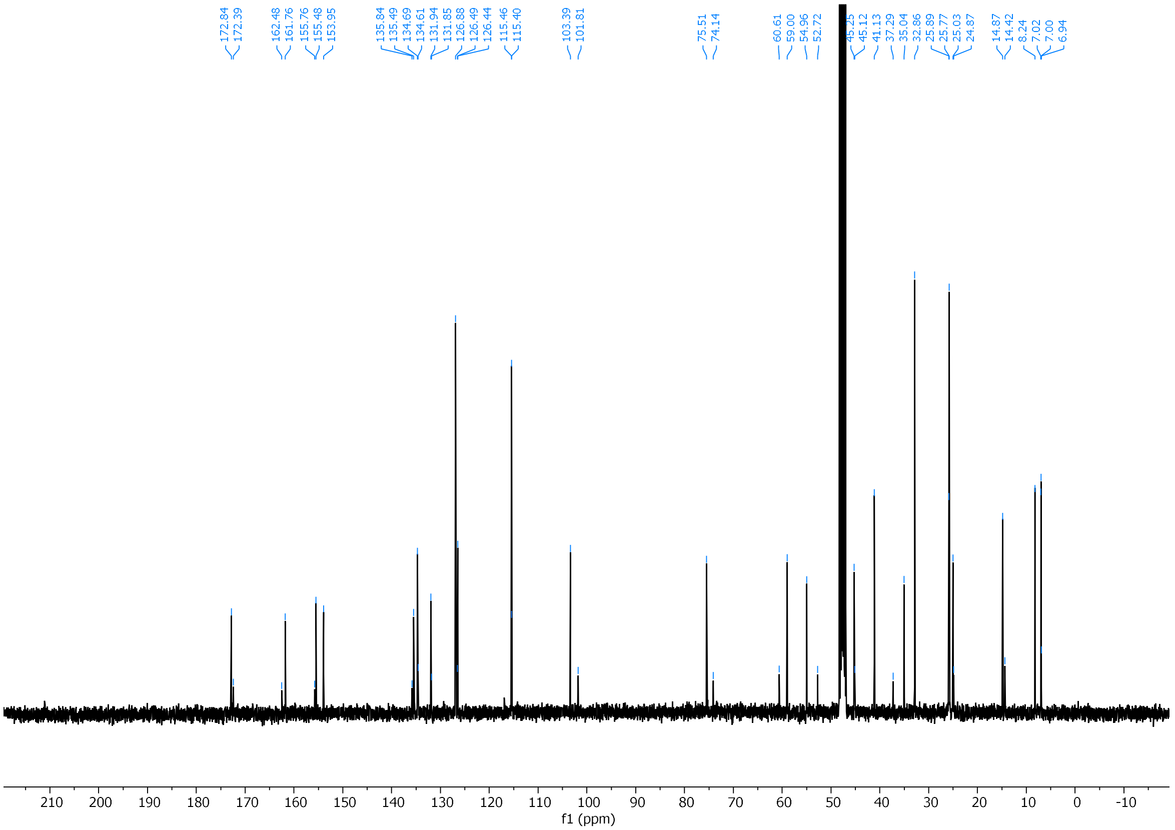

172.84
172.39
162.48
161.76
155.76
155.48
153.95
135.84
135.49
134.69
134.61
131.94
131.85
126.88
126.49
126.44
115.46
115.40
103.39
101.81
75.51
74.14
60.61
59.00
54.96
52.72
45.12
41.13
37.29
35.04
32.86
25.89
25.77
25.03
24.87
14.87
14.42
8.24
7.02
7.00
6.94
f1 (ppm)

(2*R*,4*R*)-4-(4-((*E*)-2-cyclohexylvinyl)phenoxy)-1-(3-cyclopropyl-1-ethyl-1H-pyrazole-5-carbonyl)-*N*-methylpyrrolidine-2-carboxamide (**31**)

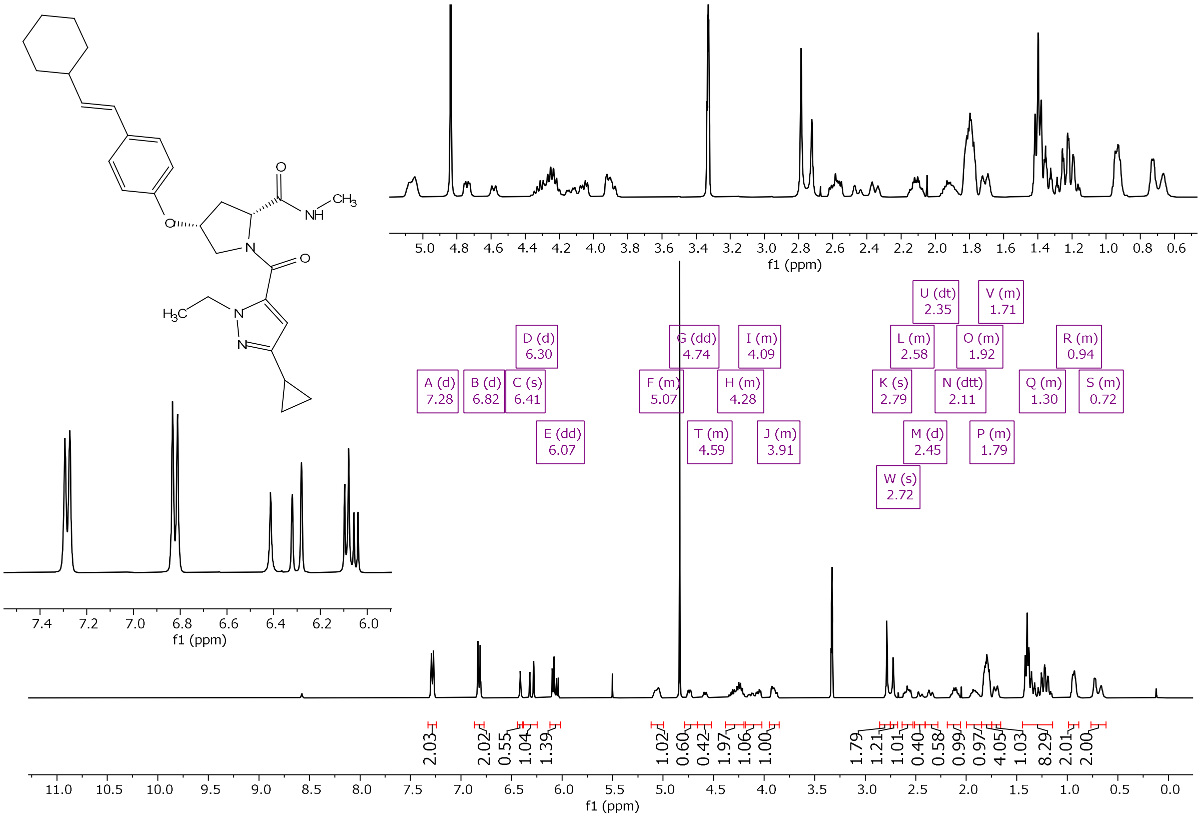

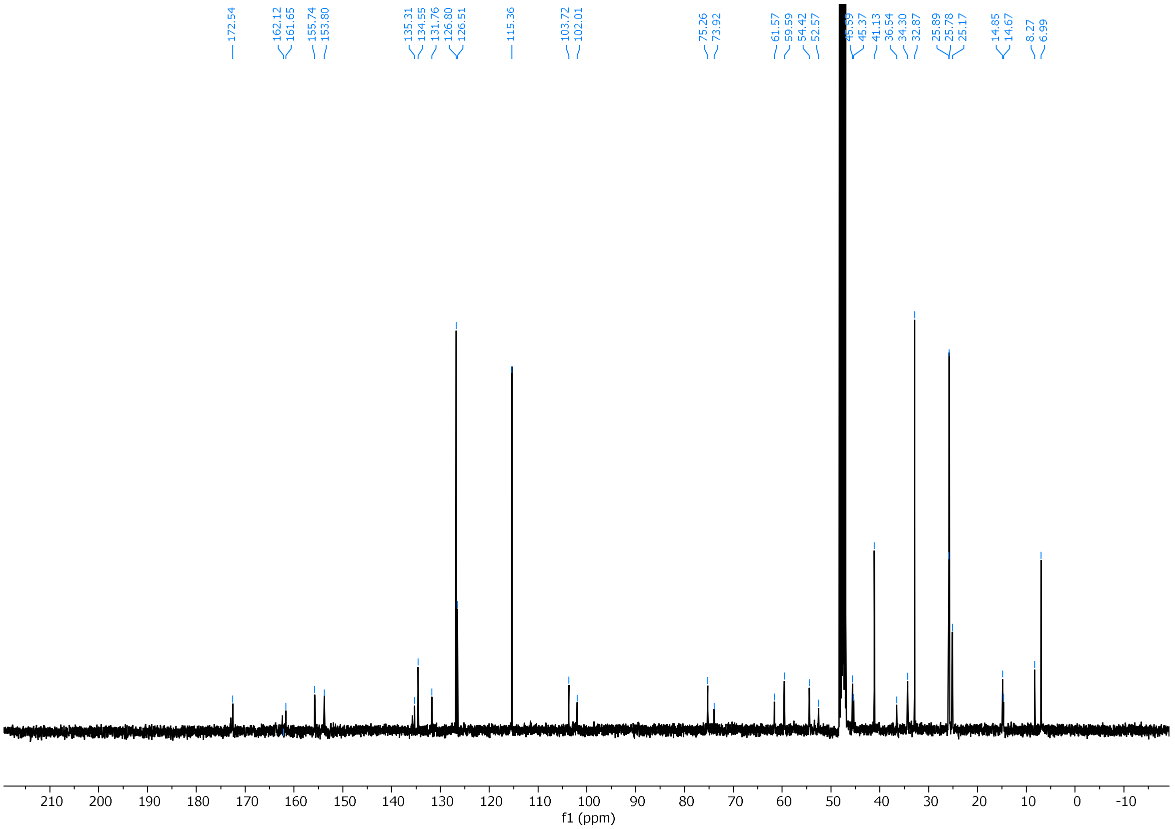

(2*R*,4*S*)-4-(4-((*E*)-2-cyclohexylvinyl)phenoxy)-1-(3-(2,3-dihydro-1H-inden-1-yl)propanoyl)-*N*-methylpyrrolidine-2-carboxamide (**32**)

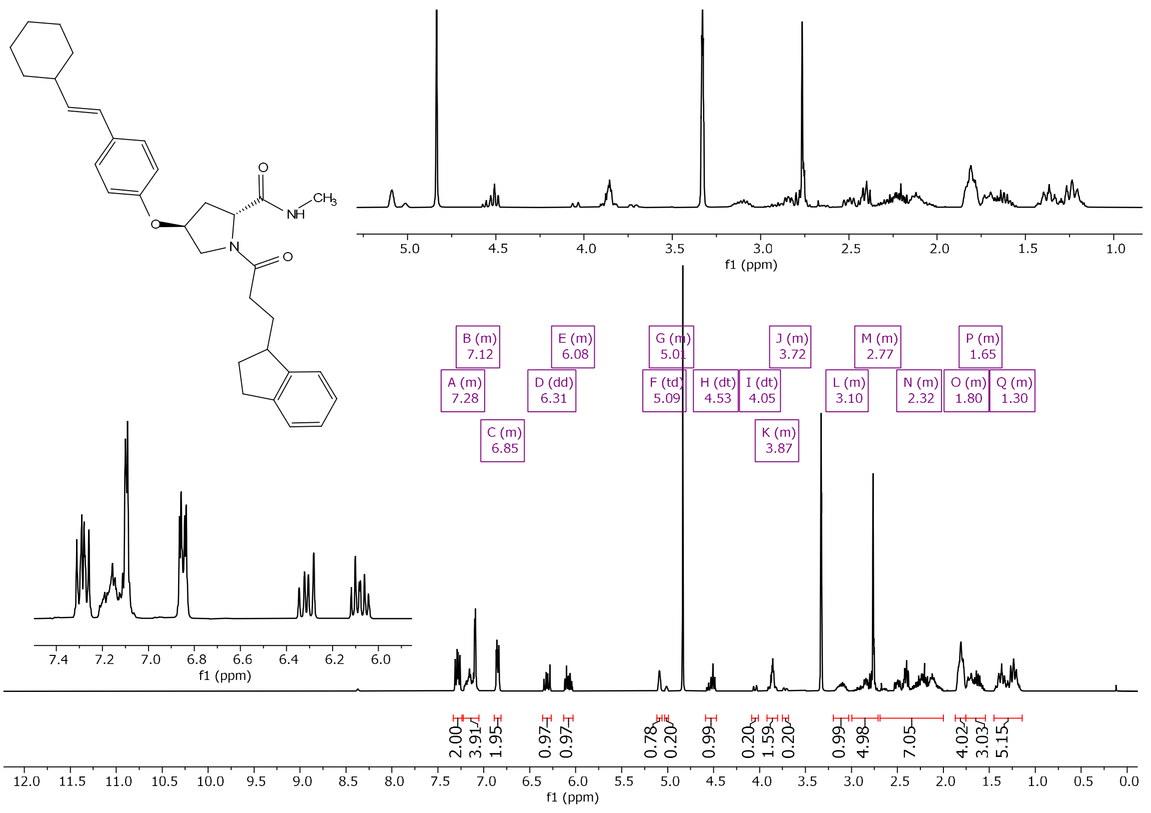

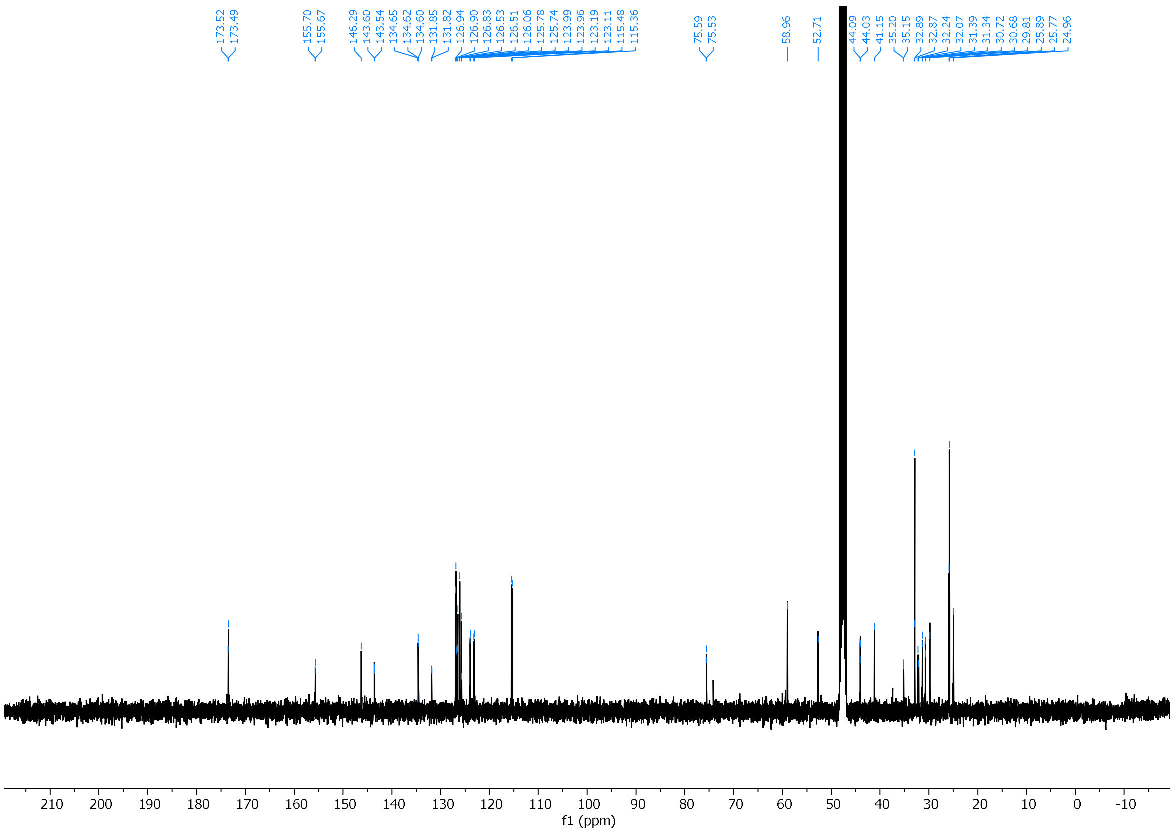
173.52
173.49
155.70
155.67
146.29
143.60
143.54
134.65
134.62
134.60
131.85
131.82
126.94
126.90
126.83
126.53
126.51
126.06
125.78
125.74
123.99
123.96
123.19
123.11
115.48
115.36
75.59
75.53
58.96
52.71
44.09
44.03
41.15
35.20
35.15
32.89
32.87
32.24
32.07
31.39
31.34
30.72
30.68
29.81
25.89
25.77
24.96
210
200
190
180
170
160
150
140
130
120
110
100
90
80
70
60
50
40
30
20
10
0
-10
f1 (ppm)

(2*R*,4*S*)-4-(4-((*E*)-2-cyclohexylvinyl)phenoxy)-1-(3-cyclopropyl-1-ethyl-1H-pyrazole-5-carbonyl)-*N*-methylpyrrolidine-2-carboxamide (**33**)

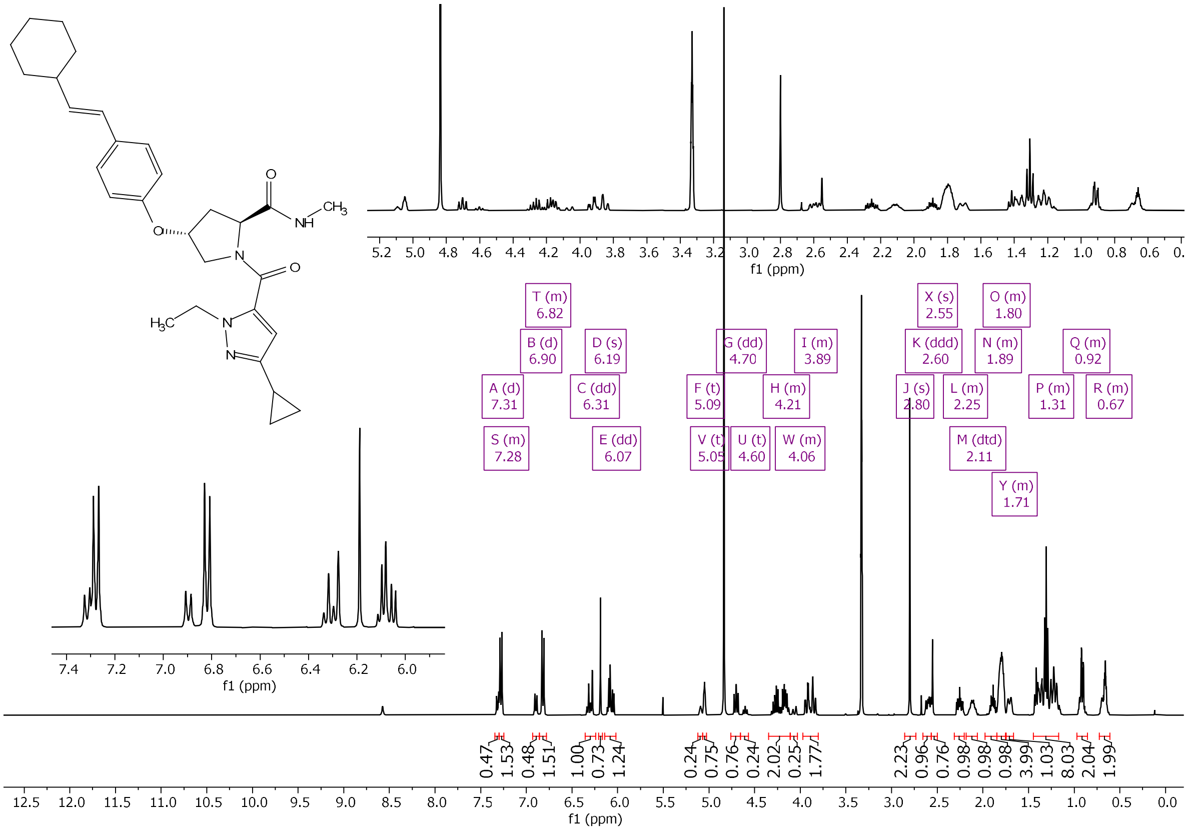
O
NH
CH_3
O
N
O
H_3C
N
N
A (d) 7.31
B (d) 6.90
C (dd) 6.31
D (s) 6.19
E (dd) 6.07
F (t) 5.09
G (dd) 4.70
H (m) 4.21
I (m) 3.89
J (s) 2.80
K (ddd) 2.60
L (m) 2.25
M (dtd) 2.11
N (m) 1.89
O (m) 1.80
P (m) 1.31
Q (m) 0.92
R (m) 0.67
S (m) 7.28
T (m) 6.82
U (t) 4.60
V (t) 5.05
W (m) 4.06
X (s) 2.55
Y (m) 1.71
0.47
1.53
0.48
1.51
1.00
0.73
1.24
0.24
0.75
0.76
0.24
2.02
0.25
1.77
2.23
0.96
0.76
0.98
0.98
0.98
3.99
1.03
8.03
2.04
1.99
f1 (ppm)

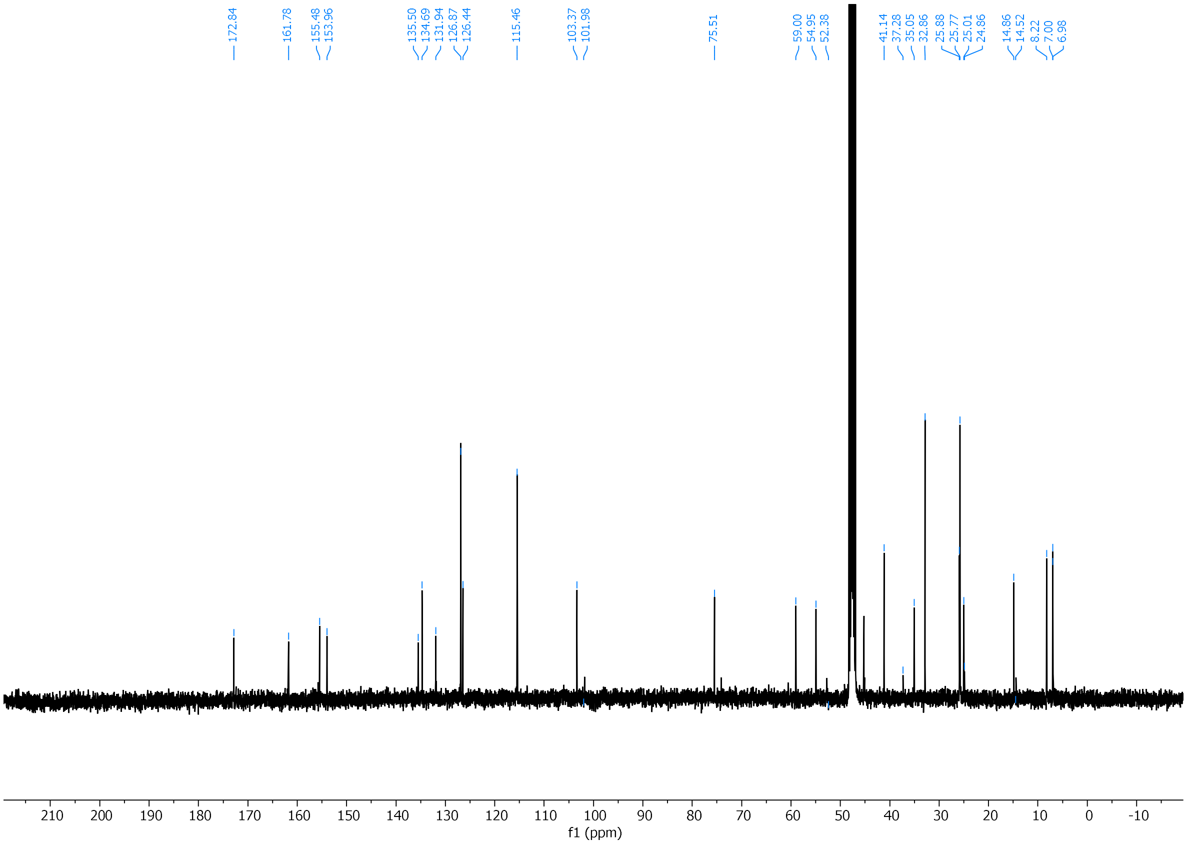
172.84
161.78
155.48
153.96
135.50
134.69
131.94
126.87
126.44
115.46
103.37
101.98
75.51
59.00
54.95
52.38
41.14
37.28
35.05
32.86
25.88
25.77
25.01
24.86
14.86
14.52
8.22
7.00
6.98
f1 (ppm)

(6*R*,7*S*,8*S*)-4-((3-chloro-4-fluorophenyl)sulfonyl)-7-(4-((*E*)-2-cyclohexylvinyl)phenyl)-8-(hydroxymethyl)-1,4-diazabicyclo[4.2.0]octan-2-one (**34**)

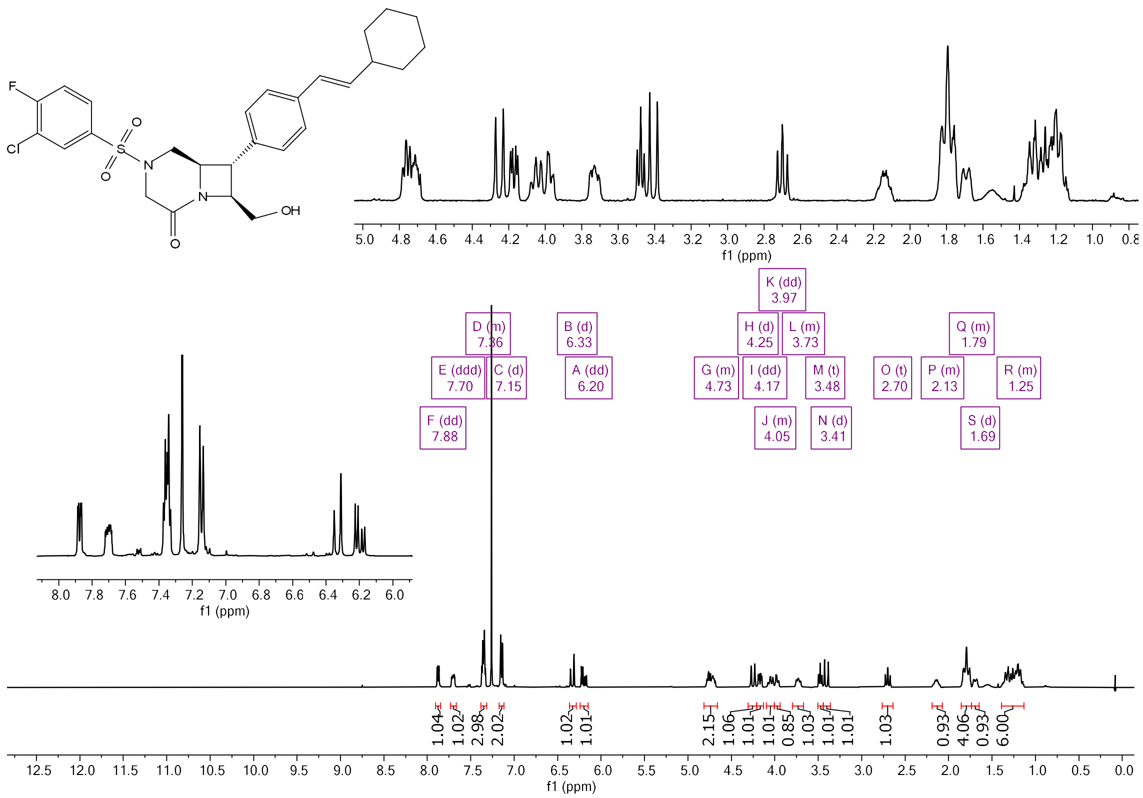

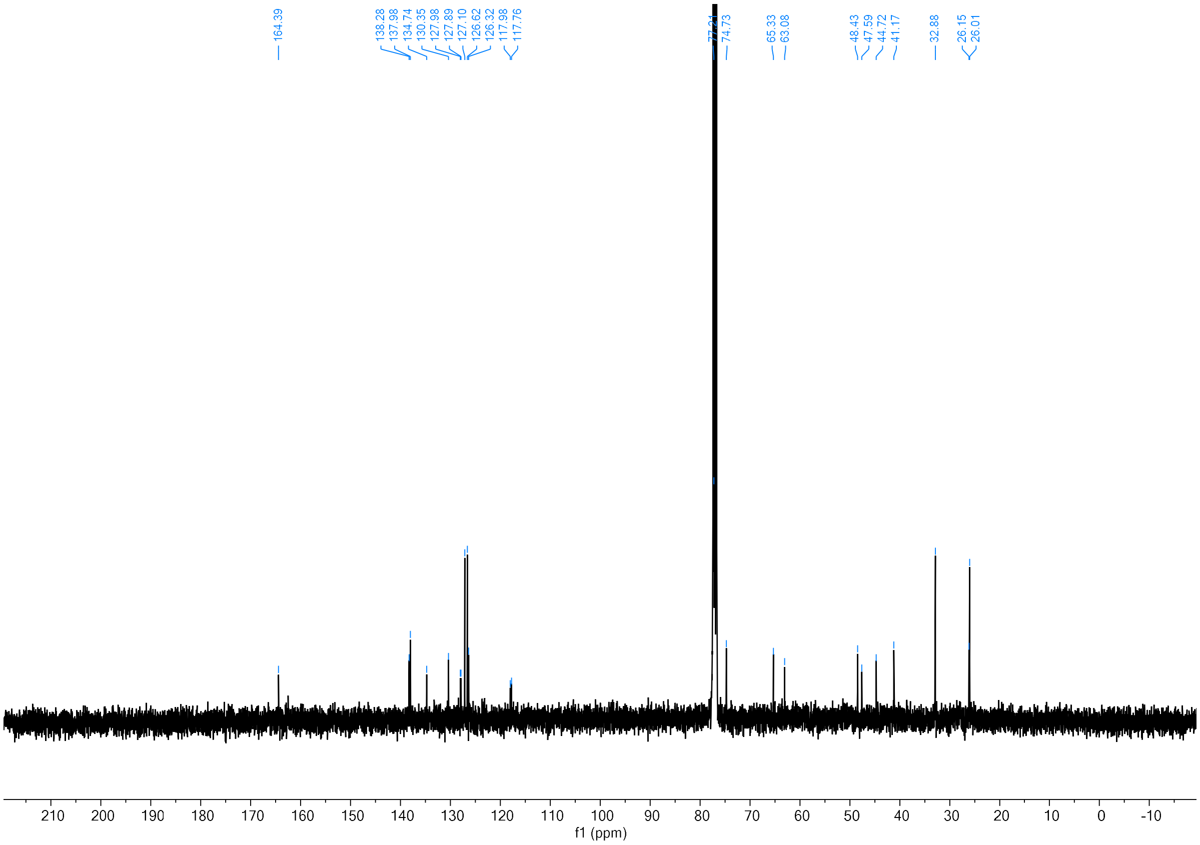
164.39
138.28
137.98
134.74
130.35
127.98
127.89
127.10
126.62
126.32
117.98
117.76
74.73
65.33
63.08
48.43
47.59
44.72
41.17
32.88
26.15
26.01
210 200 190 180 170 160 150 140 130 120 110 100 90 80 70 60 50 40 30 20 10 0 -10
f1 (ppm)

N-(((4*R*,5*R*)-8-((*E*)-2-cyclohexylvinyl)-2-((*R*)-1-hydroxypropan-2-yl)-4-methyl-1,1-dioxido-2,3,4,5-tetrahydrobenzo[*b*][1,4,5]oxathiazocin-5-yl)methyl)-*N*-methyl-2-phenylcyclopent-1-ene-1-carboxamide (**35**)

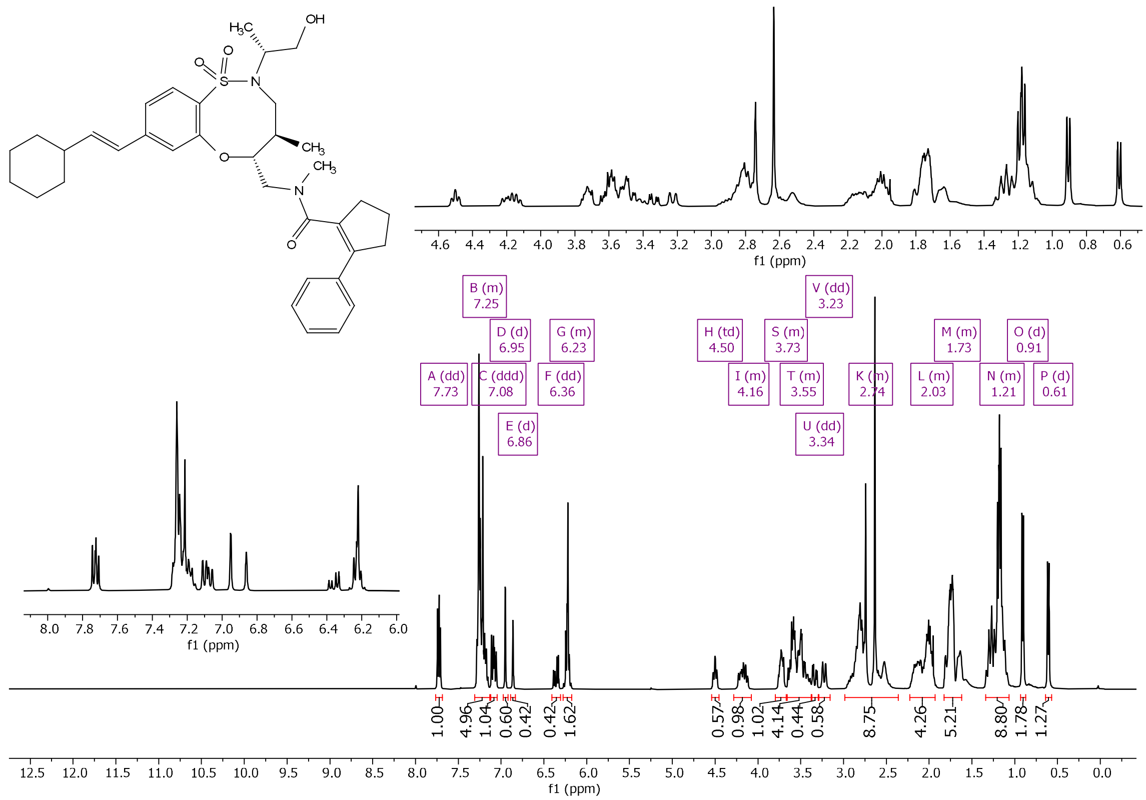

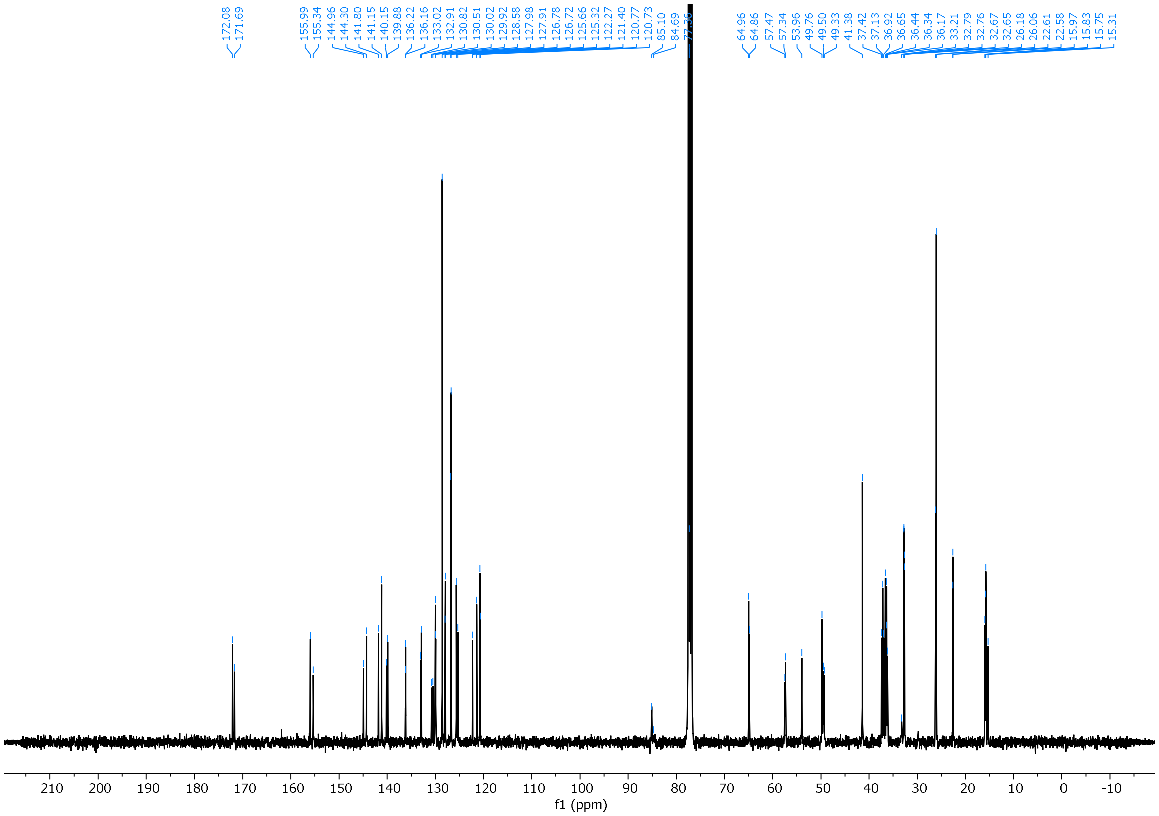

172.08
171.69
155.99
155.34
144.96
144.30
141.80
141.15
140.15
139.88
136.22
136.16
133.02
132.91
130.82
130.51
130.02
129.92
128.58
127.98
127.91
126.78
126.72
125.66
125.32
122.27
121.40
120.77
120.73
85.10
84.69
64.96
64.86
57.47
57.34
53.96
49.76
49.50
49.33
41.38
37.42
37.13
36.92
36.65
36.44
36.34
36.17
33.21
32.79
32.76
32.67
32.65
26.18
26.06
22.61
22.58
15.97
15.83
15.75
15.31
210 200 190 180 170 160 150 140 130 120 110 100 90 80 70 60 50 40 30 20 10 0 -10
f1 (ppm)

N-(((4*S*,5*S*)-8-((*E*)-2-cyclohexylvinyl)-2-((*R*)-1-hydroxypropan-2-yl)-4-methyl-1,1-dioxido-2,3,4,5-tetrahydrobenzo[*b*][1,4,5]oxathiazocin-5-yl)methyl)-*N*-methyl-2-(3-methylthiophen-2-yl)cyclopropane-1-carboxamide (**36**)

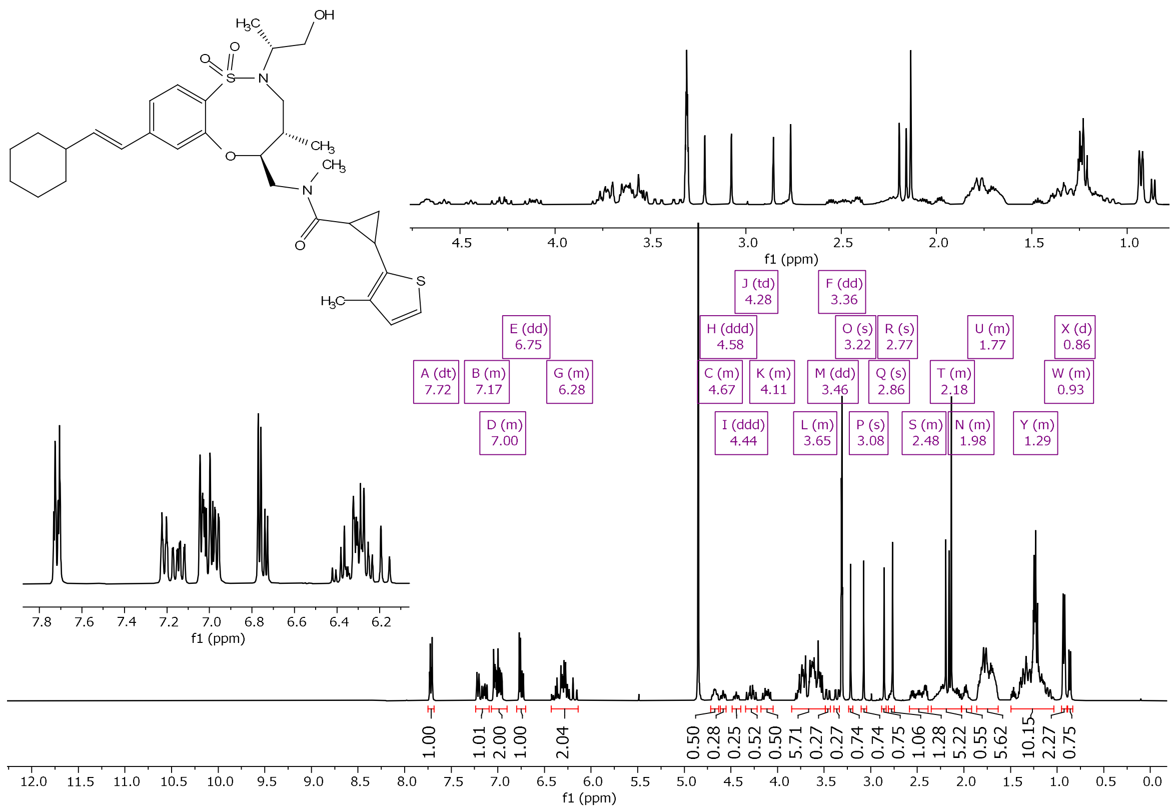

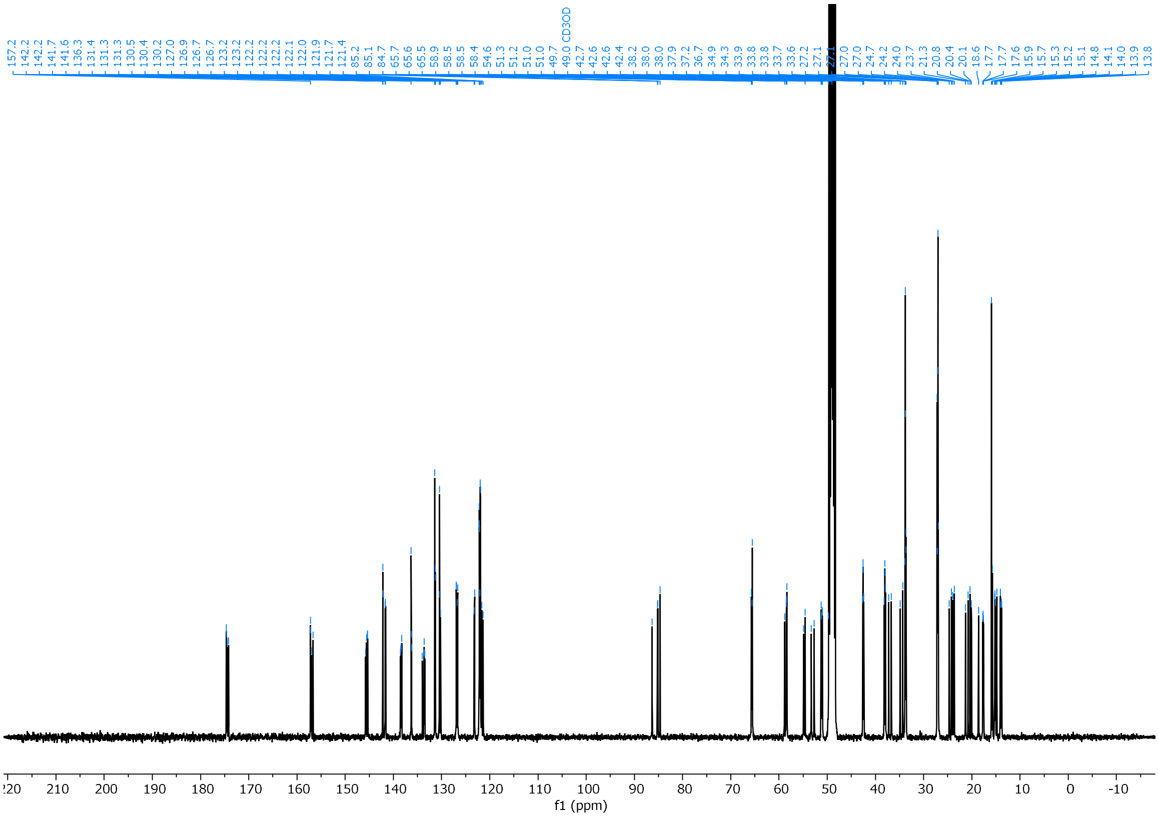
157.2
142.2
142.2
141.7
141.6
136.3
131.4
131.3
131.3
130.5
130.4
130.2
127.0
126.9
126.7
126.7
123.2
123.2
122.2
122.2
122.2
122.2
122.1
122.0
121.9
121.7
121.4
85.2
85.1
84.7
65.7
65.6
65.5
58.9
58.5
58.5
58.4
54.6
51.3
51.2
51.0
51.0
49.7
49.0 CD3OD
42.7
42.6
42.6
42.4
38.2
38.0
38.0
37.9
37.2
36.7
34.9
34.3
33.9
33.8
33.8
33.7
33.6
27.2
27.1
27.1
27.0
27.0
24.7
24.2
24.0
23.7
21.3
20.8
20.4
20.1
18.6
17.7
17.7
17.6
15.9
15.7
15.3
15.2
15.1
14.8
14.1
14.0
13.9
13.8
210 200 190 180 170 160 150 140 130 120 110 100 90 80 70 60 50 40 30 20 10 0 -10
f1 (ppm)

N-(((4*S*,5*S*)-8-(2-chloro-5-(hydroxymethyl)phenyl)-2-((*R*)-1-hydroxypropan-2-yl)-4-methyl-1,1-dioxido-2,3,4,5-tetrahydrobenzo[*b*][1,4,5]oxathiazocin-5-yl)methyl)-6-ethyl-*N*-methyl-2-oxo-1,2-dihydropyridine-4-carboxamide (**37**)

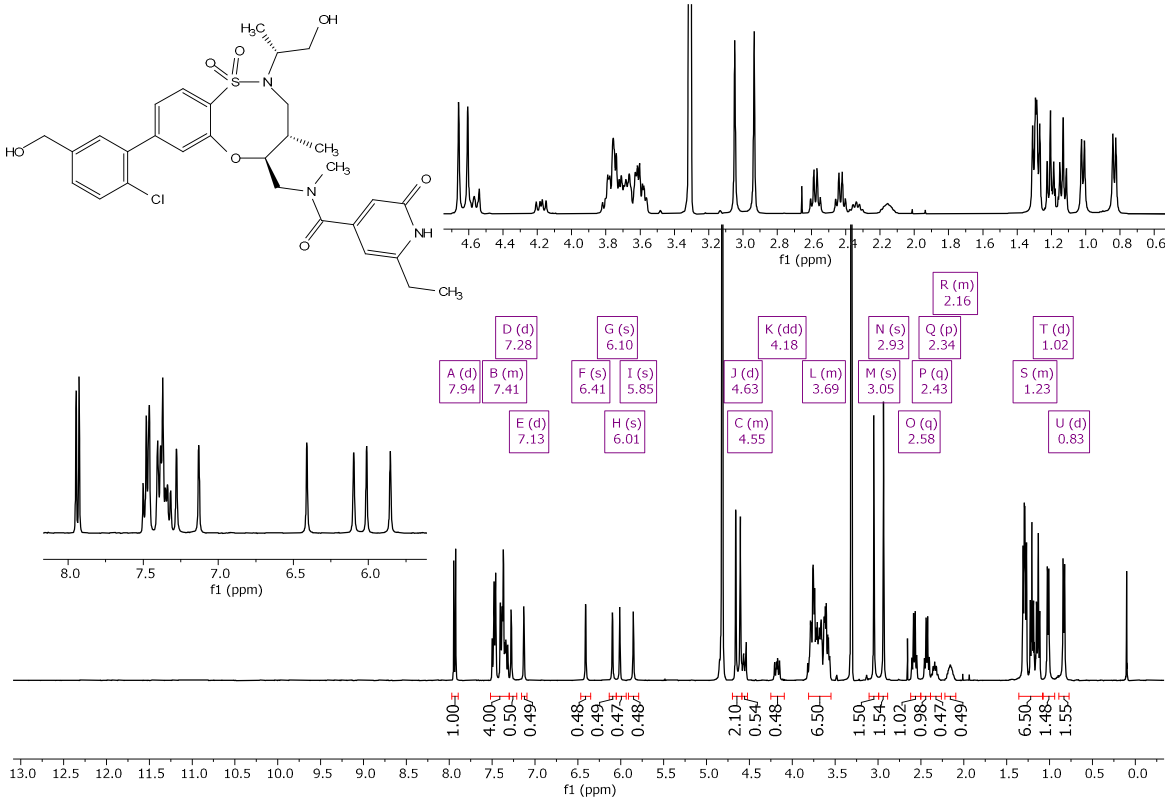

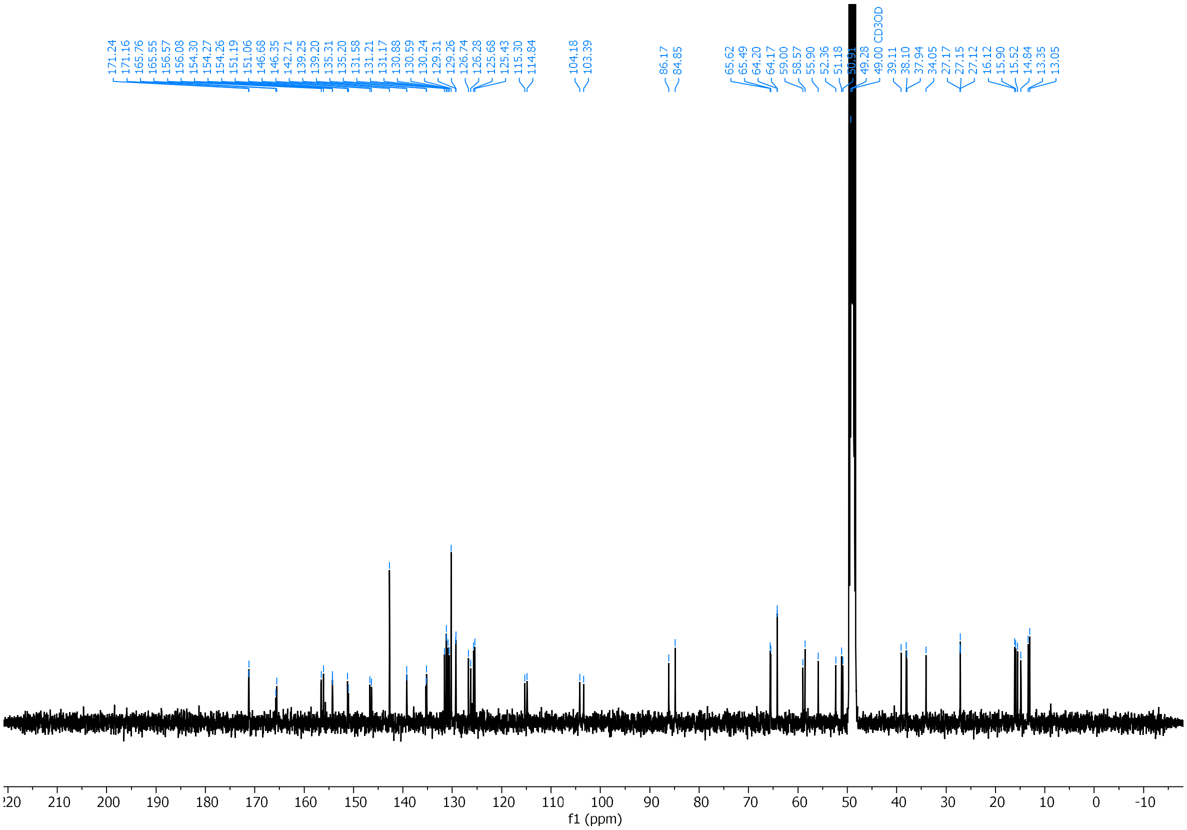
171.24
171.16
165.76
165.55
156.57
156.08
154.30
154.27
154.26
151.19
151.06
146.68
146.35
142.71
139.25
139.20
135.31
135.20
131.58
131.21
131.17
130.88
130.59
130.24
129.31
129.26
126.74
126.28
125.68
125.43
115.30
114.84
104.18
103.39
86.17
84.85
65.62
65.49
64.20
64.17
59.00
58.57
55.90
52.36
51.18
49.28
49.00 CD3OD
39.11
38.10
37.94
34.05
27.17
27.15
27.12
16.12
15.90
15.52
14.84
13.35
13.05
210
200
190
180
170
160
150
140
130
120
110
100
90
80
70
60
50
40
30
20
10
0
-10
f1 (ppm)

N-(((4*R*,5*R*)-8-((*E*)-2-cyclohexylvinyl)-2-((*S*)-1-hydroxypropan-2-yl)-4-methyl-1,1-dioxido-2,3,4,5-tetrahydrobenzo[*b*][1,4,5]oxathiazocin-5-yl)methyl)-*N*-methyl-2-phenylcyclopent-1-ene-1-carboxamide (**38**)

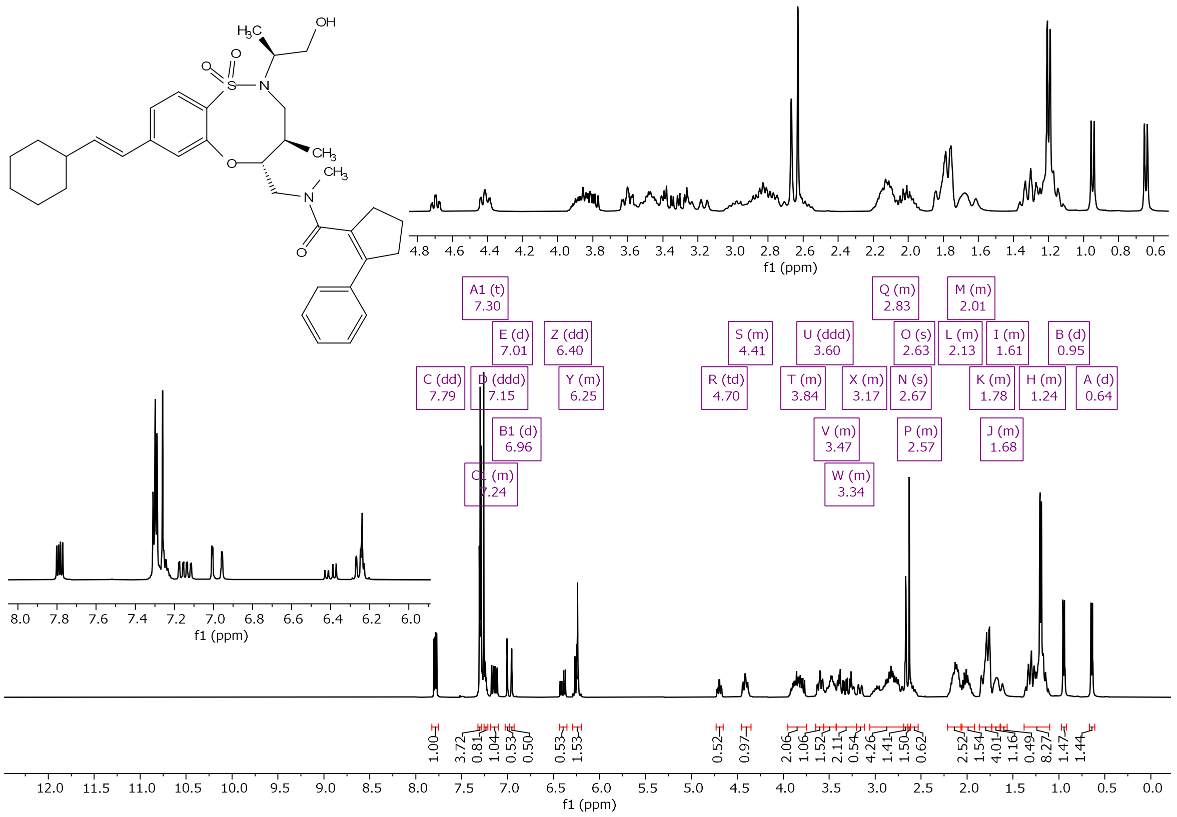
H3C
OH
O
O
S
N
CH3
O
CH3
N
O
A1 (t) 7.30
E (d) 7.01
Z (dd) 6.40
C (dd) 7.79
D (ddd) 7.15
Y (m) 6.25
B1 (d) 6.96
C1 (m) 7.24
S (m) 4.41
R (td) 4.70
U (ddd) 3.60
T (m) 3.84
X (m) 3.17
V (m) 3.47
W (m) 3.34
Q (m) 2.83
O (s) 2.63
N (s) 2.67
P (m) 2.57
M (m) 2.01
L (m) 2.13
I (m) 1.61
K (m) 1.78
J (m) 1.68
H (m) 1.24
B (d) 0.95
A (d) 0.64
1.00
3.72
0.81
1.04
0.53
0.50
0.53
1.53
0.52
0.97
2.06
1.06
1.52
2.11
0.54
4.26
1.41
1.50
0.62
2.52
1.54
4.01
1.16
0.49
8.27
1.47
1.44
f1 (ppm)

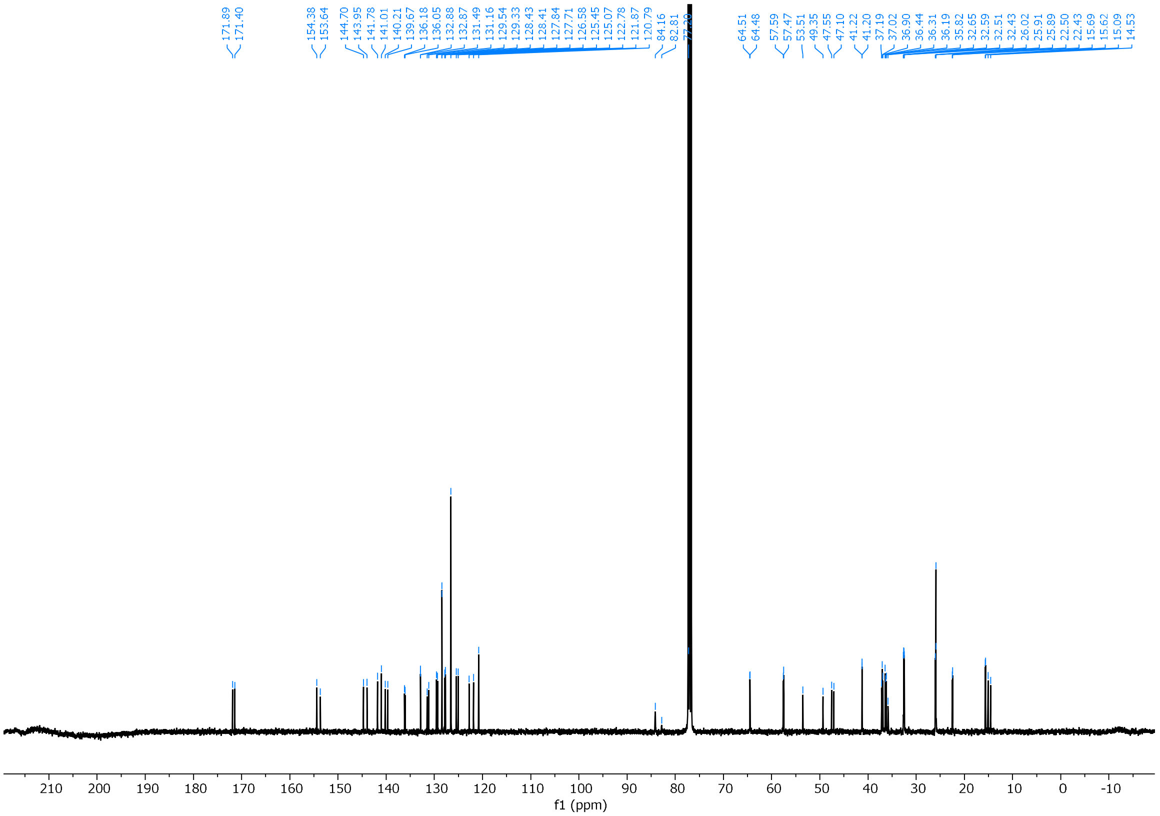
171.89
171.40
154.38
153.64
144.70
143.95
141.78
141.01
140.21
139.67
136.18
136.05
132.88
132.87
131.49
131.16
129.54
129.33
128.43
128.41
127.84
127.71
126.58
125.45
125.07
122.78
121.87
120.79
84.16
82.81
64.51
64.48
57.59
57.47
53.51
49.35
47.55
47.10
41.22
41.20
37.19
37.02
36.90
36.44
36.31
36.19
35.82
32.65
32.59
32.51
32.43
26.02
25.91
25.89
22.50
22.43
15.69
15.62
15.09
14.53
f1 (ppm)

N-(((4*R*,5*R*)-8-((*E*)-2-cyclohexylvinyl)-2-((*S*)-1-hydroxypropan-2-yl)-4-methyl-1,1-dioxido-2,3,4,5-tetrahydrobenzo[*b*][1,4,5]oxathiazocin-5-yl)methyl)-2-(2,3-dihydrobenzofuran-3-yl)-*N*-methylacetamide (**39**)

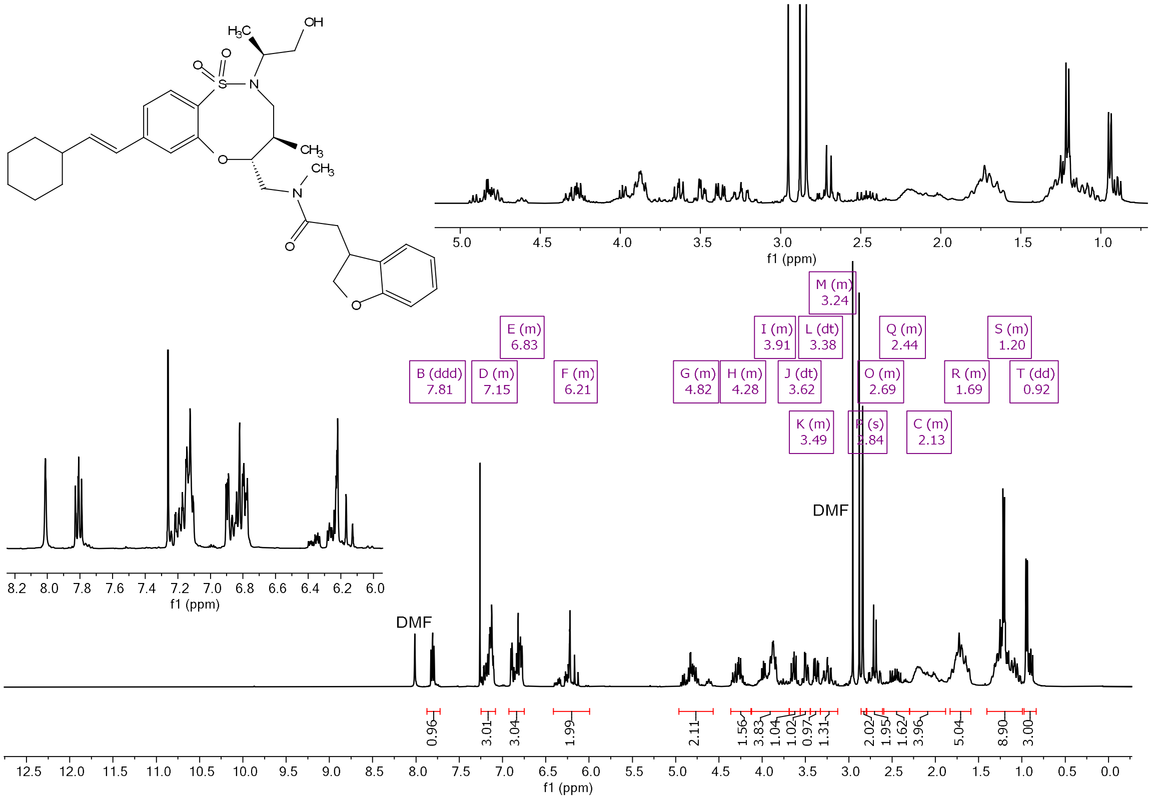

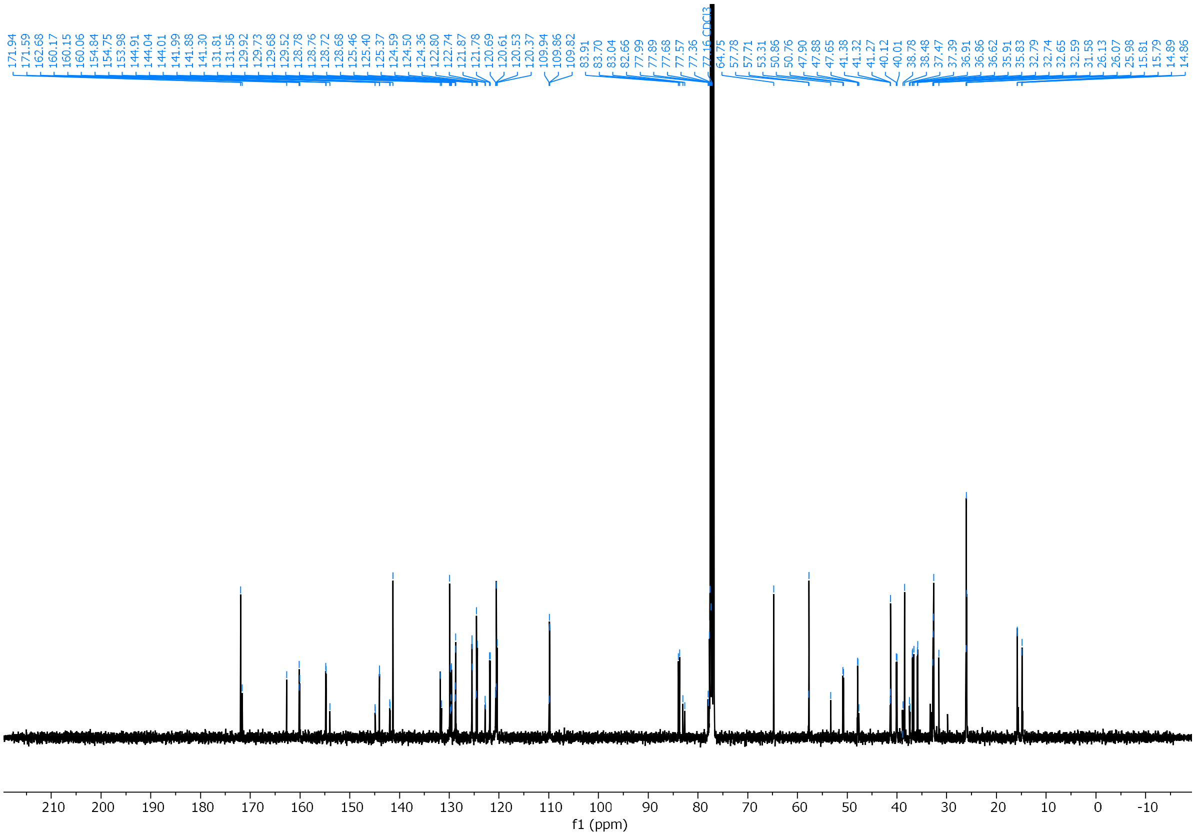
171.94
171.59
162.68
160.17
160.15
160.06
154.84
154.75
153.98
144.91
144.04
144.01
141.99
141.88
141.30
131.81
131.56
129.92
129.73
129.68
129.52
128.78
128.76
128.72
128.68
125.46
125.40
125.37
124.59
124.50
124.36
122.80
122.74
121.87
121.78
120.69
120.61
120.53
120.37
109.94
109.86
109.82
83.91
83.70
83.04
82.66
77.99
77.89
77.68
77.57
77.36
77.16 CDCl3
64.75
57.78
57.71
53.31
50.86
50.76
47.90
47.88
47.65
41.38
41.32
41.27
40.12
40.01
38.78
38.48
37.47
37.39
36.91
36.86
36.62
35.91
35.83
32.79
32.74
32.65
32.59
31.58
26.13
26.07
25.98
15.81
15.79
14.89
14.86
210 200 190 180 170 160 150 140 130 120 110 100 90 80 70 60 50 40 30 20 10 0 -10
f1 (ppm)

3-(benzo[*d*][1,3]dioxol-5-yl)-*N*-(((4*R*,5*R*)-2-((*S*)-1-hydroxypropan-2-yl)-4-methyl-1,1-dioxido-8-(1-oxo-2,3,4,5-tetrahydro-1H-benzo[*c*]azepin-7-yl)-2,3,4,5-tetrahydrobenzo[*b*][1,4,5]oxathiazocin-5-yl)methyl)-*N*-methylpropiolamide (**40**)

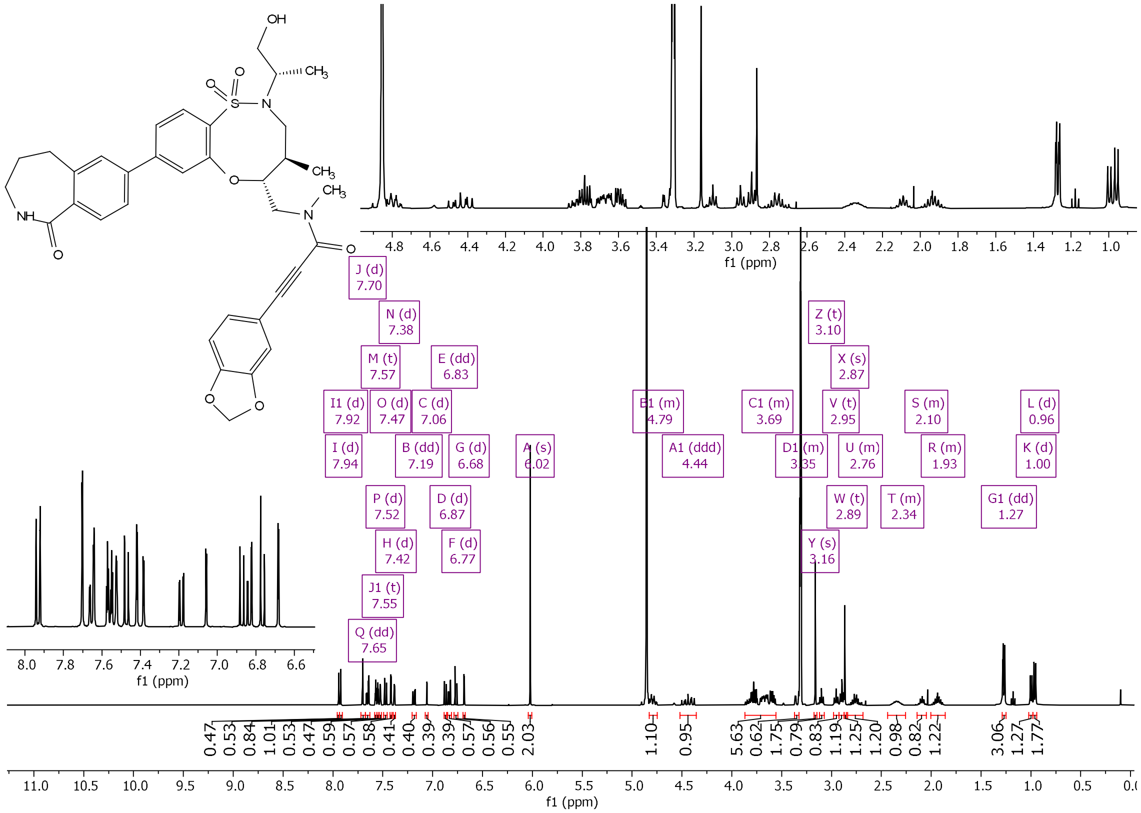

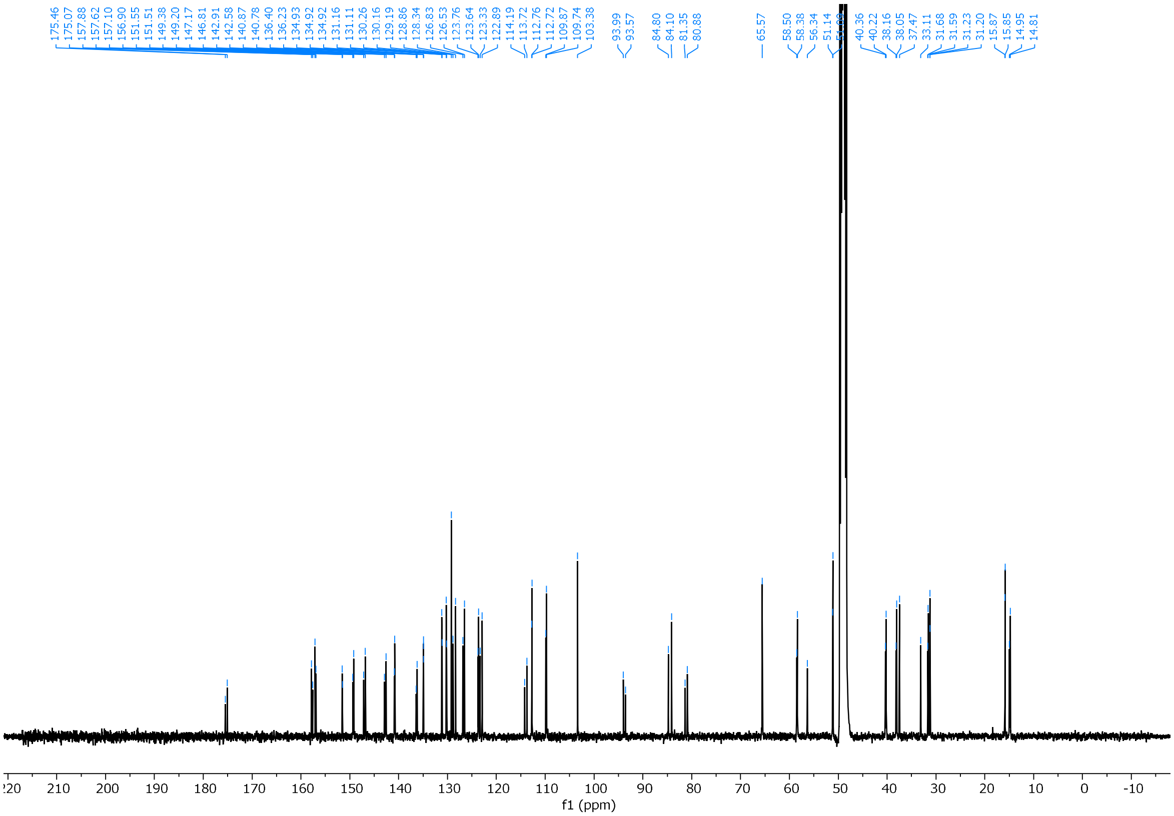

3-(Benzo[*d*][1,3]dioxol-5-yl)-*N*-(((4*R*,5*R*)-8-bromo-2-((*R*)-1-hydroxypropan-2-yl)-4-methyl-1,1-dioxido-2,3,4,5-tetrahydrobenzo[*b*][1,4,5]oxathiazocin-5-yl)methyl)-*N*-methylpropiolamide (**41**)

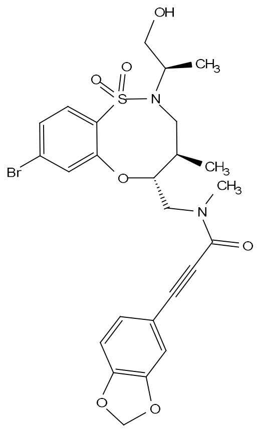
OH
CH3
O
O
S
N
CH3
Br
O
CH3
N
O
O
O

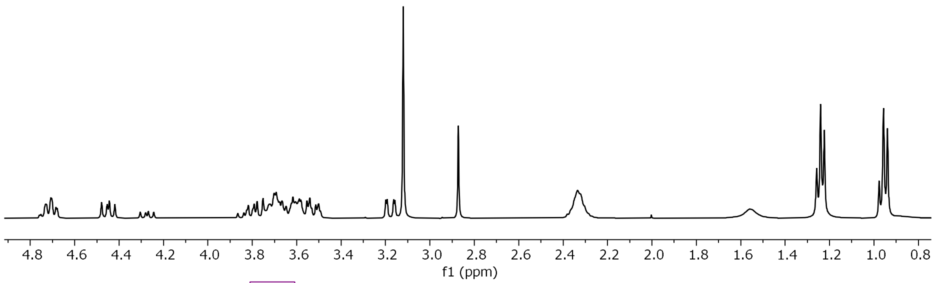

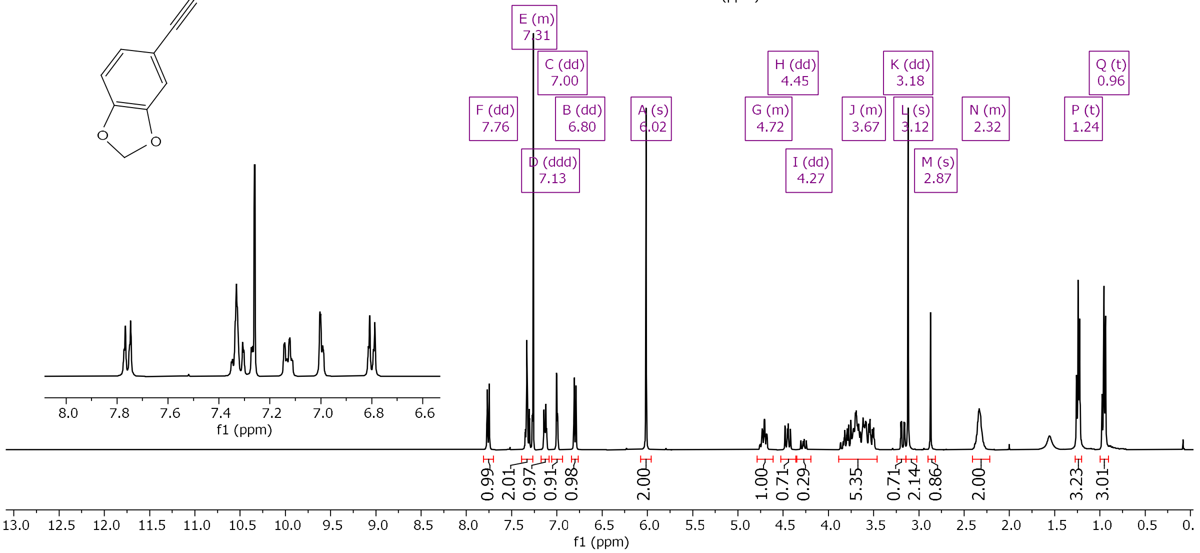
f1 (ppm)
F (dd) 7.76
E (m) 7.31
D (ddd) 7.13
C (dd) 7.00
B (dd) 6.80
A (s) 6.02
G (m) 4.72
H (dd) 4.45
I (dd) 4.27
J (m) 3.67
K (dd) 3.18
L (s) 3.12
M (s) 2.87
N (m) 2.32
P (t) 1.24
Q (t) 0.96
0.99
2.01
0.97
0.91
0.98
2.00
1.00
0.71
0.29
5.35
0.71
2.14
0.86
2.00
3.23
3.01
f1 (ppm)
f1 (ppm)

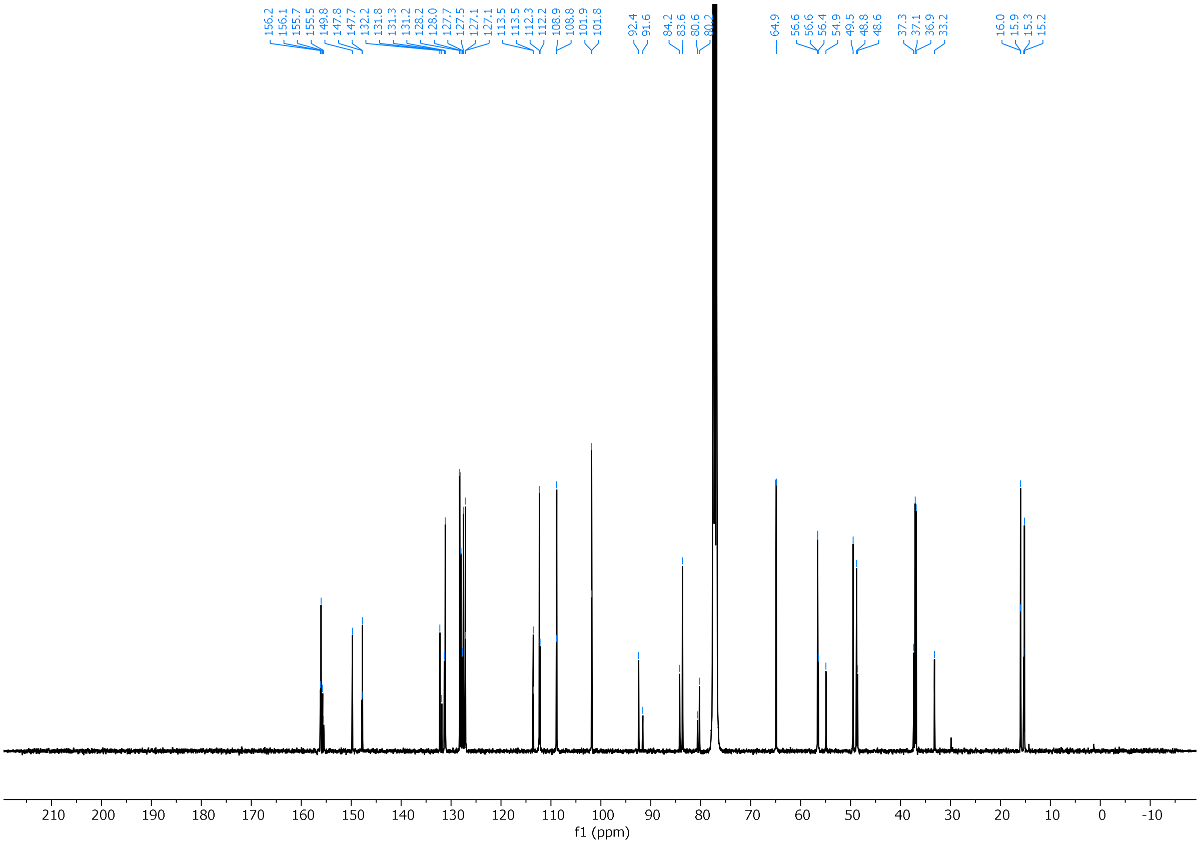
156.2
156.1
155.7
155.5
149.8
147.8
147.7
132.2
131.8
131.3
131.2
128.2
128.0
127.7
127.5
127.1
127.1
113.5
113.5
112.3
112.2
108.9
108.8
101.9
101.8
92.4
91.6
84.2
83.6
80.6
80.2
64.9
56.6
56.6
56.4
54.9
49.5
48.8
48.6
37.3
37.1
36.9
33.2
16.0
15.9
15.3
15.2
f1 (ppm)

3-(Benzo[*d*][1,3]dioxol-5-yl)-*N*-(((4*R*,5*R*)-8-bromo-2-((*S*)-1-hydroxypropan-2-yl)-4-methyl-1,1-dioxido-2,3,4,5-tetrahydrobenzo[*b*][1,4,5]oxathiazocin-5-yl)methyl)-*N*-methylpropiolamide (**42**)

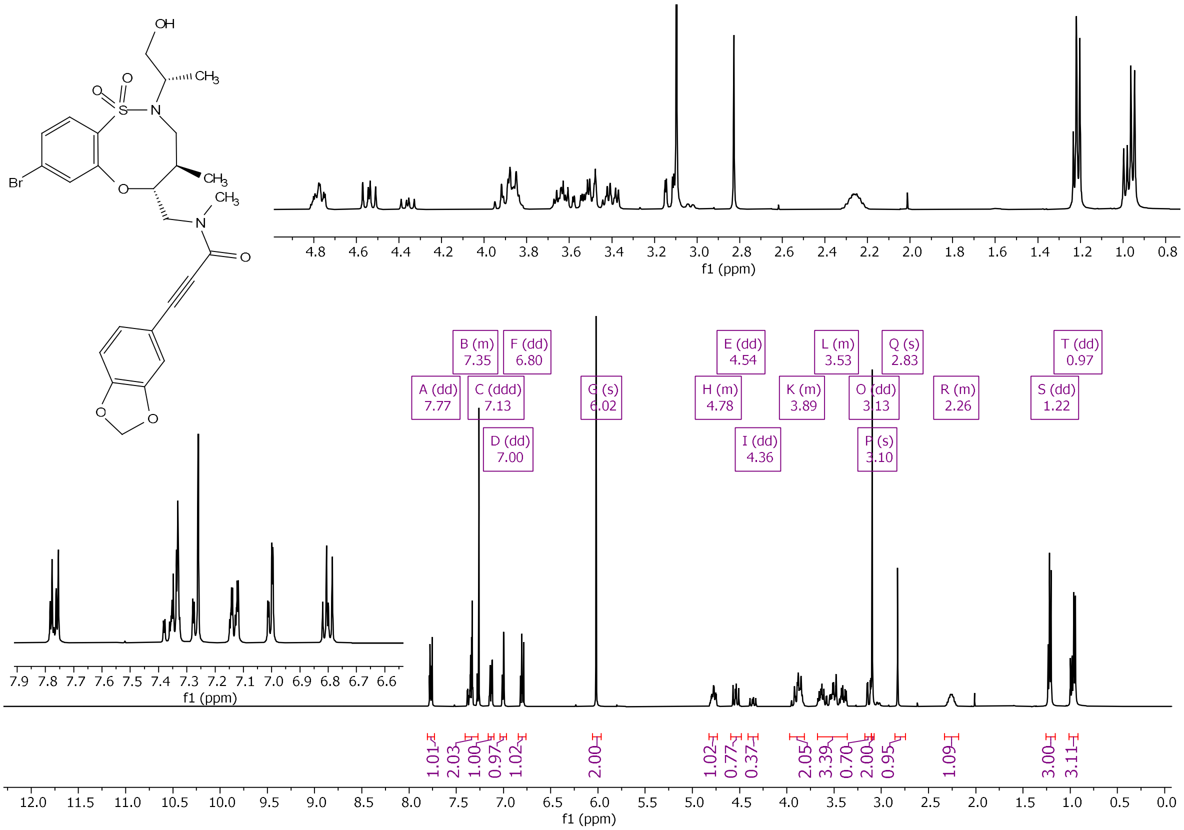

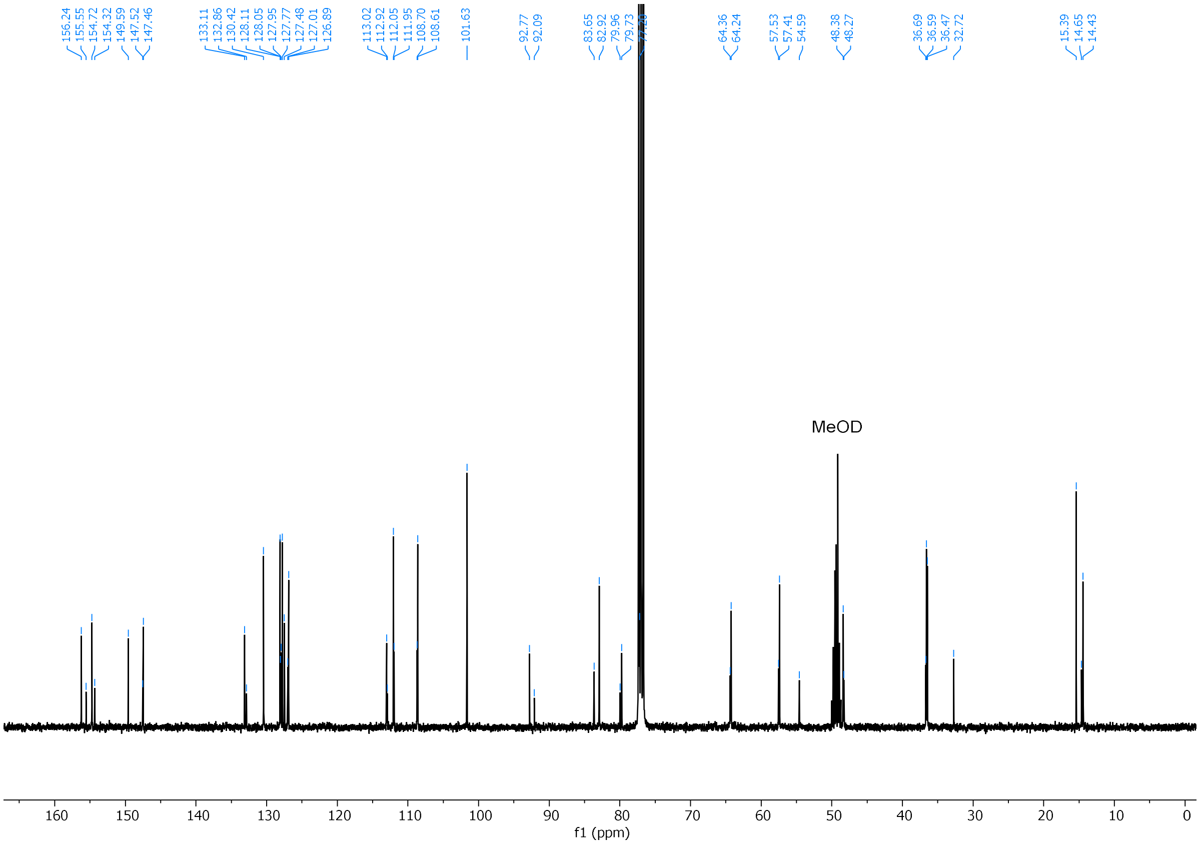

156.24
155.55
154.72
154.32
149.59
147.52
147.46
133.11
132.86
130.42
128.11
128.05
127.95
127.77
127.48
127.01
126.89
113.02
112.92
112.05
111.95
108.70
108.61
101.63
92.77
92.09
83.65
82.92
79.96
79.73
64.36
64.24
57.53
57.41
54.59
48.38
48.27
36.69
36.59
36.47
32.72
15.39
14.65
14.43
MeOD
160 150 140 130 120 110 100 90 80 70 60 50 40 30 20 10 0
f1 (ppm)

3-(Benzo[*d*][1,3]dioxol-5-yl)-1-((8*R*,9*R*,10*S*)-9-(4-bromophenyl)-10-(hydroxymethyl)-1,6-diazabicyclo[6.2.0]decan-6-yl)prop-2-yn-1-one (**43**)

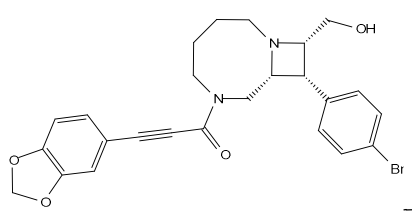

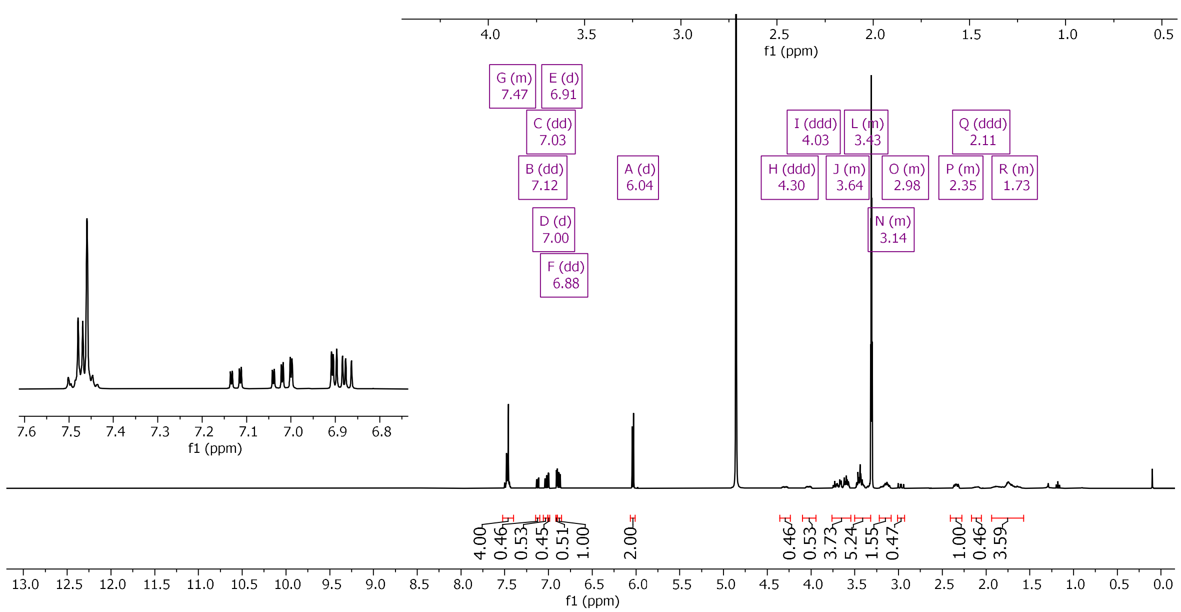

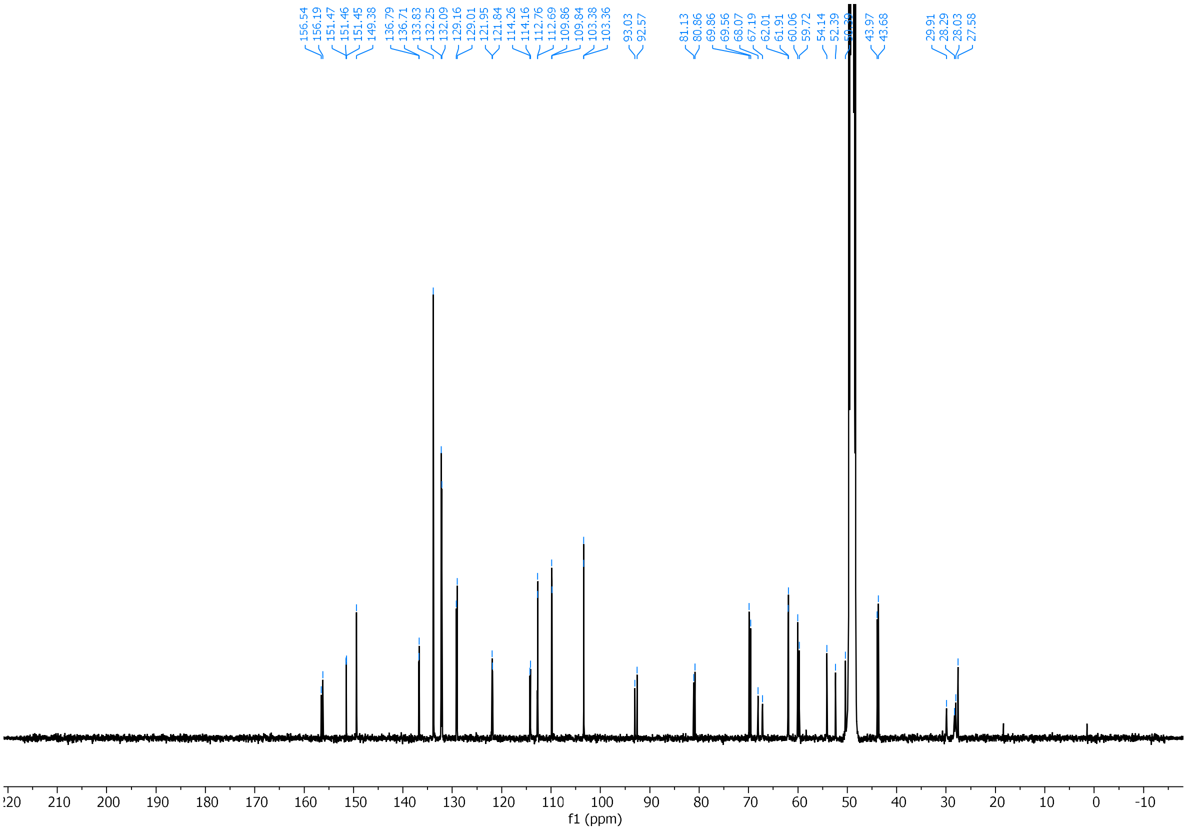

156.54
156.19
151.47
151.46
151.45
149.38
136.79
136.71
133.83
132.25
132.09
129.16
129.01
121.95
121.84
114.26
114.16
112.76
112.69
109.86
109.84
103.38
103.36
93.03
92.57
81.13
80.86
69.86
69.56
68.07
67.19
62.01
61.91
60.06
59.72
54.14
52.39
43.97
43.68
29.91
28.29
28.03
27.58
220 210 200 190 180 170 160 150 140 130 120 110 100 90 80 70 60 50 40 30 20 10 0 -10
f1 (ppm)

3-(Benzo[*d*][1,3]dioxol-5-yl)-1-((8*S*,9*S*,10*S*)-9-(4-bromophenyl)-10-(hydroxymethyl)-1,6-diazabicyclo[6.2.0]decan-6-yl)prop-2-yn-1-one (**44**)

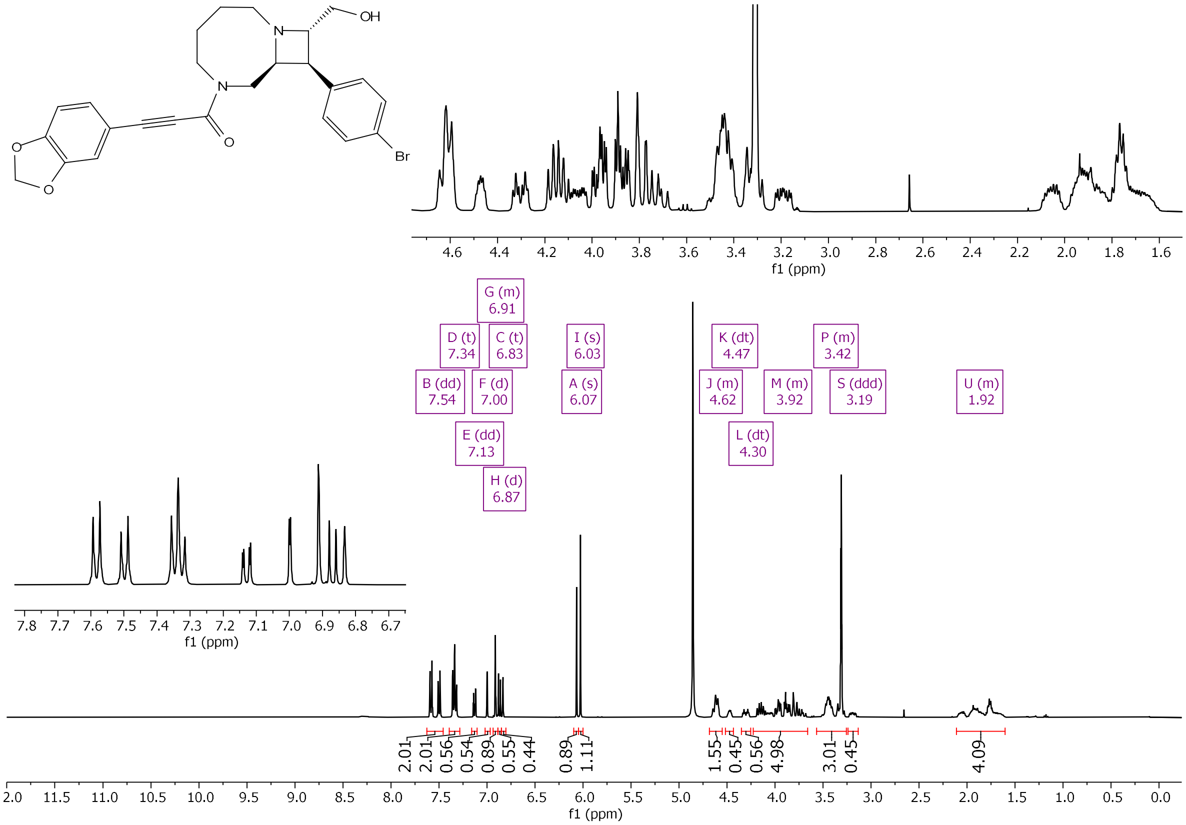

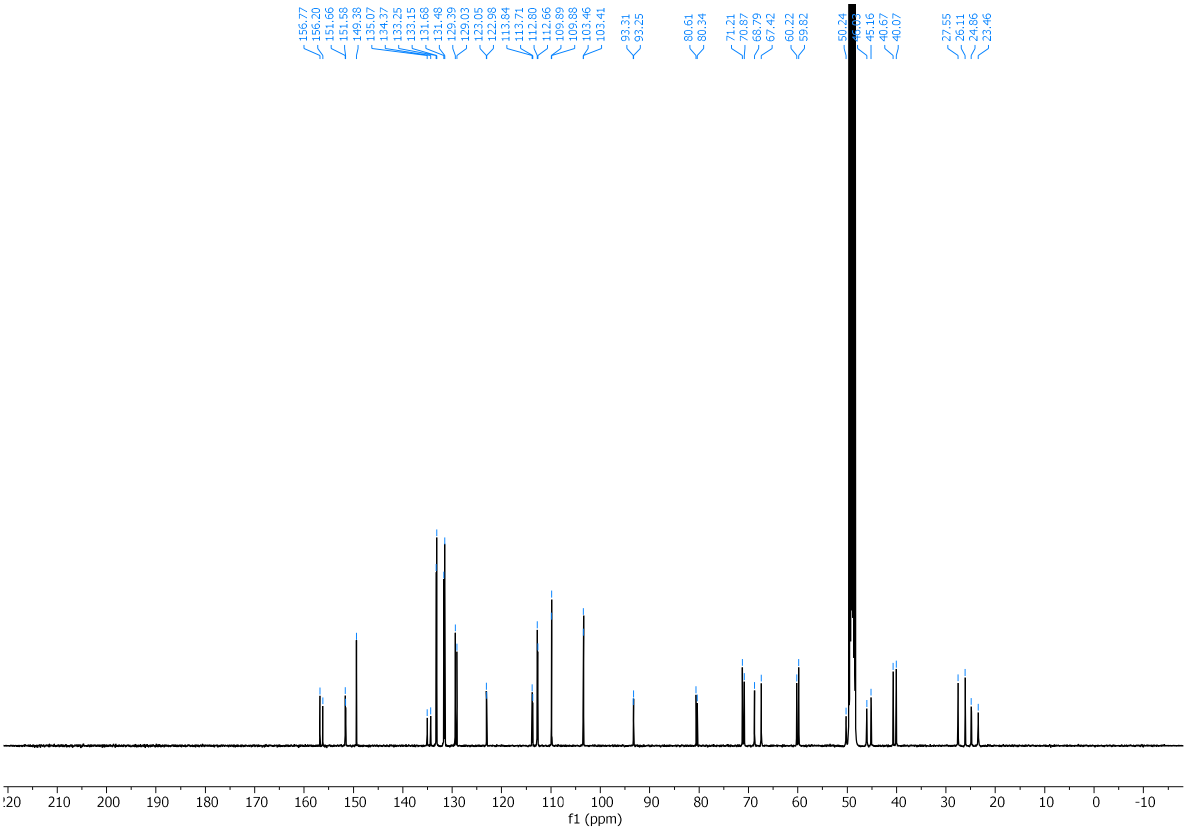

(2*R*,4*R*)-*N*-Methyl-1-((4-sulfamoylphenyl)sulfonyl)-4-(3-(1,2,3,6-tetrahydropyridin-4-yl)phenoxy)pyrrolidine-2-carboxamide (**45**)

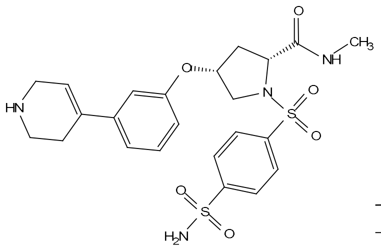
HN
O
O
NH
CH3
N
S
O
O
O
S
O
H2N

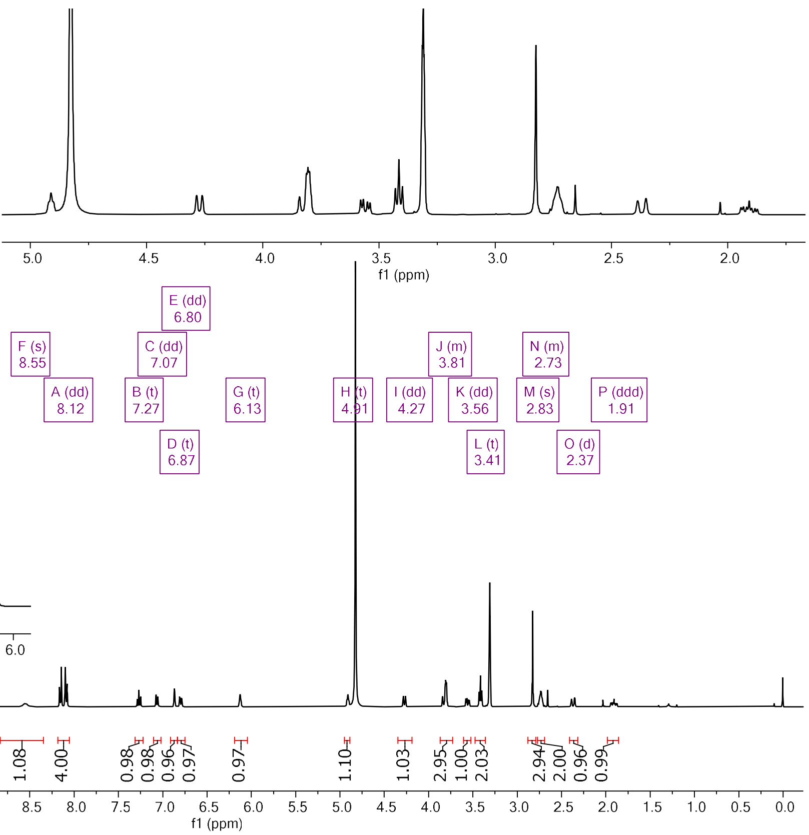
f1 (ppm)
E (dd) 6.80
F (s) 8.55
C (dd) 7.07
J (m) 3.81
N (m) 2.73
A (dd) 8.12
B (t) 7.27
G (t) 6.13
H (t) 4.91
I (dd) 4.27
K (dd) 3.56
M (s) 2.83
P (ddd) 1.91
D (t) 6.87
L (t) 3.41
O (d) 2.37
1.08
4.00
0.98
0.98
0.96
0.97
0.97
1.10
1.03
2.95
1.00
2.03
2.94
2.00
0.96
0.99
f1 (ppm)
f1 (ppm)

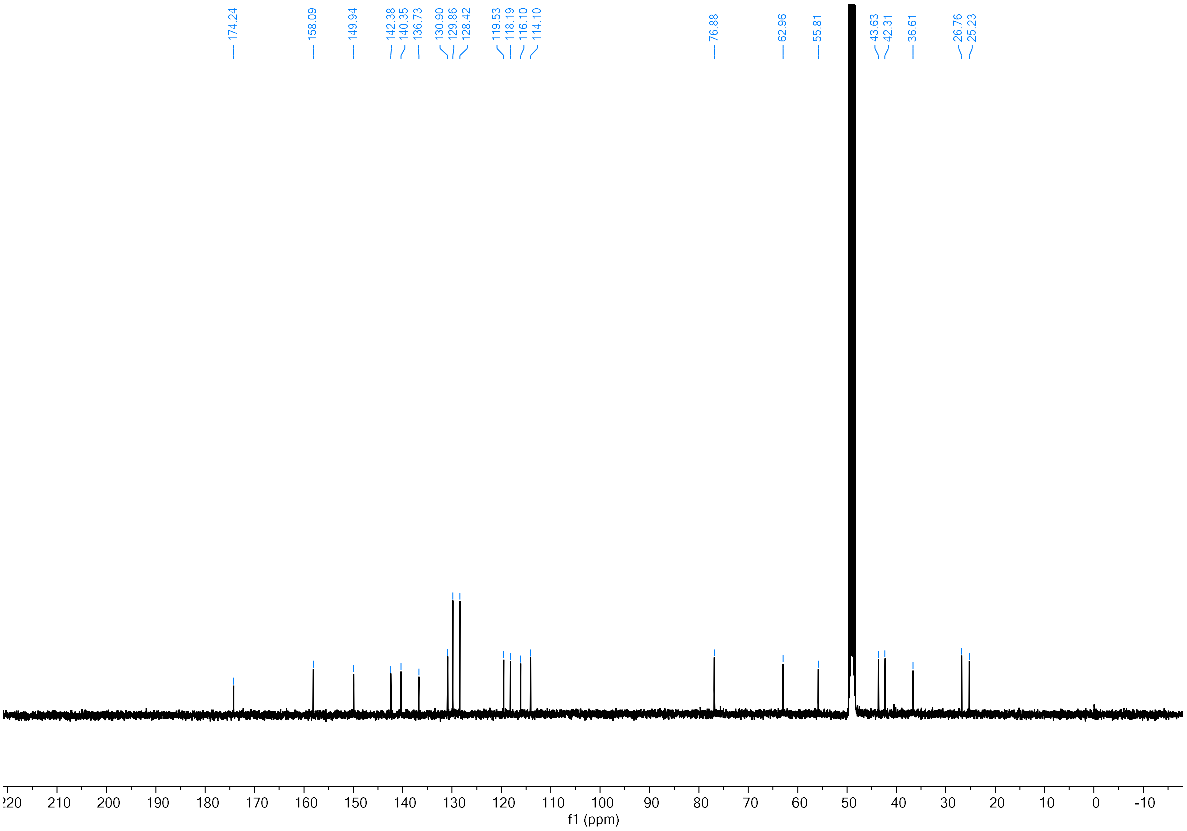
174.24
158.09
149.94
142.38
140.35
136.73
130.90
129.86
128.42
119.53
118.19
116.10
114.10
76.88
62.96
55.81
43.63
42.31
36.61
26.76
25.23
f1 (ppm)

(*R*)-5-((2-acetyl-8-(3'-((dimethylamino)methyl)-4'-fluoro-[1,1'-biphenyl]-4-yl)-2,6-diazaspiro[3.4]octan-6-yl)methyl)furan-2-sulfonamide (**46**)

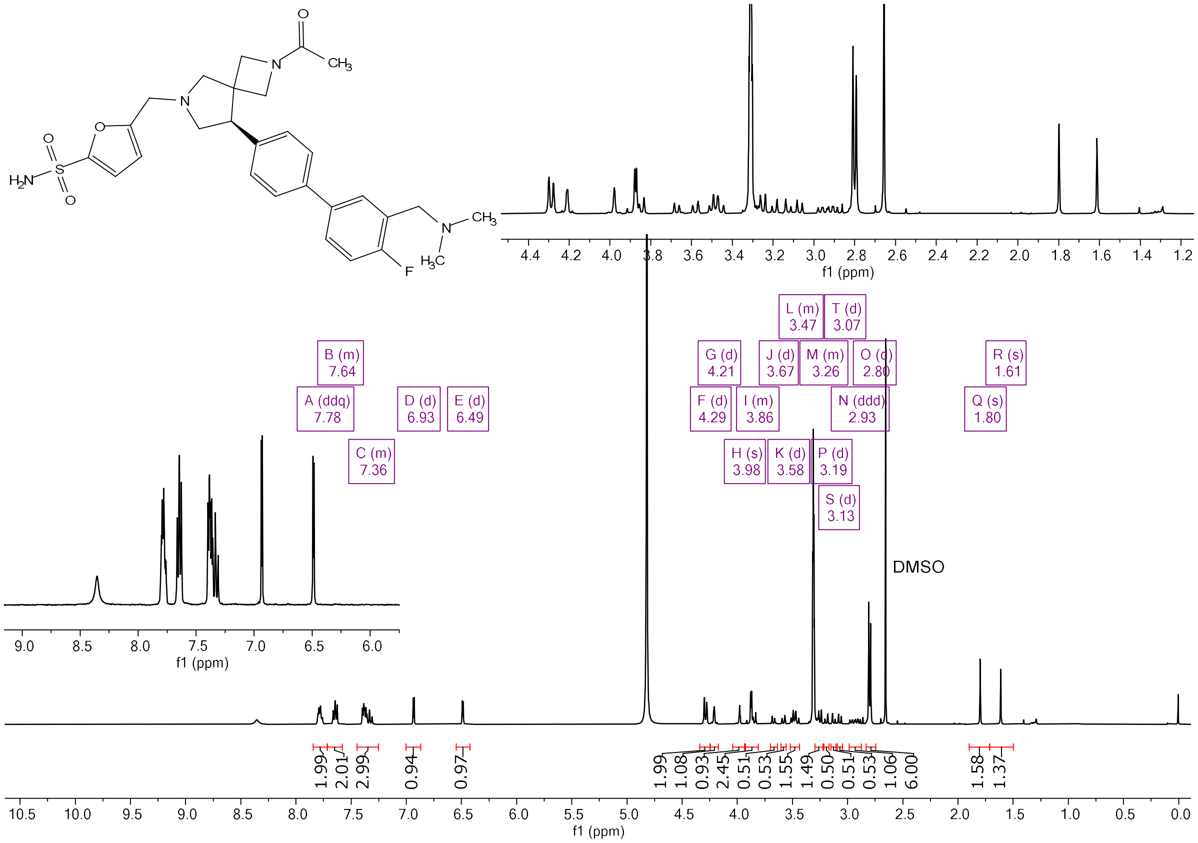

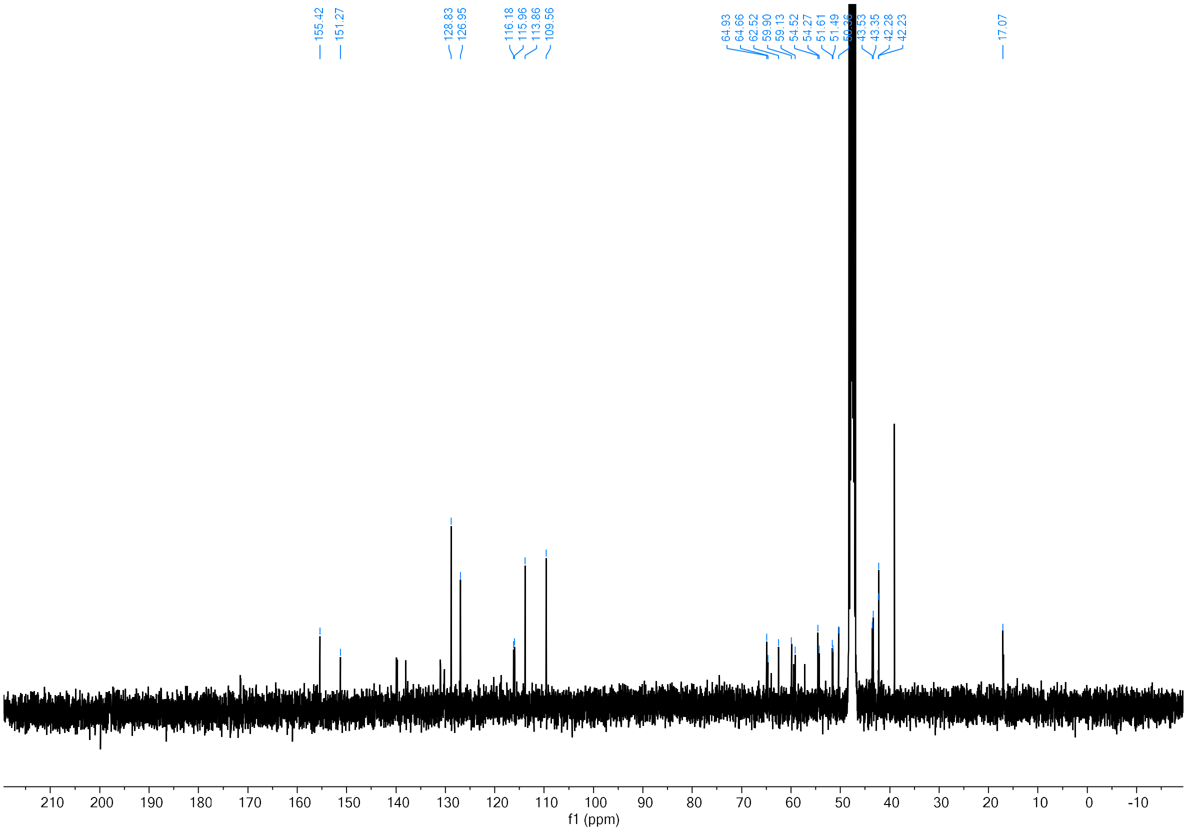

155.42
151.27
128.83
126.95
116.18
115.96
113.86
109.56
64.93
64.66
62.52
59.90
59.13
54.52
54.27
51.61
51.49
50.26
43.53
43.35
42.28
42.23
17.07
210 200 190 180 170 160 150 140 130 120 110 100 90 80 70 60 50 40 30 20 10 0 -10
f1 (ppm)

(2*S*,3*S*)-3-(3'-(ethylcarbamoyl)-[1,1'-biphenyl]-4-yl)-*N*-methyl-1-(4-sulfamoylbenzyl)pyrrolidine-2-carboxamide (**47**)

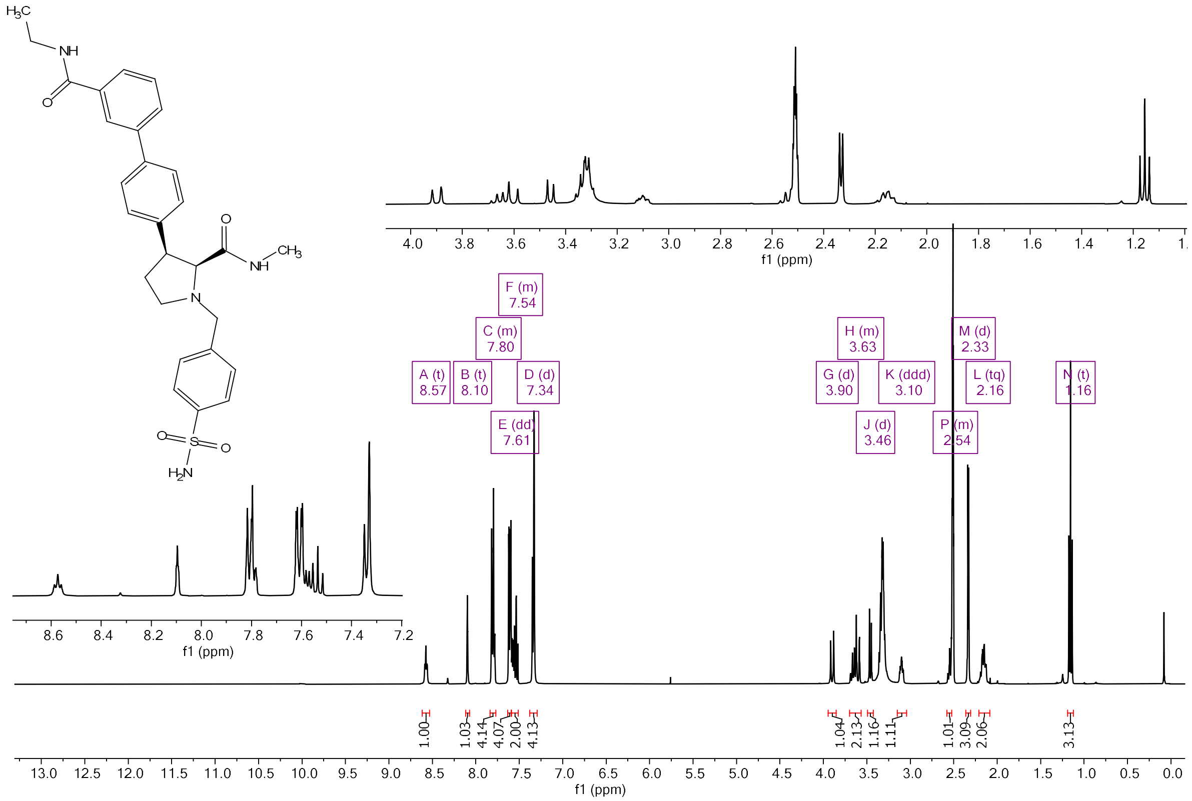

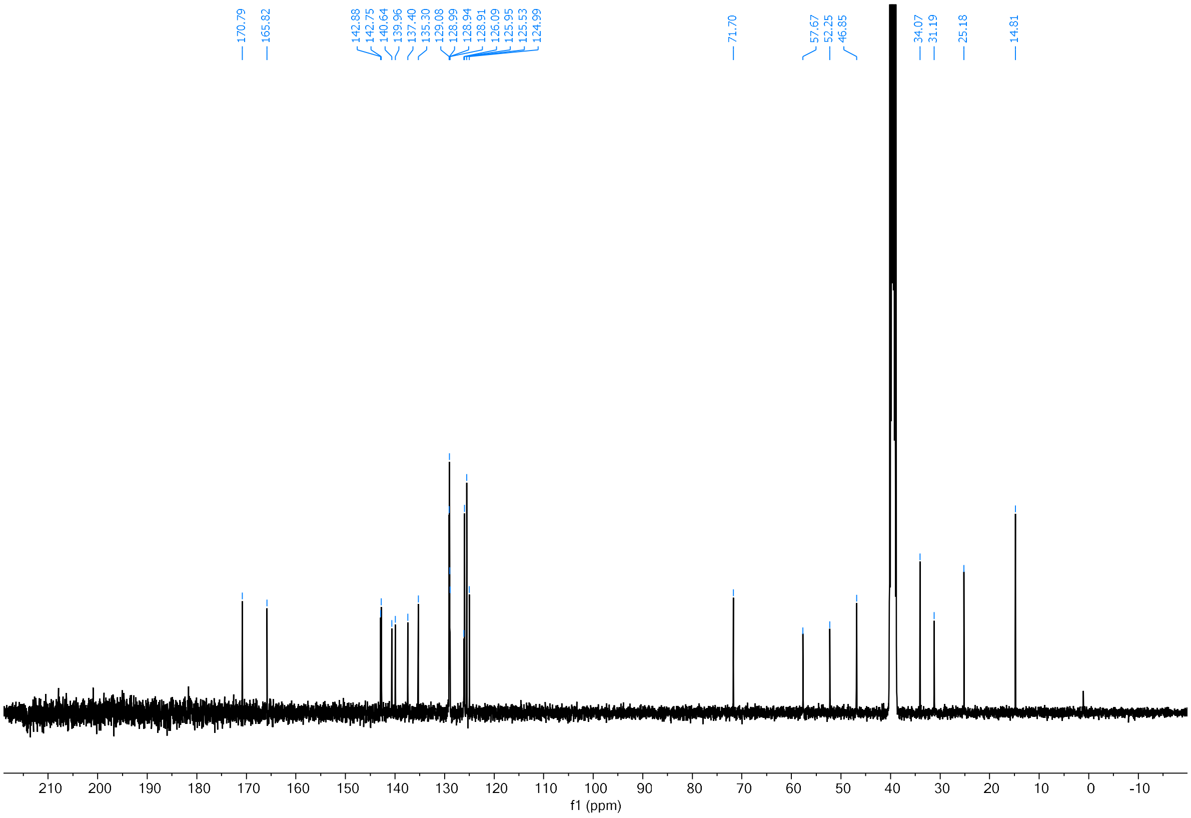

(2*R*,3*S*)-3-(3'-ethoxy-[1,1'-biphenyl]-3-yl)-*N*-methyl-1-((5-sulfamoylfuran-3-yl)methyl)pyrrolidine-2-carboxamide (**48**)

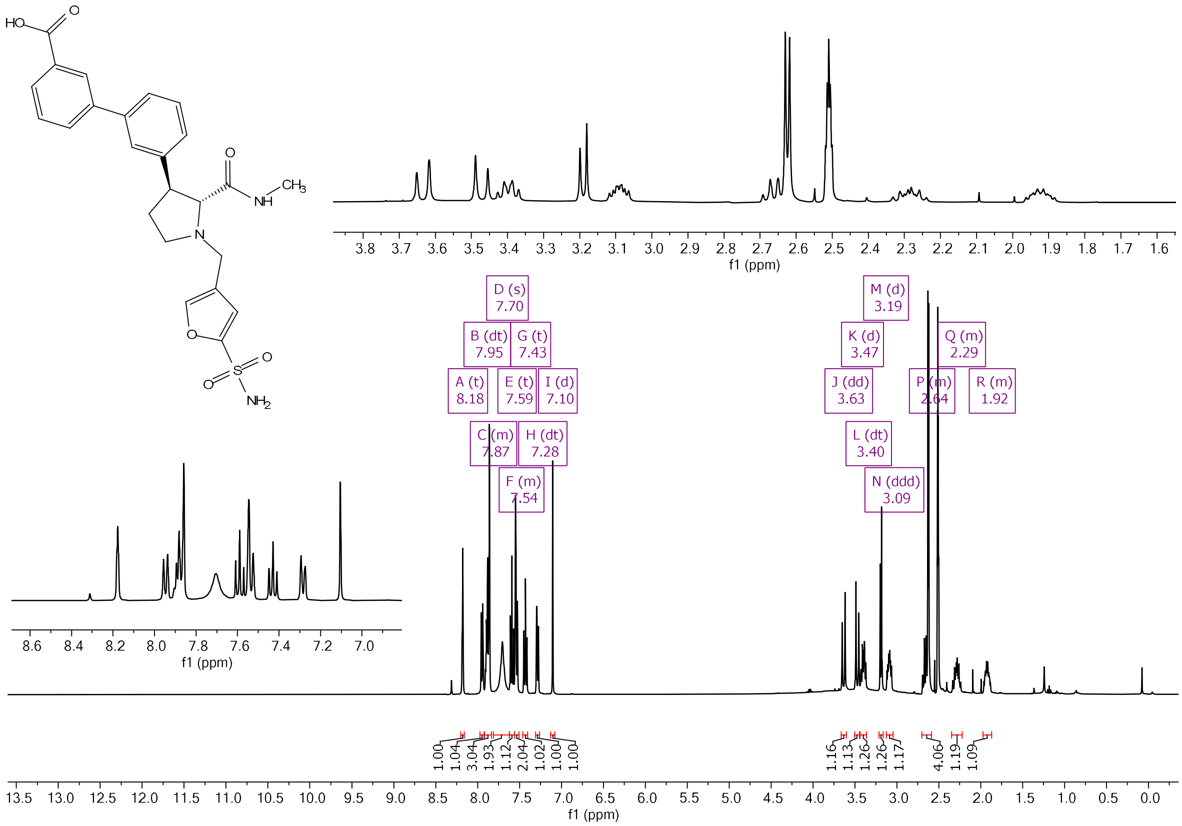

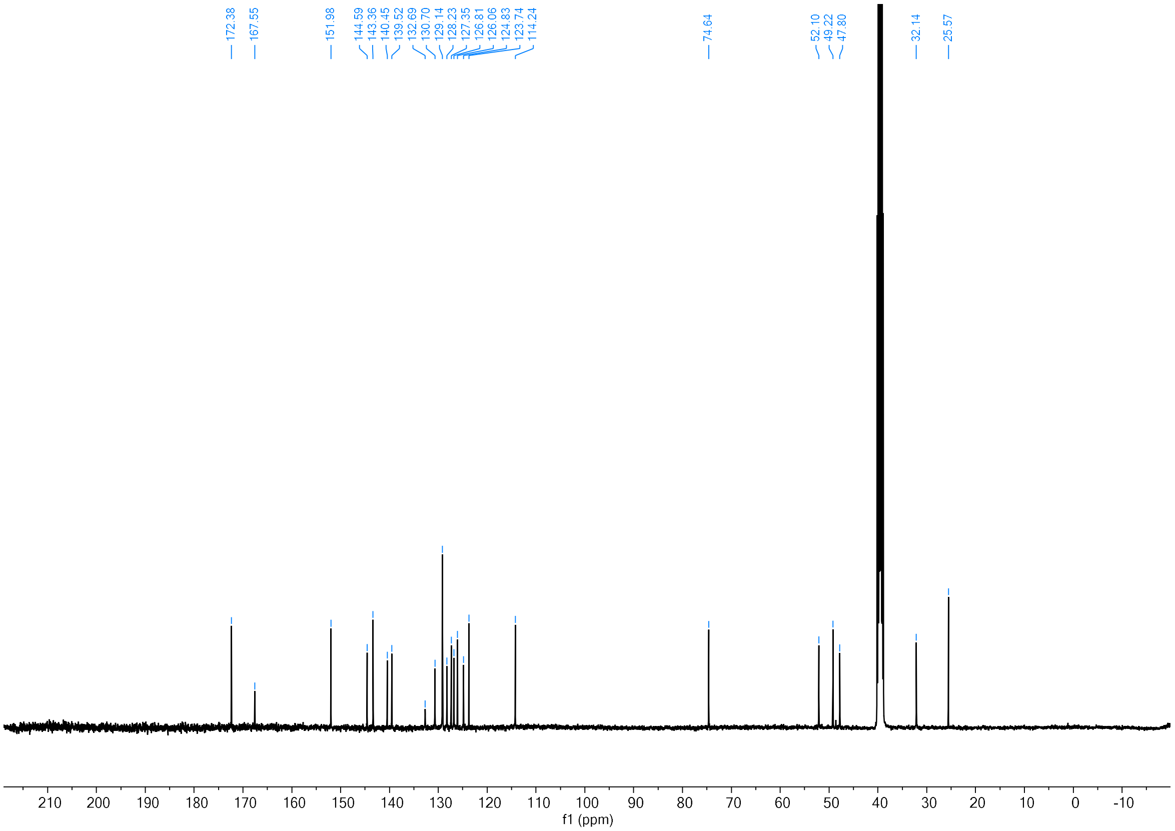

172.38
167.55
151.98
144.59
143.36
140.45
139.52
132.69
130.70
129.14
128.23
127.35
126.81
126.06
124.83
123.74
114.24
74.64
52.10
49.22
47.80
32.14
25.57
210 200 190 180 170 160 150 140 130 120 110 100 90 80 70 60 50 40 30 20 10 0 -10
f1 (ppm)

(2*R*,3*S*)-3-(3'-ethoxy-[1,1'-biphenyl]-4-yl)-*N*-methyl-1-((5-sulfamoylfuran-3-yl)methyl)pyrrolidine-2-carboxamide (**49**)

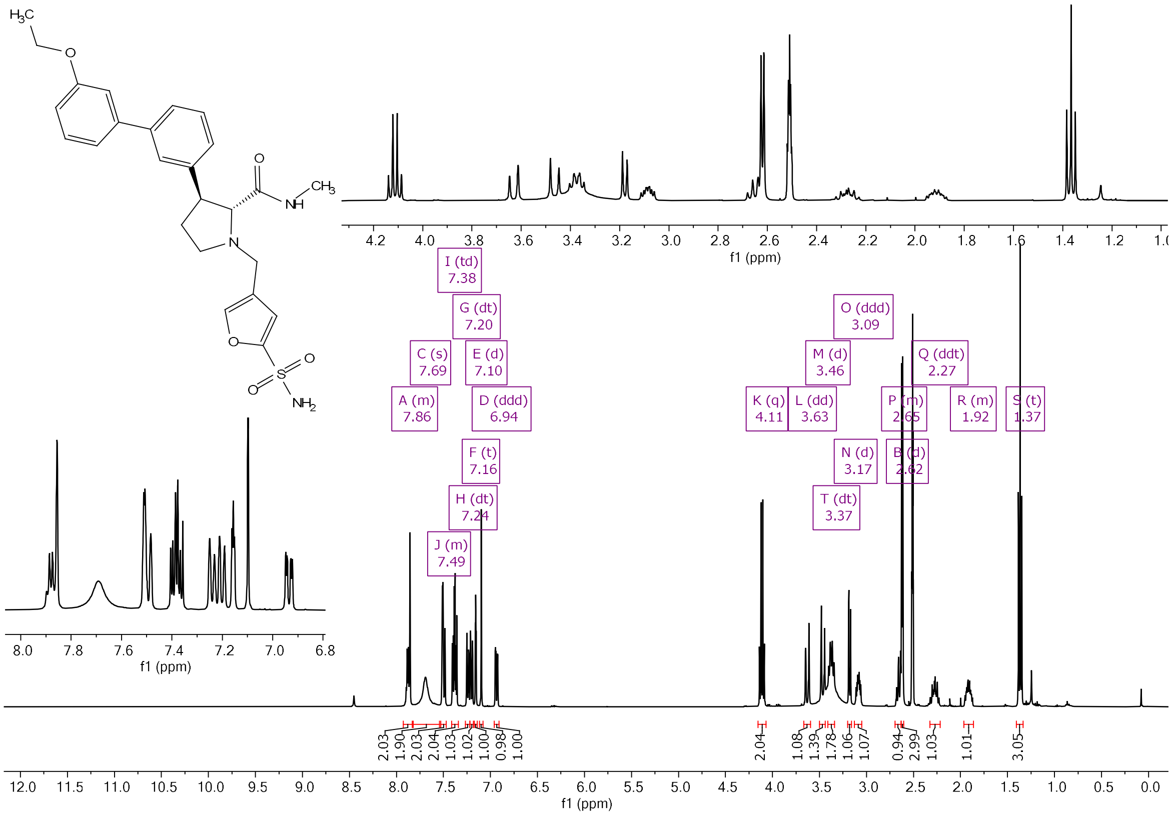

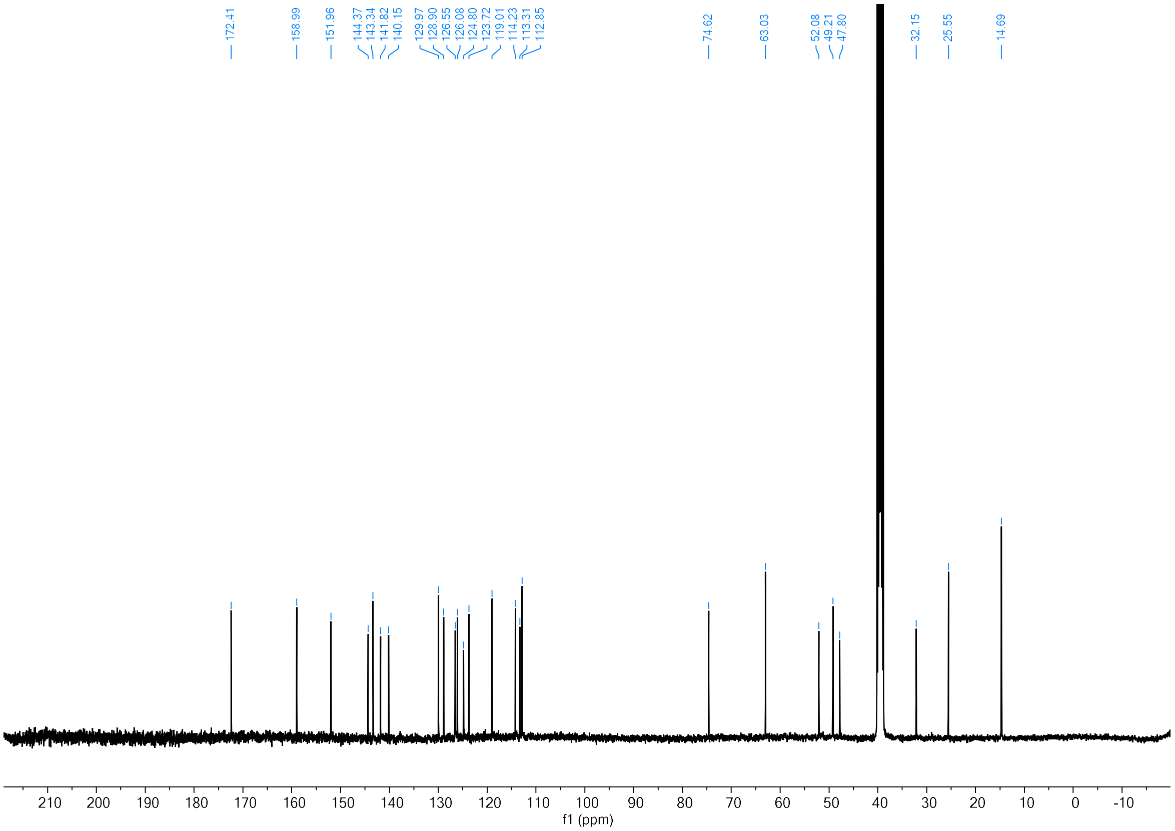
172.41
158.99
151.96
144.37
143.34
141.82
140.15
129.97
128.90
126.55
126.08
124.80
123.72
119.01
114.23
113.31
112.85
74.62
63.03
52.08
49.21
47.80
32.15
25.55
14.69
210 200 190 180 170 160 150 140 130 120 110 100 90 80 70 60 50 40 30 20 10 0 -10
f1 (ppm)

(2*S*,3*R*)-3-(3'-ethoxy-[1,1'-biphenyl]-4-yl)-*N*-methyl-1-((5-sulfamoylfuran-3-yl)methyl)azetidine-2-carboxamide (**50**)

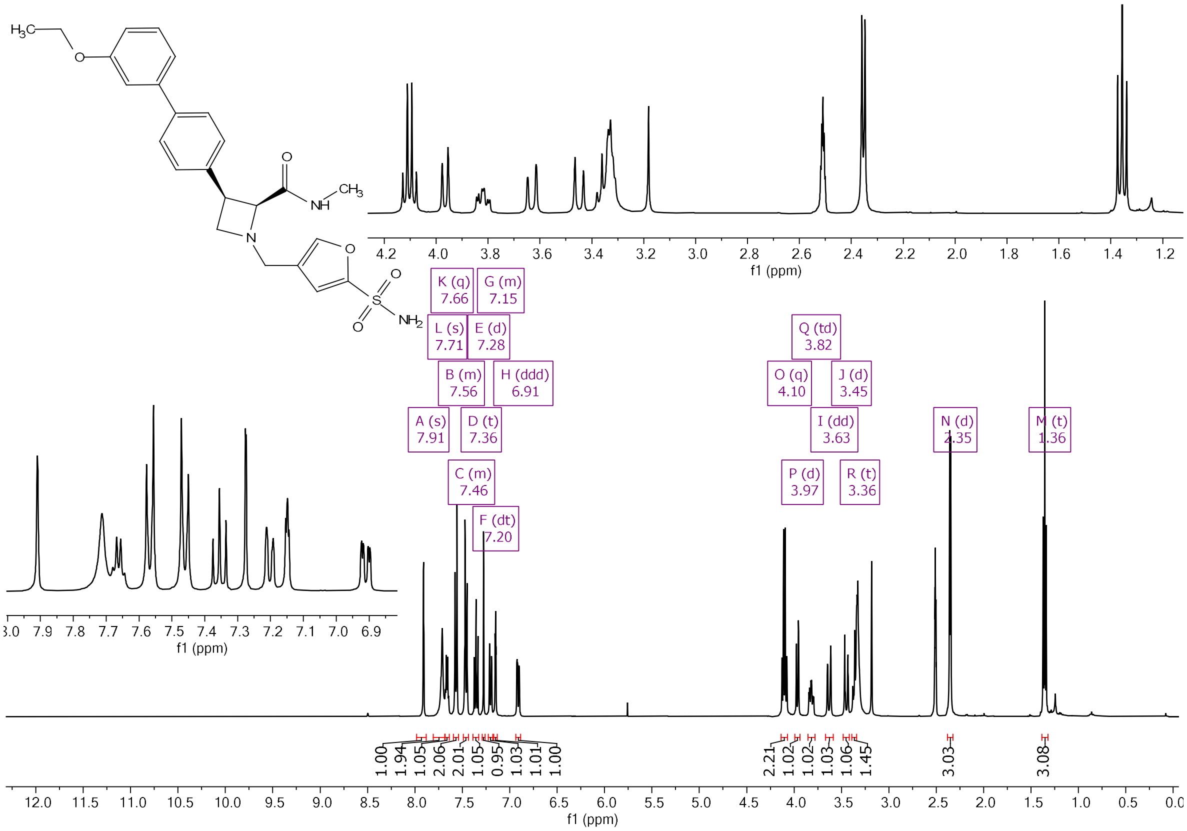

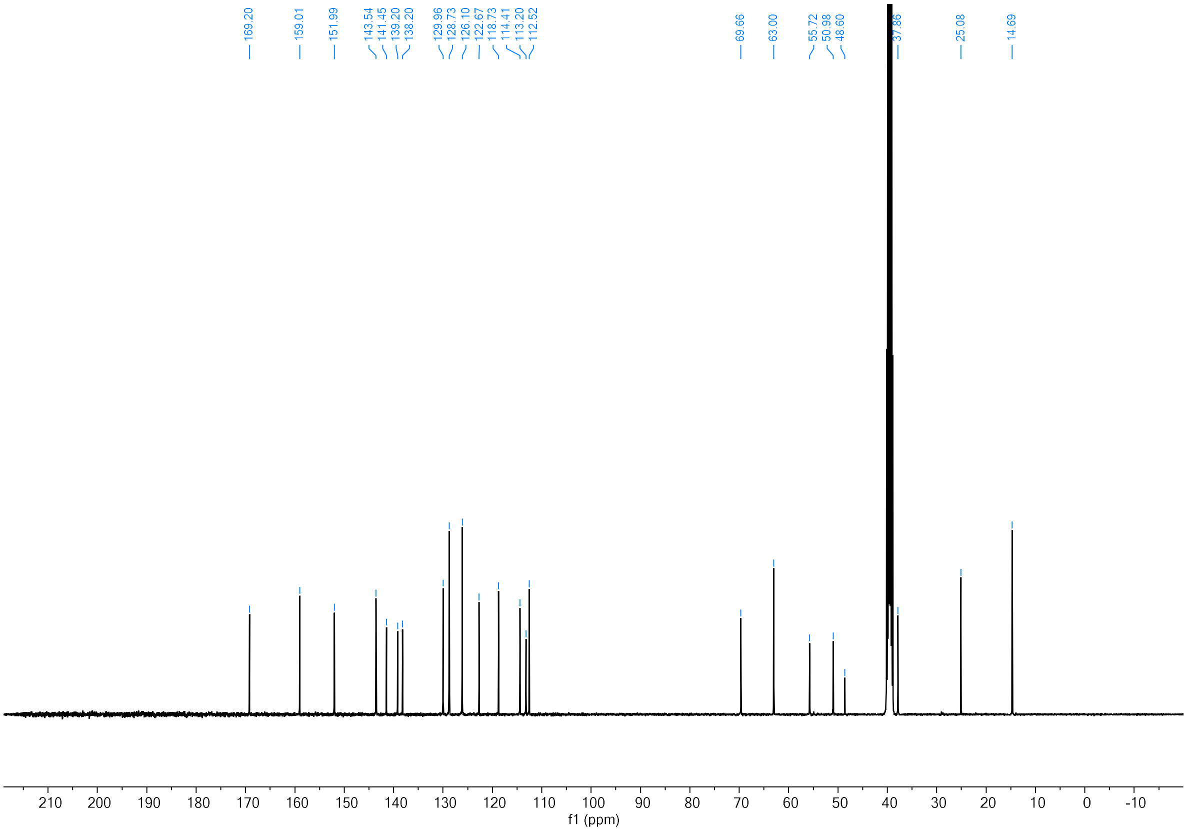
169.20
159.01
151.99
143.54
141.45
139.20
138.20
129.96
128.73
126.10
122.67
118.73
114.41
113.20
112.52
69.66
63.00
55.72
50.98
48.60
37.86
25.08
14.69
210
200
190
180
170
160
150
140
130
120
110
100
90
80
70
60
50
40
30
20
10
0
-10
f1 (ppm)

(2*S*,3*S*)-3-(5'-chloro-2'-methyl-[1,1'-biphenyl]-3-yl)-*N*-methyl-1-((5-sulfamoylfuran-3-yl)methyl)piperidine-2-carboxamide (**51**)

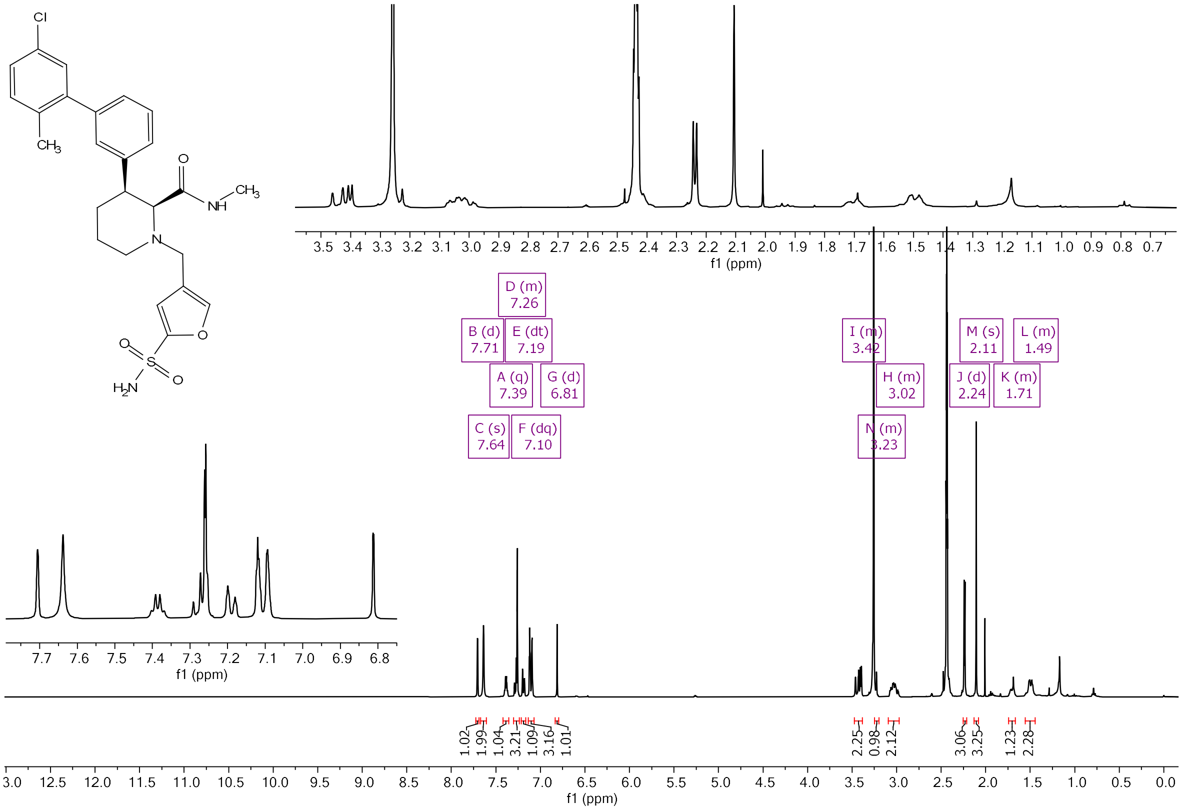

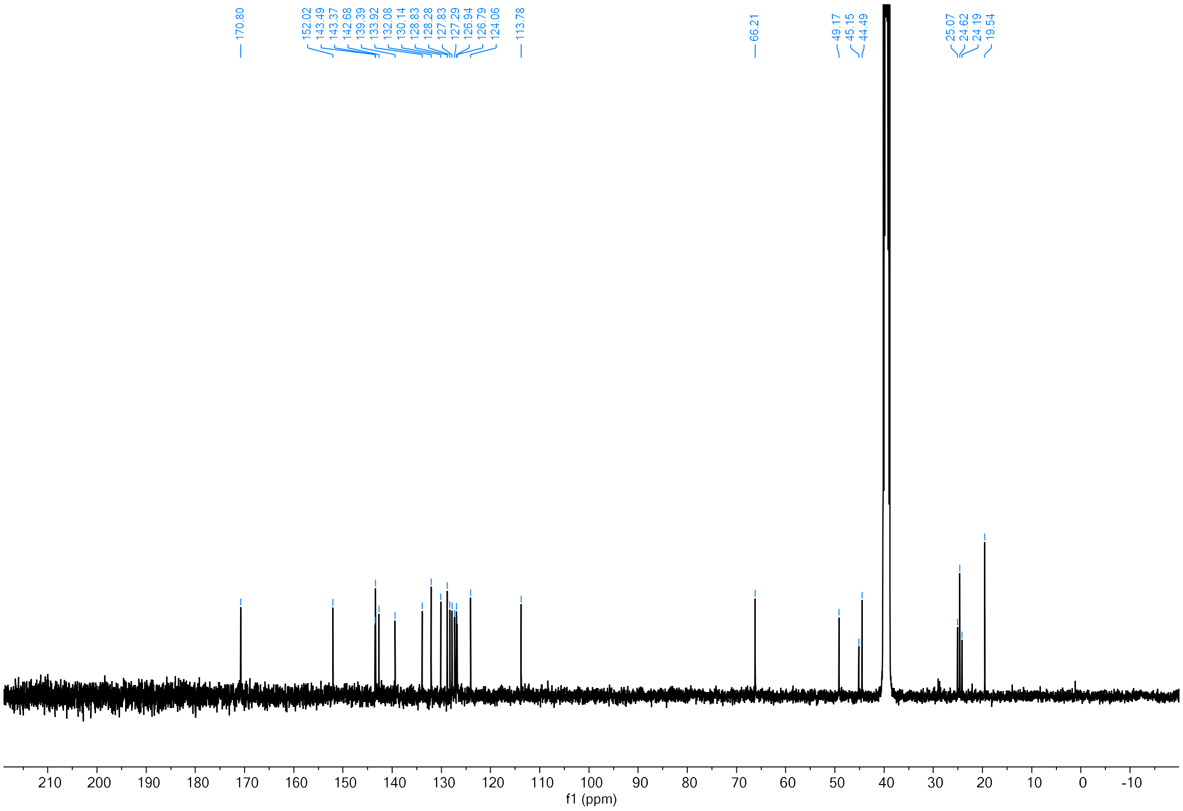

(2*R*,4*S*)-1-((6,7-dihydro-5H-pyrrolo[1,2-a]imidazol-2-yl)methyl)-*N*-methyl-4-((4'-sulfamoyl-[1,1'-biphenyl]-3-yl)oxy)pyrrolidine-2-carboxamide (**52**)

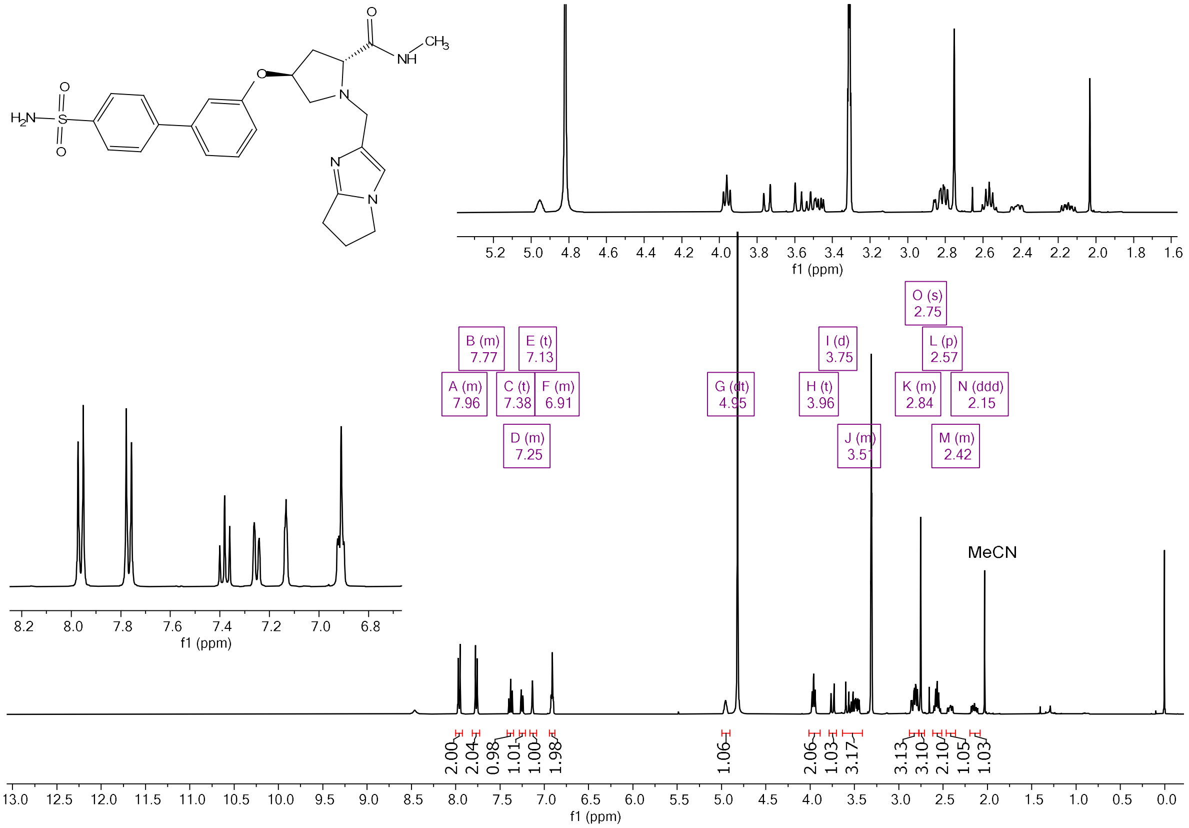

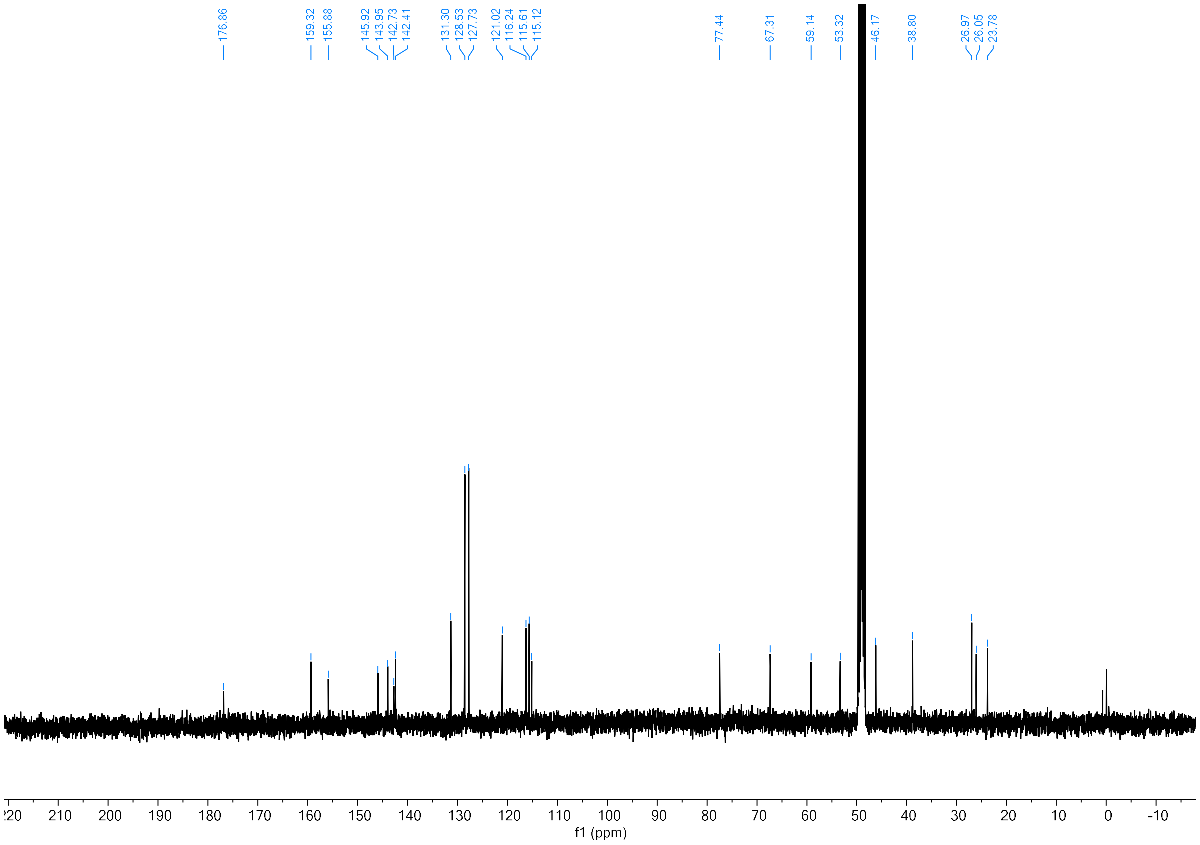
176.86
159.32
155.88
145.92
143.95
142.73
142.41
131.30
128.53
127.73
121.02
116.24
115.61
115.12
77.44
67.31
59.14
53.32
46.17
38.80
26.97
26.05
23.78
220 210 200 190 180 170 160 150 140 130 120 110 100 90 80 70 60 50 40 30 20 10 0 -10
f1 (ppm)

(*R*)-4'-(6-acetyl-2-(3-methyl-5,6,7,8-tetrahydroimidazo[1,5-*a*]pyridine-1-carbonyl)-2,6-diazaspiro[3.4]octan-8-yl)-[1,1'-biphenyl]-3-sulfonamide (**53**)

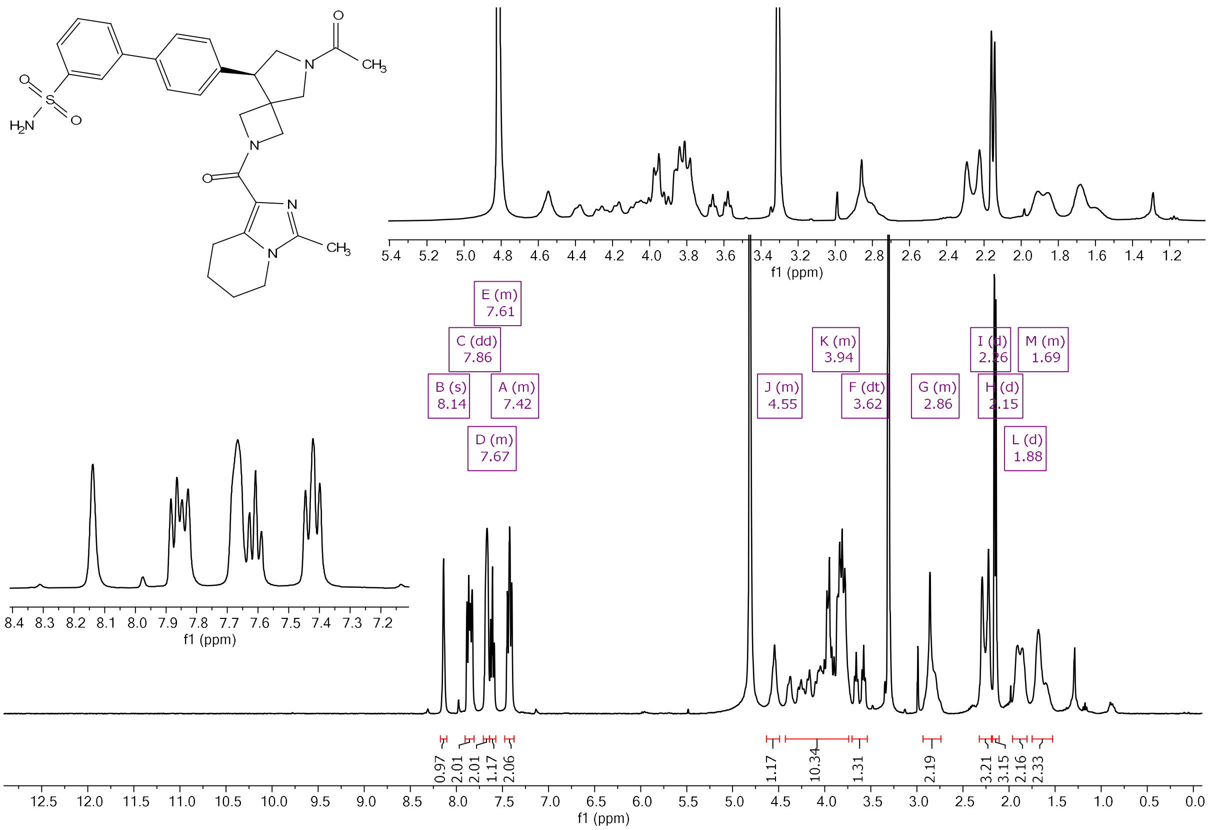

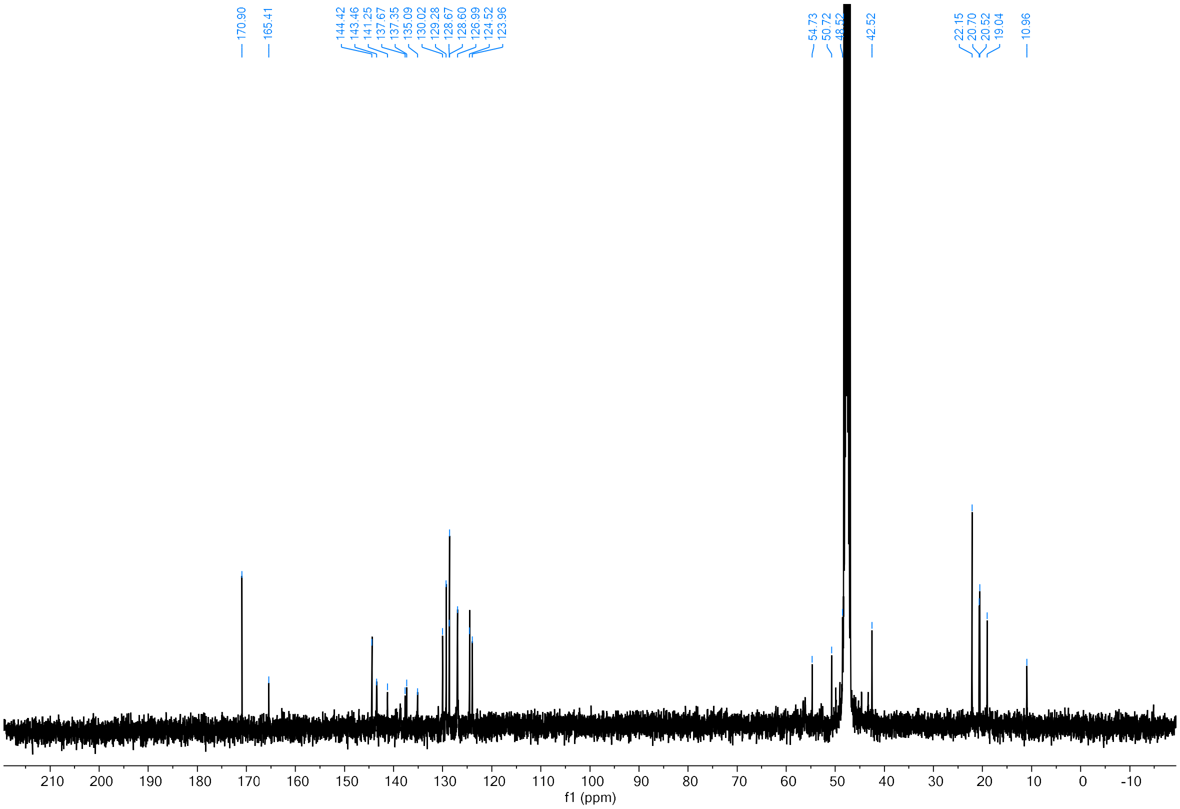
170.90
165.41
144.42
143.46
141.25
137.67
137.35
135.09
130.02
129.28
128.67
128.60
126.99
124.52
123.96
54.73
50.72
48.52
42.52
22.15
20.70
20.52
19.04
10.96
210 200 190 180 170 160 150 140 130 120 110 100 90 80 70 60 50 40 30 20 10 0 -10
f1 (ppm)

4'-((8*S*)-6-Acetyl-2-(2-(3-cyanophenoxy)propanoyl)-2,6-diazaspiro[3.4]octan-8-yl)-[1,1'-biphenyl]-4-sulfonamide (**54**)

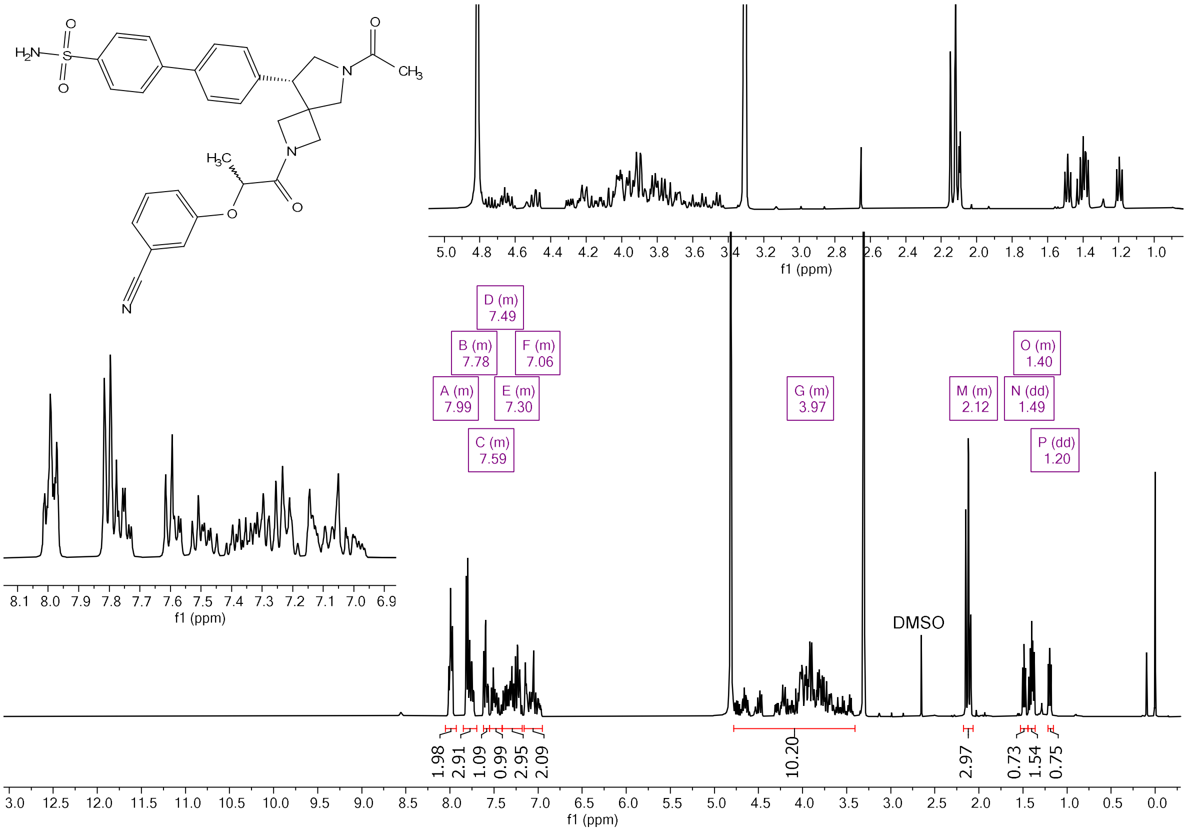

3-(3'-((2S,3R)-2-(Methylcarbamoyl)-1-(4-sulfamoylbenzoyl)pyrrolidin-3-yl)-[1,1'-biphenyl]-3-yl)propanoic acid (**55**)

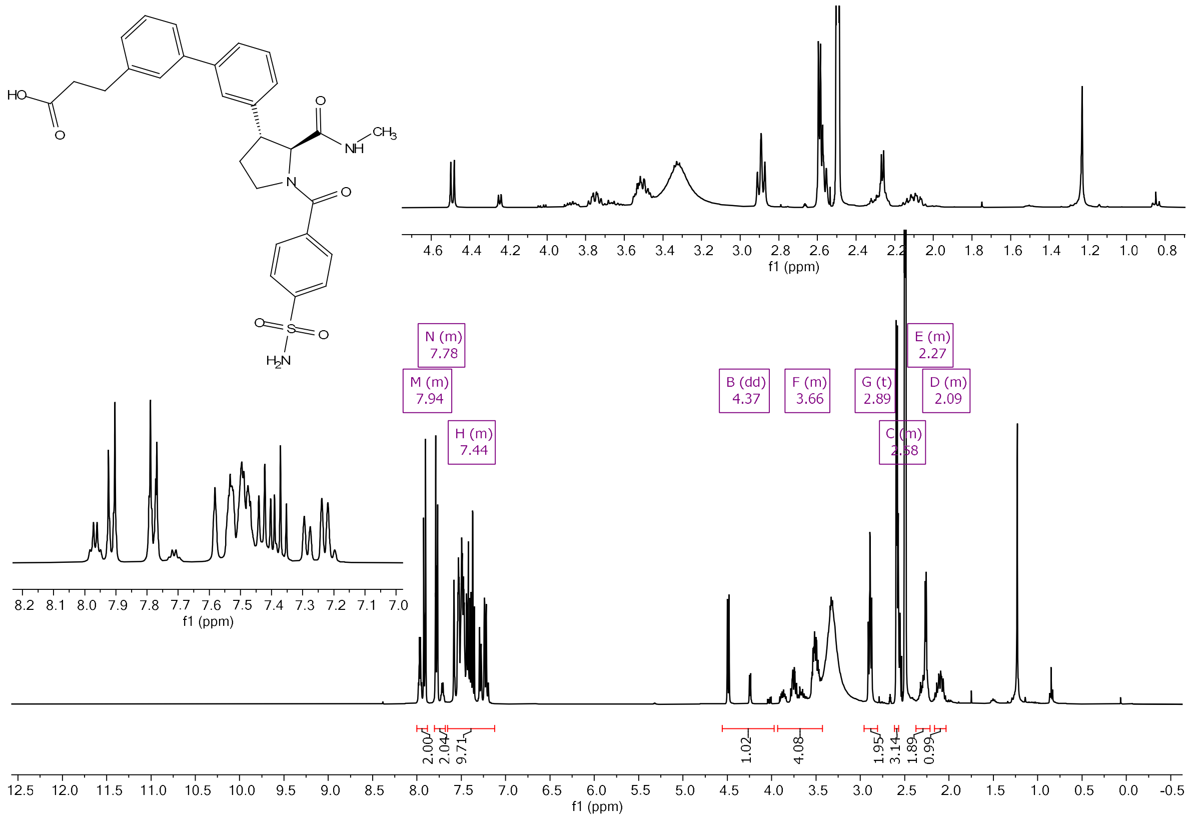

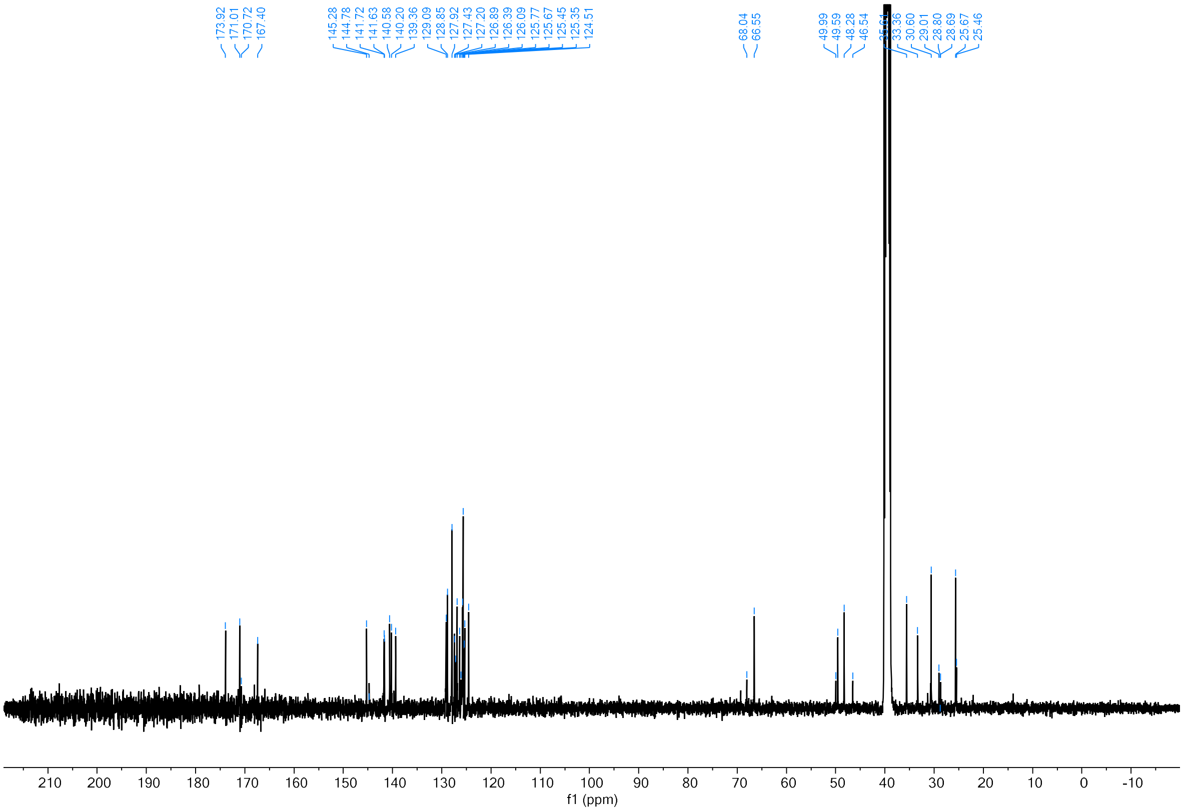

N-(((4S,5R)-2-((R)-1-hydroxypropan-2-yl)-4-methyl-1,1-dioxido-8-(3-sulfamoylphenyl)-2,3,4,5-tetrahydrobenzo[b][1,4,5]oxathiazocin-5-yl)methyl)-N,4-dimethylbenzenesulfonamide (**56**)

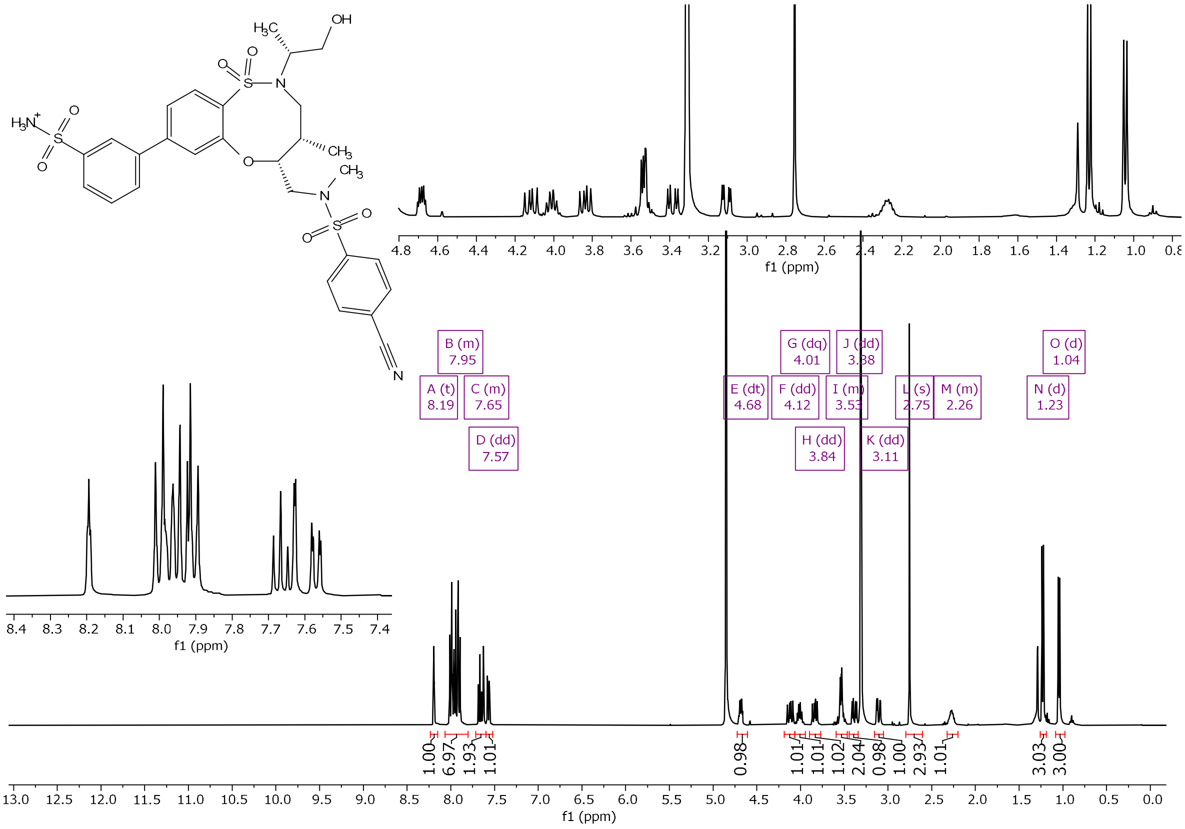

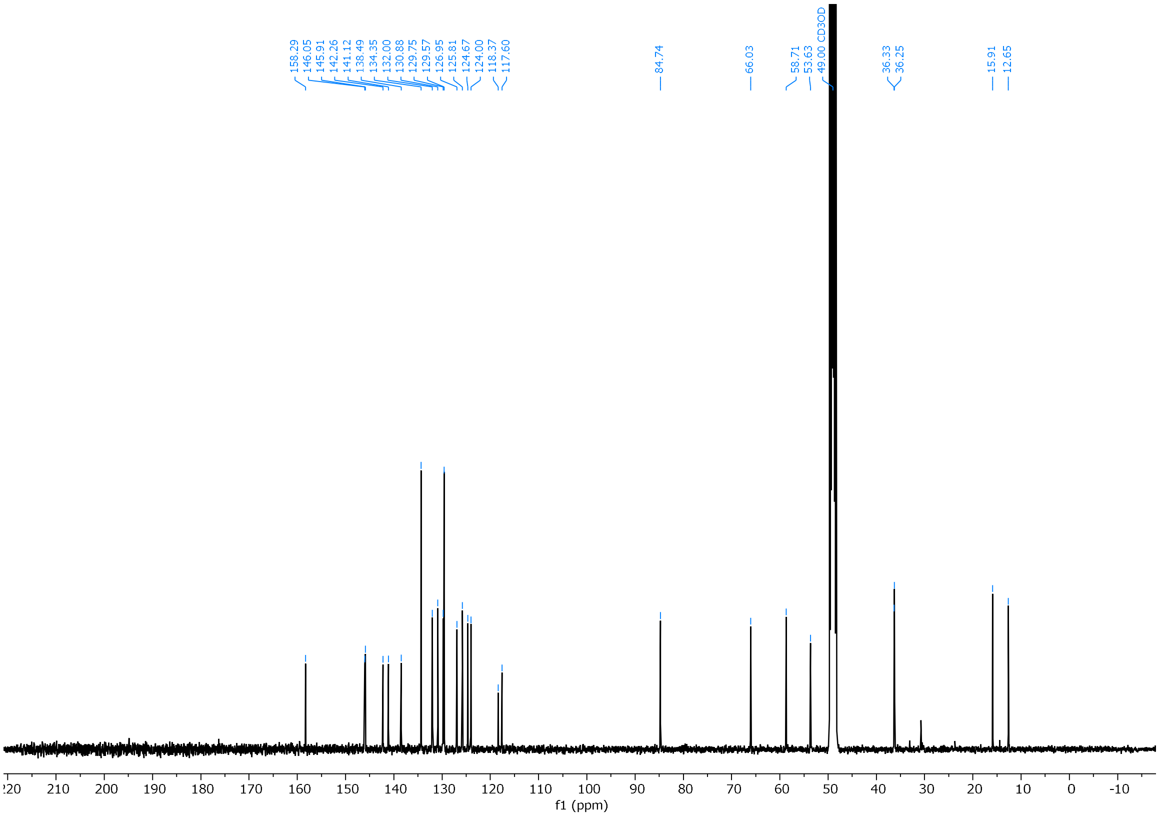
158.29
146.05
145.91
142.26
141.12
138.49
134.35
132.00
130.88
129.75
129.57
126.95
125.81
124.67
124.00
118.37
117.60
84.74
66.03
58.71
53.63
49.00 CD3OD
36.33
36.25
15.91
12.65
f1 (ppm)

5-(Furan-2-yl)-*N*-(((4*S*,5*R*)-2-((*S*)-1-hydroxypropan-2-yl)-4-methyl-1,1-dioxido-8-(4-sulfamoylphenyl)-2,3,4,5-tetrahydrobenzo[*b*][1,4,5]oxathiazocin-5-yl)methyl)-*N*-methylisoxazole-4-carboxamide (**57**)

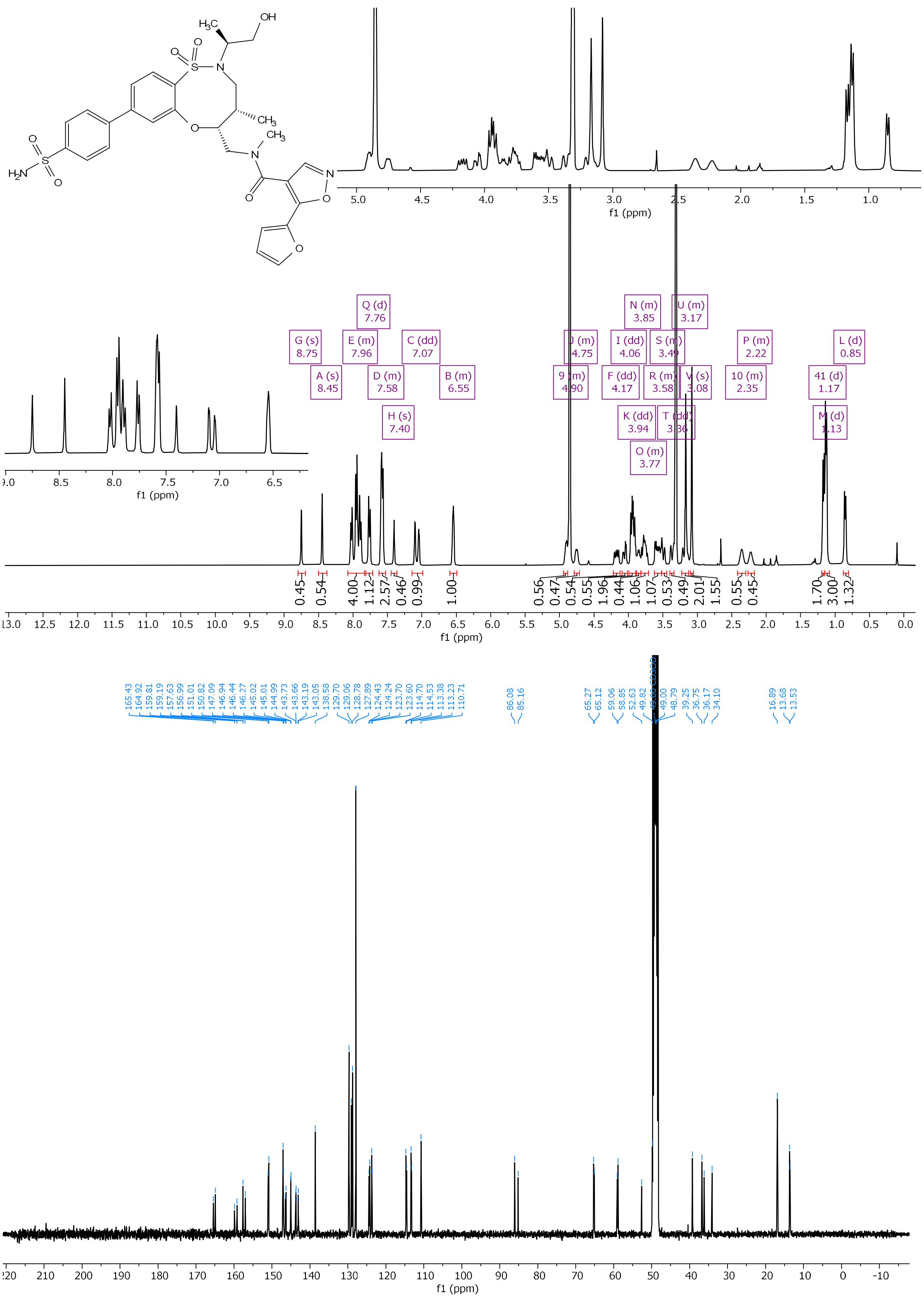

N-(((4*S*,5*S*)-2-((*R*)-1-hydroxypropan-2-yl)-4-methyl-1,1-dioxido-8-(4-sulfamoylphenyl)-2,3,4,5-tetrahydrobenzo[*b*][1,4,5]oxathiazocin-5-yl)methyl)-*N*-methyl-5-(methylsulfonyl)furan-3-carboxamide (**58**)

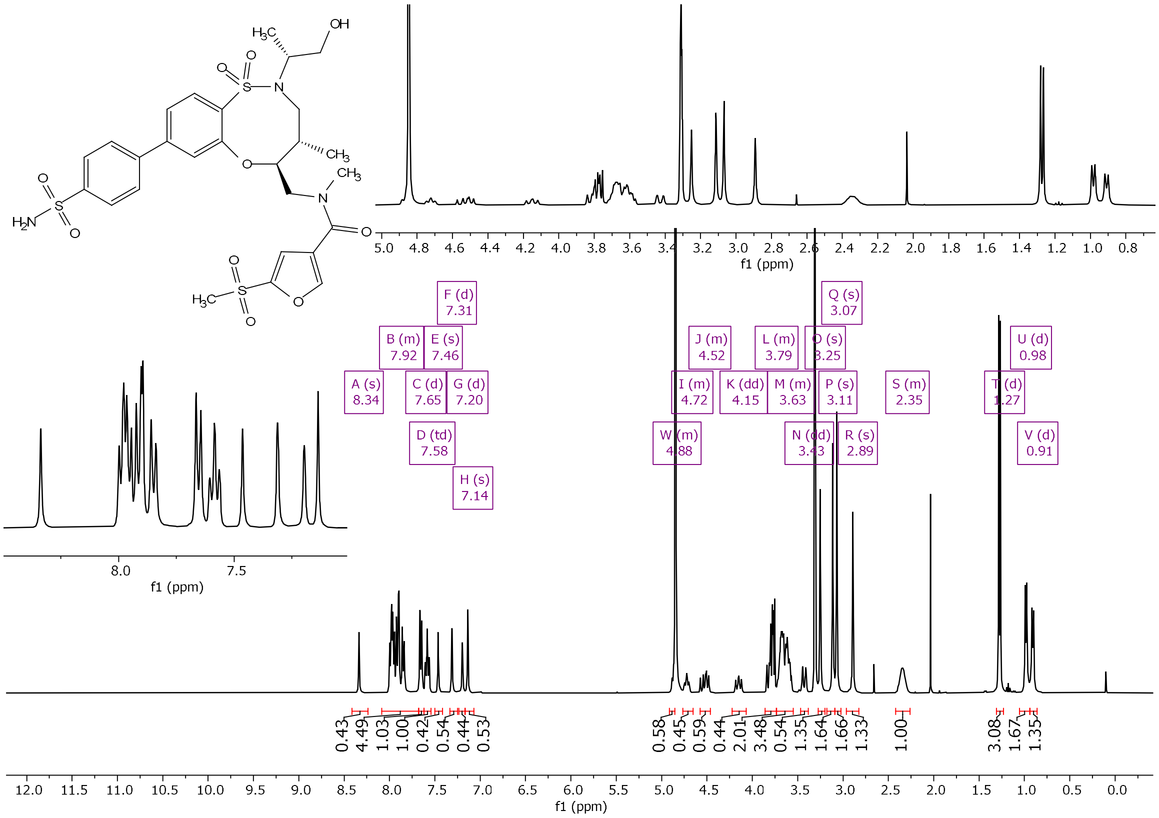

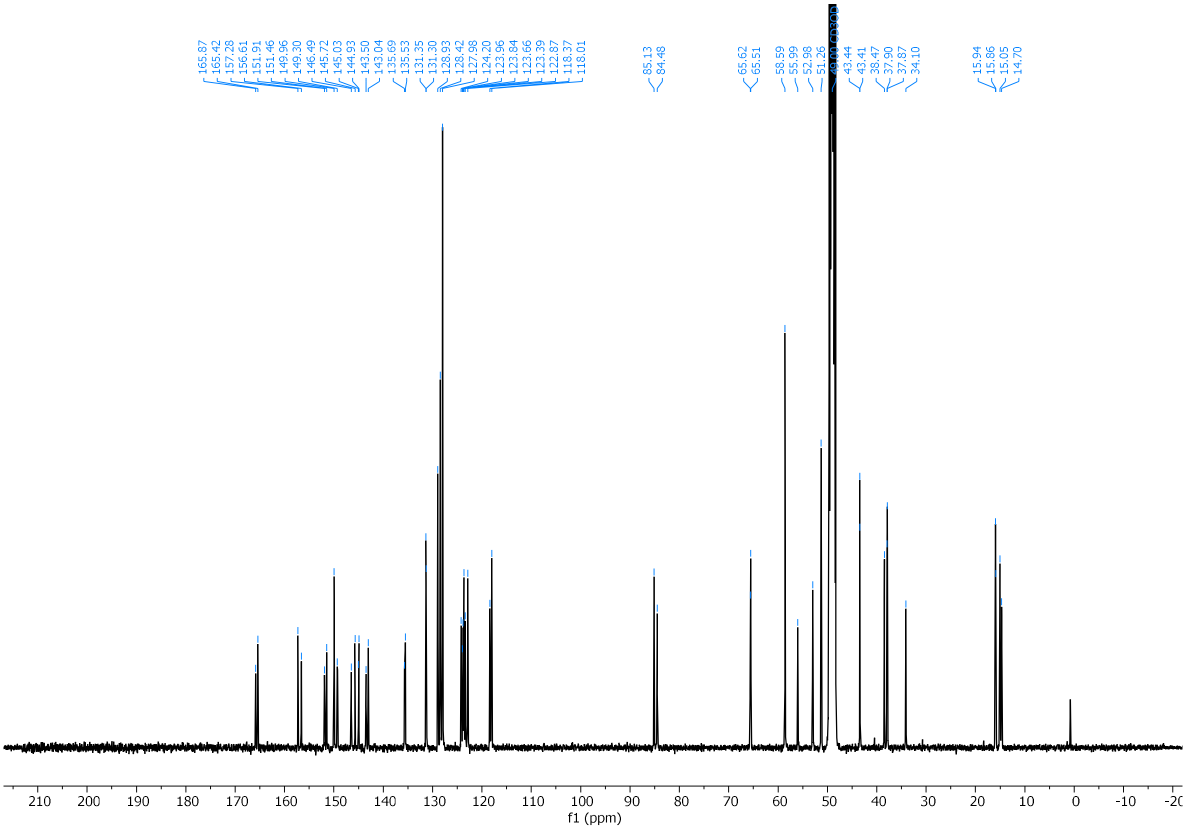
165.87
165.42
157.28
156.61
151.91
151.46
149.96
149.30
146.49
145.72
145.03
144.93
143.50
143.04
135.69
135.53
131.35
131.30
128.93
128.42
127.98
124.20
123.96
123.84
123.66
123.39
122.87
118.37
118.01
85.13
84.48
65.62
65.51
58.59
55.99
52.98
51.26
49.00 CD3OD
43.44
43.41
38.47
37.90
37.87
34.10
15.94
15.86
15.05
14.70
f1 (ppm)

5-(Furan-2-yl)-*N*-(((4*R*,5*R*)-2-((*S*)-1-hydroxypropan-2-yl)-4-methyl-1,1-dioxido-8-(4-sulfamoylphenyl)-2,3,4,5-tetrahydrobenzo[*b*][1,4,5]oxathiazocin-5-yl)methyl)-*N*-methylisoxazole-4-carboxamide (**59**)

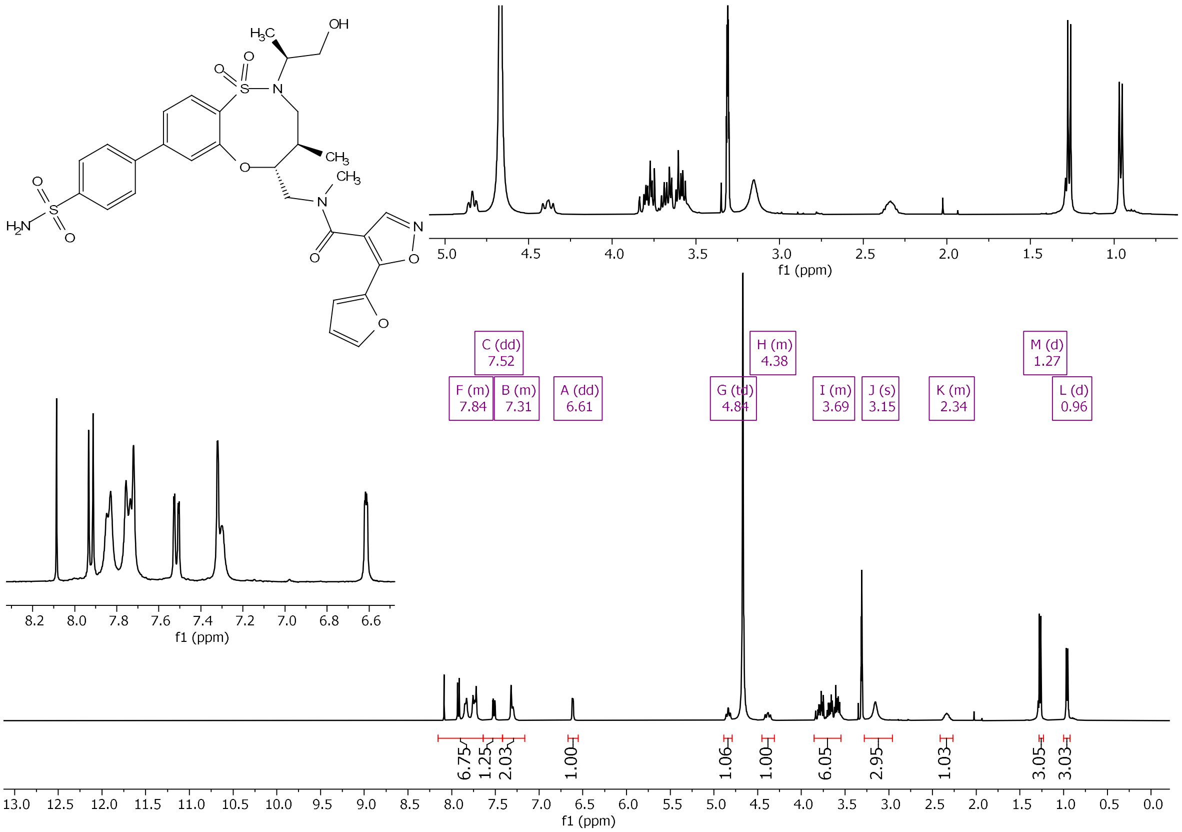

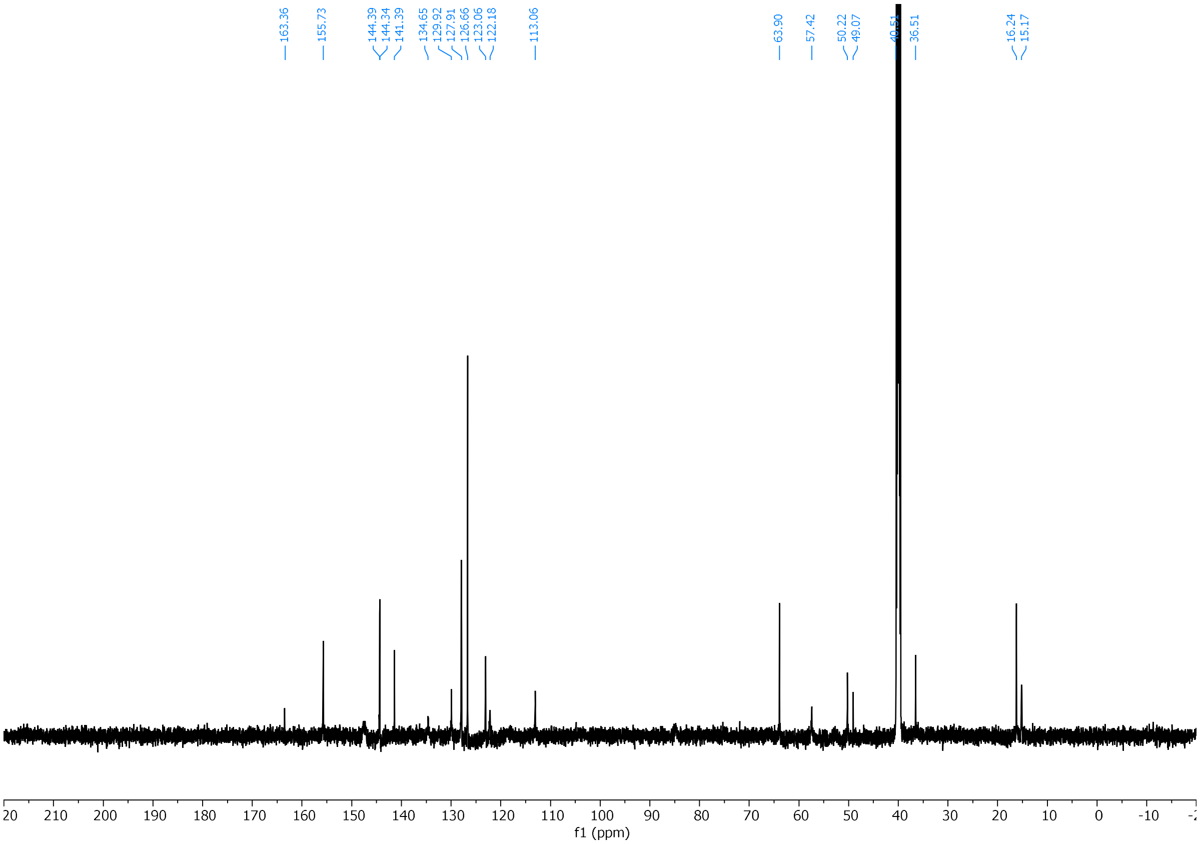
163.36
155.73
144.39
144.34
141.39
134.65
129.92
127.91
126.66
123.06
122.18
113.06
63.90
57.42
50.22
49.07
40.51
36.51
16.24
15.17
f1 (ppm)

(*R*,*E*)-4-(4-(2-cyclohexylvinyl)phenyl)-2-((3,5-dichlorophenyl)sulfonyl)-3-(2-hydroxyethyl)-*N*-methyl-2,3-dihydro-1H-pyrrolo[3,4-*c*]pyridine-6-carboxamide (**60**)

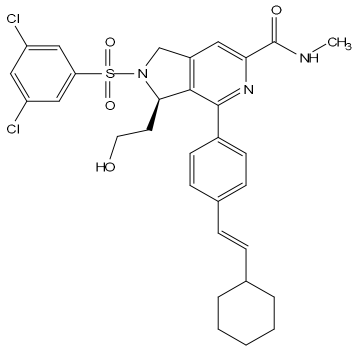

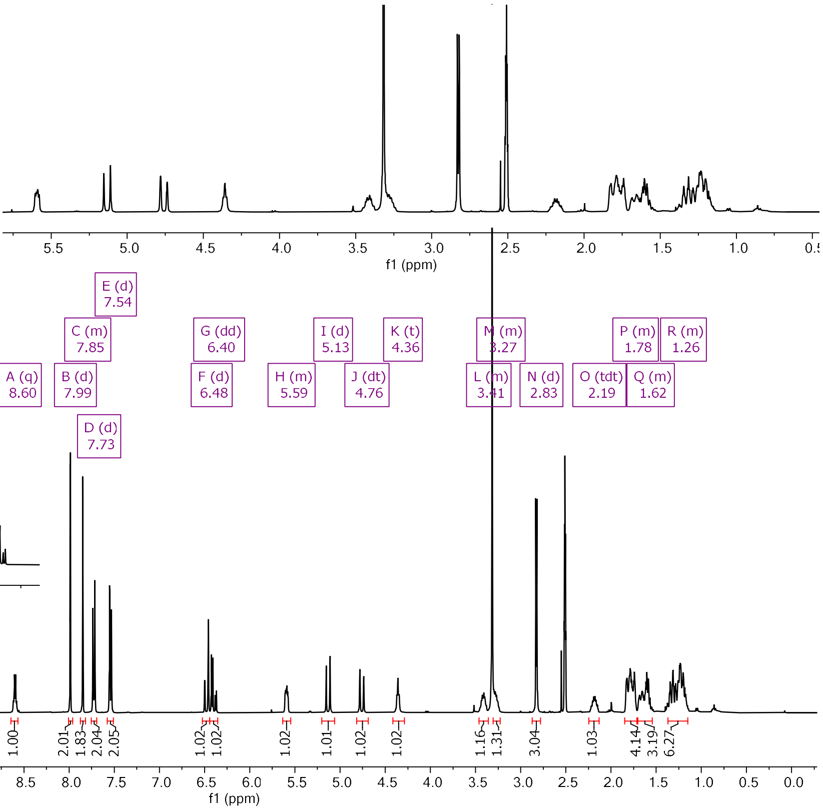

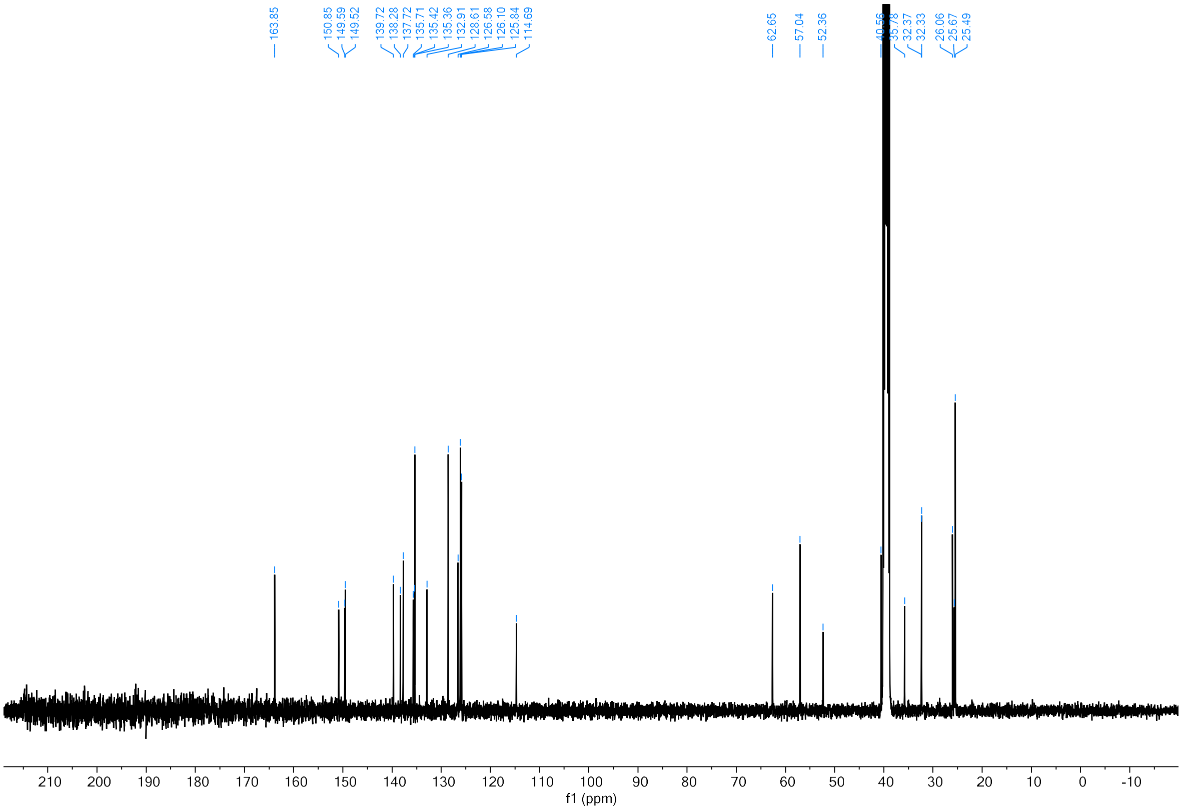

((8*R*,9*R*,10*S*)-9-(4-((*E*)-2-cyclohexylvinyl)phenyl)-6-((2-methoxyphenyl)sulfonyl)-1,6-diazabicyclo[6.2.0]decan-10-yl)methanol (**61**)

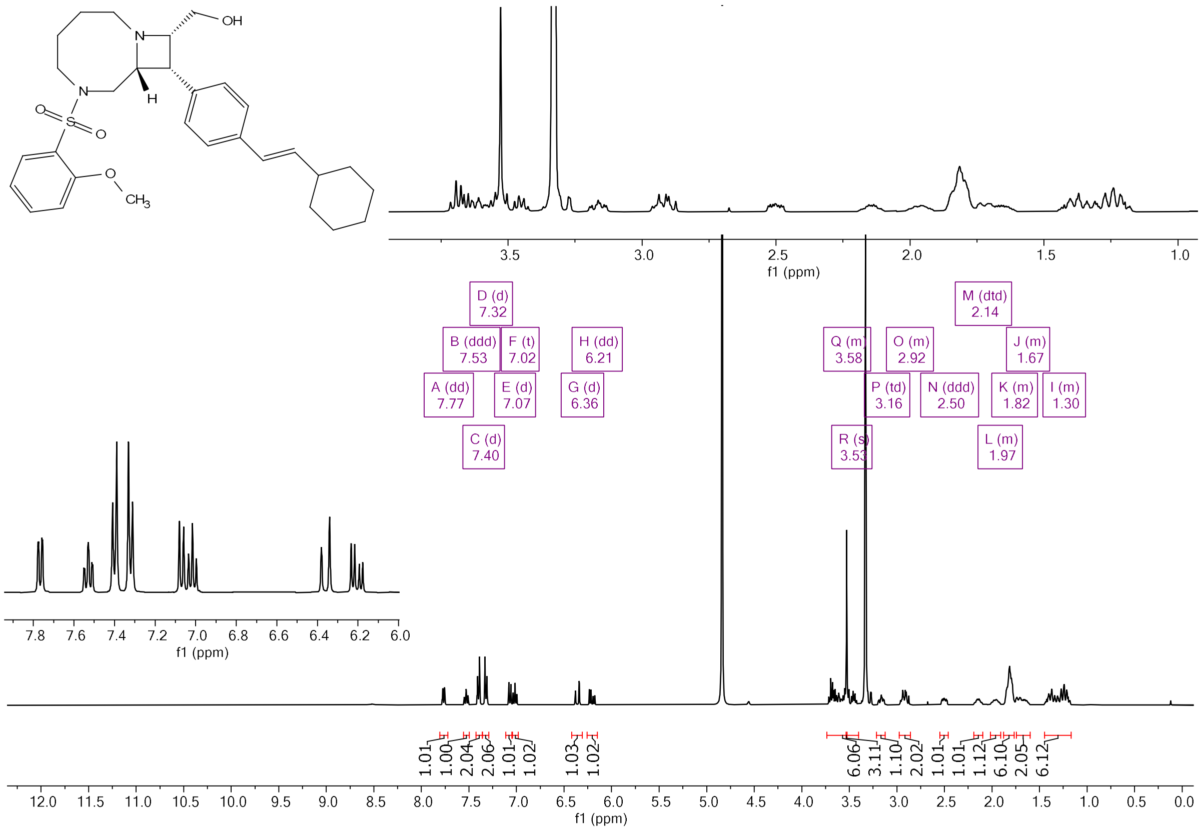

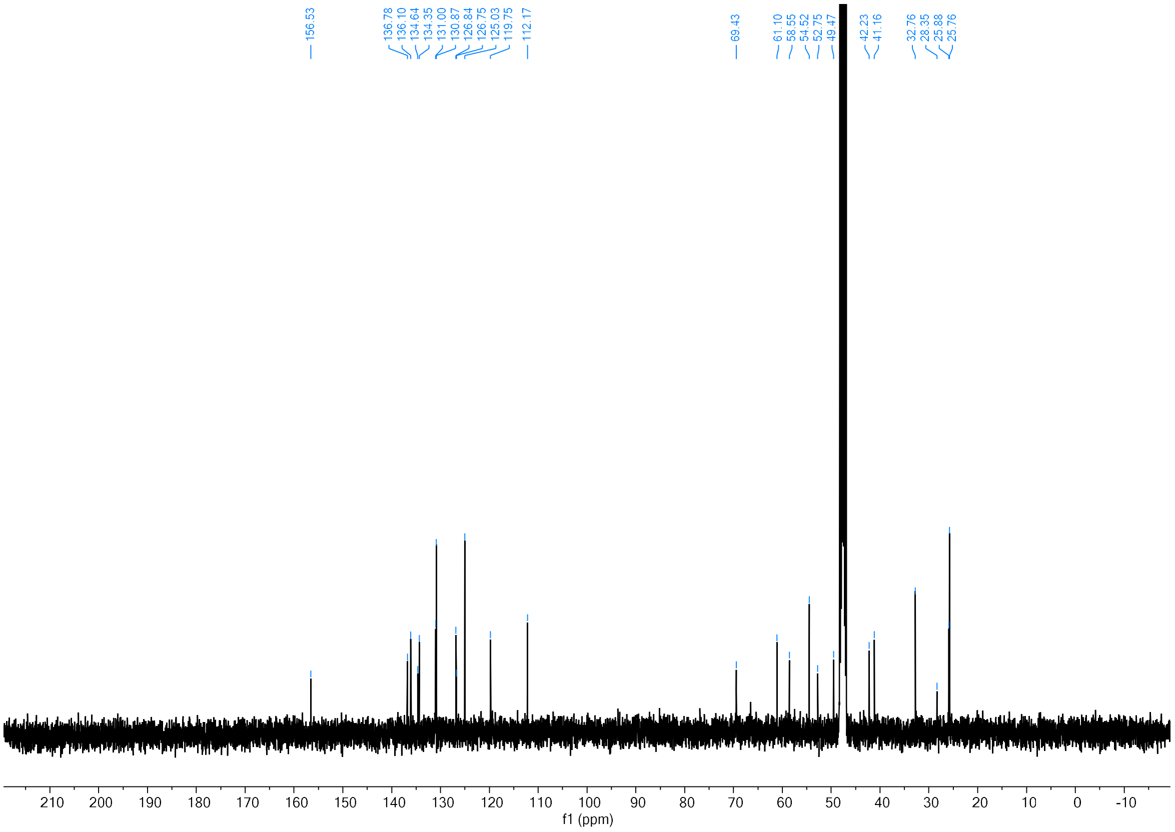
156.53
136.78
136.10
134.64
134.35
131.00
130.87
126.84
126.75
125.03
119.75
112.17
69.43
61.10
58.55
54.52
52.75
49.47
42.23
41.16
32.76
28.35
25.88
25.76
210 200 190 180 170 160 150 140 130 120 110 100 90 80 70 60 50 40 30 20 10 0 -10
f1 (ppm)

(2*R*,4*S*)-4-(3-((*E*)-2-cyclohexylvinyl)phenoxy)-1-((3,5-dichlorophenyl)sulfonyl)-*N*-methylpyrrolidine-2-carboxamide (**62**)

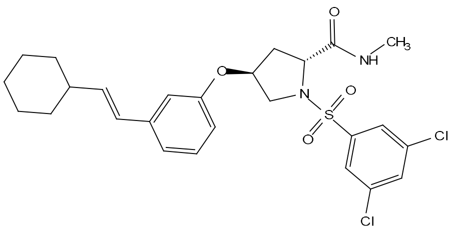

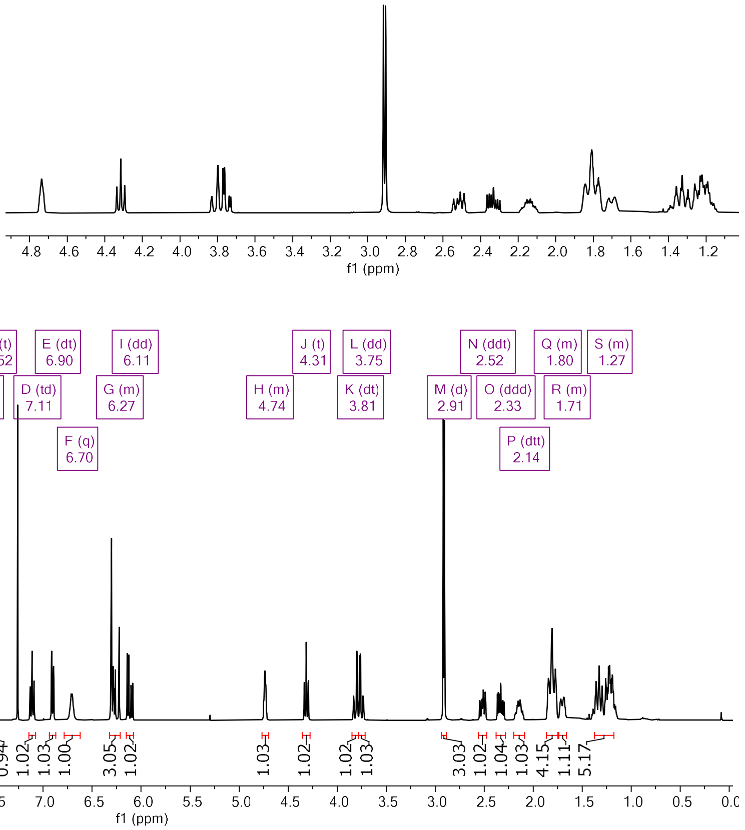

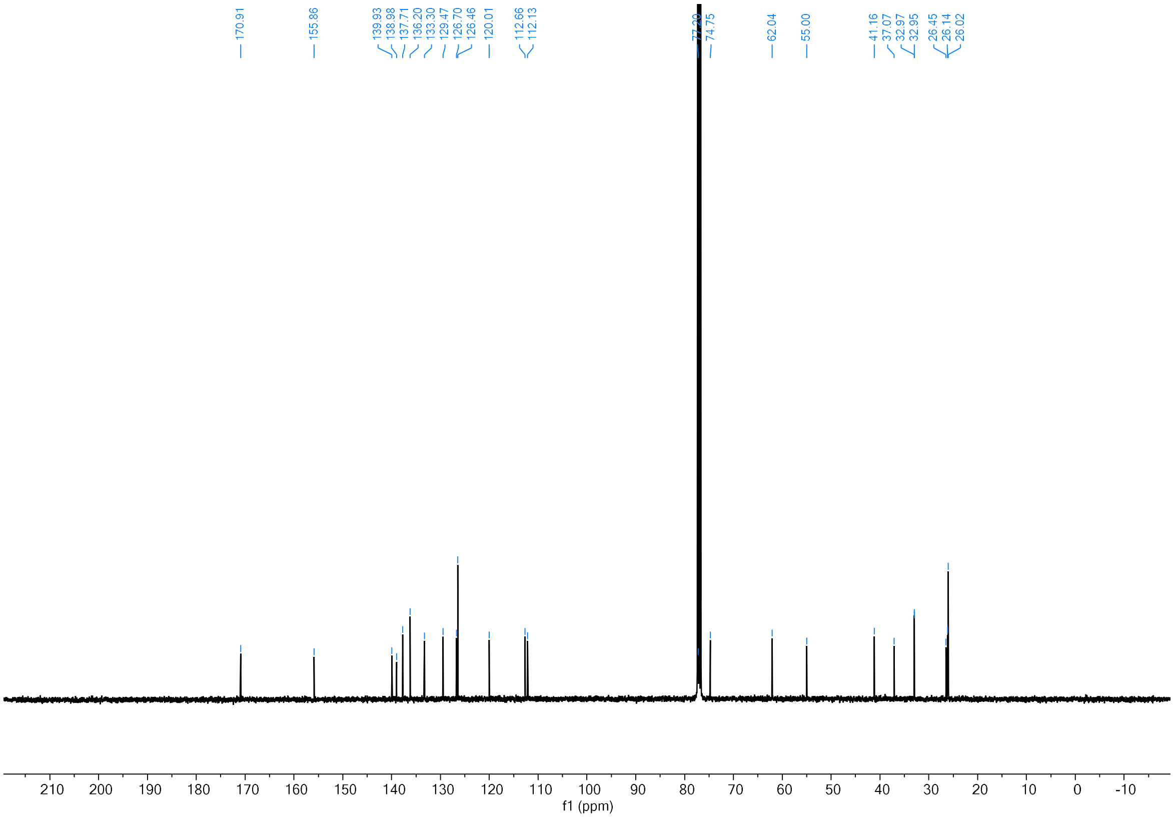

(2*S*,4*S*)-4-(4-((*E*)-2-cyclohexylvinyl)phenoxy)-1-(3-ethynylbenzyl)-*N*-methylpyrrolidine-2-carboxamide (**63**)

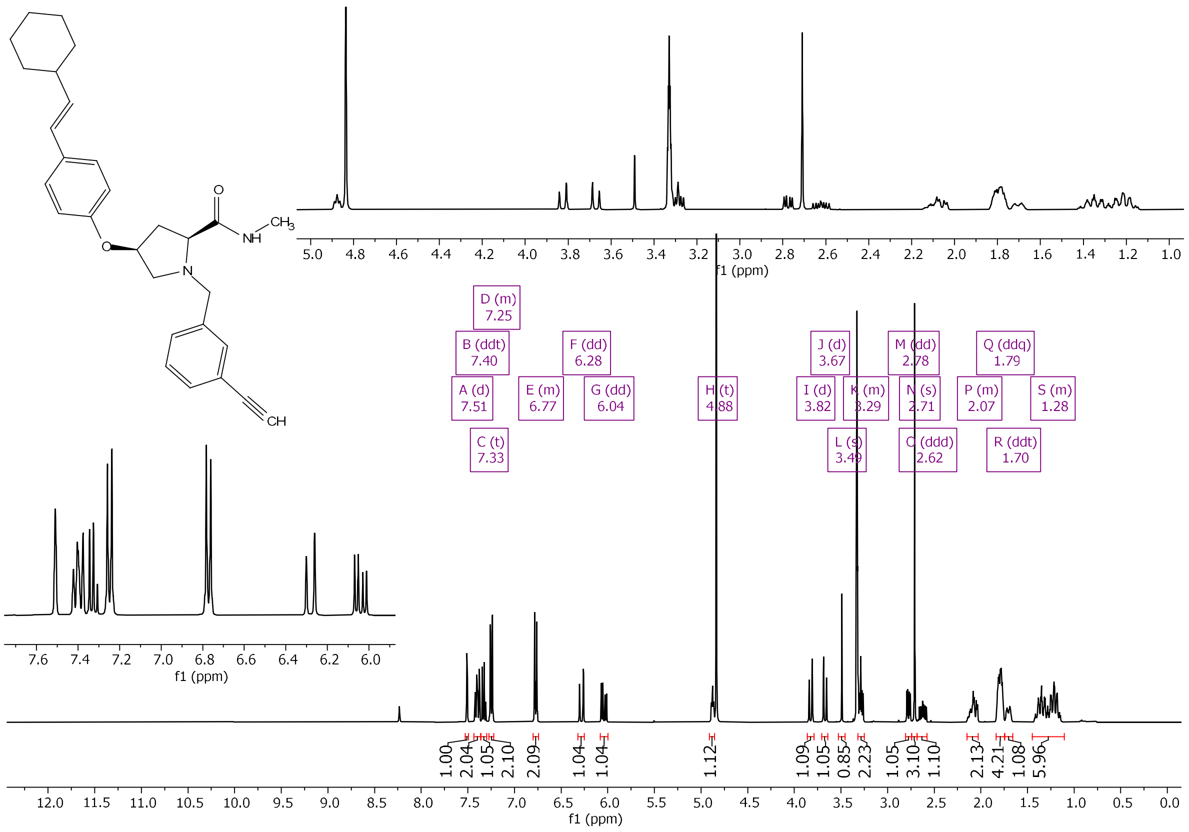

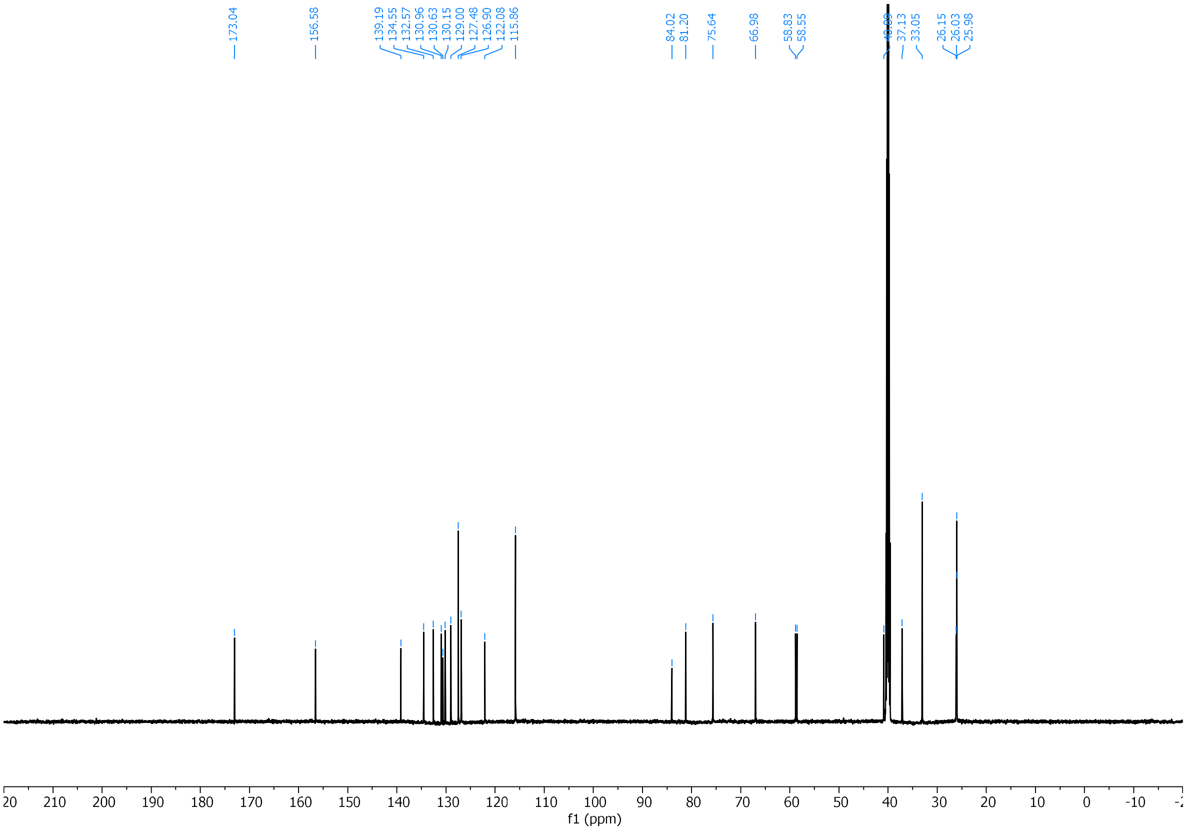

8. On-DNA analytical data

8.1. DNA-skeleton conjugate purity

Supplementary Table 11 | LCMS data for purified skeleton-AOP-headpiece conjugates. [3H] denotes DNA attachment point

i	Tag (5'-3')	smiles	%AUC	rt_min
1	GCACCATCT	[3H]CNC(=O)[C@@H]1NC[C@@H]1c1ccc(I)cc1	100	2.31
2	GCACTCTCT	[3H]CNC(=O)[C@H]1NC[C@H]1c1ccc(I)cc1	100	2.31
3	TCTCGCTCT	[3H]CNC(=O)[C@H]1NC[C@@H]1c1ccc(I)cc1	96	2.4
4	ATCTCGGCT	[3H]CNC(=O)[C@@H]1NC[C@H]1c1ccc(I)cc1	98	2.41
5	ATGGAGGCT	[3H]CNC(=O)[C@@H]1NCC[C@@H]1c1ccc(I)cc1	100	2.34
6	CGCACAACT	[3H]CNC(=O)[C@H]1NCC[C@H]1c1ccc(I)cc1	100	2.34
7	CGCACTTCT	[3H]CNC(=O)[C@H]1NCC[C@@H]1c1ccc(I)cc1	99	2.4
8	TGGAAGCCT	[3H]CNC(=O)[C@@H]1NCC[C@H]1c1ccc(I)cc1	97	2.4
9	TGGAGACCT	[3H]CNC(=O)[C@@H]1NC[C@@H]1c1cccc(I)c1	99	2.29
10	AGATGGCCT	[3H]CNC(=O)[C@H]1NC[C@H]1c1cccc(I)c1	99	2.29
11	AGGACTGCT	[3H]CNC(=O)[C@H]1NC[C@@H]1c1cccc(I)c1	96	2.33
12	AGTACCGCT	[3H]CNC(=O)[C@@H]1NC[C@H]1c1cccc(I)c1	100	2.33

13	CTCTACGCT	[3H]CNC(=O)[C@@H]1NCC[C@@H]1c1cccc(I)c1	100	2.33
14	GACAGACCT	[3H]CNC(=O)[C@H]1NCC[C@H]1c1cccc(I)c1	100	2.33
15	GGCAACACT	[3H]CNC(=O)[C@H]1NCC[C@@H]1c1cccc(I)c1	100	2.35
16	GACCGAACT	[3H]CNC(=O)[C@@H]1NCC[C@H]1c1cccc(I)c1	100	2.35
17	GAGCTGACT	[3H]CNC(=O)[C@@H]1NCCC[C@@H]1c1cccc(I)c1	100	2.39
18	GCGAAGACT	[3H]CNC(=O)[C@H]1NCCC[C@H]1c1cccc(I)c1	100	2.39
19	GGCGATTCT	[3H]CNC(=O)[C@H]1NCCC[C@@H]1c1cccc(I)c1	100	2.4
20	TAGACCGCT	[3H]CNC(=O)[C@@H]1NCCC[C@H]1c1cccc(I)c1	100	2.4
21	TCACGACCT	[3H]COC[C@@H]1[C@@H](c2ccc(Br)cc2)[C@@H]2CNCCCC N12	100	2.37
22	TCCACCACT	[3H]COC[C@@H]1[C@@H](c2ccc(Br)cc2)[C@@H]2CNCC(=O) N12	100	2.33
23	TCGCTGTCT	[3H]COC[C@H]1[C@@H](c2ccc(Br)cc2)[C@H]2CNCCCCN21	99	2.3
24	TCTTGGCCT	[3H]COC[C@@H]1[C@H](c2ccc(Br)cc2)[C@@H]2CNCC(=O)N 12	100	2.27
25	TGACGGACT	[3H]COC[C@@H]1[C@@H](c2ccc(Br)cc2)[C@H]2CNCC(=O)N 21	99	2.32
26	GATGCTCCT	[3H]COC[C@H]1[C@H](c2ccc(Br)cc2)[C@@H]2CNCC(=O)N21	100	2.32
27	GAGGTTCCT	[3H]COC[C@H]1[C@H](c2ccc(Br)cc2)[C@H]2CNCC(=O)N21	99	2.32
28	TACGGTGCT	[3H]COC[C@@H]1[C@H](c2ccc(Br)cc2)[C@H]2CNCCCCN21	98	2.24
29	GCTGCAACT	[3H]COC[C@@H]1[C@H](c2ccc(Br)cc2)[C@@H]2CNCCCCN1 2	100	2.3
30	GCTTAGGCT	[3H]COC[C@@H]1[C@@H](c2ccc(Br)cc2)[C@H]2CNCCCCN2 1	99	2.25
31	GGTCTGACT	[3H]COC[C@H](C)N1C[C@H](C)[C@H](CNC)Oc2cc(Br)ccc2S1 (=O)=O	100	2.26
32	ACTTCGCCT	[3H]COC[C@@H](C)N1C[C@H](C)[C@H](CNC)Oc2cc(Br)ccc2 S1(=O)=O	99	2.31
33	AGACGCACT	[3H]COC[C@H](C)N1C[C@@H](C)[C@@H](CNC)Oc2cc(Br)cc c2S1(=O)=O	99	2.31
34	AGCGAGACT	[3H]COC[C@@H](C)N1C[C@@H](C)[C@@H](CNC)Oc2cc(Br) ccc2S1(=O)=O	100	2.26
35	AGTCGTGCT	[3H]COC[C@@H](C)N1C[C@H](C)[C@@H](CNC)Oc2cc(Br)cc c2S1(=O)=O	93	2.34
36	ATCCAGCCT	[3H]COC[C@@H](C)N1C[C@@H](C)[C@H](CNC)Oc2cc(Br)cc c2S1(=O)=O	100	2.31

37	CGAGTCTCT	[3H]COC[C@H](C)N1C[C@H](C)[C@@H](CNC)Oc2cc(Br)ccc2S1(=O)=O	100	2.31
38	CAGCGATCT	[H]OC(=O)c1cc2c(c(-c3ccc(Br)cc3)n1)[C@@H](CCOC[3H])NC2	100	2.24
39	CAGCTACCT	[H]OC(=O)c1cc2c(c(-c3cccc(Br)c3)n1)[C@H](CCOC[3H])NC2	100	2.25
40	CATACCGCT	[3H]CNC(=O)c1cc2c(c(-c3ccc(Br)cc3)n1)[C@@H](CCO)NC2	91	2.34
41	CGACAGTCT	[3H]CNC(=O)c1cc2c(c(-c3cccc(Br)c3)n1)[C@H](CCO)NC2	90	2.33
42	CGACCAACT	[3H]CC(=O)N1C[C@H](c2ccc(I)cc2)C2(CNC2)C1	96	2.23
43	GCTTCCACT	[3H]CC(=O)N1C[C@@H](c2ccc(I)cc2)C2(CNC2)C1	98	2.23
44	CTGAGGACT	[3H]CC(=O)N1CC2(CNC[C@@H]2c2ccc(I)cc2)C1	99	2.21
45	GACCGTTCT	[3H]CC(=O)N1CC2(CNC[C@H]2c2ccc(I)cc2)C1	99	2.21
46	GAGTAGCCT	[3H]CNC(=O)[C@@H]1C[C@H](Oc2ccc(I)cc2)CN1	97	2.37
47	GCTCGAACT	[3H]CNC(=O)[C@@H]1C[C@@H](Oc2ccc(I)cc2)CN1	99	2.39
48	GCTGCTTCT	[3H]CNC(=O)[C@H]1C[C@@H](Oc2ccc(I)cc2)CN1	99	2.37
49	GCTGTCTCT	[3H]CNC(=O)[C@H]1C[C@H](Oc2ccc(I)cc2)CN1	99	2.4
50	TACGAGCCT	[3H]CNC(=O)[C@@H]1C[C@H](Oc2cccc(I)c2)CN1	100	2.4
51	TACGCGACT	[3H]CNC(=O)[C@@H]1C[C@@H](Oc2cccc(I)c2)CN1	100	2.4
52	TCCTCTGCT	[3H]CNC(=O)[C@H]1C[C@@H](Oc2cccc(I)c2)CN1	99	2.38
53	TCGGCATCT	[3H]CNC(=O)[C@H]1C[C@H](Oc2cccc(I)c2)CN1	100	2.4
54	TCGGTCACT	[3H]CNC(=O)[C@@H]1C[C@H](OCc2ccc(I)cc2)CN1	100	2.41
55	TCTGCCTCT	[3H]CNC(=O)[C@@H]1C[C@@H](OCc2ccc(I)cc2)CN1	100	2.41
56	TTGGCGTCT	[3H]CNC(=O)[C@H]1C[C@@H](OCc2ccc(I)cc2)CN1	100	2.41
57	TTGGTGCCT	[3H]CNC(=O)[C@H]1C[C@H](OCc2ccc(I)cc2)CN1	100	2.41
58	AAGGCCACT	[3H]CNC(=O)[C@@H]1C[C@H](OCc2cccc(I)c2)CN1	100	2.41
59	ACACCGACT	[3H]CNC(=O)[C@@H]1C[C@@H](OCc2cccc(I)c2)CN1	100	2.41
60	AGAACGGCT	[3H]CNC(=O)[C@H]1C[C@@H](OCc2cccc(I)c2)CN1	99	2.41
61	AGAGTGCCT	[3H]CNC(=O)[C@H]1C[C@H](OCc2cccc(I)c2)CN1	100	2.41

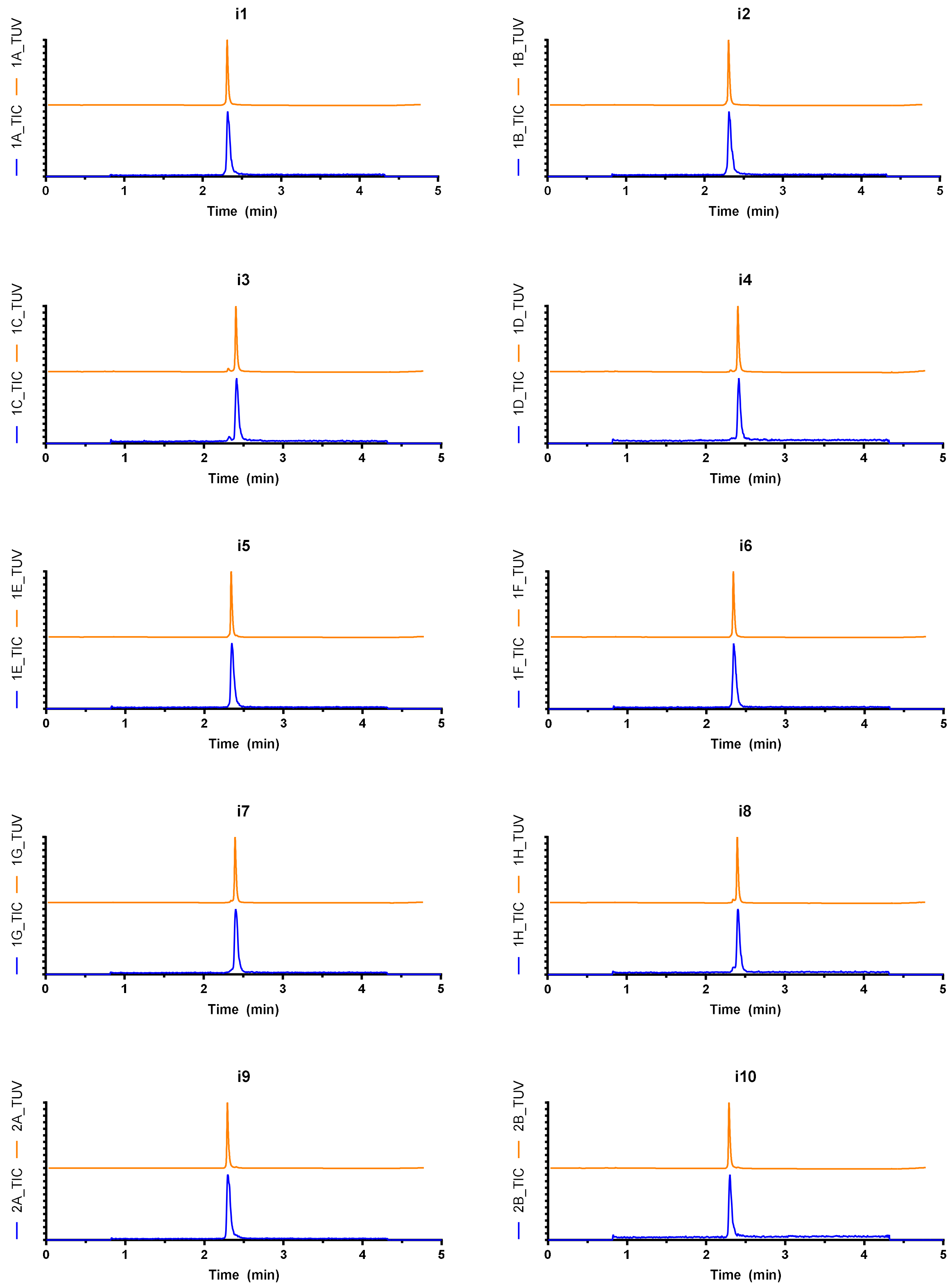

i1
1A_TIC
1A_TUV
0 1 2 3 4 5
Time (min)
i2
1B_TIC
1B_TUV
0 1 2 3 4 5
Time (min)
i3
1C_TIC
1C_TUV
0 1 2 3 4 5
Time (min)
i4
1D_TIC
1D_TUV
0 1 2 3 4 5
Time (min)
i5
1E_TIC
1E_TUV
0 1 2 3 4 5
Time (min)
i6
1F_TIC
1F_TUV
0 1 2 3 4 5
Time (min)
i7
1G_TIC
1G_TUV
0 1 2 3 4 5
Time (min)
i8
1H_TIC
1H_TUV
0 1 2 3 4 5
Time (min)
i9
2A_TIC
2A_TUV
0 1 2 3 4 5
Time (min)
i10
2B_TIC
2B_TUV
0 1 2 3 4 5
Time (min)

i11

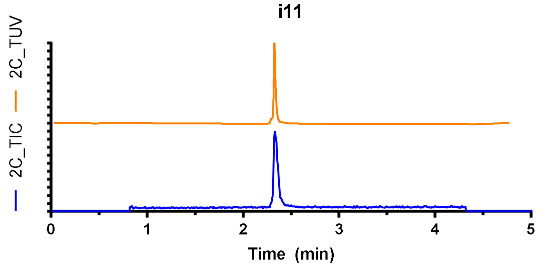

i12

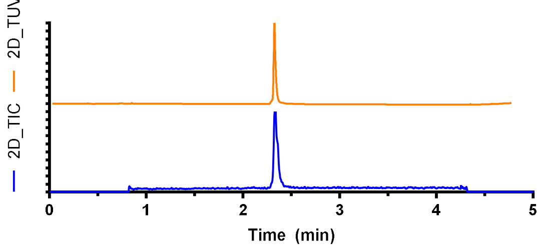

i13

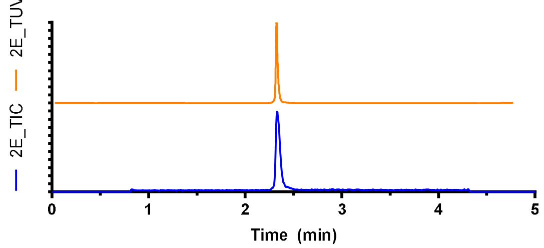

i14

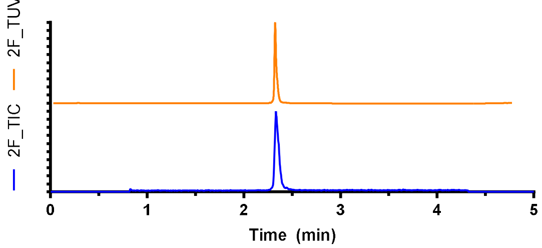

i15

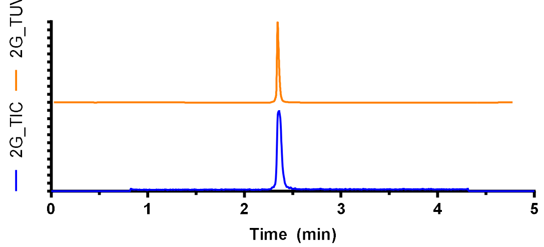

i16

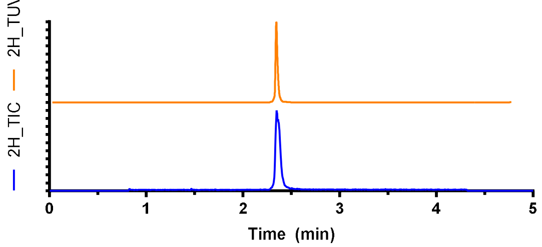

i17

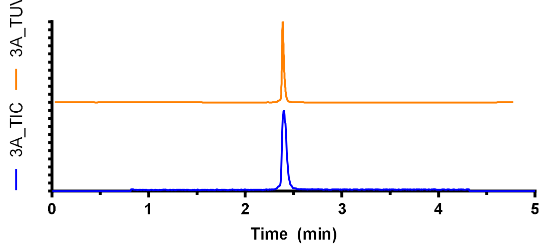

i18

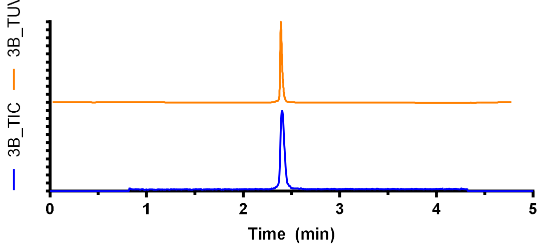

i19

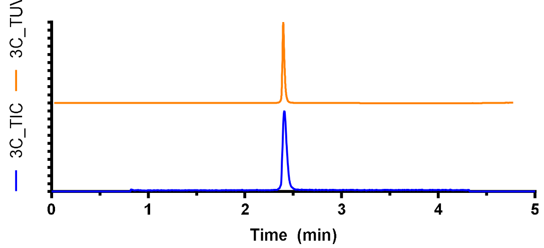

i20

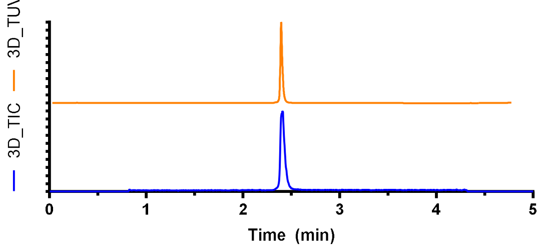

i21

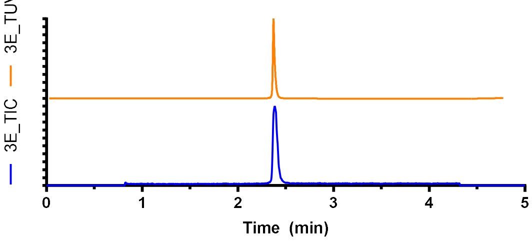

i22

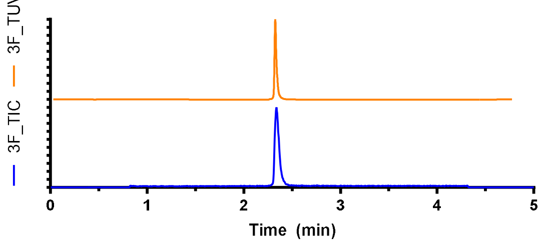

i23

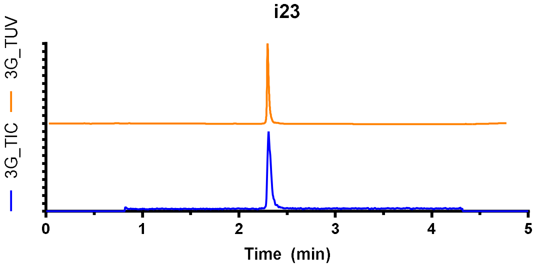

i24

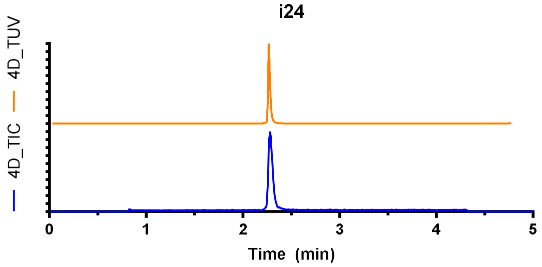

i25

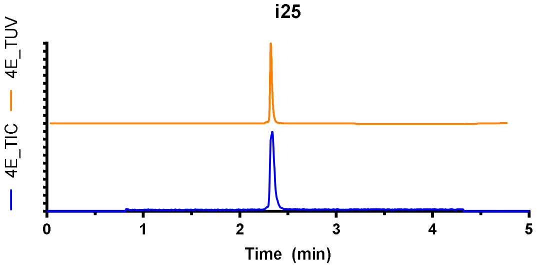

i26

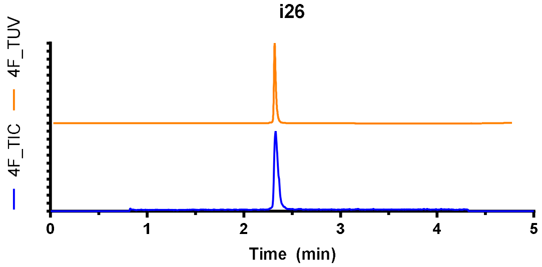

i27

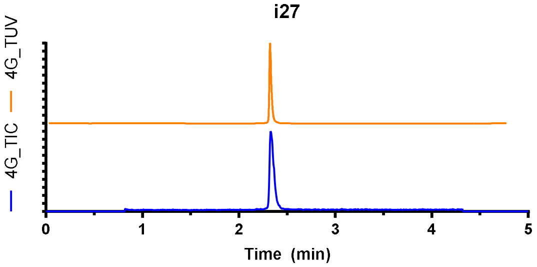

i28

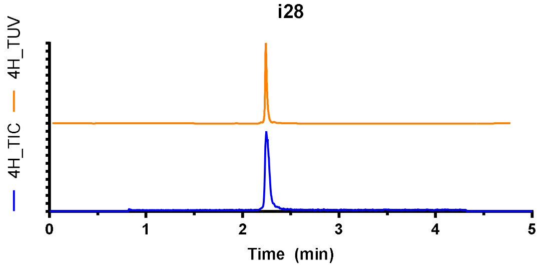

i29

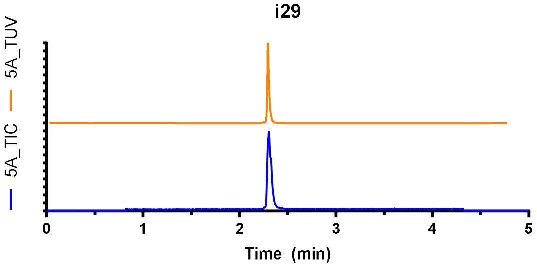

i30

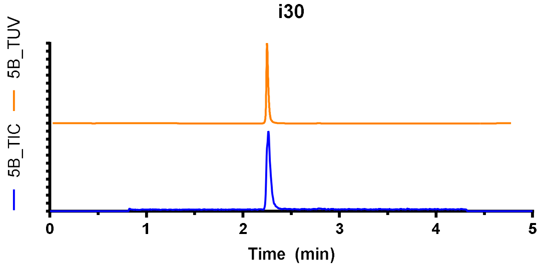

i31

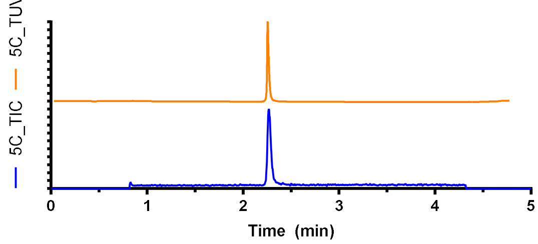

i32

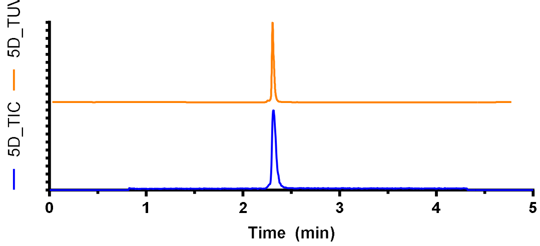

i33

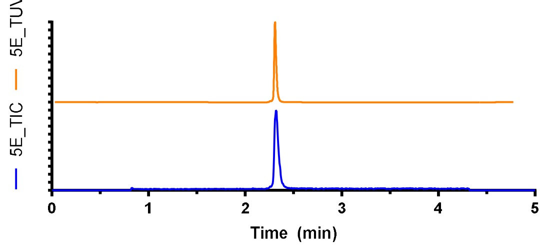

i34

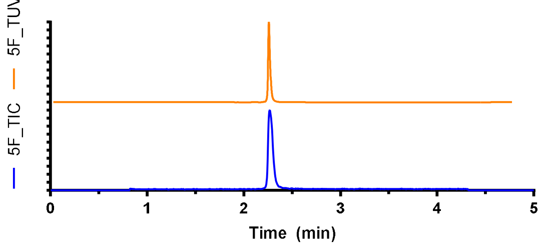

i35

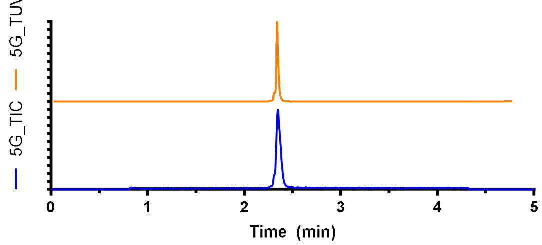

i36

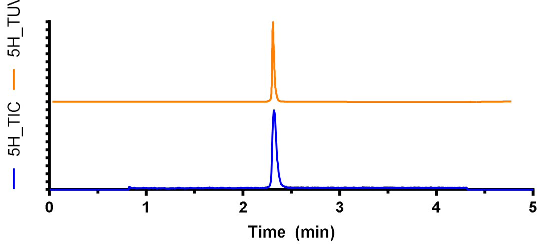

i37

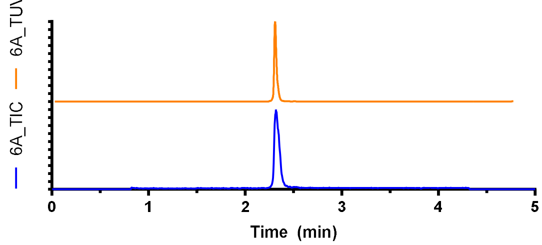

i38

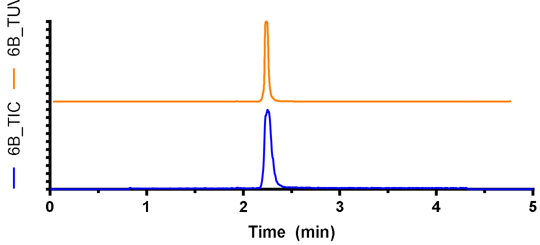

i39

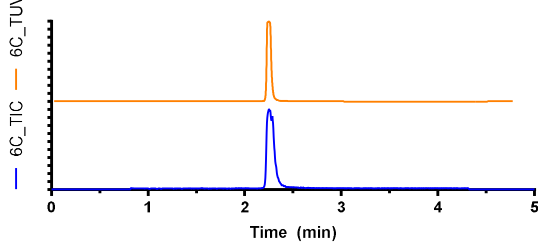

i40

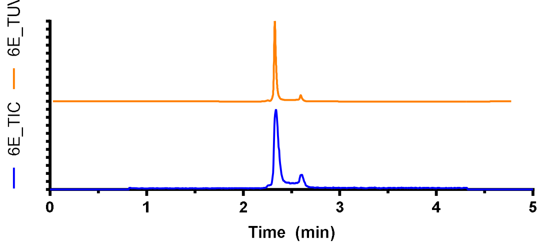

i41

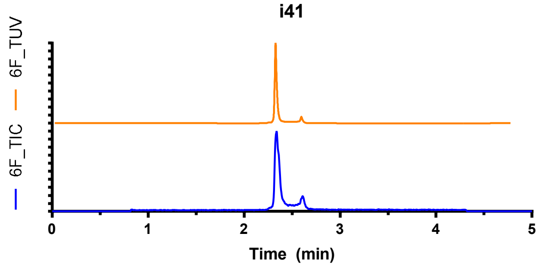

i42

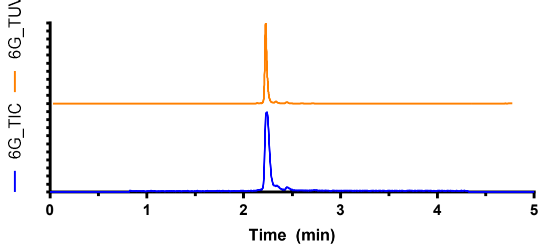

i43

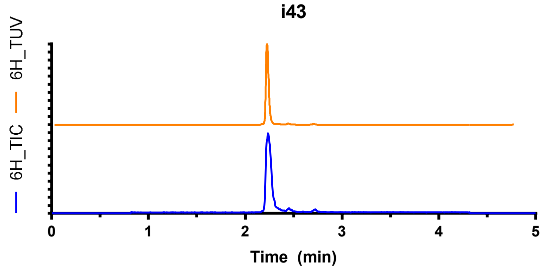

i44

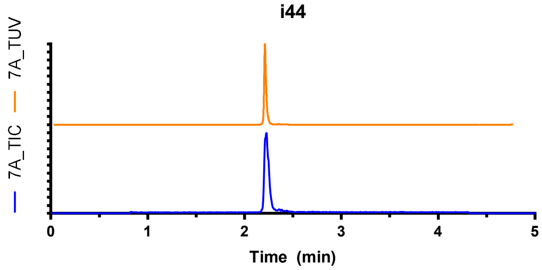

i45

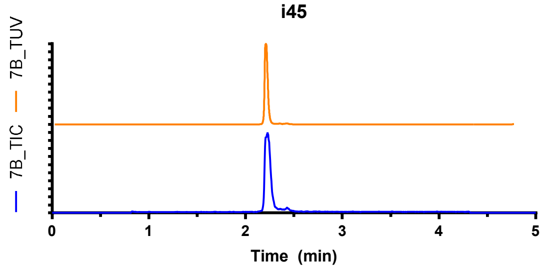

i46

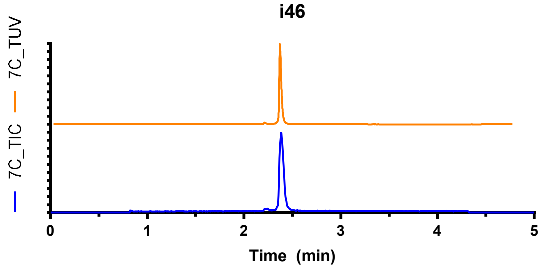

i47

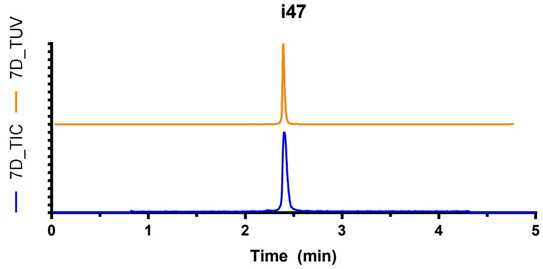

i48

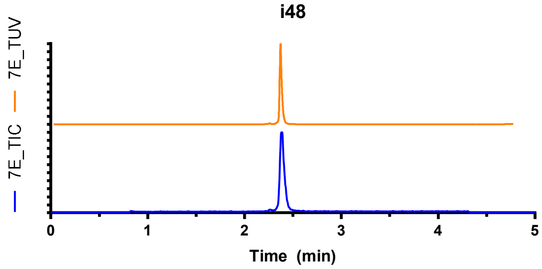

i49

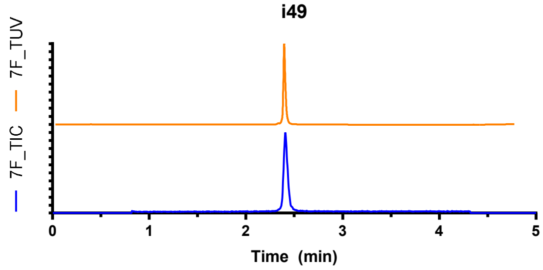

i50

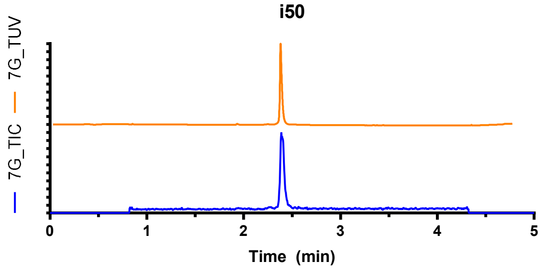

i51

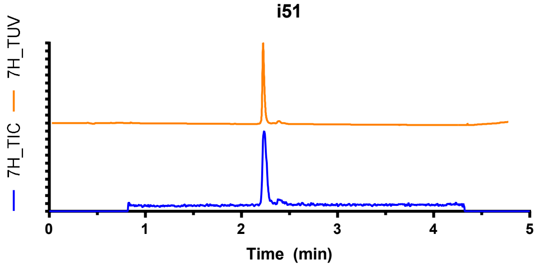

i52

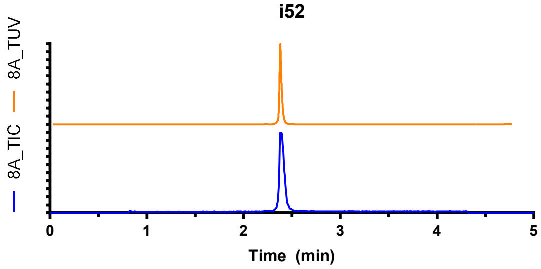

i53

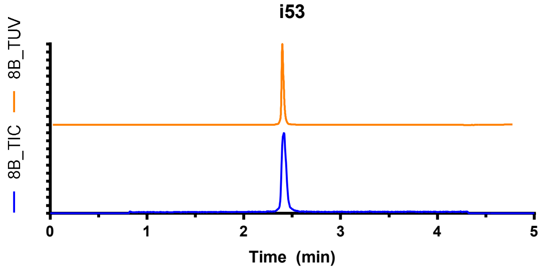

i54

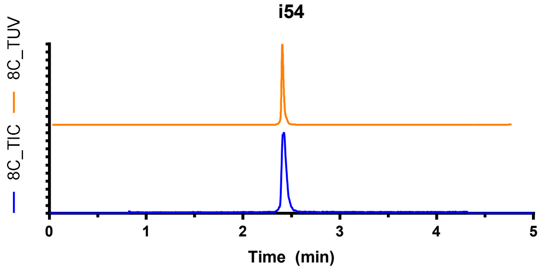

i55

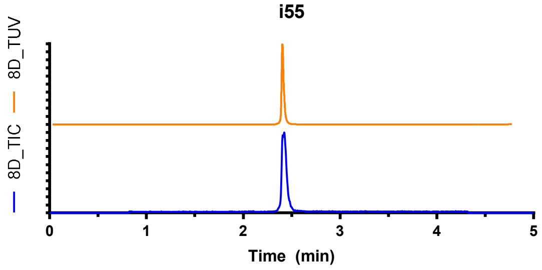

i56

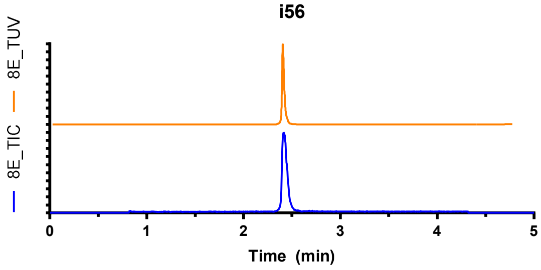

i57

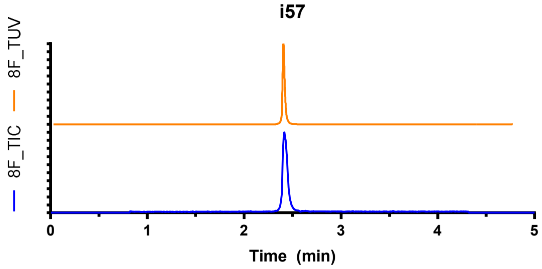

i58

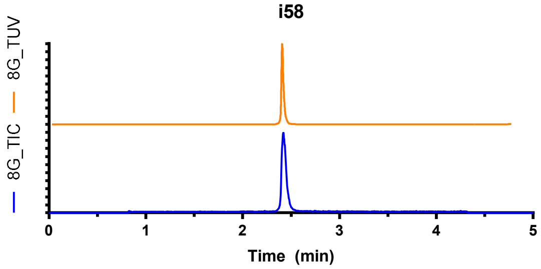

i59

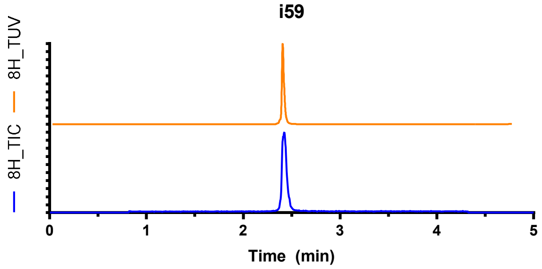

i60

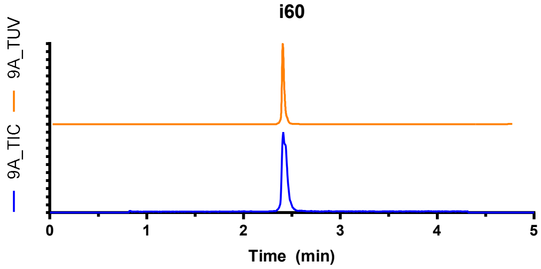

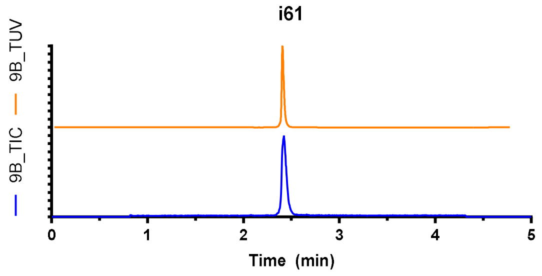

8.2. Amine capping building block validation data

Building block validation data presented below only displays the two calculated factors "P_adj%" and "unknown_adj%". "P_adj%" is the %AUC of product, or known product-derived species (e.g., carboxylic ester hydrolysis or TFA protecting group removal) when corrected for non-DNA species which may absorb at 260 nm and therefore be integrated as peaks. Non-DNA species were manually discounted based on having no analogous peak in the TIC trace, i.e., with a mass below the lower threshold of m/z monitoring (m/z = 500). "unknown_adj%" is the %AUC of any unknown DNA-based species (i.e., the mass data obtained was confounding, or simply could not be assigned to a known species) when corrected for peaks assigned as residual small molecule building block.

Supplementary Table 12 | Amine capping building block validation data

Structure	P_adj%	unknown_adj%
**Cn1cnn(CCC(=O)O)c1=O**	100	0
**C#CC1(O)CC(C(=O)O)C1**	100	0
**O=C1Cc2c(C(=O)O)ccnc2N1**	100	0
**C/C(=C\c1ccccn1)C(=O)O**	100	0
**Cn1ccc(-c2cc(C(=O)O)n[nH]2)c1**	100	0
**Cc1nc2ccc(C(=O)O)cc2n1C**	100	0
**Cn1nnc2c1NCCC2C(=O)O**	99	0
**O=C(O)c1nc2n(n1)CCCN2**	99	0
**O=C(O)c1cn(C2CCC2O)nn1**	99	1
**Cn1nnc2c(C(=O)O)ccnc21**	99	1
**O=C(O)c1ncoc1C1CCCO1**	99	0
**CCC(O)Cn1cc(C(=O)O)nn1**	99	1
**COCc1nc(C(=O)O)co1**	99	1
**CCn1nc(C)c(C(=O)O)n1**	99	1
**O=C(O)c1cc(C2CC2)[nH]c(=O)c1**	99	0
**Cn1cc(C(=O)O)c(=O)n(C)c1=O**	99	1

Structure	P_adj%	unknown_adj%
**CC1Cc2cccc(C(=O)O)c2O1**	99	1
**O=C(O)c1cccc(-c2nnco2)c1**	99	0
**CS(=O)(=O)c1cc(C(=O)O)co1**	99	1
**CC1=C(C(=O)O)C(=O)OC1(C)C**	99	0
**Cn1ncc(CCC(=O)O)n1**	99	1
**O=C(O)CC1COc2ccccc21**	99	1
**O=C(O)c1ncc2c(F)cccn12**	99	0
**O=C(O)c1n[nH]c2c1COCC2**	99	1
**Cc1csc(/C=C/C(=O)O)n1**	99	0
**C/C(=C(/F)C(=O)O)C1CC1**	99	0
**O=C(O)c1coc2c1C(=O)CCC2**	99	1
**Cn1nc(CC(=O)O)c2ccccc21**	99	1
**Cc1cccc2nc(C(=O)O)cn12**	99	1
**CCOc1cccc(CC(=O)O)n1**	99	0
**O=C(O)c1cccc(-n2ccnn2)c1**	99	1
**O=C(O)c1nsc2ccccc12**	99	1
**O=C(O)CCc1cc2ccccc2o1**	99	1
**O=C(O)c1ccc2scnc2c1**	99	1
**O=C(O)c1coc(-c2ccccn2)n1**	99	1
**O=C(O)C1=C[C@@H](O)[C@@H](O)[C@H](O)C1**	98	0
**O=C(O)c1cc([N+](=O)[O-])cnc1O**	98	0
**Cc1onc(CO)c1C(=O)O**	98	0
**CC(=O)Nc1cccc(C(=O)O)n1**	98	0
**O=C1NC(=O)C2(CC(C(=O)O)C2)N1**	98	1
**O=C(O)c1csnc1Cl**	98	2
**O=C(O)CC1=CCOCC1**	98	2
**O=C(O)C1(n2cccn2)CC1**	98	1
**O=C(O)c1cnc2[nH]c(=O)[nH]c2c1**	98	2
**O=C(O)c1cc2n(n1)CCCC2O**	98	1
**Cc1cc(C2(C(=O)O)CC2)on1**	98	3
**COCc1ccc(C(=O)O)cc1F**	98	1
**N#Cc1cccc(C2CC2C(=O)O)c1**	98	2
**O=C(O)Cc1csc(C2CC2)n1**	98	0
**Cc1ccsc1C1CC1C(=O)O**	98	0
**O=C(O)C1=C(c2ccccc2)CCC1**	98	2

Structure	P_adj%	unknown_adj%
**O=C(O)c1ncoc1-c1ccon1**	97	3
**CS(=O)(=O)N1CC(C(=O)O)C1**	97	0
**O=C(O)c1[nH]nc([N+](=O)[O-])c1Cl**	97	2
**O=C(O)CCNC(=O)C1CCCO1**	97	3
**Cc1ncsc1COCC(=O)O**	97	3
**O=C(O)CC1CCC2(CCC2)CO1**	97	3
**O=C(O)C1=NN(CCO)C(=O)CC1**	97	0
**CCC(=O)NC1CCC(C(=O)O)C1**	97	3
**O=C(O)CCc1cc(Cl)cs1**	97	3
**COC(C)c1nc(C(=O)O)cs1**	97	3
**CCOc1oc(C)nc1C(=O)O**	97	3
**O=C(O)c1cnc(CC2CC2)s1**	97	1
**Cc1nc2c(C(=O)O)cccc2o1**	97	0
**CN(C)c1cc(C#N)cc(C(=O)O)c1**	97	3
**Cc1nc(CC(=O)O)c(C)s1**	97	3
**O=C(O)c1cc(-n2cccn2)ccn1**	97	1
**O=C(O)c1cc(-c2ccccc2)co1**	97	1
**CC(=O)[C@@H]1C[C@H](CC(=O)O)C1(C)C**	96	2
**O=C(O)COCc1cc[nH]c(=O)c1**	96	4
**O=C(O)c1ccnc(-c2ncc[nH]2)c1**	96	4
**Cc1cc(C)cc(CC(=O)O)c1**	96	4
**O=C(O)/C=C/c1ccc2c(c1)CCO2**	96	0
**Cc1sc(Cl)c(C(=O)O)c1C**	96	0
**O=C(O)Cn1ncc2cccnc21**	96	2
**O=C(O)C1CCc2nnc(O)n2C1**	95	0
**CC1(C)CC2(CCO1)CC2C(=O)O**	95	0
**O=C(O)c1cc2c(s1)CCOC2**	95	1
**O=C(O)Cc1n[nH]c2c1CCCC2**	95	4
**CC(Oc1cccc(C#N)c1)C(=O)O**	95	0
**O=C(O)CC1C(=O)Nc2ccccc21**	95	0
**O=C(O)CCn1ccc2ccccc21**	95	3
**Cc1nc(CC2CC2)oc1C(=O)O**	95	5
**Cc1c(C(=O)O)nn2c1OCCC2**	95	5
**O=C(O)CN1Cc2ccccc2C1=O**	95	5
**O=C(O)/C=C/c1ccc2[nH]ccc2c1**	95	1

Structure	P_adj%	unknown_adj%
**COc1ccc2cc(C(=O)O)[nH]c2c1**	95	5
**Cc1cc(C(=O)O)c(C)n1C1CC1**	95	5
**O=C(O)/C=C1\CN2CCC1CC2**	94	1
**O=C(O)c1ccc2oc(=O)[nH]c2n1**	94	3
**Cc1oc(C2CC2)nc1C(=O)O**	94	6
**C#CCOc1ccc(C(=O)O)cc1**	94	6
**Cn1nnc(C(=O)O)c1C(F)F**	93	0
**CCN1C(=O)CCC1CC(=O)O**	93	1
**Cc1nn(C)c(C)c1OCC(=O)O**	93	1
**COC1(C(=O)O)CS(=O)(=O)C1**	93	2
**O=C(O)c1conc1C1CCCC1**	93	3
**O=C(O)C1CCc2nnnn2CC1**	93	7
**O=C(O)c1cnoc1-c1ccco1**	93	7
**O=C(O)CCC1CCc2ccccc21**	93	7
**O=C(O)c1ccc2c(c1)B(O)OC2**	93	5
**Cc1cnc2nc(C(=O)O)nn2c1**	93	7
**CCn1nc(C2CC2)cc1C(=O)O**	93	7
**Cc1nn(CC(=O)O)c(=O)o1**	92	8
**COCCN1CC(C(=O)O)CC1=O**	92	8
**Cc1n[nH]c(C)c1C(C)C(=O)O**	92	0
**CC1CCc2nc(C(=O)O)cn2C1**	92	3
**CC(C)Cc1nc(C(=O)O)c[nH]1**	92	1
**O=C(O)c1nc2c(s1)CCC2**	92	4
**O=C(O)c1cc2occc2s1**	92	3
**Cc1nc2cccnn2c1C(=O)O**	92	8
**COc1cc(C)sc1C(=O)O**	92	8
**Cc1nc(C)n(CCCC(=O)O)n1**	91	9
**CCc1cc(C(=O)O)cc(Cl)n1**	91	1
**Cc1ncc(C(=O)O)c(C(C)C)n1**	91	0
**Cc1nc2c([nH]1)CCC(C(=O)O)C2**	90	0
**N#Cc1cc(C(=O)O)cs1**	90	10
**Cc1cc(C(=O)O)c2[nH]ncc2c1**	90	2
**CCNc1cccnc1C(=O)O**	90	10
**CC(C)Oc1cnc(C(=O)O)cn1**	89	11
**Cc1cc2ccc(C(=O)O)cn2c1**	89	0

Structure	P_adj%	unknown_adj%
**O=C(O)C1(Cl)CC2CCC1O2**	88	12
**O=C(O)c1c[nH]nc1C1CCOC1**	88	6
**C#CCNc1ncc(C(=O)O)c(C)n1**	87	1
**CC1(C)C(C#N)C1(C#N)C(=O)O**	86	7
**O=C(O)c1ccc(=O)n(CCF)n1**	86	8
**O=C(O)C1CNc2ccccc2O1**	85	15
**CS(=O)c1ccccc1C(=O)O**	84	0
**Cc1cn2c(n1)CC(C(=O)O)CC2**	84	1
**O=C(O)c1cccc[n+]1[O-]**	84	11
**O=C(O)C1=NOC(c2ccccc2)C1**	84	1
**CC1(c2cc(C(=O)O)no2)CC1**	84	16
**CC1=C(C(=O)O)CCS1(=O)=O**	83	17
**O=C(O)C1CN2CCCC2CO1**	82	2
**O=C(O)c1cccnc1-n1cncn1**	82	18
**O=C(O)c1cc2sccc2[nH]1**	82	7
**N#Cc1cc(C(=O)O)n2c1CCCC2**	82	18
**CC1=C(C(=O)O)Cc2ccccc2O1**	81	6
**Cc1ccn(C(C)CC(=O)O)n1**	80	2
**Cc1nc[nH]c(=O)c1CCC(=O)O**	80	20
**C=CCNc1ncc(C(=O)O)s1**	79	6
**Cc1nc(C(=O)O)c2n1CCCC2**	79	0
**COCCn1cc(C(=O)O)c(C)n1**	78	1
**Cc1cc(C(=O)O)c2c(C)n[nH]c2n1**	78	0
**N#Cc1nc(C(=O)O)ccc1Cl**	78	7
**O=C(O)C1CC1Cc1ccccc1**	77	10
**O=C(O)c1ccc2n[nH]c(=O)n2c1**	77	23
**O=C(O)c1ocnc1C(F)(F)F**	75	12
**O=C(O)CNC(=O)Cn1cncn1**	75	1
**Cc1nc2sccn2c1C(=O)O**	75	10
**Cn1cccc1/C=C/C(=O)O**	75	6
**O=C(O)c1cc(-c2ccoc2)n[nH]1**	75	25
**CO[C@H]1C[C@H](C(=O)O)N(C(C)=O)C1**	74	4
**O=C1CCC(C(=O)O)n2cccc21**	73	4
**O=C(O)C#Cc1ccc2c(c1)OCO2**	73	3
**Cc1nnc(CCCC(=O)O)n1C**	72	4

Structure	P_adj%	unknown_adj%
**O=C(O)c1c[nH]c2ncncc12**	72	16
**C=C(C)CC(C)C(=O)O**	72	2
**CC(C)c1ccc(C(=O)O)c(=O)[nH]1**	72	0
**O=C(O)c1ccc(-n2ccnc2)nn1**	72	4
**CC(C)(C(=O)O)n1cc(Cl)cn1**	71	1
**CCn1cc(CC(=O)O)nn1**	71	28
**O=C(O)Cn1cc(C2CCC2)nn1**	71	0
**O=C(O)c1coc2cccc(O)c12**	71	2
**O=C(O)c1ncnc2sccc12**	68	1
**CN(C)Cc1ccc(C(=O)O)cn1**	67	0
**Cn1cncc1C1CC1C(=O)O**	66	5
**C#CCCN(C)CC(=O)O**	65	6
**CC(C)c1nnsc1C(=O)O**	63	3
**CC1=NNC(=O)C1CCC(=O)O**	62	0
**O=C(O)CNC(=O)c1ccsc1**	59	1
**Cn1cnc2ccc(C(=O)O)nc21**	59	6
**O=C(O)Cn1cc(CCCO)cn1**	57	0
**Cc1cc(O)cc(=O)n1CC(=O)O**	57	24
**CC(Cc1cnc[nH]1)C(=O)O**	57	0
**CC(C)(C)n1nnc(C(=O)O)n1**	56	8
**O=C(O)CCCCc1c[nH]nn1**	56	26
**CC(=O)N1CCCCC1CC(=O)O**	55	21
**CC(C)N(C)C(=O)C1(C(=O)O)CC1**	55	6
**O=C1CCC(C(=O)O)O1**	52	29
**O=C(O)c1nc(=O)[nH][nH]1**	52	5
**O=C(O)C#Cc1cccs1**	52	33
**CN(C)c1nc(O)cc(C(=O)O)n1**	51	49
**CC(C)CC1(C(=O)O)CCNC1=O**	50	2
**O=C(O)c1ccc(-c2nnn[nH]2)cn1**	50	0
**CCC/C=C/C(O)C(=O)O**	48	8
**COc1cccc(C(O)C(=O)O)c1**	47	9
**Cn1nc(C2CC2)nc1CC(=O)O**	46	0
**O=C(O)C#CC1CC(=O)C1**	45	10
**CC(C(=O)O)N(C)CC(F)(F)F**	45	1
**CC(=O)c1c(C)[nH]c(C(=O)O)c1C**	45	55

Structure	P_adj%	unknown_adj%
**O=C1NC2(C(=O)O)CC3CC1C2C3**	42	1
**O=C(O)C1(Nc2ccccc2)CC1**	42	36
**CCc1cccc2c(C(=O)O)c[nH]c12**	41	19
**O=C(O)C1OCCn2ccnc21**	40	7
**CC(C)c1nnnn1CC(=O)O**	39	3
**CC1(C)[C@@H]2CC[C@@]1(C(=O)O)C(=O)C2**	36	4
**O=C1Nc2c(C(=O)O)cccc2C1=O**	36	17
**O=C(O)CCC(=O)c1ccc[nH]1**	35	1
**CC(C)c1nnc(/C=C/C(=O)O)o1**	33	56
**C=CC(C)(CCOC)C(=O)O**	32	1
**CC1C(C(=O)O)C(O)C(=O)N1C**	30	2
**O=C(O)Cn1[nH]c(=O)ccc1=O**	27	0
**COC1(C)CN(CC(=O)O)C1**	22	0
**O=C(O)Cc1csc2nccn12**	20	2
**CN1CCC(C(=O)O)c2ccccc21**	19	2
**O=C(O)C(=O)c1ccc(Cl)s1**	19	14
**CN1N=C(CC(=O)O)CC1=O**	18	0
**CCC(C(=O)O)n1cnc(C#N)n1**	16	0
**COCC(=O)N(C)CC(=O)O**	15	8
**CCn1c(C(=O)O)cnc1O**	13	87
**CC(C)(C(=O)O)N1CCOC1=O**	12	0
**O=C(O)CCc1nc2cccnc2[nH]1**	12	36
**O=C(O)c1nc2cccnc2s1**	11	27
**O=C(O)CC(=O)NCC1CC1**	11	89
**N=c1sccn1CC(=O)O**	8	0
**CC(C)(C)c1nc[nH]c1C(=O)O**	8	21
**CCCCN1CCOCC1C(=O)O**	7	9
**COc1cc(CC(=O)O)on1**	3	29
**Cn1ncnc1COCC(=O)O**	0	1
**CCCc1nc(C)c(C(=O)O)s1**	0	3
**N=C1CCCN1CC(=O)O**	0	6
**CCS(=O)(=O)NCC(=O)O**	0	11
**O=C(O)c1csc2cncn12**	0	26
**CCS(=O)CC(=O)O**	0	49
**Cn1cc(-c2c[nH]cc2C(=O)O)cn1**	0	65

Structure	P_adj%	unknown_adj%
**O=C(O)c1ccnnc1NC1CC1**	0	71
**CC(C)CCCC(=O)C(=O)O**	0	79
**O=C(O)c1cc(=O)n2ncnc2[nH]1**	0	86
**Cc1nn(C)c(C(=O)O)c1[N+](=O)[O-]**	0	86
**Cc1oc([N+](=O)[O-])cc1C(=O)O**	0	100
**CC1=NOC(=O)C1CC(=O)O**	0	100
**CC1=CC(=O)N(CC(=O)O)C1=O**	0	100
**Cc1nc(O)n(CCC(=O)O)c1C**	0	100
**CCCc1noc(CCC(=O)O)n1**	0	100
**O=C(O)c1c[nH]c(=O)s1**	0	100
**CC1(C)NC(=O)N(CC(=O)O)C1=O**	0	0
**CN1C(=O)Cc2ccc(C(=O)O)cc21**	0	0
**CC(O)Cc1c(C(=O)O)[nH][nH]c1=O**	0	1
**Cc1nnc2cc(C(=O)O)ccn12**	0	1
**CN1CCC(C(=O)O)S1(=O)=O**	0	2
**Cc1nc([N+](=O)[O-])cn1CC(=O)O**	0	2
**C[Si](C)(C)C#CCC(=O)O**	0	2
**CC1=NS(=O)(=O)NC=C1C(=O)O**	0	5
**O=C(O)c1cc(=O)c2ccncc2o1**	0	8
**O=C(O)c1cnon1**	0	9
**C=CCCC1(C(=O)O)CC1**	0	9
**CN(C1CCCC1)C(C)(C)C(=O)O**	0	14
**CNC(=O)c1cnn(C)c1C(=O)O**	0	20
**CC1CCN(C(=O)C(=O)O)C1**	0	49
**Cc1[nH]c(=O)[nH]c(=S)c1C(=O)O**	0	91
**CC(OCC(=O)O)c1nccn1C**	0	0
**O=C(O)c1c[nH]c(=S)n1C1CC1**	0	4
**O=C(O)c1ccc(O)c2ncccc12**	0	4
**Cc1cc(NC(=O)C(=O)O)no1**	0	7
**O=C(O)c1ncnc2nc[nH]c12**	0	13
**O=C(O)Cn1cnc2c1CCCC2**	0	20
**CN(C)C(C(=O)O)c1cncn1C**	0	0
**O=C(O)c1[nH]nc(O)c1CCO**	0	0
**CN(CC(=O)O)c1ccc(C#N)cn1**	0	2
**CC(C)(NCCC#N)C(=O)O**	0	38

Structure	P_adj%	unknown_adj%
**CC(NC1(CO)CCCC1)C(=O)O**	0	0
**CCC(C)c1cc(C(=O)O)on1**	0	61
**CN1CCN(CCC(=O)O)CC1**	0	4
**Cc1sc(O)nc1C(=O)O**	0	55
**C#Cc1cccc(NC(C)C(=O)O)c1**	0	19
**O=C(O)CNc1ccc(Cl)nn1**	0	0
**O=C(O)C1CCCc2cccnc21**	0	20
**O=C(O)c1ccc2[nH]nnc2c1O**	0	9
**Cc1ccc(NCCC(=O)O)nc1**	0	12
**CNc1nnc(CC(=O)O)s1**	0	18
**CC(OCc1ccncc1)C(=O)O**	0	19
**CN(C)Cc1ccsc1C(=O)O**	0	16
**Cn1cc(NCC(=O)O)cn1**	0	32
**CC(C(=O)O)n1cnc2ccccc21**	0	1
**O=C(O)COc1ccc(Cl)cn1**	0	100
**Cn1c(C(=O)O)nc2ncccc21**	0	1
**CCn1cc(S(=O)(=O)Cl)cn1**	100	0
**COc1ccc(S(=O)(=O)Cl)cc1**	100	0
**COc1cccc(S(=O)(=O)Cl)c1**	100	0
**O=C(O)c1ccc(S(=O)(=O)Cl)c([N+](=O)[O-])c1**	100	0
**Nc1ncc(S(=O)(=O)Cl)cn1**	100	0
**O=S(=O)(Cl)c1nnc[nH]1**	100	0
**Cn1cnc(S(=O)(=O)Cl)c1**	100	0
**Cn1nncc1S(=O)(=O)Cl**	100	0
**Cc1nc(S(=O)(=O)Cl)cn1C**	100	0
**Cn1ccnc1S(=O)(=O)Cl**	100	0
**CCn1cnc(S(=O)(=O)Cl)c1**	100	0
**N#Cc1cccc(S(=O)(=O)Cl)c1**	100	0
**COc1cncc(S(=O)(=O)Cl)c1**	100	0
**CC(C)n1cc(S(=O)(=O)Cl)cn1**	100	0
**O=[N+]([O-])c1ccc(S(=O)(=O)Cl)c(Cl)c1**	100	0
**O=S(=O)(Cl)c1cc(F)cc(F)c1**	100	0
**Cc1cc(S(=O)(=O)Cl)c(C)o1**	100	0
**COc1cc(C)c(Cl)cc1S(=O)(=O)Cl**	100	0
**Cc1c(S(=O)(=O)Cl)cnn1C**	100	0

Structure	P_adj%	unknown_adj%
**COc1ccc(S(=O)(=O)Cl)cn1**	100	0
**Cc1cc(S(=O)(=O)Cl)cs1**	100	0
**CC(C)n1cnc(S(=O)(=O)Cl)c1**	100	0
**Cc1ccc(S(=O)(=O)Cl)c(C)c1**	100	0
**Cc1ccc(S(=O)(=O)Cl)cc1C**	100	0
**Cc1cc(C)cc(S(=O)(=O)Cl)c1**	100	0
**Cc1ccc(C)c(S(=O)(=O)Cl)c1**	100	0
**O=S(=O)(Cl)c1cc(Cl)cc(Cl)c1**	100	0
**Cc1ccc(S(=O)(=O)Cl)cc1**	100	0
**C=C(C)COc1cccc(S(=O)(=O)Cl)c1**	100	0
**Cc1cccc(S(=O)(=O)Cl)c1**	100	0
**O=S(=O)(Cl)c1ccc(Br)cc1C(F)(F)F**	100	0
**O=C1COc2cc(S(=O)(=O)Cl)ccc2N1**	100	0
**O=S(=O)(Cl)c1cc(F)cc(Cl)c1**	100	0
**C#CCOc1cccc(S(=O)(=O)Cl)c1**	100	0
**O=c1[nH]c2ccc(S(=O)(=O)Cl)cc2s1**	100	0
**C=Cc1ccc(S(=O)(=O)Cl)cc1**	100	0
**O=c1[nH]c(=O)c2cc(S(=O)(=O)Cl)cnc2[nH]1**	100	0
**O=c1ccc2ccc(S(=O)(=O)Cl)cc2[nH]1**	100	0
**Cc1noc(C)c1S(=O)(=O)Cl**	99	0
**O=S(=O)(Cl)c1cc(F)ccc1F**	99	0
**Cc1ccncc1S(=O)(=O)Cl**	99	1
**CCn1cc(S(=O)(=O)Cl)nc1C**	99	1
**O=S(=O)(Cl)C=Cc1ccccc1**	99	1
**CCn1ncc(S(=O)(=O)Cl)c1C**	98	2
**O=S(=O)(Cl)c1cnc2n1CCC2**	98	2
**Cc1cnc(N)c(S(=O)(=O)Cl)c1**	97	3
**O=Cc1ccc(S(=O)(=O)Cl)cc1**	97	3
**COc1ccccc1S(=O)(=O)Cl**	96	4
**N#Cc1ccc(S(=O)(=O)Cl)cc1**	96	4
**CCc1ccc(S(=O)(=O)Cl)cc1**	96	4
**O=C(N1CCc2ccc(S(=O)(=O)Cl)cc2C1)C(F)(F)F**	96	4
**C=C/C=C/S(=O)(=O)Cl**	94	6
**O=S(=O)(Cl)/C=C/c1ccccc1**	94	6
**N#Cc1ncccc1S(=O)(=O)Cl**	92	8

Structure	P_adj%	unknown_adj%
**O=S(=O)(Cl)c1ccc(F)c(Cl)c1**	92	8
**N#Cc1ccc(S(=O)(=O)Cl)cn1**	91	9
**N#Cc1cncc(S(=O)(=O)Cl)c1**	80	20
**Cc1ccc(S(=O)(=O)Cl)cc1C(=O)O**	75	25
**Cc1ccccc1S(=O)(=O)Cl**	73	1
**COc1ccncc1S(=O)(=O)Cl**	72	28
**CC(=O)OCCS(=O)(=O)Cl**	56	0
**CC(C)(C#N)CS(=O)(=O)Cl**	51	8
**CCCCS(=O)(=O)Cl**	43	1
**O=S(=O)(Cl)CCOCC1CC1**	40	10
**CC(C)(C)OCCS(=O)(=O)Cl**	38	2
**CCC1(S(=O)(=O)Cl)CC1**	28	7
**CS(=O)(=O)Cl**	25	0
**COCC1(S(=O)(=O)Cl)CC1**	24	76
**COC(=O)CCCS(=O)(=O)Cl**	24	2
**O=S(=O)(Cl)CC1CCOCC1**	21	0
**O=S(=O)(Cl)C1CCCCCC1**	20	23
**CN1CCN(S(=O)(=O)Cl)CC1**	19	0
**CC1(CS(=O)(=O)Cl)N=N1**	18	7
**CC(=O)OCCCS(=O)(=O)Cl**	18	3
**CC(C)C1(S(=O)(=O)Cl)CC1**	18	30
**O=S(=O)(Cl)c1cn[nH]c1**	17	4
**CC(C)(C)CS(=O)(=O)Cl**	10	3
**O=S(=O)(Cl)CC1CCCO1**	5	4
**O=S(=O)(Cl)N1CCCOCC1**	4	3
**CCN(CC1CC1)S(=O)(=O)Cl**	0	6
**CCN(C1CC1)S(=O)(=O)Cl**	0	11
**COC1(CS(=O)(=O)Cl)CCC1**	0	20
**CO[C@@H]1CCC[C@@H]1S(=O)(=O)Cl**	0	22
**COC(C)(C)CCS(=O)(=O)Cl**	0	39
**N#Cc1ccccc1S(=O)(=O)Cl**	0	100
**O=C1C=C(S(=O)(=O)Cl)C(=O)O1**	0	100
**COC(=O)/C=C/S(=O)(=O)Cl**	0	100
**O=[N+]([O-])c1ccccc1S(=O)(=O)Cl**	0	100
**Cn1c(=O)oc2ccc(S(=O)(=O)Cl)cc21**	0	100

Structure	P_adj%	unknown_adj%
**CCOC(=O)CS(=O)(=O)Cl**	0	2
**O=C(O)c1cc(S(=O)(=O)Cl)c[nH]1**	0	2
**COC(=O)NS(=O)(=O)Cl**	0	2
**O=C1OCCN1S(=O)(=O)Cl**	0	3
**CN(C)C1(CS(=O)(=O)Cl)CC1**	0	3
**CCN(CC)CCS(=O)(=O)Cl**	0	3
**COC(=O)CCS(=O)(=O)Cl**	0	4
**COC(=O)C(C)(C)S(=O)(=O)Cl**	0	4
**O=C1CCC(S(=O)(=O)Cl)CC1**	0	4
**O=S(=O)(Cl)N1CCCCCC1**	0	5
**CCOC(=O)CCS(=O)(=O)Cl**	0	5
**O=S(=O)(Cl)[C@@H]1CCC[C@H]1O**	0	5
**COCCOCCS(=O)(=O)Cl**	0	14
**CC(C)(C)NS(=O)(=O)Cl**	0	3
**N#CCC1(CS(=O)(=O)Cl)CC1**	0	3
**CC(C)OC(=O)CS(=O)(=O)Cl**	0	3
**O=S(=O)(Cl)c1ccccc1Cn1cncn1**	0	4
**C=CCS(=O)(=O)Cl**	0	5
**CN(C)S(=O)(=O)Cl**	0	6
**COCC(C)(C)CS(=O)(=O)Cl**	0	7
**CC(C)(C#N)S(=O)(=O)Cl**	0	10
**CC1(CS(=O)(=O)Cl)COC1**	0	2
**CC1CCN(S(=O)(=O)Cl)CC1**	0	3
**CC(C)(C)OC(=O)N1CCC(S(=O)(=O)Cl)CC1**	0	4
**CO[C@H]1COC[C@H]1S(=O)(=O)Cl**	0	0
**CO[C@@H]1CCC[C@H]1S(=O)(=O)Cl**	0	3
**Cc1ccc(O)c(S(=O)(=O)Cl)c1**	0	8
**CC(C)(C)S(=O)(=O)Cl**	0	0
**O=S(=O)(Cl)Cc1ccc(Cl)cc1Cl**	0	3
**O=S(=O)(Cl)c1nc2ccccc2s1**	0	4
**Cc1ccccc1CS(=O)(=O)Cl**	0	3
**Cc1ccc(CS(=O)(=O)Cl)cc1**	0	3
**Cc1cccc(CS(=O)(=O)Cl)c1**	0	3
**Cc1cccc(S(=O)(=O)Cl)c1C**	0	3
**COc1ccc(S(=O)(=O)Cl)c(C)c1C**	0	4

Structure	P_adj%	unknown_adj%
**O=[N+]([O-])c1cc(S(=O)(=O)Cl)ccc1Cl**	0	100
**O=Cc1c[nH]nn1**	100	0
**CCCC=O**	100	0
**O=CC1CCC1**	100	0
**CC(C)(C)OC(=O)N1CC(C=O)C1**	100	0
**O=CCC1CCOCC1**	100	0
**O=CCOCc1ccccc1**	100	0
**N#Cc1ccc(CCC=O)cc1**	100	0
**NS(=O)(=O)c1cc(C=O)co1**	100	0
**O=Cc1nccn1-c1ccccc1**	100	0
**CS(=O)(=O)c1ccc(C=O)o1**	100	0
**Cc1ccncc1CCC=O**	100	0
**Cc1cccc(C=O)n1**	100	0
**O=Cc1cn(-c2ccccc2)cn1**	100	0
**Cn1cc(-c2cccc(C=O)c2)cn1**	100	0
**O=Cc1ccc(-c2ccn[nH]2)o1**	100	0
**N#CCOc1ccccc1C=O**	100	0
**O=Cc1ccn(-c2cccnc2)c1**	100	0
**O=Cc1nc(-c2ccccc2)c[nH]1**	100	0
**CC(C)(CC=O)C1CC1**	99	0
**O=Cc1cn[nH]c1**	99	0
**O=Cc1csnn1**	99	0
**O=CC(=O)O**	99	1
**Cc1cc(C=O)cnc1-n1cccn1**	99	0
**O=Cc1ccnn1-c1ccccn1**	99	0
**CCCn1ccc(C=O)n1**	99	0
**O=Cc1ccccc1-n1cccn1**	99	0
**Cc1cccc(-c2[nH]ncc2C=O)c1**	99	0
**O=Cc1cc(C(=O)O)ccc1O**	98	0
**O=Cc1cccc(Cc2c[nH]cn2)c1**	98	0
**O=Cc1cnn(Cc2ccccc2)c1**	98	0
**CNC(=O)c1ccc(C=O)cc1**	98	0
**CS(=O)c1ccc(C=O)cc1**	98	0
**O=Cc1ccc(-n2ccnc2)cc1**	98	0
**O=Cc1ccc(-n2ccnc2)nc1**	98	0

Structure	P_adj%	unknown_adj%
**O=Cc1ccc(C(=O)NC2CC2)cc1**	98	2
**Cn1cc(C=O)cc1C#N**	98	0
**Cn1cc(-n2ccc(C=O)c2)cn1**	98	2
**O=Cc1ccc2cn[nH]c2c1**	98	2
**O=Cc1cnc[nH]1**	97	1
**CS(=O)(=O)c1cccc(C=O)c1**	97	0
**COc1ncccc1C=O**	97	0
**COC(=O)c1cc(C=O)cn1C**	97	3
**O=Cc1cn(-c2ccccc2)nn1**	97	3
**Cc1nn(-c2ccncc2)cc1C=O**	97	3
**CC(C)(O)c1ccc(C=O)cc1**	97	0
**C#Cc1cccc(C=O)c1**	97	3
**Cn1cc(C=O)cn1**	96	0
**Cc1nc2ccccc2nc1C=O**	96	0
**O=Cc1ccc2c(n1)NC(=O)CO2**	96	0
**O=Cc1n[nH]cc1-c1ccccc1**	96	2
**O=Cc1cc(CO)ccc1O**	95	0
**COC(=O)CCC=O**	95	5
**O=CC1CC2(CC2)C1**	95	0
**COc1cncc(CCC=O)c1**	95	5
**COC(C)(C)CC=O**	95	5
**NS(=O)(=O)c1ccc(C=O)o1**	94	5
**COc1cccnc1C=O**	94	3
**O=Cc1ccc(-c2cccnc2)o1**	94	6
**Cc1ccc2cc(C=O)ccc2n1**	94	6
**O=Cc1ccccc1-n1cccc1**	94	0
**CCc1ccc(C=O)cc1[N+](=O)[O-]**	94	0
**O=Cc1cc(C2CC2)n[nH]1**	94	5
**CC(=O)OCc1ccc(C=O)o1**	93	7
**CC(C)(C)c1ccc(CC=O)cc1**	93	1
**Cc1ncc(C=O)n1-c1ccccc1**	93	0
**CC(C)(C#N)c1ccc(C=O)cc1**	93	5
**COC(=O)c1[nH]cc(C=O)c1C**	93	3
**Cc1ccccc1-n1cc(C=O)cn1**	93	6
**Cn1cc(C=O)c(-c2cccnc2)n1**	93	5

Structure	P_adj%	unknown_adj%
**O=Cc1ccnc(-c2ccccc2)n1**	93	7
**O=CC1CCCCCCC1**	92	0
**COc1cccc2cc(C=O)oc12**	92	8
**COC(=O)c1ccc(C=O)o1**	92	8
**Cc1cc(C)c(C(=O)O)cc1C=O**	92	4
**COc1nc(C)ccc1C=O**	92	6
**O=Cc1c[nH]c(-c2ccccc2)n1**	92	6
**O=Cc1cc(-n2ccnc2)cs1**	92	6
**O=Cc1cnn(-c2ccccn2)c1**	92	7
**O=Cc1ccc2cccc(O)c2n1**	92	8
**O=CC1CCCCCC1**	91	0
**O=Cc1cccc(-c2ccccn2)c1**	91	9
**O=Cc1ccc(O)c(F)c1**	90	0
**COc1nccc(C=O)n1**	90	8
**O=Cc1cn2c(n1)CCC2**	90	1
**O=Cc1cccc2ccoc12**	90	7
**N#CCCCOc1cccc(C=O)c1**	90	7
**Cc1[nH]nc(Cl)c1C=O**	89	0
**CCOC(=O)c1cc(C=O)[nH]n1**	89	3
**O=Cc1cc(-c2ccccn2)n[nH]1**	89	11
**O=CC[C@@H](O)[C@@H](O)CO**	88	0
**O=CC[C@H](O)[C@H](O)CO**	88	0
**COC(=O)c1cccc(C=O)n1**	88	12
**COc1nc2ccccc2cc1C=O**	88	5
**O=Cc1cccc(-c2nn[nH]n2)c1**	88	3
**Cn1nc(-c2ccccc2)cc1C=O**	88	1
**O=Cc1ccc(-c2nn[nH]n2)cc1**	87	0
**O=Cc1occc1C(=O)O**	86	0
**Cn1cnc(C=O)c1**	86	0
**NS(=O)(=O)c1ccc(C=O)cc1**	86	11
**CC(C)(C)c1cc(O)cc(C=O)c1**	86	11
**O=Cc1c[nH]nc1-c1ccccc1**	86	6
**Cc1cc(C=O)cc2cccnc12**	86	12
**O=Cc1ccn(-c2ccccc2)n1**	86	14
**O=Cc1c[nH]nc1-c1cccs1**	85	15

Structure	P_adj%	unknown_adj%
**O=CC[C@@H](O)[C@H](O)[C@H](O)CO**	84	0
**Cc1ccc(CCC=O)cn1**	84	0
**CCOc1cc(C=O)ccc1OC**	84	0
**O=CC1CC1**	83	0
**O=Cc1cc(C(F)(F)F)[nH]n1**	83	6
**O=Cc1ccc2c(c1)CCC2**	80	0
**N#Cc1csc(C=O)c1**	80	0
**O=Cc1cc(O)c2ccsc2c1**	80	8
**O=Cc1ccnc(O)c1**	79	0
**Cc1c(C(F)(F)F)n[nH]c1C=O**	79	4
**O=Cc1cc(O)c2ccoc2c1**	77	21
**O=Cc1cc2cc[nH]c2cn1**	76	9
**CCCn1cc(C=O)c(C2CC2)n1**	76	0
**O=Cc1ccc2nccnc2c1**	75	0
**COc1ccc(C=O)c(OC)c1**	73	16
**O=C[C@H](O)CO**	72	0
**O=Cc1cc(Cl)c(O)cc1F**	71	26
**Cn1nccc1-c1ccccc1C=O**	71	3
**O=Cc1ccc([N+](=O)[O-])cn1**	69	8
**Cn1cc(-c2ncc(C=O)cn2)cn1**	67	15
**CC(C)c1cc(O)ccc1C=O**	66	0
**COc1cc(Cl)cc(C=O)c1O**	62	2
**CN(CCO)c1ccc(C=O)cn1**	62	0
**Cc1ccc(C=O)c2ccccc12**	61	11
**O=Cc1cc[n+]([O-])cc1**	60	1
**N#CC1(C=O)CC1**	60	4
**N#Cc1ccc(C=O)s1**	60	0
**COc1cc(C)c(C=O)cc1C**	59	0
**O=Cc1ccc(N2CCCC2)nc1**	59	0
**CCCCc1nc(C=O)c(Cl)[nH]1**	59	0
**O=Cc1nccn1Cc1ccccc1**	57	1
**O=Cc1ccc(O)nc1**	56	0
**CCOC(=O)c1[nH]c(C=O)cc1C**	56	2
**CC(C)C=O**	54	1
**Nc1ccc2cc(C=O)ccc2n1**	54	3

Structure	P_adj%	unknown_adj%
**O=Cc1cnc(N2CCCC2)nc1**	53	0
**COc1ccc(C=O)cc1C#N**	51	2
**O=Cc1cc2sccc2s1**	50	0
**O=Cc1ncc[nH]1**	49	0
**CS(=O)(=O)n1cccc1C=O**	47	0
**O=C[C@H](O)[C@H](O)CO**	44	11
**CC1(C=O)COC1**	44	0
**Cn1cc(-c2n[nH]cc2C=O)cn1**	41	59
**O=Cc1cc(F)c2[nH]ccc2c1**	39	8
**O=Cc1ccc2c(c1)CCCC2**	36	1
**O=Cc1cc2ccccc2[nH]1**	36	0
**Cc1occc1C=O**	35	9
**O=Cc1c[nH]c(C(F)(F)F)c1**	35	65
**C[C@]1(C=O)C[C@@H]1c1ccccc1**	33	0
**Nc1ncncc1C=O**	32	1
**COc1cc(C=O)oc(=O)c1**	32	0
**COCc1ccc(C=O)cc1**	28	8
**Nc1ncccc1C=O**	28	0
**O=Cc1cncc2ccccc12**	27	0
**Cc1cc(O)c(C=O)cc1C**	27	3
**O=Cc1ccn(Cc2ccccc2)c1**	27	1
**Cc1nn(C2CCCC2)cc1C=O**	26	0
**CN(C)c1ccncc1C=O**	26	0
**N#Cc1cccc(C=O)c1O**	25	2
**Cn1cc(CCC=O)cn1**	25	4
**O=Cc1c(O)cccc1[N+](=O)[O-]**	25	0
**O=Cc1cccc2ccc(O)cc12**	25	11
**O=Cc1c[nH]c2ncccc12**	18	1
**O=Cc1cccc2c1NCC2**	17	0
**COc1ccc(F)c(C=O)n1**	16	3
**Nc1ccc(C=O)cn1**	16	39
**Cc1nc(F)ccc1C=O**	16	10
**Cc1ccc2ccccc2c1C=O**	16	0
**Cc1ncc(C=O)c(N(C)C)n1**	16	14
**Cc1ccc(C=O)c(N)n1**	16	3

Structure	P_adj%	unknown_adj%
**O=Cc1cc[nH]c1**	15	0
**O=Cc1nccc2ccccc12**	15	3
**Cc1ncc(C=O)c(N)n1**	14	0
**O=Cc1cnc(O)nc1O**	14	8
**CC(C)(C)C(=O)CC=O**	14	17
**COc1ncncc1C=O**	13	0
**Cn1cc(C=O)c2ccccc21**	13	0
**O=Cc1cnon1**	12	4
**COc1ccnc(C=O)n1**	12	0
**O=Cc1scc2c1OCCO2**	12	0
**Cc1cc(C=O)ncn1**	11	6
**CN(C)c1nccc(C=O)n1**	11	1
**O=CCc1ccc(F)cc1Cl**	11	3
**Cc1cc(C=O)ccc1Cl**	11	0
**O=Cc1c(F)cccc1Cl**	10	0
**Cc1cc(C)c(C)c(C=O)c1C**	10	0
**Cc1cc(C)c(C=O)c(C)c1**	10	0
**Cc1ccc(C=O)nn1**	8	16
**Cc1ccc2[nH]cc(C=O)c2c1**	8	2
**CC(C)(C=O)c1ccccc1**	6	0
**Cc1ccccc1-n1nccc1C=O**	6	0
**Cn1cccc1C=O**	6	1
**CCn1nc(C=O)c2ccccc21**	5	0
**CC(=O)Nc1ccccc1C=O**	4	1
**CCN(CC)c1ncc(C=O)s1**	4	1
**CC(C)(C)c1ncc(C=O)cn1**	3	0
**N#Cc1ccc2[nH]cc(C=O)c2c1**	3	0
**COc1ccc2[nH]cc(C=O)c2c1**	3	0
**N#Cc1ccc(C=O)nc1**	0	4
**CCOCc1ccc(C=O)o1**	0	9
**CCc1ccc(C=O)cn1**	0	17
**O=CCCc1ccccc1CO**	0	25
**CN(C)c1ccc(C=O)o1**	0	48
**COc1ccc(C=O)nc1**	0	100
**N#Cc1ccc(C=O)o1**	0	100

Structure	P_adj%	unknown_adj%
**CC(C)c1nn(C)c(Cl)c1C=O**	0	100
**COc1cccc(C=O)c1Cl**	0	100
**Cc1ccc2occ(C=O)c(=O)c2c1**	0	100
**Cc1c[nH]nc1C=O**	0	100
**O=Cc1cnc2[nH]ccc2c1**	0	100
**O=CC1CCOCC1**	0	100
**CN(C)C(C)(C)C=O**	0	0
**O=Cc1ccc(C(F)(F)F)[nH]1**	0	0
**Cn1c(C=O)cnc1Cl**	0	0
**Cc1cccc(C=O)c1OC(F)F**	0	0
**O=Cc1ccsc1Cl**	0	0
**CCn1cnc(Cl)c1C=O**	0	0
**COc1nc(Cl)c(C=O)n1C**	0	0
**Cn1ncc(C=O)c1Cl**	0	1
**CC(C)n1cncc1C=O**	0	1
**Cc1cc(C=O)ccc1C#N**	0	1
**O=Cc1cnc(C2CC2)nc1**	0	1
**N[C@@H](C=O)[C@@H](O)[C@H](O)[C@H](O)CO**	0	2
**O=Cc1ccc([N+](=O)[O-])o1**	0	3
**O=Cc1cscc1Cl**	0	5
**C#Cc1ccsc1C=O**	0	10
**Cn1ncc(C=O)c1C1CC1**	0	11
**CC(C)(C=O)N1CCOCC1**	0	0
**C#CCn1cccc1C=O**	0	0
**COc1ccc(C)cc1C=O**	0	0
**CCc1cccc(C=O)c1O**	0	0
**CC(C)(C=O)Oc1ccccc1**	0	0
**O=CCCC1CC1**	0	0
**Cc1nccc(C=O)n1**	0	0
**CCCn1ccc(C=O)c1**	0	0
**CCc1nn(C)c(OC)c1C=O**	0	0
**COC(=O)c1ccsc1C=O**	0	0
**O=Cc1cscc1C(F)(F)F**	0	0
**Cc1cc(Cl)cc(C=O)c1O**	0	0
**O=Cc1cccn1C1CC1**	0	0

Structure	P_adj%	unknown_adj%
**O=Cc1ccc(Cl)nn1**	0	0
**CCc1cccc(F)c1C=O**	0	0
**CCC(C)(C=O)OC**	0	0
**COCCCn1nccc1C=O**	0	0
**O=Cc1ccc(Cl)o1**	0	0
**O=Cc1ccnc(Cl)c1F**	0	0
**Cc1ccc(F)cc1C=O**	0	0
**CC(C)Cn1nccc1C=O**	0	0
**O=Cc1sccc1Cl**	0	0
**CCc1cc(OC)ccc1C=O**	0	0
**O=Cc1ccnn1C1CCCC1**	0	0
**CCOc1cccc(C=O)n1**	0	0
**Cc1ccoc1C=O**	0	0
**N#Cc1ccc(C=O)c(F)c1**	0	0
**N#Cc1c(F)ccc(C=O)c1Cl**	0	0
**CCCn1nc(C)c(Cl)c1C=O**	0	0
**O=Cc1ccccc1N1CCCCC1**	0	0
**Cc1ccc(C=O)nc1**	0	0
**CC(C)Oc1ccccc1C=O**	0	0
**O=Cc1nccnc1Cl**	0	0
**O=Cc1ccncc1[N+](=O)[O-]**	0	0
**CC(C)(C=O)CN1CCOCC1**	0	0
**COCCOc1ccccc1C=O**	0	1
**COC(=O)C1(C=O)CC1**	0	1
**O=C[C@H](O)[C@H]1OC(=O)[C@@H](O)[C@H]1O**	0	1
**COc1nccnc1C=O**	0	2
**CC(C)(O)C=O**	0	2
**O=CCC1CCCOC1**	0	3
**Cn1nc(C2CC2)cc1C=O**	0	3
**COc1ccc(F)c(C=O)c1**	0	3
**O=Cc1ncccc1C(F)(F)F**	0	3
**COC1(C=O)CCOC1**	0	3
**Cc1ccc(O)c(C=O)n1**	0	4
**CC(C)(C)C(=O)C=O**	0	4
**Cn1cc(C=O)c(Cl)n1**	0	5

Structure	P_adj%	unknown_adj%
**CCn1cc(C=O)c(C)n1**	0	5
**Cc1cc(C=O)cc(Cl)n1**	0	6
**COCC=O**	0	19
**Cc1ncccc1C=O**	0	61
**O=Cc1ccccc1C1CC1**	0	0
**O=Cc1ccc([N+](=O)[O-])s1**	0	0
**Cc1oc2ccccc2c1C=O**	0	0
**Cn1ncc(Cl)c1C=O**	0	0
**CCCc1ccc(C=O)s1**	0	0
**O=Cc1c(O)ccc(Cl)c1F**	0	0
**CC(C)CCn1cccc1C=O**	0	0
**Cc1c(C=O)cnn1C(C)C**	0	0
**CCn1ccnc1C=O**	0	0
**COCC1(C=O)CCC1**	0	0
**COC(C)(C=O)C1CC1**	0	0
**O=CC1(F)CC1**	0	0
**O=Cc1ccc(Cl)s1**	0	0
**O=CC1(C(F)F)CC1**	0	0
**C[C@H](O)[C@@H](O)[C@@H](O)[C@H](O)C=O**	0	0
**C[C@H](O)[C@H](O)[C@@H](O)[C@@H](O)C=O**	0	0
**N#Cc1cccc(C=O)n1**	0	1
**Cc1ccc2oc(C=O)cc2c1**	0	1
**COc1cc(C)c(C=O)c(C)c1C**	0	1
**CCOCCC=O**	0	2
**COc1cc(O)ccc1C=O**	0	2
**C#Cc1ccc(C=O)cc1**	0	3
**COc1nc(C=O)ccc1C**	0	5
**N#Cc1cnc(Cl)c(C=O)c1**	0	5
**C[C@@H](O)[C@@H](O)[C@H](O)[C@H](O)C=O**	0	7
**O=CC1C[C@@H]2CC[C@H]1O2**	0	22
**CCOc1ccc(Cl)cc1C=O**	0	0
**CN(C)c1ncc(C=O)n1C**	0	0
**O=Cc1ccc(OCC2CC2)cc1**	0	0
**C[C@@H](O)[C@H](O)[C@H](O)[C@@H](O)C=O**	0	0
**O=Cc1c(O)cc(O)cc1O**	0	0

Structure	P_adj%	unknown_adj%
**O=Cc1cnc(Cl)nc1**	0	10
**C#CCn1ccnc1C=O**	0	0
**COc1cc(C)nc(C=O)c1**	0	0
**COc1cc(C)c(C=O)c(O)c1**	0	0
**CN(C)c1ncccc1C=O**	0	0
**Cn1c(Cl)cnc1C=O**	0	0
**Nc1ncc(C=O)c(N)n1**	0	0
**CC(C)(C)C=O**	0	1
**O=CC1CCCOC1**	0	2
**CC1(C)[C@H]2C[C@@](C)(C=O)C[C@H]21**	0	0
**Cc1cc(C=O)cs1**	0	2
**Cc1c(O)cc(O)c(C=O)c1O**	0	9
**Cn1cc(C=O)c(C(C)(C)C)n1**	0	3
**Cc1ccncc1C=O**	0	5
**CC(C)(C=O)S(C)(=O)=O**	0	3
**O=Cc1cc(O)c(O)cc1O**	0	7
**O=CC1(c2ccccc2)CCOC1**	0	9
**CC(C)C(C#N)C=O**	0	6
**O=Cc1ccc(O)c(O)c1O**	0	0
**CCc1ccc(C=O)o1**	0	7
**CC1=C(C=O)C(C)(C)CC=C1**	0	0
**Cc1nc(C)c(C=O)c(N)n1**	0	4
**CC(C)c1ccc(CC=O)cc1**	0	30
**Cc1ccccc1CC=O**	0	39
**Cc1cc(O)cc(O)c1C=O**	0	9
**O=CC1=COCCC1**	0	11
**O=Cc1ccc(O)c2ncccc12**	0	1
**Cc1[nH]c2ccc(F)cc2c1C=O**	0	1
**Cc1c(C=O)c2ccccn2c1C#N**	0	0
**O=Cn1nnc2ccccc21**	0	0
**CCc1cccc2c(C=O)c[nH]c12**	0	2
**O=Cc1ccc(O)c2ccccc12**	0	4
**CN(C)c1ncnc(Cl)c1C=O**	0	0

8.3. Suzuki coupling building block validation data

In the same manner as outlined in Supplementary Section 8.2, the abridged Suzuki coupling validation data shown below only reports the key calculated factors of adjusted %AUC product (or derivatives) and adjusted %AUC unknown DNA species, in both cases correcting for any residual small molecule building block peaks integrated in the LCMS data.

Scores were assigned from 1 to 4, indicating a preference for use of a given building block across ArI and ArBr validation campaigns according to the below hierarchical rules:

((ArI_adj%P >= 70) AND (ArBr_adj%P >= 70)) AND ((ArI_unknown_adj% <= 10) AND (ArBr_unknown_adj% <= 10)) => 4

(((ArI_adj%P >= 70) AND (ArBr_adj%P >= 50)) XOR ((ArI_adj%P >= 50) AND (ArBr_adj%P >= 70))) AND ((ArI_unknown_adj% <= 10) AND (ArBr_unknown_adj% <= 10)) => 3

((ArI_adj%P >= 70) AND (ArBr_adj%P >= 70)) AND ((ArI_unknown_adj% <= 10) XOR (ArBr_unknown_adj% <= 10)) => 2

NOT MISSING Structure => 1

Supplementary Table 13 | Suzuki coupling building block validation data

Structure	ArI_adj %P	ArI_unknown_adj%	ArBr_adj %P	ArBr_unknown_adj%	Score
**O=C(O)COc1cc(F)cc(B(O)O)c1**	100	0	96	0	4
**CN(C)CCc1ccc(B(O)O)cc1**	100	0	94	0	4
**Cn1c(=O)[nH]c2ccc(B3OC(C)(C)C(C)(C)O3)cc21**	100	0	92	0	4
**OB(O)c1ccc2c(c1)OCO2**	100	0	92	0	4
**CC1(C)OB(c2ccc3c(c2)COC(=O)N3)OC1(C)C**	100	0	87	0	4
**Cn1nccc1B(O)OC(C)(C)C(C)(C)O**	100	0	84	0	4
**COC(=O)c1ccc(B(O)O)c(F)c1**	100	0	81	0	4
**CC(C)(C)C1CC=C(B(O)O)CC1**	100	0	76	0	4
**COc1c(C)cc(B(O)O)cc1C**	99	0	99	0	4
**CCOC(=O)c1cccc(B(O)O)c1**	99	0	94	1	4
**OB(O)c1cccc(-c2ccccc2)c1**	99	0	93	0	4
**Nc1cccc(B(O)O)c1**	99	0	91	1	4
**COC(=O)CCc1cccc(B(O)O)c1**	99	0	90	0	4
**CCCOc1ccc(B(O)O)cc1Cl**	99	0	85	0	4

Structure	ArI_adj %P	ArI_unknown_adj%	ArBr_adj %P	ArBr_unknown_adj%	Score
**CC(C)c1cccc(B(O)OCCO)c1**	99	0	84	0	4
**OB(O)c1ccc2ccccc2c1**	99	0	81	0	4
**CC1(C)OB(c2cc3ccccc3o2)OC1(C)C**	99	0	75	0	4
**CC(=O)Nc1cccc(B(O)O)c1**	98	0	100	0	4
**COc1ccc2cc(B(O)O)ccc2c1**	98	0	97	0	4
**OCc1cccc(B(O)O)c1**	98	0	97	0	4
**Cc1cc(B(O)O)ccc1Cl**	98	0	97	1	4
**CN(C)Cc1cc(B(O)O)ccc1F**	98	0	95	0	4
**COc1cc(B(O)O)ccc1C**	98	0	95	0	4
**CNC(=O)c1ccc(B(O)O)cc1**	98	0	94	0	4
**O=C(O)c1ccc(B(O)O)cc1Cl**	98	0	94	0	4
**CC1(C)OB(c2cnc(N)nc2)OC1(C)C**	98	0	93	0	4
**Cc1ccc(B(O)O)cc1Cl**	98	0	91	0	4
**OB(O)c1cccc(F)c1**	98	0	91	0	4
**Cc1ccc(B(O)O)cc1**	98	0	90	0	4
**COc1ccc(B(O)O)cc1Cl**	98	0	90	8	4
**CC(C)Cc1ccc(B(O)O)cc1**	98	0	88	0	4
**CCc1cccc(B(O)O)c1**	98	0	88	0	4
**Cc1cc(B(O)O)ccc1F**	98	0	88	0	4
**Cc1ccc2cc(B(O)O)ccc2n1**	98	0	88	0	4
**CC(C)(C)OCc1ccc(B(O)O)cc1**	98	0	87	0	4
**OCc1ccc(B(O)O)cc1**	98	0	87	1	4
**[2H]c1c([2H])c([2H])c(B(O)O)c([2H])c1[2H]**	98	0	87	1	4
**CC(C)Oc1ccc(B(O)O)cc1F**	98	0	86	0	4
**O=C(O)c1ccc(B(O)O)c(Cl)c1**	98	0	85	0	4
**CCOc1ccc(B(O)O)cc1C**	98	0	83	0	4
**CC(C)(C)c1cccc(B(O)O)c1**	98	0	82	1	4
**COC(=O)c1cc(B2OC(C)(C)C(C)(C)O2)ccc1C**	98	0	81	0	4
**OB(O)c1ccc(-c2ccccc2)cc1**	98	0	79	3	4
**OB(O)c1cccc2ccccc12**	98	0	78	0	4
**CCCCc1ccc(B(O)O)cc1**	98	0	71	0	4
**O=C(O)Cc1cccc(B(O)O)c1**	97	0	98	0	4

Structure	ArI_adj %P	ArI_unknown_adj%	ArBr_adj %P	ArBr_unknown_adj%	Score
**OB(O)c1ccc(Cl)c(Cl)c1**	97	0	98	0	4
**Cc1cccc(B(O)O)c1**	97	0	96	0	4
**O=C(O)c1cc(B(O)O)ccc1Cl**	97	0	96	0	4
**CNC(=O)c1ccc(B2OC(C)(C)C(C)(C)O2)cc1C**	97	1	96	1	4
**OB(O)c1ccc2c(c1)CCC2**	97	0	94	0	4
**OB(O)c1cccc(Oc2ccccc2)c1**	97	0	94	0	4
**CCn1ccc(B2OC(C)(C)C(C)(C)O2)n1**	97	0	92	0	4
**COc1cc(B(O)O)ccc1Cl**	97	0	91	3	4
**COc1ccc(B(O)O)cc1F**	97	0	90	0	4
**COc1cc(B(O)O)cc(C(=O)O)c1**	97	0	90	7	4
**Cc1cc(C)cc(B(O)O)c1**	97	0	89	0	4
**OB(O)c1ccc(F)c(C(F)(F)F)c1**	97	0	89	0	4
**O=C(O)c1cccc(B(O)O)c1**	97	0	89	3	4
**CC1(C)OB(c2ccc(C3(CO)CC3)cc2)OC1(C)C**	97	1	88	0	4
**CCCc1ccc(B(O)O)cc1**	97	0	86	0	4
**CCc1ccc(B(O)O)cc1**	97	0	86	0	4
**CC1(C)OB(c2cccc(Cn3cccn3)c2)OC1(C)C**	97	0	84	1	4
**CC(=O)c1cccc(B(O)O)c1**	97	0	79	0	4
**COC(=O)c1cc(F)cc(B(O)O)c1**	97	0	75	0	4
**COc1ccc(C(C)C)cc1B(O)O**	97	0	72	0	4
**O=C(O)c1cc(Cl)cc(B(O)O)c1**	96	0	100	0	4
**CC1(C)OB(c2cnn(-c3ccncc3)c2)OC1(C)C**	96	0	99	0	4
**OB(O)c1cc(Cl)cc(Cl)c1**	96	0	98	0	4
**COc1cc(F)cc(B(O)O)c1**	96	0	95	0	4
**COc1cc(OC)cc(B(O)O)c1**	96	0	95	0	4
**CCNC(=O)c1cccc(B(O)O)c1**	96	0	95	3	4
**OB(O)c1cc(F)cc(Cl)c1**	96	0	93	0	4
**COc1ccc(B(O)O)cc1C**	96	0	93	1	4
**O=C(O)c1ccc(B(O)O)cc1F**	96	0	93	1	4

Structure	ArI_adj %P	ArI_unknown_adj%	ArBr_adj %P	ArBr_unknown_adj%	Score
**CC1(C)OB(c2ccc3c(c2)CCC(=O)N3)OC1(C)C**	96	1	93	0	4
**CCOc1cccc(B(O)O)c1**	96	0	92	2	4
**CC1(C)OB(c2cccc(S(N)(=O)=O)c2)OC1(C)C**	96	4	90	10	4
**Cc1cccc(B(O)O)c1C**	96	0	88	0	4
**OB(O)c1ccc(Oc2ccccc2)cc1**	96	0	85	0	4
**COc1ccc(F)c(B(O)O)c1**	96	0	83	0	4
**OB(O)c1cccc(OC(F)(F)F)c1**	96	0	82	0	4
**CC1(C)OB(C2=CCNCC2)OC1(C)C**	96	3	76	6	4
**CC1(C)OB(c2cnc3occc3c2)OC1(C)C**	96	0	75	0	4
**OB(O)c1cccc(Cl)c1**	95	0	98	0	4
**OCc1ccccc1B(O)O**	95	0	98	0	4
**OCCc1cccc(B(O)O)c1**	95	0	94	1	4
**OB(O)c1ccc(F)c(Cl)c1**	95	2	92	5	4
**COc1ncccc1B(O)O**	95	0	91	0	4
**O=C(O)CCc1cccc(B(O)O)c1**	95	0	89	1	4
**CC1(C)OB(c2ccc3c(c2)CCOC3)OC1(C)C**	95	1	87	0	4
**Cc1cc(F)ccc1B(O)O**	95	0	86	0	4
**NC(=O)c1ccc(B(O)O)cc1**	95	5	84	2	4
**COc1ccc(Cl)cc1B(O)O**	95	0	76	0	4
**O=C(O)c1cccc(B(O)O)c1F**	95	5	71	0	4
**Nc1cc(B(O)O)ccc1F**	94	0	100	0	4
**CCNC(=O)c1ccc(B(O)O)cc1**	94	0	97	1	4
**COc1ccc(C#N)cc1B(O)O**	94	0	96	1	4
**O=C(NC1CC1)c1cccc(B(O)O)c1**	94	0	95	0	4
**OB(O)c1ccc2c(c1)CCCO2**	94	0	94	0	4
**COc1cc(C(=O)O)ccc1B(O)O**	94	0	92	3	4
**CC(=O)c1ccc(B(O)O)cc1**	94	0	92	4	4
**OB(O)c1ccc(Cl)cc1**	94	0	90	1	4
**Cc1ccc(B(O)O)c(F)c1**	94	0	89	0	4
**OCc1ccc(Cl)c(B(O)O)c1**	94	0	89	0	4

Structure	ArI_adj %P	ArI_unknown_adj%	ArBr_adj %P	ArBr_unknown_adj%	Score
**OB(O)c1ccc(Cl)c(F)c1**	94	0	87	6	4
**CCOc1ncccc1B(O)O**	94	0	85	3	4
**CC1(C)OB(c2cccc(O)c2F)OC1(C)C**	94	0	81	0	4
**Cc1ccc(F)cc1B(O)O**	94	0	79	0	4
**CC1(C)OB(c2cccc3c2CCO3)OC1(C)C**	94	1	78	0	4
**OB(O)c1ccc(OC(F)(F)F)cc1**	94	1	78	0	4
**COCCc1cccc(B2OC(C)(C)C(C)(C)O2)c1**	94	0	75	0	4
**Nc1cc(B(O)O)ccc1Cl**	93	0	95	0	4
**CC1(C)OB(c2ccc3ccc(=O)[nH]c3c2)OC1(C)C**	93	3	95	0	4
**CC1(C)OB(c2ccc(C3CCC(=O)N3)cc2)OC1(C)C**	93	0	93	0	4
**OB(O)c1cccc2c1OCCO2**	93	0	92	0	4
**CNC(=O)Nc1ccc(B2OC(C)(C)C(C)(C)O2)cc1**	93	0	89	0	4
**CC(C)(O)c1ccc(B2OC(C)(C)C(C)(C)O2)c(F)c1**	93	0	88	0	4
**OB(O)c1cc(F)c(F)c(F)c1**	93	0	88	2	4
**CC(C)NC(=O)c1cccc(B(O)O)c1**	93	0	83	2	4
**CC(C)OCc1cccc(B(O)O)c1**	93	1	83	1	4
**COc1ncc(B2OC(C)(C)C(C)(C)O2)cc1C**	93	0	80	0	4
**Cc1c(Cl)cccc1B(O)O**	93	0	80	0	4
**COc1ncc(Cl)cc1B(O)O**	93	0	71	0	4
**CC1(C)OB(c2cnc3ccnn3c2)OC1(C)C**	93	7	71	0	4
**COC(=O)c1cc(O)cc(B2OC(C)(C)C(C)(C)O2)c1**	92	6	94	0	4
**OB(O)c1cccc2c1OCCCO2**	92	0	87	0	4
**COc1ccc(C)cc1B(O)O**	92	0	87	4	4
**CCCNC(=O)c1cccc(B(O)O)c1**	92	0	85	0	4
**CCOc1cc(F)ccc1B(O)O**	92	0	83	0	4

Structure	ArI_adj %P	ArI_unknown_adj%	ArBr_adj %P	ArBr_unknown_adj%	Score
**CC1(C)OB(c2cccc(CN)c2)OC1(C)C**	92	1	83	1	4
**Cc1cc2cc(B3OC(C)(C)C(C)(C)O3)ccc2[nH]1**	92	3	80	0	4
**COC(=O)c1ccc(B2OC(C)(C)C(C)(C)O2)o1**	92	4	79	0	4
**CC1(C)OB(c2cccc3ccoc23)OC1(C)C**	92	6	79	9	4
**CC(C)Oc1ccc(F)cc1B(O)O**	92	0	77	0	4
**COc1ncc(B(O)O)cc1Cl**	91	0	99	0	4
**Cn1nnc2ccc(B3OC(C)(C)C(C)(C)O3)cc21**	91	5	96	1	4
**Cc1ccc(Cl)cc1B(O)O**	91	0	91	4	4
**CC1(C)OB(c2ccc3c(c2)CCCNC3=O)OC1(C)C**	91	6	91	0	4
**COC(=O)Nc1ccc(B(O)O)cc1**	91	0	89	0	4
**CCOC(=O)c1ccc(B(O)O)cc1F**	91	0	88	0	4
**CCN(CC)c1ncc(B(O)O)cn1**	91	2	87	0	4
**CCCOc1cccc(B(O)O)c1**	91	5	86	2	4
**OB(O)c1cccc(Cl)c1F**	91	0	81	0	4
**OB(O)c1cc(F)cc(C(F)(F)F)c1**	90	0	97	0	4
**COc1ccc(B(O)O)cc1C#N**	90	6	92	0	4
**OB(O)c1ccc(C(F)(F)F)c(F)c1**	90	0	91	0	4
**Cc1nc2ccc(B3OC(C)(C)C(C)(C)O3)cc2o1**	90	7	91	3	4
**OCc1ccc(B(O)O)cc1Cl**	90	8	84	0	4
**CC1(C)OB(c2ccc(C3CC(=O)C3)cc2)OC1(C)C**	90	10	79	3	4
**CS(=O)(=O)c1ccc(B(O)O)cc1**	89	0	100	0	4
**Cc1ccc(C(N)=O)cc1B1OC(C)(C)C(C)(C)O1**	89	0	100	0	4
**CC1(C)OB(c2cccc3c2CNC3=O)OC1(C)C**	89	3	100	0	4
**OB(O)c1cc(F)cc(F)c1**	89	0	98	0	4
**O=C(O)CCc1ccc(B(O)O)cc1**	89	0	91	0	4
**COC(=O)c1cc(B(O)O)ccc1Cl**	89	7	91	0	4

Structure	ArI_adj %P	ArI_unknown_adj%	ArBr_adj %P	ArBr_unknown_adj%	Score
**CC(C)(C)c1ccc(B(O)O)cc1**	89	2	86	0	4
**COc1cccc(B(O)O)c1OC**	89	1	82	0	4
**COc1cccc2cc(B3OC(C)(C)C(C)(C)O3)oc12**	89	8	71	0	4
**COc1ccc(B(O)O)cc1OC(C)C**	88	0	94	0	4
**OB(O)c1ccc(F)nc1**	88	3	94	0	4
**OB(O)c1ccc(C(F)(F)F)cc1**	88	0	86	0	4
**CCOc1ccc(B(O)O)cc1**	87	0	88	0	4
**N#Cc1cc(B(O)O)ccc1Cl**	87	5	85	0	4
**Cc1ccc(C(=O)O)cc1B1OC(C)(C)C(C)(C)O1**	87	0	83	0	4
**Cc1cc(Cl)ccc1B(O)O**	87	0	80	0	4
**OB(O)c1cccc2c1OCC2**	87	0	79	0	4
**CN(C)c1ncc(B2OC(C)(C)C(C)(C)O2)cc1Cl**	87	7	77	3	4
**COc1cc(CO)ccc1B1OC(C)(C)C(C)(C)O1**	86	0	88	0	4
**CCOc1ccccc1B(O)O**	86	0	78	0	4
**N#Cc1ccc(B(O)O)cc1F**	84	0	85	0	4
**COc1ccc(B2OC(C)(C)C(C)(C)O2)cn1**	83	0	71	0	4
**CC1Cc2cc(B(O)O)ccc2O1**	81	0	96	0	4
**OB(O)c1cncnc1**	81	4	91	0	4
**CC(C)(C)n1cc(B2OC(C)(C)C(C)(C)O2)cn1**	81	0	71	0	4
**COC(=O)c1ccc(B2OC(C)(C)C(C)(C)O2)cc1F**	80	9	84	0	4
**CCN(CC)Cc1ccc(B(O)O)cc1**	79	0	100	0	4
**OB(O)c1ccc2ccc(O)cc2c1**	78	0	91	0	4
**OB(O)c1ccc2cc[nH]c2c1**	77	0	95	3	4
**COC(=O)c1cc(B2OC(C)(C)C(C)(C)O2)ccc1O**	72	0	73	4	4
**COC(=O)c1cccc(B(O)O)c1F**	99	0	69	0	3
**OB(O)c1ccc2c(c1)OCCCO2**	99	0	58	0	3

Structure	ArI_adj %P	ArI_unknown_ adj%	ArBr_adj %P	ArBr_unknown _adj%	Sco re
**Cc1cc(B(O)O)cc(C(C)(C)C)c1**	98	0	57	0	3
**OB(O)c1ccsc1**	98	1	55	0	3
**OB(O)c1cccc(N2CCCCC2)c1**	98	0	52	0	3
**Cn1ncc2ncc(B(O)O)cc21**	97	0	51	0	3
**CC1(C)OB(c2ccc(S(C)(=O)=O)cc2)OC1(C)C**	96	0	69	0	3
**COC(=O)c1ccc(C)c(B2OC(C)(C)C(C)(C)O2)c1**	96	0	54	0	3
**CNc1cccc(B2OC(C)(C)C(C)(C)O2)c1**	96	0	53	0	3
**CN(C)Cc1ccc(F)c(B2OC(C)(C)C(C)(C)O2)c1**	96	0	52	0	3
**COc1ncc(B2OC(C)(C)C(C)(C)O2)cn1**	95	2	66	0	3
**Cc1ccc(B(O)O)cc1[N+](=O)[O-]**	95	0	62	0	3
**CC1(c2ccc(B3OC(C)(C)C(C)(C)O3)cc2)CC1**	95	2	58	1	3
**OB(O)C=CC1CCCCC1**	94	0	62	0	3
**COc1cc(B(O)O)cc(OC)c1OC**	94	0	60	0	3
**CCc1ccccc1B(O)O**	93	0	67	0	3
**Cc1ccc(OC(C)C)c(B(O)O)c1**	93	1	64	2	3
**COc1cc(F)c(F)cc1B(O)O**	93	0	61	0	3
**CCOCc1ccc(B2OC(C)(C)C(C)(C)O2)cc1F**	93	0	59	0	3
**CC(C)Oc1cc(F)ccc1B(O)O**	93	0	57	0	3
**Cc1cc(B(O)O)ccc1OC(C)C**	92	0	62	0	3
**COC(=O)c1cc(Cl)cc(B(O)O)c1**	92	0	60	0	3
**Cc1cccc(B(O)O)c1Cl**	92	0	54	0	3
**COc1c(Cl)cccc1B(O)O**	90	0	59	0	3
**COc1c(F)cc(F)cc1B(O)O**	90	0	56	0	3
**Cc1ccc(B(O)O)c(Cl)c1**	87	0	67	0	3
**CC1(C)Cc2cc(B3OC(C)(C)C(C)(C)O3)ccc2O1**	87	0	62	0	3
**CC(C)COc1ccc(B(O)O)cc1**	87	0	58	3	3
**COc1cc(C)c(B(O)O)cc1C**	85	0	53	2	3

Structure	ArI_adj %P	ArI_unknown_adj%	ArBr_adj %P	ArBr_unknown_adj%	Score
**COc1ccc(B(O)O)c(C)c1**	81	0	50	0	3
**CN(C)c1cc(B2OC(C)(C)C(C)(C)O2)ccn1**	80	0	68	0	3
**Cc1cc(B(O)O)c(C)cc1F**	80	0	68	0	3
**CC(C)c1nn(C)cc1B1OC(C)(C)C(C)(C)O1**	76	8	68	1	3
**CC1(C)OB(c2cccc(O)c2)OC1(C)C**	69	0	84	0	3
**COC(=O)Cc1ccc(B(O)O)cc1**	64	2	93	0	3
**CC1(C)OB(c2ccc3occc3c2)OC1(C)C**	50	5	73	3	3
**NC(=O)c1ccc(B(O)O)cc1Cl**	96	0	74	22	2
**COc1ccc(B(O)O)cc1C(=O)O**	89	11	88	0	2
**CONC(=O)c1ccc(B(O)O)cc1**	88	12	96	0	2
**OB(O)c1ccc2c(c1)OC(F)(F)O2**	85	13	95	0	2
**CC(O)c1ccc(B(O)O)cc1**	85	13	89	5	2
**COC(=O)c1cccc(B(O)O)c1**	83	14	76	0	2
**O=C(O)c1ccc(B(O)O)c(F)c1**	82	14	84	0	2
**CC1(C)OB(c2ccc(C3(N)CCCC3)cc2)OC1(C)C**	82	13	80	4	2
**CC(=O)Nc1ccc(B2OC(C)(C)C(C)(C)O2)cn1**	79	21	89	5	2
**N#Cc1cc(Cl)cc(B(O)O)c1**	74	24	96	0	2
**OB(O)c1ccc(N2CCCC2)nc1**	74	25	87	0	2
**CC1(C)OB(c2ccc(F)c(C#N)c2)OC1(C)C**	73	22	79	0	2
**OB(O)/C=C/c1ccc(Cl)cc1**	100	0	51	20	1
**OB(O)c1ccc2nncn2c1**	100	0	40	0	1
**CCOC(=O)c1cccc(B(O)O)c1F**	100	0	33	6	1
**Nc1ccc(B(O)O)cn1**	100	0	27	44	1
**CC1(C)OB(c2cnn(-c3ccccn3)c2)OC1(C)C**	100	0	26	0	1
**CC1(C)OB(c2ccc(N)cc2F)OC1(C)C**	100	0	17	0	1
**COC(=O)/C=C/c1cccc(B(O)O)c1**	100	0	13	0	1
**CC1(C)OB(c2cnn3cccnc23)OC1(C)C**	100	0	8	1	1

Structure	ArI_adj %P	ArI_unknown_adj%	ArBr_adj %P	ArBr_unknown_adj%	Score
**Nc1ccccc1B(O)O**	100	0	0	0	1
**Cc1cc(OC(C)C)ccc1B(O)O**	100	0	0	3	1
**COc1cccc(C#N)c1B1OC(C)(C)C(C)(C)O1**	100	0	0	6	1
**COc1ccc(/C=C/B(O)O)cc1**	100	0	0	100	1
**Cc1cc(B(O)O)cs1**	99	0	48	0	1
**CSc1ccc(B(O)O)cc1**	99	0	29	0	1
**CC1(C)OB(c2cccc3cc[nH]c23)OC1(C)C**	99	0	7	0	1
**OB(O)/C=C/c1cc(F)cc(F)c1**	98	0	41	15	1
**CC1(C)OB(c2cnc3[nH]ccc3c2)OC1(C)C**	98	0	37	0	1
**CSc1ccccc1B(O)O**	98	0	33	0	1
**Cc1cc(B(O)O)cnc1O**	98	2	9	0	1
**OB(O)c1cncc(F)c1**	98	0	0	0	1
**COC/C=C/B(O)OC(C)(C)C(C)(C)O**	98	0	0	100	1
**CC1(C)OB(c2cc[nH]c2)OC1(C)C**	97	3	41	0	1
**CC1(C)OB(c2ccc(C3CC3)c(C#N)c2)OC1(C)C**	97	0	23	2	1
**OB(O)c1cnc2ccccc2c1**	97	0	12	0	1
**CC1(C)OB(c2cccc(Cn3ccnc3)c2)OC1(C)C**	97	0	0	0	1
**Cc1n[nH]c2ccc(B3OC(C)(C)C(C)(C)O3)cc12**	97	0	0	0	1
**OB(O)c1cccc2cccnc12**	97	0	0	2	1
**Cc1ccc(/C=C/B(O)O)cc1**	97	3	0	100	1
**OB(O)/C=C/c1cccc(F)c1**	96	0	40	27	1
**Cc1ccc(Cl)c(B(O)O)c1**	96	0	20	5	1
**CCOc1ccc(B(O)O)cn1**	96	0	8	0	1
**CC1(C)OB(c2cncc3sccc23)OC1(C)C**	96	0	0	1	1
**O=[N+]([O-])c1ccc(B(O)O)cn1**	96	0	0	3	1
**O=C(O)/C=C/c1ccc(B(O)O)cc1**	96	0	0	100	1
**N#Cc1cc(B(O)O)cs1**	96	2	0	0	1
**C/C=C/B(O)O**	95	0	49	2	1

Structure	ArI_adj %P	ArI_unknown_adj%	ArBr_adj %P	ArBr_unknown_adj%	Score
**Cc1nc(N)ccc1B1OC(C)(C)C(C)(C)O1**	95	0	35	0	1
**CC(C)Oc1cncc(B2OC(C)(C)C(C)(C)O2)c1**	95	0	6	1	1
**CC1(C)OB(/C=C/c2ccccn2)OC1(C)C**	95	0	0	0	1
**Cc1cncc(B2OC(C)(C)C(C)(C)O2)c1**	95	0	0	0	1
**OB(O)c1ccc2nonc2c1**	95	0	0	1	1
**CC(=O)n1ncc2ccc(B3OC(C)(C)C(C)(C)O3)cc21**	95	0	0	3	1
**COc1ccc(C=CB2OC(C)(C)C(C)(C)O2)cc1**	95	4	0	100	1
**CC(C)Oc1ccc(Cl)cc1B(O)O**	94	0	44	2	1
**COCc1cc(B2OC(C)(C)C(C)(C)O2)cs1**	94	4	40	0	1
**CC(C)COc1cccc(B(O)O)c1F**	94	0	36	0	1
**CC(C)c1ccccc1B(O)O**	94	0	21	19	1
**CC1(C)OB(c2ccc(C(N)=O)s2)OC1(C)C**	94	1	20	0	1
**OB(O)c1ccc(Cl)cc1Cl**	94	0	14	3	1
**Cc1cccc(B2OC(C)(C)C(C)(C)O2)c1C#N**	94	0	11	0	1
**OB(O)c1cc(Cl)ccc1Cl**	94	0	5	0	1
**OB(O)c1cccc2cnccc12**	94	1	0	0	1
**Cc1nc2cc(B3OC(C)(C)C(C)(C)O3)ccc2n1C**	94	3	0	8	1
**N#Cc1cncc(B(O)O)c1**	94	6	0	1	1
**COc1c(B(O)O)ccc(F)c1F**	93	0	38	7	1
**CC1(C)OB(c2ccnc3[nH]ccc23)OC1(C)C**	93	3	27	0	1
**OB(O)c1ccc(F)cc1Cl**	93	0	12	0	1
**OB(O)c1csc(Cl)c1**	93	1	11	0	1
**Cc1cc(F)ncc1B(O)O**	93	0	0	0	1
**OB(O)c1ccc(O)nc1F**	93	0	0	0	1
**NCc1cc(F)ccc1B(O)O**	93	0	0	49	1
**COc1c(C)cccc1B(O)O**	92	0	46	2	1

Structure	ArI_adj %P	ArI_unknown_adj%	ArBr_adj %P	ArBr_unknown_adj%	Score
**OB(O)c1ccccc1CC(F)(F)F**	92	0	34	4	1
**CC(=O)Nc1ccccc1B(O)O**	92	8	26	0	1
**OB(O)c1cscc1Cl**	92	4	13	0	1
**OB(O)c1cccc(Cl)c1Cl**	92	0	0	4	1
**O=[N+]([O-])c1cc(B(O)O)ccc1Cl**	92	0	0	17	1
**O=c1[nH]cc(B(O)O)cc1F**	92	7	0	5	1
**CC(N)c1ccc(B2OC(C)(C)C(C)(C)O2)cc1F**	91	9	29	0	1
**OB(O)c1cccc(C(F)(F)F)c1F**	91	0	26	1	1
**OB(O)c1cc(Cl)ccc1F**	91	0	20	3	1
**CC1(C)OB(c2ccc3ncoc3c2)OC1(C)C**	91	6	17	0	1
**OB(O)c1ccccc1OC(F)(F)F**	91	0	15	7	1
**Cn1c(B2OC(C)(C)C(C)(C)O2)cnc1Cl**	91	0	0	0	1
**CC1(C)OB(c2cnc(C3CC3)o2)OC1(C)C**	91	0	0	4	1
**CN(C)Cc1ccc(B2OC(C)(C)C(C)(C)O2)s1**	91	7	0	0	1
**NCc1cccc(B(O)O)c1F**	90	0	41	0	1
**Cc1c(F)ccc(B(O)O)c1C**	90	0	39	0	1
**COc1ccc(B(O)O)c(OC)n1**	90	0	37	0	1
**O=[N+]([O-])c1cccc(B(O)O)c1**	90	0	25	68	1
**CN(C)C(=O)c1cncc(B2OC(C)(C)C(C)(C)O2)c1**	90	1	23	0	1
**CC(C)Oc1ccc(B(O)O)c(Cl)c1**	90	0	10	2	1
**CS(=O)(=O)c1cncc(B(O)O)c1**	90	0	0	3	1
**OB(O)c1csc2ccccc12**	90	0	0	3	1
**CN(C)c1ccc(/C=C/B2OC(C)(C)C(C)(C)O2)cc1**	90	0	0	33	1
**Cc1c(B2OC(C)(C)C(C)(C)O2)cnn1C**	90	1	0	2	1
**N#Cc1cc(F)c(B(O)O)cc1F**	90	7	0	8	1
**O=[N+]([O-])c1ccc(B(O)O)cc1**	89	0	44	0	1
**OB(O)c1ccc(C2(O)CCC2)cc1**	89	0	44	39	1
**CCOc1ccc(B(O)O)c(Cl)c1**	89	0	26	0	1

Structure	ArI_adj %P	ArI_unknown_adj%	ArBr_adj %P	ArBr_unknown_adj%	Score
**CC1(C)OB(c2cncc(OC(F)F)c2)OC1(C)C**	89	0	0	1	1
**CC1(C)OB(c2ccco2)OC1(C)C**	89	0	0	7	1
**Cc1ccncc1B1OC(C)(C)C(C)(C)O1**	89	1	0	0	1
**CC1(C)OB(c2cncc(C(F)F)c2)OC1(C)C**	88	0	0	0	1
**CC1(C)OB(c2ccc3nccnc3c2)OC1(C)C**	88	4	0	0	1
**Cn1cc(B2OC(C)(C)C(C)(C)O2)cn1**	87	2	30	0	1
**[B]c1cc(C(F)(F)F)ccc1F**	87	1	29	0	1
**CCCOc1cncc(B2OC(C)(C)C(C)(C)O2)c1**	87	0	2	0	1
**O=[N+]([O-])c1cc(B(O)O)ccc1F**	87	0	0	0	1
**CC1(C)OB(c2cncc(-n3cccn3)c2)OC1(C)C**	87	0	0	17	1
**CC(=O)c1ccc(Cl)c(B(O)O)c1**	87	1	0	9	1
**COc1cc(C#N)ccc1B(O)O**	86	5	41	0	1
**Cn1cc(B2OC(C)(C)C(C)(C)O2)c2ccccc21**	86	9	16	0	1
**OB(O)c1ccc(F)cc1O**	86	0	0	6	1
**CC1(C)OB(c2ccc(OC3CC3)cc2)OC1(C)C**	85	6	39	4	1
**CC1(C)OB(c2ccc(C(F)(F)F)nc2)OC1(C)C**	85	1	35	0	1
**Cc1ccc2cccnc2c1B1OC(C)(C)C(C)(C)O1**	85	0	0	3	1
**Cc1occc1B(O)O**	84	6	29	0	1
**OB(O)c1cccc2c1OC(F)(F)O2**	84	1	26	1	1
**Cc1cc(OC(F)F)ccc1B1OC(C)(C)C(C)(C)O1**	84	0	17	4	1
**OB(O)c1cccnc1Cl**	84	0	0	2	1
**COCCn1cc(B2OC(C)(C)C(C)(C)O2)cn1**	83	0	48	0	1

Structure	ArI_adj %P	ArI_unknown_adj%	ArBr_adj %P	ArBr_unknown_adj%	Score
**Cc1cc(B2OC(C)(C)C(C)(C)O2)cnc1Cl**	83	10	39	0	1
**Cc1c(B(O)O)ccnc1F**	83	2	26	0	1
**CN(Cc1cccc(B(O)O)c1)C1CC1**	83	17	0	100	1
**COc1cc(C)ccc1B(O)O**	82	0	34	7	1
**OCc1ccc(B(O)O)s1**	82	2	5	0	1
**COCCn1nc(C)c(B2OC(C)(C)C(C)(C)O2)c1C**	82	3	0	3	1
**OB(O)c1cccnc1F**	81	9	25	2	1
**COC(=O)Cc1ccccc1B1OC(C)(C)C(C)(C)O1**	81	6	0	4	1
**Cn1cnc2ccc(B3OC(C)(C)C(C)(C)O3)cc21**	81	15	0	0	1
**Cc1cc(B(O)O)co1**	80	5	41	0	1
**[B]c1c(OC)ccc(OC)c1OC**	80	0	0	14	1
**CCOC(=O)c1cc(B2OC(C)(C)C(C)(C)O2)[nH]n1**	80	11	0	1	1
**Cc1cnc(F)cc1B(O)O**	79	4	31	0	1
**CC1(C)OB(c2c[nH]c3ncccc23)OC1(C)C**	79	1	0	0	1
**OB(O)c1ccc(Cl)nc1**	79	16	0	48	1
**OB(O)c1cccc(F)c1C(F)(F)F**	78	2	6	0	1
**CC1(C)OB(c2cccc(F)c2C#N)OC1(C)C**	78	5	0	5	1
**CC1(C)OB(c2cccc(S(=O)(=O)F)c2)OC1(C)C**	77	23	53	47	1
**N#Cc1ccc(F)c(B(O)O)c1**	77	0	13	1	1
**CC1(C)OB(c2cccc3scnc23)OC1(C)C**	77	11	2	0	1
**OB(O)c1cc(F)nc(F)c1**	77	0	0	0	1
**CSc1ncc(B(O)O)cn1**	77	0	0	2	1
**CC(C)Oc1cccc(F)c1B(O)O**	77	0	0	3	1
**CC(C)(C)Sc1cccc(B(O)O)c1**	76	0	7	4	1
**N#Cc1cccc(B(O)O)c1Cl**	76	0	0	0	1
**Nc1ccncc1B(O)O**	76	0	0	0	1

Structure	ArI_adj %P	ArI_unknown_adj%	ArBr_adj %P	ArBr_unknown_adj%	Score
**OB(O)c1ccc(F)cc1C(F)(F)F**	76	0	0	9	1
**N#Cc1cc(B(O)O)ccc1O**	75	0	39	25	1
**COc1cccc(Cl)c1B(O)O**	75	0	19	0	1
**Cc1nn(C(C)C)cc1B1OC(C)(C)C(C)(C)O1**	75	2	9	1	1
**OB(O)c1cc(F)ccc1C(F)(F)F**	75	0	0	8	1
**OB(O)c1ccccc1OCC1CC1**	75	5	0	18	1
**CC1(C)OB(c2cc(C(F)(F)F)n[nH]2)OC1(C)C**	75	13	0	1	1
**Cc1noc(C)c1B1OC(C)(C)C(C)(C)O1**	74	2	0	10	1
**COc1ncncc1B1OC(C)(C)C(C)(C)O1**	74	7	0	11	1
**O=C(O)c1cncc(B(O)O)c1**	73	16	71	14	1
**Cc1c(B2OC(C)(C)C(C)(C)O2)ccc2c1CCO2**	72	3	16	0	1
**OB(O)c1ccoc1**	71	11	49	0	1
**CC1(C)OB(c2csc(C(F)(F)F)n2)OC1(C)C**	71	15	9	0	1
**CCCn1nc(C)c(B2OC(C)(C)C(C)(C)O2)c1C**	71	0	6	9	1
**CC1(C)OB(c2cc(Cl)ccc2N)OC1(C)C**	71	4	0	12	1
**CC1(C)OB(c2c(Cl)cccc2C#N)OC1(C)C**	69	4	0	0	1
**CC1(C)OB(C2=CC(=O)CCC2)OC1(C)C**	68	3	0	100	1
**CC1(C)OB(c2cc(F)ccc2C#N)OC1(C)C**	68	6	0	0	1
**O=[N+]([O-])c1ccccc1B(O)O**	67	0	0	0	1
**C/C=C\B(O)O**	67	6	0	9	1
**Cc1cccc(C#N)c1B1OC(C)(C)C(C)(C)O1**	65	7	0	4	1
**COc1ccc(B2OC(C)(C)C(C)(C)O2)c(C#N)c1**	65	9	0	0	1
**COc1cccc(OC)c1B(O)O**	64	0	33	0	1

Structure	ArI_adj %P	ArI_unknown_adj%	ArBr_adj %P	ArBr_unknown_adj%	Score
**CC1(C)OB(c2cccc(OC(F)F)c2)OC1(C)C**	64	0	10	3	1
**N#Cc1c(Cl)ccc(B(O)O)c1F**	63	2	0	0	1
**COc1ncc(B(O)O)c(OC)n1**	62	38	58	4	1
**CC1(C)OB(c2ccsc2C#N)OC1(C)C**	62	24	27	1	1
**CCOc1cccc(F)c1B(O)O**	62	22	9	7	1
**N#Cc1ccc(Cl)cc1B(O)O**	62	0	0	3	1
**CCc1cccc(CC)c1B(O)O**	62	0	0	6	1
**Cc1ccc2[nH]ncc2c1B(O)O**	60	0	0	0	1
**CC1(C)OB(c2ccncc2Cl)OC1(C)C**	60	0	0	2	1
**OB(O)c1ccnc(Cl)c1**	60	14	0	0	1
**CC1(C)OB(c2cc(C#N)ccc2O)OC1(C)C**	59	2	12	3	1
**Cn1nc(C(F)(F)F)cc1B1OC(C)(C)C(C)(C)O1**	54	1	0	0	1
**CN(C)c1ccccc1B(O)O**	53	0	0	1	1
**COc1cc(/C=C/B2OC(C)(C)C(C)(C)O2)ccc1O**	53	48	0	100	1
**CC1(C)OB(c2ccncc2F)OC1(C)C**	52	43	0	9	1
**CC1(C)OB(c2ccc3c(c2)CCC3=O)OC1(C)C**	49	0	87	0	1
**CC1(C)OB(c2ccc(N)c(CO)c2)OC1(C)C**	49	0	31	0	1
**CC1(C)OB(c2ccc(CN)s2)OC1(C)C**	43	28	0	0	1
**COc1ccc(F)c(F)c1B(O)O**	39	0	0	5	1
**CC#Cc1cccc(B(O)O)c1**	37	9	0	19	1
**COc1cnccc1B(O)O**	36	12	0	0	1
**COc1cccc(F)c1B(O)O**	33	14	0	12	1
**CCCOc1ccc(B(O)O)c(C)c1**	32	34	9	4	1
**Cc1cc(C)c(C)c(B(O)O)c1C**	29	0	0	1	1
**Cc1cc(C)c(B(O)O)c(C)c1**	29	6	0	5	1
**COc1cc(C)cc(C)c1B(O)O**	28	0	0	0	1
**OB(O)c1ccc(C(F)(F)F)s1**	25	11	0	3	1

Structure	ArI_adj %P	ArI_unknown_adj%	ArBr_adj %P	ArBr_unknown_adj%	Score
**CC1(C)OB(c2cnc3c(c2)CC(=O)N3)OC1(C)C**	24	63	0	0	1
**OB(O)C1CCCc2ccccc21**	21	32	6	1	1
**COc1ccc(B(O)O)c(OC)c1**	20	0	0	5	1
**CB(O)O**	19	32	0	37	1
**Cc1cocc1B1OC(C)(C)C(C)(C)O1**	18	47	0	10	1
**CCOc1ccc(/C=C/B(O)O)cc1**	16	29	0	100	1
**CS(=O)c1ccc(B(O)O)cc1**	15	0	0	0	1
**CCCOc1ccc([B-](O)(O)O)nc1**	14	7	0	14	1
**COc1ccc(B(O)O)nc1**	13	12	0	8	1
**OB(O)c1c(F)cccc1F**	12	0	0	1	1
**OB(O)c1ccccc1CN1CCCC1**	11	0	0	0	1
**CCn1nccc1B1OC(C)(C)C(C)(C)O1**	8	10	0	8	1
**Cn1ccc2cccc(B(O)O)c21**	7	4	0	0	1
**OB(O)c1ccc(-c2ccccc2)s1**	6	0	0	1	1
**CC1(C)OB(c2ccc(OC(F)F)nc2)OC1(C)C**	6	10	0	0	1
**N#Cc1ccccc1B(O)O**	4	0	0	1	1
**CC1(C)COc2ccc(B3OC(C)(C)C(C)(C)O3)cc21**	3	5	57	2	1
**CC1(C)OB(c2cncc(C(F)(F)F)c2)OC1(C)C**	3	10	0	4	1
**OB(O)c1c(F)ccc2c1OCO2**	3	11	0	0	1
**O=[N+]([O-])c1ccc(Cl)cc1B(O)O**	1	0	0	4	1
**OB(O)C1=Cc2ccccc2C1**	0	100	97	2	1
**CC(=O)c1ccc(B(O)O)cc1F**	0	0	94	0	1
**OB(O)c1ccc2occc2c1**	0	0	91	9	1
**OB(O)c1ccc(C2CCCCC2)cc1**	0	97	91	2	1
**CCCCOc1cccc(B(O)O)c1**	0	0	90	0	1
**COc1ccc(OC)c(B(O)O)c1**	0	0	88	0	1
**CCOc1ccc(F)cc1B(O)O**	0	0	87	0	1
**CN(C)c1ccc(B(O)O)cc1**	0	11	87	2	1
**OB(O)c1ccc(F)c(F)c1**	0	0	85	0	1

Structure	ArI_adj %P	ArI_unknown_adj%	ArBr_adj %P	ArBr_unknown_adj%	Score
**CC1(C)OB(c2ccc3c(c2)NC(=O)C3)OC1(C)C**	0	100	85	0	1
**CC(C)COc1cccc(B(O)O)c1**	0	100	84	10	1
**COc1ccc(B(O)O)cc1CO**	0	0	83	0	1
**COc1cc(Cl)cc(B(O)O)c1**	0	100	78	5	1
**Nc1ccc(B(O)O)cc1**	0	5	74	12	1
**Cc1cccc(B(O)O)c1O**	0	40	72	0	1
**COC(=O)c1ccc(F)c(B(O)O)c1**	0	0	68	0	1
**COc1nscc1B1OC(C)(C)C(C)(C)O1**	0	0	68	1	1
**OB(O)c1cccc(F)c1O**	0	0	61	0	1
**Cc1cc(C(F)(F)F)ccc1B(O)O**	0	100	58	0	1
**OB(O)c1ccc(Cc2ccccc2)cc1**	0	100	55	9	1
**CC(=O)c1ccc(B(O)O)c(F)c1**	0	0	48	0	1
**OB(O)c1ccc(O)c(Cl)c1**	0	0	46	0	1
**Cc1cc(B2OC(C)(C)C(C)(C)O2)ccn1**	0	2	45	5	1
**OB(O)c1ccccc1-c1ccccc1**	0	0	35	0	1
**Cc1ccc(B(O)O)c2ccccc12**	0	100	35	0	1
**O=C(O)c1ccc(B(O)O)s1**	0	100	29	0	1
**CC(C)B(O)O**	0	0	21	6	1
**Cc1cccc2cc(B(O)O)cnc12**	0	2	18	0	1
**COC(=O)c1ccc(Cl)c(B(O)O)c1**	0	0	15	4	1
**COc1ccc(B2OC(C)(C)C(C)(C)O2)c(CN)c1**	0	0	14	0	1
**CC(=O)c1ccccc1B(O)O**	0	0	7	0	1
**COc1ccc(C)c(F)c1B(O)O**	0	68	5	4	1
**C=C(C)B1OC(C)(C)C(C)(C)O1**	0	0	0	0	1
**CC1(C)OB(C#CC2CC2)OC1(C)C**	0	0	0	0	1
**CC1(C)OB(c2ccc(-c3cn[nH]c3)cc2)OC1(C)C**	0	0	0	0	1
**CC1(C)OB(c2cccc(-c3ncc[nH]3)c2)OC1(C)C**	0	0	0	0	1
**COC(=O)c1ccc(F)cc1B(O)O**	0	0	0	0	1
**COC(=O)c1ccccc1B(O)O**	0	0	0	0	1
**COc1cc(B(O)O)sn1**	0	0	0	0	1

Structure	ArI_adj %P	ArI_unknown_adj%	ArBr_adj %P	ArBr_unknown_adj%	Score
**Cc1ccc(B(O)O)cc1N**	0	0	0	0	1
**Cc1ccc(CNCC2CCCO2)s1**	0	0	0	0	1
**O=C(O)c1ccc(F)cc1B(O)O**	0	0	0	0	1
**OB(O)c1c(F)cc(F)cc1F**	0	0	0	0	1
**OB(O)c1cc2ccccc2o1**	0	0	0	0	1
**OB(O)c1ccc2[nH]cnc2c1**	0	0	0	0	1
**O=C(O)c1ccccc1B(O)O**	0	0	0	1	1
**O=[N+]([O-])c1ccc(Cl)c(B(O)O)c1**	0	0	0	1	1
**OB(O)c1ccccc1C(F)(F)F**	0	0	0	1	1
**OB(O)c1c(F)cc(O)cc1F**	0	0	0	2	1
**OB(O)/C=C/c1ccccc1**	0	0	0	4	1
**OB(O)c1c(F)ccc(O)c1F**	0	0	0	4	1
**CC(C)=CB(O)O**	0	0	0	5	1
**CCn1nc(C)cc1B1OC(C)(C)C(C)(C)O1**	0	0	0	5	1
**Cc1ccc(B2OC(C)(C)C(C)(C)O2)o1**	0	0	0	6	1
**N#CC=Cc1ccc(B(O)O)cc1**	0	0	0	6	1
**OB(O)c1cnn(Cc2ccccc2)c1**	0	0	0	6	1
**OB(O)c1c(F)cccc1Cl**	0	0	0	7	1
**CC(C)CB(O)O**	0	0	0	8	1
**CC(C)(O)C(C)(C)OB(O)C1=CCCCO1**	0	0	0	11	1
**OB(O)c1cccc(C(F)(F)F)c1O**	0	0	0	15	1
**CC(C)(O)C(C)(C)OB(O)/C=C/C1CC1**	0	0	0	60	1
**COc1cccc(/C=C/B(O)O)c1**	0	0	0	100	1
**CC1(C)OB(c2[nH]ncc2C(F)(F)F)OC1(C)C**	0	2	0	0	1
**COC(=O)c1ccc(Cl)cc1B(O)O**	0	2	0	0	1
**OB(O)c1ccc(O)cc1F**	0	3	0	1	1
**Cc1nn(C)cc1B1OC(C)(C)C(C)(C)O1**	0	4	0	0	1
**CCCB(O)O**	0	4	0	2	1
**CC(C)=C(C)B(O)O**	0	4	0	100	1
**[B]c1c(F)cccc1C(F)(F)F**	0	5	0	3	1
**Cn1nncc1B1OC(C)(C)C(C)(C)O1**	0	5	0	9	1
**OB(O)c1c(F)ccc(F)c1F**	0	6	0	0	1

Structure	ArI_adj %P	ArI_unknown_ adj%	ArBr_adj %P	ArBr_unknown _adj%	Score
**CC(C)(C)C=CB(O)O**	0	7	0	0	1
**CC1(C)OB(c2cnsc2)OC1(C)C**	0	7	0	3	1
**Cc1ccc(B2OC(C)(C)C(C)(C)O2)cc1Cl**	0	8	0	3	1
**CCB(O)O**	0	9	0	3	1
**NC(=O)c1cccc(B(O)O)c1F**	0	9	0	5	1
**CC1(C)OB(C2(C#C[Si](C)(C)C)CC2)OC1(C)C**	0	9	0	8	1
**Cn1ncc(Cl)c1B1OC(C)(C)C(C)(C)O1**	0	10	0	0	1
**Cc1ccc(Cl)c(B(O)O)c1F**	0	10	0	2	1
**CC1(C)OB(c2ccsc2C(F)(F)F)OC1(C)C**	0	10	0	3	1
**Cc1ccc(B2OC(C)(C)C(C)(C)O2)cn1**	0	10	0	6	1
**CC1(C)OB(c2ccc(F)s2)OC1(C)C**	0	11	0	0	1
**Cc1nc(C)c(B2OC(C)(C)C(C)(C)O2)s1**	0	11	0	6	1
**Cn1nccc1B1OC(C)(C)C(C)(C)O1**	0	11	0	8	1
**O[B-](O)(O)c1ccccn1**	0	12	0	0	1
**O[B-](O)(O)c1cnccn1**	0	12	0	0	1
**CC1(C)OB(c2ccc(Cl)s2)OC1(C)C**	0	13	0	0	1
**CCOC(=O)c1ccccc1B(O)O**	0	13	0	0	1
**CC1(C)OB(c2cncs2)OC1(C)C**	0	13	0	2	1
**C=C(B1OC(C)(C)C(C)(C)O1)C(F)(F)F**	0	13	0	3	1
**Cn1ncc(C(F)(F)F)c1B1OC(C)(C)C(C)(C)O1**	0	13	0	3	1
**Cc1cc(B2OC(C)(C)C(C)(C)O2)on1**	0	13	0	4	1
**CC1(C)OB(c2cc(Cl)cs2)OC1(C)C**	0	14	0	0	1
**CC1(C)OB(C2=CCOC2)OC1(C)C**	0	14	0	50	1
**Cc1cc(B2OC(C)(C)C(C)(C)O2)sn1**	0	15	0	13	1
**CC1(C)OB(c2ccc(F)cc2C2CC2)OC1(C)C**	0	16	0	0	1
**N#Cc1ccc(O)c(B(O)O)c1**	0	16	0	3	1
**CC1(C)OB(c2cnc(C(F)(F)F)s2)OC1(C)C**	0	16	0	6	1

Structure	ArI_adj %P	ArI_unknown_adj%	ArBr_adj %P	ArBr_unknown_adj%	Score
**OB(O)/C=C/c1ccc(F)cc1**	0	16	0	30	1
**Cc1ccc(B2OC(C)(C)C(C)(C)O2)s1**	0	17	0	9	1
**COCc1noc(C)c1B(O)O**	0	18	0	0	1
**C#CB1OC(C)(C)C(C)(C)O1**	0	18	0	4	1
**OB(O)C=Cc1ccccc1**	0	18	0	7	1
**COc1ccc([B-](O)(O)O)nc1**	0	19	0	0	1
**CC1(C)OB(c2cnn(C(F)(F)F)c2)OC1(C)C**	0	20	0	0	1
**Cc1ccsc1B1OC(C)(C)C(C)(C)O1**	0	20	0	0	1
**CC1(C)OB(c2cccs2)OC1(C)C**	0	20	0	4	1
**Cc1ncc(B2OC(C)(C)C(C)(C)O2)s1**	0	20	0	5	1
**CC1(C)OB(c2cnc(Cl)s2)OC1(C)C**	0	20	0	7	1
**OB(O)c1ccc(O)c(F)c1**	0	21	0	1	1
**CN1CC=C(B2OC(C)(C)C(C)(C)O2)CC1**	0	22	0	0	1
**Cc1coc(B2OC(C)(C)C(C)(C)O2)c1**	0	22	0	4	1
**OB(O)c1cccs1**	0	24	0	1	1
**CC1(C)OB(C=C2CCCCC2)OC1(C)C**	0	25	0	0	1
**Cc1ccc([N+](=O)[O-])cc1B(O)O**	0	27	0	2	1
**C=C(CO)B1OC(C)(C)C(C)(C)O1**	0	32	0	100	1
**OB(O)c1cscc1C(F)F**	0	33	0	0	1
**OB(O)c1cnc(Cl)nc1**	0	37	0	0	1
**Cn1c(B(O)O)cc2ccc(Cl)cc21**	0	37	0	14	1
**CC1(C)OB(c2ccc(C#N)s2)OC1(C)C**	0	42	0	13	1
**CC1(C)OB(C2=CCCCC2)OC1(C)C**	0	42	0	100	1
**CC1(C)OB(C2=CCOCC2)OC1(C)C**	0	46	0	2	1
**O=Cc1ccsc1B(O)O**	0	79	0	0	1
**CC(C)=CB1OC(C)(C)C(C)(C)O1**	0	100	0	11	1
**CC1(C)OB(c2ccoc2)OC1(C)C**	0	100	0	17	1
**CC1(C)OB(C2=CCCOC2)OC1(C)C**	0	100	0	100	1
**OB(O)c1ccc(B(O)O)cc1**	0	100	0	100	1
**OB(O)c1cccc(B(O)O)c1**	0	100	0	100	1

9. Building block to sequence association information

Supplementary Table 14 | Building block to sequence association information

Cycle	Tag (5'-3')	Smiles
1	GCACCATCT	O=C(O)CCCCCNC(=O)[C@@H]1NC[C@@H]1c1ccc(I)cc1
1	GCACTCTCT	O=C(O)CCCCCNC(=O)[C@H]1NC[C@H]1c1ccc(I)cc1
1	TCTCGCTCT	O=C(O)CCCCCNC(=O)[C@H]1NC[C@@H]1c1ccc(I)cc1
1	ATCTCGGCT	O=C(O)CCCCCNC(=O)[C@@H]1NC[C@H]1c1ccc(I)cc1
1	ATGGAGGCT	O=C(O)CCCCCNC(=O)[C@@H]1NCC[C@@H]1c1ccc(I)cc1
1	CGCACAACT	O=C(O)CCCCCNC(=O)[C@H]1NCC[C@H]1c1ccc(I)cc1
1	CGCACTTCT	O=C(O)CCCCCNC(=O)[C@H]1NCC[C@@H]1c1ccc(I)cc1
1	TGGAAGCCT	O=C(O)CCCCCNC(=O)[C@@H]1NCC[C@H]1c1ccc(I)cc1
1	TGGAGACCT	O=C(O)[C@@H]1NC[C@@H]1c1cccc(I)c1
1	AGATGGCCT	O=C(O)[C@H]1NC[C@H]1c1cccc(I)c1
1	AGGACTGCT	O=C(O)[C@H]1NC[C@@H]1c1cccc(I)c1
1	AGTACCGCT	O=C(O)[C@@H]1NC[C@H]1c1cccc(I)c1
1	CTCTACGCT	O=C(O)[C@@H]1NCC[C@@H]1c1cccc(I)c1
1	GACAGACCT	O=C(O)[C@H]1NCC[C@H]1c1cccc(I)c1
1	GGCAACACT	O=C(O)[C@H]1NCC[C@@H]1c1cccc(I)c1
1	GACCGAACT	O=C(O)[C@@H]1NCC[C@H]1c1cccc(I)c1
1	GAGCTGACT	O=C(O)[C@@H]1NCCC[C@@H]1c1cccc(I)c1
1	GCGAAGACT	O=C(O)[C@H]1NCCC[C@H]1c1cccc(I)c1
1	GGCGATTCT	O=C(O)[C@H]1NCCC[C@@H]1c1cccc(I)c1
1	TAGACCGCT	O=C(O)[C@@H]1NCCC[C@H]1c1cccc(I)c1
1	CGACCAACT	O=C(O)CCCC(=O)N1C[C@H](c2ccc(I)cc2)C2(CNC2)C1
1	GCTTCCACT	O=C(O)CCCC(=O)N1C[C@@H](c2ccc(I)cc2)C2(CNC2)C1
1	CTGAGGACT	O=C(O)CCCC(=O)N1CC2(CNC[C@@H]2c2ccc(I)cc2)C1
1	GACCGTTCT	O=C(O)CCCC(=O)N1CC2(CNC[C@H]2c2ccc(I)cc2)C1
1	GAGTAGCCT	O=C(O)[C@@H]1C[C@H](Oc2ccc(I)cc2)CN1
1	GCTCGAACT	O=C(O)[C@@H]1C[C@@H](Oc2ccc(I)cc2)CN1
1	GCTGCTTCT	O=C(O)[C@H]1C[C@@H](Oc2ccc(I)cc2)CN1
1	GCTGTCTCT	O=C(O)[C@H]1C[C@H](Oc2ccc(I)cc2)CN1
1	TACGAGCCT	O=C(O)[C@@H]1C[C@H](Oc2cccc(I)c2)CN1
1	TACGCGACT	O=C(O)[C@@H]1C[C@@H](Oc2cccc(I)c2)CN1
1	TCCTCTGCT	O=C(O)[C@H]1C[C@@H](Oc2cccc(I)c2)CN1

Cycle	Tag (5'-3')	Smiles
1	TCGGCATCT	O=C(O)[C@H]1C[C@H](Oc2cccc(I)c2)CN1
1	TCGGTCACT	O=C(O)[C@@H]1C[C@H](OCc2ccc(I)cc2)CN1
1	TCTGCCTCT	O=C(O)[C@@H]1C[C@@H](OCc2ccc(I)cc2)CN1
1	TTGGCGTCT	O=C(O)[C@H]1C[C@@H](OCc2ccc(I)cc2)CN1
1	TTGGTGCCT	O=C(O)[C@H]1C[C@H](OCc2ccc(I)cc2)CN1
1	AAGGCCACT	O=C(O)[C@@H]1C[C@H](OCc2cccc(I)c2)CN1
1	ACACCGACT	O=C(O)[C@@H]1C[C@@H](OCc2cccc(I)c2)CN1
1	AGAACGGCT	O=C(O)[C@H]1C[C@@H](OCc2cccc(I)c2)CN1
1	AGAGTGCCT	O=C(O)[C@H]1C[C@H](OCc2cccc(I)c2)CN1
1	TCACGACCT	OC[C@@H]1[C@@H](c2ccc(Br)cc2)[C@@H]2CNCCCCN12
1	TCCACCACT	O=C1CNC[C@H]2[C@H](c3ccc(Br)cc3)[C@@H](CO)N12
1	TCGCTGTCT	OC[C@H]1[C@@H](c2ccc(Br)cc2)[C@H]2CNCCCCN21
1	TCTTGGCCT	O=C1CNC[C@H]2[C@@H](c3ccc(Br)cc3)[C@@H](CO)N12
1	TGACGGACT	O=C1CNC[C@@H]2[C@H](c3ccc(Br)cc3)[C@@H](CO)N12
1	GATGCTCCT	O=C1CNC[C@H]2[C@@H](c3ccc(Br)cc3)[C@H](CO)N12
1	GAGGTTCCT	O=C1CNC[C@@H]2[C@@H](c3ccc(Br)cc3)[C@H](CO)N12
1	TACGGTGCT	OC[C@@H]1[C@H](c2ccc(Br)cc2)[C@H]2CNCCCCN12
1	GCTGCAACT	OC[C@@H]1[C@H](c2ccc(Br)cc2)[C@@H]2CNCCCCN12
1	GCTTAGGCT	OC[C@@H]1[C@@H](c2ccc(Br)cc2)[C@H]2CNCCCCN12
1	GGTCTGACT	CNC[C@H]1Oc2cc(Br)ccc2S(=O)(=O)N([C@@H](C)CO)C[C@H]1C
1	ACTTCGCCT	CNC[C@H]1Oc2cc(Br)ccc2S(=O)(=O)N([C@H](C)CO)C[C@H]1C
1	AGACGCACT	CNC[C@@H]1Oc2cc(Br)ccc2S(=O)(=O)N([C@@H](C)CO)C[C@@H]1C
1	AGCGAGACT	CNC[C@@H]1Oc2cc(Br)ccc2S(=O)(=O)N([C@H](C)CO)C[C@@H]1C
1	AGTCGTGCT	CNC[C@@H]1Oc2cc(Br)ccc2S(=O)(=O)N([C@H](C)CO)C[C@H]1C
1	ATCCAGCCT	CNC[C@H]1Oc2cc(Br)ccc2S(=O)(=O)N([C@H](C)CO)C[C@@H]1C
1	CGAGTCTCT	CNC[C@@H]1Oc2cc(Br)ccc2S(=O)(=O)N([C@@H](C)CO)C[C@H]1C
1	CAGCGATCT	CCOC(=O)c1cc2c(c(-c3ccc(Br)cc3)n1)[C@@H](CCO)NC2
1	CAGCTACCT	CCOC(=O)c1cc2c(c(-c3cccc(Br)c3)n1)[C@H](CCO)NC2
1	CATACCGCT	O=C(O)c1cc2c(c(-c3ccc(Br)cc3)n1)[C@@H](CCO)NC2
1	CGACAGTCT	O=C(O)c1cc2c(c(-c3cccc(Br)c3)n1)[C@H](CCO)NC2
2	ACAGCAGAC	Cc1nc2cccnn2c1C(=O)O
2	CGCCTATAC	Cn1ncc(CCC(=O)O)n1
2	GCCAATGAC	N#Cc1cccc([C@H]2C[C@H]2C(=O)O)c1
2	GTTCGTCAC	Cc1nc(CC(=O)O)c(C)s1
2	ACTTGCCAC	O=C(O)[C@@H]1CCc2nnnn2CC1

Cycle	Tag (5'-3')	Smiles
2	CGTCATCAC	O=C(O)c1coc(-c2ccccn2)n1
2	GGATTGCAC	O=C1Cc2c(C(=O)O)ccnc2N1
2	TGATGCGAC	Cc1nc2ccc(C(=O)O)cc2n1C
2	ACGCTTGAC	Cc1ccsc1[C@@H]1C[C@@H]1C(=O)O
2	CGCTATGAC	COc1ccc2cc(C(=O)O)[nH]c2c1
2	GCTGCATAC	CCn1nc(C)c(C(=O)O)n1
2	TCCAGAGAC	O=C(O)CC[C@@H]1CCc2ccccc21
2	AGAGACGAC	CCn1nc(C2CC2)cc1C(=O)O
2	CGTGGTTAC	C[C@@H]1CCc2nc(C(=O)O)cn2C1
2	GGCTATCAC	O=C(O)c1cc([N+](=O)[O-])cnc1O
2	TGCCACTAC	O=C(O)c1ccc(-n2ccnc2)nn1
2	AGTACGGAC	C/C(=C\c1ccccn1)C(=O)O
2	CGCTTAGAC	CCNc1cccnc1C(=O)O
2	GGACCATAC	O=C(O)C[C@@H]1CCC2(CCC2)CO1
2	TCGTCACAC	CCOc1oc(C)nc1C(=O)O
2	AGCAAGGAC	O=C(O)c1ncoc1-c1ccon1
2	CTACCAGAC	O=C(O)c1ccc2oc(=O)[nH]c2n1
2	GGTGGATAC	O=C(O)C1=NN(CCO)C(=O)CC1
2	TGCTCAGAC	Cc1cn2c(n1)C[C@H](C(=O)O)CC2
2	ATCTGCCAC	O=C(O)C1=C(c2ccccc2)CCC1
2	CGTCAACAC	Cc1cc(C(=O)O)c(C)n1C1CC1
2	GGCAGTAAC	C=CCNc1ncc(C(=O)O)s1
2	TGCCTCTAC	Cn1nc(CC(=O)O)c2ccccc21
2	AGCGTAGAC	Cc1cnc2nc(C(=O)O)nn2c1
2	CTACGCTAC	O=C(O)c1ccnc(-c2ncc[nH]2)c1
2	GTGTACGAC	O=C(O)C[C@@H]1COc2ccccc21
2	TGGAGTGAC	O=C(O)COCc1cc[nH]c(=O)c1
2	CAACGTGAC	O=C(O)c1nsc2ccccc12
2	CTATGGCAC	Cc1nc(CC2CC2)oc1C(=O)O
2	GGCATACAC	O=C(O)CN1Cc2ccccc2C1=O
2	TGGAACCAC	O=C(O)Cn1cc(C2CCC2)nn1
2	ATCGACGAC	Cn1ccc(-c2cc(C(=O)O)[nH]n2)c1
2	CTGGTAGAC	CS(=O)(=O)c1cc(C(=O)O)co1
2	GTGTGAGAC	Cc1ccn([C@H](C)CC(=O)O)n1
2	TGTGAGCAC	C#CCOc1ccc(C(=O)O)cc1

Cycle	Tag (5'-3')	Smiles
2	CACACAGAC	O=C(O)c1coc2cccc(O)c12
2	CTCACCAAC	O=C(O)c1ccc(=O)n(CCF)n1
2	GGTAACGAC	Cn1cc(C(=O)O)c(=O)n(C)c1=O
2	TTCGGCAAC	CCC(=O)N[C@@H]1CC[C@@H](C(=O)O)C1
2	ATGTCGCAC	O=C(O)c1cccc(-n2ccnn2)c1
2	CTGTAGCAC	Cc1nc(C)n(CCCC(=O)O)n1
2	GTTCACCAC	O=C(O)c1cn([C@H]2CC[C@H]2O)nn1
2	TTAGGCGAC	CCc1cc(C(=O)O)cc(Cl)n1
2	CACAGAGAC	CO[C@H]1C[C@H](C(=O)O)N(C(C)=O)C1
2	CTCGTGAAC	CS(=O)(=O)N1CC(C(=O)O)C1
2	GGTATGGAC	CC1(C)C[C@]2(CCO1)C[C@@H]2C(=O)O
2	AACCGACAC	Cc1cccc2nc(C(=O)O)cn12
2	ATTCGGCAC	Cc1oc(C2CC2)nc1C(=O)O
2	CTTCGGAAC	Cc1cc(C)cc(CC(=O)O)c1
2	TACTCGCAC	CC[C@H](O)Cn1cc(C(=O)O)nn1
2	TTGGAGCAC	Cc1cc(C2(C(=O)O)CC2)on1
2	CACGCATAC	N#Cc1nc(C(=O)O)ccc1Cl
2	CTGAACCAC	Cc1ncsc1COCC(=O)O
2	GGTGGTAAC	O=C(O)c1n[nH]c2c1COCC2
2	AACCTCCAC	COCCN1C[C@@H](C(=O)O)CC1=O
2	CACCTCAAC	O=C(O)CCNC(=O)[C@@H]1CCCO1
2	GCACCTAAC	O=C(O)CC1=CCOCC1
2	TAGCCTGAC	O=C(O)/C=C/c1ccc2c(c1)CCO2
2	AACAGCGAC	O=C(O)c1c[nH]nc1[C@@H]1CCOC1
2	CACGCTAAC	O=C(O)c1conc1C1CCCC1
2	CTTCGCTAC	COCCn1cc(C(=O)O)c(C)n1
2	GTCACTCAC	CN(C)c1cc(C#N)cc(C(=O)O)c1
2	AACGGACAC	O=C1N=c2cc(C(=O)O)cnc2=N1
2	CAGTCCTAC	O=C1NC(=O)C2(CC(C(=O)O)C2)N1
2	GCCACATAC	CC1=C(C(=O)O)C(=O)OC1(C)C
2	TAGTGCGAC	O=C(O)c1cnoc1-c1ccco1
2	AACGCAGAC	Cc1ncc(C(=O)O)c(C(C)C)n1
2	CAGAACGAC	Cc1cc(C(=O)O)c2n[nH]cc2c1
2	CTTGCACAC	O=C(O)Cc1n[nH]c2c1CCCC2
2	GTCGCTAAC	O=C(O)CCc1cc2ccccc2o1

Cycle	Tag (5'-3')	Smiles
2	ACATCGCAC	O=C(O)C#Cc1ccc2c(c1)OCO2
2	CGAACACAC	CC(C)Cc1nc(C(=O)O)c[nH]1
2	GCGTGATAC	Cc1nn(C)c(C)c1OCC(=O)O
2	TCCTCACAC	O=C(O)[C@@H]1CN2CCC[C@@H]2CO1
2	AAGTCGCAC	Cc1cc(C(=O)O)c2c(C)n[nH]c2n1
2	CGAAGACAC	O=C(O)c1cc2occc2s1
2	GCAGACTAC	Cn1nnc2c1NCC[C@@H]2C(=O)O
2	GTCTGCTAC	O=C(O)c1cc(C2CC2)[nH]c(=O)c1
2	ACCTCTGAC	C[C@H](Oc1cccc(C#N)c1)C(=O)O
2	CGTAGTGAC	Cc1nn(CC(=O)O)c(=O)o1
2	GCTACGAAC	C#CC1(O)CC(C(=O)O)C1
2	TCGTCCTAC	CS(=O)c1ccccc1C(=O)O
2	ACACACCAC	O=C(O)CCn1ccc2ccccc21
2	CGCCATAAC	C[C@@H]1Cc2cccc(C(=O)O)c2O1
2	GCATGTCAC	O=C(O)c1n[nH]c([N+](=O)[O-])c1Cl
2	GTGTCCTAC	O=C(O)c1coc2c1C(=O)CCC2
2	ACGACCAAC	CC1(C)[C@H](C#N)[C@@]1(C#N)C(=O)O
2	CGTATCGAC	C#CCNc1ncc(C(=O)O)c(C)n1
2	GCTCATCAC	O=C(O)c1nc2n(n1)CCCN2
2	TCTCGTGAC	COCc1ccc(C(=O)O)cc1F
2	ACTCCGAAC	O=C(O)c1cc2n(n1)CCC[C@H]2O
2	AGACTCGAC	CC(=O)Nc1cccc(C(=O)O)n1
2	CACCTCTAC	O=C(O)c1ncoc1[C@@H]1CCCO1
2	GCAGGTTAC	CC(C)(C(=O)O)n1cc(Cl)cn1
2	GTTCACGAC	O=C(O)c1cccc(-c2nnco2)c1
2	ACAGCGAAC	CCN1C(=O)CC[C@H]1CC(=O)O
2	ATGGTGGAC	O=C(O)c1ccc2scnc2c1
2	GGAACCAAC	Cc1sc(Cl)c(C(=O)O)c1C
2	TCGCAAGAC	O=C(O)C1(n2cccn2)CC1
2	AGAGAGGAC	O=C(O)Cn1ncc2cccnc21
2	CATCCAGAC	COC1(C(=O)O)CS(=O)(=O)C1
2	GCCTGTAAC	CC(C)c1ccc(C(=O)O)c(=O)[nH]1
2	TAAGCCGAC	O=C(O)CNC(=O)Cn1cncn1
2	ACAGGACAC	O=C(O)c1ncc2c(F)cccn12
2	CAAGGACAC	Cc1n[nH]c(C)c1[C@H](C)C(=O)O

Cycle	Tag (5'-3')	Smiles
2	GGACGTTAC	Cc1nc2c(C(=O)O)cccc2o1
2	TCTACCGAC	Cc1nc(C(=O)O)c2n1CCCC2
2	AGAGGACAC	CC1=C(C(=O)O)Cc2ccccc2O1
2	CGAACCTAC	O=C(O)c1cnc(CC2CC2)s1
2	GCGTAACAC	O=C(O)c1cc(-n2cccn2)ccn1
2	TATGCGGAC	O=C(O)Cc1csc(C2CC2)n1
2	ACATGCCAC	Cn1cnn(CCC(=O)O)c1=O
2	CACTGACAC	Cc1nnc(CCCC(=O)O)n1C
2	GGCAATCAC	O=C(O)c1cc2sccc2[nH]1
2	TGCGTGAAC	Cc1nc2c([nH]1)CC[C@H](C(=O)O)C2
2	AGAGTGGAC	C/C(=C(/F)C(=O)O)C1CC1
2	CGTATCCAC	O=C(O)C[C@@H]1C(=O)Nc2ccccc21
2	GCGTATCAC	Cc1cc2ccc(C(=O)O)cn2c1
2	TCACCTCAC	COCc1nc(C(=O)O)co1
2	ACCGACAAC	O=C(O)/C=C1\CN2CCC1CC2
2	CAGCCTAAC	CC(=O)[C@@H]1C[C@H](CC(=O)O)C1(C)C
2	GGTAAGCAC	O=C(O)C1=NO[C@@H](c2ccccc2)C1
2	TGCTCACAC	Cc1onc(CO)c1C(=O)O
2	AGGACAGAC	O=C(O)c1ccc2c(c1)B(O)OC2
2	CTACGAGAC	O=C(O)C1=C[C@@H](O)[C@@H](O)[C@H](O)C1
2	GCTTGAGAC	O=C(O)[C@@H]1CCc2nnc(O)n2C1
2	TCACGCTAC	CO[C@H](C)c1nc(C(=O)O)cs1
2	ACTGCGAAC	CCOc1cccc(CC(=O)O)n1
2	CTCGCTAAC	O=C(O)c1nc2c(s1)CCC2
2	GGTACACAC	O=C(O)c1csnc1Cl
2	TGGAGAGAC	O=C(O)c1cc2c(s1)CCOC2
2	AGTCCTGAC	C=C(C)C[C@@H](C)C(=O)O
2	CTCCTACAC	Cn1nnc(C(=O)O)c1C(F)F
2	GGAGATGAC	O=C1CC[C@@H](C(=O)O)n2cccc21
2	TCGCGTTAC	Cc1c(C(=O)O)nn2c1OCCC2
2	AGACCACAC	Cn1nnc2c(C(=O)O)ccnc21
2	CTGACTGAC	NS(=O)(=O)c1ccc(C(=O)O)cc1
2	GGTATGCAC	CC(C)(C)OC(=O)N1CC(C=O)C1
2	TGTCAGCAC	COc1cncc(CCC=O)c1
2	AGTGCTCAC	O=Cc1ccc2nccnc2c1

Cycle	Tag (5'-3')	Smiles
2	CTCGTCTAC	Cc1ccc(CCC=O)cn1
2	GGTAGTGAC	O=Cc1cc(-n2ccnc2)cs1
2	TGAAGGCAC	O=Cc1cc(CO)ccc1O
2	AGCGAAGAC	CS(=O)c1ccc(C=O)cc1
2	CTGTTCCAC	O=Cc1cc(C(F)(F)F)[nH]n1
2	GGTCTCAAC	Cc1nn(-c2ccncc2)cc1C=O
2	TGTGGTCAC	Cn1cnc(C=O)c1
2	AGTGGTCAC	Cc1n[nH]c(Cl)c1C=O
2	CTCTTGGAC	Cn1nccc1-c1ccccc1C=O
2	GGTCACTAC	O=Cc1occc1C(=O)O
2	TGACGAGAC	O=Cc1n[nH]cc1-c1ccccc1
2	AGGAGAGAC	O=Cc1ccc(C(=O)NC2CC2)cc1
2	CTTGGTGAC	O=Cc1cnc[nH]1
2	GGTGGTTAC	N#Cc1ccc(CCC=O)cc1
2	TTCCAGGAC	O=Cc1cccc(Cc2cnc[nH]2)c1
2	ATGGACGAC	CS(=O)(=O)c1cccc(C=O)c1
2	CTGCATCAC	NS(=O)(=O)c1ccc(C=O)o1
2	GGTGCTAAC	O=Cc1cnc(-c2ccccc2)[nH]1
2	TGCTGAGAC	Cn1nc(-c2ccccc2)cc1C=O
2	AGGATGCAC	Cc1ncc(C=O)n1-c1ccccc1
2	GCATCCAAC	O=Cc1cnn(Cc2ccccc2)c1
2	GTGGAAGAC	Cc1c(C(F)(F)F)n[nH]c1C=O
2	TTCTCGGAC	COC(=O)c1[nH]cc(C=O)c1C
2	CAACCTGAC	O=Cc1cn[nH]c1
2	CTGCTTCAC	O=Cc1ccc2cn[nH]c2c1
2	GTAACGCAC	O=Cc1ccnn1-c1ccccn1
2	TGGCACAAC	N#Cc1csc(C=O)c1
2	ATAGGCGAC	O=Cc1ccnc(-c2ccccc2)n1
2	GCATTGGAC	O=Cc1cc(C2CC2)[nH]n1
2	GTGTCTCAC	COc1cccc2cc(C=O)oc12
2	TTGAGCGAC	N#CCOc1ccccc1C=O
2	CAAGGTGAC	O=Cc1cccc(-c2ccccn2)c1
2	CTGGTTGAC	O=Cc1ccc(-n2ccnc2)cc1
2	GTCTAGGAC	NS(=O)(=O)c1cc(C=O)co1
2	TGGCTGAAC	O=Cc1cc(O)c2ccsc2c1

Cycle	Tag (5'-3')	Smiles
2	ATCCTCGAC	Cn1cc(-c2cccc(C=O)c2)cn1
2	GCCATTGAC	O=Cc1ccc(O)c(F)c1
2	TAAGGCGAC	Cc1cc(C=O)cnc1-n1cccn1
2	TTGGCACAC	Cn1cc(C=O)c(-c2cccnc2)n1
2	CACAGACAC	Cc1ccccc1-n1cc(C=O)cn1
2	GCACACAAC	Cc1nc2ccccc2nc1C=O
2	GTGACCTAC	COC(=O)c1cc(C=O)cn1C
2	AACCGTCAC	O=Cc1ccn(-c2cccnc2)c1
2	ATGCGAGAC	CC(C)(C#N)c1ccc(C=O)cc1
2	GCCTCATAC	O=Cc1cn[nH]c1-c1ccccc1
2	TAGCCTCAC	Cc1ccc2cc(C=O)ccc2n1
2	AAGGCTGAC	COc1nc2ccccc2cc1C=O
2	ACACCAGAC	O=Cc1nc(-c2ccccc2)c[nH]1
2	CGCTACAAC	CCOC(=O)c1cc(C=O)[nH]n1
2	GGACTTGAC	O=Cc1cn(-c2ccccc2)cn1
2	TCTGAGGAC	O=Cc1nccn1-c1ccccc1
2	AGACCTGAC	O=Cc1ccccc1-n1cccn1
2	ATGCACGAC	Cn1cc(-n2ccc(C=O)c2)cn1
2	CTTGCAGAC	Cc1cccc(-c2n[nH]cc2C=O)c1
2	TGTGCCAAC	CCCn1cc(C=O)c(C2CC2)n1
2	AGACGCTAC	CNC(=O)c1ccc(C=O)cc1
2	CGCTCATAC	O=Cc1cnn(-c2ccccn2)c1
2	GGAGCATAC	Cc1ccncc1CCC=O
2	TCTTGCGAC	O=Cc1ccc(-n2ccnc2)nc1
2	AGATGCGAC	O=Cc1ccc(-c2cccnc2)o1
2	ATGGCGAAC	N#CCCCOc1cccc(C=O)c1
2	GCACTCAAC	CCOc1cc(C=O)ccc1OC
2	TGTGTCCAC	O=CC1CCCCCCC1
2	AGCCGATAC	CCCn1ccc(C=O)n1
2	CGTAGGAAC	COc1nccc(C=O)n1
2	GGATCTGAC	O=CCC1CCOCC1
2	TGAGGACAC	O=Cc1cn2c(n1)CCC2
2	AGCATGGAC	O=Cc1cc(C(=O)O)ccc1O
2	CACGTTGAC	O=CC(=O)O
2	GCATGAGAC	COc1cccnc1C=O

Cycle	Tag (5'-3')	Smiles
2	TGTTGCCAC	C#Cc1cccc(C=O)c1
2	AGTCGTCAC	O=Cc1ccnc(O)c1
2	CGTCAAGAC	O=Cc1ccc2c(c1)CCC2
2	GGCTATGAC	O=Cc1cn(-c2ccccc2)nn1
2	TGCGGTAAC	O=Cc1cnn[nH]1
2	AGCGTGAAC	O=CC[C@@H](O)[C@H](O)[C@H](O)CO
2	CAGTCCAAC	O=CC1CCC1
2	GCTTACCAC	CC(=O)OCc1ccc(C=O)o1
2	TTGGCTGAC	CCCC=O
2	ATCGAGGAC	O=C[C@H](O)CO
2	CGTCACAAC	O=CCOCc1ccccc1
2	GTCAGGAAC	O=CC[C@@H](O)[C@@H](O)CO
2	TTCGTGGAC	COC(=O)CCC=O
2	AGGAACCAC	O=CC[C@H](O)[C@H](O)CO
2	CATCGCTAC	O=Cc1ccccc1-n1cccc1
2	GGAGTCAAC	COc1ncccc1C=O
2	TTGGTGGAC	Cn1cc(C=O)cn1
2	CACTTCGAC	Cc1cccc(C=O)n1
2	CGTCCTTAC	O=Cc1cc2cc[nH]c2cn1
2	GTCGTGAAC	O=Cc1cccc(-c2nn[nH]n2)c1
2	AACTGGCAC	O=Cc1ccc(-c2nn[nH]n2)cc1
2	AGGTAGCAC	COc1nc(C)ccc1C=O
2	CGAAGTCAC	O=CC1CC1
2	GGTCCTAAC	CC(C)(CC=O)C1CC1
2	AAGCACCAC	O=CC1CCCCCC1
2	CAGTTCCAC	CC(C)(C)c1ccc(CC=O)cc1
2	CGTCGTAAC	CC(C)(O)c1ccc(C=O)cc1
2	GTGGTAGAC	O=CC1CC2(CC2)C1
2	AATCGCCAC	COC(C)(C)CC=O
2	AGGTGAGAC	O=Cc1ccc2c(n1)NC(=O)CO2
2	CGAATGCAC	Cc1cc(C)c(C(=O)O)cc1C=O
2	GTTGGAGAC	O=Cc1cccc2ccoc12
2	AAGGCACAC	O=Cc1csnn1
2	CGAGAAGAC	NS(=O)(=O)c1ccc(C=O)cc1
2	CTGCGATAC	O=S(=O)(Cl)c1cc(F)ccc1F

Cycle	Tag (5'-3')	Smiles
2	GTTGAGCAC	COc1cc(C)c(Cl)cc1S(=O)(=O)Cl
2	ACAGGCTAC	O=C1COc2cc(S(=O)(=O)Cl)ccc2N1
2	AGGTGCTAC	Cc1noc(C)c1S(=O)(=O)Cl
2	CGAGTGAAC	Cc1ccc(S(=O)(=O)Cl)cc1
2	TCAGGTCAC	O=S(=O)(Cl)c1cc(F)cc(F)c1
2	ACCTCAGAC	O=S(=O)(Cl)c1cc(Cl)cc(Cl)c1
2	CGAGACTAC	O=[N+]([O-])c1ccc(S(=O)(=O)Cl)c(Cl)c1
2	CTTGGCAAC	O=S(=O)(Cl)c1ccc(F)c(Cl)c1
2	TAGCTGCAC	O=c1ccc2ccc(S(=O)(=O)Cl)cc2[nH]1
2	ACCATCCAC	O=c1[nH]c(=O)c2cc(S(=O)(=O)Cl)cnc2[nH]1
2	AGTAGGCAC	O=c1[nH]c2ccc(S(=O)(=O)Cl)cc2s1
2	CGTTCTGAC	O=C(N1CCc2ccc(S(=O)(=O)Cl)cc2C1)C(F)(F)F
2	TCGGTGAAC	C#CCOc1cccc(S(=O)(=O)Cl)c1
2	ACGGTAGAC	Cc1ccncc1S(=O)(=O)Cl
2	CGAGGTTAC	C=C(C)COc1cccc(S(=O)(=O)Cl)c1
2	GCACTGAAC	N#Cc1ccc(S(=O)(=O)Cl)cc1
2	TAGGCACAC	O=S(=O)(Cl)c1cc(F)cc(Cl)c1
2	ACGCAAGAC	O=C(O)c1ccc(S(=O)(=O)Cl)c([N+](=O)[O-])c1
2	AGTTGCGAC	Cn1nncc1S(=O)(=O)Cl
2	CGTTGACAC	CCc1ccc(S(=O)(=O)Cl)cc1
2	TCTCCGAAC	O=Cc1ccc(S(=O)(=O)Cl)cc1
2	AGAAGGCAC	Cn1ccnc1S(=O)(=O)Cl
2	CGCAGATAC	Nc1ncc(S(=O)(=O)Cl)cn1
2	GCTACAGAC	O=S(=O)(Cl)c1nnc[nH]1
2	TCATGGCAC	COc1cccc(S(=O)(=O)Cl)c1
2	ACGCCTTAC	CC(C)n1cnc(S(=O)(=O)Cl)c1
2	ATCCGACAC	Cc1ccccc1S(=O)(=O)Cl
2	CTTAGCCAC	Cc1c(S(=O)(=O)Cl)cnn1C
2	TGAGCTGAC	COc1ccccc1S(=O)(=O)Cl
2	AGACTGCAC	CCn1cc(S(=O)(=O)Cl)cn1
2	CGCATCTAC	O=S(=O)(Cl)c1cnc2n1CCC2
2	GGAACGAAC	N#Cc1ncccc1S(=O)(=O)Cl
2	TCCGTAGAC	Cn1cnc(S(=O)(=O)Cl)c1
2	ACTTCCGAC	N#Cc1ccc(S(=O)(=O)Cl)cn1
2	ATCGCTGAC	CCn1cnc(S(=O)(=O)Cl)c1

Cycle	Tag (5’-3’)	Smiles
2	CTTCAGCAC	N#Cc1cccc(S(=O)(=O)Cl)c1
2	TGGATCGAC	COc1cncc(S(=O)(=O)Cl)c1
2	AGGACCAAC	CCn1cc(S(=O)(=O)Cl)nc1C
2	AGGACTCAC	NS(=O)(=O)c1ccc(S(=O)(=O)Cl)cc1
2	AGGAGCAAC	Cc1nc(S(=O)(=O)Cl)cn1C
2	AGTCTCGAC	CC(C)n1cc(S(=O)(=O)Cl)cn1
2	AGTGGTGAC	Cc1cnc(N)c(S(=O)(=O)Cl)c1
2	ATCACGCAC	COc1ccc(S(=O)(=O)Cl)cn1
2	ATCGGTGAC	COc1ccc(S(=O)(=O)Cl)cc1
2	ATGCGACAC	CCn1ncc(S(=O)(=O)Cl)c1C
3	CAACGAGTT	COC(=O)c1cc(B2OC(C)(C)C(C)(C)O2)ccc1C
3	TACTCGGTT	CC1(C)OB(c2ccc(S(C)(=O)=O)cc2)OC1(C)C
3	TGGAGCATT	Cc1ccc(C(=O)O)cc1B1OC(C)(C)C(C)(C)O1
3	GAGTCTGTT	OB(O)c1ccc(Cl)cc1
3	CAATCCGTT	COc1cccc(B(O)O)c1OC
3	GACTCTCTT	CC1(C)OB(c2cccc(S(N)(=O)=O)c2)OC1(C)C
3	TATCGCCTT	CN(C)CCc1ccc(B(O)O)cc1
3	TGGATGCTT	Cn1nnc2ccc(B3OC(C)(C)C(C)(C)O3)cc21
3	GTCGTCATT	CC1(C)OB(c2cccc3ccoc23)OC1(C)C
3	CAGAAGGTT	COC(=O)c1cc(B2OC(C)(C)C(C)(C)O2)ccc1O
3	GGCATGTTT	CC1(C)Cc2cc(B3OC(C)(C)C(C)(C)O3)ccc2O1
3	CGACTCTTT	Cc1cc2cc(B3OC(C)(C)C(C)(C)O3)ccc2[nH]1
3	GCAGATGTT	CC1(C)OB(c2ccc(C3(CO)CC3)cc2)OC1(C)C
3	CAGGAAGTT	Cc1nc2ccc(B3OC(C)(C)C(C)(C)O3)cc2o1
3	TCACACGTT	CC1(C)OB(c2cccc3c2CCO3)OC1(C)C
3	CAACAGCTT	CC(C)OCc1cccc(B(O)O)c1
3	CGTCGAATT	COC(=O)c1ccc(B2OC(C)(C)C(C)(C)O2)o1
3	GTGCCAATT	OCc1ccc(B(O)O)cc1Cl
3	CAGTCGTTT	CC1(C)OB(c2cnc3ccnn3c2)OC1(C)C
3	CACCGTATT	COc1ccc(B2OC(C)(C)C(C)(C)O2)cn1
3	CGTCTCATT	CC1(C)OB(c2ccc3ccc(=O)[nH]c3c2)OC1(C)C
3	GCTAACCTT	CC1(C)OB(c2ccc3c(c2)CCCNC3=O)OC1(C)C
3	GTGCGTATT	OB(O)c1cccc2c1OCCCO2
3	GTGAGGTTT	CC(C)c1nn(C)cc1B1OC(C)(C)C(C)(C)O1
3	CAGTCTCTT	CC1(C)OB(c2cccc(Cn3cccn3)c2)OC1(C)C

Cycle	Tag (5'-3')	Smiles
3	GTGGCTATT	COc1cc(CO)ccc1B1OC(C)(C)C(C)(C)O1
3	GCACAGTTT	CC1(C)OB(c2ccc(C3(N)CCCC3)cc2)OC1(C)C
3	TCTGGTGTT	OCc1cccc(B(O)O)c1
3	CCACAACTT	CC1(C)OB(c2cccc(O)c2)OC1(C)C
3	GCTGTAGTT	CC1(C)OB(c2ccc(C3CC(=O)C3)cc2)OC1(C)C
3	GCAGTCATT	CC1(C)OB(c2cccc3c2CNC3=O)OC1(C)C
3	TGAGGTCTT	COc1cc(B(O)O)ccc1Cl
3	CCACTCATT	CC1(C)OB(c2cccc(CN)c2)OC1(C)C
3	CTCCGATTT	CC1(C)OB(c2ccc3c(c2)COC(=O)N3)OC1(C)C
3	CCTTGCTTT	OB(O)c1ccc(F)nc1
3	GCCAGTATT	CC1(C)OB(c2cnc(N)nc2)OC1(C)C
3	GTGTCGTTT	Cn1c(=O)[nH]c2ccc(B3OC(C)(C)C(C)(C)O3)cc21
3	TGATCGGTT	CC1(C)OB(c2ccc([C@@H]3CCC(=O)N3)cc2)OC1(C)C
3	CTCTCCTTT	Cc1cc(F)ccc1B(O)O
3	TACCGAGTT	CC(C)(C)n1cc(B2OC(C)(C)C(C)(C)O2)cn1
3	CGCTGTATT	Cc1cccc(B(O)O)c1C
3	GTTCTCCTT	Cc1ccc2cc(B(O)O)ccc2n1
3	CCATAGGTT	COc1ncc(B2OC(C)(C)C(C)(C)O2)cn1
3	GCTAAGGTT	Cc1ccc(C(N)=O)cc1B1OC(C)(C)C(C)(C)O1
3	TGCGACTTT	CN(C)Cc1cc(B(O)O)ccc1F
3	CTGTGAGTT	CNC(=O)c1ccc(B2OC(C)(C)C(C)(C)O2)cc1C
3	TCCAACGTT	CC(=O)c1cccc(B(O)O)c1
3	GTTGGTCTT	CC1(C)OB(c2cnn(-c3ccncc3)c2)OC1(C)C
3	TGGACCTTT	CNC(=O)Nc1ccc(B2OC(C)(C)C(C)(C)O2)cc1
3	CCGCAATTT	OB(O)c1ccc2c(c1)CCC2
3	CTGTGCTTT	CN(C)c1cc(B2OC(C)(C)C(C)(C)O2)ccn1
3	TCGGTAGTT	OB(O)c1ccc(F)c(Cl)c1
3	CGAGAACTT	OB(O)c1cccc(Cl)c1F
3	GGAACCTTT	CC1(C)OB(c2cc3ccccc3o2)OC1(C)C
3	TAGCAGGTT	COCCc1cccc(B2OC(C)(C)C(C)(C)O2)c1
3	CAACCACTT	Cc1ccc(Cl)cc1B(O)O
3	CGACCTATT	COc1ncc(Cl)cc1B(O)O
3	GAGTTCGTT	NC(=O)c1ccc(B(O)O)cc1
3	GTCCACATT	O=C(O)c1cc(B(O)O)ccc1Cl
3	TCGTTGGTT	CN(C)c1ncc(B2OC(C)(C)C(C)(C)O2)cc1Cl

Cycle	Tag (5'-3')	Smiles
3	CGATTGCTT	C[C@@H]1Cc2cc(B(O)O)ccc2O1
3	TAGCGCATT	Cc1c(Cl)cccc1B(O)O
3	GCAACAGTT	OB(O)c1cccc2c1OCC2
3	TCTGCGTTT	COc1ccc(B(O)O)cc1C#N
3	GGTACAGTT	CC1(C)OB(c2ccc3c(c2)CCC(=O)N3)OC1(C)C
3	TCATCGCTT	CC1(C)OB(c2ccc3c(c2)CCOC3)OC1(C)C
3	CAGACCATT	CC1(C)OB(c2cccc(O)c2F)OC1(C)C
3	GCAAGACTT	OB(O)c1ccc2c(c1)CCCO2
3	GTGGTGTTT	COc1ccc(B(O)O)cc1F
3	TGCCAAGTT	COC(=O)c1cc(O)cc(B2OC(C)(C)C(C)(C)O2)c1
3	GGTGAAGTT	COc1ncc(B2OC(C)(C)C(C)(C)O2)cc1C
3	CAGGACATT	CC1(C)OB(C2=CCNCC2)OC1(C)C
3	TGCTTGCTT	OB(O)c1ccc2c(c1)OCO2
3	GGTTAGGTT	Nc1cccc(B(O)O)c1
3	TCGTGGTTT	COC(=O)c1ccc(B2OC(C)(C)C(C)(C)O2)cc1F
3	CTACGACTT	OB(O)c1cncnc1
3	GCGGTATTT	CC(C)(O)c1ccc(B2OC(C)(C)C(C)(C)O2)c(F)c1
3	GTTGCTGTT	C[C@H](O)c1ccc(B(O)O)cc1
3	CAACTCGTT	CC1(C)OB(c2cnc3occc3c2)OC1(C)C
3	CGTAGAGTT	Nc1cc(B(O)O)ccc1Cl
3	GTGGAACTT	CCN(CC)c1ncc(B(O)O)cn1
3	TCTCGACTT	OB(O)c1ccc2ccc(O)cc2c1
3	CAGTCAGTT	COc1cccc2cc(B3OC(C)(C)C(C)(C)O3)oc12
3	CTCATCCTT	CCn1ccc(B2OC(C)(C)C(C)(C)O2)n1
3	GCGTTCTTT	OB(O)c1cccc2ccccc12
3	TACGTCCTT	COC(=O)CCc1cccc(B(O)O)c1
3	CAAGGCTTT	COc1ccc(C)cc1B(O)O
3	GTGGTTCTT	CCc1ccccc1B(O)O
3	TGAGCAGTT	OCc1ccc(B(O)O)cc1
3	CAGTGTGTT	OB(O)c1cccc(Cl)c1
3	CTCATGGTT	COc1ccc2cc(B(O)O)ccc2c1
3	TAGCTCCTT	CC(C)Oc1ccc(B(O)O)cc1F
3	CTCGCAATT	COC(=O)c1cccc(B(O)O)c1
3	GTTGCACTT	OB(O)c1ccc(Cl)c(F)c1
3	TGCCTTCTT	CCCc1ccc(B(O)O)cc1

Cycle	Tag (5’-3’)	Smiles
3	CATCCTGTT	O=C(O)CCc1ccc(B(O)O)cc1
3	CTGAGCATT	COc1ccc(B(O)O)cc1C(=O)O
3	TCACCAGTT	CC(C)(C)c1ccc(B(O)O)cc1
3	CCACACTTT	OB(O)c1ccc(Oc2ccccc2)cc1
3	CTGCCTATT	COc1ccc(F)c(B(O)O)c1
3	TACAGCGTT	CCOc1ccc(B(O)O)cc1C
3	TGGAGGTTT	OB(O)c1cccc(OC(F)(F)F)c1
3	GAATGCGTT	CC(C)Cc1ccc(B(O)O)cc1
3	GGCATCATT	OB(O)c1cc(F)c(F)c(F)c1
3	CCTAGTGTT	Cc1cc(C)cc(B(O)O)c1
3	CCATACCTT	OB(O)c1ccc2ccccc2c1
3	GACACAGTT	CC(C)(C)c1cccc(B(O)O)c1
3	GTAGAGGTT	O=C(O)c1ccc(B(O)O)c(F)c1
3	CCTTCTGTT	O=C(O)c1ccc(B(O)O)c(Cl)c1
3	TGTCCAGTT	CCOc1ccc(B(O)O)cc1
3	GACCTGTTT	CCCOc1cccc(B(O)O)c1
3	GTAGGCTTT	CCOc1cc(F)ccc1B(O)O
3	TCCACTGTT	Cc1ccc(B(O)O)c(Cl)c1
3	CGACGATTT	Cn1nccc1B(O)OC(C)(C)C(C)(C)O
3	GCGTAGTTT	OB(O)c1cccc(-c2ccccc2)c1
3	CCGTCAATT	COc1ccc(C(C)C)cc1B(O)O
3	GACTCGTTT	OCc1ccccc1B(O)O
3	GTATCGCTT	OB(O)c1ccc2cc[nH]c2c1
3	TCTGCTCTT	CCc1ccc(B(O)O)cc1
3	TGCTCCTTT	OB(O)/C=C/C1CCCCC1
3	GAACTCCTT	N#Cc1cc(B(O)O)ccc1Cl
3	GCTCTCATT	Cc1ccc(OC(C)C)c(B(O)O)c1
3	TCCGATGTT	COc1c(C)cc(B(O)O)cc1C
3	CGCTATCTT	OB(O)c1cccc(F)c1
3	GAGCTTCTT	CCc1cccc(B(O)O)c1
3	GTTCCAGTT	O=C(NC1CC1)c1cccc(B(O)O)c1
3	CCGTGATTT	CCOc1ncccc1B(O)O
3	GACGATGTT	O=C(O)c1cc(Cl)cc(B(O)O)c1
3	GGAAGTCTT	CC(C)c1cccc(B(O)OCCO)c1
3	TCGCTTCTT	CCOC(=O)c1cccc(B(O)O)c1

Cycle	Tag (5'-3')	Smiles
3	CGCTGATTT	Cc1cccc(B(O)O)c1
3	GAGTCCATT	COc1ccc(Cl)cc1B(O)O
3	TAACCGCTT	COc1cc(F)c(F)cc1B(O)O
3	TGGTGAGTT	COC(=O)Nc1ccc(B(O)O)cc1
3	CCTGTCTTT	OB(O)c1cc(F)cc(Cl)c1
3	GAGGTAGTT	CCOc1ccccc1B(O)O
3	GGAGTAGTT	O=C(O)c1cccc(B(O)O)c1
3	TCGGACATT	CS(=O)(=O)c1ccc(B(O)O)cc1
3	CGGAGAATT	OB(O)c1ccc(C(F)(F)F)cc1
3	GATAGCGTT	Nc1cc(B(O)O)ccc1F
3	TATGCCGTT	COC(=O)c1cc(B(O)O)ccc1Cl
3	CACCAAGTT	O=C(O)Cc1cccc(B(O)O)c1
3	CGAGGTATT	OB(O)c1ccc(F)c(C(F)(F)F)c1
3	GGTGTTGTT	Cc1cc(Cl)ccc1B(O)O
3	TGCGTCATT	OB(O)c1ccc(Cl)c(Cl)c1
3	CGGATACTT	OB(O)c1cc(Cl)cc(Cl)c1
3	GCAGAACTT	O=C(O)c1cccc(B(O)O)c1F
3	TCACAGCTT	COc1cc(B(O)O)cc(C(=O)O)c1
3	CACGACTTT	OB(O)c1cccc(Oc2ccccc2)c1
3	CGCTCAATT	CC(C)(C)[C@H]1CC=C(B(O)O)CC1
3	GATGTCCTT	OB(O)c1cccc2c1OCCO2
3	GTCATGCTT	Cc1cc(B(O)O)ccc1OC(C)C
3	TGCTAGGTT	OCCc1cccc(B(O)O)c1
3	CGGTAGTTT	N#Cc1ccc(B(O)O)cc1F
3	GCATGGTTT	OB(O)c1ccc(C(F)(F)F)c(F)c1
3	TCAGAGGTT	COC(=O)c1ccc(B(O)O)c(F)c1
3	CGGCTAATT	OB(O)c1ccc2c(c1)OC(F)(F)O2
3	GATTGCCTT	Cc1ccc(F)cc1B(O)O
3	CTCTTGCTT	OB(O)c1cc(F)cc(C(F)(F)F)c1
3	GGATGAGTT	Cc1cc(B(O)O)ccc1Cl
3	TCCTGTGTT	COC(=O)c1cc(Cl)cc(B(O)O)c1
3	CGTACTCTT	COc1ncc(B(O)O)cc1Cl
3	GTGTGTCTT	OB(O)c1cc(F)cc(F)c1
3	CAGGTTGTT	Cc1cc(B(O)O)c(C)cc1F
3	CTTCCTCTT	Cc1ccc(B(O)O)cc1

Cycle	Tag (5'-3')	Smiles
3	GGTAGACTT	COc1ccc(B(O)O)cc1C
3	TCGTTCCTT	COc1cc(B(O)O)cc(OC)c1OC
3	CATAGGCTT	Cc1ccc(B(O)O)c(F)c1
3	CGTGACTTT	CCOc1cccc(B(O)O)c1
3	GCAATCGTT	COc1cc(F)cc(B(O)O)c1
3	CATCGGTTT	CCN(CC)Cc1ccc(B(O)O)cc1
3	CTTCTCGTT	CC(=O)Nc1cccc(B(O)O)c1
3	GGTGACATT	O=C(O)COc1cc(F)cc(B(O)O)c1
3	TGACGCATT	CCNC(=O)c1cccc(B(O)O)c1
3	CATCGACTT	COc1ccc(C#N)cc1B(O)O
3	CTAAGCGTT	CNC(=O)c1ccc(B(O)O)cc1
3	GCAGGATTT	COc1cc(OC)cc(B(O)O)c1
3	TACCACCTT	CC(C)(C)OCc1ccc(B(O)O)cc1
3	CCAACACTT	CCCCc1ccc(B(O)O)cc1
3	GTAACGGTT	CCNC(=O)c1ccc(B(O)O)cc1
3	TGAGTGCTT	O=C(O)c1ccc(B(O)O)cc1Cl
3	CCACCATTT	COc1cc(C(=O)O)ccc1B(O)O
3	GCCTAACTT	O=C(O)c1ccc(B(O)O)cc1F
3	TACGAGGTT	CC(C)Oc1ccc(F)cc1B(O)O
3	CCAACCTTT	Cc1cc(B(O)O)ccc1F
3	GAACGCTTT	COc1ccc(B(O)O)cc1Cl
3	GTCTACCTT	CC(C)NC(=O)c1cccc(B(O)O)c1
3	TGCCAGTTT	CCCOc1ccc(B(O)O)cc1Cl
3	CCGAACATT	COC(=O)c1cccc(B(O)O)c1F
3	CTCACTGTT	OB(O)c1ccc(-c2ccccc2)cc1
3	GCTCCTATT	OB(O)c1ccccc1
3	TAGGACCTT	CC(=O)c1ccc(B(O)O)cc1
3	CCACGTATT	COc1ncccc1B(O)O
3	GACACCATT	OCc1ccc(Cl)c(B(O)O)c1
3	GTCTCAGTT	O=C(O)CCc1cccc(B(O)O)c1
3	TGCTCTCTT	CONC(=O)c1ccc(B(O)O)cc1
3	CCGAATGTT	CCCNC(=O)c1cccc(B(O)O)c1
3	GCTCTACTT	COC(=O)c1cc(F)cc(B(O)O)c1
3	CCATCAGTT	CCOC(=O)c1ccc(B(O)O)cc1F
3	GAGCAGTTT	Cc1ccc(B(O)O)cc1Cl

Cycle	Tag (5'-3')	Smiles
3	GTGTTGCTT	COc1cc(B(O)O)ccc1C
3	TGTAGCCTT	OB(O)c1ccc(OC(F)(F)F)cc1
3	TGTGCTGTT	COC(=O)Cc1ccc(B(O)O)cc1
3	TGTGGAGTT	COc1ccc(B(O)O)cc1OC(C)C
3	TGTGGCTTT	NS(=O)(=O)c1ccc(B(O)O)cc1
3	TGTGTCGTT	Null-reaction; heated with Pd 'catalyst'
3	CACGGAATT	Null-reaction; not heated without Pd 'catalyst'

10. Supplementary References

1. Maetani, M. *et al.* Synthesis of a bicyclic azetidine with in vivo antimalarial activity enabled by stereospecific, directed C(sp^3)-H arylation. *J. Am. Chem. Soc.* **139**, 11300−11306 (2017).

Uncropped Gel Images

Uncropped gel images for Supplementary Fig. 12

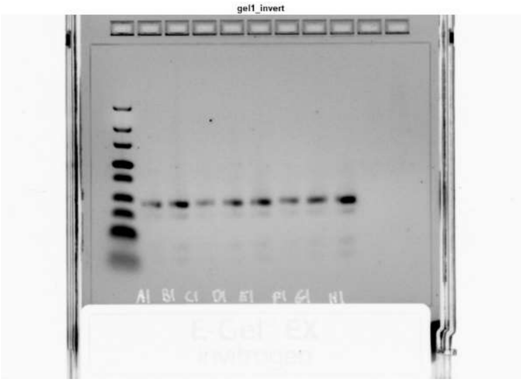

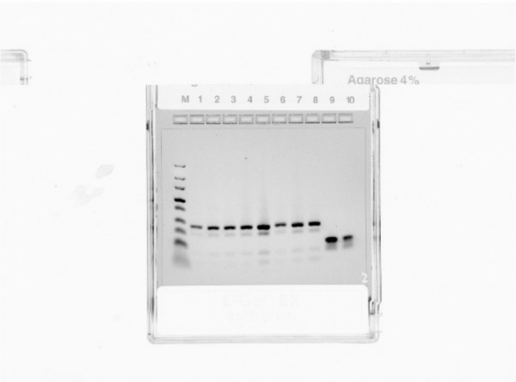

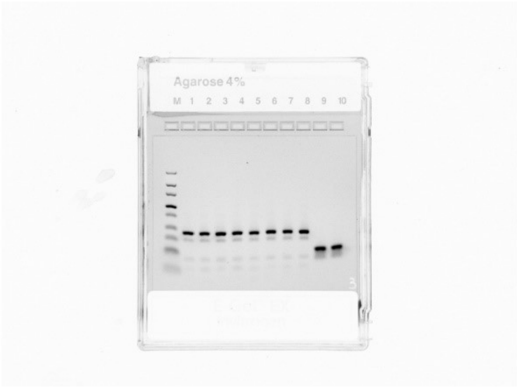

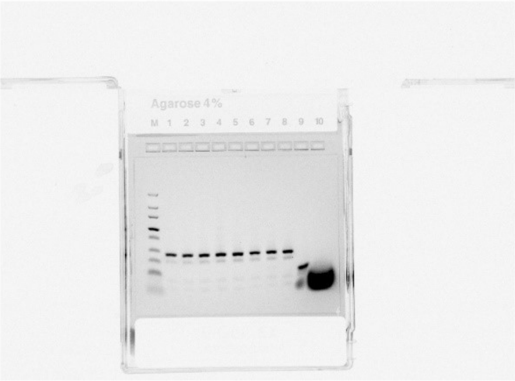

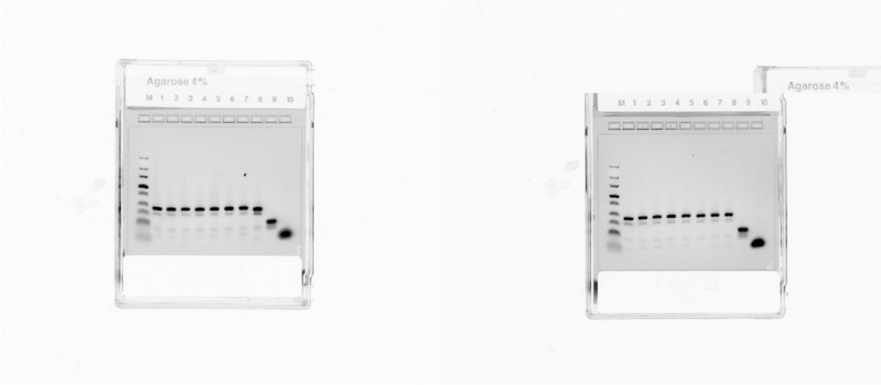

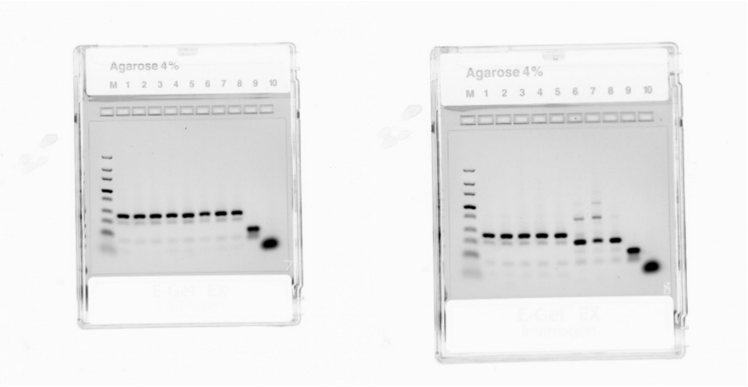

Uncropped gel images for Supplementary Fig. 13

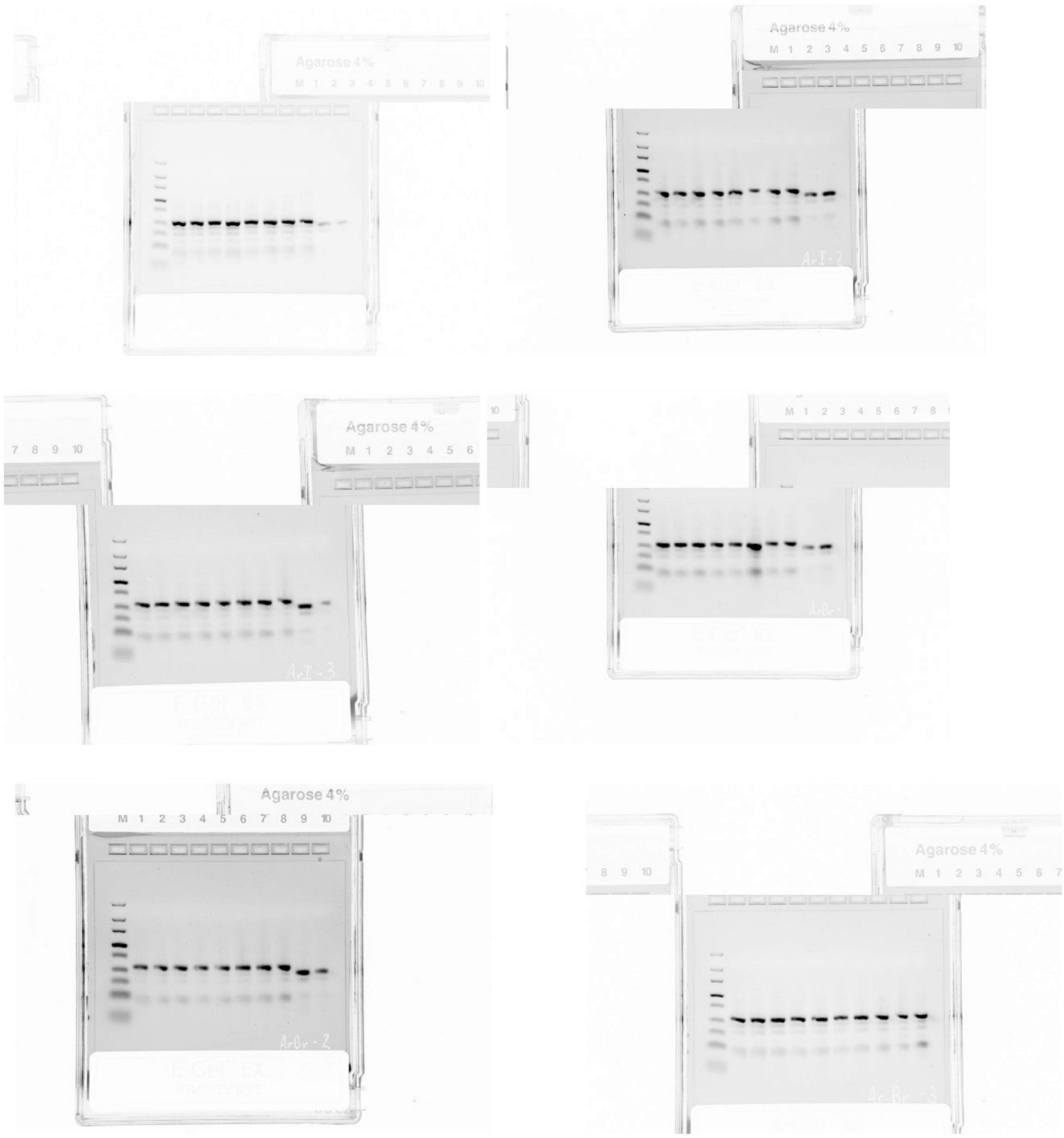

Uncropped gel images for Supplementary Fig. 14

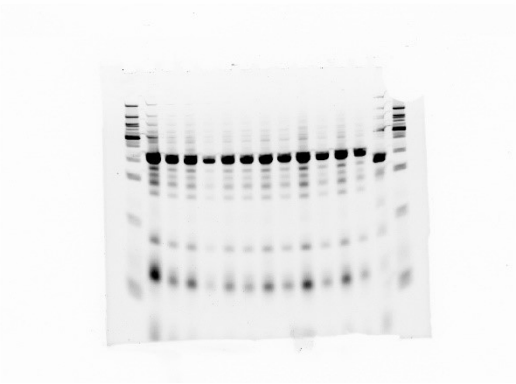

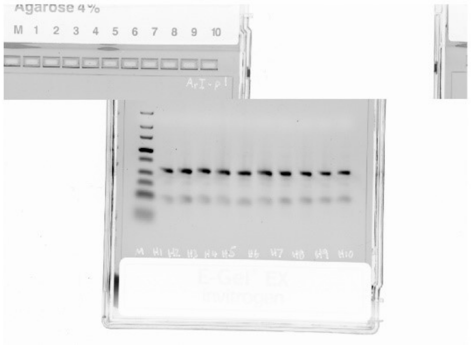

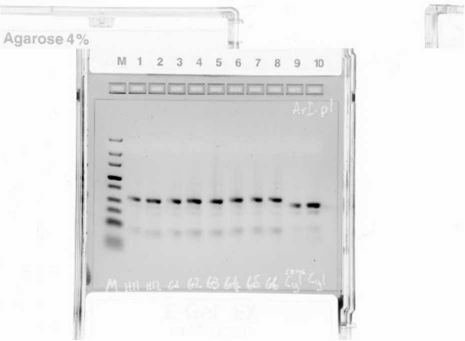

Uncropped gel images for Supplementary Fig. 15

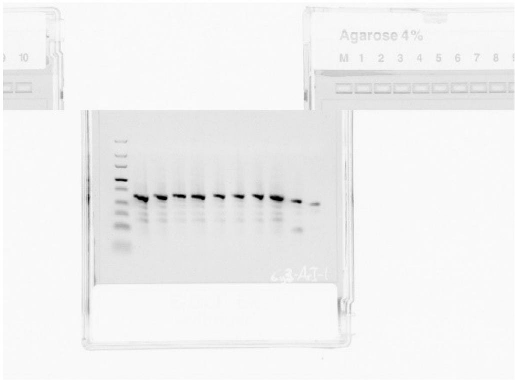

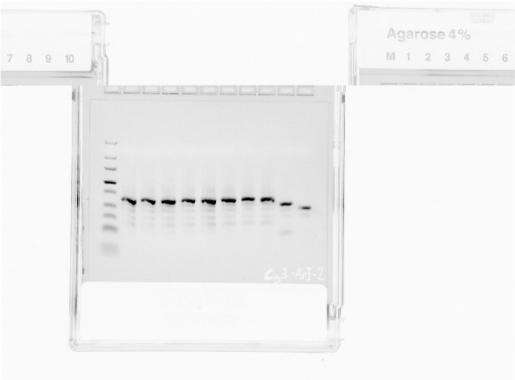

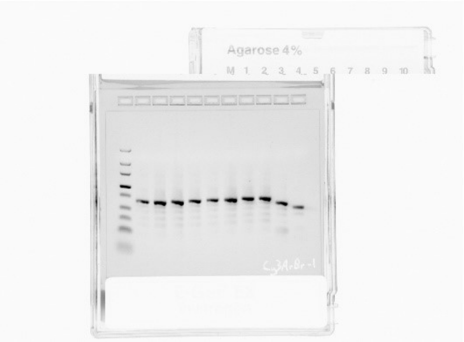

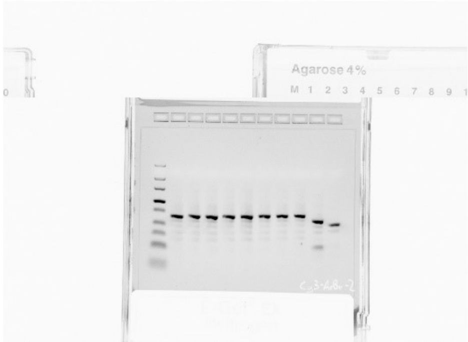

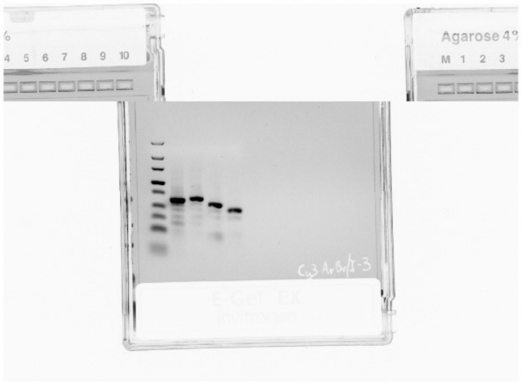

Uncropped gel images for Supplementary Fig. 16

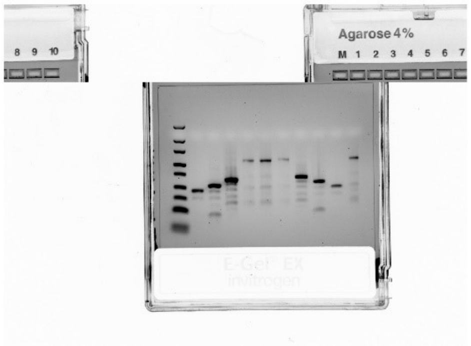

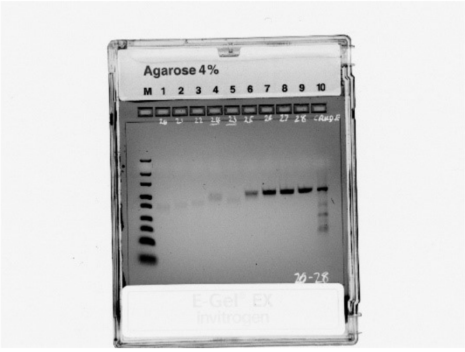

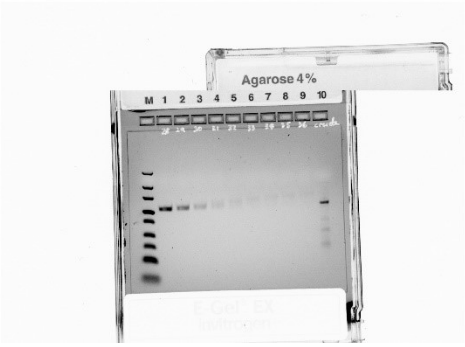

Uncropped gel images for Supplementary Fig. 17

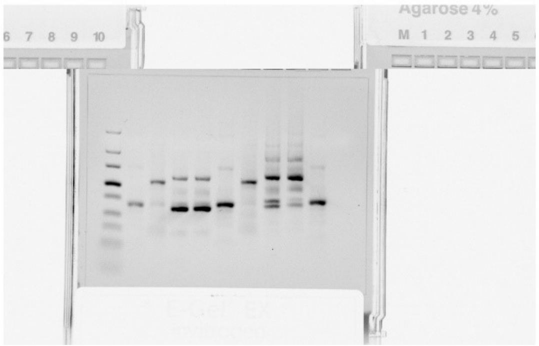

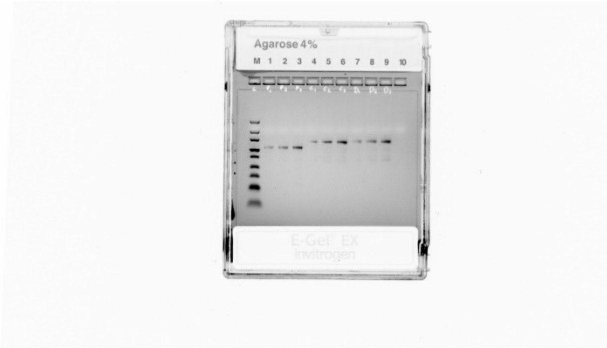

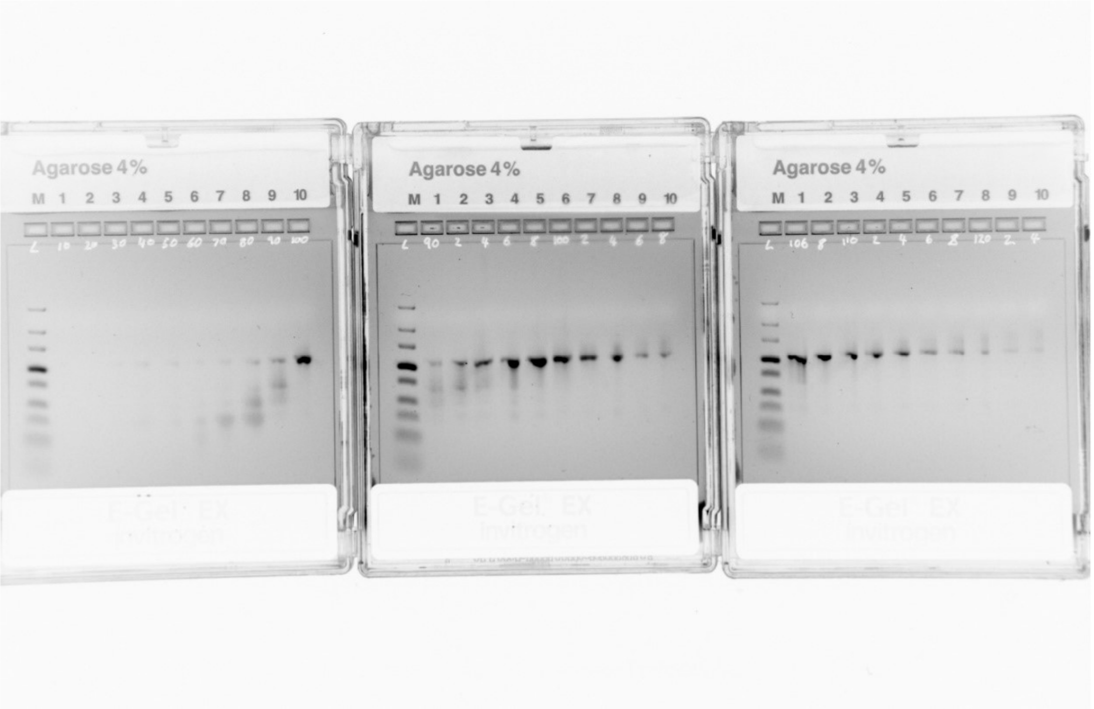

Agarose 4%
M 1 2 3 4 5 6 7 8 9 10
Agarose 4%
M 1 2 3 4 5 6 7 8 9 10
Agarose 4%
M 1 2 3 4 5 6 7 8 9 10
