# Supplementary material for: Diversity-oriented synthesis encoded by deoxyoligonucleotides
Source: Nat Commun. 2023 Aug 15;14:4930. doi: 10.1038/s41467-023-40575-5 (PMC10427684; doi:10.1038/s41467-023-40575-5)
Supplement: Supplementary file 2 — Reporting Summary [file 41467_2023_40575_MOESM2_ESM.pdf]

nature portfolio

Corresponding author(s): Dr. Frederic Zecri

Last updated by author(s): 07/14/2023

Reporting Summary

Nature Portfolio wishes to improve the reproducibility of the work that we publish. This form provides structure for consistency and transparency in reporting. For further information on Nature Portfolio policies, see our Editorial Policies and the Editorial Policy Checklist.

Statistics

For all statistical analyses, confirm that the following items are present in the figure legend, table legend, main text, or Methods section.

n/a	Confirmed	
☐	☒	The exact sample size (n) for each experimental group/condition, given as a discrete number and unit of measurement
☐	☒	A statement on whether measurements were taken from distinct samples or whether the same sample was measured repeatedly
☐	☒	The statistical test(s) used AND whether they are one- or two-sided<br>*Only common tests should be described solely by name; describe more complex techniques in the Methods section.*
☐	☒	A description of all covariates tested
☐	☒	A description of any assumptions or corrections, such as tests of normality and adjustment for multiple comparisons
☐	☒	A full description of the statistical parameters including central tendency (e.g. means) or other basic estimates (e.g. regression coefficient) AND variation (e.g. standard deviation) or associated estimates of uncertainty (e.g. confidence intervals)
☐	☒	For null hypothesis testing, the test statistic (e.g. F, t, r) with confidence intervals, effect sizes, degrees of freedom and P value noted<br>*Give P values as exact values whenever suitable.*
☒	☐	For Bayesian analysis, information on the choice of priors and Markov chain Monte Carlo settings
☒	☐	For hierarchical and complex designs, identification of the appropriate level for tests and full reporting of outcomes
☒	☐	Estimates of effect sizes (e.g. Cohen's d, Pearson's r), indicating how they were calculated

Our web collection on statistics for biologists contains articles on many of the points above.

Software and code

Policy information about availability of computer code

Data collection	NMR spectroscopy: ICON-NMR under TopSpin program control and processed with MestReNova (Version 12.0.2-20910).<br>HPLC: Agilent OpenLab CDS (C.01.07 SR1)<br>Gel imaging: BioRad Imager
Data analysis	Surface Plasmon Resonance: affinity mode of the Biacore T200 Evaluation Software<br>Differential Scanning Fluorimetry: Protein Thermal Shift Software v1.3 (Applied Biosystems) was used to determine the melting temperature, Tm, from the derivative of the melt curve.<br>Structure determination: data were integrated using the Bruker SAINT Software program and scaled using the SADABS software program.<br>Library design and determination: Knime Analytics Platform<br>Assay dose response curves: Dose-response curves were fitted and IC50 values and 95% confidence intervals were calculated using GraphPad GraphPad Prism v9.2.0. Outliers were detected and removed via GraphPad Prism's ROUT method.<br>Enzymatic inhibition: Percent residual activities at 11 compound concentrations were fitted using GraphPad Prism to obtain the IC50 value<br>Gel Image Analysis: Bio-Rad Image Lab

For manuscripts utilizing custom algorithms or software that are central to the research but not yet described in published literature, software must be made available to editors and reviewers. We strongly encourage code deposition in a community repository (e.g. GitHub). See the Nature Portfolio guidelines for submitting code & software for further information.

Data

Policy information about availability of data

All manuscripts must include a data availability statement. This statement should provide the following information, where applicable:

- Accession codes, unique identifiers, or web links for publicly available datasets
- A description of any restrictions on data availability
- For clinical datasets or third party data, please ensure that the statement adheres to our policy

The datasets generated and/or analyzed during the current study are available from the corresponding author upon request. Source data and Supplementary Information files are provided with this paper. The SMILES strings data generated in this study are deposited in the Zenodo database under accession code ZZ [add hyperlink here]. X-ray crystallographic coordinates for structures reported in this study have been deposited at the Cambridge Crystallographic Data Centre (CCDC), under deposition number 2069871. These data can be obtained free of charge from The Cambridge Crystallographic Data Centre via www.ccdc.cam.ac.uk/data_request/cif.

Human research participants

Policy information about studies involving human research participants and Sex and Gender in Research.

Reporting on sex and gender	N/A
Population characteristics	N/A
Recruitment	N/A
Ethics oversight	N/A

Note that full information on the approval of the study protocol must also be provided in the manuscript.

Field-specific reporting

Please select the one below that is the best fit for your research. If you are not sure, read the appropriate sections before making your selection.

☒ Life sciences ☐ Behavioural & social sciences ☐ Ecological, evolutionary & environmental sciences

For a reference copy of the document with all sections, see nature.com/documents/nr-reporting-summary-flat.pdf

Life sciences study design

All studies must disclose on these points even when the disclosure is negative.

Sample size	No biological samples were taken, so this is not relevant to our study.
Data exclusions	No data were excluded from the analysis.
Replication	At least 3 technical replicates were measured whenever possible.
Randomization	This is not relevant to our study, as every compound generated in the DOSEDO library is unique. No biological samples or groups were used in this study.
Blinding	This is not relevant to our study because there was no group allocation.

Reporting for specific materials, systems and methods

We require information from authors about some types of materials, experimental systems and methods used in many studies. Here, indicate whether each material, system or method listed is relevant to your study. If you are not sure if a list item applies to your research, read the appropriate section before selecting a response.

Materials & experimental systems

n/a	Involved in the study
☒	☐ Antibodies
☒	☐ Eukaryotic cell lines
☒	☐ Palaeontology and archaeology
☒	☐ Animals and other organisms
☒	☐ Clinical data
☒	☐ Dual use research of concern

Methods

n/a	Involved in the study
☒	☐ ChIP-seq
☒	☐ Flow cytometry
☒	☐ MRI-based neuroimaging
